# Causal associations between lifestyle factors and hemorrhoidal disease: Insights from Mendelian randomization analysis

**DOI:** 10.1097/MD.0000000000048945

**Published:** 2026-05-22

**Authors:** Jinqiu Xiong, Yuanyuan Xu, Xiangdong Liu, Chunxiao Liu, Qianqian Gao

**Affiliations:** a Gastrointestinal Surgery Medical Center, Weifang People’s Hospital (The First Affiliated Hospital of Shandong Second Medical University), Weifang, Shandong, China; b Departments of Obstetrics Medical Center, Weifang People’s Hospital (The First Affiliated Hospital of Shandong Second Medical University), Weifang, Shandong, China.

**Keywords:** hemorrhoidal disease, mendelian randomization, physical activity, sedentary, tobacco and alcohol use

## Abstract

Prior investigations have indicated an association between sedentary and physically active behaviors, tobacco and alcohol intake behaviors, and hemorrhoidal disease (HD). Yet, the causal relationship between these factors and HD remains unclear and is a topic of debate. The data from the genome-wide association study were selected as the exposures (sedentary behavior, physical activity behavior, and tobacco and alcohol intake behavior) and the outcome (HD). We employed a range of Mendelian randomization (MR) analysis methods for causal estimation. The primary analysis method was the inverse variance weighted random effect model (IVW[RE]), with the MR-Egger, weighted median estimator, MR-pleiotropy residual sum and outlier (MR-PRESSO), MR-Radial, and MR-LAP methods serving as auxiliary and supplementary. Additionally, secondary MR analyses were conducted by identifying and removing outlier single nucleotide polymorphisms through MR-Radial. A series of sensitivity analyses were performed to ascertain the reliability and robustness of the results. Among the MR analysis results, the IVW(RE) results for Leisure screen time’s analysis with HD showed an odds ratio (OR) of 1.052 (95% confidence interval [CI]: 1.001–1.106, *P*: .047). After the removal of outliers, the IVW(RE) OR was 1.055 (95% CI: 1.015–1.096, *P*: .006). For the smoking initiation analysis with HD, the *P* difference in the MR-LAP results was 0.031. The MR-LAP corrected IVW results were deemed to be more plausible, with an OR of 1.058 (95% CI: 1.020–1.096, *P*: .002). After the removal of outliers, the IVW(RE) yielded an OR of 1.066 (95% CI: 1.007–1.129, *P*: .029). In the initial analysis of moderate-to-vigorous intensity physical activity during leisure time with HD, weighted median estimator yielded a negative correlation (OR: 0.856, 95% CI: 0.750–0.978, *P*: .022). Following the removal of outliers, the IVW(RE) results indicated a negative correlation (OR: 0.900, 95% CI: 0.814–0.995, *P*: .039). For smoking cessation analysis with HD, *P* difference was found to be statistically significant (.034) in the MR-LAP results. The corrected IVW results yielded an OR of 0.951 (95% CI: 0.907–0.997, *P*: .035). The secondary analysis yielded an OR of 0.901 (95% CI: 0.812–1.000, *P*: .05). However, no notable correlation was identified between alcohol consumption and HD in the conducted analyses. The results indicate that sedentary and smoking behaviors are risk factors, whereas physical active and smoking cessation are possible protective factors. The study provides evidence for further research into the etiology of HD and enables the development of prevention strategies.

## 1. Introduction

Hemorrhoidal disease (HD) is one of the most prevalent diseases, with colonoscopy screening indicating a prevalence of hemorrhoids approaching 40%.^[1,2]^ Both HD and its treatment result in significant pain and a reduction in quality of life in patients.^[3]^ Furthermore, HD may also increase the risk of colorectal adenomas and even colorectal cancer.^[4,5]^Diagnosis and treatment of HD present a certain degree of difficulty,^[6]^ consuming a substantial amount of medical resources.^[7]^ In addition, the available therapeutic modalities have varying rates of recurrence and complication.^[8,9]^ The treatment of hemorrhoids represents a significant physical and economic burden, underscoring the importance of active and effective preventive strategies.

Nevertheless, there are numerous areas of HD that remain poorly understood.^[10]^ The current epidemiological evidence for HD is insufficient.^[8]^ Behaviours such as sedentary, physical activity, smoking and alcohol consumption have been demonstrated to exert a significant impact on health outcomes.^[11–13]^ Previous epidemiological studies have reported associations between the aforementioned behaviors and HD; however, the findings have been inconsistent.^[2,14–18]^ Moreover, the majority of the evidence is derived from cross-sectional and retrospective studies,^[8]^ which are prone to confounding factors and reverse causality, among other issues. It is therefore necessary to employ more rigorous and scientific methods in order to further investigate these associations.

Mendelian randomization (MR) studies based on genome-wide association study (GWAS) data employ single-nucleotide polymorphisms (SNPs) as instrumental variables (IVs) in analyses to identify causal associations between risk and disease.^[19]^ MR analyses perform random assignment in accordance with MR principles, which can effectively circumvent confounding effects, thereby enhancing causal inference.^[20]^ Consequently, we employed MR studies to investigate the correlation between the daily behavioral patterns and HD at the genetic level. The results provide compelling evidence to support further etiological studies of hemorrhoids and inform the development of effective prevention strategies.

## 2. Methods

### 2.1. Study design and data source

**Figure 1.**
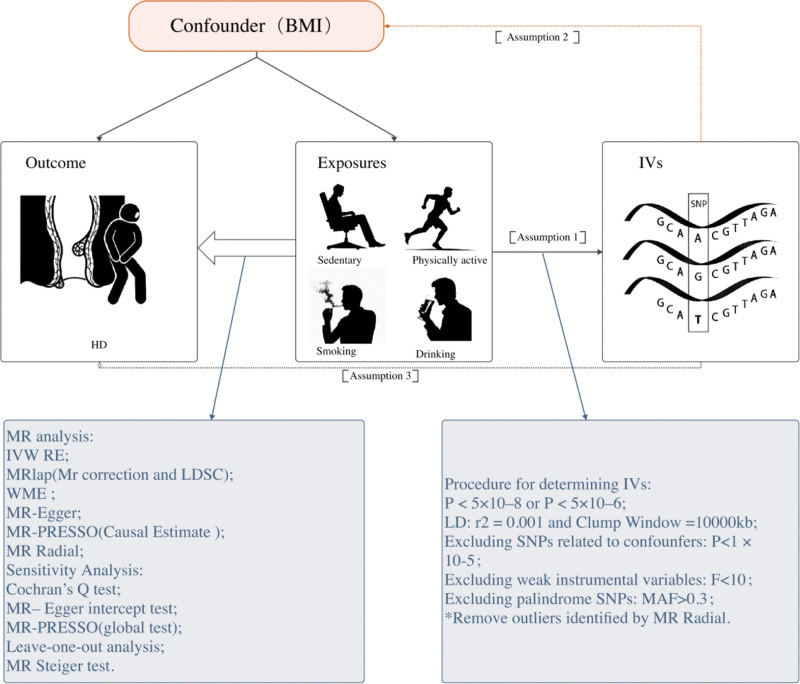
Study design. Assumption 1: Correlation-instrumental variables are strongly correlated with exposure; Assumption 2: Independence-instrumental variables are not associated with confounders affecting both exposure and outcome; Assumption 3: Exclusivity-genetic variant affect outcomes only through exposure. * Remove outliers identified by MR-Radial were used for analyses after removal of outliers. BMI = body mass index, IVs = instrumental variables, IVW(RE) = inverse-variance weighted random effect model, LD = linkage disequilibrium, LDSC = linkage disequilibrium score regression, MR = Mendelian randomization, WME = weighted median estimator.

This study employs a 2-sample MR approach to investigate the causal association between sedentary behavior, physical activity, smoking, and drinking and HD (Fig. 1).

The GWAS data on sedentary and physically active behaviors were derived from a meta-analysis, which combined data from up to 703,901 individuals (94.0% European, 2.1% African, 0.8% East Asian, 1.3% South Asian, and 1.9% Hispanic) from 51 studies.^[21]^ The genetic models were harmonized in accordance with the specified criteria, including age, population structure and other characteristics pertinent to the study. Leisure screen time (LST) was defined as time spent watching television, playing video games, sitting in front of a computer, etc. It was treated as a continuous variable. Moderate-to-vigorous intensity physical activity during leisure time (MVPA) was defined as a categorical variable based on whether or not one participates in physical activity and the duration of the activity. Sedentary behavior at work (SDW) was defined as a categorical variable based on sedentary status and time at work. Sedentary commuting behavior (SDC) was defined as a categorical variable based on commuting mode and time.

The GWAS data for tobacco and alcohol intake behaviors were obtained from the latest release data of the Sequencing Consortium for Alcohol and Nicotine Use, which consists of a meta-analysis of GWAS summary statistics from 60 study cohorts comprising 3,383,199 individuals with ancestral diversity.^[22]^ Participants of European ancestry were included in this study. The variables included in the analysis were as follows: age of initiation (AgeSmk), denoting age of initiation of smoking; cigarettes per day, number of cigarettes smoked by smokers per day; smoking cessation (SmkCes); smoking initiation (SmkInit), whether or not one smokes on a regular basis; and drinks per week (DrnkWk), weekly alcohol consumption.

The data on HD were obtained from a meta-analysis GWAS, comprising a total of 944,133 participants of European ancestry from 5 extensive population-based cohorts.^[23]^ The data were treated as a binary variable, encompassing 725,213 controls and 218,920 cases of HD.

The GWAS data employed in this article are derived from publicly accessible GWAS studies. For further details on the summary data, please consult the original literature. The summary data for HD are available at https://www.ebi.ac.uk/gwas/ Study: GCST90014033. The summary data for LST, MVPA, SDC, SDW are available at https://www.ebi.ac.uk/gwas/ Study: GCST90104339; GCST90104341; GCST90104343; GCST90104345. The summary data for tobacco and alcohol use are retrieved from the Data Repository for the University of Minnesota, https://doi.org/10.13020/przg-dp88.The studies obtained informed consent from all participating researchers in accordance with protocols approved by the respective institutional review boards. Accordingly, an additional ethical statement was not deemed necessary for this study.

### 2.2. Procedure for determining IVs

In the 2-sample MR analysis, SNPs in the exposure data that satisfied the core assumptions were selected as IVs. Firstly, IVs needed to satisfy the strong correlation with exposure, and the primary threshold (*P* < 5 × 10^−8^) was used as the threshold for significance. Following the screening procedure, exposure data with an insufficient number of SNPs (< 3) could not be obtained. The second threshold (*P* < 5 × 10^−6^) was employed as the threshold. Secondly, the SNPs were clumped by removing linkage disequilibrium (LD) (clumping *R*^2^ cutoff = 0.001 and clump window = 10000 kb). Thirdly, the phenotypic traits associated with the identified IVs were queried through the LD trait database,^[24]^ with the exclusion of SNPs that were found to be associated with confounders (body mass index^[11,12,14,25]^) (https://ldlink.nih.gov/?tab=ldtrait, accessed on June 25,2024).The *F*-statistic and *R*^2^ of the SNPs screened were calculated to assess the presence of weak IVs(*R*^2^ = [2 ×*β*^2^]/[(2 × *β*^2^) + (2 × N × SE^2^)], *F* = *R*^2^ × [N-2]/[1-*R*^2^], *β*: Ratio of the SNP-exposure association estimate, SE: standard error of *β*, N: Sample size). An *F*-statistic > 10 indicated the absence of weak IVs.^[26]^ Subsequently, the palindromic SNPs were removed based on a minor allele frequency > 0.3.^[27]^ When specific SNPs were not available in the data, we sought proxy SNPs through LD (*r*^2^ = 0.8; minor allele frequency = 0.3).

Similarly, MR-LAP analyses employed the identical significance thresholds for IV screening as the other 2-sample MR analyses. Moreover, the MR-LAP statistical approach utilizes LD parameters (*R*^2^ = 0, clump window = 5000 kb), which is a notable distinction from other methods.^[28]^

### 2.3. Statistical analysis

### 2.4. 1 Two-sample MR analysis

We employed the aforementioned procedure to screen the IVs for an initial 2-sample MR analysis. The main analytical method employed was the IVW random effect model (IVW[RE]).^[29]^As both exposures and outcomes are summary data, the MR-LAP method was used for validation, while correcting for bias due to sample overlap. The MR-LAP method utilizes summary data, making the analysis reliable by assuming a spike-and-slab genomic architecture, and employs LD score regressions and other techniques to correct for IVW bias. The MR-LAP results were also provided in conjunction with the LD score regression results, which pertained to the assessment of the genetic correlation.^[28]^

In addition, the study employed the MR-Egger^[30]^ and the weighted median estimator (WME),^[31]^the MR-pleiotropy residual sum and outlier (MR-PRESSO),^[32]^and the MR-Radial^[33]^to assess the causal effect of exposures and outcomes. MR-PRESSO identifies and corrects for outliers, and performs causal estimation to provide the original estimate of the results and results after correcting for identified outliers. The IVW radial function of MR-Radial employs first-order, second-order, or modified second-order weights to fit a radial inverse variance weighted model. Furthermore, Cochran *Q*-statistic was employed to identify outliers and calculate them, including iterative and exact inverse variance weighted estimates. This approach enhanced the precision of MR of 2-sample summary data beyond the NO measurement error assumption.^[34]^

#### 2.4.1. Sensitivity MR analyses

In sensitivity analyses, we conducted heterogeneity and pleiotropy tests, including the Cochran *Q*-test, the Egger-intercept test, and the MR-PRESSO global test. Furthermore, we performed a robustness analysis using the leave-one-out method. Finally, we employed the Steiger test to assess the directionality of causal associations.^[35]^

The higher heterogeneity observed in the sensitivity analyses (Cochran-*Q* test, MR-Egger *P* < .05 and MR-PRESSO global test, *P* < .05) In such circumstances, the results of causal extrapolations are susceptible to bias.^[36]^To address this, we employed MR-Radial Cochran *Q*-statistics to identify outliers and remove outlier SNPs from the IVs. Subsequently, a secondary MR analysis was conducted with outliers removed.

### 2.5. Estimation of causality

The causal estimates between the exposures and the outcome were based on the IVW(RE) results initially and after removal of the outlier SNPs. Additionally, a combination of MR-Egger and WME, MR-PRESSO, and MR-Radial results were considered. When both the initial and the IVW(RE) results, after removal of outlier SNPs, support the same causal estimate and are supported by the results of other analyses, we consider the causal inference of gene prediction to be valid. When one of the IVW(RE) results, both initially and after removal of the outlier SNP, supported the causal association and was partially supported by the results of other analyses, we considered it probable that a gene-predicted causal association existed. In the event that neither the initial nor the IVW(RE) results, following the removal of outlier SNPs, supported a causal association, no causal association was deemed to exist. In order to ascertain causal estimates, bias resulting from the overlap of summary data was evaluated using the MR-LAP method. When a significant difference was identified (*P* difference < .05), the result of correction by the MR-LAP method was prioritized.

All statistical analyses were conducted using the R software, version 4.3.2 (R Foundation for Statistical Computing, Vienna, Austria), with the relevant package. The package utilized for these analyses included TwoSampleMR version 0.6.4, MR- Radial version 1.1, and MRlap version 0.0.3.2.

## 3. Results

### 3.1. Screening results of IVs

In the 2-sample MR analysis, a significance threshold of *P* < 5 × 10^−6^ was employed for SDC, due to constraints imposed by sample size, whereas *P* < 5 × 10^−8^ was applied to all other exposures. Phenotypic traits associated with IVs were queried through the LD trait database, and IVs associated with the confounding variable body mass index were removed (Table S6, accessed on June 25,2024). The *F*-statistic for each IV included in the analysis exceeded 10, indicating a strong association between the IV and the exposure factor. (Table S4 and S5).

### 3.2. Sedentary and physical activity behaviors to HD

**Figure 2.**
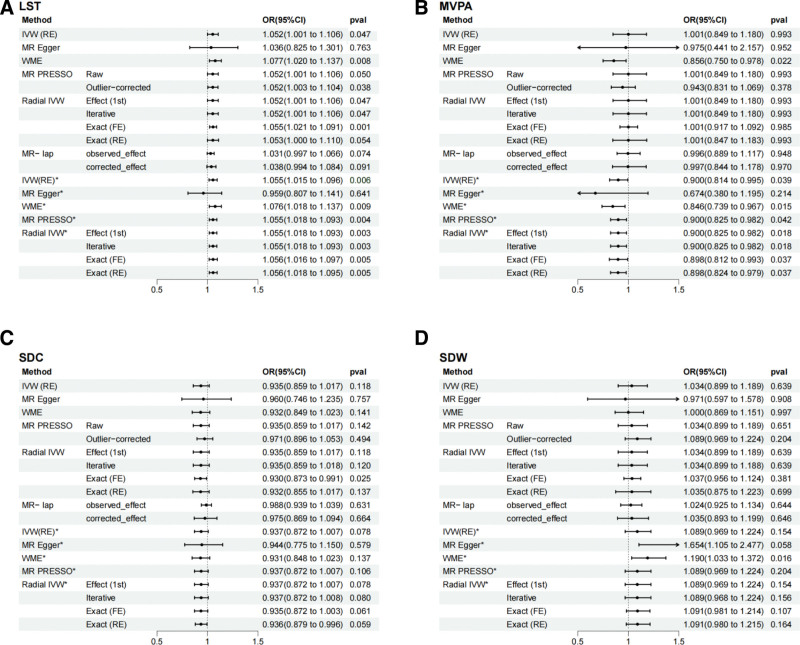
The causal effect estimates of sedentary and physically activity on HD. (A) Causal effect estimates of LST and HD; (B) Causal effect estimates of MVPA and HD; (C) Causal effect estimates of SDC and HD; (D) Causal effect estimates of SDW and HD. * Analysis after removal of outliers. OR indicates odds ratio and plots represent OR (95% CI). CI = confidence interval, HD = hemorrhoidal disease, LST = leisure screen time, MVPA = moderate-to-vigorous intensity physical activity during leisure time, OR = odds ratio, SDC = sedentary commuting behavior, SDW = sedentary behavior at work.

The results of the MR analysis indicated a positive genetically predicted causal association between LST and HD, while there was a possible negative correlation between MVPA and HD. No causal associations were identified between SDC and SDW and HD.(Fig. 2)

In the initial analysis of LST versus HD, the IVW(RE) yielded significant positive results (odds ratio [OR] = 1.052, 95% confidence interval [CI] = 1.001–1.106 *P* = .047). Furthermore, the results of the WME, MR-PRESSO (Outlier-corrected), and Radial IVW methods exhibited a directionally consistent outcome with the IVW method and a *P* value < .05. The association remained significant after the removal of outliers (IVW OR = 1.055, 95% CI = 1.015–1.096, *P* = .006). Furthermore, the direction of the analytical results of the WME, MR-PRESSO, and Radial IVW methods was consistent with that of the IVW, and the *P* value < .05. The results of the MRlap method indicated a *P* difference = .151, which did not reach the threshold for statistical significance. This suggests that the IVW(RE) analysis results are credible. (Fig. 2A, Tables S1, and S2)

The initial analysis of MVPA and HD using the IVW(RE) method did not yield a definitive association (OR = 1.001, 95% CI = 0.849–1.180, *P* = .993). However, the WME method did indicate a negative association (OR = 0.856, 95% CI = 0.750–0.978, *P* = .022). Following the removal of outliers, the IVW(RE) yielded a negative correlation (OR = 0.900, 95% CI = 0.814–0.995, *P* = .039). Additionally, the results of the WME, MR-PRESSO, and Radial IVW methods were in alignment with those of the IVW(RE) methods, with *P* < .05. The *P* value for the MR-LAP method results was 0.980, which did not reach the significance threshold. (Fig. 2B, Tables S1 and S2)

In the MR analysis of SDC, SDW and HD, no significant correlation was obtained for the IVW(RE) results. Furthermore, the significance threshold was reached in the other results only in the single corrected results. The *P*difference in the MR-LAP results was > .05, which did not reach the significance threshold. (Fig. 2C and D, Tables S1, and S2)

### 3.3. Tobacco and alcohol intake behaviors to HD

**Figure 3.**
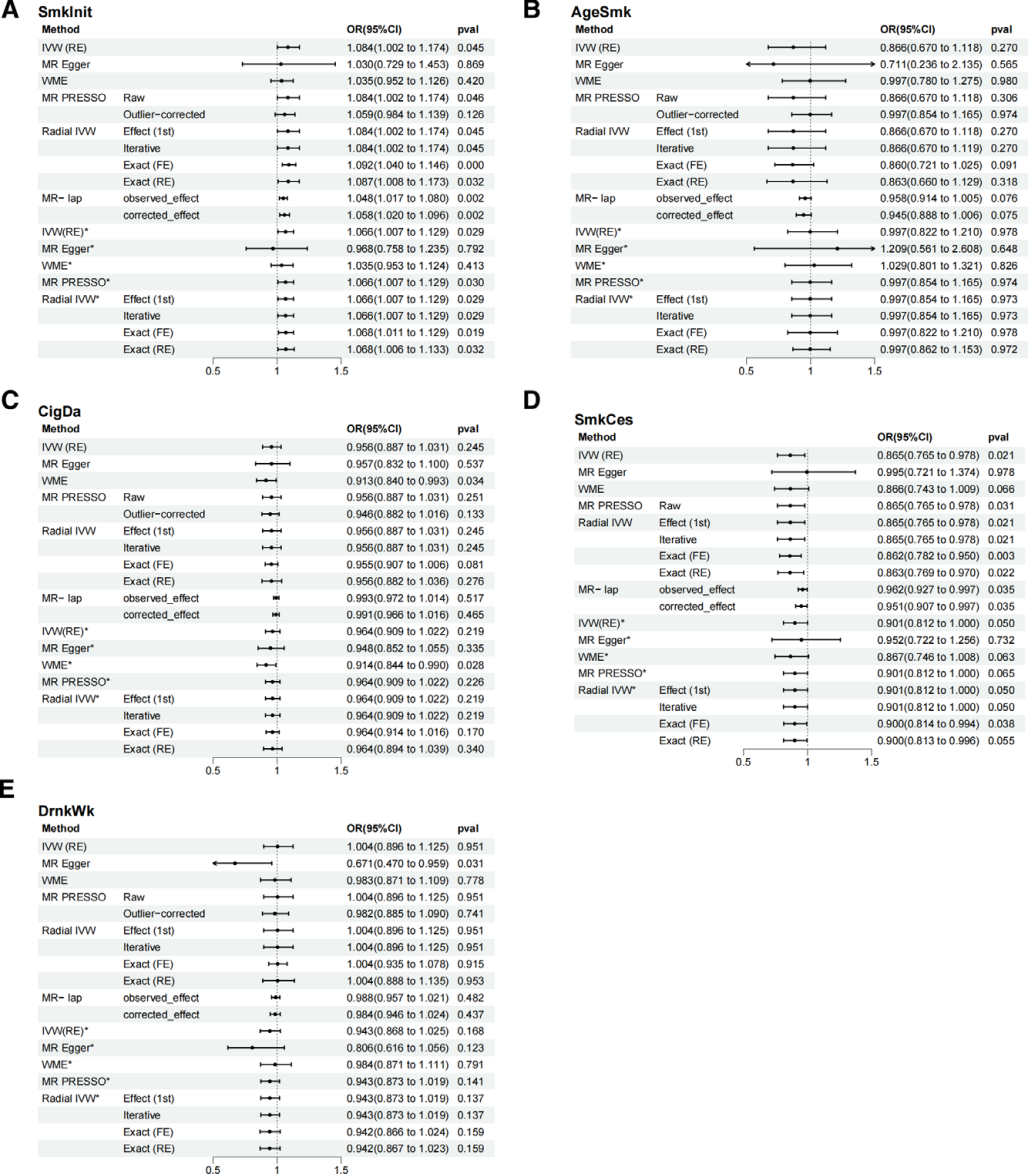
The causal effect estimates of Sedentary and Physically activity on HD. (A) Causal effect estimates of SmkInit and HD; (B) Causal effect estimates of AgeSmk and HD; (C) Causal effect estimates of CigDay and HD; (D) Causal effect estimates of SmkCes and HD; (E) Causal effect estimates of DrnkWk and HD. * Analysis after removal of outliers. ** OR indicates odds ratio and plots represent OR (95% CI). AgeSmk = age of initiation, CI = confidence interval, CigDay = cigarettes per day, DrnkWk = Drinks per week, HD = hemorrhoidal disease, OR = odds ratio, SmkCes = Smoking cessation, SmkInit = smoking initiation.

The findings indicate a positive causal association between genetically predicted SmkInit for HD. Conversely, there is a possible negative correlation betwe-en SmkCes and HD. However, there is no evidence to suggest that there is a genetically predicted correlation between DrnkWk and HD. (Fig. 3)

For SmkInit analysis with HD, *P* difference = .031 and test difference = -2.162 in the MR-LAP method results, indicated a discrepancy between the IVW method analysis results and the corrected effects, which point to an underestimation of the effect estimate obtained using IVW. The corrected IVW(RE) sults in the MR-LAP method were more credible (OR = 1.058, 95% CI = 1.020–1.096, *P* = .002). And initial analyses of the IVW(RE) method results showed (OR = 1.084, 95% CI = 1.002–1.174, *P* = .045). The results of the WME, MR-Egger, MR-PRESSO, and Radial IVW methods were in agreement with the IVW method in terms of directionality. Furthermore, the *P* value for MR-PRESSO (Raw), Radial IVW method was < .05. The IVW(RE) also indicated a positive association in the results of the analyses conducted after excluding the outliers (OR = 1.066, 95% CI = 1.007–1.129, *P* = .029). The results of the analyses of WME, MR-PRESSO, and Radial IVW method were found to be in alignment with the IVW directionality. Additionally, the *P* value for MR-PRESSO and Radial IVW method was found to be < .05 (Fig, 3A, Table S1 and S2).The above analytical findings collectively support the existence of a positive causal relationship between SmkInit and HD.

Similarly, for the analysis of SmkCes with HD, *P* difference = .034 and test_difference = 2.123 in the MR-LAP method results indicated a bias in the results of the IVW(RE) analyses. The corrected IVW results in the MR-LAP method results were more plausible (OR = 0.951, 95% CI = 0.907–0.997 *P* = .035). The original IVW(RE) method results showed an OR = 0.865, 95% CI = 0.765–0.978, *P* = .021; the direction of the analyzed results of WME, MR-Egger, MR-PRESSO (Raw) and Radial IVW methods were consistent with the IVW(RE) method direction. And the *P* value of WME, MR-PRESSO (Raw), Radial IVW methods was < 0.05. The IVW(RE) results in the analyses performed after excluding the outliers showed an OR = 0.901, 95% CI = 0.812–1.000, *P* = .05; the direction of the results of the analyses of WME, MR-Egger, MR-PRESSO and Radial IVW methods were consistent with the IVW(RE) directionality. (Fig. 3D, Tables S1 and S2).

For the MR analyses of AgeSmk, cigarettes per day, DrnkWk and HD, no significant associations were found for the IVW(RE) results. The *P* difference in the MR-LAP method results was > .05 and did not reach the significance threshold. (Fig. 3B, 3C and 3E, Table S1, S2, and S3)

### 3.4. Sensitivity analyses

In the sensitivity analyses, the *P* value of the Cochran *Q*-test and the MR-PRESSO Global test in the initial analysis was < 0.05, and the *P* value of the Egger intercept test was > .05. After correction for outliers, there was no significant heterogeneity or polytropy. The causal direction of the Steiger test was TRUE (Table S3). The leave-one-out sensitivity analysis of the MR data of the 2 samples did not reveal any SNP loci in the IVs that had a strong influence on the results (Supplementary file 1). The MR-Radial Cochran *Q*-statistic identified outliers (Supplementary file 2). The LD score regression results of the MR-LAP method showed that the exposure heritability estimate was > 0.02, and the genetic correlation between exposure data and outcome was < 0.2 (Table S2).

## 4. Discussion

Previous epidemiological studies have considered sedentary and physical activity, tobacco and alcohol consumption as risk factors for HD, but these have been controversial in different studies. In addition, unmeasured confounders in clinical studies may bias the association. In our study, we applied the large-sample GWAS data and performed Two-sample MR analysis based on multiple MR analysis methods for causal assessment, while genetic correlation was assessed. Our results showed positive genetic correlations between sedentary and smoking behavior and HD, whereas there may be negative genetic correlations for physical activity and smoking cessation behavior. No clear genetic association was found between alcohol consumption and HD. Certain behavioral traits (SDC, SDW, AgeSmk, SmkCes) did not show a clear and convincing genetic association with HD in the MR analysis.

Sedentary behavior and physical activity have long been known to correlate with the development of HD, but previous studies have shown inconsistent results. A study by Peeters GM on the longitudinal relationship between sedentary behavior and a range of physical and psychological symptoms in Australian women reported that no significant association was found between sedentary behavior and HD (OR = 1.18; 95% CI = 0.92–1.50; *P* = .08).^[17]^ While the results of a cross-sectional study by Peery AF, et al suggested that sedentary behavior was associated with a reduced risk of HD, no association was found between physical activity and HD.^[2]^ Hong YS, et al, a cross-sectional study of 194,620 healthy Korean men and women, suggested that moderate exercise was a protective factor for the development of HD in the study.^[14]^ Another Korean retrospective study reported that the prevalence of HD was higher in the 1 to 4 times per week exercise group than in the non-exercise group in both diagnostic and surgical situations.^[15]^ Our results support that sedentary behavior is a risk factor for HD, whereas physical activity appears to be a protective factor. While SDC and SDW did not find a clear association with HD. The current study suggests that sedentary-induced pathophysiological changes focus on the vascular system, autonomic nervous system and metabolic factors.^[11]^ We suggest that sedentary behavior may trigger HD by affecting the vascular and connective tissue system, causing degeneration of the supporting tissues in the anal canal as well as varicose or even thrombotic formation of the vascular system. Physical activity, on the other hand, may reduce the risk of HD through the molecular stimulation and metabolic changes induced by muscular activity and the replacement of sedentary time.^[37]^

Smoking is considered to be a risk factor for a number of diseases and has a significant effect on the digestive and vascular systems. A retrospective case-control study by Nagaraj SV, et al^[16]^ reported that smokers had a 2.4 times higher risk of HD compared to nonsmokers. The study also reported no statistically significant difference in the prevalence of HD between former and current smokers, suggesting that smoking cessation does not reduce the risk of developing HD. A study by Hong YS, et al reported that ever or current smoking was independently associated with the prevalence of HD.^[14]^ The study by Hong Y^[15]^ also reported that smoking is a risk factor for HD. Smoking may directly increase the risk of HD by promoting inflammatory response and muscle atrophy^[38]^ and effects on collagen metabolism,^[39]^ and may also play a role by causing vascular endothelial damage with effects on the vasculature.^[40]^ In our MR study, the results showed that smoking was positively associated with the risk of HD, which is consistent with previous studies. On the other hand, smoking cessation may be negatively associated with the risk of HD, which is different from previous studies and may be due to the fact that smoking cessation removes continuous exposure to some components of tobacco, but this needs to be clarified in further studies. There was no clear causal relationship with the age at which smoking started or the number of cigarettes smoked per day.

In our study, weekly alcohol consumption was used for MR analysis and the results of the study did not show a causal relationship with HD. The association between alcohol consumption and HD in previous studies is debatable.^[41]^ A retrospective study by Hong J, et al^[15]^ showed that alcohol consumption was a risk factor for the development of HD. Some other studies found no association.^[14,25]^ We suggest that alcohol consumption may not be a risk factor for HD, but alcohol consumption may cause HD to worsen, leading to an increase in hemorrhoid visits and the discovery of occult HD.

In this study, the data used are from the latest large GWAS studies, and the large sample size ensures sufficient statistical power. At the same time, it may bring problems such as heterogeneity and sample overlap. A variety of MR analysis methods and a rigorous screening process for IVs were used to reduce the bias caused by various confounding factors. In our study, the reliability of IVW analysis results was assessed and corrected using the MR-LAP method, and the genetic correlation between exposure and outcome was also assessed. We used a series of sensitivity analyses to test the hypotheses of MR. To ensure consistency of genetic background, our MR analyses included only participants of European ancestry, which limits the applicability of the results to other ethnic groups. A number of different MR analyses were used in the analyses, and 2 MR analyses were performed initially, with outliers removed. Some of the methods gave inconsistent results in cases where precise conclusions could not be drawn.

## 5. Conclusions

The results of our study supported sedentary and smoking behaviors as risk factors for HD, whereas physical activity and smoking cessation behaviors appeared to be protective factors for the development of HD, and there was no evidence of a causal relationship between alcohol consumption and HD. Our study confirms the genetic association between sedentary, physical activity and smoking behaviors and HD, and provides strong evidence for investigating the etiology of HD. Although some of the results need to be validated in further studies, they can provide a reference for the development of prevention strategies for HD.

## Acknowledgments

We thank the original GWAS and associated consortia for generously sharing and managing the summary statistics.

## Author contributions

**Resources:** Jinqiu Xiong.

**Software:** Jinqiu Xiong, Yuanyuan Xu.

**Supervision:** Qianqian Gao.

**Visualization:** Xiangdong Liu.

**Writing – original draft:** Jinqiu Xiong.

**Writing – review & editing:** Yuanyuan Xu, Xiangdong Liu, Chunxiao Liu, Qianqian Gao.
















