# Supplementary material for: Causal associations between lifestyle factors and hemorrhoidal disease: Insights from Mendelian randomization analysis
Source: Medicine (Baltimore). 2026 May 22;105(21):e48945. doi: 10.1097/MD.0000000000048945 (PMC13200937; doi:10.1097/MD.0000000000048945)
Supplement: Supplementary file 1 [file medi-105-e48945-s001.docx]

| Supplementary Table 6 . Phenotypic traits associated with IVs were queried through the "LDtrait" database. | | | | | | | | | |
| --- | --- | --- | --- | --- | --- | --- | --- | --- | --- |
| Expose | Query | GWAS_Trait | PMID | Position_GRCh37 | Alleles | Risk_Allele | Effect_Size_95_CI | Beta_or_OR | P_value |
| LST | rs197439 | Leisure screen time | 36071172 | chr1:112280990 | A=0.572, G=0.428 | 0.601 | 0.026 | 0.018-0.034 | 3.00E-11 |
| LST | rs61813324 | Body mass index | 30595370 | chr1:156049877 | C=0.87, T=0.13 | NR | NA | NA | 8.00E-24 |
| LST | rs61813324 | Body mass index | 30239722 | chr1:156049877 | C=0.87, T=0.13 | 0.1312 | 0.0289 | 0.023-0.034 | 3.00E-24 |
| LST | rs61813324 | Body mass index | 30239722 | chr1:156049877 | C=0.87, T=0.13 | 0.1312 | 0.0289 | 0.023-0.034 | 3.00E-24 |
| LST | rs61813324 | Body mass index | 30239722 | chr1:156049877 | C=0.87, T=0.13 | 0.1312 | 0.0289 | 0.023-0.034 | 3.00E-24 |
| LST | rs61813324 | Body mass index | 31669095 | chr1:156049877 | C=0.87, T=0.13 | NR | NA | NA | 6.00E-24 |
| LST | rs61813324 | Body mass index | 34594039 | chr1:156049877 | C=0.87, T=0.13 | NR | 0.028 | 0.021-0.035 | 5.00E-16 |
| LST | rs61813324 | Weight | 34594039 | chr1:156049877 | C=0.87, T=0.13 | NR | 0.0224 | 0.016-0.028 | 2.00E-13 |
| LST | rs61813324 | Adult body size | 32376654 | chr1:156049877 | C=0.87, T=0.13 | 0.864324 | 0.0182963 | 0.014-0.022 | 2.00E-19 |
| LST | rs61813324 | Body mass index | 38538606 | chr1:156049877 | C=0.87, T=0.13 | NR | 0.0287467 | 0.022-0.036 | 7.00E-16 |
| LST | rs61813324 | Body mass index (MTAG) | 36376304 | chr1:156049877 | C=0.87, T=0.13 | NR | 0.030992 | 0.025-0.037 | 4.00E-24 |
| LST | rs61813324 | Body mass index | 36581621 | chr1:156049877 | C=0.87, T=0.13 | NR | 0.0265 | 0.022-0.031 | 3.00E-27 |
| LST | rs61813324 | Body mass index | 37280435 | chr1:156049877 | C=0.87, T=0.13 | NR | 0.0284463 | 0.023-0.034 | 3.00E-24 |
| LST | rs61813324 | Whole body fat mass (UKB data field 23100) | 38538606 | chr1:156049877 | C=0.87, T=0.13 | NR | 0.0244053 | 0.018-0.031 | 3.00E-12 |
| LST | rs6685030 | Leisure screen time | 36071172 | chr1:171805284 | A=0.427, G=0.573 | 0.48 | 0.022 | 0.014-0.03 | 5.00E-10 |
| LST | rs3791033 | ADHD vs autism spectrum disorder (ordinary least squares (OLS)) | 33686288 | chr1:44134077 | C=0.312, T=0.688 | NR | 0.0151 | NA | 4.00E-09 |
| LST | rs3791033 | Problematic opioid prescription use | 34728798 | chr1:44134077 | C=0.312, T=0.688 | NR | 5.491 | NA | 4.00E-08 |
| LST | rs3791033 | Educational attainment (years of education) | 30038396 | chr1:44134077 | C=0.312, T=0.688 | 0.6703 | 0.0136 | 0.01-0.017 | 2.00E-17 |
| LST | rs3791033 | Leisure screen time | 36071172 | chr1:44134077 | C=0.312, T=0.688 | 0.331 | 0.033 | 0.025-0.041 | 4.00E-16 |
| LST | rs3791033 | Random glucose levels | 37679419 | chr1:44134077 | C=0.312, T=0.688 | 0.67 | 0.0017 | 0.0011-0.0023 | 4.00E-08 |
| LST | rs3791033 | Random glucose levels | 37679419 | chr1:44134077 | C=0.312, T=0.688 | 0.6722 | 0.00171956 | 0.0011-0.0023 | 3.00E-08 |
| LST | rs10889193 | Leisure screen time | 36071172 | chr1:61106174 | A=0.596, C=0.404 | 0.445 | 0.024 | 0.016-0.032 | 5.00E-10 |
| LST | rs71658797 | High light scatter reticulocyte percentage of red cells | 32888494 | chr1:77967507 | A=0.086, T=0.914 | 0.12166 | 0.024480302 | 0.018-0.031 | 1.00E-12 |
| LST | rs71658797 | Immature fraction of reticulocytes | 32888494 | chr1:77967507 | A=0.086, T=0.914 | 0.121718 | 0.020882582 | 0.014-0.028 | 1.00E-09 |
| LST | rs71658797 | C-reactive protein levels | 31900758 | chr1:77967507 | A=0.086, T=0.914 | 0.876576 | 0.0195452 | 0.014-0.026 | 1.00E-10 |
| LST | rs71658797 | Lung adenocarcinoma | 28604730 | chr1:77967507 | A=0.086, T=0.914 | 0.102617403 | 1.1845306 | 1.123671054-1.248686293 | 3.00E-10 |
| LST | rs71658797 | Lung cancer | 28604730 | chr1:77967507 | A=0.086, T=0.914 | 0.103377896 | 1.1362852 | 1.094197108-1.179992156 | 3.00E-11 |
| LST | rs71658797 | Lung cancer in ever smokers | 28604730 | chr1:77967507 | A=0.086, T=0.914 | 0.102898044 | 1.145921 | 1.092331759-1.202139175 | 2.00E-08 |
| LST | rs71658797 | Non-small cell lung cancer | 32889700 | chr1:77967507 | A=0.086, T=0.914 | NR | 1.16 | 1.09-1.22 | 9.00E-07 |
| LST | rs71658797 | Metabolic biomarkers (multivariate analysis) | 33980691 | chr1:77967507 | A=0.086, T=0.914 | 0.12 | NA | NA | 3.00E-10 |
| LST | rs71658797 | Body fat percentage | 30593698 | chr1:77967507 | A=0.086, T=0.914 | NR | 0.1857 | 0.12-0.25 | 1.00E-08 |
| LST | rs71658797 | Predicted visceral adipose tissue | 31501611 | chr1:77967507 | A=0.086, T=0.914 | 0.1226 | 0.034701146 | 0.027-0.042 | 5.00E-20 |
| LST | rs71658797 | Body fat percentage | 33980691 | chr1:77967507 | A=0.086, T=0.914 | 0.12 | 0.024 | % | 6.00E-26 |
| LST | rs71658797 | Leisure screen time | 36071172 | chr1:77967507 | A=0.086, T=0.914 | 0.878 | 0.037 | 0.025-0.049 | 2.00E-10 |
| LST | rs71658797 | Diastolic blood pressure | 35762941 | chr1:77967507 | A=0.086, T=0.914 | NR | 0.2082 | 0.14-0.27 | 1.00E-10 |
| LST | rs71658797 | Educational attainment | 35361970 | chr1:77967507 | A=0.086, T=0.914 | 0.1116 | 0.0174527 | 0.014-0.021 | 9.00E-26 |
| LST | rs71658797 | COVID-19 (critical illness vs population) | 37198478 | chr1:77967507 | A=0.086, T=0.914 | 0.12 | 1.1 | 1.09-1.18 | 3.00E-09 |
| LST | rs71658797 | Whole body fat free mass (UKB data field 23101) | 38538606 | chr1:77967507 | A=0.086, T=0.914 | NR | 0.0289194 | 0.025-0.033 | 5.00E-46 |
| LST | rs71658797 | Body mass index | 38538606 | chr1:77967507 | A=0.086, T=0.914 | NR | 0.0389024 | 0.032-0.046 | 8.00E-26 |
| LST | rs71658797 | Childhood body mass index | 37280435 | chr1:77967507 | A=0.086, T=0.914 | NR | 0.014759 | 0.0085-0.021 | 4.00E-06 |
| LST | rs71658797 | Body mass index | 37280435 | chr1:77967507 | A=0.086, T=0.914 | NR | 0.037552 | 0.032-0.043 | 1.00E-36 |
| LST | rs71658797 | Whole body fat mass (UKB data field 23100) | 38538606 | chr1:77967507 | A=0.086, T=0.914 | NR | 0.042166 | 0.035-0.049 | 5.00E-31 |
| LST | rs12062845 | Leisure screen time | 36071172 | chr1:98342685 | A=0.212, C=0.788 | 0.784 | 0.028 | 0.02-0.036 | 2.00E-11 |
| LST | rs841020 | Leisure screen time | 36071172 | chr10:125409953 | C=0.806, T=0.194 | 0.808 | 0.027 | 0.019-0.035 | 1.00E-09 |
| LST | rs1017550 | Leisure screen time (MTAG) | 36071172 | chr10:63587683 | A=0.624, G=0.376 | NR | 0.02 | 0.014-0.026 | 4.00E-09 |
| LST | rs68049022 | Leisure screen time | 36071172 | chr10:66407019 | C=0.183, T=0.817 | 0.202 | 0.031 | 0.021-0.041 | 6.00E-11 |
| LST | rs4483592 | Leisure screen time | 36071172 | chr11:65990439 | C=0.827, T=0.173 | 0.837 | 0.036 | 0.026-0.046 | 4.00E-12 |
| LST | rs1391954 | Leisure screen time | 36071172 | chr11:88575965 | G=0.567, T=0.433 | 0.556 | 0.025 | 0.017-0.033 | 2.00E-09 |
| LST | rs10765775 | Educational attainment (years of education) | 30595370 | chr11:95656362 | A=0.39, G=0.61 | NR | NA | NA | 2.00E-15 |
| LST | rs10765775 | Educational attainment | 35361970 | chr11:95656362 | A=0.39, G=0.61 | 0.3878 | 0.0159004 | 0.012-0.02 | 2.00E-13 |
| LST | rs7969719 | Fish- and plant-related diet | 32066663 | chr12:109883577 | C=0.677, T=0.323 | NR | 0.0189213 | 0.014-0.024 | 5.00E-13 |
| LST | rs7969719 | Leisure screen time | 36071172 | chr12:109883577 | C=0.677, T=0.323 | 0.687 | 0.027 | 0.019-0.035 | 4.00E-13 |
| LST | rs73405293 | Noncognitive aspects of educational attainment | 33414549 | chr12:117522917 | A=0.167, G=0.833 | 0.166998 | 0.053416945 | 0.035-0.072 | 1.00E-08 |
| LST | rs73405293 | Educational attainment (years of education) | 30038396 | chr12:117522917 | A=0.167, G=0.833 | 0.1516 | 0.0113 | 0.0074-0.0152 | 7.00E-09 |
| LST | rs73405293 | Educational attainment | 35361970 | chr12:117522917 | A=0.167, G=0.833 | 0.1486 | 0.0114466 | 0.0086-0.0143 | 8.00E-15 |
| LST | rs10772643 | Leisure sedentary behaviour (television watching) | 32317632 | chr12:13415288 | C=0.107, T=0.893 | NR | 0.0247995 | 0.018-0.032 | 1.00E-12 |
| LST | rs10772643 | Leisure screen time | 36071172 | chr12:13415288 | C=0.107, T=0.893 | 0.892 | 0.039 | 0.027-0.051 | 6.00E-10 |
| LST | rs74996610 | Leisure sedentary behaviour (television watching) | 32317632 | chr12:24075007 | C=0.961, G=0.039 | NR | 0.0305519 | 0.02-0.041 | 4.00E-09 |
| LST | rs3759344 | Leisure screen time | 36071172 | chr12:6862646 | A=0.083, G=0.917 | 0.894 | 0.045 | 0.033-0.057 | 4.00E-13 |
| LST | rs3759344 | Educational attainment | 35361970 | chr12:6862646 | A=0.083, G=0.917 | 0.1002 | 0.00984225 | 0.0064-0.0133 | 2.00E-08 |
| LST | rs7991062 | Leisure sedentary behaviour (television watching) | 32317632 | chr13:100713194 | C=0.641, G=0.359 | NR | 0.0177381 | 0.013-0.022 | 9.00E-15 |
| LST | rs10400776 | Leisure screen time | 36071172 | chr14:97326366 | A=0.237, C=0.763 | 0.259 | 0.026 | 0.018-0.034 | 3.00E-09 |
| LST | rs12324720 | Leisure screen time | 36071172 | chr15:64092140 | A=0.195, G=0.805 | 0.176 | 0.027 | 0.017-0.037 | 4.00E-09 |
| LST | rs56151256 | Leisure screen time | 36071172 | chr15:78024806 | A=0.792, C=0.208 | 0.25 | 0.029 | 0.021-0.037 | 1.00E-10 |
| LST | rs11074658 | Leisure screen time | 36071172 | chr16:10308335 | C=0.398, T=0.602 | 0.591 | 0.024 | 0.016-0.032 | 9.00E-10 |
| LST | rs4889530 | Leisure screen time | 36071172 | chr16:31065918 | A=0.4, T=0.6 | 0.381 | 0.025 | 0.017-0.033 | 1.00E-10 |
| LST | rs1860337 | Leisure screen time | 36071172 | chr17:60851559 | C=0.445, T=0.555 | 0.595 | 0.025 | 0.017-0.033 | 9.00E-11 |
| LST | rs73420302 | Leisure screen time | 36071172 | chr17:77768068 | C=0.176, G=0.824 | 0.179 | 0.03 | 0.02-0.04 | 3.00E-09 |
| LST | rs12962050 | Appendicular lean mass | 33097823 | chr18:35179808 | A=0.641, G=0.359 | 0.6445 | 0.0153 | 0.011-0.019 | 2.00E-14 |
| LST | rs12962050 | Leisure screen time | 36071172 | chr18:35179808 | A=0.641, G=0.359 | 0.647 | 0.023 | 0.015-0.031 | 1.00E-10 |
| LST | rs12981974 | Leisure screen time | 36071172 | chr19:19388071 | C=0.146, G=0.854 | 0.857 | 0.033 | 0.021-0.045 | 4.00E-09 |
| LST | rs12463321 | Leisure screen time (MTAG) | 36071172 | chr19:37651855 | A=0.161, G=0.839 | NR | 0.032 | 0.022-0.042 | 3.00E-09 |
| LST | rs6857 | Body mass index | 30239722 | chr19:45392254 | C=0.835, T=0.165 | 0.1656 | 0.0246 | 0.02-0.029 | 6.00E-26 |
| LST | rs6857 | Body mass index | 30239722 | chr19:45392254 | C=0.835, T=0.165 | 0.1656 | 0.0246 | 0.02-0.029 | 6.00E-26 |
| LST | rs6857 | Age-related macular degeneration | 23326517 | chr19:45392254 | C=0.835, T=0.165 | 0.85 | 1.23 | 1.14-1.35 | 1.00E-06 |
| LST | rs6857 | Age-related macular degeneration | 23326517 | chr19:45392254 | C=0.835, T=0.165 | 0.85 | 1.23 | 1.14-1.35 | 1.00E-06 |
| LST | rs6857 | Brain amyloid deposition (PET imaging) | 32568366 | chr19:45392254 | C=0.835, T=0.165 | NR | 1.6742 | 1.54-1.81 | 6.00E-132 |
| LST | rs6857 | Body mass index | 31669095 | chr19:45392254 | C=0.835, T=0.165 | NR | NA | NA | 7.00E-23 |
| LST | rs6857 | Frontotemporal dementia | 26154020 | chr19:45392254 | C=0.835, T=0.165 | 0.13 | 1.7 | 1.46-1.94 | 8.00E-06 |
| LST | rs6857 | Body fat percentage | 26833246 | chr19:45392254 | C=0.835, T=0.165 | 0.83 | 0.048 | 0.032-0.064% | 7.00E-09 |
| LST | rs6857 | Body fat percentage | 26833246 | chr19:45392254 | C=0.835, T=0.165 | 0.83 | 0.058 | 0.035-0.081% | 7.00E-07 |
| LST | rs6857 | Body fat percentage | 26833246 | chr19:45392254 | C=0.835, T=0.165 | 0.83 | 0.053 | 0.035-0.071% | 7.00E-10 |
| LST | rs6857 | Body fat percentage | 26833246 | chr19:45392254 | C=0.835, T=0.165 | 0.83 | 0.062 | 0.038-0.086% | 2.00E-07 |
| LST | rs6857 | Fruit consumption | 32066663 | chr19:45392254 | C=0.835, T=0.165 | NR | 0.0201864 | 0.014-0.026 | 4.00E-10 |
| LST | rs6857 | Fish- and plant-related diet | 32066663 | chr19:45392254 | C=0.835, T=0.165 | NR | 0.0203136 | 0.014-0.027 | 3.00E-10 |
| LST | rs6857 | Alzheimer's disease biomarkers | 23419831 | chr19:45392254 | C=0.835, T=0.165 | 0.28 | NA | NA | 1.00E-10 |
| LST | rs6857 | LDL cholesterol levels x long total sleep time interaction (2df test) | 31719535 | chr19:45392254 | C=0.835, T=0.165 | NR | 6.459 | 5.83-7.08 | 3.00E-98 |
| LST | rs6857 | HDL cholesterol levels x long total sleep time interaction (2df test) | 31719535 | chr19:45392254 | C=0.835, T=0.165 | NR | 0.0197 | 0.016-0.023 | 2.00E-35 |
| LST | rs6857 | Dementia and core Alzheimer's disease neuropathologic changes | 25188341 | chr19:45392254 | C=0.835, T=0.165 | 0.2883 | 1.6136 | 1.42-1.8 | 2.00E-62 |
| LST | rs6857 | Neuritic plaque | 25188341 | chr19:45392254 | C=0.835, T=0.165 | 0.2661 | 1.2932 | 1.06-1.53 | 2.00E-27 |
| LST | rs6857 | Cerebral amyloid angiopathy | 25188341 | chr19:45392254 | C=0.835, T=0.165 | 0.3429 | 0.6708 | 0.53-0.81 | 3.00E-21 |
| LST | rs6857 | Dementia and core Alzheimer's disease neuropathologic changes | 25188341 | chr19:45392254 | C=0.835, T=0.165 | 0.2864 | 1.501 | 1.27-1.73 | 3.00E-38 |
| LST | rs6857 | Neurofibrillary tangles | 25188341 | chr19:45392254 | C=0.835, T=0.165 | 0.3285 | 0.6632 | 0.57-0.75 | 5.00E-47 |
| LST | rs6857 | Neurofibrillary tangles | 25188341 | chr19:45392254 | C=0.835, T=0.165 | 0.3219 | 0.7871 | 0.68-0.9 | 5.00E-44 |
| LST | rs6857 | Neuritic plaque | 25188341 | chr19:45392254 | C=0.835, T=0.165 | 0.3155 | 0.9467 | 0.82-1.08 | 3.00E-47 |
| LST | rs6857 | Alzheimer's disease | 28183528 | chr19:45392254 | C=0.835, T=0.165 | NR | 1.27 | 1.15-1.40 | 6.00E-07 |
| LST | rs6857 | Alzheimer's disease | 28183528 | chr19:45392254 | C=0.835, T=0.165 | NR | 3.22 | 2.45-4.24 | 7.00E-18 |
| LST | rs6857 | Verbal declarative memory | 25648963 | chr19:45392254 | C=0.835, T=0.165 | 0.16 | NA | NA | 4.00E-13 |
| LST | rs6857 | Verbal declarative memory | 25648963 | chr19:45392254 | C=0.835, T=0.165 | 0.16 | NA | NA | 4.00E-13 |
| LST | rs6857 | Coronary artery disease or plasminogen activator inhibitor 1 levels (pleiotropy) | 35285134 | chr19:45392254 | C=0.835, T=0.165 | NR | NA | NA | 9.00E-13 |
| LST | rs6857 | Sphingomyelin (d18:0/22:0) levels | 35668104 | chr19:45392254 | C=0.835, T=0.165 | NR | 0.184 | 0.13-0.24 | 7.00E-10 |
| LST | rs6857 | Phosphatidylcholine (36:0) levels | 35668104 | chr19:45392254 | C=0.835, T=0.165 | NR | 0.183 | 0.12-0.24 | 9.00E-10 |
| LST | rs6857 | Cognitive impairment | 31201950 | chr19:45392254 | C=0.835, T=0.165 | 0.14 | 1.79 | 1.38-2.53 | 4.00E-12 |
| LST | rs6857 | C-reactive protein levels | 31201950 | chr19:45392254 | C=0.835, T=0.165 | 0.16 | 2.1 | 1.53-3.37 | 2.00E-07 |
| LST | rs6857 | Type 2 diabetes | 27189021 | chr19:45392254 | C=0.835, T=0.165 | 0.844 | 1.12 | 1.08-1.16 | 7.00E-09 |
| LST | rs6857 | Body mass index | 30595370 | chr19:45392254 | C=0.835, T=0.165 | NR | NA | NA | 3.00E-23 |
| LST | rs6857 | Predicted visceral adipose tissue | 31501611 | chr19:45392254 | C=0.835, T=0.165 | 0.1716 | 0.029516095 | 0.023-0.036 | 3.00E-19 |
| LST | rs6857 | LDL cholesterol levels | 32203549 | chr19:45392254 | C=0.835, T=0.165 | 0.829568 | 0.157677 | 0.15-0.16 | 0 |
| LST | rs6857 | Verbal short term memory | 35974141 | chr19:45392254 | C=0.835, T=0.165 | 0.1611 | 5.508 | NA | 4.00E-08 |
| LST | rs6857 | Verbal learning | 35974141 | chr19:45392254 | C=0.835, T=0.165 | 0.1611 | 5.482 | NA | 4.00E-08 |
| LST | rs6857 | Cerebral amyloid deposition (PET imaging) | 30361487 | chr19:45392254 | C=0.835, T=0.165 | 0.2 | 0.15 | NA | 2.00E-20 |
| LST | rs6857 | Leisure screen time | 36071172 | chr19:45392254 | C=0.835, T=0.165 | 0.169 | 0.037 | 0.027-0.047 | 6.00E-15 |
| LST | rs6857 | 1-margaroyl-GPC (17:0) levels | 36357675 | chr19:45392254 | C=0.835, T=0.165 | 0.1709 | 0.086707644 | 0.061-0.113 | 6.00E-11 |
| LST | rs6857 | Verbal short term memory (paragraph recall test) | 35974141 | chr19:45392254 | C=0.835, T=0.165 | 0.1611 | 6.87 | NA | 6.00E-12 |
| LST | rs6857 | Verbal learning (visual presentation) | 35974141 | chr19:45392254 | C=0.835, T=0.165 | 0.1611 | 5.192 | NA | 2.00E-07 |
| LST | rs6857 | BMI (standard GWA) | 37106081 | chr19:45392254 | C=0.835, T=0.165 | NR | 0.10082 | 0.068-0.133 | 1.00E-09 |
| LST | rs6857 | Plasma amyloid beta 42 levels | 37208024 | chr19:45392254 | C=0.835, T=0.165 | NR | 0.211 | NA | 3.00E-08 |
| LST | rs6857 | D-3-phosphoglycerate dehydrogenase levels | 34648354 | chr19:45392254 | C=0.835, T=0.165 | 0.16 | 0.743 | 0.71-0.78 | 0 |
| LST | rs6857 | Retinol dehydrogenase 16 levels | 34648354 | chr19:45392254 | C=0.835, T=0.165 | 0.16 | 0.177 | 0.14-0.21 | 4.00E-22 |
| LST | rs6857 | Conjugated linoleic acid | 38448586 | chr19:45392254 | C=0.835, T=0.165 | 0.1697 | 0.0618 | 0.047-0.077 | 1.00E-15 |
| LST | rs6857 | Body mass index | 37280435 | chr19:45392254 | C=0.835, T=0.165 | NR | 0.0250844 | 0.02-0.03 | 5.00E-22 |
| LST | rs4303732 | Moderate-to-vigorous intensity physical activity during leisure time | 36071172 | chr2:100830040 | C=0.391, T=0.609 | 0.405 | 0.016 | 0.01-0.022 | 7.00E-07 |
| LST | rs4303732 | Leisure screen time | 36071172 | chr2:100830040 | C=0.391, T=0.609 | 0.402 | 0.027 | 0.019-0.035 | 5.00E-14 |
| LST | rs4303732 | Body mass index (MTAG) | 36376304 | chr2:100830040 | C=0.391, T=0.609 | NR | 0.016996 | 0.014-0.02 | 2.00E-22 |
| LST | rs62151809 | Leisure screen time | 36071172 | chr2:104433256 | C=0.492, T=0.508 | 0.547 | 0.023 | 0.015-0.031 | 4.00E-09 |
| LST | rs62151809 | Genetic generalized epilepsy | 37653029 | chr2:104433256 | C=0.492, T=0.508 | NR | NA | NA | 1.00E-11 |
| LST | rs114590429 | Leisure screen time | 36071172 | chr2:166176789 | A=0.107, C=0.893 | 0.884 | 0.038 | 0.026-0.05 | 3.00E-10 |
| LST | rs12617870 | Leisure screen time | 36071172 | chr2:193746283 | G=0.468, T=0.532 | 0.462 | 0.026 | 0.018-0.034 | 7.00E-14 |
| LST | rs36079846 | Leisure screen time | 36071172 | chr2:215367159 | C=0.455, T=0.545 | 0.521 | 0.024 | 0.016-0.032 | 5.00E-10 |
| LST | rs10189857 | Intelligence (MTAG) | 29326435 | chr2:60713235 | A=0.58, G=0.42 | NR | 0.023793397 | 0.018-0.03 | 6.00E-16 |
| LST | rs10189857 | Educational attainment (years of education) | 30595370 | chr2:60713235 | A=0.58, G=0.42 | NR | NA | NA | 7.00E-16 |
| LST | rs10189857 | Intelligence | 29942086 | chr2:60713235 | A=0.58, G=0.42 | NR | 6.908 | - | 5.00E-12 |
| LST | rs10189857 | Leisure sedentary behaviour (television watching) | 32317632 | chr2:60713235 | A=0.58, G=0.42 | NR | 0.020463 | 0.016-0.025 | 6.00E-21 |
| LST | rs10189857 | Driving (hours per day) | 32317632 | chr2:60713235 | A=0.58, G=0.42 | NR | 0.0116306 | 0.0072-0.016 | 2.00E-07 |
| LST | rs10189857 | Highest math class taken | 30038396 | chr2:60713235 | A=0.58, G=0.42 | 0.5739 | 0.0136 | 0.0091-0.0181 | 3.00E-09 |
| LST | rs10189857 | Highest math class taken (MTAG) | 30038396 | chr2:60713235 | A=0.58, G=0.42 | 0.5739 | 0.0185 | 0.015-0.022 | 1.00E-28 |
| LST | rs10189857 | Cognitive aspects of educational attainment | 33414549 | chr2:60713235 | A=0.58, G=0.42 | 0.420477 | 0.050195884 | 0.038-0.063 | 4.00E-15 |
| LST | rs10189857 | Educational attainment (years of education) | 30038396 | chr2:60713235 | A=0.58, G=0.42 | 0.5654 | 0.0158 | 0.013-0.019 | 5.00E-29 |
| LST | rs10189857 | Educational attainment | 34855049 | chr2:60713235 | A=0.58, G=0.42 | NR | NA | NA | 9.00E-19 |
| LST | rs10189857 | Height | 30595370 | chr2:60713235 | A=0.58, G=0.42 | NR | NA | NA | 3.00E-12 |
| LST | rs10189857 | Educational attainment (MTAG) | 30038396 | chr2:60713235 | A=0.58, G=0.42 | 0.5654 | 0.0162 | 0.014-0.019 | 9.00E-35 |
| LST | rs10189857 | Cognitive performance (MTAG) | 30038396 | chr2:60713235 | A=0.58, G=0.42 | 0.5707 | 0.0252 | 0.021-0.03 | 1.00E-27 |
| LST | rs10189857 | Cognitive performance | 30038396 | chr2:60713235 | A=0.58, G=0.42 | 0.5707 | 0.0229 | 0.017-0.029 | 2.00E-15 |
| LST | rs10189857 | Leisure screen time | 36071172 | chr2:60713235 | A=0.58, G=0.42 | 0.565 | 0.027 | 0.019-0.035 | 8.00E-15 |
| LST | rs10189857 | Educational attainment | 35361970 | chr2:60713235 | A=0.58, G=0.42 | 0.5701 | 0.0137772 | 0.012-0.016 | 8.00E-39 |
| LST | rs10189857 | Attention deficit hyperactivity disorder or autism spectrum disorder or intelligence (pleiotropy) | 35764056 | chr2:60713235 | A=0.58, G=0.42 | NR | NA | NA | 6.00E-12 |
| LST | rs10189857 | Principal component-derived dietary pattern 1 | 32193382 | chr2:60713235 | A=0.58, G=0.42 | 0.567707 | 0.0303838 | 0.021-0.04 | 1.00E-10 |
| LST | rs10189857 | Height | 36224396 | chr2:60713235 | A=0.58, G=0.42 | 0.4587 | 0.0091 | 0.0079-0.0103 | 2.00E-47 |
| LST | rs6102913 | Educational attainment (years of education) | 30595370 | chr20:41202958 | C=0.515, T=0.485 | NR | NA | NA | 3.00E-08 |
| LST | rs6102913 | Educational attainment (years of education) | 30038396 | chr20:41202958 | C=0.515, T=0.485 | 0.4845 | 0.0085 | 0.0058-0.0112 | 1.00E-09 |
| LST | rs17801257 | Educational attainment (MTAG) | 30038396 | chr20:58892520 | A=0.144, G=0.856 | 0.1258 | 0.0118 | 0.0079-0.0157 | 2.00E-09 |
| LST | rs6010651 | Menarche (age at onset) | 30595370 | chr20:62418243 | A=0.626, C=0.374 | NR | NA | NA | 1.00E-08 |
| LST | rs6010651 | Leisure screen time | 36071172 | chr20:62418243 | A=0.626, C=0.374 | 0.38 | 0.024 | 0.016-0.032 | 3.00E-09 |
| LST | rs9867121 | Leisure sedentary behaviour (television watching) | 32317632 | chr3:114631548 | A=0.179, C=0.821 | NR | 0.0194994 | 0.014-0.025 | 4.00E-12 |
| LST | rs9867121 | Leisure screen time | 36071172 | chr3:114631548 | A=0.179, C=0.821 | 0.183 | 0.032 | 0.022-0.042 | 2.00E-10 |
| LST | rs7615206 | Sedentary behavior at work | 36071172 | chr3:49937505 | C=0.421, T=0.579 | 0.584 | 0.019 | 0.011-0.027 | 8.00E-06 |
| LST | rs7615206 | Leisure screen time | 36071172 | chr3:49937505 | C=0.421, T=0.579 | 0.57 | 0.035 | 0.027-0.043 | 2.00E-22 |
| LST | rs76267866 | Educational attainment (years of education) | 30038396 | chr3:70540347 | A=0.813, T=0.187 | 0.7926 | 0.0104 | 0.0071-0.0137 | 2.00E-09 |
| LST | rs76267866 | Leisure screen time | 36071172 | chr3:70540347 | A=0.813, T=0.187 | 0.792 | 0.03 | 0.02-0.04 | 4.00E-10 |
| LST | rs62244886 | Salami liking | 35585065 | chr3:71587392 | C=0.59, G=0.41 | 0.3989 | 0.020448 | 0.013-0.028 | 3.00E-08 |
| LST | rs62244886 | Leisure screen time | 36071172 | chr3:71587392 | C=0.59, G=0.41 | 0.396 | 0.024 | 0.016-0.032 | 1.00E-09 |
| LST | rs7430216 | Leisure screen time | 36071172 | chr3:75201030 | C=0.797, T=0.203 | 0.777 | 0.025 | 0.017-0.033 | 3.00E-09 |
| LST | rs1375561 | Cognitive performance (MTAG) | 30038396 | chr3:85658230 | C=0.356, T=0.644 | 0.6538 | 0.0191 | 0.014-0.024 | 2.00E-15 |
| LST | rs1375561 | Leisure screen time | 36071172 | chr3:85658230 | C=0.356, T=0.644 | 0.347 | 0.023 | 0.015-0.031 | 3.00E-10 |
| LST | rs1375561 | Body mass index (MTAG) | 36376304 | chr3:85658230 | C=0.356, T=0.644 | NR | 0.017446 | 0.014-0.021 | 9.00E-25 |
| LST | rs1375561 | Body mass index | 36581621 | chr3:85658230 | C=0.356, T=0.644 | NR | 0.0152 | 0.012-0.018 | 3.00E-23 |
| LST | rs13107325 | Systolic blood pressure | 27618447 | chr4:103188709 | C=0.92, T=0.08 | 0.0603 | 0.042 | NA | 1.00E-07 |
| LST | rs13107325 | Body mass index | 26961502 | chr4:103188709 | C=0.92, T=0.08 | NR | 0.04 | 0.024-0.056 | 4.00E-07 |
| LST | rs13107325 | Mean arterial pressure | 27618448 | chr4:103188709 | C=0.92, T=0.08 | 0.062717118 | 0.6347496 | 0.45-0.82 | 1.00E-11 |
| LST | rs13107325 | Body mass index in physically active individuals | 28448500 | chr4:103188709 | C=0.92, T=0.08 | 0.0781 | 0.0661 | 0.04-0.092/2 | 4.00E-07 |
| LST | rs13107325 | Body mass index in physically active individuals | 28448500 | chr4:103188709 | C=0.92, T=0.08 | 0.0765 | 0.0531 | 0.036-0.07/2 | 1.00E-09 |
| LST | rs13107325 | Body mass index in physically active individuals | 28448500 | chr4:103188709 | C=0.92, T=0.08 | 0.0754 | 0.0508 | 0.034-0.068/2 | 4.00E-09 |
| LST | rs13107325 | Body mass index in physically active individuals | 28448500 | chr4:103188709 | C=0.92, T=0.08 | 0.0875 | 0.0868 | 0.051-0.122/2 | 2.00E-06 |
| LST | rs13107325 | Intelligence (MTAG) | 29326435 | chr4:103188709 | C=0.92, T=0.08 | NR | 0.05044097 | 0.039-0.062 | 3.00E-18 |
| LST | rs13107325 | Balding type 1 | 30595370 | chr4:103188709 | C=0.92, T=0.08 | NR | NA | NA | 2.00E-10 |
| LST | rs13107325 | Waist-to-hip ratio adjusted for BMI | 30239722 | chr4:103188709 | C=0.92, T=0.08 | 0.0808 | 0.0306 | 0.024-0.037 | 4.00E-19 |
| LST | rs13107325 | Waist-to-hip ratio adjusted for BMI | 30239722 | chr4:103188709 | C=0.92, T=0.08 | 0.0808 | 0.0306 | 0.024-0.037 | 4.00E-19 |
| LST | rs13107325 | Alcohol consumption (drinks per week) | 30679032 | chr4:103188709 | C=0.92, T=0.08 | 0.0721 | 0.0383 | 0.029-0.047 | 5.00E-17 |
| LST | rs13107325 | Body mass index | 30239722 | chr4:103188709 | C=0.92, T=0.08 | 0.082 | 0.0468 | 0.041-0.053 | 4.00E-47 |
| LST | rs13107325 | Body mass index | 30595370 | chr4:103188709 | C=0.92, T=0.08 | NR | NA | NA | 1.00E-36 |
| LST | rs13107325 | Body mass index | 30239722 | chr4:103188709 | C=0.92, T=0.08 | 0.082 | 0.0468 | 0.041-0.053 | 4.00E-47 |
| LST | rs13107325 | Body mass index | 30239722 | chr4:103188709 | C=0.92, T=0.08 | 0.082 | 0.0468 | 0.041-0.053 | 4.00E-47 |
| LST | rs13107325 | Systolic blood pressure x alcohol consumption interaction (2df test) | 29912962 | chr4:103188709 | C=0.92, T=0.08 | 0.07 | NA | NA | 1.00E-15 |
| LST | rs13107325 | Blood pressure | 21909110 | chr4:103188709 | C=0.92, T=0.08 | 0.12 | 0.633 | 0.44-0.82 | 1.00E-10 |
| LST | rs13107325 | HDL cholesterol | 20686565 | chr4:103188709 | C=0.92, T=0.08 | 0.07 | 0.84 | 0.53-1.15/ | 7.00E-11 |
| LST | rs13107325 | Systolic blood pressure | 21909115 | chr4:103188709 | C=0.92, T=0.08 | 0.05 | 0.981 | NA | 3.00E-14 |
| LST | rs13107325 | Diastolic blood pressure | 21909115 | chr4:103188709 | C=0.92, T=0.08 | 0.05 | 0.684 | NA | 2.00E-17 |
| LST | rs13107325 | Hypertension | 21909115 | chr4:103188709 | C=0.92, T=0.08 | 0.05 | 0.105 | NA | 5.00E-07 |
| LST | rs13107325 | Body mass index | 20935630 | chr4:103188709 | C=0.92, T=0.08 | 0.07 | 0.19 | 0.11-0.27/2 | 2.00E-13 |
| LST | rs13107325 | Lung function (FVC) | 30595370 | chr4:103188709 | C=0.92, T=0.08 | NR | NA | NA | 9.00E-18 |
| LST | rs13107325 | Systolic blood pressure | 30595370 | chr4:103188709 | C=0.92, T=0.08 | NR | NA | NA | 1.00E-36 |
| LST | rs13107325 | Red blood cell count | 32888493 | chr4:103188709 | C=0.92, T=0.08 | 0.074343 | 0.029182 | 0.022-0.036 | 1.00E-16 |
| LST | rs13107325 | Alcohol use disorder | 32451486 | chr4:103188709 | C=0.92, T=0.08 | 0.0792 | 8.864 | NA | 8.00E-19 |
| LST | rs13107325 | Schizophrenia (MTAG) | 32606422 | chr4:103188709 | C=0.92, T=0.08 | NR | NA | NA | 8.00E-11 |
| LST | rs13107325 | Schizophrenia (MTAG) | 32606422 | chr4:103188709 | C=0.92, T=0.08 | NR | NA | NA | 5.00E-09 |
| LST | rs13107325 | Body mass index | 31669095 | chr4:103188709 | C=0.92, T=0.08 | NR | NA | NA | 8.00E-39 |
| LST | rs13107325 | Liver fibrosis and steatohepatitis severity (MRI cT1 measure) | 32247823 | chr4:103188709 | C=0.92, T=0.08 | 0.07 | 0.544 | 0.5-0.59 | 1.00E-133 |
| LST | rs13107325 | Red blood cell count | 32888493 | chr4:103188709 | C=0.92, T=0.08 | 0.0715291 | NA | NA | 2.00E-14 |
| LST | rs13107325 | Diastolic blood pressure | 27618452 | chr4:103188709 | C=0.92, T=0.08 | 0.07 | 0.602 | 0.45-0.75 | 2.00E-14 |
| LST | rs13107325 | Systolic blood pressure | 27618452 | chr4:103188709 | C=0.92, T=0.08 | 0.07 | 0.837 | 0.59-1.09 | 5.00E-11 |
| LST | rs13107325 | Childhood body mass index | 26604143 | chr4:103188709 | C=0.92, T=0.08 | 0.07 | 0.081 | 0.05-0.112 | 4.00E-07 |
| LST | rs13107325 | Childhood body mass index | 26604143 | chr4:103188709 | C=0.92, T=0.08 | NR | NA | NA | 1.00E-08 |
| LST | rs13107325 | NT-proBNP levels in acute coronary syndrome | 26908625 | chr4:103188709 | C=0.92, T=0.08 | 0.078 | 0.216 | 0.15-0.28/ | 6.00E-10 |
| LST | rs13107325 | Type 2 diabetes | 32541925 | chr4:103188709 | C=0.92, T=0.08 | 0.9265 | 0.0473 | 0.03-0.064 | 3.00E-08 |
| LST | rs13107325 | Platelet distribution width | 32888494 | chr4:103188709 | C=0.92, T=0.08 | 0.074663 | 0.048567016 | 0.04-0.057 | 1.00E-29 |
| LST | rs13107325 | Brain morphology (MOSTest) | 32665545 | chr4:103188709 | C=0.92, T=0.08 | NR | NA | NA | 3.00E-124 |
| LST | rs13107325 | Cortical surface area (MOSTest) | 32665545 | chr4:103188709 | C=0.92, T=0.08 | NR | NA | NA | 5.00E-20 |
| LST | rs13107325 | Subcortical volume (MOSTest) | 32665545 | chr4:103188709 | C=0.92, T=0.08 | NR | NA | NA | 5.00E-73 |
| LST | rs13107325 | Cortical thickness (MOSTest) | 32665545 | chr4:103188709 | C=0.92, T=0.08 | NR | NA | NA | 8.00E-38 |
| LST | rs13107325 | Brain morphology (min-P) | 32665545 | chr4:103188709 | C=0.92, T=0.08 | NR | NA | NA | 2.00E-23 |
| LST | rs13107325 | Subcortical volume (min-P) | 32665545 | chr4:103188709 | C=0.92, T=0.08 | NR | NA | NA | 1.00E-23 |
| LST | rs13107325 | Eosinophil percentage of white cells | 32888494 | chr4:103188709 | C=0.92, T=0.08 | 0.074637 | 0.027977897 | 0.02-0.036 | 4.00E-11 |
| LST | rs13107325 | Waist circumference adjusted for body mass index | 31669095 | chr4:103188709 | C=0.92, T=0.08 | NR | NA | NA | 5.00E-20 |
| LST | rs13107325 | Mean corpuscular volume | 32888494 | chr4:103188709 | C=0.92, T=0.08 | 0.074597 | 0.0265556 | 0.019-0.034 | 1.00E-10 |
| LST | rs13107325 | Hematocrit | 32888494 | chr4:103188709 | C=0.92, T=0.08 | 0.07459 | 0.049112022 | 0.041-0.057 | 2.00E-31 |
| LST | rs13107325 | Hemoglobin concentration | 32888493 | chr4:103188709 | C=0.92, T=0.08 | 0.071471 | NA | NA | 1.00E-37 |
| LST | rs13107325 | Hematocrit | 32888493 | chr4:103188709 | C=0.92, T=0.08 | 0.074412 | 0.046911 | 0.04-0.054 | 2.00E-40 |
| LST | rs13107325 | Hematocrit | 32888493 | chr4:103188709 | C=0.92, T=0.08 | 0.0715958 | NA | NA | 3.00E-38 |
| LST | rs13107325 | Eczema | 30595370 | chr4:103188709 | C=0.92, T=0.08 | NR | NA | NA | 2.00E-10 |
| LST | rs13107325 | Hemoglobin concentration | 32888493 | chr4:103188709 | C=0.92, T=0.08 | 0.074272 | 0.04586 | 0.039-0.053 | 1.00E-38 |
| LST | rs13107325 | Hemoglobin | 32888494 | chr4:103188709 | C=0.92, T=0.08 | 0.074589 | 0.049483463 | 0.041-0.058 | 7.00E-32 |
| LST | rs13107325 | Alcohol consumption | 31358974 | chr4:103188709 | C=0.92, T=0.08 | 0.07 | 0.016 | 0.012-0.02 | 1.00E-15 |
| LST | rs13107325 | LDL cholesterol levels | 32203549 | chr4:103188709 | C=0.92, T=0.08 | 0.925102 | 0.0245488 | 0.017-0.032 | 6.00E-10 |
| LST | rs13107325 | Body mass index | 29381148 | chr4:103188709 | C=0.92, T=0.08 | NR | 0.011 | NA | 8.00E-06 |
| LST | rs13107325 | Brain region volumes | 31676860 | chr4:103188709 | C=0.92, T=0.08 | NR | NA | NA | 2.00E-15 |
| LST | rs13107325 | Parkinson's disease | 32201043 | chr4:103188709 | C=0.92, T=0.08 | NR | 0.1495 | 0.095-0.204 | 7.00E-08 |
| LST | rs13107325 | Schizophrenia | 31740837 | chr4:103188709 | C=0.92, T=0.08 | NR | 1.16637 | 1.12-1.21 | 4.00E-13 |
| LST | rs13107325 | Waist-hip ratio | 30595370 | chr4:103188709 | C=0.92, T=0.08 | NR | NA | NA | 1.00E-20 |
| LST | rs13107325 | Nucleus accumbens volume | 31636452 | chr4:103188709 | C=0.92, T=0.08 | 0.0638 | 6.15 | NA | 8.00E-10 |
| LST | rs13107325 | Triglyceride levels | 32203549 | chr4:103188709 | C=0.92, T=0.08 | 0.925092 | 0.0295524 | 0.022-0.037 | 6.00E-15 |
| LST | rs13107325 | Brain region volumes | 31676860 | chr4:103188709 | C=0.92, T=0.08 | NR | NA | NA | 2.00E-18 |
| LST | rs13107325 | Apolipoprotein A1 levels | 32203549 | chr4:103188709 | C=0.92, T=0.08 | 0.925003 | 0.0715695 | 0.064-0.079 | 5.00E-82 |
| LST | rs13107325 | Hemoglobin levels | 32327693 | chr4:103188709 | C=0.92, T=0.08 | NR | 0.048 | NA | 2.00E-24 |
| LST | rs13107325 | HDL cholesterol levels | 32203549 | chr4:103188709 | C=0.92, T=0.08 | 0.925055 | 0.0801624 | 0.073-0.087 | 2.00E-108 |
| LST | rs13107325 | Alcohol consumption (heavy vs. light/non-drinkers) | 31998841 | chr4:103188709 | C=0.92, T=0.08 | 0.928 | 5.798 | NA | 7.00E-09 |
| LST | rs13107325 | HDL cholesterol | 24097068 | chr4:103188709 | C=0.92, T=0.08 | 0.08 | 0.071 | NA | 1.00E-15 |
| LST | rs13107325 | Body mass index (joint analysis main effects and physical activity interaction) | 28448500 | chr4:103188709 | C=0.92, T=0.08 | NR | NA | NA | 2.00E-08 |
| LST | rs13107325 | Body mass index (joint analysis main effects and physical activity interaction) | 28448500 | chr4:103188709 | C=0.92, T=0.08 | NR | NA | NA | 2.00E-10 |
| LST | rs13107325 | Body mass index (joint analysis main effects and smoking interaction) | 28443625 | chr4:103188709 | C=0.92, T=0.08 | 0.0737 | NA | NA | 2.00E-10 |
| LST | rs13107325 | Body mass index (joint analysis main effects and smoking interaction) | 28443625 | chr4:103188709 | C=0.92, T=0.08 | 0.0737 | NA | NA | 1.00E-08 |
| LST | rs13107325 | Body mass index (joint analysis main effects and physical activity interaction) | 28448500 | chr4:103188709 | C=0.92, T=0.08 | NR | NA | NA | 2.00E-09 |
| LST | rs13107325 | Body mass index (joint analysis main effects and physical activity interaction) | 28448500 | chr4:103188709 | C=0.92, T=0.08 | NR | NA | NA | 8.00E-10 |
| LST | rs13107325 | BMI in non-smokers | 28443625 | chr4:103188709 | C=0.92, T=0.08 | 0.0737 | 0.0449 | 0.028-0.062/2 | 1.00E-07 |
| LST | rs13107325 | BMI in non-smokers | 28443625 | chr4:103188709 | C=0.92, T=0.08 | 0.0737 | 0.0574 | 0.035-0.08/2 | 7.00E-07 |
| LST | rs13107325 | Systolic blood pressure | 28739976 | chr4:103188709 | C=0.92, T=0.08 | 0.065 | 0.933 | 0.64-1.23 | 6.00E-10 |
| LST | rs13107325 | Body mass index | 28448500 | chr4:103188709 | C=0.92, T=0.08 | 0.0754 | 0.0698 | 0.047-0.092/2 | 1.00E-09 |
| LST | rs13107325 | Diastolic blood pressure | 28739976 | chr4:103188709 | C=0.92, T=0.08 | 0.065 | 0.686 | 0.51-0.87 | 1.00E-13 |
| LST | rs13107325 | BMI (adjusted for smoking behaviour) | 28443625 | chr4:103188709 | C=0.92, T=0.08 | 0.0737 | 0.0469 | 0.032-0.062/2 | 3.00E-10 |
| LST | rs13107325 | BMI (adjusted for smoking behaviour) | 28443625 | chr4:103188709 | C=0.92, T=0.08 | 0.0737 | 0.0607 | 0.041-0.081/2 | 4.00E-09 |
| LST | rs13107325 | Body mass index | 28448500 | chr4:103188709 | C=0.92, T=0.08 | 0.0765 | 0.0741 | 0.051-0.097/2 | 5.00E-10 |
| LST | rs13107325 | Body mass index | 28448500 | chr4:103188709 | C=0.92, T=0.08 | 0.0754 | 0.0488 | 0.034-0.064/2 | 3.00E-10 |
| LST | rs13107325 | Body mass index | 28448500 | chr4:103188709 | C=0.92, T=0.08 | 0.0765 | 0.0505 | 0.035-0.066/2 | 2.00E-10 |
| LST | rs13107325 | Total cholesterol levels | 29507422 | chr4:103188709 | C=0.92, T=0.08 | 0.925 | 0.053 | NA | 1.00E-09 |
| LST | rs13107325 | Total cholesterol levels | 29507422 | chr4:103188709 | C=0.92, T=0.08 | NR | 0.055 | NA | 5.00E-11 |
| LST | rs13107325 | Intelligence | 29942086 | chr4:103188709 | C=0.92, T=0.08 | NR | 9.494 | - | 2.00E-21 |
| LST | rs13107325 | Refractive error | 32231278 | chr4:103188709 | C=0.92, T=0.08 | NR | NA | NA | 4.00E-17 |
| LST | rs13107325 | Low density lipoprotein cholesterol levels | 32154731 | chr4:103188709 | C=0.92, T=0.08 | NR | 0.0313 | 0.023-0.04 | 1.00E-10 |
| LST | rs13107325 | Triglyceride levels | 32154731 | chr4:103188709 | C=0.92, T=0.08 | NR | 0.031 | 0.022-0.04 | 5.00E-10 |
| LST | rs13107325 | Leisure sedentary behaviour (television watching) | 32317632 | chr4:103188709 | C=0.92, T=0.08 | NR | 0.0291705 | 0.021-0.037 | 2.00E-12 |
| LST | rs13107325 | Spherical equivalent | 32352494 | chr4:103188709 | C=0.92, T=0.08 | NR | 0.13 | 0.091-0.169 | 3.00E-10 |
| LST | rs13107325 | Youthful appearance (self-reported) | 32339537 | chr4:103188709 | C=0.92, T=0.08 | 0.924534 | 0.94332457 | NA | 1.00E-12 |
| LST | rs13107325 | Hypertension | 31879980 | chr4:103188709 | C=0.92, T=0.08 | NR | 0.07963385 | NA | 5.00E-12 |
| LST | rs13107325 | Waist-to-hip ratio adjusted for BMI | 31669095 | chr4:103188709 | C=0.92, T=0.08 | NR | NA | NA | 3.00E-19 |
| LST | rs13107325 | Diastolic blood pressure | 29615537 | chr4:103188709 | C=0.92, T=0.08 | 0.0818 | 0.91369206 | 0.62-1.21 | 9.00E-10 |
| LST | rs13107325 | Red blood cell count | 30595370 | chr4:103188709 | C=0.92, T=0.08 | NR | NA | NA | 6.00E-24 |
| LST | rs13107325 | General cognitive ability | 29844566 | chr4:103188709 | C=0.92, T=0.08 | NR | 8.327 | - | 8.00E-17 |
| LST | rs13107325 | Body mass index | 25673413 | chr4:103188709 | C=0.92, T=0.08 | 0.072 | 0.048 | 0.034-0.061/2 | 2.00E-12 |
| LST | rs13107325 | Body mass index | 25673413 | chr4:103188709 | C=0.92, T=0.08 | 0.072 | 0.047 | 0.034-0.06/2 | 1.00E-12 |
| LST | rs13107325 | Schizophrenia | 26198764 | chr4:103188709 | C=0.92, T=0.08 | NR | 1.16 | NA | 2.00E-12 |
| LST | rs13107325 | Body mass index | 25673413 | chr4:103188709 | C=0.92, T=0.08 | 0.074 | 0.053 | 0.035-0.07/2 | 3.00E-09 |
| LST | rs13107325 | Body mass index | 25673413 | chr4:103188709 | C=0.92, T=0.08 | 0.071 | 0.045 | 0.028-0.062/2 | 3.00E-07 |
| LST | rs13107325 | Diastolic blood pressure | 27841878 | chr4:103188709 | C=0.92, T=0.08 | NR | 0.477 | NA | 8.00E-11 |
| LST | rs13107325 | Diastolic blood pressure | 27841878 | chr4:103188709 | C=0.92, T=0.08 | NR | 0.466 | NA | 4.00E-11 |
| LST | rs13107325 | Systolic blood pressure | 27841878 | chr4:103188709 | C=0.92, T=0.08 | NR | 0.719 | NA | 2.00E-21 |
| LST | rs13107325 | Diastolic blood pressure | 27841878 | chr4:103188709 | C=0.92, T=0.08 | NR | 0.55 | NA | 5.00E-33 |
| LST | rs13107325 | Systolic blood pressure | 27841878 | chr4:103188709 | C=0.92, T=0.08 | NR | 0.643 | NA | 3.00E-09 |
| LST | rs13107325 | Systolic blood pressure | 27841878 | chr4:103188709 | C=0.92, T=0.08 | NR | 0.646 | NA | 1.00E-08 |
| LST | rs13107325 | Body mass index | 26426971 | chr4:103188709 | C=0.92, T=0.08 | 0.072500065 | 0.04919773 | NA | 4.00E-16 |
| LST | rs13107325 | High density lipoprotein cholesterol levels | 29507422 | chr4:103188709 | C=0.92, T=0.08 | 0.925 | 0.067 | NA | 6.00E-21 |
| LST | rs13107325 | High density lipoprotein cholesterol levels | 29507422 | chr4:103188709 | C=0.92, T=0.08 | NR | 0.067 | NA | 6.00E-22 |
| LST | rs13107325 | Low density lipoprotein cholesterol levels | 29507422 | chr4:103188709 | C=0.92, T=0.08 | NR | 0.038 | NA | 8.00E-06 |
| LST | rs13107325 | IDP T1 FAST ROIs R cerebellum IX | 33875891 | chr4:103188709 | C=0.92, T=0.08 | 0.07 | 0.275 | 0.24-0.31 | 1.00E-49 |
| LST | rs13107325 | IDP T1 FAST ROIs V cerebellum X | 33875891 | chr4:103188709 | C=0.92, T=0.08 | 0.07 | 0.257 | 0.22-0.29 | 1.00E-43 |
| LST | rs13107325 | IDP T1 FAST ROIs L ventral striatum | 33875891 | chr4:103188709 | C=0.92, T=0.08 | 0.07 | 0.43 | 0.39-0.47 | 1.00E-119 |
| LST | rs13107325 | IDP T1 FAST ROIs R cerebellum VI | 33875891 | chr4:103188709 | C=0.92, T=0.08 | 0.07 | 0.168 | 0.13-0.21 | 2.00E-19 |
| LST | rs13107325 | IDP T1 FAST ROIs V cerebellum VIIIb | 33875891 | chr4:103188709 | C=0.92, T=0.08 | 0.07 | 0.212 | 0.17-0.25 | 3.00E-30 |
| LST | rs13107325 | IDP T1 FAST ROIs L cerebellum IX | 33875891 | chr4:103188709 | C=0.92, T=0.08 | 0.07 | 0.274 | 0.24-0.31 | 4.00E-49 |
| LST | rs13107325 | IDP T1 FAST ROIs V cerebellum VIIIa | 33875891 | chr4:103188709 | C=0.92, T=0.08 | 0.07 | 0.2 | 0.16-0.24 | 6.00E-27 |
| LST | rs13107325 | IDP T1 FAST ROIs L cerebellum VIIIb | 33875891 | chr4:103188709 | C=0.92, T=0.08 | 0.07 | 0.226 | 0.19-0.26 | 5.00E-34 |
| LST | rs13107325 | IDP T1 FAST ROIs R cerebellum VIIIb | 33875891 | chr4:103188709 | C=0.92, T=0.08 | 0.07 | 0.215 | 0.18-0.25 | 7.00E-31 |
| LST | rs13107325 | IDP T1 FAST ROIs V cerebellum IX | 33875891 | chr4:103188709 | C=0.92, T=0.08 | 0.07 | 0.38 | 0.34-0.42 | 1.00E-93 |
| LST | rs13107325 | IDP T1 FAST ROIs R ventral striatum | 33875891 | chr4:103188709 | C=0.92, T=0.08 | 0.07 | 0.422 | 0.39-0.46 | 2.00E-115 |
| LST | rs13107325 | IDP T1 FAST ROIs brain stem | 33875891 | chr4:103188709 | C=0.92, T=0.08 | 0.07 | 0.194 | 0.16-0.23 | 2.00E-25 |
| LST | rs13107325 | IDP T1 FAST ROIs L cerebellum I-IV | 33875891 | chr4:103188709 | C=0.92, T=0.08 | 0.07 | 0.205 | 0.17-0.24 | 3.00E-28 |
| LST | rs13107325 | IDP T1 FAST ROIs L cerebellum V | 33875891 | chr4:103188709 | C=0.92, T=0.08 | 0.07 | 0.175 | 0.14-0.21 | 4.00E-21 |
| LST | rs13107325 | IDP T1 FAST ROIs L cerebellum VI | 33875891 | chr4:103188709 | C=0.92, T=0.08 | 0.07 | 0.164 | 0.13-0.2 | 1.00E-18 |
| LST | rs13107325 | IDP dMRI TBSS ICVF Cerebral peduncle R | 33875891 | chr4:103188709 | C=0.92, T=0.08 | 0.07 | 0.242 | 0.2-0.28 | 2.00E-36 |
| LST | rs13107325 | IDP dMRI TBSS ICVF Cerebral peduncle L | 33875891 | chr4:103188709 | C=0.92, T=0.08 | 0.07 | 0.244 | 0.21-0.28 | 4.00E-37 |
| LST | rs13107325 | IDP dMRI TBSS ICVF Anterior limb of internal capsule R | 33875891 | chr4:103188709 | C=0.92, T=0.08 | 0.07 | 0.131 | 0.094-0.168 | 9.00E-12 |
| LST | rs13107325 | IDP dMRI TBSS ICVF Anterior limb of internal capsule L | 33875891 | chr4:103188709 | C=0.92, T=0.08 | 0.07 | 0.144 | 0.11-0.18 | 8.00E-14 |
| LST | rs13107325 | aparc-DKTatlas rh thickness transversetemporal | 33875891 | chr4:103188709 | C=0.92, T=0.08 | 0.07 | 0.127 | 0.09-0.164 | 2.00E-11 |
| LST | rs13107325 | aparc-a2009s lh thickness G-cuneus | 33875891 | chr4:103188709 | C=0.92, T=0.08 | 0.07 | 0.16 | 0.12-0.2 | 3.00E-17 |
| LST | rs13107325 | aparc-a2009s rh thickness G-cuneus | 33875891 | chr4:103188709 | C=0.92, T=0.08 | 0.07 | 0.181 | 0.14-0.22 | 1.00E-21 |
| LST | rs13107325 | aparc-a2009s rh thickness G-oc-temp-med-Lingual | 33875891 | chr4:103188709 | C=0.92, T=0.08 | 0.07 | 0.14 | 0.1-0.18 | 2.00E-13 |
| LST | rs13107325 | aparc-a2009s rh thickness Pole-occipital | 33875891 | chr4:103188709 | C=0.92, T=0.08 | 0.07 | 0.186 | 0.15-0.22 | 1.00E-22 |
| LST | rs13107325 | aseg lh intensity Cerebellum-Cortex | 33875891 | chr4:103188709 | C=0.92, T=0.08 | 0.07 | 0.209 | 0.17-0.25 | 3.00E-28 |
| LST | rs13107325 | aseg lh intensity Putamen | 33875891 | chr4:103188709 | C=0.92, T=0.08 | 0.07 | 0.352 | 0.31-0.39 | 7.00E-77 |
| LST | rs13107325 | aseg lh intensity Pallidum | 33875891 | chr4:103188709 | C=0.92, T=0.08 | 0.07 | 0.243 | 0.21-0.28 | 1.00E-37 |
| LST | rs13107325 | BA-exvivo rh thickness BA4a | 33875891 | chr4:103188709 | C=0.92, T=0.08 | 0.07 | 0.145 | 0.11-0.18 | 3.00E-14 |
| LST | rs13107325 | BA-exvivo rh thickness V1 | 33875891 | chr4:103188709 | C=0.92, T=0.08 | 0.07 | 0.184 | 0.15-0.22 | 3.00E-22 |
| LST | rs13107325 | BA-exvivo rh thickness V2 | 33875891 | chr4:103188709 | C=0.92, T=0.08 | 0.07 | 0.186 | 0.15-0.22 | 1.00E-22 |
| LST | rs13107325 | aparc-DKTatlas lh thickness cuneus | 33875891 | chr4:103188709 | C=0.92, T=0.08 | 0.07 | 0.154 | 0.12-0.19 | 6.00E-16 |
| LST | rs13107325 | aparc-DKTatlas lh thickness lateraloccipital | 33875891 | chr4:103188709 | C=0.92, T=0.08 | 0.07 | 0.168 | 0.13-0.21 | 8.00E-19 |
| LST | rs13107325 | aparc-DKTatlas lh thickness paracentral | 33875891 | chr4:103188709 | C=0.92, T=0.08 | 0.07 | 0.127 | 0.09-0.164 | 2.00E-11 |
| LST | rs13107325 | aparc-DKTatlas lh thickness precentral | 33875891 | chr4:103188709 | C=0.92, T=0.08 | 0.07 | 0.112 | 0.075-0.149 | 4.00E-09 |
| LST | rs13107325 | aparc-DKTatlas rh thickness cuneus | 33875891 | chr4:103188709 | C=0.92, T=0.08 | 0.07 | 0.169 | 0.13-0.21 | 7.00E-19 |
| LST | rs13107325 | aparc-DKTatlas rh thickness lateraloccipital | 33875891 | chr4:103188709 | C=0.92, T=0.08 | 0.07 | 0.172 | 0.13-0.21 | 1.00E-19 |
| LST | rs13107325 | aparc-DKTatlas rh thickness lingual | 33875891 | chr4:103188709 | C=0.92, T=0.08 | 0.07 | 0.156 | 0.12-0.19 | 2.00E-16 |
| LST | rs13107325 | aparc-DKTatlas rh thickness paracentral | 33875891 | chr4:103188709 | C=0.92, T=0.08 | 0.07 | 0.139 | 0.1-0.18 | 3.00E-13 |
| LST | rs13107325 | aparc-DKTatlas rh thickness pericalcarine | 33875891 | chr4:103188709 | C=0.92, T=0.08 | 0.07 | 0.144 | 0.11-0.18 | 4.00E-14 |
| LST | rs13107325 | wg lh intensity-contrast entorhinal | 33875891 | chr4:103188709 | C=0.92, T=0.08 | 0.07 | 0.15 | 0.11-0.19 | 3.00E-15 |
| LST | rs13107325 | wg lh intensity-contrast inferiorparietal | 33875891 | chr4:103188709 | C=0.92, T=0.08 | 0.07 | 0.119 | 0.082-0.156 | 4.00E-10 |
| LST | rs13107325 | aseg lh intensity Accumbens-area | 33875891 | chr4:103188709 | C=0.92, T=0.08 | 0.07 | 0.36 | 0.32-0.4 | 1.00E-80 |
| LST | rs13107325 | aseg lh intensity VentralDC | 33875891 | chr4:103188709 | C=0.92, T=0.08 | 0.07 | 0.138 | 0.1-0.18 | 5.00E-13 |
| LST | rs13107325 | aseg rh intensity Cerebellum-Cortex | 33875891 | chr4:103188709 | C=0.92, T=0.08 | 0.07 | 0.188 | 0.15-0.23 | 4.00E-23 |
| LST | rs13107325 | aseg rh intensity Caudate | 33875891 | chr4:103188709 | C=0.92, T=0.08 | 0.07 | 0.129 | 0.092-0.166 | 1.00E-11 |
| LST | rs13107325 | aseg rh intensity Putamen | 33875891 | chr4:103188709 | C=0.92, T=0.08 | 0.07 | 0.353 | 0.32-0.39 | 1.00E-77 |
| LST | rs13107325 | wg lh intensity-contrast inferiortemporal | 33875891 | chr4:103188709 | C=0.92, T=0.08 | 0.07 | 0.114 | 0.077-0.151 | 2.00E-09 |
| LST | rs13107325 | wg lh intensity-contrast isthmuscingulate | 33875891 | chr4:103188709 | C=0.92, T=0.08 | 0.07 | 0.152 | 0.11-0.19 | 1.00E-15 |
| LST | rs13107325 | aseg rh intensity Pallidum | 33875891 | chr4:103188709 | C=0.92, T=0.08 | 0.07 | 0.287 | 0.25-0.32 | 9.00E-52 |
| LST | rs13107325 | aseg rh intensity Accumbens-area | 33875891 | chr4:103188709 | C=0.92, T=0.08 | 0.07 | 0.396 | 0.36-0.43 | 5.00E-97 |
| LST | rs13107325 | wg lh intensity-contrast caudalmiddlefrontal | 33875891 | chr4:103188709 | C=0.92, T=0.08 | 0.07 | 0.125 | 0.088-0.162 | 5.00E-11 |
| LST | rs13107325 | aparc-Desikan lh thickness precentral | 33875891 | chr4:103188709 | C=0.92, T=0.08 | 0.07 | 0.112 | 0.075-0.149 | 3.00E-09 |
| LST | rs13107325 | aparc-Desikan rh thickness GlobalMeanThickness | 33875891 | chr4:103188709 | C=0.92, T=0.08 | 0.07 | 0.126 | 0.089-0.163 | 3.00E-11 |
| LST | rs13107325 | aparc-Desikan rh thickness cuneus | 33875891 | chr4:103188709 | C=0.92, T=0.08 | 0.07 | 0.169 | 0.13-0.21 | 5.00E-19 |
| LST | rs13107325 | aparc-Desikan rh thickness lateraloccipital | 33875891 | chr4:103188709 | C=0.92, T=0.08 | 0.07 | 0.171 | 0.13-0.21 | 3.00E-19 |
| LST | rs13107325 | aparc-Desikan rh thickness lingual | 33875891 | chr4:103188709 | C=0.92, T=0.08 | 0.07 | 0.156 | 0.12-0.19 | 3.00E-16 |
| LST | rs13107325 | aparc-Desikan rh thickness paracentral | 33875891 | chr4:103188709 | C=0.92, T=0.08 | 0.07 | 0.138 | 0.1-0.18 | 3.00E-13 |
| LST | rs13107325 | aparc-Desikan rh thickness pericalcarine | 33875891 | chr4:103188709 | C=0.92, T=0.08 | 0.07 | 0.144 | 0.11-0.18 | 4.00E-14 |
| LST | rs13107325 | aparc-Desikan rh thickness transversetemporal | 33875891 | chr4:103188709 | C=0.92, T=0.08 | 0.07 | 0.127 | 0.09-0.164 | 2.00E-11 |
| LST | rs13107325 | BA-exvivo lh thickness BA4a | 33875891 | chr4:103188709 | C=0.92, T=0.08 | 0.07 | 0.144 | 0.11-0.18 | 4.00E-14 |
| LST | rs13107325 | BA-exvivo lh thickness BA4p | 33875891 | chr4:103188709 | C=0.92, T=0.08 | 0.07 | 0.124 | 0.087-0.161 | 7.00E-11 |
| LST | rs13107325 | BA-exvivo lh thickness V2 | 33875891 | chr4:103188709 | C=0.92, T=0.08 | 0.07 | 0.171 | 0.13-0.21 | 2.00E-19 |
| LST | rs13107325 | IDP dMRI TBSS OD Cerebral peduncle R | 33875891 | chr4:103188709 | C=0.92, T=0.08 | 0.07 | 0.221 | 0.18-0.26 | 1.00E-30 |
| LST | rs13107325 | IDP dMRI TBSS OD Cerebral peduncle L | 33875891 | chr4:103188709 | C=0.92, T=0.08 | 0.07 | 0.223 | 0.19-0.26 | 3.00E-31 |
| LST | rs13107325 | IDP dMRI TBSS OD Anterior limb of internal capsule R | 33875891 | chr4:103188709 | C=0.92, T=0.08 | 0.07 | 0.119 | 0.082-0.156 | 7.00E-10 |
| LST | rs13107325 | IDP dMRI TBSS OD Anterior limb of internal capsule L | 33875891 | chr4:103188709 | C=0.92, T=0.08 | 0.07 | 0.178 | 0.14-0.22 | 2.00E-20 |
| LST | rs13107325 | QC SWI-to-T1 linear alignment discrepancy | 33875891 | chr4:103188709 | C=0.92, T=0.08 | 0.07 | 0.192 | 0.15-0.23 | 3.00E-22 |
| LST | rs13107325 | wg lh intensity-contrast posteriorcingulate | 33875891 | chr4:103188709 | C=0.92, T=0.08 | 0.07 | 0.117 | 0.08-0.154 | 8.00E-10 |
| LST | rs13107325 | wg lh intensity-contrast precentral | 33875891 | chr4:103188709 | C=0.92, T=0.08 | 0.07 | 0.125 | 0.088-0.162 | 5.00E-11 |
| LST | rs13107325 | wg lh intensity-contrast precuneus | 33875891 | chr4:103188709 | C=0.92, T=0.08 | 0.07 | 0.172 | 0.13-0.21 | 1.00E-19 |
| LST | rs13107325 | wg lh intensity-contrast supramarginal | 33875891 | chr4:103188709 | C=0.92, T=0.08 | 0.07 | 0.116 | 0.079-0.153 | 1.00E-09 |
| LST | rs13107325 | wg lh intensity-contrast parahippocampal | 33875891 | chr4:103188709 | C=0.92, T=0.08 | 0.07 | 0.112 | 0.075-0.149 | 4.00E-09 |
| LST | rs13107325 | wg lh intensity-contrast paracentral | 33875891 | chr4:103188709 | C=0.92, T=0.08 | 0.07 | 0.134 | 0.097-0.171 | 2.00E-12 |
| LST | rs13107325 | wg lh intensity-contrast parsopercularis | 33875891 | chr4:103188709 | C=0.92, T=0.08 | 0.07 | 0.116 | 0.079-0.153 | 1.00E-09 |
| LST | rs13107325 | aparc-Desikan lh thickness GlobalMeanThickness | 33875891 | chr4:103188709 | C=0.92, T=0.08 | 0.07 | 0.108 | 0.071-0.145 | 1.00E-08 |
| LST | rs13107325 | aparc-Desikan lh thickness cuneus | 33875891 | chr4:103188709 | C=0.92, T=0.08 | 0.07 | 0.156 | 0.12-0.19 | 3.00E-16 |
| LST | rs13107325 | aparc-Desikan lh thickness lateraloccipital | 33875891 | chr4:103188709 | C=0.92, T=0.08 | 0.07 | 0.166 | 0.13-0.2 | 2.00E-18 |
| LST | rs13107325 | aparc-Desikan lh thickness paracentral | 33875891 | chr4:103188709 | C=0.92, T=0.08 | 0.07 | 0.124 | 0.087-0.161 | 7.00E-11 |
| LST | rs13107325 | IDP dMRI TBSS L1 Cerebral peduncle R | 33875891 | chr4:103188709 | C=0.92, T=0.08 | 0.07 | 0.137 | 0.1-0.174 | 1.00E-12 |
| LST | rs13107325 | IDP dMRI TBSS L1 Cerebral peduncle L | 33875891 | chr4:103188709 | C=0.92, T=0.08 | 0.07 | 0.138 | 0.1-0.18 | 7.00E-13 |
| LST | rs13107325 | Body mass index and systole blood pressure (pairwise) | 33619380 | chr4:103188709 | C=0.92, T=0.08 | 0.0795 | NA | NA | 2.00E-22 |
| LST | rs13107325 | wg rh intensity-contrast entorhinal | 33875891 | chr4:103188709 | C=0.92, T=0.08 | 0.07 | 0.138 | 0.1-0.18 | 3.00E-13 |
| LST | rs13107325 | wg rh intensity-contrast inferiorparietal | 33875891 | chr4:103188709 | C=0.92, T=0.08 | 0.07 | 0.137 | 0.1-0.174 | 7.00E-13 |
| LST | rs13107325 | wg rh intensity-contrast isthmuscingulate | 33875891 | chr4:103188709 | C=0.92, T=0.08 | 0.07 | 0.141 | 0.1-0.18 | 1.00E-13 |
| LST | rs13107325 | IDP T1 FAST ROIs L caudate | 33875891 | chr4:103188709 | C=0.92, T=0.08 | 0.07 | 0.13 | 0.093-0.167 | 3.00E-12 |
| LST | rs13107325 | IDP T1 FAST ROIs R caudate | 33875891 | chr4:103188709 | C=0.92, T=0.08 | 0.07 | 0.139 | 0.1-0.18 | 8.00E-14 |
| LST | rs13107325 | IDP T1 FAST ROIs L putamen | 33875891 | chr4:103188709 | C=0.92, T=0.08 | 0.07 | 0.4 | 0.36-0.44 | 2.00E-103 |
| LST | rs13107325 | IDP T1 FAST ROIs R putamen | 33875891 | chr4:103188709 | C=0.92, T=0.08 | 0.07 | 0.401 | 0.37-0.44 | 3.00E-104 |
| LST | rs13107325 | wg rh intensity-contrast parahippocampal | 33875891 | chr4:103188709 | C=0.92, T=0.08 | 0.07 | 0.138 | 0.1-0.18 | 4.00E-13 |
| LST | rs13107325 | wg rh intensity-contrast paracentral | 33875891 | chr4:103188709 | C=0.92, T=0.08 | 0.07 | 0.116 | 0.079-0.153 | 1.00E-09 |
| LST | rs13107325 | Schizophrenia | 29483656 | chr4:103188709 | C=0.92, T=0.08 | NR | 1.1737089 | 1.14-1.21 | 1.00E-16 |
| LST | rs13107325 | HAVCR2/TNFRSF1B protein level ratio | 38412862 | chr4:103188709 | C=0.92, T=0.08 | NR | 0.0990791 | 0.074-0.124 | 2.00E-14 |
| LST | rs13107325 | Brain shape (segment 20) | 33821002 | chr4:103188709 | C=0.92, T=0.08 | NR | NA | NA | 4.00E-12 |
| LST | rs13107325 | Childhood body mass index | 33045005 | chr4:103188709 | C=0.92, T=0.08 | 0.07 | 0.095 | 0.062-0.128 | 4.00E-08 |
| LST | rs13107325 | IDP dMRI TBSS MO Cerebral peduncle R | 33875891 | chr4:103188709 | C=0.92, T=0.08 | 0.07 | 0.168 | 0.13-0.21 | 3.00E-18 |
| LST | rs13107325 | IDP dMRI TBSS MO Cerebral peduncle L | 33875891 | chr4:103188709 | C=0.92, T=0.08 | 0.07 | 0.172 | 0.13-0.21 | 4.00E-19 |
| LST | rs13107325 | IDP dMRI TBSS MD Cerebral peduncle L | 33875891 | chr4:103188709 | C=0.92, T=0.08 | 0.07 | 0.125 | 0.088-0.162 | 7.00E-11 |
| LST | rs13107325 | ThalamNuclei lh volume VAmc | 33875891 | chr4:103188709 | C=0.92, T=0.08 | 0.07 | 0.165 | 0.13-0.2 | 4.00E-18 |
| LST | rs13107325 | ThalamNuclei rh volume MGN | 33875891 | chr4:103188709 | C=0.92, T=0.08 | 0.07 | 0.14 | 0.1-0.18 | 2.00E-13 |
| LST | rs13107325 | ThalamNuclei rh volume VAmc | 33875891 | chr4:103188709 | C=0.92, T=0.08 | 0.07 | 0.153 | 0.12-0.19 | 1.00E-15 |
| LST | rs13107325 | ThalamNuclei lh volume PuI | 33875891 | chr4:103188709 | C=0.92, T=0.08 | 0.07 | 0.156 | 0.12-0.19 | 3.00E-16 |
| LST | rs13107325 | wg rh intensity-contrast precuneus | 33875891 | chr4:103188709 | C=0.92, T=0.08 | 0.07 | 0.178 | 0.14-0.22 | 6.00E-21 |
| LST | rs13107325 | wg rh intensity-contrast superiorparietal | 33875891 | chr4:103188709 | C=0.92, T=0.08 | 0.07 | 0.134 | 0.097-0.171 | 2.00E-12 |
| LST | rs13107325 | wg rh intensity-contrast supramarginal | 33875891 | chr4:103188709 | C=0.92, T=0.08 | 0.07 | 0.134 | 0.097-0.171 | 2.00E-12 |
| LST | rs13107325 | Low hand grip strength (60 years and older) (EWGSOP) | 33510174 | chr4:103188709 | C=0.92, T=0.08 | 0.0739 | 0.0897 | 0.063-0.117 | 7.00E-11 |
| LST | rs13107325 | IDP SWI T2star left caudate | 33875891 | chr4:103188709 | C=0.92, T=0.08 | 0.07 | 0.216 | 0.18-0.26 | 2.00E-28 |
| LST | rs13107325 | IDP SWI T2star right caudate | 33875891 | chr4:103188709 | C=0.92, T=0.08 | 0.07 | 0.225 | 0.19-0.26 | 1.00E-30 |
| LST | rs13107325 | IDP SWI T2star left pallidum | 33875891 | chr4:103188709 | C=0.92, T=0.08 | 0.07 | 0.299 | 0.26-0.34 | 6.00E-53 |
| LST | rs13107325 | IDP SWI T2star right pallidum | 33875891 | chr4:103188709 | C=0.92, T=0.08 | 0.07 | 0.338 | 0.3-0.38 | 5.00E-67 |
| LST | rs13107325 | AmygNuclei rh volume Accessory-Basal-nucleus | 33875891 | chr4:103188709 | C=0.92, T=0.08 | 0.07 | 0.151 | 0.11-0.19 | 2.00E-15 |
| LST | rs13107325 | AmygNuclei rh volume Central-nucleus | 33875891 | chr4:103188709 | C=0.92, T=0.08 | 0.07 | 0.292 | 0.25-0.33 | 2.00E-53 |
| LST | rs13107325 | AmygNuclei rh volume Medial-nucleus | 33875891 | chr4:103188709 | C=0.92, T=0.08 | 0.07 | 0.303 | 0.27-0.34 | 1.00E-57 |
| LST | rs13107325 | AmygNuclei rh volume Cortical-nucleus | 33875891 | chr4:103188709 | C=0.92, T=0.08 | 0.07 | 0.249 | 0.21-0.29 | 2.00E-39 |
| LST | rs13107325 | AmygNuclei lh volume Accessory-Basal-nucleus | 33875891 | chr4:103188709 | C=0.92, T=0.08 | 0.07 | 0.112 | 0.075-0.149 | 4.00E-09 |
| LST | rs13107325 | AmygNuclei lh volume Central-nucleus | 33875891 | chr4:103188709 | C=0.92, T=0.08 | 0.07 | 0.218 | 0.18-0.26 | 2.00E-30 |
| LST | rs13107325 | AmygNuclei lh volume Medial-nucleus | 33875891 | chr4:103188709 | C=0.92, T=0.08 | 0.07 | 0.239 | 0.2-0.28 | 2.00E-36 |
| LST | rs13107325 | AmygNuclei lh volume Cortical-nucleus | 33875891 | chr4:103188709 | C=0.92, T=0.08 | 0.07 | 0.17 | 0.13-0.21 | 3.00E-19 |
| LST | rs13107325 | aseg rh volume Pallidum | 33875891 | chr4:103188709 | C=0.92, T=0.08 | 0.07 | 0.167 | 0.13-0.2 | 1.00E-18 |
| LST | rs13107325 | Left-handedness | 32989287 | chr4:103188709 | C=0.92, T=0.08 | 0.0788 | 1.0570797 | NA | 5.00E-14 |
| LST | rs13107325 | Alcohol use disorder (consumption score) | 30336701 | chr4:103188709 | C=0.92, T=0.08 | NR | 0.01 | 0.0061-0.0139 | 3.00E-10 |
| LST | rs13107325 | Sleep duration (short sleep) | 30846698 | chr4:103188709 | C=0.92, T=0.08 | NR | 1.078 | NA | 3.00E-13 |
| LST | rs13107325 | Cognitive aspects of educational attainment | 33414549 | chr4:103188709 | C=0.92, T=0.08 | 0.0795229 | 0.11577766 | 0.093-0.139 | 6.00E-23 |
| LST | rs13107325 | OMD/SPP1 protein level ratio | 38412862 | chr4:103188709 | C=0.92, T=0.08 | NR | 0.103862 | 0.078-0.129 | 2.00E-15 |
| LST | rs13107325 | CD300C/LRRC25 protein level ratio | 38412862 | chr4:103188709 | C=0.92, T=0.08 | NR | 0.270571 | 0.25-0.3 | 2.00E-95 |
| LST | rs13107325 | FOLR2/THY1 protein level ratio | 38412862 | chr4:103188709 | C=0.92, T=0.08 | NR | 0.115753 | 0.09-0.141 | 8.00E-19 |
| LST | rs13107325 | CD300C/FOLR2 protein level ratio | 38412862 | chr4:103188709 | C=0.92, T=0.08 | NR | 0.21354 | 0.19-0.24 | 1.00E-62 |
| LST | rs13107325 | CD300C/GOLM2 protein level ratio | 38412862 | chr4:103188709 | C=0.92, T=0.08 | NR | 0.218329 | 0.19-0.24 | 6.00E-63 |
| LST | rs13107325 | CD300C/HAVCR2 protein level ratio | 38412862 | chr4:103188709 | C=0.92, T=0.08 | NR | 0.204803 | 0.18-0.23 | 1.00E-55 |
| LST | rs13107325 | ANPEP/NT5E protein level ratio | 38412862 | chr4:103188709 | C=0.92, T=0.08 | NR | 0.133441 | 0.11-0.16 | 4.00E-24 |
| LST | rs13107325 | Alcohol use disorder (consumption score) | 33861876 | chr4:103188709 | C=0.92, T=0.08 | NR | 0.153 | 0.11-0.19 | 4.00E-14 |
| LST | rs13107325 | RGMA/RGMB protein level ratio | 38412862 | chr4:103188709 | C=0.92, T=0.08 | NR | 0.16294 | 0.14-0.19 | 3.00E-36 |
| LST | rs13107325 | Calcium levels | 33887147 | chr4:103188709 | C=0.92, T=0.08 | 0.0758 | 0.006227 | 0.0054-0.0071/ | 3.00E-48 |
| LST | rs13107325 | Urate levels | 33356394 | chr4:103188709 | C=0.92, T=0.08 | 0.07 | 0.0297 | 0.023-0.037 | 3.00E-17 |
| LST | rs13107325 | Edge-level brain connectivity (multivariate analysis) | 36800424 | chr4:103188709 | C=0.92, T=0.08 | 0.929925 | 8.738254 | NA | 2.00E-18 |
| LST | rs13107325 | Osteoarthritis | 34450027 | chr4:103188709 | C=0.92, T=0.08 | 0.0714 | 1.081447 | 1.061913357-1.10134005 | 3.00E-17 |
| LST | rs13107325 | Node-level brain connectivity (multivariate analysis) | 36800424 | chr4:103188709 | C=0.92, T=0.08 | 0.929925 | 5.7687464 | NA | 8.00E-09 |
| LST | rs13107325 | Triglycerides to total lipids ratio in very small VLDL | 35213538 | chr4:103188709 | C=0.92, T=0.08 | 0.925718 | 0.0470502 | 0.032-0.062 | 3.00E-10 |
| LST | rs13107325 | Glycine levels | 35213538 | chr4:103188709 | C=0.92, T=0.08 | 0.92569 | 0.0493389 | 0.035-0.064 | 3.00E-11 |
| LST | rs13107325 | Cholesteryl ester levels in chylomicrons and extremely large VLDL | 35213538 | chr4:103188709 | C=0.92, T=0.08 | 0.925718 | 0.0415676 | 0.027-0.056 | 5.00E-08 |
| LST | rs13107325 | Cholesterol levels in medium HDL | 35213538 | chr4:103188709 | C=0.92, T=0.08 | 0.925718 | 0.0710526 | 0.057-0.085 | 1.00E-22 |
| LST | rs13107325 | HDL cholesterol levels | 35213538 | chr4:103188709 | C=0.92, T=0.08 | 0.925718 | 0.0745959 | 0.061-0.088 | 3.00E-26 |
| LST | rs13107325 | Total esterified cholesterol levels | 35213538 | chr4:103188709 | C=0.92, T=0.08 | 0.925718 | 0.0414386 | 0.027-0.056 | 5.00E-08 |
| LST | rs13107325 | Free cholesterol to total lipids ratio in very large HDL | 35213538 | chr4:103188709 | C=0.92, T=0.08 | 0.925714 | 0.0581901 | 0.045-0.072 | 7.00E-17 |
| LST | rs13107325 | Cholesteryl esters to total lipids ratio in very small VLDL | 35213538 | chr4:103188709 | C=0.92, T=0.08 | 0.925718 | 0.0572357 | 0.043-0.072 | 9.00E-15 |
| LST | rs13107325 | Concentration of medium HDL particles | 35213538 | chr4:103188709 | C=0.92, T=0.08 | 0.925718 | 0.0682079 | 0.054-0.082 | 5.00E-21 |
| LST | rs13107325 | Cholesteryl ester levels in very large HDL | 35213538 | chr4:103188709 | C=0.92, T=0.08 | 0.925718 | 0.0563786 | 0.043-0.07 | 1.00E-15 |
| LST | rs13107325 | Whole brain restricted isotropic diffusion (multivariate analysis) | 35505052 | chr4:103188709 | C=0.92, T=0.08 | NR | NA | NA | 6.00E-125 |
| LST | rs13107325 | Triglycerides to total lipids ratio in very large HDL | 35213538 | chr4:103188709 | C=0.92, T=0.08 | 0.925714 | 0.0438462 | 0.029-0.058 | 3.00E-09 |
| LST | rs13107325 | Whole brain free water diffusion (multivariate analysis) | 35505052 | chr4:103188709 | C=0.92, T=0.08 | NR | NA | NA | 4.00E-45 |
| LST | rs13107325 | Phosphatidylcholine levels | 35213538 | chr4:103188709 | C=0.92, T=0.08 | 0.925726 | 0.0485053 | 0.034-0.063 | 4.00E-11 |
| LST | rs13107325 | Triglycerides to total lipids ratio in small HDL | 35213538 | chr4:103188709 | C=0.92, T=0.08 | 0.925718 | 0.0571594 | 0.043-0.072 | 2.00E-14 |
| LST | rs13107325 | Concentration of very large HDL particles | 35213538 | chr4:103188709 | C=0.92, T=0.08 | 0.925718 | 0.0536293 | 0.04-0.068 | 4.00E-14 |
| LST | rs13107325 | Cholesterol to total lipids ratio in very small VLDL | 35213538 | chr4:103188709 | C=0.92, T=0.08 | 0.925718 | 0.054294 | 0.04-0.069 | 3.00E-13 |
| LST | rs13107325 | Ratio of triglycerides to phosphoglycerides | 35213538 | chr4:103188709 | C=0.92, T=0.08 | 0.925726 | 0.053297 | 0.039-0.068 | 3.00E-13 |
| LST | rs13107325 | Sphingomyelin levels | 35213538 | chr4:103188709 | C=0.92, T=0.08 | 0.925726 | 0.05971 | 0.045-0.074 | 5.00E-16 |
| LST | rs13107325 | Free cholesterol levels in very large HDL | 35213538 | chr4:103188709 | C=0.92, T=0.08 | 0.925718 | 0.0425959 | 0.028-0.057 | 7.00E-09 |
| LST | rs13107325 | Phosphoglycerides levels | 35213538 | chr4:103188709 | C=0.92, T=0.08 | 0.925726 | 0.0457592 | 0.031-0.06 | 7.00E-10 |
| LST | rs13107325 | Total lipid levels in very large HDL | 35213538 | chr4:103188709 | C=0.92, T=0.08 | 0.925718 | 0.052796 | 0.039-0.067 | 9.00E-14 |
| LST | rs13107325 | Whole brain restricted directional diffusion (multivariate analysis) | 35505052 | chr4:103188709 | C=0.92, T=0.08 | NR | NA | NA | 7.00E-74 |
| LST | rs13107325 | Ratio of monounsaturated fatty acids to total fatty acids | 35213538 | chr4:103188709 | C=0.92, T=0.08 | 0.925726 | 0.0432974 | 0.029-0.058 | 9.00E-09 |
| LST | rs13107325 | Total phospholipid levels in lipoprotein particles | 35213538 | chr4:103188709 | C=0.92, T=0.08 | 0.925718 | 0.0442121 | 0.03-0.059 | 4.00E-09 |
| LST | rs13107325 | Total concentration of lipoprotein particles | 35213538 | chr4:103188709 | C=0.92, T=0.08 | 0.925718 | 0.063093 | 0.049-0.078 | 2.00E-17 |
| LST | rs13107325 | Cholesterol levels in very large HDL | 35213538 | chr4:103188709 | C=0.92, T=0.08 | 0.925718 | 0.053841 | 0.04-0.068 | 4.00E-14 |
| LST | rs13107325 | Phospholipid levels in very large HDL | 35213538 | chr4:103188709 | C=0.92, T=0.08 | 0.925718 | 0.0522766 | 0.038-0.066 | 2.00E-13 |
| LST | rs13107325 | Phospholipids to total lipids ratio in very small VLDL | 35213538 | chr4:103188709 | C=0.92, T=0.08 | 0.925718 | 0.0619694 | 0.047-0.077 | 7.00E-16 |
| LST | rs13107325 | Body mass index | 34594039 | chr4:103188709 | C=0.92, T=0.08 | NR | 0.0493 | 0.041-0.058 | 1.00E-28 |
| LST | rs13107325 | Serum albumin levels | 34594039 | chr4:103188709 | C=0.92, T=0.08 | NR | 0.0559 | 0.047-0.065 | 8.00E-33 |
| LST | rs13107325 | Aspartate aminotransferase levels | 34594039 | chr4:103188709 | C=0.92, T=0.08 | NR | 0.0593 | 0.051-0.068 | 3.00E-42 |
| LST | rs13107325 | Calcium levels | 34594039 | chr4:103188709 | C=0.92, T=0.08 | NR | 0.064 | 0.055-0.073 | 5.00E-42 |
| LST | rs13107325 | Estimated glomerular filtration rate (creatinine) | 35710981 | chr4:103188709 | C=0.92, T=0.08 | 0.0722 | 5.742 | NA | 9.00E-09 |
| LST | rs13107325 | HDL cholesterol | 34594039 | chr4:103188709 | C=0.92, T=0.08 | NR | 0.082 | 0.074-0.09 | 6.00E-80 |
| LST | rs13107325 | Diastolic blood pressure | 34594039 | chr4:103188709 | C=0.92, T=0.08 | NR | 0.0432 | 0.034-0.052 | 1.00E-21 |
| LST | rs13107325 | Gamma glutamyl transpeptidase | 34594039 | chr4:103188709 | C=0.92, T=0.08 | NR | 0.0283 | 0.02-0.037 | 2.00E-11 |
| LST | rs13107325 | Vertex-wise sulcal depth | 34910505 | chr4:103188709 | C=0.92, T=0.08 | 0.08 | 15.53 | NA | 2.00E-54 |
| LST | rs13107325 | Hemoglobin | 34594039 | chr4:103188709 | C=0.92, T=0.08 | NR | 0.0373 | 0.03-0.044 | 4.00E-25 |
| LST | rs13107325 | High density lipoprotein cholesterol levels | 33339817 | chr4:103188709 | C=0.92, T=0.08 | NR | 0.0865 | 0.059-0.114 | 4.00E-10 |
| LST | rs13107325 | Hand grip strength | 29691431 | chr4:103188709 | C=0.92, T=0.08 | 0.075 | 0.006 | 0.0048-0.0072 | 4.00E-23 |
| LST | rs13107325 | Brain imaging measurements | 30305740 | chr4:103188709 | C=0.92, T=0.08 | 0.073861 | 0.37008 | 0.31-0.43 | 7.00E-39 |
| LST | rs13107325 | Brain imaging measurements | 30305740 | chr4:103188709 | C=0.92, T=0.08 | 0.073861 | 0.36694 | 0.31-0.42 | 2.00E-39 |
| LST | rs13107325 | Brain imaging measurements | 30305740 | chr4:103188709 | C=0.92, T=0.08 | 0.073861 | 0.33842 | 0.29-0.39 | 1.00E-42 |
| LST | rs13107325 | Brain imaging measurements | 30305740 | chr4:103188709 | C=0.92, T=0.08 | 0.073861 | 0.33352 | 0.29-0.38 | 2.00E-41 |
| LST | rs13107325 | Walking pace | 33128006 | chr4:103188709 | C=0.92, T=0.08 | NR | 0.024021 | 0.019-0.029 | 6.00E-24 |
| LST | rs13107325 | Adult body size | 32376654 | chr4:103188709 | C=0.92, T=0.08 | 0.92511 | 0.0287138 | 0.024-0.034 | 3.00E-28 |
| LST | rs13107325 | Alcohol use disorder (consumption score) | 30940813 | chr4:103188709 | C=0.92, T=0.08 | 0.921 | 0.1034 | 0.084-0.123 | 1.00E-25 |
| LST | rs13107325 | Alcohol use disorder (consumption score) | 30940813 | chr4:103188709 | C=0.92, T=0.08 | 0.937 | 11.45 | NA | 2.00E-30 |
| LST | rs13107325 | Alcohol use disorder | 30940813 | chr4:103188709 | C=0.92, T=0.08 | 0.937 | 7.6 | NA | 3.00E-14 |
| LST | rs13107325 | Alcohol use disorder | 30940813 | chr4:103188709 | C=0.92, T=0.08 | 0.921 | 1.1325 | 1.1-1.16 | 3.00E-14 |
| LST | rs13107325 | Body mass index | 29273807 | chr4:103188709 | C=0.92, T=0.08 | 0.06 | 0.0495 | 0.042-0.057 | 5.00E-40 |
| LST | rs13107325 | Body mass index | 30108127 | chr4:103188709 | C=0.92, T=0.08 | NR | 0.051 | NA | 3.00E-21 |
| LST | rs13107325 | HDL cholesterol | 30275531 | chr4:103188709 | C=0.92, T=0.08 | 0.0765 | 0.0798 | 0.069-0.09-1 | 2.00E-50 |
| LST | rs13107325 | Alcohol consumption (drinks per week) | 30643251 | chr4:103188709 | C=0.92, T=0.08 | 0.0722 | 0.027504515 | 0.022-0.033 | 2.00E-22 |
| LST | rs13107325 | Shoulder impingement or rotator cuff tear | 33482370 | chr4:103188709 | C=0.92, T=0.08 | 0.0745 | 1.1557 | NA | 4.00E-08 |
| LST | rs13107325 | Educational attainment (years of education) | 30038396 | chr4:103188709 | C=0.92, T=0.08 | 0.0721 | 0.0239 | 0.019-0.029 | 2.00E-18 |
| LST | rs13107325 | Voxel-wise structural brain imaging measurements | 30649180 | chr4:103188709 | C=0.92, T=0.08 | NR | NA | NA | 2.00E-11 |
| LST | rs13107325 | Voxel-wise structural brain imaging measurements | 30649180 | chr4:103188709 | C=0.92, T=0.08 | NR | NA | NA | 9.00E-18 |
| LST | rs13107325 | Osteoarthritis | 30664745 | chr4:103188709 | C=0.92, T=0.08 | 0.08 | 1.1 | 1.07-1.12 | 8.00E-19 |
| LST | rs13107325 | Alcohol consumption (drinks per week) (MTAG) | 30643251 | chr4:103188709 | C=0.92, T=0.08 | 0.0722 | 0.0256461 | 0.021-0.031 | 1.00E-23 |
| LST | rs13107325 | Metabolic biomarkers (multivariate analysis) | 33980691 | chr4:103188709 | C=0.92, T=0.08 | 0.07 | NA | NA | 5.00E-32 |
| LST | rs13107325 | Aspartate aminotransferase levels | 33980691 | chr4:103188709 | C=0.92, T=0.08 | 0.07 | 0.058 | / | 3.00E-54 |
| LST | rs13107325 | Triglycerides | 33980691 | chr4:103188709 | C=0.92, T=0.08 | 0.07 | 0.029 | / | 2.00E-14 |
| LST | rs13107325 | Alanine aminotransferase levels | 33980691 | chr4:103188709 | C=0.92, T=0.08 | 0.07 | 0.022 | / | 8.00E-10 |
| LST | rs13107325 | High density lipoprotein cholesterol levels | 33980691 | chr4:103188709 | C=0.92, T=0.08 | 0.07 | 0.078 | / | 5.00E-118 |
| LST | rs13107325 | A body shape index | 34021172 | chr4:103188709 | C=0.92, T=0.08 | NR | 0.0366395 | 0.026-0.047 | 2.00E-11 |
| LST | rs13107325 | Waist-to-hip ratio adjusted for BMI | 34021172 | chr4:103188709 | C=0.92, T=0.08 | NR | 0.0387088 | 0.028-0.049 | 8.00E-13 |
| LST | rs13107325 | Decaffeinated coffee consumption and/or lifetime cannabis use | 35898629 | chr4:103188709 | C=0.92, T=0.08 | NR | NA | NA | 1.00E-19 |
| LST | rs13107325 | Decaffeinated coffee consumption and/or neuroticism | 35898629 | chr4:103188709 | C=0.92, T=0.08 | NR | NA | NA | 2.00E-16 |
| LST | rs13107325 | Decaffeinated coffee consumption and/or alcohol use disorder score | 35898629 | chr4:103188709 | C=0.92, T=0.08 | NR | NA | NA | 4.00E-23 |
| LST | rs13107325 | Decaffeinated coffee consumption and/or alcohol consumption score | 35898629 | chr4:103188709 | C=0.92, T=0.08 | NR | NA | NA | 7.00E-22 |
| LST | rs13107325 | Decaffeinated coffee consumption and/or alcohol dependence and hazardous-use score | 35898629 | chr4:103188709 | C=0.92, T=0.08 | NR | NA | NA | 3.00E-24 |
| LST | rs13107325 | Decaffeinated coffee consumption or major depression disorder | 35898629 | chr4:103188709 | C=0.92, T=0.08 | NR | NA | NA | 2.00E-16 |
| LST | rs13107325 | Decaffeinated coffee consumption and/or insomnia | 35898629 | chr4:103188709 | C=0.92, T=0.08 | NR | NA | NA | 2.00E-16 |
| LST | rs13107325 | Cholesteryl ester levels in HDL | 35213538 | chr4:103188709 | C=0.92, T=0.08 | 0.925718 | 0.0746876 | 0.061-0.089 | 4.00E-26 |
| LST | rs13107325 | Apolipoprotein A1 levels | 35213538 | chr4:103188709 | C=0.92, T=0.08 | 0.925718 | 0.0692251 | 0.055-0.083 | 7.00E-22 |
| LST | rs13107325 | Total cholines levels | 35213538 | chr4:103188709 | C=0.92, T=0.08 | 0.925726 | 0.0498448 | 0.035-0.064 | 2.00E-11 |
| LST | rs13107325 | Triglyceride to HDL cholesterol ratio | 38200128 | chr4:103188709 | C=0.92, T=0.08 | 0.0749 | 0.0703 | 0.061-0.08 | 5.00E-32 |
| LST | rs13107325 | Cholesterol to total lipids ratio in medium HDL | 35213538 | chr4:103188709 | C=0.92, T=0.08 | 0.925718 | 0.0561251 | 0.041-0.071 | 6.00E-14 |
| LST | rs13107325 | Waist-hip index | 34021172 | chr4:103188709 | C=0.92, T=0.08 | NR | 0.0411267 | 0.031-0.052 | 3.00E-14 |
| LST | rs13107325 | Cholesteryl ester levels in medium HDL | 35213538 | chr4:103188709 | C=0.92, T=0.08 | 0.925718 | 0.0708212 | 0.057-0.085 | 2.00E-22 |
| LST | rs13107325 | Cholesteryl esters to total lipids ratio in medium HDL | 35213538 | chr4:103188709 | C=0.92, T=0.08 | 0.925718 | 0.0447636 | 0.03-0.06 | 4.00E-09 |
| LST | rs13107325 | Free cholesterol levels in medium HDL | 35213538 | chr4:103188709 | C=0.92, T=0.08 | 0.925718 | 0.0703087 | 0.056-0.084 | 9.00E-23 |
| LST | rs13107325 | Free cholesterol to total lipids ratio in medium HDL | 35213538 | chr4:103188709 | C=0.92, T=0.08 | 0.925718 | 0.0716693 | 0.058-0.085 | 9.00E-25 |
| LST | rs13107325 | Triglyceride to HDL cholesterol ratio | 38200128 | chr4:103188709 | C=0.92, T=0.08 | 0.076 | 0.0628 | 0.05-0.076 | 2.00E-18 |
| LST | rs13107325 | Free cholesterol levels in HDL | 35213538 | chr4:103188709 | C=0.92, T=0.08 | 0.925718 | 0.0715063 | 0.058-0.085 | 1.00E-24 |
| LST | rs13107325 | Triglycerides to total lipids ratio in IDL | 35213538 | chr4:103188709 | C=0.92, T=0.08 | 0.925718 | 0.0586839 | 0.044-0.073 | 8.00E-15 |
| LST | rs13107325 | Cholesteryl esters to total lipids ratio in large HDL | 35213538 | chr4:103188709 | C=0.92, T=0.08 | 0.925718 | 0.0435269 | 0.029-0.058 | 6.00E-09 |
| LST | rs13107325 | Anterior amygdaloid area volume | 37391117 | chr4:103188709 | C=0.92, T=0.08 | NR | 1.773 | 1.51-2.03 | 5.00E-41 |
| LST | rs13107325 | Cortical nucleus volume | 37391117 | chr4:103188709 | C=0.92, T=0.08 | NR | 0.6444 | 0.5-0.79 | 2.00E-18 |
| LST | rs13107325 | Lateral nucleus volume | 37391117 | chr4:103188709 | C=0.92, T=0.08 | NR | 5.968 | 4.62-7.32 | 4.00E-18 |
| LST | rs13107325 | Central nucleus volume | 37391117 | chr4:103188709 | C=0.92, T=0.08 | NR | 2.925 | 2.63-3.22 | 1.00E-84 |
| LST | rs13107325 | Cholesterol to total lipids ratio in large LDL | 35213538 | chr4:103188709 | C=0.92, T=0.08 | 0.925718 | 0.0532963 | 0.038-0.068 | 3.00E-12 |
| LST | rs13107325 | Triglycerides to total lipids ratio in large LDL | 35213538 | chr4:103188709 | C=0.92, T=0.08 | 0.925718 | 0.0535091 | 0.039-0.068 | 3.00E-12 |
| LST | rs13107325 | Total lipid levels in medium HDL | 35213538 | chr4:103188709 | C=0.92, T=0.08 | 0.925718 | 0.0652315 | 0.051-0.08 | 4.00E-19 |
| LST | rs13107325 | Concentration of HDL particles | 35213538 | chr4:103188709 | C=0.92, T=0.08 | 0.925718 | 0.066025 | 0.052-0.081 | 4.00E-19 |
| LST | rs13107325 | Average diameter for HDL particles | 35213538 | chr4:103188709 | C=0.92, T=0.08 | 0.925718 | 0.0621989 | 0.049-0.076 | 4.00E-19 |
| LST | rs13107325 | Cholesterol levels in large HDL | 35213538 | chr4:103188709 | C=0.92, T=0.08 | 0.925718 | 0.0671483 | 0.054-0.081 | 4.00E-22 |
| LST | rs13107325 | Free cholesterol levels in large HDL | 35213538 | chr4:103188709 | C=0.92, T=0.08 | 0.925718 | 0.0665832 | 0.053-0.08 | 5.00E-22 |
| LST | rs13107325 | Total lipid levels in large HDL | 35213538 | chr4:103188709 | C=0.92, T=0.08 | 0.925718 | 0.0689495 | 0.055-0.082 | 2.00E-23 |
| LST | rs13107325 | Basal nucleus volume | 37391117 | chr4:103188709 | C=0.92, T=0.08 | NR | 2.668 | 1.81-3.53 | 1.00E-09 |
| LST | rs13107325 | Lateral nucleus volume | 37391117 | chr4:103188709 | C=0.92, T=0.08 | NR | 5.993 | 4.73-7.26 | 2.00E-20 |
| LST | rs13107325 | Cortical nucleus volume | 37391117 | chr4:103188709 | C=0.92, T=0.08 | NR | 0.6511 | 0.52-0.79 | 3.00E-21 |
| LST | rs13107325 | Medial nucleus volume | 37391117 | chr4:103188709 | C=0.92, T=0.08 | NR | 1.204 | 1-1.41 | 1.00E-29 |
| LST | rs13107325 | Accessory basal nucleus volume | 37391117 | chr4:103188709 | C=0.92, T=0.08 | NR | 3.54 | 2.92-4.16 | 4.00E-29 |
| LST | rs13107325 | Free cholesterol to total lipids ratio in large LDL | 35213538 | chr4:103188709 | C=0.92, T=0.08 | 0.925718 | 0.0520563 | 0.037-0.067 | 3.00E-12 |
| LST | rs13107325 | Total lipid levels in HDL | 35213538 | chr4:103188709 | C=0.92, T=0.08 | 0.925718 | 0.071311 | 0.057-0.085 | 6.00E-24 |
| LST | rs13107325 | Phospholipid levels in HDL | 35213538 | chr4:103188709 | C=0.92, T=0.08 | 0.925718 | 0.0687325 | 0.055-0.083 | 4.00E-22 |
| LST | rs13107325 | Cholesterol to total lipids ratio in large HDL | 35213538 | chr4:103188709 | C=0.92, T=0.08 | 0.925718 | 0.0468314 | 0.032-0.061 | 2.00E-10 |
| LST | rs13107325 | Cholesteryl ester levels in large HDL | 35213538 | chr4:103188709 | C=0.92, T=0.08 | 0.925718 | 0.0670132 | 0.053-0.081 | 6.00E-22 |
| LST | rs13107325 | Phospholipid levels in large HDL | 35213538 | chr4:103188709 | C=0.92, T=0.08 | 0.925718 | 0.0694866 | 0.056-0.083 | 1.00E-23 |
| LST | rs13107325 | Concentration of large HDL particles | 35213538 | chr4:103188709 | C=0.92, T=0.08 | 0.925718 | 0.0688612 | 0.055-0.082 | 2.00E-23 |
| LST | rs13107325 | Anterior amygdaloid area volume | 37391117 | chr4:103188709 | C=0.92, T=0.08 | NR | 1.726 | 1.48-1.97 | 8.00E-44 |
| LST | rs13107325 | Central nucleus volume | 37391117 | chr4:103188709 | C=0.92, T=0.08 | NR | 2.972 | 2.7-3.25 | 7.00E-99 |
| LST | rs13107325 | Accessory basal nucleus volume | 37391117 | chr4:103188709 | C=0.92, T=0.08 | NR | 3.538 | 2.88-4.2 | 8.00E-26 |
| LST | rs13107325 | Phospholipid levels in medium HDL | 35213538 | chr4:103188709 | C=0.92, T=0.08 | 0.925718 | 0.0610888 | 0.047-0.075 | 9.00E-17 |
| LST | rs13107325 | Phospholipids to total lipids ratio in medium HDL | 35213538 | chr4:103188709 | C=0.92, T=0.08 | 0.925718 | 0.059838 | 0.045-0.074 | 1.00E-15 |
| LST | rs13107325 | Triglycerides to total lipids ratio in medium HDL | 35213538 | chr4:103188709 | C=0.92, T=0.08 | 0.925718 | 0.0499628 | 0.035-0.065 | 3.00E-11 |
| LST | rs13107325 | Triglyceride to HDL cholesterol ratio | 38200128 | chr4:103188709 | C=0.92, T=0.08 | 0.0739 | 0.0595 | 0.048-0.071 | 9.00E-18 |
| LST | rs13107325 | Cholesterol to total lipids ratio in medium LDL | 35213538 | chr4:103188709 | C=0.92, T=0.08 | 0.925718 | 0.0459371 | 0.03-0.061 | 6.00E-09 |
| LST | rs13107325 | Triglycerides to total lipids ratio in medium LDL | 35213538 | chr4:103188709 | C=0.92, T=0.08 | 0.925718 | 0.0432072 | 0.028-0.058 | 2.00E-08 |
| LST | rs13107325 | Mean arterial pressure x alcohol consumption interaction (2df test) | 29912962 | chr4:103188709 | C=0.92, T=0.08 | 0.07 | NA | NA | 4.00E-11 |
| LST | rs13107325 | Body fat percentage | 30593698 | chr4:103188709 | C=0.92, T=0.08 | NR | 0.25064 | 0.17-0.33 | 5.00E-10 |
| LST | rs13107325 | Height | 30595370 | chr4:103188709 | C=0.92, T=0.08 | NR | NA | NA | 1.00E-21 |
| LST | rs13107325 | Male-pattern baldness | 30573740 | chr4:103188709 | C=0.92, T=0.08 | 0.0758327 | 0.0535076 | 0.042-0.065 | 4.00E-20 |
| LST | rs13107325 | Body fat percentage | 30593698 | chr4:103188709 | C=0.92, T=0.08 | NR | 0.30825 | 0.21-0.4 | 4.00E-10 |
| LST | rs13107325 | Alcohol consumption (drinks per week) | 30643258 | chr4:103188709 | C=0.92, T=0.08 | 0.0744 | 0.04357754 | 0.035-0.052 | 1.00E-24 |
| LST | rs13107325 | Systolic blood pressure | 30578418 | chr4:103188709 | C=0.92, T=0.08 | 0.0752 | 0.5532 | 0.43-0.68 | 1.00E-17 |
| LST | rs13107325 | Diastolic blood pressure x alcohol consumption interaction (2df test) | 29912962 | chr4:103188709 | C=0.92, T=0.08 | 0.07 | NA | NA | 2.00E-23 |
| LST | rs13107325 | Diastolic blood pressure x alcohol consumption interaction (2df test) | 29912962 | chr4:103188709 | C=0.92, T=0.08 | 0.07 | NA | NA | 4.00E-22 |
| LST | rs13107325 | Metabolic syndrome | 31589552 | chr4:103188709 | C=0.92, T=0.08 | 0.0748556 | 0.0760904 | 0.052-0.1 | 4.00E-10 |
| LST | rs13107325 | Diastolic blood pressure | 30578418 | chr4:103188709 | C=0.92, T=0.08 | 0.3032 | 0.322 | 0.23-0.41 | 8.00E-12 |
| LST | rs13107325 | Risk-taking tendency (4-domain principal component model) | 30643258 | chr4:103188709 | C=0.92, T=0.08 | 0.0734 | 0.035913233 | 0.026-0.046 | 4.00E-13 |
| LST | rs13107325 | Brain region volumes | 31676860 | chr4:103188709 | C=0.92, T=0.08 | NR | NA | NA | 7.00E-14 |
| LST | rs13107325 | HDL cholesterol levels | 30926973 | chr4:103188709 | C=0.92, T=0.08 | 0.0723 | 0.0123 | 0.0072-0.0174 | 2.00E-06 |
| LST | rs13107325 | Vertex-wise cortical thickness | 34910505 | chr4:103188709 | C=0.92, T=0.08 | 0.08 | 28.2 | NA | 6.00E-175 |
| LST | rs13107325 | Total cholesterol levels | 34594039 | chr4:103188709 | C=0.92, T=0.08 | NR | 0.0418 | 0.033-0.05 | 3.00E-21 |
| LST | rs13107325 | Serum uric acid levels | 34594039 | chr4:103188709 | C=0.92, T=0.08 | NR | 0.031 | 0.023-0.039 | 1.00E-15 |
| LST | rs13107325 | Vertex-wise cortical surface area | 34910505 | chr4:103188709 | C=0.92, T=0.08 | 0.08 | 10.21 | NA | 2.00E-24 |
| LST | rs13107325 | High density lipoprotein cholesterol levels | 34887591 | chr4:103188709 | C=0.92, T=0.08 | 0.0638957 | 0.0827466 | 0.077-0.088 | 3.00E-115 |
| LST | rs13107325 | Cortical surface area | 34560273 | chr4:103188709 | C=0.92, T=0.08 | 0.0706 | NA | NA | 1.00E-17 |
| LST | rs13107325 | Cortical thickness | 34560273 | chr4:103188709 | C=0.92, T=0.08 | 0.0706 | NA | NA | 1.00E-17 |
| LST | rs13107325 | Liver enzyme levels (gamma-glutamyl transferase) | 33972514 | chr4:103188709 | C=0.92, T=0.08 | 0.925031 | 0.00710285 | 0.0052-0.009 | 8.00E-14 |
| LST | rs13107325 | Gastroesophageal reflux disease | 34187846 | chr4:103188709 | C=0.92, T=0.08 | 0.93 | 0.07 | NA | 2.00E-14 |
| LST | rs13107325 | Lateral thalamic nuclei volume | 34006833 | chr4:103188709 | C=0.92, T=0.08 | 0.0759 | 0.113293 | 0.082-0.145 | 1.00E-12 |
| LST | rs13107325 | Anterior thalamic nuclei volume | 34006833 | chr4:103188709 | C=0.92, T=0.08 | 0.0759 | 0.113983 | 0.083-0.145 | 9.00E-13 |
| LST | rs13107325 | Body fat percentage | 33980691 | chr4:103188709 | C=0.92, T=0.08 | 0.07 | 0.031 | % | 3.00E-28 |
| LST | rs13107325 | Red blood cell count | 34594039 | chr4:103188709 | C=0.92, T=0.08 | NR | 0.0349 | 0.027-0.043 | 2.00E-19 |
| LST | rs13107325 | Systolic blood pressure | 34594039 | chr4:103188709 | C=0.92, T=0.08 | NR | 0.0284 | 0.02-0.037 | 3.00E-11 |
| LST | rs13107325 | Urine argininate* levels in chronic kidney disease | 37277652 | chr4:103188709 | C=0.92, T=0.08 | 0.08 | 0.183 | 0.13-0.23 | 3.00E-12 |
| LST | rs13107325 | Plasma argininate* levels in chronic kidney disease | 37277652 | chr4:103188709 | C=0.92, T=0.08 | 0.08 | 0.184 | 0.13-0.24 | 2.00E-11 |
| LST | rs13107325 | Hematocrit | 34594039 | chr4:103188709 | C=0.92, T=0.08 | NR | 0.0375 | 0.03-0.045 | 4.00E-24 |
| LST | rs13107325 | Mean arterial pressure | 34594039 | chr4:103188709 | C=0.92, T=0.08 | NR | 0.0363 | 0.028-0.045 | 7.00E-17 |
| LST | rs13107325 | Vegetables liking | 35585065 | chr4:103188709 | C=0.92, T=0.08 | 0.0726 | 0.037435 | 0.024-0.051 | 5.00E-08 |
| LST | rs13107325 | Medication use (agents acting on the renin-angiotensin system) | 34594039 | chr4:103188709 | C=0.92, T=0.08 | NR | 0.0716 | 0.049-0.094 | 4.00E-10 |
| LST | rs13107325 | Brussel sprout liking | 35585065 | chr4:103188709 | C=0.92, T=0.08 | 0.0726 | 0.041452 | 0.028-0.055 | 1.00E-09 |
| LST | rs13107325 | Cognitive performance | 30038396 | chr4:103188709 | C=0.92, T=0.08 | 0.075 | 0.0543 | 0.044-0.065 | 1.00E-23 |
| LST | rs13107325 | Cognitive performance (MTAG) | 30038396 | chr4:103188709 | C=0.92, T=0.08 | 0.075 | 0.038 | 0.029-0.047 | 3.00E-18 |
| LST | rs13107325 | Self-reported math ability | 30038396 | chr4:103188709 | C=0.92, T=0.08 | 0.9166 | 0.0219 | 0.015-0.029 | 5.00E-10 |
| LST | rs13107325 | Educational attainment (MTAG) | 30038396 | chr4:103188709 | C=0.92, T=0.08 | 0.0721 | 0.0259 | 0.021-0.031 | 2.00E-24 |
| LST | rs13107325 | Medication use (agents acting on the renin-angiotensin system) | 31015401 | chr4:103188709 | C=0.92, T=0.08 | 0.0745797 | 0.071593836 | 0.049-0.094 | 4.00E-10 |
| LST | rs13107325 | Height | 34594039 | chr4:103188709 | C=0.92, T=0.08 | NR | 0.0266 | 0.021-0.033 | 2.00E-17 |
| LST | rs13107325 | Low density lipoprotein cholesterol levels | 34887591 | chr4:103188709 | C=0.92, T=0.08 | 0.0642707 | 0.0251333 | 0.02-0.031 | 7.00E-15 |
| LST | rs13107325 | Brain morphology (MOSTest) | 35164939 | chr4:103188709 | C=0.92, T=0.08 | NR | NA | NA | 6.00E-169 |
| LST | rs13107325 | Moderate-to-vigorous intensity physical activity during leisure time | 36071172 | chr4:103188709 | C=0.92, T=0.08 | 0.93 | 0.031 | 0.017-0.045 | 2.00E-06 |
| LST | rs13107325 | Leisure screen time | 36071172 | chr4:103188709 | C=0.92, T=0.08 | 0.926 | 0.04 | 0.026-0.054 | 2.00E-09 |
| LST | rs13107325 | Risk-taking behavior (multivariate analysis) | 36324656 | chr4:103188709 | C=0.92, T=0.08 | NR | NA | NA | 2.00E-17 |
| LST | rs13107325 | Insomnia | 35835914 | chr4:103188709 | C=0.92, T=0.08 | NR | 0.017 | 0.013-0.021 | 7.00E-24 |
| LST | rs13107325 | 2-oxoarginine levels | 35050183 | chr4:103188709 | C=0.92, T=0.08 | NR | 0.19 | 0.14-0.24 | 1.00E-11 |
| LST | rs13107325 | Triglyceride levels in non-type 2 diabetes | 36269708 | chr4:103188709 | C=0.92, T=0.08 | NR | NA | NA | 2.00E-10 |
| LST | rs13107325 | Albumin levels | 34321204 | chr4:103188709 | C=0.92, T=0.08 | 0.08 | 0.21 | 0.16-0.26/ | 2.00E-19 |
| LST | rs13107325 | Insomnia | 35835914 | chr4:103188709 | C=0.92, T=0.08 | NR | 0.019 | 0.015-0.023 | 1.00E-17 |
| LST | rs13107325 | Intelligence | 36150907 | chr4:103188709 | C=0.92, T=0.08 | NR | NA | NA | 7.00E-10 |
| LST | rs13107325 | Common executive function | 36150907 | chr4:103188709 | C=0.92, T=0.08 | NR | NA | NA | 1.00E-08 |
| LST | rs13107325 | X-12339 levels | 36357675 | chr4:103188709 | C=0.92, T=0.08 | 0.0744 | 0.17742912 | 0.14-0.22 | 3.00E-20 |
| LST | rs13107325 | Maximum habitual alcohol consumption (MTAG) | 36301540 | chr4:103188709 | C=0.92, T=0.08 | NR | 0.05433835 | 0.046-0.063 | 6.00E-36 |
| LST | rs13107325 | Problematic alcohol use (MTAG) | 36301540 | chr4:103188709 | C=0.92, T=0.08 | NR | 0.0551 | 0.047-0.063 | 6.00E-40 |
| LST | rs13107325 | Maximum habitual alcohol consumption | 36301540 | chr4:103188709 | C=0.92, T=0.08 | NR | NA | NA | 9.00E-15 |
| LST | rs13107325 | Maximum habitual alcohol consumption | 36301540 | chr4:103188709 | C=0.92, T=0.08 | NR | NA | NA | 7.00E-15 |
| LST | rs13107325 | X-12681 levels | 36357675 | chr4:103188709 | C=0.92, T=0.08 | 0.074 | 0.24227376 | 0.21-0.28 | 3.00E-37 |
| LST | rs13107325 | Total cholesterol levels | 34887591 | chr4:103188709 | C=0.92, T=0.08 | 0.0640003 | 0.0395317 | 0.034-0.045 | 1.00E-34 |
| LST | rs13107325 | Systolic blood pressure | 35762941 | chr4:103188709 | C=0.92, T=0.08 | NR | 0.8966 | 0.76-1.04 | 5.00E-36 |
| LST | rs13107325 | Triglyceride levels | 34887591 | chr4:103188709 | C=0.92, T=0.08 | NR | NA | NA | 2.00E-37 |
| LST | rs13107325 | Homoarginine levels | 36357675 | chr4:103188709 | C=0.92, T=0.08 | 0.0739 | 0.16970313 | 0.13-0.21 | 5.00E-19 |
| LST | rs13107325 | Triglyceride levels | 34887591 | chr4:103188709 | C=0.92, T=0.08 | 0.0641593 | 0.0372865 | 0.032-0.043 | 2.00E-27 |
| LST | rs13107325 | Waist circumference adjusted for body mass index | 34021172 | chr4:103188709 | C=0.92, T=0.08 | NR | 0.040075 | 0.029-0.051 | 2.00E-13 |
| LST | rs13107325 | Diastolic blood pressure | 35762941 | chr4:103188709 | C=0.92, T=0.08 | NR | 0.6382 | 0.56-0.72 | 6.00E-54 |
| LST | rs13107325 | Total cholesterol levels | 34887591 | chr4:103188709 | C=0.92, T=0.08 | NR | NA | NA | 6.00E-45 |
| LST | rs13107325 | Drinks per week | 36477530 | chr4:103188709 | C=0.92, T=0.08 | 0.0774 | 0.0269 | 0.024-0.03 | 2.00E-56 |
| LST | rs13107325 | High density lipoprotein cholesterol levels | 34887591 | chr4:103188709 | C=0.92, T=0.08 | NR | NA | NA | 1.00E-185 |
| LST | rs13107325 | High-density lipoprotein levels (MTAG) | 36376304 | chr4:103188709 | C=0.92, T=0.08 | NR | 0.07886 | 0.069-0.089 | 2.00E-57 |
| LST | rs13107325 | Schizophrenia | 35396580 | chr4:103188709 | C=0.92, T=0.08 | 0.94 | 0.85095 | 0.82-0.88 | 3.00E-23 |
| LST | rs13107325 | Attention deficit hyperactivity disorder or autism spectrum disorder or intelligence (pleiotropy) | 35764056 | chr4:103188709 | C=0.92, T=0.08 | NR | NA | NA | 2.00E-22 |
| LST | rs13107325 | Low density lipoprotein cholesterol levels | 34887591 | chr4:103188709 | C=0.92, T=0.08 | NR | NA | NA | 1.00E-17 |
| LST | rs13107325 | Diastolic blood pressure x depressive symptoms interaction (2df test) | 34734193 | chr4:103188709 | C=0.92, T=0.08 | NR | NA | NA | 8.00E-11 |
| LST | rs13107325 | Drinks per week | 36477530 | chr4:103188709 | C=0.92, T=0.08 | 0.0737 | 0.0264 | 0.023-0.03 | 3.00E-59 |
| LST | rs13107325 | Femur length to body height ratio | 37471560 | chr4:103188709 | C=0.92, T=0.08 | NR | 0.00139366 | 0.0012-0.0016 | 3.00E-43 |
| LST | rs13107325 | Cholesteryl esters in large HDL (UKB data field 23562) | 36764567 | chr4:103188709 | C=0.92, T=0.08 | 0.0749924 | 0.0666365 | 0.051-0.082 | 2.00E-16 |
| LST | rs13107325 | Argininate levels | 36635386 | chr4:103188709 | C=0.92, T=0.08 | 0.0739321 | 0.253074 | 0.2-0.31 | 7.00E-19 |
| LST | rs13107325 | Low-density lipoprotein levels (MTAG) | 36376304 | chr4:103188709 | C=0.92, T=0.08 | NR | 0.02936 | 0.024-0.035 | 9.00E-29 |
| LST | rs13107325 | Medial temporal thickness (unadjusted for global measures) | 36893272 | chr4:103188709 | C=0.92, T=0.08 | NR | 0.0772 | 0.05-0.104 | 2.00E-08 |
| LST | rs13107325 | Gamma glutamyl transferase levels | 38632349 | chr4:103188709 | C=0.92, T=0.08 | NR | 0.0074 | 0.0054-0.0094 | 2.00E-14 |
| LST | rs13107325 | Age when finished full-time education (standard GWA) | 37106081 | chr4:103188709 | C=0.92, T=0.08 | NR | 0.049374 | 0.033-0.066 | 4.00E-09 |
| LST | rs13107325 | Idiopathic knee osteoarthritis | 37247657 | chr4:103188709 | C=0.92, T=0.08 | 0.07 | 1.1 | 1.08-1.12 | 3.00E-18 |
| LST | rs13107325 | Genetically independent pain phenotypes (GIP1) | 32587327 | chr4:103188709 | C=0.92, T=0.08 | 0.009255 | 0.032105863 | 0.022-0.042 | 9.00E-10 |
| LST | rs13107325 | Neck pain or shoulder pain | 32587327 | chr4:103188709 | C=0.92, T=0.08 | NR | NA | NA | 2.00E-08 |
| LST | rs13107325 | Triglyceride levels (MTAG) | 36376304 | chr4:103188709 | C=0.92, T=0.08 | NR | 0.030182 | 0.021-0.039 | 1.00E-10 |
| LST | rs13107325 | Serum urate levels | 38658550 | chr4:103188709 | C=0.92, T=0.08 | NR | 0.0327 | 0.022-0.043 | 6.00E-10 |
| LST | rs13107325 | T2 brain MRIs Unsupervised Deep learning derived Imaging Phenotypes (dimension 67) | 38580839 | chr4:103188709 | C=0.92, T=0.08 | NR | NA | NA | 5.00E-17 |
| LST | rs13107325 | Serum urate levels | 38658550 | chr4:103188709 | C=0.92, T=0.08 | NR | 0.0321 | 0.022-0.042 | 1.00E-09 |
| LST | rs13107325 | Systolic blood pressure | 38689001 | chr4:103188709 | C=0.92, T=0.08 | NR | 0.7456 | 0.66-0.84 | 6.00E-59 |
| LST | rs13107325 | Left-hemisphere salience/ventral attention network to amygdala white-matter structural connectivity | 38438384 | chr4:103188709 | C=0.92, T=0.08 | NR | 0.106565 | 0.077-0.137 | 3.00E-12 |
| LST | rs13107325 | Left-hemisphere limbic network to caudate white-matter structural connectivity | 38438384 | chr4:103188709 | C=0.92, T=0.08 | NR | 0.129895 | 0.1-0.16 | 2.00E-18 |
| LST | rs13107325 | Left-hemisphere limbic network to hippocampus white-matter structural connectivity | 38438384 | chr4:103188709 | C=0.92, T=0.08 | NR | 0.125233 | 0.094-0.156 | 2.00E-15 |
| LST | rs13107325 | Right-hemisphere somatomotor network to amygdala white-matter structural connectivity | 38438384 | chr4:103188709 | C=0.92, T=0.08 | NR | 0.0936863 | 0.064-0.124 | 1.00E-09 |
| LST | rs13107325 | Right-hemisphere limbic network to hippocampus white-matter structural connectivity | 38438384 | chr4:103188709 | C=0.92, T=0.08 | NR | 0.199318 | 0.17-0.23 | 3.00E-35 |
| LST | rs13107325 | Right-hemisphere default mode network to hippocampus white-matter structural connectivity | 38438384 | chr4:103188709 | C=0.92, T=0.08 | NR | 0.124226 | 0.094-0.155 | 1.00E-15 |
| LST | rs13107325 | Systolic blood pressure | 38459180 | chr4:103188709 | C=0.92, T=0.08 | NR | 0.04 | 0.032-0.048 | 1.00E-16 |
| LST | rs13107325 | Diastolic blood pressure | 38459180 | chr4:103188709 | C=0.92, T=0.08 | NR | 0.06 | 0.048-0.072 | 9.00E-21 |
| LST | rs13107325 | Diastolic blood pressure | 38459180 | chr4:103188709 | C=0.92, T=0.08 | NR | 0.06 | 0.052-0.068 | 2.00E-43 |
| LST | rs13107325 | sMRI-derived phenotypes (MOSTest) (pleiotropy) | 38531894 | chr4:103188709 | C=0.92, T=0.08 | NR | NA | NA | 4.94065645841247e-324 |
| LST | rs13107325 | Short sleep duration (<5 hours) | 37770476 | chr4:103188709 | C=0.92, T=0.08 | NR | 0.0852 | 0.061-0.11 | 9.00E-12 |
| LST | rs13107325 | Short sleep duration (<5 hours) | 37770476 | chr4:103188709 | C=0.92, T=0.08 | NR | NA | NA | 4.00E-12 |
| LST | rs13107325 | Tinnitus | 38242899 | chr4:103188709 | C=0.92, T=0.08 | 0.0791 | 6.444 | NA | 1.00E-10 |
| LST | rs13107325 | Tinnitus | 38242899 | chr4:103188709 | C=0.92, T=0.08 | 0.0707 | 6.114 | NA | 1.00E-09 |
| LST | rs13107325 | Left-hemisphere somatomotor network to amygdala white-matter structural connectivity | 38438384 | chr4:103188709 | C=0.92, T=0.08 | NR | 0.0946559 | 0.064-0.125 | 2.00E-09 |
| LST | rs13107325 | Diastolic blood pressure | 38689001 | chr4:103188709 | C=0.92, T=0.08 | NR | 0.6118 | 0.56-0.67 | 5.00E-100 |
| LST | rs13107325 | Pulse pressure | 38689001 | chr4:103188709 | C=0.92, T=0.08 | NR | 0.1862 | 0.12-0.25 | 2.00E-08 |
| LST | rs13107325 | Cannabis use disorder (MTAG) | 37985822 | chr4:103188709 | C=0.92, T=0.08 | 0.08 | 0.041 | 0.03-0.052 | 5.00E-13 |
| LST | rs13107325 | Osteoarthritis | 36411363 | chr4:103188709 | C=0.92, T=0.08 | 0.005 | 1.09 | 1.06-1.12 | 1.00E-08 |
| LST | rs13107325 | Osteoarthritis | 36411363 | chr4:103188709 | C=0.92, T=0.08 | NR | 1.08 | 1.05-1.11 | 4.00E-10 |
| LST | rs13107325 | Osteoarthritis | 36411363 | chr4:103188709 | C=0.92, T=0.08 | NR | 1.08939 | 1.06-1.12 | 3.00E-08 |
| LST | rs13107325 | Ventromedial occipital thickness (unadjusted for global measures) | 36893272 | chr4:103188709 | C=0.92, T=0.08 | NR | 0.1467 | 0.12-0.17 | 4.00E-26 |
| LST | rs13107325 | Occipital thickness (unadjusted for global measures) | 36893272 | chr4:103188709 | C=0.92, T=0.08 | NR | 0.089 | 0.062-0.116 | 1.00E-10 |
| LST | rs13107325 | Ventral frontal thickness (unadjusted for global measures) | 36893272 | chr4:103188709 | C=0.92, T=0.08 | NR | 0.0721 | 0.045-0.099 | 2.00E-07 |
| LST | rs13107325 | Temporal pole thickness (unadjusted for global measures) | 36893272 | chr4:103188709 | C=0.92, T=0.08 | NR | 0.0754 | 0.048-0.103 | 5.00E-08 |
| LST | rs13107325 | Non-response to survey questionnaires: prefer not to answer item | 37386106 | chr4:103188709 | C=0.92, T=0.08 | NR | NA | NA | 7.00E-10 |
| LST | rs13107325 | Post-traumatic stress disorder (MTAG) | 37218628 | chr4:103188709 | C=0.92, T=0.08 | 0.07952 | 0.022835834 | 0.015-0.031 | 6.00E-09 |
| LST | rs13107325 | Gastroesophageal reflux disease or posttraumatic stress disorder (pleiotropy) | 36753304 | chr4:103188709 | C=0.92, T=0.08 | NR | NA | NA | 4.00E-08 |
| LST | rs13107325 | Gastroesophageal reflux disease or schizophrenia (pleiotropy) | 36753304 | chr4:103188709 | C=0.92, T=0.08 | NR | NA | NA | 2.00E-14 |
| LST | rs13107325 | Osteoarthritis | 36411363 | chr4:103188709 | C=0.92, T=0.08 | NR | 1.09 | 0.01-1.07 | 6.00E-17 |
| LST | rs13107325 | Body mass index or knee osteoarthritis (pleiotropy) | 36889626 | chr4:103188709 | C=0.92, T=0.08 | NR | NA | NA | 2.00E-48 |
| LST | rs13107325 | Body mass index or hip osteoarthritis (pleiotropy) | 36889626 | chr4:103188709 | C=0.92, T=0.08 | NR | NA | NA | 2.00E-52 |
| LST | rs13107325 | Body mass index or osteoarthritis (pleiotropy) | 36889626 | chr4:103188709 | C=0.92, T=0.08 | NR | NA | NA | 1.00E-25 |
| LST | rs13107325 | Body mass index or osteoarthritis (pleiotropy) | 36889626 | chr4:103188709 | C=0.92, T=0.08 | NR | NA | NA | 2.00E-31 |
| LST | rs13107325 | Body mass index or osteoarthritis (pleiotropy) | 36889626 | chr4:103188709 | C=0.92, T=0.08 | NR | NA | NA | 2.00E-51 |
| LST | rs13107325 | Personality traits or cognitive traits (multivariate analysis) | 37365406 | chr4:103188709 | C=0.92, T=0.08 | NR | NA | NA | 1.00E-25 |
| LST | rs13107325 | Myelin protein P0 levels | 34648354 | chr4:103188709 | C=0.92, T=0.08 | 0.07 | 0.238 | 0.19-0.28 | 3.00E-25 |
| LST | rs13107325 | Apolipoprotein A1 levels (UKB data field 23440) | 36764567 | chr4:103188709 | C=0.92, T=0.08 | 0.0749924 | 0.0680362 | 0.052-0.084 | 2.00E-16 |
| LST | rs13107325 | Sphingomyelins levels (UKB data field 23438) | 36764567 | chr4:103188709 | C=0.92, T=0.08 | 0.0749819 | 0.0534147 | 0.037-0.07 | 2.00E-10 |
| LST | rs13107325 | Cholesteryl esters in HDL (UKB data field 23418) | 36764567 | chr4:103188709 | C=0.92, T=0.08 | 0.0749924 | 0.0735194 | 0.058-0.09 | 2.00E-19 |
| LST | rs13107325 | Free cholesterol in HDL (UKB data field 23422) | 36764567 | chr4:103188709 | C=0.92, T=0.08 | 0.0749924 | 0.0695127 | 0.054-0.085 | 5.00E-18 |
| LST | rs13107325 | Body mass index | 36581621 | chr4:103188709 | C=0.92, T=0.08 | NR | 0.0522 | 0.047-0.057 | 2.00E-94 |
| LST | rs13107325 | Phospholipids in HDL (UKB data field 23414) | 36764567 | chr4:103188709 | C=0.92, T=0.08 | 0.0749924 | 0.0678526 | 0.052-0.084 | 9.00E-17 |
| LST | rs13107325 | Concentration of HDL particles (UKB data field 23430) | 36764567 | chr4:103188709 | C=0.92, T=0.08 | 0.0749924 | 0.0641545 | 0.048-0.081 | 3.00E-14 |
| LST | rs13107325 | HDL cholesterol levels (UKB data field 23406) | 36764567 | chr4:103188709 | C=0.92, T=0.08 | 0.0749924 | 0.0732333 | 0.057-0.089 | 2.00E-19 |
| LST | rs13107325 | Total concentration of lipoprotein particles (UKB data field 23427) | 36764567 | chr4:103188709 | C=0.92, T=0.08 | 0.0749924 | 0.0602993 | 0.044-0.077 | 1.00E-12 |
| LST | rs13107325 | Total lipids in HDL (UKB data field 23426) | 36764567 | chr4:103188709 | C=0.92, T=0.08 | 0.0749924 | 0.0700924 | 0.054-0.086 | 6.00E-18 |
| LST | rs13107325 | Protein SET levels | 34648354 | chr4:103188709 | C=0.92, T=0.08 | 0.07 | 0.193 | 0.14-0.24 | 4.00E-14 |
| LST | rs13107325 | Intelligence | 36378351 | chr4:103188709 | C=0.92, T=0.08 | NR | NA | NA | 5.00E-23 |
| LST | rs13107325 | Hemoglobin concentration | 35964923 | chr4:103188709 | C=0.92, T=0.08 | 0.07419 | 0.04091 | NA | 9.00E-11 |
| LST | rs13107325 | Body mass index (MTAG) | 36376304 | chr4:103188709 | C=0.92, T=0.08 | NR | 0.041619 | 0.036-0.047 | 4.00E-46 |
| LST | rs13107325 | Heparan-sulfate 6-O-sulfotransferase 1 levels | 34648354 | chr4:103188709 | C=0.92, T=0.08 | 0.07 | 0.181 | 0.13-0.23 | 1.00E-12 |
| LST | rs13107325 | Non-surgical knee osteoarthritis | 36376028 | chr4:103188709 | C=0.92, T=0.08 | 0.044 | 1.1 | 1.06-1.14 | 9.00E-08 |
| LST | rs13107325 | Bifunctional heparan sulfate N-deacetylase/N-sulfotransferase 1 levels | 34648354 | chr4:103188709 | C=0.92, T=0.08 | 0.07 | 0.216 | 0.17-0.27 | 9.00E-17 |
| LST | rs13107325 | Protein kinase C-binding protein NELL1 levels | 34648354 | chr4:103188709 | C=0.92, T=0.08 | 0.07 | 0.185 | 0.13-0.24 | 8.00E-13 |
| LST | rs13107325 | Cyclic AMP-dependent transcription factor ATF-6 alpha levels | 34648354 | chr4:103188709 | C=0.92, T=0.08 | 0.07 | 0.214 | 0.17-0.26 | 1.00E-19 |
| LST | rs13107325 | Obstructive sleep apnea | 36989840 | chr4:103188709 | C=0.92, T=0.08 | 0.086427 | 1.073008 | 1.05-1.09 | 6.00E-13 |
| LST | rs13107325 | Free cholesterol in very large HDL | 38448586 | chr4:103188709 | C=0.92, T=0.08 | 0.0682 | 0.0764 | 0.058-0.095 | 4.00E-16 |
| LST | rs13107325 | Total lipids in very large HDL | 38448586 | chr4:103188709 | C=0.92, T=0.08 | 0.0682 | 0.0719 | 0.053-0.09 | 2.00E-14 |
| LST | rs13107325 | Mean diameter of HDL particles | 38448586 | chr4:103188709 | C=0.92, T=0.08 | 0.0681 | 0.0628 | 0.045-0.081 | 2.00E-11 |
| LST | rs13107325 | Phospholipids in very large HDL | 38448586 | chr4:103188709 | C=0.92, T=0.08 | 0.0683 | 0.0611 | 0.043-0.08 | 7.00E-11 |
| LST | rs13107325 | Total cholesterol levels in very large HDL | 38448586 | chr4:103188709 | C=0.92, T=0.08 | 0.0676 | 0.0777 | 0.059-0.096 | 2.00E-16 |
| LST | rs13107325 | Cholesterol esters in very large HDL | 38448586 | chr4:103188709 | C=0.92, T=0.08 | 0.0675 | 0.0759 | 0.057-0.095 | 1.00E-15 |
| LST | rs13107325 | Multi-trait sum score | 37277458 | chr4:103188709 | C=0.92, T=0.08 | NR | NA | NA | 1.00E-08 |
| LST | rs13107325 | Free cholesterol to total lipids in medium HDL percentage (UKB data field 23642) | 36764567 | chr4:103188709 | C=0.92, T=0.08 | 0.0749924 | 0.0689706 | 0.053-0.085 | 1.00E-17 |
| LST | rs13107325 | Free cholesterol in medium HDL (UKB data field 23570) | 36764567 | chr4:103188709 | C=0.92, T=0.08 | 0.0749924 | 0.069249 | 0.053-0.085 | 3.00E-17 |
| LST | rs13107325 | Phospholipids in medium HDL (UKB data field 23567) | 36764567 | chr4:103188709 | C=0.92, T=0.08 | 0.0749924 | 0.0607746 | 0.044-0.077 | 5.00E-13 |
| LST | rs13107325 | Cholesterol in medium HDL (UKB data field 23568) | 36764567 | chr4:103188709 | C=0.92, T=0.08 | 0.0749924 | 0.0705141 | 0.054-0.087 | 2.00E-17 |
| LST | rs13107325 | Cholesteryl esters in medium HDL (UKB data field 23569) | 36764567 | chr4:103188709 | C=0.92, T=0.08 | 0.0749924 | 0.0704255 | 0.054-0.087 | 3.00E-17 |
| LST | rs13107325 | Concentration of medium HDL particles (UKB data field 23565) | 36764567 | chr4:103188709 | C=0.92, T=0.08 | 0.0749924 | 0.0677031 | 0.051-0.084 | 4.00E-16 |
| LST | rs13107325 | Total lipids in medium HDL (UKB data field 23566) | 36764567 | chr4:103188709 | C=0.92, T=0.08 | 0.0749924 | 0.0647636 | 0.048-0.081 | 9.00E-15 |
| LST | rs13107325 | Phospholipids to total lipids in very small VLDL percentage (UKB data field 23604) | 36764567 | chr4:103188709 | C=0.92, T=0.08 | 0.0749924 | 0.0580204 | 0.041-0.075 | 4.00E-11 |
| LST | rs13107325 | Free cholesterol to total lipids in very large HDL percentage (UKB data field 23632) | 36764567 | chr4:103188709 | C=0.92, T=0.08 | 0.0749941 | 0.0596599 | 0.044-0.075 | 1.00E-13 |
| LST | rs13107325 | Phospholipids to total lipids in medium HDL percentage (UKB data field 23639) | 36764567 | chr4:103188709 | C=0.92, T=0.08 | 0.0749924 | 0.0580962 | 0.041-0.075 | 1.00E-11 |
| LST | rs13107325 | Cholesterol to total lipids in medium HDL percentage (UKB data field 23640) | 36764567 | chr4:103188709 | C=0.92, T=0.08 | 0.0749924 | 0.0552683 | 0.038-0.072 | 1.00E-10 |
| LST | rs13107325 | Alcohol use disorder | 37282553 | chr4:103188709 | C=0.92, T=0.08 | 0.065 | 9.703 | - | 3.00E-22 |
| LST | rs13107325 | Alcohol use disorder | 37282553 | chr4:103188709 | C=0.92, T=0.08 | 0.0808 | 0.8773 | 0.85-0.9 | 8.00E-23 |
| LST | rs13107325 | Lung function (forced vital capacity) | 36914875 | chr4:103188709 | C=0.92, T=0.08 | 0.0694 | 6.707 | NA | 2.00E-11 |
| LST | rs13107325 | Body mass index | 37280435 | chr4:103188709 | C=0.92, T=0.08 | NR | 0.0461563 | 0.039-0.053 | 4.00E-38 |
| LST | rs13107325 | Alcohol use disorder (MTAG) | 37156939 | chr4:103188709 | C=0.92, T=0.08 | NR | 0.0478908 | 0.037-0.059 | 1.00E-18 |
| LST | rs743699 | Leisure screen time | 36071172 | chr4:3305116 | A=0.745, G=0.255 | 0.743 | 0.027 | 0.019-0.035 | 1.00E-09 |
| LST | rs4416502 | Leisure screen time | 36071172 | chr4:77030872 | A=0.232, G=0.768 | 0.8 | 0.029 | 0.019-0.039 | 1.00E-09 |
| LST | rs396321 | Leisure screen time | 36071172 | chr5:112113735 | C=0.495, T=0.505 | 0.513 | 0.021 | 0.013-0.029 | 1.00E-09 |
| LST | rs10041724 | Leisure sedentary behaviour (television watching) | 32317632 | chr5:124273520 | C=0.204, T=0.796 | NR | 0.0180931 | 0.013-0.023 | 4.00E-11 |
| LST | rs2964252 | Educational attainment (MTAG) | 30038396 | chr5:152067929 | A=0.29, G=0.71 | 0.3132 | 0.009 | 0.0063-0.0117 | 2.00E-10 |
| LST | rs2964252 | Leisure screen time | 36071172 | chr5:152067929 | A=0.29, G=0.71 | 0.316 | 0.024 | 0.016-0.032 | 3.00E-10 |
| LST | rs2964252 | Educational attainment | 35361970 | chr5:152067929 | A=0.29, G=0.71 | 0.3111 | 0.010242 | 0.008-0.0125 | 1.00E-19 |
| LST | rs1947066 | Leisure screen time | 36071172 | chr5:161101615 | A=0.823, G=0.177 | 0.197 | 0.03 | 0.022-0.038 | 9.00E-12 |
| LST | rs262890 | Leisure sedentary behaviour (television watching) | 32317632 | chr5:62930015 | A=0.703, G=0.297 | NR | 0.0185683 | 0.014-0.023 | 3.00E-15 |
| LST | rs262890 | Intelligence | 29942086 | chr5:62930015 | A=0.703, G=0.297 | NR | 6.245 | - | 4.00E-10 |
| LST | rs262890 | Leisure screen time | 36071172 | chr5:62930015 | A=0.703, G=0.297 | 0.7 | 0.034 | 0.026-0.042 | 2.00E-16 |
| LST | rs262890 | Attention deficit hyperactivity disorder or autism spectrum disorder or intelligence (pleiotropy) | 35764056 | chr5:62930015 | A=0.703, G=0.297 | NR | NA | NA | 3.00E-09 |
| LST | rs249960 | Leisure screen time | 36071172 | chr5:96164771 | A=0.822, G=0.178 | 0.182 | 0.03 | 0.02-0.04 | 2.00E-09 |
| LST | rs78394231 | Leisure sedentary behaviour (television watching) | 32317632 | chr6:107649123 | C=0.114, T=0.886 | NR | 0.0208531 | 0.014-0.028 | 1.00E-08 |
| LST | rs78394231 | Leisure screen time | 36071172 | chr6:107649123 | C=0.114, T=0.886 | 0.902 | 0.038 | 0.024-0.052 | 4.00E-09 |
| LST | rs558134 | Leisure screen time | 36071172 | chr6:12693454 | C=0.583, T=0.417 | 0.384 | 0.023 | 0.015-0.031 | 5.00E-10 |
| LST | rs58541850 | Leisure screen time | 36071172 | chr6:166165563 | A=0.056, G=0.944 | 0.942 | 0.052 | 0.036-0.068 | 2.00E-10 |
| LST | rs9278004 | Idiopathic achalasia | 34197731 | chr6:33319815 | A=0.134, G=0.866 | 0.045 | 2.9 | 1.83-4.6 | 6.00E-06 |
| LST | rs6457816 | Leisure screen time | 36071172 | chr6:35362848 | C=0.073, T=0.927 | 0.934 | 0.041 | 0.027-0.055 | 4.00E-09 |
| LST | rs2529484 | Leisure screen time | 36071172 | chr7:111180444 | C=0.382, G=0.618 | 0.649 | 0.022 | 0.014-0.03 | 1.00E-09 |
| LST | rs13235840 | Leisure screen time | 36071172 | chr7:133505091 | A=0.778, T=0.222 | 0.819 | 0.031 | 0.021-0.041 | 3.00E-10 |
| LST | rs17621391 | Leisure screen time | 36071172 | chr7:140176596 | C=0.291, T=0.709 | 0.265 | 0.024 | 0.016-0.032 | 2.00E-09 |
| LST | rs10253861 | Height | 30595370 | chr7:8110475 | A=0.524, G=0.476 | NR | NA | NA | 3.00E-40 |
| LST | rs10253861 | Height | 35399580 | chr7:8110475 | A=0.524, G=0.476 | 0.5289 | 0.0282 | 0.018-0.039 | 2.00E-07 |
| LST | rs10253861 | Bread type: White vs wholemeal/wholegrain and brown (UKB data field 1448) | 32193382 | chr7:8110475 | A=0.524, G=0.476 | 0.447798 | 0.0133401 | 0.0088-0.0179 | 1.00E-08 |
| LST | rs10253861 | Height | 36224396 | chr7:8110475 | A=0.524, G=0.476 | 0.5523 | 0.0158 | 0.011-0.021 | 7.00E-11 |
| LST | rs10253861 | Height | 36224396 | chr7:8110475 | A=0.524, G=0.476 | 0.4552 | 0.0212 | 0.02-0.022 | 6.00E-255 |
| LST | rs7821826 | Leisure screen time | 36071172 | chr8:10769439 | C=0.512, T=0.488 | 0.508 | 0.021 | 0.013-0.029 | 3.00E-09 |
| LST | rs1362910 | Highest math class taken | 30038396 | chr8:30856464 | A=0.386, G=0.614 | 0.4168 | 0.0179 | 0.013-0.022 | 6.00E-15 |
| LST | rs1362910 | Body mass index | 36581621 | chr8:30856464 | A=0.386, G=0.614 | NR | 0.0126 | 0.0097-0.0155 | 2.00E-16 |
| LST | rs12678836 | Leisure screen time | 36071172 | chr8:92690148 | A=0.413, C=0.587 | 0.576 | 0.023 | 0.015-0.031 | 5.00E-11 |
| LST | rs1999065 | Leisure screen time | 36071172 | chr9:120514574 | C=0.671, T=0.329 | 0.662 | 0.025 | 0.017-0.033 | 1.00E-11 |
| LST | rs28458909 | Morning person | 30595370 | chr9:140257189 | C=0.862, T=0.138 | NR | NA | NA | 3.00E-23 |
| LST | rs28458909 | Morningness | 30804565 | chr9:140257189 | C=0.862, T=0.138 | 0.1224 | 0.03 | 0.022-0.038 | 2.00E-16 |
| LST | rs28458909 | Ease of getting up in the morning | 28604731 | chr9:140257189 | C=0.862, T=0.138 | 0.12 | 0.036 | 0.024-0.048 | 6.00E-09 |
| LST | rs28458909 | Chronotype | 30696823 | chr9:140257189 | C=0.862, T=0.138 | 0.8775 | 1.0727227 | NA | 1.00E-33 |
| LST | rs28458909 | Morning person | 30696823 | chr9:140257189 | C=0.862, T=0.138 | 0.8775 | 1.0713521 | NA | 1.00E-33 |
| LST | rs28458909 | Educational attainment | 35361970 | chr9:140257189 | C=0.862, T=0.138 | 0.1235 | 0.0190198 | 0.016-0.022 | 6.00E-31 |
| LST | rs7875078 | Highest math class taken (MTAG) | 30038396 | chr9:14494845 | A=0.476, C=0.524 | 0.4595 | 0.0111 | 0.0078-0.0144 | 2.00E-11 |
| LST | rs7875078 | Educational attainment (years of education) | 30038396 | chr9:14494845 | A=0.476, C=0.524 | 0.4564 | 0.0078 | 0.0051-0.0105 | 3.00E-08 |
| LST | rs7875078 | Educational attainment (MTAG) | 30038396 | chr9:14494845 | A=0.476, C=0.524 | 0.4564 | 0.0086 | 0.0061-0.0111 | 5.00E-11 |
| LST | rs7875078 | Leisure screen time (MTAG) | 36071172 | chr9:14494845 | A=0.476, C=0.524 | NR | 0.021 | 0.015-0.027 | 1.00E-09 |
| LST | rs7875078 | Educational attainment | 35361970 | chr9:14494845 | A=0.476, C=0.524 | 0.4609 | 0.00880226 | 0.0067-0.0109 | 5.00E-17 |
| LST | rs2783992 | Leisure screen time | 36071172 | chr9:1722044 | C=0.4, T=0.6 | 0.539 | 0.024 | 0.016-0.032 | 3.00E-10 |
| LST | rs34864022 | Leisure sedentary behaviour (television watching) | 32317632 | chr9:22609110 | A=0.935, G=0.065 | NR | 0.0263815 | 0.018-0.035 | 1.00E-09 |
| LST | rs34864022 | Driving (hours per day) | 32317632 | chr9:22609110 | A=0.935, G=0.065 | NR | 0.0219251 | 0.013-0.031 | 1.00E-06 |
| LST | rs34864022 | Leisure screen time | 36071172 | chr9:22609110 | A=0.935, G=0.065 | 0.934 | 0.048 | 0.032-0.064 | 5.00E-10 |
| MVPA | rs12357890 | Apolipoprotein A1 levels | 32203549 | chr10:99762693 | A=0.447, G=0.553 | 0.443073 | 0.0115686 | 0.0077-0.0155 | 6.00E-09 |
| MVPA | rs12357890 | C-reactive protein levels | 31900758 | chr10:99762693 | A=0.447, G=0.553 | 0.441773 | 0.0145353 | 0.01-0.019 | 6.00E-12 |
| MVPA | rs12357890 | Adult body size | 32376654 | chr10:99762693 | A=0.447, G=0.553 | 0.442815 | 0.0121927 | 0.0095-0.0149 | 2.00E-18 |
| MVPA | rs12357890 | Cholesteryl ester levels in very large VLDL | 35213538 | chr10:99762693 | A=0.447, G=0.553 | 0.442785 | 0.0235745 | 0.016-0.032 | 1.00E-08 |
| MVPA | rs12357890 | C-reactive protein | 34594039 | chr10:99762693 | A=0.447, G=0.553 | NR | 0.0143 | 0.0096-0.019 | 5.00E-09 |
| MVPA | rs12357890 | Weight | 34594039 | chr10:99762693 | A=0.447, G=0.553 | NR | 0.0167 | 0.013-0.021 | 1.00E-15 |
| MVPA | rs12357890 | Cholesterol levels in large VLDL | 35213538 | chr10:99762693 | A=0.447, G=0.553 | 0.442785 | 0.0247656 | 0.017-0.033 | 1.00E-09 |
| MVPA | rs12357890 | Cholesteryl ester levels in large VLDL | 35213538 | chr10:99762693 | A=0.447, G=0.553 | 0.442785 | 0.0231358 | 0.015-0.031 | 2.00E-08 |
| MVPA | rs12357890 | Triglycerides | 34594039 | chr10:99762693 | A=0.447, G=0.553 | NR | 0.0172 | 0.013-0.022 | 2.00E-13 |
| MVPA | rs12357890 | High density lipoprotein cholesterol levels | 33462484 | chr10:99762693 | A=0.447, G=0.553 | NR | 0.0147 | 0.0098-0.0196 | 5.00E-09 |
| MVPA | rs12357890 | Whole body fat mass (UKB data field 23100) | 38538606 | chr10:99762693 | A=0.447, G=0.553 | NR | 0.0178607 | 0.013-0.023 | 8.00E-14 |
| MVPA | rs12357890 | Body mass index | 38538606 | chr10:99762693 | A=0.447, G=0.553 | NR | 0.0192821 | 0.015-0.024 | 2.00E-15 |
| MVPA | rs568546 | Moderate-to-vigorous intensity physical activity during leisure time (MTAG) | 36071172 | chr11:107321156 | C=0.478, T=0.522 | NR | 0.022 | 0.014-0.03 | 9.00E-10 |
| MVPA | rs1424751 | Reticulocyte count | 32888494 | chr11:57479732 | C=0.43, G=0.57 | 0.282451 | 0.01665921 | 0.012-0.022 | 1.00E-10 |
| MVPA | rs1625595 | Moderate-to-vigorous intensity physical activity during leisure time | 36071172 | chr11:66078129 | C=0.519, T=0.481 | 0.525 | 0.021 | 0.015-0.027 | 2.00E-11 |
| MVPA | rs385301 | Moderate-to-vigorous intensity physical activity during leisure time | 36071172 | chr17:19806828 | C=0.719, T=0.281 | 0.736 | 0.028 | 0.018-0.038 | 2.00E-09 |
| MVPA | rs9903845 | Moderate-to-vigorous intensity physical activity during leisure time | 36071172 | chr17:50291181 | A=0.297, C=0.703 | 0.691 | 0.02 | 0.014-0.026 | 4.00E-09 |
| MVPA | rs1160545 | Moderate-to-vigorous intensity physical activity during leisure time | 36071172 | chr2:100832269 | C=0.609, T=0.391 | 0.403 | 0.025 | 0.017-0.033 | 2.00E-09 |
| MVPA | rs1160545 | Leisure screen time | 36071172 | chr2:100832269 | C=0.609, T=0.391 | 0.403 | 0.029 | 0.021-0.037 | 1.00E-13 |
| MVPA | rs2668196 | Serum levels of protein BCHE | 35078996 | chr3:165502709 | A=0.191, T=0.809 | 0.1441 | 0.301503 | 0.25-0.35 | 1.00E-36 |
| MVPA | rs2668196 | Highest math class taken (MTAG) | 30038396 | chr3:165502709 | A=0.191, T=0.809 | 0.1947 | 0.0127 | 0.0086-0.0168 | 1.00E-09 |
| MVPA | rs2668196 | Kinetochore protein NDC80 homolog levels (NDC80.12730.3.3) | 29875488 | chr3:165502709 | A=0.191, T=0.809 | 0.817 | 0.23 | 0.12 | 5.00E-13 |
| MVPA | rs2668196 | Educational attainment (MTAG) | 30038396 | chr3:165502709 | A=0.191, T=0.809 | 0.1904 | 0.0106 | 0.0073-0.0139 | 2.00E-10 |
| MVPA | rs2668196 | Educational attainment | 35361970 | chr3:165502709 | A=0.191, T=0.809 | 0.1939 | 0.011857 | 0.0093-0.0145 | 3.00E-19 |
| MVPA | rs2668196 | Cholinesterase levels | 34648354 | chr3:165502709 | A=0.191, T=0.809 | 0.19 | 0.529 | 0.5-0.56 | 1.00E-251 |
| MVPA | rs2668196 | Carnitine O-palmitoyltransferase 1, muscle isoform levels | 34648354 | chr3:165502709 | A=0.191, T=0.809 | 0.19 | 0.284 | 0.25-0.32 | 4.00E-67 |
| MVPA | rs2668196 | Short-chain specific acyl-CoA dehydrogenase, mitochondrial levels | 34648354 | chr3:165502709 | A=0.191, T=0.809 | 0.19 | 0.19 | 0.16-0.22 | 6.00E-29 |
| MVPA | rs7613360 | Feeling miserable | 29500382 | chr3:49916710 | C=0.616, T=0.384 | 0.386537 | 5.61 | NA | 2.00E-08 |
| MVPA | rs7613360 | Depressed affect | 29942085 | chr3:49916710 | C=0.616, T=0.384 | NR | 0.01326 | 0.0086-0.0179 | 2.00E-08 |
| MVPA | rs7613360 | Intelligence | 29942086 | chr3:49916710 | C=0.616, T=0.384 | NR | 8.072 | - | 7.00E-16 |
| MVPA | rs7613360 | General cognitive ability | 29844566 | chr3:49916710 | C=0.616, T=0.384 | NR | 6.517 | - | 7.00E-11 |
| MVPA | rs7613360 | Noncognitive aspects of educational attainment | 33414549 | chr3:49916710 | C=0.616, T=0.384 | 0.383698 | 0.055265676 | 0.041-0.069 | 3.00E-14 |
| MVPA | rs7613360 | Insomnia | 35835914 | chr3:49916710 | C=0.616, T=0.384 | NR | 0.007 | 0.005-0.009 | 3.00E-14 |
| MVPA | rs7613360 | Sedentary behavior at work | 36071172 | chr3:49916710 | C=0.616, T=0.384 | 0.605 | 0.031 | 0.019-0.043 | 3.00E-08 |
| MVPA | rs7613360 | Moderate-to-vigorous intensity physical activity during leisure time | 36071172 | chr3:49916710 | C=0.616, T=0.384 | 0.604 | 0.025 | 0.017-0.033 | 3.00E-09 |
| MVPA | rs7613360 | Leisure screen time | 36071172 | chr3:49916710 | C=0.616, T=0.384 | 0.604 | 0.032 | 0.024-0.04 | 7.00E-16 |
| MVPA | rs7613360 | Disruptive behavior (multivariate analysis) | 36324656 | chr3:49916710 | C=0.616, T=0.384 | NR | NA | NA | 8.00E-09 |
| MVPA | rs7613360 | School performance E-factor 1 (overall performance) | 36624241 | chr3:49916710 | C=0.616, T=0.384 | NR | 0.045 | 0.031-0.059 | 4.00E-09 |
| MVPA | rs7613360 | Smoking cessation | 36477530 | chr3:49916710 | C=0.616, T=0.384 | 0.387 | 0.0129 | 0.01-0.016 | 2.00E-21 |
| MVPA | rs7613360 | Attention deficit hyperactivity disorder or autism spectrum disorder or intelligence (pleiotropy) | 35764056 | chr3:49916710 | C=0.616, T=0.384 | NR | NA | NA | 5.00E-23 |
| MVPA | rs7613360 | Smoking cessation | 36477530 | chr3:49916710 | C=0.616, T=0.384 | 0.359 | 0.0119 | 0.0095-0.0144 | 5.00E-22 |
| MVPA | rs7613360 | Attention deficit hyperactivity disorder or risk-taking behavior (pleiotropy) | 38565336 | chr3:49916710 | C=0.616, T=0.384 | NR | NA | NA | 3.00E-08 |
| MVPA | rs7613360 | Gastroesophageal reflux disease or attention-deficit/hyperactivity disorder (pleiotropy) | 36753304 | chr3:49916710 | C=0.616, T=0.384 | NR | NA | NA | 4.00E-08 |
| MVPA | rs1691471 | Moderate-to-vigorous intensity physical activity during leisure time | 36071172 | chr3:85011013 | C=0.599, T=0.401 | 0.376 | 0.038 | 0.03-0.046 | 2.00E-19 |
| MVPA | rs4865512 | General risk tolerance (MTAG) | 30643258 | chr5:50661601 | A=0.642, G=0.358 | 0.616 | 0.009 | 0.0066-0.0114 | 9.00E-14 |
| MVPA | rs4352559 | Waist-to-hip ratio adjusted for BMI | 34021172 | chr5:60586625 | C=0.492, T=0.508 | NR | 0.0169573 | 0.011-0.023 | 2.00E-09 |
| MVPA | rs4352559 | Waist-hip index | 34021172 | chr5:60586625 | C=0.492, T=0.508 | NR | 0.0171973 | 0.012-0.023 | 1.00E-09 |
| MVPA | rs4352559 | Leisure screen time (MTAG) | 36071172 | chr5:60586625 | C=0.492, T=0.508 | NR | 0.018 | 0.012-0.024 | 1.00E-09 |
| MVPA | rs4352559 | Moderate-to-vigorous intensity physical activity during leisure time (MTAG) | 36071172 | chr5:60586625 | C=0.492, T=0.508 | NR | 0.018 | 0.012-0.024 | 1.00E-09 |
| MVPA | rs13201721 | Moderate-to-vigorous intensity physical activity during leisure time | 36071172 | chr6:141799534 | C=0.265, T=0.735 | 0.736 | 0.026 | 0.018-0.034 | 2.00E-10 |
| SDC | rs112560164 | Post bronchodilator FEV1/FVC ratio | 26634245 | chr14:93112924 | A=0.185, G=0.815 | 0.163 | 0.013 | NA | 2.00E-07 |
| SDC | rs112560164 | Plateletcrit | 27863252 | chr14:93112924 | A=0.185, G=0.815 | 0.1912 | 0.03535423 | 0.026-0.044 | 3.00E-14 |
| SDC | rs112560164 | Monocyte count | 34594039 | chr14:93112924 | A=0.185, G=0.815 | NR | 0.0263 | 0.021-0.032 | 5.00E-19 |
| SDC | rs1229984 | Body mass index | 28892062 | chr4:100239319 | C=0.971, T=0.029 | 0.27 | 0.034 | 0.022-0.046 | 1.00E-07 |
| SDC | rs1229984 | Alcohol dependence | 24166409 | chr4:100239319 | C=0.971, T=0.029 | 0.071 | 0.0164 | (,) | 2.00E-23 |
| SDC | rs1229984 | Alcohol dependence | 24166409 | chr4:100239319 | C=0.971, T=0.029 | NR | NA | NA | 2.00E-22 |
| SDC | rs1229984 | Maximum habitual alcohol consumption | 31151762 | chr4:100239319 | C=0.971, T=0.029 | NR | 14.821 | NA | 1.00E-49 |
| SDC | rs1229984 | Maximum habitual alcohol consumption | 31151762 | chr4:100239319 | C=0.971, T=0.029 | NR | 0.3553 | NA | 5.00E-47 |
| SDC | rs1229984 | Blood urea nitrogen levels | 31152163 | chr4:100239319 | C=0.971, T=0.029 | 0.571 | 0.0079 | 0.0052-0.0106 | 1.00E-08 |
| SDC | rs1229984 | Alcohol consumption (drinks per week) | 30679032 | chr4:100239319 | C=0.971, T=0.029 | 0.98 | 0.246 | 0.23-0.26 | 2.00E-173 |
| SDC | rs1229984 | Body mass index | 30595370 | chr4:100239319 | C=0.971, T=0.029 | NR | NA | NA | 1.00E-12 |
| SDC | rs1229984 | Oral cavity and pharyngeal cancer | 21437268 | chr4:100239319 | C=0.971, T=0.029 | 0.94 | 1.56 | 1.41-1.69 | 1.00E-20 |
| SDC | rs1229984 | Alcohol dependence | 23456092 | chr4:100239319 | C=0.971, T=0.029 | NR | 2.35 | 1.95-2.84 | 3.00E-21 |
| SDC | rs1229984 | High light scatter reticulocyte count | 32888494 | chr4:100239319 | C=0.971, T=0.029 | 0.9747907 | 0.048780907 | 0.035-0.063 | 6.00E-12 |
| SDC | rs1229984 | High light scatter reticulocyte percentage of red cells | 32888494 | chr4:100239319 | C=0.971, T=0.029 | 0.9747934 | 0.04492588 | 0.031-0.059 | 2.00E-10 |
| SDC | rs1229984 | Systolic blood pressure | 30595370 | chr4:100239319 | C=0.971, T=0.029 | NR | NA | NA | 6.00E-13 |
| SDC | rs1229984 | Alcohol use disorder | 32451486 | chr4:100239319 | C=0.971, T=0.029 | 0.0302 | 22 | NA | 3.00E-107 |
| SDC | rs1229984 | Problematic alcohol use (MTAG) | 32451486 | chr4:100239319 | C=0.971, T=0.029 | 0.9698 | 0.24346012 | 0.23-0.26 | 6.00E-267 |
| SDC | rs1229984 | Alcohol consumption (drinks per week) (MTAG) | 32451486 | chr4:100239319 | C=0.971, T=0.029 | 0.953 | 0.15401822 | 0.15-0.16 | 7.00E-280 |
| SDC | rs1229984 | Problematic alcohol use | 32451486 | chr4:100239319 | C=0.971, T=0.029 | 0.0302 | 22 | NA | 3.00E-107 |
| SDC | rs1229984 | Esophageal cancer | 32514122 | chr4:100239319 | C=0.971, T=0.029 | 0.261191323 | 2.0655997 | 1.865488484-2.287176659 | 3.00E-44 |
| SDC | rs1229984 | Immature fraction of reticulocytes | 32888494 | chr4:100239319 | C=0.971, T=0.029 | 0.9748287 | 0.046019696 | 0.032-0.06 | 1.00E-10 |
| SDC | rs1229984 | Cardiovascular disease | 30595370 | chr4:100239319 | C=0.971, T=0.029 | NR | NA | NA | 8.00E-15 |
| SDC | rs1229984 | LDL cholesterol levels | 32203549 | chr4:100239319 | C=0.971, T=0.029 | 0.0272003 | 0.0528485 | 0.04-0.065 | 7.00E-17 |
| SDC | rs1229984 | Relative fat intake | 32393786 | chr4:100239319 | C=0.971, T=0.029 | NR | 0.098 | 0.08-0.116 | 1.00E-28 |
| SDC | rs1229984 | Relative protein intake | 32393786 | chr4:100239319 | C=0.971, T=0.029 | NR | 0.044 | 0.026-0.062 | 9.00E-07 |
| SDC | rs1229984 | Apolipoprotein B levels | 32203549 | chr4:100239319 | C=0.971, T=0.029 | 0.0271895 | 0.0388703 | 0.026-0.051 | 8.00E-10 |
| SDC | rs1229984 | Alcohol consumption (heavy vs. light/non-drinkers) | 31998841 | chr4:100239319 | C=0.971, T=0.029 | 0.98 | 17.209 | NA | 2.00E-66 |
| SDC | rs1229984 | Alcohol consumption (drinks per week) | 31959922 | chr4:100239319 | C=0.971, T=0.029 | NR | 0.07 | 0.055-0.085 | 4.00E-19 |
| SDC | rs1229984 | Serum 25-Hydroxyvitamin D levels | 32059762 | chr4:100239319 | C=0.971, T=0.029 | 0.973099 | 0.046854 | 0.034-0.06 | 5.00E-13 |
| SDC | rs1229984 | Alcohol consumption (drinkers vs non-drinkers) | 31959922 | chr4:100239319 | C=0.971, T=0.029 | NR | 0.038 | 0.034-0.042 | 2.00E-72 |
| SDC | rs1229984 | Esophageal cancer | 19698717 | chr4:100239319 | C=0.971, T=0.029 | NR | 1.79 | 1.69-1.88 | 8.00E-24 |
| SDC | rs1229984 | Alcohol consumption (drinks per week) | 28485404 | chr4:100239319 | C=0.971, T=0.029 | 0.05 | 0.19 | 0.22-0.16 | 3.00E-32 |
| SDC | rs1229984 | Alcohol consumption (drinks per week) | 28485404 | chr4:100239319 | C=0.971, T=0.029 | 0.05 | 0.21 | 0.30-0.12 | 3.00E-06 |
| SDC | rs1229984 | Alcohol consumption (drinkers vs non-drinkers) | 28485404 | chr4:100239319 | C=0.971, T=0.029 | 0.95 | 1.27 | 1.19-1.35 | 2.00E-20 |
| SDC | rs1229984 | Alcohol consumption (drinkers vs non-drinkers) | 28485404 | chr4:100239319 | C=0.971, T=0.029 | 0.93 | 1.39 | 1.18-1.69 | 4.00E-07 |
| SDC | rs1229984 | Mean corpuscular hemoglobin | 30595370 | chr4:100239319 | C=0.971, T=0.029 | NR | NA | NA | 2.00E-18 |
| SDC | rs1229984 | Low density lipoprotein cholesterol levels | 32154731 | chr4:100239319 | C=0.971, T=0.029 | NR | 0.0804 | 0.065-0.095 | 1.00E-20 |
| SDC | rs1229984 | Red blood cell count | 30595370 | chr4:100239319 | C=0.971, T=0.029 | NR | NA | NA | 7.00E-12 |
| SDC | rs1229984 | Pulse pressure | 28135244 | chr4:100239319 | C=0.971, T=0.029 | 0.9744467 | 0.465 | 0.28-0.65 | 5.00E-07 |
| SDC | rs1229984 | Alcohol consumption | 23743675 | chr4:100239319 | C=0.971, T=0.029 | 0.03 | NA | NA | 2.00E-08 |
| SDC | rs1229984 | Serum 25-Hydroxyvitamin D levels | 32242144 | chr4:100239319 | C=0.971, T=0.029 | 0.0248886 | 0.039 | 0.027-0.051 | 5.00E-10 |
| SDC | rs1229984 | Gout | 33832965 | chr4:100239319 | C=0.971, T=0.029 | 0.02 | 1.75 | 1.57-1.95 | 5.00E-23 |
| SDC | rs1229984 | Gout vs asymptomatic hyperuricemia | 33832965 | chr4:100239319 | C=0.971, T=0.029 | 0.03 | 1.49 | 1.35-1.64 | 1.00E-15 |
| SDC | rs1229984 | Aerodigestive squamous cell cancer (pleiotropy) | 33667223 | chr4:100239319 | C=0.971, T=0.029 | 0.05 | 0.8 | 0.72-0.88 | 2.00E-09 |
| SDC | rs1229984 | Alcohol use disorder (consumption score) | 30336701 | chr4:100239319 | C=0.971, T=0.029 | NR | 0.05 | 0.044-0.056 | 4.00E-56 |
| SDC | rs1229984 | Alcohol use disorder (dependence and problematic use scores) | 30336701 | chr4:100239319 | C=0.971, T=0.029 | NR | 0.04 | NA | 1.00E-45 |
| SDC | rs1229984 | Total testosterone levels | 32042192 | chr4:100239319 | C=0.971, T=0.029 | 0.9763163 | 0.0489634 | 0.031-0.067 | 1.00E-08 |
| SDC | rs1229984 | Alcohol use disorder (consumption score) | 33861876 | chr4:100239319 | C=0.971, T=0.029 | NR | 0.592 | 0.52-0.66 | 2.00E-65 |
| SDC | rs1229984 | Daily alcohol intake in rs671 GG genotype | 38277453 | chr4:100239319 | C=0.971, T=0.029 | NR | 0.134 | 0.11-0.15 | 6.00E-39 |
| SDC | rs1229984 | Alcohol drinking status in rs671 GG genotype | 38277453 | chr4:100239319 | C=0.971, T=0.029 | NR | 1.14 | 1.11-1.17 | 2.00E-24 |
| SDC | rs1229984 | Alcohol drinking status (ever drinker vs never drinker) | 38277453 | chr4:100239319 | C=0.971, T=0.029 | NR | 1.18 | 1.16-1.2 | 2.00E-73 |
| SDC | rs1229984 | Daily alcohol intake in rs671 GA genotype | 38277453 | chr4:100239319 | C=0.971, T=0.029 | NR | 0.243 | 0.22-0.26 | 3.00E-101 |
| SDC | rs1229984 | Alcohol drinking status in rs671 GA genotype | 38277453 | chr4:100239319 | C=0.971, T=0.029 | NR | 1.37 | 1.33-1.41 | 4.00E-86 |
| SDC | rs1229984 | Daily alcohol intake x rs671 interaction (2df) | 38277453 | chr4:100239319 | C=0.971, T=0.029 | NR | NA | NA | 3.00E-95 |
| SDC | rs1229984 | Alcohol drinking status x rs671 interaction (2df) | 38277453 | chr4:100239319 | C=0.971, T=0.029 | NR | NA | NA | 7.00E-100 |
| SDC | rs1229984 | Daily alcohol intake | 38277453 | chr4:100239319 | C=0.971, T=0.029 | NR | 0.168 | 0.15-0.18 | 6.00E-91 |
| SDC | rs1229984 | Oropharynx cancer and human papilloma virus 16 negative oropharyngeal cancer | 34642315 | chr4:100239319 | C=0.971, T=0.029 | NR | 0.5916 | NA | 4.00E-08 |
| SDC | rs1229984 | Esophageal cancer | 34594039 | chr4:100239319 | C=0.971, T=0.029 | NR | 0.6715 | 0.58-0.76 | 3.00E-45 |
| SDC | rs1229984 | Esophageal cancer | 34594039 | chr4:100239319 | C=0.971, T=0.029 | 0.244615766 | 0.71232766 | 0.61-0.81 | 2.00E-46 |
| SDC | rs1229984 | Ratio of saturated fatty acids to total fatty acids | 35213538 | chr4:100239319 | C=0.971, T=0.029 | 0.0274594 | 0.145346 | 0.12-0.17 | 4.00E-32 |
| SDC | rs1229984 | Body mass index | 34594039 | chr4:100239319 | C=0.971, T=0.029 | NR | 0.0292 | 0.022-0.036 | 5.00E-17 |
| SDC | rs1229984 | Blood urea nitrogen levels | 34594039 | chr4:100239319 | C=0.971, T=0.029 | NR | 0.0296 | 0.023-0.037 | 3.00E-16 |
| SDC | rs1229984 | Estimated glomerular filtration rate (creatinine) | 35710981 | chr4:100239319 | C=0.971, T=0.029 | 0.756 | 5.968 | NA | 2.00E-09 |
| SDC | rs1229984 | Weight | 34594039 | chr4:100239319 | C=0.971, T=0.029 | NR | 0.0185 | 0.013-0.024 | 5.00E-10 |
| SDC | rs1229984 | Alcohol use disorder | 35094024 | chr4:100239319 | C=0.971, T=0.029 | NR | NA | NA | 3.00E-17 |
| SDC | rs1229984 | Gamma glutamyl transpeptidase | 34594039 | chr4:100239319 | C=0.971, T=0.029 | NR | 0.0225 | 0.015-0.03 | 1.00E-09 |
| SDC | rs1229984 | Adult body size | 32376654 | chr4:100239319 | C=0.971, T=0.029 | 0.0271609 | 0.02304 | 0.015-0.031 | 3.00E-08 |
| SDC | rs1229984 | Alcohol use disorder (consumption score) | 30940813 | chr4:100239319 | C=0.971, T=0.029 | 0.97 | 0.3402 | 0.31-0.37 | 5.00E-102 |
| SDC | rs1229984 | Alcohol use disorder | 30940813 | chr4:100239319 | C=0.971, T=0.029 | 0.97 | 1.742 | 1.68-1.8 | 5.00E-74 |
| SDC | rs1229984 | Alcohol use disorder (consumption score) | 30940813 | chr4:100239319 | C=0.971, T=0.029 | 0.97 | 24.56 | NA | 4.00E-133 |
| SDC | rs1229984 | Alcohol use disorder (consumption score) | 30940813 | chr4:100239319 | C=0.971, T=0.029 | 0.989 | 0.412 | 0.32-0.5 | 1.00E-19 |
| SDC | rs1229984 | Alcohol use disorder (consumption score) | 30940813 | chr4:100239319 | C=0.971, T=0.029 | 0.938 | 0.349 | 0.26-0.43 | 4.00E-16 |
| SDC | rs1229984 | Alcohol use disorder | 30940813 | chr4:100239319 | C=0.971, T=0.029 | 0.938 | 1.7746 | 1.64-1.91 | 6.00E-17 |
| SDC | rs1229984 | Alcohol use disorder | 30940813 | chr4:100239319 | C=0.971, T=0.029 | 0.969 | 19.54 | NA | 5.00E-85 |
| SDC | rs1229984 | Bitter alcoholic beverage consumption | 31046077 | chr4:100239319 | C=0.971, T=0.029 | 0.9725 | 0.0821 | 0.076-0.089 | 2.00E-140 |
| SDC | rs1229984 | Reticulocyte count | 32888494 | chr4:100239319 | C=0.971, T=0.029 | 0.9748243 | 0.042106487 | 0.028-0.056 | 4.00E-09 |
| SDC | rs1229984 | Alcohol consumption (drinks per week) | 30643251 | chr4:100239319 | C=0.971, T=0.029 | 0.963 | 0.15053357 | 0.14-0.16 | 2e-308 |
| SDC | rs1229984 | Serum total protein levels | 33462484 | chr4:100239319 | C=0.971, T=0.029 | NR | 0.0697 | 0.054-0.086 | 1.00E-17 |
| SDC | rs1229984 | Alanine aminotransferase levels | 35810165 | chr4:100239319 | C=0.971, T=0.029 | NR | NA | NA | 1.00E-14 |
| SDC | rs1229984 | Urea levels | 33462484 | chr4:100239319 | C=0.971, T=0.029 | NR | 0.0775 | 0.062-0.093 | 2.00E-23 |
| SDC | rs1229984 | Urinary sodium excretion | 33462484 | chr4:100239319 | C=0.971, T=0.029 | NR | 0.0482 | 0.033-0.064 | 1.00E-09 |
| SDC | rs1229984 | Low density lipoprotein cholesterol levels | 33462484 | chr4:100239319 | C=0.971, T=0.029 | NR | 0.0774 | 0.062-0.093 | 3.00E-23 |
| SDC | rs1229984 | Urate levels | 33462484 | chr4:100239319 | C=0.971, T=0.029 | NR | 0.0694 | 0.054-0.085 | 5.00E-19 |
| SDC | rs1229984 | Vitamin D levels | 33462484 | chr4:100239319 | C=0.971, T=0.029 | NR | 0.0469 | 0.031-0.063 | 4.00E-09 |
| SDC | rs1229984 | Phospholipids to total lipids ratio in large LDL | 35213538 | chr4:100239319 | C=0.971, T=0.029 | 0.0274543 | 0.0893003 | 0.065-0.114 | 8.00E-13 |
| SDC | rs1229984 | Cholesteryl esters to total lipids ratio in large LDL | 35213538 | chr4:100239319 | C=0.971, T=0.029 | 0.0274543 | 0.0746017 | 0.05-0.099 | 3.00E-09 |
| SDC | rs1229984 | Oral cavity and pharyngeal cancer | 27749845 | chr4:100239319 | C=0.971, T=0.029 | 0.94 | 1.7898692 | NA | 2.00E-15 |
| SDC | rs1229984 | Oropharynx cancer | 27749845 | chr4:100239319 | C=0.971, T=0.029 | 0.940145546 | 1.832173 | NA | 9.00E-09 |
| SDC | rs1229984 | Oral cavity cancer | 27749845 | chr4:100239319 | C=0.971, T=0.029 | 0.94 | 1.7436792 | NA | 1.00E-09 |
| SDC | rs1229984 | Alcohol dependence | 24166409 | chr4:100239319 | C=0.971, T=0.029 | 0.971 | 6.5789475 | (,) | 1.00E-31 |
| SDC | rs1229984 | Heel bone mineral density | 30598549 | chr4:100239319 | C=0.971, T=0.029 | 0.02372 | 0.0360185 | 0.024-0.048 | 6.00E-09 |
| SDC | rs1229984 | Alcohol consumption (drinks per week) | 30643258 | chr4:100239319 | C=0.971, T=0.029 | 0.0228 | 0.2266165 | 0.21-0.24 | 8.00E-202 |
| SDC | rs1229984 | Heel bone mineral density | 30595370 | chr4:100239319 | C=0.971, T=0.029 | NR | NA | NA | 4.00E-08 |
| SDC | rs1229984 | Regular attendance at a pub or social club | 29970889 | chr4:100239319 | C=0.971, T=0.029 | 0.0236479 | 0.0312757 | 0.025-0.037 | 4.00E-25 |
| SDC | rs1229984 | Urinary sodium excretion | 31409800 | chr4:100239319 | C=0.971, T=0.029 | 0.0265565 | 0.0241902 | 0.017-0.032 | 2.00E-10 |
| SDC | rs1229984 | Predicted visceral adipose tissue | 31501611 | chr4:100239319 | C=0.971, T=0.029 | 0.02239 | 0.049819224 | 0.033-0.066 | 3.00E-09 |
| SDC | rs1229984 | Risk-taking tendency (4-domain principal component model) | 30643258 | chr4:100239319 | C=0.971, T=0.029 | 0.023 | 0.083182804 | 0.066-0.1 | 4.00E-22 |
| SDC | rs1229984 | Serum albumin levels | 33462484 | chr4:100239319 | C=0.971, T=0.029 | NR | 0.0559 | 0.04-0.072 | 6.00E-12 |
| SDC | rs1229984 | Serum alkaline phosphatase levels | 33462484 | chr4:100239319 | C=0.971, T=0.029 | NR | 0.0797 | 0.064-0.095 | 1.00E-24 |
| SDC | rs1229984 | Total cholesterol levels | 33462484 | chr4:100239319 | C=0.971, T=0.029 | NR | 0.0645 | 0.049-0.08 | 1.00E-16 |
| SDC | rs1229984 | Liver enzyme levels (gamma-glutamyl transferase) | 33972514 | chr4:100239319 | C=0.971, T=0.029 | 0.0264666 | 0.012214 | 0.009-0.0154 | 2.00E-14 |
| SDC | rs1229984 | Apolipoprotein B levels | 33462484 | chr4:100239319 | C=0.971, T=0.029 | NR | 0.0567 | 0.041-0.072 | 3.00E-13 |
| SDC | rs1229984 | Insulin-like growth factor 1 levels | 33462484 | chr4:100239319 | C=0.971, T=0.029 | NR | 0.0921 | 0.077-0.107 | 3.00E-32 |
| SDC | rs1229984 | F-wine liking (derived food-liking factor) | 35585065 | chr4:100239319 | C=0.971, T=0.029 | 0.9745 | 0.153736 | 0.13-0.18 | 2.00E-42 |
| SDC | rs1229984 | Lager liking | 35585065 | chr4:100239319 | C=0.971, T=0.029 | 0.9745 | 0.065856 | 0.044-0.088 | 5.00E-09 |
| SDC | rs1229984 | Bitter ale liking | 35585065 | chr4:100239319 | C=0.971, T=0.029 | 0.9745 | 0.065969 | 0.044-0.088 | 5.00E-09 |
| SDC | rs1229984 | Alcohol liking | 35585065 | chr4:100239319 | C=0.971, T=0.029 | 0.9745 | 0.425957 | 0.35-0.5 | 3.00E-27 |
| SDC | rs1229984 | F-coffee/alcohol liking (derived food-liking factor) | 35585065 | chr4:100239319 | C=0.971, T=0.029 | 0.9745 | 0.322925 | 0.24-0.41 | 6.00E-14 |
| SDC | rs1229984 | Alcohol dependence | 31090166 | chr4:100239319 | C=0.971, T=0.029 | NR | NA | NA | 5.00E-09 |
| SDC | rs1229984 | Alcohol dependence symptom count | 31090166 | chr4:100239319 | C=0.971, T=0.029 | NR | NA | NA | 4.00E-10 |
| SDC | rs1229984 | Alcohol dependence (tolerance) | 31090166 | chr4:100239319 | C=0.971, T=0.029 | NR | NA | NA | 1.00E-09 |
| SDC | rs1229984 | Alcohol dependence (desire to cut drinking) | 31090166 | chr4:100239319 | C=0.971, T=0.029 | NR | NA | NA | 1.00E-17 |
| SDC | rs1229984 | Risk-taking behavior (multivariate analysis) | 36324656 | chr4:100239319 | C=0.971, T=0.029 | NR | NA | NA | 5.00E-12 |
| SDC | rs1229984 | Relative fat intake | 34426670 | chr4:100239319 | C=0.971, T=0.029 | 0.0445 | 0.5206 | 0.42-0.62 | 1.00E-22 |
| SDC | rs1229984 | Relative protein intake | 34426670 | chr4:100239319 | C=0.971, T=0.029 | 0.5963 | 0.0728 | 0.054-0.092 | 3.00E-14 |
| SDC | rs1229984 | Alcohol consumption (drinks per month) (UKB data field 1578, 4424) | 32193382 | chr4:100239319 | C=0.971, T=0.029 | 0.025062 | 0.226432 | 0.21-0.24 | 4.00E-248 |
| SDC | rs1229984 | X-12127 levels | 36357675 | chr4:100239319 | C=0.971, T=0.029 | 0.0312 | 0.2193142 | 0.16-0.28 | 1.00E-13 |
| SDC | rs1229984 | Maximum habitual alcohol consumption (MTAG) | 36301540 | chr4:100239319 | C=0.971, T=0.029 | NR | 0.19434102 | 0.18-0.21 | 3.00E-176 |
| SDC | rs1229984 | Problematic alcohol use (MTAG) | 36301540 | chr4:100239319 | C=0.971, T=0.029 | NR | 0.2214 | 0.21-0.24 | 2.00E-182 |
| SDC | rs1229984 | Maximum habitual alcohol consumption | 36301540 | chr4:100239319 | C=0.971, T=0.029 | NR | NA | NA | 2.00E-101 |
| SDC | rs1229984 | Maximum habitual alcohol consumption | 36301540 | chr4:100239319 | C=0.971, T=0.029 | NR | NA | NA | 3.00E-101 |
| SDC | rs1229984 | Ideal cardiovascular health score (clinical and behavioral) | 35613103 | chr4:100239319 | C=0.971, T=0.029 | 0.96 | 0.1 | NA | 2.00E-09 |
| SDC | rs1229984 | X-24422 levels | 36357675 | chr4:100239319 | C=0.971, T=0.029 | 0.031 | 0.34922126 | 0.29-0.41 | 6.00E-31 |
| SDC | rs1229984 | Drinks per week | 36477530 | chr4:100239319 | C=0.971, T=0.029 | 0.9273 | 0.139142 | 0.13-0.15 | 2.00E-157 |
| SDC | rs1229984 | Drinks per week | 36477530 | chr4:100239319 | C=0.971, T=0.029 | 0.2878 | 0.0653 | 0.058-0.073 | 5.00E-63 |
| SDC | rs1229984 | Drinks per week | 36477530 | chr4:100239319 | C=0.971, T=0.029 | 0.973 | 0.193 | 0.18-0.2 | 6.00E-284 |
| SDC | rs1229984 | C-reactive protein levels (MTAG) | 36376304 | chr4:100239319 | C=0.971, T=0.029 | NR | 0.03207 | 0.021-0.043 | 3.00E-08 |
| SDC | rs1229984 | Non-HDL cholesterol levels | 34887591 | chr4:100239319 | C=0.971, T=0.029 | NR | NA | NA | 3.00E-14 |
| SDC | rs1229984 | Body composition (MOSTest) | 36402844 | chr4:100239319 | C=0.971, T=0.029 | NR | 5.98 | NA | 2.00E-09 |
| SDC | rs1229984 | Liver proton density fat fraction | 36402844 | chr4:100239319 | C=0.971, T=0.029 | 0.03 | 7.45 | NA | 9.00E-14 |
| SDC | rs1229984 | Drinks per week | 36477530 | chr4:100239319 | C=0.971, T=0.029 | 0.87 | 0.0822 | 0.078-0.086 | 4.94065645841247e-324 |
| SDC | rs1229984 | Nonalcoholic fatty liver disease | 37709864 | chr4:100239319 | C=0.971, T=0.029 | 0.95 | 6.56 | NA | 6.00E-11 |
| SDC | rs1229984 | Problematic alcohol use | 37250466 | chr4:100239319 | C=0.971, T=0.029 | NR | NA | NA | 4.00E-68 |
| SDC | rs1229984 | Gamma glutamyl transferase levels | 38632349 | chr4:100239319 | C=0.971, T=0.029 | NR | 0.0132 | 0.01-0.016 | 3.00E-16 |
| SDC | rs1229984 | LDL (standard GWA) | 37106081 | chr4:100239319 | C=0.971, T=0.029 | NR | 0.046518 | 0.031-0.062 | 3.00E-09 |
| SDC | rs1229984 | Oropharyngeal or hypopharyngeal squamous cell carcinoma | 37706329 | chr4:100239319 | C=0.971, T=0.029 | NR | 1.8277 | 1.54676-2.15966 | 1.00E-12 |
| SDC | rs1229984 | Serum urate levels | 38658550 | chr4:100239319 | C=0.971, T=0.029 | NR | 0.0625 | 0.045-0.08 | 7.00E-12 |
| SDC | rs1229984 | Systolic blood pressure | 38689001 | chr4:100239319 | C=0.971, T=0.029 | NR | 0.5988 | 0.42-0.77 | 2.00E-11 |
| SDC | rs1229984 | Pulse pressure | 38689001 | chr4:100239319 | C=0.971, T=0.029 | NR | 0.5111 | 0.39-0.63 | 4.00E-17 |
| SDC | rs1229984 | Ratio of saturated fatty acids to total fatty acids | 38448586 | chr4:100239319 | C=0.971, T=0.029 | 0.1986 | 0.0804 | 0.058-0.102 | 8.00E-13 |
| SDC | rs1229984 | Pulse pressure | 33230300 | chr4:100239319 | C=0.971, T=0.029 | 0.0258 | 0.756563 | 0.61-0.9 | 3.00E-24 |
| SDC | rs1229984 | Systolic blood pressure | 33230300 | chr4:100239319 | C=0.971, T=0.029 | 0.0258 | 0.8607134 | 0.64-1.08 | 9.00E-15 |
| SDC | rs1229984 | Pulse pressure | 33230300 | chr4:100239319 | C=0.971, T=0.029 | 0.0254 | 0.7580625 | 0.61-0.9 | 3.00E-25 |
| SDC | rs1229984 | Saturated fatty acids to total fatty acids percentage (UKB data field 23455) | 36764567 | chr4:100239319 | C=0.971, T=0.029 | 0.0267934 | 0.134744 | 0.11-0.16 | 2.00E-20 |
| SDC | rs1229984 | Body mass index | 36581621 | chr4:100239319 | C=0.971, T=0.029 | NR | 0.0391 | 0.03-0.049 | 3.00E-16 |
| SDC | rs1229984 | Body mass index (MTAG) | 36376304 | chr4:100239319 | C=0.971, T=0.029 | NR | 0.03446 | 0.023-0.046 | 6.00E-09 |
| SDC | rs1229984 | Systolic blood pressure | 33230300 | chr4:100239319 | C=0.971, T=0.029 | 0.0255 | 0.88558203 | 0.67-1.1 | 6.00E-16 |
| SDC | rs1229984 | Multi-trait sum score | 37277458 | chr4:100239319 | C=0.971, T=0.029 | NR | NA | NA | 2.00E-08 |
| SDC | rs1229984 | Phospholipids to total lipids in large LDL percentage (UKB data field 23614) | 36764567 | chr4:100239319 | C=0.971, T=0.029 | 0.0267919 | 0.099785 | 0.071-0.129 | 1.00E-11 |
| SDC | rs1229984 | Alcohol use disorder | 37282553 | chr4:100239319 | C=0.971, T=0.029 | 0.0338 | 27.11 | - | 7.00E-162 |
| SDC | rs1229984 | Alcohol use disorder | 37282553 | chr4:100239319 | C=0.971, T=0.029 | 0.03662 | 0.5576 | 0.51-0.6 | 1.00E-131 |
| SDC | rs1229984 | Alcohol use disorder | 37282553 | chr4:100239319 | C=0.971, T=0.029 | 0.0654 | 0.5666 | 0.48-0.65 | 5.00E-37 |
| SDC | rs1229984 | Alcohol use disorder (consumption score) | 37282553 | chr4:100239319 | C=0.971, T=0.029 | 0.0337 | 32.299 | NA | 7.00E-229 |
| SDC | rs1229984 | Alcohol use disorder (consumption score) | 37282553 | chr4:100239319 | C=0.971, T=0.029 | 0.03662 | 0.5775 | 0.54-0.62 | 7.00E-173 |
| SDC | rs1229984 | Alcohol use disorder (consumption score) | 37282553 | chr4:100239319 | C=0.971, T=0.029 | 0.0654 | 0.7686 | 0.67-0.87 | 3.00E-50 |
| SDC | rs1229984 | Body mass index | 37280435 | chr4:100239319 | C=0.971, T=0.029 | NR | 0.0400103 | 0.028-0.052 | 2.00E-11 |
| SDC | rs1229984 | Metabolite levels (octadecanedioate; octadecanedioate (C18-DC)) | 37253714 | chr4:100239319 | C=0.971, T=0.029 | 0.9588 | 0.267 | 0.19-0.34 | 3.00E-12 |
| SDC | rs1229984 | Metabolite levels (hexadecanedioate; hexadecanedioate (C16-DC)) | 37253714 | chr4:100239319 | C=0.971, T=0.029 | 0.9587 | 0.252 | 0.18-0.32 | 1.00E-11 |
| SDW | rs8450 | Sedentary behavior at work | 36071172 | chr1:153920286 | A=0.291, G=0.709 | 0.305 | 0.032 | 0.022-0.042 | 2.00E-10 |
| SDW | rs79248502 | Educational attainment | 35361970 | chr5:111012600 | C=0.94, G=0.06 | 0.944 | 0.0118431 | 0.0074-0.0163 | 2.00E-07 |
| SDW | rs4518438 | Sedentary behavior at work | 36071172 | chr5:88157552 | C=0.525, T=0.475 | 0.532 | 0.026 | 0.018-0.034 | 4.00E-10 |
| SDW | rs4596363 | Sedentary behavior at work | 36071172 | chr5:92596445 | A=0.291, G=0.709 | 0.313 | 0.034 | 0.022-0.046 | 4.00E-09 |
| SDW | rs9482120 | Sedentary behavior at work | 36071172 | chr6:98392667 | A=0.587, C=0.413 | 0.603 | 0.054 | 0.042-0.066 | 3.00E-21 |
| SmkInit | rs10786721 | Number of sexual partners | 30643258 | chr10:104654383 | A=0.417, C=0.583 | 0.3924 | 0.014717075 | 0.01-0.0195 | 1.00E-09 |
| SmkInit | rs10786721 | Smoking initiation | 36477530 | chr10:104654383 | A=0.417, C=0.583 | 0.408 | 0.0151 | 0.013-0.017 | 2.00E-64 |
| SmkInit | rs1291865 | Educational attainment | 35361970 | chr10:11082192 | G=0.468, T=0.532 | 0.5105 | 0.0103137 | 0.0083-0.0124 | 7.00E-23 |
| SmkInit | rs4751614 | Inferior parietal cortex volume | 31530798 | chr10:118696266 | A=0.768, T=0.232 | NR | NA | NA | 1.00E-06 |
| SmkInit | rs4751614 | Cortical surface area | 32193296 | chr10:118696266 | A=0.768, T=0.232 | 0.7629 | 20.74429 | 15.06-26.43 | 9.00E-13 |
| SmkInit | rs4751614 | Age of smoking initiation (MTAG) | 30643251 | chr10:118696266 | A=0.768, T=0.232 | 0.235 | 0.010499 | 0.0071-0.0139 | 1.00E-09 |
| SmkInit | rs4751614 | Left-hemisphere limbic network to right-hemisphere dorsal attention network white-matter structural connectivity | 38438384 | chr10:118696266 | A=0.768, T=0.232 | NR | 0.0502751 | 0.033-0.068 | 1.00E-08 |
| SmkInit | rs9423279 | Smoking initiation (ever regular vs never regular) | 30679032 | chr10:125680419 | C=0.363, G=0.637 | 0.657 | 0.0139 | 0.0092-0.0186 | 8.00E-09 |
| SmkInit | rs9423279 | Smoking status | 30595370 | chr10:125680419 | C=0.363, G=0.637 | NR | NA | NA | 2.00E-11 |
| SmkInit | rs9423279 | Smoking initiation (ever regular vs never regular) (MTAG) | 30643251 | chr10:125680419 | C=0.363, G=0.637 | 0.645 | 0.0102363 | 0.0079-0.0126 | 2.00E-17 |
| SmkInit | rs9423279 | Age of smoking initiation (MTAG) | 30643251 | chr10:125680419 | C=0.363, G=0.637 | 0.637 | 0.0101094 | 0.007-0.0132 | 2.00E-10 |
| SmkInit | rs9423279 | Smoking initiation (ever regular vs never regular) | 30643251 | chr10:125680419 | C=0.363, G=0.637 | 0.645 | 0.018580785 | 0.013-0.024 | 3.00E-12 |
| SmkInit | rs9423279 | Smoking status (ever vs never smokers) | 30643258 | chr10:125680419 | C=0.363, G=0.637 | 0.3433 | 0.015488954 | 0.011-0.02 | 1.00E-11 |
| SmkInit | rs9423279 | Smoking initiation | 36477530 | chr10:125680419 | C=0.363, G=0.637 | 0.649 | 0.0111 | 0.0093-0.0129 | 3.00E-34 |
| SmkInit | rs9423279 | Smoking initiation | 36477530 | chr10:125680419 | C=0.363, G=0.637 | 0.652 | 0.0103 | 0.0086-0.012 | 7.00E-33 |
| SmkInit | rs11258417 | Smoking initiation (ever regular vs never regular) | 30643251 | chr10:13533053 | C=0.664, T=0.336 | 0.391 | 0.014513622 | 0.0094-0.0196 | 3.00E-08 |
| SmkInit | rs11258417 | Smoking initiation | 36477530 | chr10:13533053 | C=0.664, T=0.336 | 0.383 | 0.00763 | 0.0059-0.0094 | 1.00E-17 |
| SmkInit | rs11258417 | Smoking initiation | 36477530 | chr10:13533053 | C=0.664, T=0.336 | 0.351 | 0.00693 | 0.0054-0.0085 | 3.00E-17 |
| SmkInit | rs11012726 | Gall stone disease or coronary artery disease | 37705021 | chr10:21797272 | C=0.33, T=0.67 | NR | NA | NA | 9.00E-10 |
| SmkInit | rs1776631 | Smoking initiation (ever regular vs never regular) (MTAG) | 30643251 | chr10:31422577 | C=0.775, T=0.225 | 0.782 | 0.00715359 | 0.0046-0.0097 | 4.00E-08 |
| SmkInit | rs13377168 | Smoking initiation | 36477530 | chr10:63675271 | A=0.491, T=0.509 | 0.467 | 0.0116 | 0.01-0.013 | 6.00E-54 |
| SmkInit | rs540860 | Smoking initiation (ever regular vs never regular) | 30643251 | chr11:121530888 | A=0.437, G=0.563 | 0.543 | 0.017608598 | 0.013-0.023 | 6.00E-12 |
| SmkInit | rs71491832 | Smoking initiation (ever regular vs never regular) (MTAG) | 30643251 | chr11:124611997 | C=0.925, G=0.075 | 0.0758 | 0.012431 | 0.0084-0.0164 | 1.00E-09 |
| SmkInit | rs540356 | Smoking initiation | 36477530 | chr11:132203816 | A=0.373, C=0.627 | 0.401 | 0.00933 | 0.0076-0.0111 | 4.00E-26 |
| SmkInit | rs540356 | Smoking initiation | 36477530 | chr11:132203816 | A=0.373, C=0.627 | 0.359 | 0.00856 | 0.007-0.0101 | 4.00E-26 |
| SmkInit | rs35891966 | Smoking initiation (ever regular vs never regular) | 30679032 | chr11:20129311 | A=0.072, G=0.928 | 0.0719 | 0.0237 | 0.016-0.032 | 1.00E-08 |
| SmkInit | rs35891966 | Smoking status | 30595370 | chr11:20129311 | A=0.072, G=0.928 | NR | NA | NA | 2.00E-08 |
| SmkInit | rs35891966 | Brain morphology (MOSTest) | 32665545 | chr11:20129311 | A=0.072, G=0.928 | NR | NA | NA | 1.00E-14 |
| SmkInit | rs35891966 | Cortical surface area (MOSTest) | 32665545 | chr11:20129311 | A=0.072, G=0.928 | NR | NA | NA | 4.00E-09 |
| SmkInit | rs35891966 | General cognitive ability | 29844566 | chr11:20129311 | A=0.072, G=0.928 | NR | 5.69 | - | 1.00E-08 |
| SmkInit | rs35891966 | Reaction time | 29844566 | chr11:20129311 | A=0.072, G=0.928 | NR | 0.0081298 | 0.0048-0.0115 | 2.00E-06 |
| SmkInit | rs35891966 | Whole brain restricted isotropic diffusion (multivariate analysis) | 35505052 | chr11:20129311 | A=0.072, G=0.928 | NR | NA | NA | 4.00E-18 |
| SmkInit | rs35891966 | Whole brain restricted directional diffusion (multivariate analysis) | 35505052 | chr11:20129311 | A=0.072, G=0.928 | NR | NA | NA | 3.00E-16 |
| SmkInit | rs35891966 | Smoking initiation (ever regular vs never regular) (MTAG) | 30643251 | chr11:20129311 | A=0.072, G=0.928 | 0.069 | 0.0149029 | 0.01-0.02 | 5.00E-10 |
| SmkInit | rs35891966 | Cortical surface area | 34560273 | chr11:20129311 | A=0.072, G=0.928 | 0.0748 | NA | NA | 1.00E-17 |
| SmkInit | rs35891966 | Cortical thickness | 34560273 | chr11:20129311 | A=0.072, G=0.928 | 0.0748 | NA | NA | 7.00E-16 |
| SmkInit | rs35891966 | Smoking status (ever vs never smokers) | 30643258 | chr11:20129311 | A=0.072, G=0.928 | 0.0722 | 0.024323925 | 0.016-0.033 | 7.00E-09 |
| SmkInit | rs35891966 | Vertex-wise cortical thickness | 34910505 | chr11:20129311 | A=0.072, G=0.928 | 0.07 | 8.2 | NA | 3.00E-16 |
| SmkInit | rs35891966 | Vertex-wise cortical surface area | 34910505 | chr11:20129311 | A=0.072, G=0.928 | 0.07 | 10.55 | NA | 5.00E-26 |
| SmkInit | rs35891966 | Brain morphology (MOSTest) | 35164939 | chr11:20129311 | A=0.072, G=0.928 | NR | NA | NA | 2.00E-17 |
| SmkInit | rs35891966 | Smoking initiation | 36477530 | chr11:20129311 | A=0.072, G=0.928 | 0.0682 | 0.011 | 0.0076-0.0144 | 2.00E-10 |
| SmkInit | rs35891966 | Smoking initiation | 36477530 | chr11:20129311 | A=0.072, G=0.928 | 0.0636 | 0.0111 | 0.0079-0.0143 | 2.00E-12 |
| SmkInit | rs35891966 | Smoking status (standard GWA) | 37106081 | chr11:20129311 | A=0.072, G=0.928 | NR | 0.018977 | 0.012-0.026 | 2.00E-08 |
| SmkInit | rs35891966 | Personality traits or cognitive traits (multivariate analysis) | 37365406 | chr11:20129311 | A=0.072, G=0.928 | NR | NA | NA | 1.00E-09 |
| SmkInit | rs6265 | Smoking initiation | 30617275 | chr11:27679916 | C=0.803, T=0.197 | NR | 0.019 | 0.012-0.026() | 8.00E-12 |
| SmkInit | rs6265 | Body mass index | 28892062 | chr11:27679916 | C=0.803, T=0.197 | 0.2 | 0.04 | 0.034-0.046 | 2.00E-51 |
| SmkInit | rs6265 | Waist-hip ratio | 30239722 | chr11:27679916 | C=0.803, T=0.197 | 0.1839 | 0.0183 | 0.014-0.023 | 4.00E-17 |
| SmkInit | rs6265 | Smoking initiation (ever regular vs never regular) | 30679032 | chr11:27679916 | C=0.803, T=0.197 | 0.19 | 0.01 | 0.0041-0.0159 | 2.00E-10 |
| SmkInit | rs6265 | Body mass index | 30239722 | chr11:27679916 | C=0.803, T=0.197 | 0.1836 | 0.0413 | 0.037-0.045 | 7.00E-89 |
| SmkInit | rs6265 | Body mass index | 30239722 | chr11:27679916 | C=0.803, T=0.197 | 0.1836 | 0.0413 | 0.037-0.045 | 7.00E-89 |
| SmkInit | rs6265 | Body mass index | 30239722 | chr11:27679916 | C=0.803, T=0.197 | 0.1836 | 0.0413 | 0.037-0.045 | 7.00E-89 |
| SmkInit | rs6265 | Body mass index | 30595370 | chr11:27679916 | C=0.803, T=0.197 | NR | NA | NA | 8.00E-61 |
| SmkInit | rs6265 | Smoking behavior | 20418890 | chr11:27679916 | C=0.803, T=0.197 | 0.79 | 1.06 | 1.04-1.08 | 2.00E-08 |
| SmkInit | rs6265 | Weight | 19079260 | chr11:27679916 | C=0.803, T=0.197 | 0.85 | 4 | 2.47-5.53 | 2.00E-07 |
| SmkInit | rs6265 | Body mass index | 19079260 | chr11:27679916 | C=0.803, T=0.197 | 0.85 | 4.58 | 3.07-6.09% | 5.00E-10 |
| SmkInit | rs6265 | Smoking status | 30595370 | chr11:27679916 | C=0.803, T=0.197 | NR | NA | NA | 2.00E-15 |
| SmkInit | rs6265 | Body mass index | 31669095 | chr11:27679916 | C=0.803, T=0.197 | NR | NA | NA | 3.00E-58 |
| SmkInit | rs6265 | Caffeine consumption from coffee or tea | 33287642 | chr11:27679916 | C=0.803, T=0.197 | 0.810755 | 0.021572 | 0.016-0.027 | 3.00E-13 |
| SmkInit | rs6265 | Smoking initiation | 33082346 | chr11:27679916 | C=0.803, T=0.197 | NR | 0.966 | NA | 1.00E-14 |
| SmkInit | rs6265 | C-reactive protein levels | 31900758 | chr11:27679916 | C=0.803, T=0.197 | 0.811757 | 0.0227799 | 0.018-0.028 | 8.00E-19 |
| SmkInit | rs6265 | Coffee consumption | 31959922 | chr11:27679916 | C=0.803, T=0.197 | NR | 0.054 | 0.032-0.076 | 3.00E-06 |
| SmkInit | rs6265 | Hip circumference | 25673412 | chr11:27679916 | C=0.803, T=0.197 | 0.1926 | 0.0343 | 0.025-0.043 | 1.00E-14 |
| SmkInit | rs6265 | Hip circumference | 25673412 | chr11:27679916 | C=0.803, T=0.197 | 0.1926 | 0.0353 | 0.024-0.047 | 1.00E-09 |
| SmkInit | rs6265 | Hip circumference | 25673412 | chr11:27679916 | C=0.803, T=0.197 | 0.1926 | 0.0317 | 0.02-0.044 | 4.00E-07 |
| SmkInit | rs6265 | Externalizing behaviour (multivariate analysis) | 34446935 | chr11:27679916 | C=0.803, T=0.197 | 0.814 | 0.015 | 0.013-0.017 | 2.00E-24 |
| SmkInit | rs6265 | Weight | 34594039 | chr11:27679916 | C=0.803, T=0.197 | NR | 0.0306 | 0.027-0.034 | 3.00E-58 |
| SmkInit | rs6265 | Adult body size | 32376654 | chr11:27679916 | C=0.803, T=0.197 | 0.811547 | 0.0245417 | 0.021-0.028 | 2.00E-44 |
| SmkInit | rs6265 | Smoking initiation (ever regular vs never regular) (MTAG) | 30643251 | chr11:27679916 | C=0.803, T=0.197 | 0.188 | 0.0153708 | 0.013-0.018 | 9.00E-29 |
| SmkInit | rs6265 | Age of smoking initiation (MTAG) | 30643251 | chr11:27679916 | C=0.803, T=0.197 | 0.181 | 0.0153388 | 0.012-0.019 | 6.00E-16 |
| SmkInit | rs6265 | Cigarettes smoked per day (MTAG) | 30643251 | chr11:27679916 | C=0.803, T=0.197 | 0.181 | 0.014062 | 0.0085-0.0196 | 7.00E-07 |
| SmkInit | rs6265 | Body mass index | 30108127 | chr11:27679916 | C=0.803, T=0.197 | NR | 0.042 | NA | 2.00E-39 |
| SmkInit | rs6265 | Smoking initiation (ever regular vs never regular) | 30643251 | chr11:27679916 | C=0.803, T=0.197 | 0.188 | 0.029275492 | 0.023-0.036 | 3.00E-19 |
| SmkInit | rs6265 | Body mass index | 31217584 | chr11:27679916 | C=0.803, T=0.197 | NR | 0.04783296 | 0.03-0.065 | 1.00E-07 |
| SmkInit | rs6265 | Body mass index | 29273807 | chr11:27679916 | C=0.803, T=0.197 | 0.816 | 0.0405 | 0.036-0.045 | 3.00E-68 |
| SmkInit | rs6265 | Lifetime smoking | 34855049 | chr11:27679916 | C=0.803, T=0.197 | NR | NA | NA | 2.00E-16 |
| SmkInit | rs6265 | Lifetime smoking (without educational attainment) | 34855049 | chr11:27679916 | C=0.803, T=0.197 | NR | NA | NA | 2.00E-11 |
| SmkInit | rs6265 | Predicted visceral adipose tissue | 31501611 | chr11:27679916 | C=0.803, T=0.197 | 0.1896 | 0.02457576 | 0.018-0.031 | 2.00E-13 |
| SmkInit | rs6265 | Smoking status (ever vs never smokers) | 30643258 | chr11:27679916 | C=0.803, T=0.197 | 0.1917 | 0.02215149 | 0.017-0.027 | 4.00E-18 |
| SmkInit | rs6265 | General risk tolerance (MTAG) | 30643258 | chr11:27679916 | C=0.803, T=0.197 | 0.188 | 0.0095 | 0.0066-0.0124 | 3.00E-10 |
| SmkInit | rs6265 | Smoking initiation | 36477530 | chr11:27679916 | C=0.803, T=0.197 | 0.187 | 0.0196 | 0.017-0.022 | 9.00E-70 |
| SmkInit | rs6265 | Risk-taking behavior (multivariate analysis) | 36324656 | chr11:27679916 | C=0.803, T=0.197 | NR | NA | NA | 2.00E-18 |
| SmkInit | rs6265 | Smoking initiation | 36477530 | chr11:27679916 | C=0.803, T=0.197 | 0.43685 | 0.0163 | 0.011-0.021 | 5.00E-10 |
| SmkInit | rs6265 | Penn alcohol craving scale in alcohol dependence (recessive genetic model) | 36551763 | chr11:27679916 | C=0.803, T=0.197 | NR | 12.724868 | NA | 5.00E-07 |
| SmkInit | rs6265 | Smoking initiation | 36477530 | chr11:27679916 | C=0.803, T=0.197 | 0.203 | 0.019 | 0.017-0.021 | 2.00E-87 |
| SmkInit | rs6265 | Body mass index | 38538606 | chr11:27679916 | C=0.803, T=0.197 | NR | 0.0413936 | 0.035-0.047 | 2.00E-41 |
| SmkInit | rs6265 | Cigarettes smoked per day (MTAG) | 36376304 | chr11:27679916 | C=0.803, T=0.197 | NR | 0.02098 | 0.015-0.027 | 5.00E-12 |
| SmkInit | rs6265 | Body mass index | 36581621 | chr11:27679916 | C=0.803, T=0.197 | NR | 0.0396 | 0.036-0.043 | 1.00E-122 |
| SmkInit | rs6265 | Body mass index (MTAG) | 36376304 | chr11:27679916 | C=0.803, T=0.197 | NR | 0.03997 | 0.036-0.044 | 3.00E-83 |
| SmkInit | rs6265 | Sleep apnea (MTAG) | 36525587 | chr11:27679916 | C=0.803, T=0.197 | NR | 0.02198 | 0.012-0.032 | 2.00E-14 |
| SmkInit | rs6265 | Coronary artery disease | 35915156 | chr11:27679916 | C=0.803, T=0.197 | 0.2298 | 0.0254 | 0.016-0.034 | 4.00E-08 |
| SmkInit | rs6265 | Cannabis use disorder (MTAG) | 37156939 | chr11:27679916 | C=0.803, T=0.197 | NR | 0.0231466 | 0.016-0.031 | 8.00E-10 |
| SmkInit | rs6265 | Smoking initiation (MTAG) | 37156939 | chr11:27679916 | C=0.803, T=0.197 | NR | 0.0152717 | 0.011-0.019 | 2.00E-14 |
| SmkInit | rs6265 | Body mass index | 37280435 | chr11:27679916 | C=0.803, T=0.197 | NR | 0.0395946 | 0.035-0.044 | 1.00E-57 |
| SmkInit | rs2939756 | Smoking status | 30595370 | chr11:41436297 | A=0.489, G=0.511 | NR | NA | NA | 4.00E-12 |
| SmkInit | rs2939756 | Smoking initiation (ever regular vs never regular) | 30643251 | chr11:41436297 | A=0.489, G=0.511 | 0.48 | 0.015699685 | 0.011-0.021 | 7.00E-10 |
| SmkInit | rs2939756 | Smoking status (ever vs never smokers) | 30643258 | chr11:41436297 | A=0.489, G=0.511 | 0.4805 | 0.011942013 | 0.008-0.0159 | 3.00E-09 |
| SmkInit | rs574835 | Crohn's disease or Leprosy (opposite effect) | 35512355 | chr11:64110668 | A=0.366, G=0.634 | NR | NA | NA | 3.00E-09 |
| SmkInit | rs7947391 | Waist circumference adjusted for body mass index | 31669095 | chr11:66186882 | A=0.422, G=0.578 | NR | NA | NA | 3.00E-17 |
| SmkInit | rs7947391 | Externalizing behaviour (multivariate analysis) | 34446935 | chr11:66186882 | A=0.422, G=0.578 | 0.414 | 0.007 | 0.005-0.009 | 2.00E-08 |
| SmkInit | rs7947391 | General risk tolerance (MTAG) | 30643258 | chr11:66186882 | A=0.422, G=0.578 | 0.411 | 0.0066 | 0.0042-0.009 | 4.00E-08 |
| SmkInit | rs7947391 | Risk-taking behavior (multivariate analysis) | 36324656 | chr11:66186882 | A=0.422, G=0.578 | NR | NA | NA | 1.00E-08 |
| SmkInit | rs7947391 | Waist circumference adjusted for body mass index | 34021172 | chr11:66186882 | A=0.422, G=0.578 | NR | 0.0194938 | 0.014-0.025 | 2.00E-11 |
| SmkInit | rs4944844 | Smoking initiation (ever regular vs never regular) (MTAG) | 30643251 | chr11:73311705 | C=0.845, T=0.155 | 0.829 | 0.0101559 | 0.0073-0.013 | 2.00E-12 |
| SmkInit | rs7929518 | Smoking initiation (ever regular vs never regular) (MTAG) | 30643251 | chr11:85980958 | A=0.228, G=0.772 | 0.773 | 0.010027 | 0.0075-0.0125 | 6.00E-15 |
| SmkInit | rs7929518 | Smoking initiation (ever regular vs never regular) | 30643251 | chr11:85980958 | A=0.228, G=0.772 | 0.773 | 0.019236272 | 0.013-0.025 | 3.00E-10 |
| SmkInit | rs7929518 | Smoking initiation | 36477530 | chr11:85980958 | A=0.228, G=0.772 | 0.771 | 0.009 | 0.007-0.011 | 2.00E-18 |
| SmkInit | rs7929518 | Smoking initiation | 36477530 | chr11:85980958 | A=0.228, G=0.772 | 0.75 | 0.00869 | 0.0069-0.0104 | 3.00E-22 |
| SmkInit | rs7929518 | Smoking initiation (MTAG) | 37156939 | chr11:85980958 | A=0.228, G=0.772 | NR | 0.0123421 | 0.0086-0.016 | 6.00E-11 |
| SmkInit | rs1971318 | High-density lipoprotein levels (MTAG) | 36376304 | chr12:121389500 | C=0.872, T=0.128 | NR | 0.03015 | 0.023-0.037 | 6.00E-17 |
| SmkInit | rs2292239 | Smoking initiation | 30617275 | chr12:56482180 | G=0.667, T=0.333 | 0.66 | 0.0121 | 0.0062-0.018() | 3.00E-08 |
| SmkInit | rs2292239 | Alopecia areata | 25608926 | chr12:56482180 | G=0.667, T=0.333 | NR | 1.25 | NA | 4.00E-09 |
| SmkInit | rs2292239 | Type 1 diabetes | 19430480 | chr12:56482180 | G=0.667, T=0.333 | NR | NA | NA | 2.00E-25 |
| SmkInit | rs2292239 | Type 1 diabetes | 18978792 | chr12:56482180 | G=0.667, T=0.333 | NR | NA | NA | 3.00E-16 |
| SmkInit | rs2292239 | Type 1 diabetes | 17554260 | chr12:56482180 | G=0.667, T=0.333 | 0.34 | 1.28 | 1.21-1.35 | 2.00E-20 |
| SmkInit | rs2292239 | Type 1 diabetes | 21829393 | chr12:56482180 | G=0.667, T=0.333 | NR | 1.3 | NA | 3.00E-27 |
| SmkInit | rs2292239 | Smoking initiation | 36477530 | chr12:56482180 | G=0.667, T=0.333 | 0.676 | 0.00915 | 0.0075-0.0108 | 2.00E-28 |
| SmkInit | rs7969559 | Smoking status | 30595370 | chr12:69655167 | A=0.318, G=0.682 | NR | NA | NA | 1.00E-10 |
| SmkInit | rs7969559 | Smoking initiation | 33082346 | chr12:69655167 | A=0.318, G=0.682 | NR | 1.025 | NA | 2.00E-10 |
| SmkInit | rs7969559 | Externalizing behaviour (multivariate analysis) | 34446935 | chr12:69655167 | A=0.318, G=0.682 | 0.284 | 0.007 | 0.005-0.009 | 6.00E-09 |
| SmkInit | rs7969559 | Smoking initiation (ever regular vs never regular) (MTAG) | 30643251 | chr12:69655167 | A=0.318, G=0.682 | 0.713 | 0.00803583 | 0.0057-0.0104 | 2.00E-11 |
| SmkInit | rs7969559 | Smoking initiation (ever regular vs never regular) | 30643251 | chr12:69655167 | A=0.318, G=0.682 | 0.713 | 0.017015876 | 0.011-0.023 | 2.00E-09 |
| SmkInit | rs7969559 | Lifetime smoking | 34855049 | chr12:69655167 | A=0.318, G=0.682 | NR | NA | NA | 3.00E-09 |
| SmkInit | rs7969559 | Lifetime smoking (without educational attainment) | 34855049 | chr12:69655167 | A=0.318, G=0.682 | NR | NA | NA | 1.00E-08 |
| SmkInit | rs7969559 | Lysozyme C levels | 36168886 | chr12:69655167 | A=0.318, G=0.682 | 0.370698 | 0.213253 | 0.16-0.27 | 1.00E-14 |
| SmkInit | rs7969559 | Smoking initiation | 36477530 | chr12:69655167 | A=0.318, G=0.682 | 0.71 | 0.00867 | 0.0068-0.0105 | 1.00E-19 |
| SmkInit | rs7984311 | Smoking initiation | 36477530 | chr13:100741752 | A=0.386, G=0.614 | 0.407 | 0.00925 | 0.0077-0.0108 | 8.00E-33 |
| SmkInit | rs7986094 | Urate levels | 31578528 | chr13:31029931 | A=0.306, C=0.694 | 0.3016 | 0.023916 | 0.016-0.032/ | 2.00E-08 |
| SmkInit | rs7986094 | Smoking initiation | 36477530 | chr13:31029931 | A=0.306, C=0.694 | 0.71 | 0.00661 | 0.0049-0.0083 | 7.00E-15 |
| SmkInit | rs61959481 | Smoking initiation | 33082346 | chr13:55834929 | A=0.203, G=0.797 | NR | 0.977 | NA | 3.00E-08 |
| SmkInit | rs61959481 | Smoking initiation (ever regular vs never regular) (MTAG) | 30643251 | chr13:55834929 | A=0.203, G=0.797 | 0.21 | 0.0105913 | 0.008-0.0132 | 3.00E-15 |
| SmkInit | rs61959481 | Smoking initiation (ever regular vs never regular) | 30643251 | chr13:55834929 | A=0.203, G=0.797 | 0.21 | 0.0203442 | 0.014-0.026 | 8.00E-11 |
| SmkInit | rs61959481 | Smoking initiation | 36477530 | chr13:55834929 | A=0.203, G=0.797 | 0.207 | 0.012 | 0.0099-0.0141 | 4.00E-29 |
| SmkInit | rs9538536 | Noncognitive aspects of educational attainment | 33414549 | chr13:60536321 | G=0.669, T=0.331 | 0.331014 | 0.04845775 | 0.034-0.063 | 9.00E-11 |
| SmkInit | rs9529052 | Smoking initiation | 36477530 | chr13:66940097 | A=0.506, T=0.494 | 0.476 | 0.0106 | 0.0091-0.0121 | 2.00E-42 |
| SmkInit | rs2783130 | Externalizing behaviour (multivariate analysis) | 34446935 | chr13:80170160 | A=0.497, G=0.503 | 0.516 | 0.009 | 0.007-0.011 | 2.00E-13 |
| SmkInit | rs2783130 | Smoking status (ever vs never smokers) | 30643258 | chr13:80170160 | A=0.497, G=0.503 | 0.5229 | 0.011386399 | 0.0074-0.0153 | 1.00E-08 |
| SmkInit | rs8001839 | Insomnia | 35835914 | chr13:97110073 | A=0.677, G=0.323 | NR | 0.006 | 0.004-0.008 | 1.00E-08 |
| SmkInit | rs7333559 | Smoking status | 30595370 | chr13:100546450 | A=0.791, G=0.209 | NR | NA | NA | 5.00E-09 |
| SmkInit | rs7333559 | Smoking initiation (ever regular vs never regular) (MTAG) | 30643251 | chr13:100546450 | A=0.791, G=0.209 | 0.783 | 0.0116935 | 0.0091-0.0143 | 1.00E-18 |
| SmkInit | rs7333559 | Smoking initiation (ever regular vs never regular) | 30643251 | chr13:100546450 | A=0.791, G=0.209 | 0.783 | 0.023212526 | 0.017-0.029 | 6.00E-14 |
| SmkInit | rs7333559 | Age of smoking initiation (MTAG) | 30643251 | chr13:100546450 | A=0.791, G=0.209 | 0.78 | 0.013817 | 0.01-0.017 | 8.00E-15 |
| SmkInit | rs7333559 | Smoking status (ever vs never smokers) | 30643258 | chr13:100546450 | A=0.791, G=0.209 | 0.7902 | 0.015744165 | 0.011-0.021 | 3.00E-09 |
| SmkInit | rs7333559 | Lifetime smoking index | 31689377 | chr13:100546450 | A=0.791, G=0.209 | 0.212 | 0.015 | 0.011-0.019 | 3.00E-10 |
| SmkInit | rs2022815 | Smoking initiation | 36477530 | chr14:28356733 | A=0.336, G=0.664 | 0.3 | 0.0073 | 0.0057-0.0089 | 9.00E-18 |
| SmkInit | rs66480876 | Smoking initiation | 36477530 | chr14:32448189 | C=0.558, G=0.442 | 0.452 | 0.00812 | 0.0064-0.0098 | 1.00E-20 |
| SmkInit | rs66480876 | Smoking initiation | 36477530 | chr14:32448189 | C=0.558, G=0.442 | 0.468 | 0.00734 | 0.0058-0.0089 | 5.00E-21 |
| SmkInit | rs9323328 | Smoking initiation (ever regular vs never regular) (MTAG) | 30643251 | chr14:58653514 | A=0.473, G=0.527 | 0.537 | 0.00694448 | 0.0048-0.0091 | 2.00E-10 |
| SmkInit | rs9323328 | Smoking initiation (ever regular vs never regular) | 30643251 | chr14:58653514 | A=0.473, G=0.527 | 0.537 | 0.01423703 | 0.0092-0.0192 | 3.00E-08 |
| SmkInit | rs9323328 | Lifetime smoking | 34855049 | chr14:58653514 | A=0.473, G=0.527 | NR | NA | NA | 4.00E-08 |
| SmkInit | rs9323328 | Smoking initiation | 36477530 | chr14:58653514 | A=0.473, G=0.527 | 0.535 | 0.00803 | 0.0063-0.0097 | 2.00E-20 |
| SmkInit | rs17594561 | Externalizing behaviour (multivariate analysis) | 34446935 | chr14:79618750 | A=0.583, G=0.417 | 0.556 | 0.006 | 0.004-0.008 | 4.00E-08 |
| SmkInit | rs1381287 | Self-reported risk-taking behaviour | 30271922 | chr14:98597552 | C=0.495, T=0.505 | 0.46 | 1.023 | 1.015-1.032 | 1.00E-08 |
| SmkInit | rs1381287 | Smoking status (ever vs never smokers) | 30271922 | chr14:98597552 | C=0.495, T=0.505 | 0.456905 | 0.0050533 | 0.003-0.0071 | 1.00E-06 |
| SmkInit | rs1381287 | Self-reported risk-taking behaviour | 30181555 | chr14:98597552 | C=0.495, T=0.505 | 0.45 | 0.025 | 0.015-0.035 | 3.00E-06 |
| SmkInit | rs1381287 | Externalizing behaviour (multivariate analysis) | 34446935 | chr14:98597552 | C=0.495, T=0.505 | 0.543 | 0.014 | 0.012-0.016 | 8.00E-30 |
| SmkInit | rs1381287 | Smoking initiation (ever regular vs never regular) (MTAG) | 30643251 | chr14:98597552 | C=0.495, T=0.505 | 0.467 | 0.00921863 | 0.0071-0.0114 | 3.00E-17 |
| SmkInit | rs1381287 | Smoking initiation (ever regular vs never regular) | 30643251 | chr14:98597552 | C=0.495, T=0.505 | 0.467 | 0.01801658 | 0.013-0.023 | 2.00E-12 |
| SmkInit | rs1381287 | General risk tolerance (MTAG) | 30643258 | chr14:98597552 | C=0.495, T=0.505 | 0.47 | 0.0098 | 0.0074-0.0122 | 1.00E-16 |
| SmkInit | rs1381287 | Risk-taking behavior (multivariate analysis) | 36324656 | chr14:98597552 | C=0.495, T=0.505 | NR | NA | NA | 5.00E-20 |
| SmkInit | rs1381287 | Smoking initiation | 36477530 | chr14:98597552 | C=0.495, T=0.505 | 0.474 | 0.0113 | 0.0096-0.013 | 1.00E-38 |
| SmkInit | rs1381287 | Smoking initiation | 36477530 | chr14:98597552 | C=0.495, T=0.505 | 0.457 | 0.0111 | 0.0096-0.0126 | 2.00E-46 |
| SmkInit | rs1435679 | Externalizing behaviour (multivariate analysis) | 34446935 | chr15:36399245 | A=0.582, G=0.418 | 0.445 | 0.006 | 0.004-0.008 | 5.00E-08 |
| SmkInit | rs1435679 | Smoking initiation | 36477530 | chr15:36399245 | A=0.582, G=0.418 | 0.56 | 0.00811 | 0.0064-0.0098 | 1.00E-20 |
| SmkInit | rs1435679 | Smoking initiation | 36477530 | chr15:36399245 | A=0.582, G=0.418 | 0.574 | 0.00703 | 0.0055-0.0086 | 4.00E-18 |
| SmkInit | rs34488670 | Major depressive disorder (broad) | 38177345 | chr15:47684936 | C=0.207, T=0.793 | 0.7856 | 0.0228 | 0.016-0.03 | 1.00E-09 |
| SmkInit | rs34488670 | Depression | 30718901 | chr15:47684936 | C=0.207, T=0.793 | 0.2113 | 1.0193679 | 1.01-1.02 | 5.00E-13 |
| SmkInit | rs34488670 | Age of smoking initiation (MTAG) | 30643251 | chr15:47684936 | C=0.207, T=0.793 | 0.217 | 0.0153569 | 0.012-0.019 | 4.00E-18 |
| SmkInit | rs34488670 | Risk-taking tendency (4-domain principal component model) | 30643258 | chr15:47684936 | C=0.207, T=0.793 | 0.7885 | 0.021578157 | 0.015-0.028 | 8.00E-12 |
| SmkInit | rs34488670 | Number of sexual partners | 30643258 | chr15:47684936 | C=0.207, T=0.793 | 0.7887 | 0.016623866 | 0.011-0.022 | 1.00E-08 |
| SmkInit | rs34488670 | Depression | 34924174 | chr15:47684936 | C=0.207, T=0.793 | NR | 3.00E-04 | NA | 9.00E-10 |
| SmkInit | rs34488670 | Smoking initiation | 36477530 | chr15:47684936 | C=0.207, T=0.793 | 0.212 | 0.0176 | 0.016-0.02 | 8.00E-62 |
| SmkInit | rs34488670 | Educational attainment | 35361970 | chr15:47684936 | C=0.207, T=0.793 | 0.7882 | 0.0140229 | 0.012-0.017 | 7.00E-28 |
| SmkInit | rs34488670 | Depression | 37464041 | chr15:47684936 | C=0.207, T=0.793 | 0.784725 | 0.0231049 | 0.016-0.03 | 1.00E-11 |
| SmkInit | rs2289791 | Smoking initiation (ever regular vs never regular) | 30643251 | chr15:67476952 | G=0.759, T=0.241 | 0.247 | 0.017725516 | 0.012-0.024 | 2.00E-09 |
| SmkInit | rs2289791 | Smoking initiation | 36477530 | chr15:67476952 | G=0.759, T=0.241 | 0.247 | 0.0099 | 0.0079-0.0119 | 6.00E-23 |
| SmkInit | rs11632439 | Smoking initiation | 36477530 | chr15:80987012 | A=0.552, G=0.448 | 0.465 | 0.00717 | 0.0055-0.0089 | 1.00E-16 |
| SmkInit | rs11632439 | Insomnia | 35835914 | chr15:80987012 | A=0.552, G=0.448 | NR | 0.005 | 0.003-0.007 | 6.00E-09 |
| SmkInit | rs11632439 | Systolic blood pressure (standard GWA) | 37106081 | chr15:80987012 | A=0.552, G=0.448 | NR | 0.3198 | 0.22-0.42 | 1.00E-09 |
| SmkInit | rs11632439 | Systolic blood pressure (weighted GWA) | 37106081 | chr15:80987012 | A=0.552, G=0.448 | NR | 0.40516 | 0.24-0.57 | 8.00E-07 |
| SmkInit | rs1155641 | Externalizing behaviour (multivariate analysis) | 34446935 | chr15:97502995 | A=0.335, G=0.665 | 0.652 | 0.01 | 0.008-0.012 | 1.00E-15 |
| SmkInit | rs1155641 | Smoking initiation | 36477530 | chr15:97502995 | A=0.335, G=0.665 | 0.345 | 0.0072 | 0.0054-0.009 | 3.00E-15 |
| SmkInit | rs1155641 | Smoking initiation | 36477530 | chr15:97502995 | A=0.335, G=0.665 | 0.347 | 0.00668 | 0.0051-0.0083 | 1.00E-16 |
| SmkInit | rs6598539 | Age of smoking initiation (MTAG) | 30643251 | chr15:99204483 | C=0.513, T=0.487 | 0.511 | 0.00875411 | 0.0059-0.0116 | 2.00E-09 |
| SmkInit | rs6598539 | Smoking status (ever vs never smokers) | 30643258 | chr15:99204483 | C=0.513, T=0.487 | 0.4883 | 0.012742009 | 0.0085-0.017 | 4.00E-09 |
| SmkInit | rs6598539 | Lifetime smoking index | 31689377 | chr15:99204483 | C=0.513, T=0.487 | 0.489 | 0.012 | 0.0081-0.0159 | 5.00E-09 |
| SmkInit | rs6598539 | Smoking initiation | 36477530 | chr15:99204483 | C=0.513, T=0.487 | 0.51 | 0.00804 | 0.0063-0.0097 | 3.00E-20 |
| SmkInit | rs56225373 | Externalizing behaviour (multivariate analysis) | 34446935 | chr16:13755408 | G=0.158, T=0.842 | 0.839 | 0.011 | 0.0071-0.0149 | 2.00E-10 |
| SmkInit | rs6497840 | Smoking initiation (ever regular vs never regular) (MTAG) | 30643251 | chr16:25351633 | A=0.714, G=0.286 | 0.707 | 0.0115813 | 0.0092-0.014 | 1.00E-21 |
| SmkInit | rs6497840 | Smoking initiation (ever regular vs never regular) | 30643251 | chr16:25351633 | A=0.714, G=0.286 | 0.707 | 0.022770278 | 0.017-0.028 | 2.00E-15 |
| SmkInit | rs6497840 | Age of smoking initiation (MTAG) | 30643251 | chr16:25351633 | A=0.714, G=0.286 | 0.71 | 0.0100816 | 0.0069-0.0133 | 5.00E-10 |
| SmkInit | rs6497840 | Smoking initiation | 36477530 | chr16:25351633 | A=0.714, G=0.286 | 0.708 | 0.0142 | 0.012-0.016 | 5.00E-49 |
| SmkInit | rs6497840 | Smoking initiation | 36477530 | chr16:25351633 | A=0.714, G=0.286 | 0.697 | 0.0126 | 0.011-0.014 | 1.00E-50 |
| SmkInit | rs55942317 | Externalizing behaviour (multivariate analysis) | 34446935 | chr16:49626772 | A=0.081, G=0.919 | 0.911 | 0.016 | 0.012-0.02 | 5.00E-14 |
| SmkInit | rs55942317 | Number of sexual partners | 30643258 | chr16:49626772 | A=0.081, G=0.919 | 0.0903 | 0.029158004 | 0.021-0.037 | 2.00E-12 |
| SmkInit | rs55942317 | Risk-taking tendency (4-domain principal component model) | 30643258 | chr16:49626772 | A=0.081, G=0.919 | 0.09 | 0.024579043 | 0.016-0.033 | 5.00E-08 |
| SmkInit | rs4785187 | Externalizing behaviour (multivariate analysis) | 34446935 | chr16:49766772 | A=0.245, G=0.755 | 0.778 | 0.009 | 0.007-0.011 | 1.00E-11 |
| SmkInit | rs4785187 | Educational attainment (years of education) | 30038396 | chr16:49766772 | A=0.245, G=0.755 | 0.2253 | 0.01 | 0.0067-0.0133 | 3.00E-09 |
| SmkInit | rs4785187 | Smoking initiation (ever regular vs never regular) | 30643251 | chr16:49766772 | A=0.245, G=0.755 | 0.223 | 0.01997724 | 0.014-0.026 | 7.00E-11 |
| SmkInit | rs4785187 | Educational attainment (MTAG) | 30038396 | chr16:49766772 | A=0.245, G=0.755 | 0.2253 | 0.0098 | 0.0067-0.0129 | 4.00E-10 |
| SmkInit | rs4785187 | Educational attainment | 35361970 | chr16:49766772 | A=0.245, G=0.755 | 0.2233 | 0.0116212 | 0.0092-0.0141 | 2.00E-20 |
| SmkInit | rs4785187 | Smoking initiation | 36477530 | chr16:49766772 | A=0.245, G=0.755 | 0.224 | 0.0123 | 0.01-0.014 | 2.00E-32 |
| SmkInit | rs4785187 | Smoking initiation | 36477530 | chr16:49766772 | A=0.245, G=0.755 | 0.254 | 0.0112 | 0.0095-0.0129 | 2.00E-36 |
| SmkInit | rs763053 | Smoking initiation (ever regular vs never regular) | 30679032 | chr16:735921 | C=0.267, T=0.733 | 0.225 | 0.016 | 0.011-0.021 | 5.00E-09 |
| SmkInit | rs763053 | Lifetime smoking (without educational attainment) | 34855049 | chr16:735921 | C=0.267, T=0.733 | NR | NA | NA | 2.00E-08 |
| SmkInit | rs763053 | Lifetime smoking | 34855049 | chr16:735921 | C=0.267, T=0.733 | NR | NA | NA | 7.00E-13 |
| SmkInit | rs763053 | Age at first sexual intercourse | 34211149 | chr16:735921 | C=0.267, T=0.733 | 0.774886 | 0.0162579 | 0.011-0.021 | 5.00E-11 |
| SmkInit | rs763053 | Smoking initiation | 36477530 | chr16:735921 | C=0.267, T=0.733 | 0.231 | 0.0138 | 0.012-0.016 | 3.00E-41 |
| SmkInit | rs114900182 | Smoking status | 30595370 | chr16:72629056 | C=0.941, G=0.059 | NR | NA | NA | 3.00E-10 |
| SmkInit | rs114900182 | General risk tolerance (MTAG) | 30643258 | chr16:72629056 | C=0.941, G=0.059 | 0.932 | 0.0136 | 0.0091-0.0181 | 6.00E-09 |
| SmkInit | rs114900182 | Whole body fat free mass (UKB data field 23101) | 38538606 | chr16:72629056 | C=0.941, G=0.059 | NR | 0.0171853 | 0.012-0.022 | 9.00E-12 |
| SmkInit | rs4888444 | Smoking status | 30595370 | chr16:75690279 | A=0.96, G=0.04 | NR | NA | NA | 1.00E-08 |
| SmkInit | rs4888444 | Externalizing behaviour (multivariate analysis) | 34446935 | chr16:75690279 | A=0.96, G=0.04 | 0.953 | 0.023 | 0.017-0.029 | 6.00E-15 |
| SmkInit | rs4888444 | Age of smoking initiation (MTAG) | 30643251 | chr16:75690279 | A=0.96, G=0.04 | 0.0406 | 0.0212826 | 0.014-0.029 | 9.00E-09 |
| SmkInit | rs4888444 | Smoking status (ever vs never smokers) | 30643258 | chr16:75690279 | A=0.96, G=0.04 | 0.9479 | 0.025578208 | 0.017-0.034 | 2.00E-08 |
| SmkInit | rs4888444 | Smoking initiation | 36477530 | chr16:75690279 | A=0.96, G=0.04 | 0.0442 | 0.024 | 0.02-0.028 | 4.00E-30 |
| SmkInit | rs4888444 | Smoking initiation | 36477530 | chr16:75690279 | A=0.96, G=0.04 | 0.0417 | 0.0224 | 0.018-0.026 | 4.00E-28 |
| SmkInit | rs4888444 | Educational attainment | 35361970 | chr16:75690279 | A=0.96, G=0.04 | 0.9553 | 0.0183364 | 0.013-0.023 | 6.00E-13 |
| SmkInit | rs4888444 | Height | 36224396 | chr16:75690279 | A=0.96, G=0.04 | 0.0408 | 0.0141 | 0.011-0.017 | 6.00E-19 |
| SmkInit | rs1050847 | Educational attainment (years of education) | 30595370 | chr16:87443734 | C=0.449, T=0.551 | NR | NA | NA | 4.00E-09 |
| SmkInit | rs1050847 | Smoking status | 30595370 | chr16:87443734 | C=0.449, T=0.551 | NR | NA | NA | 6.00E-08 |
| SmkInit | rs1050847 | General cognitive ability | 29844566 | chr16:87443734 | C=0.449, T=0.551 | NR | 4.489 | - | 7.00E-06 |
| SmkInit | rs1050847 | Educational attainment (years of education) | 30038396 | chr16:87443734 | C=0.449, T=0.551 | 0.5626 | 0.0092 | 0.0065-0.0119 | 9.00E-11 |
| SmkInit | rs1050847 | Smoking initiation (ever regular vs never regular) | 30643251 | chr16:87443734 | C=0.449, T=0.551 | 0.559 | 0.014830017 | 0.0098-0.0199 | 7.00E-09 |
| SmkInit | rs1050847 | Smoking initiation (ever regular vs never regular) (MTAG) | 30643251 | chr16:87443734 | C=0.449, T=0.551 | 0.559 | 0.00745744 | 0.0053-0.0096 | 9.00E-12 |
| SmkInit | rs1050847 | Educational attainment (MTAG) | 30038396 | chr16:87443734 | C=0.449, T=0.551 | 0.5626 | 0.0094 | 0.0069-0.0119 | 1.00E-12 |
| SmkInit | rs1050847 | Decaffeinated coffee consumption and/or neuroticism | 35898629 | chr16:87443734 | C=0.449, T=0.551 | NR | NA | NA | 4.00E-10 |
| SmkInit | rs1050847 | Lifetime smoking index | 31689377 | chr16:87443734 | C=0.449, T=0.551 | 0.426 | 0.011 | 0.0071-0.0149 | 1.00E-08 |
| SmkInit | rs1050847 | Cognitive performance (MTAG) | 30038396 | chr16:87443734 | C=0.449, T=0.551 | 0.5751 | 0.0149 | 0.01-0.019 | 1.00E-10 |
| SmkInit | rs1050847 | Smoking initiation | 36477530 | chr16:87443734 | C=0.449, T=0.551 | 0.553 | 0.00722 | 0.0055-0.0089 | 1.00E-16 |
| SmkInit | rs7195043 | Basal cell carcinoma | 38182794 | chr16:90020861 | C=0.556, T=0.444 | NR | 0.0770213 | 0.062-0.092 | 8.00E-25 |
| SmkInit | rs8067305 | Aspartate aminotransferase levels | 34594039 | chr17:1975657 | A=0.575, G=0.425 | NR | 0.0132 | 0.0093-0.0171 | 4.00E-11 |
| SmkInit | rs8067305 | Lifetime smoking (without educational attainment) | 34855049 | chr17:1975657 | A=0.575, G=0.425 | NR | NA | NA | 2.00E-09 |
| SmkInit | rs8067305 | Lifetime smoking | 34855049 | chr17:1975657 | A=0.575, G=0.425 | NR | NA | NA | 2.00E-11 |
| SmkInit | rs8067305 | Plasma anti-thyroglobulin and anti-thyroid peroxidase levels (bivariate analysis) | 29678681 | chr17:1975657 | A=0.575, G=0.425 | NR | 0.011 | 0.0064-0.0156 | 1.00E-06 |
| SmkInit | rs8067305 | Aspartate aminotransferase levels | 33462484 | chr17:1975657 | A=0.575, G=0.425 | NR | 0.0154 | 0.011-0.02 | 2.00E-10 |
| SmkInit | rs8069451 | Lung function (FVC) | 30595370 | chr17:37504933 | C=0.246, T=0.754 | NR | NA | NA | 1.00E-16 |
| SmkInit | rs8069451 | Lung function (FVC) | 30804560 | chr17:37504933 | C=0.246, T=0.754 | 0.7494 | 0.0202 | 0.015-0.025 | 7.00E-14 |
| SmkInit | rs8069451 | FEV1 | 30804560 | chr17:37504933 | C=0.246, T=0.754 | 0.7494 | 0.0189 | 0.014-0.024 | 2.00E-12 |
| SmkInit | rs8069451 | Asthma and cardiovascular disease | 35126453 | chr17:37504933 | C=0.246, T=0.754 | NR | NA | NA | 4.00E-08 |
| SmkInit | rs8069451 | Childhood asthma with severe exacerbations | 33328473 | chr17:37504933 | C=0.246, T=0.754 | NR | 0.2183 | 0.16-0.28 | 4.00E-12 |
| SmkInit | rs8069451 | Smoking initiation | 36477530 | chr17:37504933 | C=0.246, T=0.754 | 0.256 | 0.00967 | 0.0077-0.0116 | 2.00E-22 |
| SmkInit | rs11656151 | Waist-hip index | 34021172 | chr17:44068492 | A=0.799, G=0.201 | NR | 0.0187504 | 0.012-0.025 | 2.00E-08 |
| SmkInit | rs11656151 | Waist-to-hip ratio adjusted for BMI | 34021172 | chr17:44068492 | A=0.799, G=0.201 | NR | 0.0182514 | 0.012-0.025 | 5.00E-08 |
| SmkInit | rs11656151 | A body shape index | 34021172 | chr17:44068492 | A=0.799, G=0.201 | NR | 0.0233655 | 0.017-0.03 | 4.00E-12 |
| SmkInit | rs11656151 | Waist circumference adjusted for body mass index | 34021172 | chr17:44068492 | A=0.799, G=0.201 | NR | 0.0226537 | 0.016-0.029 | 2.00E-11 |
| SmkInit | rs11078713 | Smoking status | 30595370 | chr17:7795972 | A=0.592, G=0.408 | NR | NA | NA | 2.00E-08 |
| SmkInit | rs11078713 | Smoking initiation (ever regular vs never regular) | 30643251 | chr17:7795972 | A=0.592, G=0.408 | 0.419348 | 0.014582893 | 0.0095-0.0196 | 2.00E-08 |
| SmkInit | rs11078713 | Smoking initiation (ever regular vs never regular) (MTAG) | 30643251 | chr17:7795972 | A=0.592, G=0.408 | 0.419 | 0.00849662 | 0.0063-0.0106 | 1.00E-14 |
| SmkInit | rs11078713 | Risk-taking behavior (multivariate analysis) | 36324656 | chr17:7795972 | A=0.592, G=0.408 | NR | NA | NA | 2.00E-08 |
| SmkInit | rs745570 | Breast cancer | 25751625 | chr17:77781725 | A=0.505, G=0.495 | 0.5 | 1.0526316 | NA | 5.00E-07 |
| SmkInit | rs745570 | Breast cancer | 25751625 | chr17:77781725 | A=0.505, G=0.495 | 0.5 | 1.0526316 | 1.03-1.08 | 1.00E-09 |
| SmkInit | rs745570 | Monocyte count | 32888493 | chr17:77781725 | A=0.505, G=0.495 | 0.513574 | 0.014068 | 0.01-0.018 | 5.00E-13 |
| SmkInit | rs745570 | PR interval | 32439900 | chr17:77781725 | A=0.505, G=0.495 | 0.5133 | 0.3345 | 0.2-0.47 | 5.00E-07 |
| SmkInit | rs745570 | Monocyte count | 32888494 | chr17:77781725 | A=0.505, G=0.495 | 0.48581 | 0.013016205 | 0.0087-0.0174 | 4.00E-09 |
| SmkInit | rs745570 | PR interval | 32439900 | chr17:77781725 | A=0.505, G=0.495 | 0.53 | 0.35 | 0.23-0.47 | 3.00E-08 |
| SmkInit | rs745570 | Breast cancer | 27117709 | chr17:77781725 | A=0.505, G=0.495 | 0.51 | 1.0416667 | 1-1.09(1) | 6.00E-06 |
| SmkInit | rs745570 | Brain morphology (MOSTest) | 32665545 | chr17:77781725 | A=0.505, G=0.495 | NR | NA | NA | 1.00E-09 |
| SmkInit | rs745570 | Monocyte count | 32888493 | chr17:77781725 | A=0.505, G=0.495 | 0.495708 | NA | NA | 5.00E-13 |
| SmkInit | rs745570 | Breast cancer | 29059683 | chr17:77781725 | A=0.505, G=0.495 | 0.5 | 1.03 | 1.01-1.05() | 4.00E-10 |
| SmkInit | rs745570 | Smoking initiation (ever regular vs never regular) (MTAG) | 30643251 | chr17:77781725 | A=0.505, G=0.495 | 0.509 | 0.00681051 | 0.0047-0.0089 | 3.00E-10 |
| SmkInit | rs745570 | Smoking status (ever vs never smokers) | 30643258 | chr17:77781725 | A=0.505, G=0.495 | 0.4869 | 0.011424577 | 0.0075-0.0154 | 1.00E-08 |
| SmkInit | rs745570 | Vertex-wise cortical thickness | 34910505 | chr17:77781725 | A=0.505, G=0.495 | 0.5 | 6.37 | NA | 2.00E-10 |
| SmkInit | rs745570 | Smoking initiation (MTAG) | 37156939 | chr17:77781725 | A=0.505, G=0.495 | NR | 0.0087935 | 0.0056-0.0119 | 4.00E-08 |
| SmkInit | rs12945403 | Heel bone mineral density | 30048462 | chr17:80004094 | C=0.616, T=0.384 | NR | 0.0171079 | 0.013-0.021 | 8.00E-15 |
| SmkInit | rs12945403 | Heel bone mineral density | 30598549 | chr17:80004094 | C=0.616, T=0.384 | 0.33748 | 0.0166697 | 0.013-0.021 | 2.00E-17 |
| SmkInit | rs12945403 | Hip circumference adjusted for BMI | 34021172 | chr17:80004094 | C=0.616, T=0.384 | NR | 0.0178205 | 0.012-0.024 | 3.00E-09 |
| SmkInit | rs11873164 | Drinks per week | 36477530 | chr18:42659922 | C=0.869, T=0.131 | 0.156 | 0.00919 | 0.0067-0.0116 | 2.00E-13 |
| SmkInit | rs11873164 | Drinks per week | 36477530 | chr18:42659922 | C=0.869, T=0.131 | 0.145 | 0.00925 | 0.0069-0.0116 | 6.00E-15 |
| SmkInit | rs1945737 | Corneal resistance factor | 33311554 | chr18:53744545 | C=0.515, T=0.485 | 0.47406 | 0.0513574 | 0.033-0.069 | 9.00E-09 |
| SmkInit | rs3213876 | Smoking initiation | 36477530 | chr18:73183978 | C=0.315, T=0.685 | 0.333 | 0.00908 | 0.0073-0.0109 | 4.00E-23 |
| SmkInit | rs3213876 | Smoking initiation | 36477530 | chr18:73183978 | C=0.315, T=0.685 | 0.291 | 0.00894 | 0.0073-0.0106 | 2.00E-25 |
| SmkInit | rs71367545 | Smoking status | 30595370 | chr18:77576337 | A=0.161, G=0.839 | NR | NA | NA | 5.00E-10 |
| SmkInit | rs71367545 | Smoking status (ever vs never smokers) | 30643258 | chr18:77576337 | A=0.161, G=0.839 | 0.2107 | 0.015605067 | 0.01-0.021 | 4.00E-09 |
| SmkInit | rs71367545 | Lifetime smoking index | 31689377 | chr18:77576337 | A=0.161, G=0.839 | 0.791 | 0.015 | 0.011-0.019 | 1.00E-09 |
| SmkInit | rs71367545 | Smoking initiation | 36477530 | chr18:77576337 | A=0.161, G=0.839 | 0.2 | 0.0108 | 0.0087-0.0129 | 2.00E-23 |
| SmkInit | rs71367545 | Smoking initiation | 36477530 | chr18:77576337 | A=0.161, G=0.839 | 0.189 | 0.0107 | 0.0088-0.0126 | 4.00E-27 |
| SmkInit | rs172032 | Breast cancer | 29059683 | chr19:18633755 | C=0.423, T=0.577 | 0.4209 | 0.0531 | 0.041-0.066 | 1.00E-16 |
| SmkInit | rs172032 | Smoking initiation (ever regular vs never regular) (MTAG) | 30643251 | chr19:18633755 | C=0.423, T=0.577 | 0.427 | 0.00677628 | 0.0046-0.0089 | 9.00E-10 |
| SmkInit | rs172032 | Smoking initiation | 36477530 | chr19:18633755 | C=0.423, T=0.577 | 0.426 | 0.00711 | 0.0056-0.0086 | 2.00E-19 |
| SmkInit | rs76608582 | Smoking initiation | 30617275 | chr19:4474725 | A=0.046, C=0.954 | 0.029 | 0.036 | 0.022-0.05() | 9.00E-09 |
| SmkInit | rs76608582 | Educational attainment (years of education) | 30595370 | chr19:4474725 | A=0.046, C=0.954 | NR | NA | NA | 1.00E-09 |
| SmkInit | rs76608582 | Smoking initiation (ever regular vs never regular) | 30679032 | chr19:4474725 | A=0.046, C=0.954 | 0.0482 | 0.0304 | 0.02-0.041 | 1.00E-08 |
| SmkInit | rs76608582 | General cognitive ability | 29844566 | chr19:4474725 | A=0.046, C=0.954 | NR | 4.957 | - | 7.00E-07 |
| SmkInit | rs76608582 | Smoking status | 30595370 | chr19:4474725 | A=0.046, C=0.954 | NR | NA | NA | 2.00E-12 |
| SmkInit | rs76608582 | Intelligence | 29942086 | chr19:4474725 | A=0.046, C=0.954 | NR | 5.57 | - | 3.00E-08 |
| SmkInit | rs76608582 | Smoking initiation (ever regular vs never regular) | 30643251 | chr19:4474725 | A=0.046, C=0.954 | 0.0489 | 0.034549292 | 0.023-0.046 | 5.00E-09 |
| SmkInit | rs76608582 | Smoking status (ever vs never smokers) | 30643258 | chr19:4474725 | A=0.046, C=0.954 | 0.0475 | 0.033407107 | 0.023-0.043 | 6.00E-11 |
| SmkInit | rs76608582 | Lifetime smoking index | 31689377 | chr19:4474725 | A=0.046, C=0.954 | 0.953 | 0.031 | 0.021-0.041 | 3.00E-10 |
| SmkInit | rs76608582 | Smoking status (standard GWA) | 37106081 | chr19:4474725 | A=0.046, C=0.954 | NR | 0.025117 | 0.016-0.034 | 3.00E-08 |
| SmkInit | rs1126757 | Response to antidepressants | 20360315 | chr19:55879872 | C=0.528, T=0.472 | 0.48 | 10.4 | NA | 3.00E-06 |
| SmkInit | rs1126757 | Smoking initiation (ever regular vs never regular) | 30643251 | chr19:55879872 | C=0.528, T=0.472 | 0.473 | 0.014162277 | 0.0092-0.0192 | 3.00E-08 |
| SmkInit | rs1126757 | Smoking initiation (ever regular vs never regular) (MTAG) | 30643251 | chr19:55879872 | C=0.528, T=0.472 | 0.473 | 0.00649966 | 0.0044-0.0086 | 2.00E-09 |
| SmkInit | rs1126757 | Smoking initiation (MTAG) | 37156939 | chr19:55879872 | C=0.528, T=0.472 | NR | 0.00937531 | 0.0062-0.0125 | 5.00E-09 |
| SmkInit | rs10745324 | Smoking initiation (ever regular vs never regular) (MTAG) | 30643251 | chr1:112708722 | A=0.327, G=0.673 | 0.649 | 0.00698752 | 0.0047-0.0093 | 2.00E-09 |
| SmkInit | rs10745324 | Principal component-derived dietary pattern 1 | 32193382 | chr1:112708722 | A=0.327, G=0.673 | 0.352076 | 0.0285444 | 0.019-0.038 | 8.00E-09 |
| SmkInit | rs12027999 | Smoking initiation | 33082346 | chr1:154206358 | C=0.152, T=0.848 | NR | 0.97 | NA | 1.00E-08 |
| SmkInit | rs12027999 | Smoking initiation (ever regular vs never regular) (MTAG) | 30643251 | chr1:154206358 | C=0.152, T=0.848 | 0.12 | 0.011901 | 0.0087-0.0152 | 7.00E-13 |
| SmkInit | rs12027999 | Smoking initiation (ever regular vs never regular) | 30643251 | chr1:154206358 | C=0.152, T=0.848 | 0.12 | 0.024358766 | 0.017-0.032 | 5.00E-10 |
| SmkInit | rs12027999 | Smoking initiation (MTAG) | 37156939 | chr1:154206358 | C=0.152, T=0.848 | NR | 0.0168563 | 0.012-0.022 | 4.00E-12 |
| SmkInit | rs10753630 | Smoking initiation (ever regular vs never regular) (MTAG) | 30643251 | chr1:163790451 | C=0.651, T=0.349 | 0.336 | 0.006137 | 0.0039-0.0084 | 7.00E-08 |
| SmkInit | rs147052174 | Smoking initiation (ever regular vs never regular) | 30679032 | chr1:179783167 | G=0.985, T=0.015 | 0.02 | 0.043 | 0.027-0.059 | 1.00E-07 |
| SmkInit | rs147052174 | Smoking initiation (ever regular vs never regular) (MTAG) | 30643251 | chr1:179783167 | G=0.985, T=0.015 | 0.0171 | 0.0313254 | 0.023-0.04 | 3.00E-13 |
| SmkInit | rs147052174 | Smoking initiation (ever regular vs never regular) | 30643251 | chr1:179783167 | G=0.985, T=0.015 | 0.0171 | 0.062309448 | 0.043-0.082 | 2.00E-10 |
| SmkInit | rs147052174 | Smoking initiation | 36477530 | chr1:179783167 | G=0.985, T=0.015 | 0.017 | 0.0401 | 0.034-0.047 | 7.00E-33 |
| SmkInit | rs147052174 | Smoking initiation | 36477530 | chr1:179783167 | G=0.985, T=0.015 | 0.0168 | 0.0394 | 0.033-0.046 | 2.00E-31 |
| SmkInit | rs55921136 | Smoking initiation (ever regular vs never regular) | 30679032 | chr1:210359333 | C=0.242, T=0.758 | 0.203 | 0.0158 | 0.01-0.021 | 3.00E-08 |
| SmkInit | rs55921136 | Smoking status (ever vs never smokers) | 30643258 | chr1:210359333 | C=0.242, T=0.758 | 0.7966 | 0.015542869 | 0.01-0.021 | 8.00E-09 |
| SmkInit | rs55921136 | Smoking initiation | 36477530 | chr1:210359333 | C=0.242, T=0.758 | 0.203 | 0.0112 | 0.0091-0.0133 | 3.00E-25 |
| SmkInit | rs55921136 | Smoking status (standard GWA) | 37106081 | chr1:210359333 | C=0.242, T=0.758 | NR | 0.01295 | 0.0086-0.0173 | 4.00E-09 |
| SmkInit | rs76132272 | Smoking initiation (ever regular vs never regular) (MTAG) | 30643251 | chr1:227456065 | C=0.944, T=0.056 | 0.0716 | 0.0139991 | 0.0098-0.0182 | 6.00E-11 |
| SmkInit | rs76132272 | Smoking initiation | 36477530 | chr1:227456065 | C=0.944, T=0.056 | 0.0686 | 0.0121 | 0.0089-0.0153 | 1.00E-13 |
| SmkInit | rs4659805 | Externalizing behaviour (multivariate analysis) | 34446935 | chr1:237859755 | G=0.395, T=0.605 | 0.391 | 0.008 | 0.006-0.01 | 2.00E-12 |
| SmkInit | rs4659805 | General risk tolerance (MTAG) | 30643258 | chr1:237859755 | G=0.395, T=0.605 | 0.622 | 0.0072 | 0.0048-0.0096 | 3.00E-09 |
| SmkInit | rs951740 | Externalizing behaviour (multivariate analysis) | 34446935 | chr1:44011737 | A=0.634, G=0.366 | 0.383 | 0.014 | 0.012-0.016 | 5.00E-30 |
| SmkInit | rs951740 | Smoking initiation (ever regular vs never regular) (MTAG) | 30643251 | chr1:44011737 | A=0.634, G=0.366 | 0.625 | 0.0148989 | 0.013-0.017 | 1.00E-40 |
| SmkInit | rs951740 | Smoking initiation (ever regular vs never regular) | 30643251 | chr1:44011737 | A=0.634, G=0.366 | 0.625 | 0.029540867 | 0.024-0.035 | 4.00E-29 |
| SmkInit | rs951740 | Smoking initiation | 36477530 | chr1:44011737 | A=0.634, G=0.366 | 0.625 | 0.017 | 0.015-0.019 | 2.00E-80 |
| SmkInit | rs332827 | Coronary artery disease | 32514122 | chr1:61743160 | A=0.452, G=0.548 | NR | 1.0522777 | 1.03256141696704-1.0723704457929 | 1.00E-07 |
| SmkInit | rs332827 | Externalizing behaviour (multivariate analysis) | 34446935 | chr1:61743160 | A=0.452, G=0.548 | 0.542 | 0.01 | 0.008-0.012 | 3.00E-17 |
| SmkInit | rs332827 | General risk tolerance (MTAG) | 30643258 | chr1:61743160 | A=0.452, G=0.548 | 0.457 | 0.0089 | 0.0065-0.0113 | 5.00E-14 |
| SmkInit | rs1937443 | Smoking initiation (ever regular vs never regular) (MTAG) | 30643251 | chr1:66469643 | C=0.431, G=0.569 | 0.563 | 0.0107674 | 0.0086-0.0129 | 5.00E-23 |
| SmkInit | rs1937443 | Smoking initiation (ever regular vs never regular) | 30643251 | chr1:66469643 | C=0.431, G=0.569 | 0.563 | 0.02043622 | 0.015-0.025 | 2.00E-15 |
| SmkInit | rs12129573 | Schizophrenia | 25056061 | chr1:73768366 | A=0.397, C=0.603 | 0.358 | 1.078 | 1.056-1.101 | 2.00E-12 |
| SmkInit | rs12129573 | Anorexia nervosa, attention-deficit/hyperactivity disorder, autism spectrum disorder, bipolar disorder, major depression, obsessive-compulsive disorder, schizophrenia, or Tourette syndrome (pleiotropy) | 31835028 | chr1:73768366 | A=0.397, C=0.603 | NR | NA | NA | 5.00E-17 |
| SmkInit | rs12129573 | Schizophrenia | 29483656 | chr1:73768366 | A=0.397, C=0.603 | NR | 1.0799136 | 1.06-1.10 | 9.00E-15 |
| SmkInit | rs12129573 | Major depressive disorder or stress-related disorder | 34634379 | chr1:73768366 | A=0.397, C=0.603 | NR | NA | NA | 2.00E-08 |
| SmkInit | rs12129573 | Major depressive disorder | 34634379 | chr1:73768366 | A=0.397, C=0.603 | NR | 1.04896 | 1.03-1.07 | 5.00E-09 |
| SmkInit | rs12129573 | Schizophrenia | 31268507 | chr1:73768366 | A=0.397, C=0.603 | NR | NA | NA | 7.00E-10 |
| SmkInit | rs12129573 | Type 2 diabetes | 35893037 | chr1:73768366 | A=0.397, C=0.603 | NR | NA | NA | 9.00E-21 |
| SmkInit | rs12129573 | Depression | 29700475 | chr1:73768366 | A=0.397, C=0.603 | 0.37 | 1.04 | 1.03-1.05 | 4.00E-12 |
| SmkInit | rs6688826 | Externalizing behaviour (multivariate analysis) | 34446935 | chr1:80812329 | C=0.302, T=0.698 | 0.699 | 0.009 | 0.007-0.011 | 1.00E-13 |
| SmkInit | rs6688826 | Risk-taking behavior (multivariate analysis) | 36324656 | chr1:80812329 | C=0.302, T=0.698 | NR | NA | NA | 4.00E-08 |
| SmkInit | rs11162019 | Smoking initiation (ever regular vs never regular) (MTAG) | 30643251 | chr1:87913176 | C=0.62, T=0.38 | 0.363 | 0.00690692 | 0.0047-0.0091 | 7.00E-10 |
| SmkInit | rs11162019 | Smoking initiation (ever regular vs never regular) | 30643251 | chr1:87913176 | C=0.62, T=0.38 | 0.363 | 0.015494716 | 0.01-0.021 | 5.00E-09 |
| SmkInit | rs12133063 | Noncognitive aspects of educational attainment | 33414549 | chr1:91214714 | A=0.372, C=0.628 | 0.371769 | 0.053425286 | 0.039-0.068 | 2.00E-13 |
| SmkInit | rs6141314 | Cigarettes smoked per day | 36477530 | chr20:31093514 | A=0.218, G=0.782 | 0.26 | 0.0204 | 0.016-0.024 | 3.00E-23 |
| SmkInit | rs6141314 | Insomnia | 35835914 | chr20:31093514 | A=0.218, G=0.782 | NR | 0.009 | 0.007-0.011 | 8.00E-10 |
| SmkInit | rs6141314 | Cigarettes smoked per day | 36477530 | chr20:31093514 | A=0.218, G=0.782 | 0.343 | 0.0156 | 0.012-0.019 | 3.00E-22 |
| SmkInit | rs6088618 | Appendicular lean mass | 31761296 | chr20:33409350 | A=0.432, G=0.568 | 0.43735 | 0.0883923 | 0.069-0.108 | 3.00E-19 |
| SmkInit | rs6088618 | Venous thromboembolism | 31420334 | chr20:33409350 | A=0.432, G=0.568 | NR | 0.0589 | 0.039-0.079 | 5.00E-09 |
| SmkInit | rs6088618 | Smoking initiation | 36477530 | chr20:33409350 | A=0.432, G=0.568 | 0.449 | 0.00732 | 0.0056-0.009 | 9.00E-17 |
| SmkInit | rs6088618 | Educational attainment | 35361970 | chr20:33409350 | A=0.432, G=0.568 | 0.449 | 0.00813085 | 0.0061-0.0102 | 1.00E-14 |
| SmkInit | rs78175438 | Smoking status (ever vs never smokers) | 30643258 | chr21:40663255 | C=0.118, T=0.882 | 0.8713 | 0.018702744 | 0.012-0.025 | 8.00E-09 |
| SmkInit | rs9613472 | Highest math class taken (MTAG) | 30038396 | chr22:27972479 | A=0.457, G=0.543 | 0.4673 | 0.0108 | 0.0075-0.0141 | 6.00E-11 |
| SmkInit | rs9613472 | Alcohol consumption (drinks per week) (MTAG) | 30643251 | chr22:27972479 | A=0.457, G=0.543 | 0.54 | 0.00747007 | 0.0048-0.0101 | 3.00E-08 |
| SmkInit | rs9613472 | Smoking initiation (ever regular vs never regular) (MTAG) | 30643251 | chr22:27972479 | A=0.457, G=0.543 | 0.54 | 0.00700029 | 0.0049-0.0091 | 2.00E-10 |
| SmkInit | rs9613472 | Self-reported math ability (MTAG) | 30038396 | chr22:27972479 | A=0.457, G=0.543 | 0.4679 | 0.0116 | 0.0081-0.0151 | 1.00E-10 |
| SmkInit | rs9613472 | Self-reported math ability | 30038396 | chr22:27972479 | A=0.457, G=0.543 | 0.4679 | 0.0113 | 0.0074-0.0152 | 8.00E-09 |
| SmkInit | rs9613472 | Smoking initiation | 36477530 | chr22:27972479 | A=0.457, G=0.543 | 0.519 | 0.0069 | 0.0054-0.0084 | 2.00E-18 |
| SmkInit | rs134529 | Smoking initiation | 36477530 | chr22:28781758 | C=0.379, T=0.621 | 0.364 | 0.00815 | 0.0064-0.0099 | 1.00E-19 |
| SmkInit | rs134529 | Smoking initiation | 36477530 | chr22:28781758 | C=0.379, T=0.621 | 0.368 | 0.00826 | 0.0067-0.0098 | 1.00E-24 |
| SmkInit | rs5751239 | Body mass index (MTAG) | 36376304 | chr22:42592239 | C=0.472, T=0.528 | NR | 0.009881 | 0.0065-0.0132 | 6.00E-09 |
| SmkInit | rs5751239 | Body mass index | 36581621 | chr22:42592239 | C=0.472, T=0.528 | NR | 0.0119 | 0.009-0.0148 | 6.00E-15 |
| SmkInit | rs5751239 | Body mass index | 37280435 | chr22:42592239 | C=0.472, T=0.528 | NR | 0.0109723 | 0.0072-0.0148 | 4.00E-08 |
| SmkInit | rs9627272 | Smoking initiation (ever regular vs never regular) | 30643251 | chr22:46442288 | C=0.39, G=0.61 | 0.407 | 0.015473696 | 0.01-0.021 | 2.00E-09 |
| SmkInit | rs9627272 | Smoking initiation (ever regular vs never regular) (MTAG) | 30643251 | chr22:46442288 | C=0.39, G=0.61 | 0.407 | 0.00762052 | 0.0054-0.0098 | 7.00E-12 |
| SmkInit | rs9679319 | Problematic alcohol use | 32451486 | chr2:104134432 | G=0.564, T=0.436 | 0.4797 | 6.01 | NA | 2.00E-09 |
| SmkInit | rs6705147 | Smoking initiation | 36477530 | chr2:133196926 | C=0.679, T=0.321 | 0.321 | 0.00661 | 0.0048-0.0084 | 1.00E-12 |
| SmkInit | rs6705147 | Smoking initiation | 36477530 | chr2:133196926 | C=0.679, T=0.321 | 0.321 | 0.006 | 0.0044-0.0076 | 4.00E-13 |
| SmkInit | rs1994247 | Feeling miserable | 29500382 | chr2:156023165 | G=0.485, T=0.515 | 0.479456 | 5.46 | NA | 5.00E-08 |
| SmkInit | rs1994247 | Smoking initiation | 36477530 | chr2:156023165 | G=0.485, T=0.515 | 0.521 | 0.0103 | 0.0088-0.0118 | 2.00E-40 |
| SmkInit | rs11693702 | Smoking status | 30595370 | chr2:162802184 | A=0.417, T=0.583 | NR | NA | NA | 1.00E-11 |
| SmkInit | rs11693702 | Cognitive aspects of educational attainment | 33414549 | chr2:162802184 | A=0.417, T=0.583 | 0.416501 | 0.045211416 | 0.033-0.058 | 1.00E-12 |
| SmkInit | rs11693702 | Cognitive performance | 30038396 | chr2:162802184 | A=0.417, T=0.583 | 0.4612 | 0.0205 | 0.015-0.026 | 9.00E-13 |
| SmkInit | rs13009008 | Lifetime smoking index | 31689377 | chr2:174043233 | A=0.349, G=0.651 | 0.328 | 0.012 | 0.0081-0.0159 | 5.00E-09 |
| SmkInit | rs13009008 | Smoking initiation | 36477530 | chr2:174043233 | A=0.349, G=0.651 | 0.661 | 0.0094 | 0.0076-0.0112 | 9.00E-25 |
| SmkInit | rs13009008 | Smoking initiation | 36477530 | chr2:174043233 | A=0.349, G=0.651 | 0.613 | 0.00905 | 0.0075-0.0106 | 6.00E-30 |
| SmkInit | rs2135160 | Externalizing behaviour (multivariate analysis) | 34446935 | chr2:213214467 | C=0.091, T=0.909 | 0.9 | 0.011 | 0.0071-0.0149 | 2.00E-08 |
| SmkInit | rs61533748 | Bipolar disorder or major depressive disorder | 31926635 | chr2:22582968 | C=0.378, T=0.622 | NR | 1.0309278 | NA | 4.00E-11 |
| SmkInit | rs61533748 | Smoking initiation (ever regular vs never regular) | 30643251 | chr2:22582968 | C=0.378, T=0.622 | 0.384 | 0.017435705 | 0.012-0.023 | 3.00E-11 |
| SmkInit | rs61533748 | Smoking initiation (MTAG) | 37156939 | chr2:22582968 | C=0.378, T=0.622 | NR | 0.01010751 | 0.0069-0.0133 | 1.00E-09 |
| SmkInit | rs2163413 | Age of smoking initiation (MTAG) | 30643251 | chr2:226349200 | A=0.825, G=0.175 | 0.179 | 0.0123293 | 0.0086-0.0161 | 1.00E-10 |
| SmkInit | rs62107261 | Smoking status | 30595370 | chr2:422144 | C=0.045, T=0.955 | NR | NA | NA | 4.00E-08 |
| SmkInit | rs62107261 | Obese vs. thin | 30677029 | chr2:422144 | C=0.045, T=0.955 | 0.9338326 | 1.65 | 1.46-1.87 | 1.00E-15 |
| SmkInit | rs62107261 | Lung function (FEV1/FVC) | 30595370 | chr2:422144 | C=0.045, T=0.955 | NR | NA | NA | 9.00E-11 |
| SmkInit | rs62107261 | Meat-related diet | 32066663 | chr2:422144 | C=0.045, T=0.955 | NR | 0.0435786 | 0.028-0.059 | 2.00E-08 |
| SmkInit | rs62107261 | Type 2 diabetes | 30297969 | chr2:422144 | C=0.045, T=0.955 | 0.9536 | 1.12 | 1.08-1.15 | 4.00E-12 |
| SmkInit | rs62107261 | Body fat distribution (arm fat ratio) | 30664634 | chr2:422144 | C=0.045, T=0.955 | 0.04782 | 0.05549 | () | 5.00E-10 |
| SmkInit | rs62107261 | Body fat distribution (arm fat ratio) | 30664634 | chr2:422144 | C=0.045, T=0.955 | 0.04782 | 0.07675 | () | 2.00E-38 |
| SmkInit | rs62107261 | Body fat distribution (arm fat ratio) | 30664634 | chr2:422144 | C=0.045, T=0.955 | 0.04782 | 0.09445 | () | 1.00E-32 |
| SmkInit | rs62107261 | Body fat distribution (leg fat ratio) | 30664634 | chr2:422144 | C=0.045, T=0.955 | 0.04782 | 0.03319 | () | 3.00E-07 |
| SmkInit | rs62107261 | Body fat distribution (leg fat ratio) | 30664634 | chr2:422144 | C=0.045, T=0.955 | 0.04782 | 0.04719 | () | 2.00E-08 |
| SmkInit | rs62107261 | Weight | 28552196 | chr2:422144 | C=0.045, T=0.955 | 0.9509 | 0.0811 | 0.048-0.114 | 2.00E-06 |
| SmkInit | rs62107261 | Hip circumference | 28552196 | chr2:422144 | C=0.045, T=0.955 | 0.9515 | 0.1012 | 0.058-0.144 | 3.00E-06 |
| SmkInit | rs62107261 | Childhood body mass index | 33045005 | chr2:422144 | C=0.045, T=0.955 | 0.95 | 0.118 | 0.073-0.163 | 3.00E-07 |
| SmkInit | rs62107261 | Hand grip strength | 29691431 | chr2:422144 | C=0.045, T=0.955 | 0.9518 | 0.006 | 0.0046-0.0074 | 8.00E-21 |
| SmkInit | rs62107261 | Metabolic syndrome | 31589552 | chr2:422144 | C=0.045, T=0.955 | 0.0482567 | 0.089776 | 0.059-0.12 | 5.00E-09 |
| SmkInit | rs62107261 | Type 2 diabetes | 35551307 | chr2:422144 | C=0.045, T=0.955 | 0.954 | 0.105 | 0.074-0.136 | 7.00E-11 |
| SmkInit | rs62107261 | Type 2 diabetes | 35551307 | chr2:422144 | C=0.045, T=0.955 | NR | 1.11 | 1.07-1.14 | 5.00E-10 |
| SmkInit | rs62107261 | Drinks per week | 36477530 | chr2:422144 | C=0.045, T=0.955 | 0.0474 | 0.0142 | 0.01-0.018 | 3.00E-11 |
| SmkInit | rs62107261 | Drinks per week | 36477530 | chr2:422144 | C=0.045, T=0.955 | 0.0465 | 0.0137 | 0.0097-0.0177 | 2.00E-11 |
| SmkInit | rs62107261 | Medication use for hypertension (number of purchases) | 36653479 | chr2:422144 | C=0.045, T=0.955 | NR | 0.028591 | 0.021-0.036 | 3.00E-13 |
| SmkInit | rs62107261 | Serum urate levels | 38658550 | chr2:422144 | C=0.045, T=0.955 | NR | 0.0491 | 0.036-0.062 | 3.00E-13 |
| SmkInit | rs62107261 | Smoking initiation | 36477530 | chr2:422144 | C=0.045, T=0.955 | 0.046 | 0.0252 | 0.021-0.029 | 1.00E-37 |
| SmkInit | rs62135525 | Alcohol consumption (drinks per week) | 30643258 | chr2:44299879 | C=0.961, T=0.039 | 0.0495 | 0.0342315 | 0.024-0.044 | 3.00E-11 |
| SmkInit | rs1004787 | Smoking status | 30595370 | chr2:45159091 | A=0.54, G=0.46 | NR | NA | NA | 9.00E-19 |
| SmkInit | rs1004787 | Smoking status (ever vs never smokers) | 32231276 | chr2:45159091 | A=0.54, G=0.46 | 0.5306 | 1.0333781 | NA | 9.00E-11 |
| SmkInit | rs1004787 | Alcohol consumption | 31358974 | chr2:45159091 | A=0.54, G=0.46 | 0.54 | 0.009 | 0.007-0.011 | 7.00E-17 |
| SmkInit | rs1004787 | Smoking status (current vs never) | 33082346 | chr2:45159091 | A=0.54, G=0.46 | NR | 1.052 | NA | 4.00E-08 |
| SmkInit | rs1004787 | Smoking initiation | 33082346 | chr2:45159091 | A=0.54, G=0.46 | NR | 0.967 | NA | 7.00E-22 |
| SmkInit | rs1004787 | Cannabis smoking or cigarette smoking or schizophrenia | 35235886 | chr2:45159091 | A=0.54, G=0.46 | NR | NA | NA | 7.00E-11 |
| SmkInit | rs1004787 | Externalizing behaviour (multivariate analysis) | 34446935 | chr2:45159091 | A=0.54, G=0.46 | 0.467 | 0.014 | 0.012-0.016 | 1.00E-32 |
| SmkInit | rs1004787 | Smoking initiation (ever regular vs never regular) (MTAG) | 30643251 | chr2:45159091 | A=0.54, G=0.46 | 0.552 | 0.0149653 | 0.013-0.017 | 5.00E-43 |
| SmkInit | rs1004787 | Bitter alcoholic beverage consumption | 31046077 | chr2:45159091 | A=0.54, G=0.46 | 0.5328 | 0.0087 | 0.0067-0.0107 | 2.00E-17 |
| SmkInit | rs1004787 | Smoking initiation (ever regular vs never regular) | 30643251 | chr2:45159091 | A=0.54, G=0.46 | 0.552 | 0.028414343 | 0.023-0.033 | 1.00E-28 |
| SmkInit | rs1004787 | Alcohol consumption (drinks per week) | 30643251 | chr2:45159091 | A=0.54, G=0.46 | 0.550918 | 0.008442835 | 0.0056-0.0113 | 8.00E-09 |
| SmkInit | rs1004787 | Age of smoking initiation (MTAG) | 30643251 | chr2:45159091 | A=0.54, G=0.46 | 0.557 | 0.0125114 | 0.0096-0.0154 | 2.00E-17 |
| SmkInit | rs1004787 | Smoking cessation (MTAG) | 30643251 | chr2:45159091 | A=0.54, G=0.46 | 0.558 | 0.0113736 | 0.0083-0.0144 | 3.00E-13 |
| SmkInit | rs1004787 | Alcohol consumption (drinks per week) (MTAG) | 30643251 | chr2:45159091 | A=0.54, G=0.46 | 0.551 | 0.0157003 | 0.013-0.018 | 5.00E-32 |
| SmkInit | rs1004787 | Longevity | 31484785 | chr2:45159091 | A=0.54, G=0.46 | NR | 0.009248 | 0.0052-0.0133 | 6.00E-06 |
| SmkInit | rs1004787 | Smoking status (ever vs never smokers) | 30643258 | chr2:45159091 | A=0.54, G=0.46 | 0.5303 | 0.017661564 | 0.013-0.022 | 4.00E-16 |
| SmkInit | rs1004787 | Vertex-wise cortical thickness | 34910505 | chr2:45159091 | A=0.54, G=0.46 | 0.46 | 6.89 | NA | 6.00E-12 |
| SmkInit | rs1004787 | Horseradish liking | 35585065 | chr2:45159091 | A=0.54, G=0.46 | 0.5293 | 0.050124 | 0.043-0.057 | 4.00E-43 |
| SmkInit | rs1004787 | Educational attainment | 35361970 | chr2:45159091 | A=0.54, G=0.46 | 0.5525 | 0.00875926 | 0.0067-0.0108 | 9.00E-17 |
| SmkInit | rs1004787 | Smoking initiation | 36477530 | chr2:45159091 | A=0.54, G=0.46 | 0.67739 | 0.0176 | 0.012-0.023 | 5.00E-10 |
| SmkInit | rs1004787 | Smoking initiation | 36477530 | chr2:45159091 | A=0.54, G=0.46 | 0.555 | 0.0161 | 0.014-0.018 | 5.00E-76 |
| SmkInit | rs1004787 | Drinks per week | 36477530 | chr2:45159091 | A=0.54, G=0.46 | 0.555 | 0.013 | 0.011-0.015 | 3.00E-46 |
| SmkInit | rs1004787 | Smoking initiation | 36477530 | chr2:45159091 | A=0.54, G=0.46 | 0.61 | 0.0154 | 0.014-0.017 | 4.00E-84 |
| SmkInit | rs7585579 | Smoking initiation (ever regular vs never regular) | 30679032 | chr2:60024857 | C=0.482, G=0.518 | 0.489 | 0.0157 | 0.011-0.02 | 6.00E-12 |
| SmkInit | rs7585579 | Smoking initiation (ever regular vs never regular) | 30643251 | chr2:60024857 | C=0.482, G=0.518 | 0.499 | 0.020396171 | 0.015-0.026 | 5.00E-15 |
| SmkInit | rs7585579 | Smoking status (ever vs never smokers) | 30643258 | chr2:60024857 | C=0.482, G=0.518 | 0.5061 | 0.013979038 | 0.01-0.018 | 3.00E-12 |
| SmkInit | rs7585579 | Risk-taking tendency (4-domain principal component model) | 30643258 | chr2:60024857 | C=0.482, G=0.518 | 0.5087 | 0.014212011 | 0.0092-0.0193 | 3.00E-08 |
| SmkInit | rs7585579 | Risk-taking behavior (multivariate analysis) | 36324656 | chr2:60024857 | C=0.482, G=0.518 | NR | NA | NA | 6.00E-15 |
| SmkInit | rs6728726 | Smoking initiation | 33082346 | chr2:623976 | C=0.83, T=0.17 | NR | 0.967 | NA | 6.00E-13 |
| SmkInit | rs6728726 | Childhood body fatness | 33491310 | chr2:623976 | C=0.83, T=0.17 | NR | 11.527 | NA | 2.00E-12 |
| SmkInit | rs6728726 | Lifetime smoking | 34855049 | chr2:623976 | C=0.83, T=0.17 | NR | NA | NA | 2.00E-12 |
| SmkInit | rs6728726 | Lifetime smoking (without educational attainment) | 34855049 | chr2:623976 | C=0.83, T=0.17 | NR | NA | NA | 4.00E-10 |
| SmkInit | rs12714017 | Smoking initiation (ever regular vs never regular) | 30643251 | chr2:80999398 | C=0.534, T=0.466 | 0.511 | 0.015396497 | 0.01-0.021 | 4.00E-09 |
| SmkInit | rs11695197 | Cortical surface area | 34560273 | chr2:97711421 | A=0.111, G=0.889 | 0.1036 | NA | NA | 6.00E-13 |
| SmkInit | rs326341 | Smoking status | 30595370 | chr3:107811142 | A=0.462, G=0.538 | NR | NA | NA | 6.00E-08 |
| SmkInit | rs326341 | Lifetime smoking index | 31689377 | chr3:107811142 | A=0.462, G=0.538 | 0.525 | 0.014 | 0.01-0.018 | 1.00E-11 |
| SmkInit | rs326341 | Risk-taking behavior (multivariate analysis) | 36324656 | chr3:107811142 | A=0.462, G=0.538 | NR | NA | NA | 2.00E-08 |
| SmkInit | rs62266876 | Body mass index | 36581621 | chr3:128976451 | C=0.927, G=0.073 | NR | 0.0125 | 0.008-0.017 | 4.00E-08 |
| SmkInit | rs62266876 | Smoking initiation | 36477530 | chr3:128976451 | C=0.927, G=0.073 | 0.0931 | 0.011 | 0.0083-0.0137 | 2.00E-15 |
| SmkInit | rs114050142 | Smoking initiation (ever regular vs never regular) (MTAG) | 30643251 | chr3:150805177 | A=0.062, T=0.938 | 0.0567 | 0.0172834 | 0.012-0.022 | 5.00E-12 |
| SmkInit | rs114050142 | Smoking initiation | 36477530 | chr3:150805177 | A=0.062, T=0.938 | 0.0568 | 0.0178 | 0.014-0.021 | 2.00E-21 |
| SmkInit | rs114050142 | Smoking initiation | 36477530 | chr3:150805177 | A=0.062, T=0.938 | 0.0536 | 0.0172 | 0.014-0.021 | 2.00E-21 |
| SmkInit | rs963354 | Smoking initiation (ever regular vs never regular) | 30679032 | chr3:157393770 | A=0.705, C=0.295 | 0.672 | 0.0141 | 0.0093-0.0189 | 7.00E-09 |
| SmkInit | rs963354 | Smoking initiation (ever regular vs never regular) | 30643251 | chr3:157393770 | A=0.705, C=0.295 | 0.687 | 0.015048863 | 0.0097-0.0204 | 4.00E-08 |
| SmkInit | rs963354 | Lifetime smoking | 34855049 | chr3:157393770 | A=0.705, C=0.295 | NR | NA | NA | 5.00E-10 |
| SmkInit | rs963354 | Lifetime smoking (without educational attainment) | 34855049 | chr3:157393770 | A=0.705, C=0.295 | NR | NA | NA | 5.00E-09 |
| SmkInit | rs963354 | Smoking initiation | 36477530 | chr3:157393770 | A=0.705, C=0.295 | 0.702 | 0.00904 | 0.0074-0.0107 | 2.00E-26 |
| SmkInit | rs7629352 | Smoking initiation | 36477530 | chr3:16848835 | A=0.705, G=0.295 | 0.306 | 0.00887 | 0.007-0.0107 | 4.00E-21 |
| SmkInit | rs12485391 | Smoking status | 30595370 | chr3:181205593 | A=0.124, C=0.876 | NR | NA | NA | 1.00E-07 |
| SmkInit | rs1485272 | Smoking initiation (ever regular vs never regular) (MTAG) | 30643251 | chr3:3727589 | C=0.377, T=0.623 | 0.346 | 0.00657202 | 0.0043-0.0088 | 7.00E-09 |
| SmkInit | rs1485272 | Educational attainment | 35361970 | chr3:3727589 | C=0.377, T=0.623 | 0.6535 | 0.00718106 | 0.005-0.0093 | 7.00E-11 |
| SmkInit | rs12632110 | Smoking initiation | 33082346 | chr3:50224225 | A=0.337, G=0.663 | NR | 1.024 | NA | 1.00E-10 |
| SmkInit | rs12632110 | Smoking initiation (MTAG) | 37156939 | chr3:50224225 | A=0.337, G=0.663 | NR | 0.01208179 | 0.0088-0.0154 | 6.00E-13 |
| SmkInit | rs11130381 | Cardiovascular disease | 30595370 | chr3:53850005 | C=0.474, T=0.526 | NR | NA | NA | 2.00E-13 |
| SmkInit | rs11130381 | Drinks per week | 36477530 | chr3:53850005 | C=0.474, T=0.526 | 0.53 | 0.00538 | 0.0038-0.007 | 6.00E-11 |
| SmkInit | rs4479577 | Smoking initiation (ever regular vs never regular) | 30679032 | chr3:5723818 | C=0.501, T=0.499 | 0.482 | 0.0142 | 0.0097-0.0187 | 5.00E-10 |
| SmkInit | rs4479577 | Smoking status | 30595370 | chr3:5723818 | C=0.501, T=0.499 | NR | NA | NA | 8.00E-09 |
| SmkInit | rs4479577 | Externalizing behaviour (multivariate analysis) | 34446935 | chr3:5723818 | C=0.501, T=0.499 | 0.516 | 0.007 | 0.005-0.009 | 7.00E-09 |
| SmkInit | rs4479577 | Smoking status (ever vs never smokers) | 30643258 | chr3:5723818 | C=0.501, T=0.499 | 0.4813 | 0.012443462 | 0.0082-0.0167 | 1.00E-08 |
| SmkInit | rs11720703 | Smoking initiation | 33082346 | chr3:71060640 | C=0.494, T=0.506 | NR | 1.02 | NA | 1.00E-08 |
| SmkInit | rs11720703 | Externalizing behaviour (multivariate analysis) | 34446935 | chr3:71060640 | C=0.494, T=0.506 | 0.471 | 0.013 | 0.011-0.015 | 3.00E-27 |
| SmkInit | rs11720703 | Body mass index | 36581621 | chr3:71060640 | C=0.494, T=0.506 | NR | 0.0083 | 0.0054-0.0112 | 4.00E-08 |
| SmkInit | rs11720703 | Smoking initiation (MTAG) | 37156939 | chr3:71060640 | C=0.494, T=0.506 | NR | 0.00952862 | 0.0064-0.0127 | 3.00E-09 |
| SmkInit | rs62250713 | Feeling worry | 29500382 | chr3:85513793 | A=0.367, G=0.633 | 0.375418 | 8.411 | NA | 4.00E-17 |
| SmkInit | rs62250713 | Problematic alcohol use | 32451486 | chr3:85513793 | A=0.367, G=0.633 | 0.368 | 6.049 | NA | 1.00E-09 |
| SmkInit | rs62250713 | Problematic alcohol use (MTAG) | 36301540 | chr3:85513793 | A=0.367, G=0.633 | NR | 0.0137 | 0.0092-0.0182 | 2.00E-09 |
| SmkInit | rs62250713 | Substance use disorder (pleiotropy) | 37250466 | chr3:85513793 | A=0.367, G=0.633 | NR | 0.009 | NA | 1.00E-18 |
| SmkInit | rs62250713 | Cannabis use disorder (MTAG) | 37156939 | chr3:85513793 | A=0.367, G=0.633 | NR | 0.02623935 | 0.02-0.032 | 4.00E-17 |
| SmkInit | rs62250713 | Opioid use disorder (MTAG) | 37156939 | chr3:85513793 | A=0.367, G=0.633 | NR | 0.02516404 | 0.018-0.032 | 5.00E-13 |
| SmkInit | rs62250713 | Alcohol use disorder (MTAG) | 37156939 | chr3:85513793 | A=0.367, G=0.633 | NR | 0.0195951 | 0.014-0.025 | 3.00E-13 |
| SmkInit | rs62250713 | Smoking initiation (MTAG) | 37156939 | chr3:85513793 | A=0.367, G=0.633 | NR | 0.01600516 | 0.013-0.019 | 6.00E-22 |
| SmkInit | rs3934797 | Smoking initiation (ever regular vs never regular) | 30643251 | chr4:112467612 | A=0.172, G=0.828 | 0.182 | 0.021297466 | 0.015-0.028 | 1.00E-10 |
| SmkInit | rs3934797 | Cannabis use disorder (MTAG) | 37156939 | chr4:112467612 | A=0.172, G=0.828 | NR | 0.0259288 | 0.018-0.034 | 8.00E-11 |
| SmkInit | rs3934797 | Opioid use disorder (MTAG) | 37156939 | chr4:112467612 | A=0.172, G=0.828 | NR | 0.0300055 | 0.021-0.039 | 1.00E-11 |
| SmkInit | rs3934797 | Alcohol use disorder (MTAG) | 37156939 | chr4:112467612 | A=0.172, G=0.828 | NR | 0.024819 | 0.018-0.032 | 3.00E-12 |
| SmkInit | rs3934797 | Smoking initiation (MTAG) | 37156939 | chr4:112467612 | A=0.172, G=0.828 | NR | 0.0143613 | 0.01-0.019 | 2.00E-11 |
| SmkInit | rs10001365 | Externalizing behaviour (multivariate analysis) | 34446935 | chr4:147797214 | A=0.379, G=0.621 | 0.614 | 0.011 | 0.009-0.013 | 3.00E-19 |
| SmkInit | rs10001365 | Smoking initiation (ever regular vs never regular) (MTAG) | 30643251 | chr4:147797214 | A=0.379, G=0.621 | 0.394 | 0.0125846 | 0.01-0.015 | 3.00E-30 |
| SmkInit | rs10001365 | Age of smoking initiation (MTAG) | 30643251 | chr4:147797214 | A=0.379, G=0.621 | 0.39 | 0.0124203 | 0.0095-0.0153 | 9.00E-17 |
| SmkInit | rs10001365 | Lifetime smoking (without educational attainment) | 34855049 | chr4:147797214 | A=0.379, G=0.621 | NR | NA | NA | 1.00E-08 |
| SmkInit | rs10001365 | Smoking initiation (MTAG) | 37156939 | chr4:147797214 | A=0.379, G=0.621 | NR | 0.0121895 | 0.009-0.0154 | 8.00E-14 |
| SmkInit | rs6852117 | Smoking status | 30595370 | chr4:173076888 | C=0.598, G=0.402 | NR | NA | NA | 6.00E-08 |
| SmkInit | rs6852117 | Smoking status (ever vs never smokers) | 30643258 | chr4:173076888 | C=0.598, G=0.402 | 0.5802 | 0.01401889 | 0.01-0.018 | 6.00E-12 |
| SmkInit | rs12642744 | Smoking initiation (ever regular vs never regular) | 30643251 | chr4:28027176 | G=0.243, T=0.757 | 0.744 | 0.016590752 | 0.011-0.022 | 3.00E-08 |
| SmkInit | rs12642744 | Smoking initiation | 36477530 | chr4:28027176 | G=0.243, T=0.757 | 0.745 | 0.0117 | 0.0097-0.0137 | 2.00E-31 |
| SmkInit | rs58400863 | Smoking status | 30595370 | chr4:31184484 | A=0.388, G=0.612 | NR | NA | NA | 5.00E-10 |
| SmkInit | rs58400863 | Smoking initiation (ever regular vs never regular) (MTAG) | 30643251 | chr4:31184484 | A=0.388, G=0.612 | 0.347 | 0.00989187 | 0.0076-0.0121 | 5.00E-18 |
| SmkInit | rs58400863 | Smoking initiation (ever regular vs never regular) | 30643251 | chr4:31184484 | A=0.388, G=0.612 | 0.347 | 0.020171883 | 0.015-0.025 | 5.00E-14 |
| SmkInit | rs58400863 | General risk tolerance (MTAG) | 30643258 | chr4:31184484 | A=0.388, G=0.612 | 0.341 | 0.01 | 0.0076-0.0124 | 1.00E-15 |
| SmkInit | rs58400863 | Smoking initiation | 36477530 | chr4:31184484 | A=0.388, G=0.612 | 0.348 | 0.0131 | 0.011-0.015 | 7.00E-47 |
| SmkInit | rs58400863 | Smoking initiation | 36477530 | chr4:31184484 | A=0.388, G=0.612 | 0.315 | 0.0122 | 0.011-0.014 | 4.00E-47 |
| SmkInit | rs993700 | Smoking initiation | 33082346 | chr4:67825894 | C=0.767, T=0.233 | NR | 1.026 | NA | 4.00E-10 |
| SmkInit | rs993700 | Age at first sexual intercourse | 34211149 | chr4:67825894 | C=0.767, T=0.233 | 0.22252 | 0.0157496 | 0.011-0.021 | 3.00E-10 |
| SmkInit | rs1503211 | Height | 36224396 | chr4:94079508 | A=0.454, G=0.546 | 0.4995 | 0.0069 | 0.0057-0.0081 | 9.00E-28 |
| SmkInit | rs288181 | Major depressive disorder (broad) | 38177345 | chr5:107349285 | C=0.682, T=0.318 | 0.331 | 0.0188 | 0.013-0.025 | 6.00E-09 |
| SmkInit | rs288181 | Depression | 37464041 | chr5:107349285 | C=0.682, T=0.318 | 0.670475 | 0.0165031 | 0.011-0.022 | 4.00E-08 |
| SmkInit | rs2313500 | Smoking initiation | 36477530 | chr5:154808532 | C=0.755, T=0.245 | 0.245 | 0.0101 | 0.0081-0.0121 | 7.00E-24 |
| SmkInit | rs2237303 | Smoking initiation | 36477530 | chr7:21483605 | A=0.656, G=0.344 | 0.651 | 0.0062 | 0.0044-0.008 | 9.00E-12 |
| SmkInit | rs12112638 | Cognitive aspects of educational attainment | 33414549 | chr7:69735251 | A=0.766, G=0.234 | 0.233598 | 0.049299136 | 0.035-0.064 | 3.00E-11 |
| SmkInit | rs12112638 | Cognitive performance | 30038396 | chr7:69735251 | A=0.766, G=0.234 | 0.7366 | 0.0216 | 0.015-0.028 | 2.00E-11 |
| SmkInit | rs3801289 | Externalizing behaviour (multivariate analysis) | 34446935 | chr7:96638267 | A=0.636, C=0.364 | 0.663 | 0.007 | 0.005-0.009 | 3.00E-09 |
| SmkInit | rs3801289 | Smoking initiation | 36477530 | chr7:96638267 | A=0.636, C=0.364 | 0.35 | 0.0111 | 0.0093-0.0129 | 3.00E-34 |
| SmkInit | rs3801289 | Smoking initiation | 36477530 | chr7:96638267 | A=0.636, C=0.364 | 0.328 | 0.0108 | 0.0092-0.0124 | 4.00E-39 |
| SmkInit | rs7804551 | Body mass index | 36329257 | chr7:99119110 | A=0.837, G=0.163 | NR | 0.110561 | NA | 1.00E-08 |
| SmkInit | rs7804551 | Body fat percentage | 36329257 | chr7:99119110 | A=0.837, G=0.163 | NR | 0.20657 | NA | 5.00E-11 |
| SmkInit | rs7804551 | Smoking initiation | 36477530 | chr7:99119110 | A=0.837, G=0.163 | 0.182 | 0.0139 | 0.012-0.016 | 2.00E-42 |
| SmkInit | rs1565735 | Smoking initiation (ever regular vs never regular) | 30679032 | chr8:27426077 | A=0.2, T=0.8 | 0.201 | 0.0235 | 0.018-0.029 | 2.00E-16 |
| SmkInit | rs1565735 | Smoking status | 30595370 | chr8:27426077 | A=0.2, T=0.8 | NR | NA | NA | 9.00E-28 |
| SmkInit | rs1565735 | Smoking initiation | 33082346 | chr8:27426077 | A=0.2, T=0.8 | NR | 0.961 | NA | 2.00E-20 |
| SmkInit | rs1565735 | Smoking cessation (MTAG) | 30643251 | chr8:27426077 | A=0.2, T=0.8 | 0.199 | 0.0167706 | 0.013-0.021 | 3.00E-18 |
| SmkInit | rs1565735 | Smoking cessation | 30643251 | chr8:27426077 | A=0.2, T=0.8 | 0.199 | 0.034557395 | 0.025-0.044 | 2.00E-12 |
| SmkInit | rs1565735 | Smoking initiation (ever regular vs never regular) | 30643251 | chr8:27426077 | A=0.2, T=0.8 | 0.204463 | 0.019163726 | 0.013-0.025 | 1.00E-09 |
| SmkInit | rs1565735 | Lifetime smoking | 34855049 | chr8:27426077 | A=0.2, T=0.8 | NR | NA | NA | 2.00E-22 |
| SmkInit | rs1565735 | Lifetime smoking (without educational attainment) | 34855049 | chr8:27426077 | A=0.2, T=0.8 | NR | NA | NA | 1.00E-17 |
| SmkInit | rs1565735 | Smoking cessation | 36477530 | chr8:27426077 | A=0.2, T=0.8 | 0.201 | 0.0195 | 0.016-0.023 | 2.00E-32 |
| SmkInit | rs1565735 | Cigarettes smoked per day | 36477530 | chr8:27426077 | A=0.2, T=0.8 | 0.2 | 0.0189 | 0.014-0.023 | 4.00E-17 |
| SmkInit | rs1565735 | Smoking cessation | 36477530 | chr8:27426077 | A=0.2, T=0.8 | 0.213 | 0.0174 | 0.015-0.02 | 1.00E-33 |
| SmkInit | rs1565735 | Cigarettes smoked per day | 36477530 | chr8:27426077 | A=0.2, T=0.8 | 0.218 | 0.0219 | 0.018-0.026 | 5.00E-30 |
| SmkInit | rs1565735 | Height | 36224396 | chr8:27426077 | A=0.2, T=0.8 | 0.2185 | 0.0057 | 0.0041-0.0073 | 2.00E-12 |
| SmkInit | rs7829715 | Smoking initiation | 33082346 | chr8:59803836 | C=0.561, T=0.439 | NR | 1.025 | NA | 6.00E-13 |
| SmkInit | rs7829715 | Smoking initiation | 36477530 | chr8:59803836 | C=0.561, T=0.439 | 0.521 | 0.00836 | 0.0067-0.0101 | 5.00E-22 |
| SmkInit | rs6472232 | Mean corpuscular volume | 32888493 | chr8:66792632 | G=0.375, T=0.625 | 0.324905 | NA | NA | 1.00E-16 |
| SmkInit | rs6472232 | Mean corpuscular volume | 32888494 | chr8:66792632 | G=0.375, T=0.625 | 0.3515 | 0.0151683 | 0.011-0.019 | 4.00E-11 |
| SmkInit | rs6472232 | Mean corpuscular volume | 32888493 | chr8:66792632 | G=0.375, T=0.625 | 0.354316 | 0.014987 | 0.011-0.019 | 4.00E-15 |
| SmkInit | rs6472232 | Mean corpuscular volume | 34594039 | chr8:66792632 | G=0.375, T=0.625 | NR | 0.0174 | 0.013-0.022 | 2.00E-14 |
| SmkInit | rs9987376 | Smoking initiation (ever regular vs never regular) (MTAG) | 30643251 | chr8:93190014 | G=0.57, T=0.43 | 0.574 | 0.00888939 | 0.0068-0.011 | 4.00E-16 |
| SmkInit | rs9987376 | Smoking initiation (ever regular vs never regular) | 30643251 | chr8:93190014 | G=0.57, T=0.43 | 0.574251 | 0.020468244 | 0.015-0.026 | 2.00E-15 |
| SmkInit | rs9987376 | Cortical surface area | 34560273 | chr8:93190014 | G=0.57, T=0.43 | 0.4204 | NA | NA | 1.00E-17 |
| SmkInit | rs9987376 | Vertex-wise cortical surface area | 34910505 | chr8:93190014 | G=0.57, T=0.43 | 0.43 | 8.89 | NA | 6.00E-19 |
| SmkInit | rs9987376 | Vertex-wise cortical surface area | 34910505 | chr8:93190014 | G=0.57, T=0.43 | 0.43 | NA | NA | 6.00E-06 |
| SmkInit | rs9987376 | Smoking initiation | 36477530 | chr8:93190014 | G=0.57, T=0.43 | 0.573 | 0.0102 | 0.0085-0.0119 | 2.00E-31 |
| SmkInit | rs10121930 | Smoking initiation | 36477530 | chr9:11175974 | A=0.509, T=0.491 | 0.506 | 0.00951 | 0.008-0.011 | 1.00E-34 |
| SmkInit | rs4837631 | Smoking status | 30595370 | chr9:122061948 | C=0.541, T=0.459 | NR | NA | NA | 3.00E-09 |
| SmkInit | rs4837631 | Externalizing behaviour (multivariate analysis) | 34446935 | chr9:122061948 | C=0.541, T=0.459 | 0.55 | 0.008 | 0.006-0.01 | 2.00E-11 |
| SmkInit | rs4837631 | Smoking initiation (ever regular vs never regular) (MTAG) | 30643251 | chr9:122061948 | C=0.541, T=0.459 | 0.446 | 0.00733822 | 0.0052-0.0095 | 1.00E-11 |
| SmkInit | rs4837631 | Smoking initiation (ever regular vs never regular) | 30643251 | chr9:122061948 | C=0.541, T=0.459 | 0.446 | 0.015357429 | 0.01-0.02 | 2.00E-09 |
| SmkInit | rs4837631 | Smoking status (ever vs never smokers) | 30643258 | chr9:122061948 | C=0.541, T=0.459 | 0.4517 | 0.012634879 | 0.0084-0.0169 | 7.00E-09 |
| SmkInit | rs4837631 | Smoking initiation | 36477530 | chr9:122061948 | C=0.541, T=0.459 | 0.444 | 0.00797 | 0.0063-0.0097 | 9.00E-20 |
| SmkInit | rs4837631 | Smoking initiation | 36477530 | chr9:122061948 | C=0.541, T=0.459 | 0.436 | 0.00726 | 0.0057-0.0088 | 4.00E-20 |
| SmkInit | rs11103667 | Smoking status | 30595370 | chr9:137978360 | C=0.82, T=0.18 | NR | NA | NA | 5.00E-09 |
| SmkInit | rs11103667 | Blood protein levels | 30072576 | chr9:137978360 | C=0.82, T=0.18 | 0.81484375 | 0.1932553 | 0.13-0.26 | 3.00E-09 |
| SmkInit | rs10119117 | Externalizing behaviour (multivariate analysis) | 34446935 | chr9:29740028 | C=0.502, T=0.498 | 0.481 | 0.008 | 0.006-0.01 | 1.00E-10 |
| SmkInit | rs4543592 | Smoking status | 30595370 | chr9:3014254 | C=0.491, T=0.509 | NR | NA | NA | 2.00E-11 |
| SmkInit | rs4543592 | Smoking initiation | 33082346 | chr9:3014254 | C=0.491, T=0.509 | NR | 1.019 | NA | 2.00E-08 |
| SmkInit | rs4543592 | Smoking status (ever vs never smokers) | 30643258 | chr9:3014254 | C=0.491, T=0.509 | 0.5205 | 0.012717051 | 0.0088-0.0167 | 3.00E-10 |
| SmkInit | rs4543592 | Lifetime smoking index | 31689377 | chr9:3014254 | C=0.491, T=0.509 | 0.52 | 0.012 | 0.0081-0.0159 | 5.00E-10 |
| SmkInit | rs4543592 | Smoking initiation | 36477530 | chr9:3014254 | C=0.491, T=0.509 | 0.476 | 0.0111 | 0.0094-0.0128 | 3.00E-37 |
| SmkInit | rs4543592 | Smoking initiation | 36477530 | chr9:3014254 | C=0.491, T=0.509 | 0.448 | 0.00985 | 0.0083-0.0114 | 1.00E-35 |
| SmkInit | rs72733235 | Smoking status | 30595370 | chr9:38275772 | C=0.178, T=0.822 | NR | NA | NA | 1.00E-08 |
| SmkInit | rs72733235 | Smoking status (ever vs never smokers) | 30643258 | chr9:38275772 | C=0.178, T=0.822 | 0.8378 | 0.01716322 | 0.011-0.023 | 5.00E-09 |
| SmkInit | rs72733235 | Smoking initiation | 36477530 | chr9:38275772 | C=0.178, T=0.822 | 0.147 | 0.00707 | 0.0049-0.0093 | 6.00E-11 |
| SmkInit | rs1246265 | Cannabis smoking or cigarette smoking or schizophrenia | 35235886 | chr9:86761745 | C=0.702, T=0.298 | NR | NA | NA | 4.00E-08 |
| SmkInit | rs1246265 | Smoking status (ever vs never smokers) | 30643258 | chr9:86761745 | C=0.702, T=0.298 | 0.3046 | 0.015632885 | 0.011-0.02 | 3.00E-11 |
| SmkInit | rs1246265 | Lifetime smoking index | 31689377 | chr9:86761745 | C=0.702, T=0.298 | 0.305 | 0.013 | 0.0091-0.0169 | 4.00E-09 |
| SmkInit | rs1246265 | Smoking initiation | 36477530 | chr9:86761745 | C=0.702, T=0.298 | 0.696 | 0.00839 | 0.0068-0.01 | 3.00E-23 |
| SmkInit | rs4044321 | Smoking initiation | 33082346 | chr5:166989513 | A=0.331, G=0.669 | NR | 1.029 | NA | 2.00E-15 |
| SmkInit | rs4044321 | Age of smoking initiation (MTAG) | 30643251 | chr5:166989513 | A=0.331, G=0.669 | 0.64 | 0.00924222 | 0.0063-0.0122 | 1.00E-09 |
| SmkInit | rs4044321 | Smoking initiation (ever regular vs never regular) (MTAG) | 30643251 | chr5:166989513 | A=0.331, G=0.669 | 0.644 | 0.011401 | 0.0092-0.0136 | 4.00E-24 |
| SmkInit | rs4044321 | Smoking initiation (ever regular vs never regular) | 30643251 | chr5:166989513 | A=0.331, G=0.669 | 0.644 | 0.022640966 | 0.017-0.028 | 2.00E-17 |
| SmkInit | rs4044321 | Lifetime smoking | 34855049 | chr5:166989513 | A=0.331, G=0.669 | NR | NA | NA | 7.00E-14 |
| SmkInit | rs4044321 | Lifetime smoking (without educational attainment) | 34855049 | chr5:166989513 | A=0.331, G=0.669 | NR | NA | NA | 2.00E-12 |
| SmkInit | rs2173019 | Smoking initiation (ever regular vs never regular) (MTAG) | 30643251 | chr5:167614971 | A=0.172, T=0.828 | 0.177 | 0.0119549 | 0.0092-0.0147 | 3.00E-17 |
| SmkInit | rs2173019 | Smoking initiation (ever regular vs never regular) | 30643251 | chr5:167614971 | A=0.172, T=0.828 | 0.177 | 0.028206952 | 0.022-0.035 | 3.00E-17 |
| SmkInit | rs2173019 | Smoking initiation | 36477530 | chr5:167614971 | A=0.172, T=0.828 | 0.198 | 0.0104 | 0.0085-0.0123 | 4.00E-29 |
| SmkInit | rs1392446 | Catenin beta-1 levels | 34648354 | chr5:30831387 | C=0.467, T=0.533 | 0.55 | 0.857 | 0.84-0.88 | 0 |
| SmkInit | rs71627577 | Smoking initiation | 36477530 | chr5:43125795 | A=0.882, G=0.118 | 0.109 | 0.0156 | 0.013-0.018 | 2.00E-29 |
| SmkInit | rs71627577 | Smoking initiation | 36477530 | chr5:43125795 | A=0.882, G=0.118 | 0.103 | 0.0148 | 0.012-0.017 | 2.00E-28 |
| SmkInit | rs4571506 | Smoking initiation | 33082346 | chr5:87756918 | C=0.519, T=0.481 | NR | 0.978 | NA | 9.00E-11 |
| SmkInit | rs4571506 | Lifetime smoking index | 31689377 | chr5:87756918 | C=0.519, T=0.481 | 0.54 | 0.011 | 0.0071-0.0149 | 2.00E-08 |
| SmkInit | rs4571506 | Smoking initiation | 36477530 | chr5:87756918 | C=0.519, T=0.481 | 0.472 | 0.0153 | 0.014-0.017 | 8.00E-70 |
| SmkInit | rs4571506 | Smoking initiation | 36477530 | chr5:87756918 | C=0.519, T=0.481 | 0.476 | 0.0128 | 0.011-0.014 | 2.00E-67 |
| SmkInit | rs846781 | Smoking initiation | 36477530 | chr6:101280434 | C=0.738, T=0.262 | 0.721 | 0.0112 | 0.0095-0.0129 | 7.00E-40 |
| SmkInit | rs557544 | Reaction time | 29844566 | chr6:109020332 | C=0.357, T=0.643 | NR | 0.010194 | 0.0067-0.0136 | 7.00E-09 |
| SmkInit | rs557544 | Intelligence (MTAG) | 29326435 | chr6:109020332 | C=0.357, T=0.643 | NR | 0.017957335 | 0.012-0.024 | 5.00E-08 |
| SmkInit | rs465646 | Smoking status (ever vs never smokers) | 32231276 | chr6:111620758 | A=0.85, G=0.15 | 0.1588 | 1.055 | 1.04-1.07 | 8.00E-15 |
| SmkInit | rs9402093 | Externalizing behaviour (multivariate analysis) | 34446935 | chr6:129353671 | G=0.307, T=0.693 | 0.3 | 0.009 | 0.007-0.011 | 2.00E-12 |
| SmkInit | rs2876586 | Smoking initiation (ever regular vs never regular) (MTAG) | 30643251 | chr6:144862998 | A=0.408, G=0.592 | 0.405 | 0.00617405 | 0.004-0.0083 | 2.00E-08 |
| SmkInit | rs10698713 | Smoking initiation (ever regular vs never regular) | 30643251 | chr6:158882320 | A=0.053, G=0.947 | 0.0544 | 0.03351702 | 0.023-0.045 | 2.00E-09 |
| SmkInit | rs10698713 | Smoking initiation | 36477530 | chr6:158882320 | A=0.053, G=0.947 | 0.0527 | 0.0138 | 0.01-0.017 | 2.00E-14 |
| SmkInit | rs79222572 | Major depressive disorder (broad) | 38177345 | chr6:165108555 | G=0.255, T=0.745 | 0.732 | 0.0239 | 0.017-0.031 | 1.00E-11 |
| SmkInit | rs160631 | Externalizing behaviour (multivariate analysis) | 34446935 | chr6:52895230 | G=0.729, T=0.271 | 0.261 | 0.01 | 0.008-0.012 | 1.00E-13 |
| SmkInit | rs160631 | Smoking initiation (ever regular vs never regular) | 30643251 | chr6:52895230 | G=0.729, T=0.271 | 0.731 | 0.017262844 | 0.012-0.023 | 2.00E-09 |
| SmkInit | rs146628116 | Smoking initiation | 36477530 | chr6:67551428 | A=0.371, T=0.629 | 0.392 | 0.0123 | 0.01-0.014 | 2.00E-40 |
| SmkInit | rs146628116 | Smoking initiation | 36477530 | chr6:67551428 | A=0.371, T=0.629 | 0.357 | 0.0113 | 0.0097-0.0129 | 6.00E-42 |
| SmkInit | rs10233018 | Smoking initiation (ever regular vs never regular) | 30679032 | chr7:117523709 | A=0.487, G=0.513 | 0.504 | 0.015 | 0.011-0.019 | 6.00E-11 |
| SmkInit | rs10233018 | Smoking status | 30595370 | chr7:117523709 | A=0.487, G=0.513 | NR | NA | NA | 7.00E-15 |
| SmkInit | rs10233018 | Depression | 27089181 | chr7:117523709 | A=0.487, G=0.513 | NR | 5.3463354 | NA | 9.00E-08 |
| SmkInit | rs10233018 | Smoking initiation | 33082346 | chr7:117523709 | A=0.487, G=0.513 | NR | 0.972 | NA | 5.00E-16 |
| SmkInit | rs10233018 | Depressive symptoms | 29292387 | chr7:117523709 | A=0.487, G=0.513 | 0.4886 | 0.0138 | 0.0091-0.0185 | 6.00E-09 |
| SmkInit | rs10233018 | Age of smoking initiation (MTAG) | 30643251 | chr7:117523709 | A=0.487, G=0.513 | 0.522 | 0.010904 | 0.008-0.0138 | 8.00E-14 |
| SmkInit | rs10233018 | Smoking initiation (ever regular vs never regular) (MTAG) | 30643251 | chr7:117523709 | A=0.487, G=0.513 | 0.516 | 0.0122421 | 0.01-0.014 | 8.00E-30 |
| SmkInit | rs10233018 | Smoking initiation (ever regular vs never regular) | 30643251 | chr7:117523709 | A=0.487, G=0.513 | 0.516 | 0.024612404 | 0.02-0.03 | 5.00E-22 |
| SmkInit | rs10233018 | Smoking initiation | 36477530 | chr7:117523709 | A=0.487, G=0.513 | 0.519 | 0.012 | 0.01-0.014 | 1.00E-43 |
| SmkInit | rs10233018 | Smoking initiation | 36477530 | chr7:117523709 | A=0.487, G=0.513 | 0.511 | 0.0113 | 0.0098-0.0128 | 3.00E-48 |
| SmkInit | rs1899689 | Body mass index | 30239722 | chr7:121964349 | C=0.62, T=0.38 | 0.3939 | 0.012 | 0.0087-0.0153 | 4.00E-13 |
| SmkInit | rs1899689 | Body mass index | 31669095 | chr7:121964349 | C=0.62, T=0.38 | NR | NA | NA | 9.00E-10 |
| SmkInit | rs1899689 | Adult body size | 32376654 | chr7:121964349 | C=0.62, T=0.38 | 0.610356 | 0.00773624 | 0.005-0.0105 | 4.00E-08 |
| SmkInit | rs1899689 | Lifetime smoking | 34855049 | chr7:121964349 | C=0.62, T=0.38 | NR | NA | NA | 1.00E-08 |
| SmkInit | rs1899689 | Height | 36224396 | chr7:121964349 | C=0.62, T=0.38 | 0.4129 | 0.0166 | 0.012-0.021 | 3.00E-14 |
| SmkInit | rs1899689 | Body mass index | 37280435 | chr7:121964349 | C=0.62, T=0.38 | NR | 0.0126282 | 0.0088-0.0165 | 1.00E-10 |
| SmkInit | rs1899689 | Height | 36224396 | chr7:121964349 | C=0.62, T=0.38 | 0.3985 | 0.0163 | 0.015-0.017 | 3.00E-146 |
| SmkInit | rs10279261 | Smoking initiation (ever regular vs never regular) (MTAG) | 30643251 | chr7:133589846 | A=0.63, G=0.37 | 0.618 | 0.00958076 | 0.0074-0.0118 | 7.00E-18 |
| SmkInit | rs10279261 | Smoking initiation (ever regular vs never regular) | 30643251 | chr7:133589846 | A=0.63, G=0.37 | 0.618 | 0.018872635 | 0.014-0.024 | 6.00E-13 |
| SmkInit | rs10279261 | Smoking status (ever vs never smokers) | 30643258 | chr7:133589846 | A=0.63, G=0.37 | 0.6167 | 0.01130086 | 0.0073-0.0154 | 5.00E-08 |
| SmkInit | rs10279261 | Smoking initiation | 36477530 | chr7:133589846 | A=0.63, G=0.37 | 0.616 | 0.00964 | 0.0079-0.0114 | 3.00E-27 |
| SmkInit | rs10279261 | Smoking initiation | 36477530 | chr7:133589846 | A=0.63, G=0.37 | 0.608 | 0.00876 | 0.0072-0.0103 | 3.00E-28 |
| SmkInit | rs6464024 | Smoking status | 30595370 | chr7:1688369 | C=0.563, T=0.437 | NR | NA | NA | 2.00E-08 |
| AgeSmk | rs2491383 | Age of smoking initiation (MTAG) | 30643251 | chr10:106616640 | G=0.62, T=0.38 | 0.599 | 0.00956608 | 0.0067-0.0125 | 1.00E-10 |
| AgeSmk | rs12575642 | Attention deficit hyperactivity disorder | 21784300 | chr11:63979643 | G=0.816, T=0.184 | NR | NA | NA | 1.00E-06 |
| AgeSmk | rs11080208 | Smoking initiation | 36477530 | chr17:31550443 | C=0.752, T=0.248 | 0.76 | 0.00686 | 0.0049-0.0088 | 1.00E-11 |
| AgeSmk | rs571312 | Body mass index in physically inactive individuals | 28448500 | chr18:57839769 | A=0.24, C=0.76 | 0.2878 | 0.0657 | 0.042-0.089 | 6.00E-08 |
| AgeSmk | rs571312 | Body mass index in physically inactive individuals | 28448500 | chr18:57839769 | A=0.24, C=0.76 | 0.2689 | 0.0603 | 0.043-0.077 | 4.00E-12 |
| AgeSmk | rs571312 | Body mass index in physically inactive individuals | 28448500 | chr18:57839769 | A=0.24, C=0.76 | 0.2347 | 0.063 | 0.039-0.087 | 3.00E-07 |
| AgeSmk | rs571312 | Body mass index | 20935630 | chr18:57839769 | A=0.24, C=0.76 | 0.24 | 0.23 | 0.17-0.29/2 | 6.00E-42 |
| AgeSmk | rs571312 | C-reactive protein levels or triglyceride levels (pleiotropy) | 27286809 | chr18:57839769 | A=0.24, C=0.76 | NR | NA | NA | 3.00E-08 |
| AgeSmk | rs571312 | Body mass index (joint analysis main effects and physical activity interaction) | 28448500 | chr18:57839769 | A=0.24, C=0.76 | NR | NA | NA | 5.00E-24 |
| AgeSmk | rs571312 | Body mass index | 28448500 | chr18:57839769 | A=0.24, C=0.76 | 0.2455 | 0.0646 | 0.052-0.077/2 | 1.00E-25 |
| AgeSmk | rs571312 | Body mass index | 28448500 | chr18:57839769 | A=0.24, C=0.76 | 0.2365 | 0.065 | 0.052-0.078/2 | 7.00E-22 |
| AgeSmk | rs571312 | Body mass index (age interaction) | 25953783 | chr18:57839769 | A=0.24, C=0.76 | 0.234 | NA | NA | 5.00E-09 |
| AgeSmk | rs571312 | Body mass index | 25953783 | chr18:57839769 | A=0.24, C=0.76 | 0.234 | 0.14 | 0.14-0.14 | 5.00E-07 |
| AgeSmk | rs571312 | Body mass index | 25953783 | chr18:57839769 | A=0.24, C=0.76 | 0.234 | NA | NA | 4.00E-09 |
| AgeSmk | rs571312 | Childhood body mass index | 33045005 | chr18:57839769 | A=0.24, C=0.76 | 0.23 | 0.059 | 0.041-0.077 | 2.00E-10 |
| AgeSmk | rs571312 | Body mass index | 36581621 | chr18:57839769 | A=0.24, C=0.76 | NR | 0.0486 | 0.045-0.052 | 2.00E-213 |
| AgeSmk | rs571312 | Height | 36224396 | chr18:57839769 | A=0.24, C=0.76 | 0.2053 | 0.0303 | 0.024-0.036 | 3.00E-24 |
| AgeSmk | rs62180314 | Age of smoking initiation | 36477530 | chr2:63373133 | C=0.208, G=0.792 | 0.208 | 0.0174 | 0.013-0.022 | 5.00E-15 |
| AgeSmk | rs62180314 | Age of smoking initiation | 36477530 | chr2:63373133 | C=0.208, G=0.792 | 0.19 | 0.0165 | 0.012-0.021 | 1.00E-14 |
| AgeSmk | rs11915747 | Educational attainment (years of education) | 30595370 | chr3:85699040 | C=0.632, G=0.368 | NR | NA | NA | 4.00E-17 |
| AgeSmk | rs11915747 | Adult body size | 32376654 | chr3:85699040 | C=0.632, G=0.368 | 0.647257 | 0.010795 | 0.008-0.0136 | 5.00E-14 |
| AgeSmk | rs11915747 | Age of smoking initiation (MTAG) | 30643251 | chr3:85699040 | C=0.632, G=0.368 | 0.354 | 0.0147128 | 0.012-0.018 | 6.00E-22 |
| AgeSmk | rs11915747 | Age of smoking initiation | 30643251 | chr3:85699040 | C=0.632, G=0.368 | 0.354 | 0.02 | 0.015-0.025 | 2.00E-15 |
| AgeSmk | rs11915747 | Self-reported math ability (MTAG) | 30038396 | chr3:85699040 | C=0.632, G=0.368 | 0.6481 | 0.0144 | 0.011-0.018 | 2.00E-14 |
| AgeSmk | rs11915747 | Body surface area | 36502284 | chr3:85699040 | C=0.632, G=0.368 | NR | 0.00304395 | 0.0022-0.0039 | 5.00E-13 |
| AgeSmk | rs11915747 | Body mass index | 38538606 | chr3:85699040 | C=0.632, G=0.368 | NR | 0.0208712 | 0.016-0.026 | 1.00E-16 |
| AgeSmk | rs11915747 | Whole body fat mass (UKB data field 23100) | 38538606 | chr3:85699040 | C=0.632, G=0.368 | NR | 0.0182985 | 0.013-0.023 | 1.00E-13 |
| AgeSmk | rs624833 | Age of smoking initiation (MTAG) | 30643251 | chr4:2881256 | G=0.328, T=0.672 | 0.302 | 0.0114003 | 0.0083-0.0145 | 7.00E-13 |
| AgeSmk | rs624833 | Age of smoking initiation | 30643251 | chr4:2881256 | G=0.328, T=0.672 | 0.302 | 0.016 | 0.011-0.021 | 2.00E-09 |
| AgeSmk | rs624833 | Lifetime smoking index | 31689377 | chr4:2881256 | G=0.328, T=0.672 | 0.695 | 0.013 | 0.0091-0.0169 | 7.00E-10 |
| AgeSmk | rs2607015 | Waist-hip index | 34021172 | chr6:31762843 | C=0.441, G=0.559 | NR | 0.0188809 | 0.013-0.024 | 4.00E-11 |
| AgeSmk | rs2607015 | Waist-to-hip ratio adjusted for BMI | 34021172 | chr6:31762843 | C=0.441, G=0.559 | NR | 0.0196764 | 0.014-0.025 | 5.00E-12 |
| AgeSmk | rs2607015 | Waist-to-hip ratio adjusted for BMI | 34021172 | chr6:31762843 | C=0.441, G=0.559 | NR | 0.0175576 | 0.011-0.024 | 3.00E-08 |
| AgeSmk | rs11780471 | Lung cancer | 28604730 | chr8:27344719 | A=0.076, G=0.924 | 0.940381112 | 1.1515831 | 1.1-1.21 | 2.00E-08 |
| AgeSmk | rs11780471 | Age of smoking initiation | 30643251 | chr8:27344719 | A=0.076, G=0.924 | 0.0601 | 0.033 | 0.023-0.043 | 9.00E-11 |
| AgeSmk | rs11780471 | Smoking initiation (ever regular vs never regular) | 30643251 | chr8:27344719 | A=0.076, G=0.924 | 0.063121 | 0.038677923 | 0.028-0.049 | 2.00E-13 |
| AgeSmk | rs11780471 | Age of smoking initiation | 36477530 | chr8:27344719 | A=0.076, G=0.924 | 0.0611 | 0.0295 | 0.022-0.037 | 4.00E-15 |
| AgeSmk | rs11780471 | Smoking initiation | 36477530 | chr8:27344719 | A=0.076, G=0.924 | 0.0606 | 0.0377 | 0.034-0.041 | 5.00E-110 |
| AgeSmk | rs11780471 | Smoking status (standard GWA) | 37106081 | chr8:27344719 | A=0.076, G=0.924 | NR | 0.020043 | 0.013-0.027 | 4.00E-08 |
| CigDay | rs7928017 | Educational attainment (years of education) | 30038396 | chr11:113448762 | A=0.409, C=0.591 | 0.4341 | 0.0096 | 0.0069-0.0123 | 1.00E-11 |
| CigDay | rs7928017 | Cigarettes smoked per day | 36477530 | chr11:113448762 | A=0.409, C=0.591 | 0.43 | 0.0158 | 0.012-0.019 | 3.00E-18 |
| CigDay | rs7928017 | Cigarettes smoked per day (MTAG) | 36376304 | chr11:113448762 | A=0.409, C=0.591 | NR | 0.01562 | 0.011-0.021 | 4.00E-10 |
| CigDay | rs7928017 | Cigarettes smoked per day | 36477530 | chr11:113448762 | A=0.409, C=0.591 | 0.404 | 0.014 | 0.011-0.017 | 2.00E-17 |
| CigDay | rs7933830 | Cigarettes smoked per day | 36477530 | chr11:16377119 | C=0.681, T=0.319 | 0.31 | 0.0157 | 0.012-0.019 | 5.00E-17 |
| CigDay | rs17197116 | Cigarettes smoked per day (MTAG) | 30643251 | chr11:46520302 | C=0.084, T=0.916 | 0.0835 | 0.0266115 | 0.019-0.034 | 2.00E-11 |
| CigDay | rs8021229 | Cigarettes smoked per day (MTAG) | 36376304 | chr14:104157578 | C=0.704, T=0.296 | NR | 0.016916 | 0.012-0.022 | 2.00E-10 |
| CigDay | rs1657936 | Cigarettes smoked per day | 36477530 | chr15:57113013 | C=0.219, T=0.781 | 0.792 | 0.0196 | 0.015-0.024 | 7.00E-19 |
| CigDay | rs72738704 | Post bronchodilator FEV1/FVC ratio in COPD | 26634245 | chr15:78719832 | C=0.37, G=0.63 | 0.39 | 0.012 | NA | 4.00E-06 |
| CigDay | rs72738704 | Post bronchodilator FEV1 | 26634245 | chr15:78719832 | C=0.37, G=0.63 | 0.34 | 0.075 | NA | 2.00E-13 |
| CigDay | rs72738704 | Red blood cell count | 30595370 | chr15:78719832 | C=0.37, G=0.63 | NR | NA | NA | 1.00E-07 |
| CigDay | rs72738704 | Post bronchodilator FEV1/FVC ratio | 26634245 | chr15:78719832 | C=0.37, G=0.63 | 0.336 | 0.015 | NA | 2.00E-12 |
| CigDay | rs669696 | Smoking initiation | 36477530 | chr16:69626136 | A=0.423, C=0.577 | 0.382 | 0.0107 | 0.0091-0.0123 | 7.00E-39 |
| CigDay | rs112178027 | Brainstem volume | 31636452 | chr17:27564013 | C=0.806, T=0.194 | 0.1745 | 6.157 | NA | 7.00E-10 |
| CigDay | rs112178027 | Platelet count | 34469753 | chr17:27564013 | C=0.806, T=0.194 | 0.832 | NA | NA | 2.00E-15 |
| CigDay | rs11663346 | Cigarettes smoked per day (MTAG) | 30643251 | chr18:62152458 | A=0.408, T=0.592 | 0.582 | 0.0139519 | 0.0096-0.0183 | 3.00E-10 |
| CigDay | rs34406232 | Height | 36224396 | chr19:41305530 | A=0.026, C=0.974 | 0.0243 | 0.0227 | 0.019-0.027 | 8.00E-28 |
| CigDay | rs56113850 | Smoking behaviour (cigarettes smoked per day) | 30679032 | chr19:41353107 | C=0.592, T=0.408 | 0.575 | 0.0719 | 0.064-0.08 | 3.00E-68 |
| CigDay | rs56113850 | Smoking behaviour (cigarette pack-years) | 30679032 | chr19:41353107 | C=0.592, T=0.408 | 0.572 | 0.0385 | 0.031-0.046 | 6.00E-25 |
| CigDay | rs56113850 | Caffeine consumption from coffee | 33287642 | chr19:41353107 | C=0.592, T=0.408 | 0.421821 | 0.02071 | 0.016-0.025 | 5.00E-19 |
| CigDay | rs56113850 | Caffeine consumption from coffee or tea | 33287642 | chr19:41353107 | C=0.592, T=0.408 | 0.421755 | 0.02649 | 0.022-0.031 | 2.00E-29 |
| CigDay | rs56113850 | Post bronchodilator FEV1 | 26634245 | chr19:41353107 | C=0.592, T=0.408 | 0.458 | 0.067 | NA | 2.00E-08 |
| CigDay | rs56113850 | Nicotine metabolite ratio in current smokers | 27488534 | chr19:41353107 | C=0.592, T=0.408 | NR | 0.3644 | 0.32-0.41 | 1.00E-50 |
| CigDay | rs56113850 | Post bronchodilator FEV1/FVC ratio | 26634245 | chr19:41353107 | C=0.592, T=0.408 | 0.462 | 0.014 | NA | 5.00E-09 |
| CigDay | rs56113850 | Caffeine metabolism (plasma 1,7-dimethylxanthine (paraxanthine) to 1,3,7-trimethylxanthine (caffeine) ratio) | 27702941 | chr19:41353107 | C=0.592, T=0.408 | 0.43 | 9.59 | - | 9.00E-22 |
| CigDay | rs56113850 | Caffeine metabolism (plasma 1,7-dimethylxanthine (paraxanthine) level) | 27702941 | chr19:41353107 | C=0.592, T=0.408 | 0.43 | 5.43 | NA | 6.00E-08 |
| CigDay | rs56113850 | Caffeine metabolism (plasma 3,7-dimethylxanthine (theobromine) level) | 27702941 | chr19:41353107 | C=0.592, T=0.408 | 0.43 | 4.59 | - | 4.00E-06 |
| CigDay | rs56113850 | Nicotine metabolite ratio in current smokers | 26407342 | chr19:41353107 | C=0.592, T=0.408 | NR | 0.653 | 0.59-0.72 | 6.00E-86 |
| CigDay | rs56113850 | LDL cholesterol levels | 32203549 | chr19:41353107 | C=0.592, T=0.408 | 0.422594 | 0.013364 | 0.0093-0.0175 | 2.00E-10 |
| CigDay | rs56113850 | Smoking cessation | 33082346 | chr19:41353107 | C=0.592, T=0.408 | NR | 0.946 | NA | 7.00E-33 |
| CigDay | rs56113850 | Apolipoprotein B levels | 32203549 | chr19:41353107 | C=0.592, T=0.408 | 0.42254 | 0.0135934 | 0.0095-0.0177 | 6.00E-11 |
| CigDay | rs56113850 | Local histogram emphysema pattern | 25006744 | chr19:41353107 | C=0.592, T=0.408 | 0.4 | 0.02 | 0.012-0.028 | 1.00E-09 |
| CigDay | rs56113850 | Local histogram emphysema pattern | 25006744 | chr19:41353107 | C=0.592, T=0.408 | 0.4 | 0.02 | 0.012-0.028 | 1.00E-06 |
| CigDay | rs56113850 | Lung cancer | 28604730 | chr19:41353107 | C=0.592, T=0.408 | 0.559578448 | 1.1306498 | 1.1-1.16 | 5.00E-19 |
| CigDay | rs56113850 | Lung adenocarcinoma | 28604730 | chr19:41353107 | C=0.592, T=0.408 | 0.555412482 | 1.121034 | 1.08-1.16 | 9.00E-10 |
| CigDay | rs56113850 | Squamous cell lung carcinoma | 28604730 | chr19:41353107 | C=0.592, T=0.408 | 0.55529151 | 1.1555213 | 1.11-1.21 | 7.00E-11 |
| CigDay | rs56113850 | Lung cancer in ever smokers | 28604730 | chr19:41353107 | C=0.592, T=0.408 | 0.564992436 | 1.1372905 | 1.1-1.18 | 4.00E-13 |
| CigDay | rs56113850 | Lung adenocarcinoma | 32889700 | chr19:41353107 | C=0.592, T=0.408 | NR | 0.93 | 0.89-0.96 | 8.00E-06 |
| CigDay | rs56113850 | Non-small cell lung cancer | 32889700 | chr19:41353107 | C=0.592, T=0.408 | NR | 0.92 | 0.89-0.94 | 3.00E-09 |
| CigDay | rs56113850 | Nicotine metabolite ratio in current smokers | 32157176 | chr19:41353107 | C=0.592, T=0.408 | 0.447 | 0.682 | 0.64-0.72 | 6.00E-261 |
| CigDay | rs56113850 | Smoking intensity (cotinine levels/cigarettes per day) | 32157176 | chr19:41353107 | C=0.592, T=0.408 | 0.447 | 0.155 | 0.11-0.2 | 3.00E-13 |
| CigDay | rs56113850 | Plasma letrozole concentrations in letrozole treated hormone-receptor-positive breast cancer | 34096894 | chr19:41353107 | C=0.592, T=0.408 | 0.46 | 4693.72 | 3102.87-6284.57 | 2.00E-13 |
| CigDay | rs56113850 | Nicotine dependence | 33144568 | chr19:41353107 | C=0.592, T=0.408 | NR | 0.031 | 0.023-0.039 | 4.00E-16 |
| CigDay | rs56113850 | Aerodigestive squamous cell cancer (pleiotropy) | 33667223 | chr19:41353107 | C=0.592, T=0.408 | 0.41 | 0.9 | 0.86-0.94 | 7.00E-11 |
| CigDay | rs56113850 | Linoleic acid levels | 35213538 | chr19:41353107 | C=0.592, T=0.408 | 0.420788 | 0.0239432 | 0.016-0.032 | 6.00E-09 |
| CigDay | rs56113850 | Glycine levels | 35213538 | chr19:41353107 | C=0.592, T=0.408 | 0.420828 | 0.0218076 | 0.014-0.03 | 3.00E-08 |
| CigDay | rs56113850 | Apolipoprotein B levels | 35213538 | chr19:41353107 | C=0.592, T=0.408 | 0.420796 | 0.0239612 | 0.016-0.032 | 9.00E-09 |
| CigDay | rs56113850 | Cholesterol levels in small VLDL | 35213538 | chr19:41353107 | C=0.592, T=0.408 | 0.420796 | 0.0240384 | 0.016-0.032 | 8.00E-09 |
| CigDay | rs56113850 | Free cholesterol levels in small VLDL | 35213538 | chr19:41353107 | C=0.592, T=0.408 | 0.420796 | 0.0235212 | 0.015-0.032 | 2.00E-08 |
| CigDay | rs56113850 | Free cholesterol levels in very small VLDL | 35213538 | chr19:41353107 | C=0.592, T=0.408 | 0.420796 | 0.0231331 | 0.015-0.031 | 2.00E-08 |
| CigDay | rs56113850 | Phospholipid levels in small LDL | 35213538 | chr19:41353107 | C=0.592, T=0.408 | 0.420796 | 0.0243826 | 0.016-0.033 | 4.00E-09 |
| CigDay | rs56113850 | Concentration of small LDL particles | 35213538 | chr19:41353107 | C=0.592, T=0.408 | 0.420796 | 0.0241421 | 0.016-0.032 | 8.00E-09 |
| CigDay | rs56113850 | Concentration of very small VLDL particles | 35213538 | chr19:41353107 | C=0.592, T=0.408 | 0.420796 | 0.0238214 | 0.016-0.032 | 7.00E-09 |
| CigDay | rs56113850 | Total lipid levels in very small VLDL | 35213538 | chr19:41353107 | C=0.592, T=0.408 | 0.420796 | 0.0224965 | 0.014-0.031 | 4.00E-08 |
| CigDay | rs56113850 | Phospholipid levels in small VLDL | 35213538 | chr19:41353107 | C=0.592, T=0.408 | 0.420796 | 0.0231974 | 0.015-0.031 | 3.00E-08 |
| CigDay | rs56113850 | Cholesteryl ester levels in small VLDL | 35213538 | chr19:41353107 | C=0.592, T=0.408 | 0.420796 | 0.0240607 | 0.016-0.032 | 8.00E-09 |
| CigDay | rs56113850 | Total lipid levels in small LDL | 35213538 | chr19:41353107 | C=0.592, T=0.408 | 0.420796 | 0.0237331 | 0.016-0.032 | 1.00E-08 |
| CigDay | rs56113850 | Phospholipid levels in very small VLDL | 35213538 | chr19:41353107 | C=0.592, T=0.408 | 0.420796 | 0.0234529 | 0.015-0.031 | 1.00E-08 |
| CigDay | rs56113850 | Serum alkaline phosphatase levels | 34594039 | chr19:41353107 | C=0.592, T=0.408 | NR | 0.0472 | 0.042-0.052 | 1.00E-89 |
| CigDay | rs56113850 | Estimated glomerular filtration rate (creatinine) | 35710981 | chr19:41353107 | C=0.592, T=0.408 | 0.5599 | 7.151 | NA | 9.00E-13 |
| CigDay | rs56113850 | Chronotype | 30696823 | chr19:41353107 | C=0.592, T=0.408 | 0.5769 | 1.0231643 | NA | 2.00E-10 |
| CigDay | rs56113850 | Bitter non-alcoholic beverage consumption | 31046077 | chr19:41353107 | C=0.592, T=0.408 | 0.5766 | 0.0107 | 0.0076-0.0138 | 7.00E-11 |
| CigDay | rs56113850 | Coffee consumption | 31046077 | chr19:41353107 | C=0.592, T=0.408 | 0.5776 | 0.0121 | 0.0092-0.015 | 8.00E-16 |
| CigDay | rs56113850 | Bitter beverage consumption | 31046077 | chr19:41353107 | C=0.592, T=0.408 | 0.5766 | 0.0107 | 0.0076-0.0138 | 8.00E-11 |
| CigDay | rs56113850 | Cigarettes smoked per day (MTAG) | 30643251 | chr19:41353107 | C=0.592, T=0.408 | 0.555 | 0.0361686 | 0.032-0.041 | 5.00E-58 |
| CigDay | rs56113850 | Smoking cessation (MTAG) | 30643251 | chr19:41353107 | C=0.592, T=0.408 | 0.567 | 0.00974331 | 0.0067-0.0128 | 6.00E-10 |
| CigDay | rs56113850 | Smoking behaviour (cigarettes smoked per day) | 30643251 | chr19:41353107 | C=0.592, T=0.408 | 0.555 | 0.0523096 | 0.047-0.057 | 4.00E-99 |
| CigDay | rs56113850 | Smoking cessation | 30643251 | chr19:41353107 | C=0.592, T=0.408 | 0.567 | 0.05760633 | 0.05-0.065 | 2.00E-48 |
| CigDay | rs56113850 | Ratio of apolipoprotein B to apolipoprotein A1 levels | 35213538 | chr19:41353107 | C=0.592, T=0.408 | 0.420796 | 0.0227187 | 0.015-0.031 | 3.00E-08 |
| CigDay | rs56113850 | Concentration of LDL particles | 35213538 | chr19:41353107 | C=0.592, T=0.408 | 0.420796 | 0.0237631 | 0.016-0.032 | 1.00E-08 |
| CigDay | rs56113850 | Triglyceride levels in large LDL | 35213538 | chr19:41353107 | C=0.592, T=0.408 | 0.420796 | 0.0230106 | 0.015-0.031 | 2.00E-08 |
| CigDay | rs56113850 | Concentration of large LDL particles | 35213538 | chr19:41353107 | C=0.592, T=0.408 | 0.420796 | 0.0237825 | 0.016-0.032 | 1.00E-08 |
| CigDay | rs56113850 | Non-small cell lung cancer | 31326317 | chr19:41353107 | C=0.592, T=0.408 | 0.487 | 1.09 | 1.06-1.12 | 3.00E-09 |
| CigDay | rs56113850 | Lung adenocarcinoma | 35915169 | chr19:41353107 | C=0.592, T=0.408 | NR | 0.07 | 0.031-0.109 | 8.00E-11 |
| CigDay | rs56113850 | Squamous cell lung carcinoma | 35915169 | chr19:41353107 | C=0.592, T=0.408 | NR | 0.09 | 0.031-0.149 | 3.00E-11 |
| CigDay | rs56113850 | Lung cancer | 35915169 | chr19:41353107 | C=0.592, T=0.408 | NR | 0.08 | 0.041-0.119 | 8.00E-27 |
| CigDay | rs56113850 | Urine X-21831 levels in chronic kidney disease | 37277652 | chr19:41353107 | C=0.592, T=0.408 | 0.59 | 0.469 | 0.39-0.55 | 8.00E-31 |
| CigDay | rs56113850 | Urine X-18935 levels in chronic kidney disease | 37277652 | chr19:41353107 | C=0.592, T=0.408 | 0.58 | 0.294 | 0.24-0.34 | 1.00E-29 |
| CigDay | rs56113850 | Alanine aminotransferase levels | 33462484 | chr19:41353107 | C=0.592, T=0.408 | NR | 0.0167 | 0.012-0.021 | 3.00E-12 |
| CigDay | rs56113850 | Nicotine metabolite ratio in current smokers | 34599228 | chr19:41353107 | C=0.592, T=0.408 | NR | 0.671 | 0.54-0.8 | 7.00E-22 |
| CigDay | rs56113850 | Nicotine metabolite ratio in current smokers | 34599228 | chr19:41353107 | C=0.592, T=0.408 | NR | 0.748 | 0.64-0.85 | 1.00E-37 |
| CigDay | rs56113850 | Liver enzyme levels (alanine transaminase) | 33972514 | chr19:41353107 | C=0.592, T=0.408 | 0.422385 | 0.00312488 | 0.0024-0.0039 | 1.00E-15 |
| CigDay | rs56113850 | Urine X-12126 levels in chronic kidney disease | 37277652 | chr19:41353107 | C=0.592, T=0.408 | 0.58 | 0.189 | 0.14-0.24 | 3.00E-14 |
| CigDay | rs56113850 | Plasma X-12216 levels in chronic kidney disease | 37277652 | chr19:41353107 | C=0.592, T=0.408 | 0.59 | 0.173 | 0.12-0.22 | 8.00E-12 |
| CigDay | rs56113850 | F-chocolate/coffee liking (derived food-liking factor) | 35585065 | chr19:41353107 | C=0.592, T=0.408 | 0.5774 | 0.090892 | 0.062-0.12 | 1.00E-09 |
| CigDay | rs56113850 | Low density lipoprotein cholesterol levels | 34887591 | chr19:41353107 | C=0.592, T=0.408 | NR | NA | NA | 3.00E-17 |
| CigDay | rs56113850 | Low density lipoprotein cholesterol levels | 34887591 | chr19:41353107 | C=0.592, T=0.408 | 0.567435 | 0.0142152 | 0.011-0.017 | 2.00E-16 |
| CigDay | rs56113850 | Coffee consumption (cups per day) | 32193382 | chr19:41353107 | C=0.592, T=0.408 | 0.422202 | 0.0186901 | 0.015-0.023 | 8.00E-19 |
| CigDay | rs56113850 | X-12216 levels | 35347128 | chr19:41353107 | C=0.592, T=0.408 | NR | 0.15 | NA | 3.00E-15 |
| CigDay | rs56113850 | Theobromine levels | 35347128 | chr19:41353107 | C=0.592, T=0.408 | NR | 0.13 | NA | 3.00E-12 |
| CigDay | rs56113850 | Paraxanthine levels | 35347128 | chr19:41353107 | C=0.592, T=0.408 | NR | 0.17 | NA | 2.00E-19 |
| CigDay | rs56113850 | 3-methylxanthine levels | 35347128 | chr19:41353107 | C=0.592, T=0.408 | NR | 0.16 | NA | 9.00E-18 |
| CigDay | rs56113850 | 6-hydroxyindole sulfate levels | 35347128 | chr19:41353107 | C=0.592, T=0.408 | NR | 0.12 | NA | 2.00E-11 |
| CigDay | rs56113850 | Indolin-2-one levels | 35347128 | chr19:41353107 | C=0.592, T=0.408 | NR | 0.17 | NA | 7.00E-15 |
| CigDay | rs56113850 | 3-methylxanthine levels | 35050183 | chr19:41353107 | C=0.592, T=0.408 | NR | 0.155 | 0.13-0.18 | 2.00E-24 |
| CigDay | rs56113850 | X-21286 levels | 35050183 | chr19:41353107 | C=0.592, T=0.408 | NR | 0.104 | 0.075-0.133 | 8.00E-12 |
| CigDay | rs56113850 | X-12216 levels | 35050183 | chr19:41353107 | C=0.592, T=0.408 | NR | 0.139 | 0.11-0.17 | 4.00E-20 |
| CigDay | rs56113850 | 6-hydroxyindole sulfate levels | 36357675 | chr19:41353107 | C=0.592, T=0.408 | 0.4287 | 0.09685272 | 0.076-0.117 | 2.00E-20 |
| CigDay | rs56113850 | X-21286 levels | 36357675 | chr19:41353107 | C=0.592, T=0.408 | 0.4289 | 0.13240077 | 0.11-0.15 | 7.00E-38 |
| CigDay | rs56113850 | Theobromine levels | 36357675 | chr19:41353107 | C=0.592, T=0.408 | 0.4286 | 0.09372097 | 0.074-0.114 | 8.00E-20 |
| CigDay | rs56113850 | X-12216 levels | 36357675 | chr19:41353107 | C=0.592, T=0.408 | 0.4278 | 0.1274714 | 0.11-0.15 | 7.00E-35 |
| CigDay | rs56113850 | X-12126 levels | 36357675 | chr19:41353107 | C=0.592, T=0.408 | 0.4165 | 0.1277143 | 0.1-0.15 | 6.00E-26 |
| CigDay | rs56113850 | Indolin-2-one levels | 36357675 | chr19:41353107 | C=0.592, T=0.408 | 0.4373 | 0.1445022 | 0.12-0.17 | 1.00E-30 |
| CigDay | rs56113850 | 3-methylxanthine levels | 36357675 | chr19:41353107 | C=0.592, T=0.408 | 0.4288 | 0.14389715 | 0.12-0.16 | 3.00E-44 |
| CigDay | rs56113850 | 3-hydroxycotinine glucuronide levels | 36357675 | chr19:41353107 | C=0.592, T=0.408 | 0.4184 | 0.26954755 | 0.2-0.34 | 3.00E-15 |
| CigDay | rs56113850 | Paraxanthine levels | 36357675 | chr19:41353107 | C=0.592, T=0.408 | 0.4292 | 0.11057526 | 0.09-0.131 | 1.00E-26 |
| CigDay | rs56113850 | 1,7-dimethylurate levels | 36357675 | chr19:41353107 | C=0.592, T=0.408 | 0.4287 | 0.10510567 | 0.085-0.125 | 3.00E-24 |
| CigDay | rs56113850 | Smoking cessation | 36477530 | chr19:41353107 | C=0.592, T=0.408 | 0.572 | 0.0303 | 0.028-0.033 | 5.00E-114 |
| CigDay | rs56113850 | Non-HDL cholesterol levels | 34887591 | chr19:41353107 | C=0.592, T=0.408 | 0.562613 | 0.014895 | 0.012-0.018 | 2.00E-13 |
| CigDay | rs56113850 | Cigarettes smoked per day | 36477530 | chr19:41353107 | C=0.592, T=0.408 | 0.565 | 0.0557 | 0.052-0.059 | 6.00E-207 |
| CigDay | rs56113850 | C-reactive protein levels (MTAG) | 36376304 | chr19:41353107 | C=0.592, T=0.408 | NR | 0.01645 | 0.012-0.02 | 8.00E-16 |
| CigDay | rs56113850 | Non-HDL cholesterol levels | 34887591 | chr19:41353107 | C=0.592, T=0.408 | NR | NA | NA | 2.00E-14 |
| CigDay | rs56113850 | Smoking cessation | 36477530 | chr19:41353107 | C=0.592, T=0.408 | 0.563 | 0.0303 | 0.028-0.033 | 1.00E-120 |
| CigDay | rs56113850 | Indolin-2-one levels | 36635386 | chr19:41353107 | C=0.592, T=0.408 | 0.570252 | 0.167321 | 0.14-0.2 | 2.00E-24 |
| CigDay | rs56113850 | X-18935 levels | 36635386 | chr19:41353107 | C=0.592, T=0.408 | 0.606272 | 0.131701 | 0.092-0.171 | 8.00E-11 |
| CigDay | rs56113850 | X-21286 levels | 36635386 | chr19:41353107 | C=0.592, T=0.408 | 0.576068 | 0.136846 | 0.11-0.17 | 2.00E-19 |
| CigDay | rs56113850 | X-12216 levels | 36635386 | chr19:41353107 | C=0.592, T=0.408 | 0.577382 | 0.129952 | 0.1-0.16 | 7.00E-18 |
| CigDay | rs56113850 | Alanine aminotransferase levels | 38632349 | chr19:41353107 | C=0.592, T=0.408 | NR | 0.0034 | 0.0026-0.0042 | 6.00E-18 |
| CigDay | rs56113850 | Cigarettes smoked per day (MTAG) | 36376304 | chr19:41353107 | C=0.592, T=0.408 | NR | 0.04714 | 0.042-0.052 | 2.00E-78 |
| CigDay | rs56113850 | Glycine levels (UKB data field 23462) | 36764567 | chr19:41353107 | C=0.592, T=0.408 | 0.420048 | 0.0279433 | 0.019-0.037 | 7.00E-10 |
| CigDay | rs56113850 | Cigarettes smoked per day | 36477530 | chr19:41353107 | C=0.592, T=0.408 | 0.55 | 0.0534 | 0.05-0.057 | 3.00E-214 |
| CigDay | rs56113850 | 6-hydroxyindole sulfate levels | 36635386 | chr19:41353107 | C=0.592, T=0.408 | 0.57613 | 0.109573 | 0.08-0.139 | 6.00E-13 |
| CigDay | rs56113850 | Paraxanthine levels | 36635386 | chr19:41353107 | C=0.592, T=0.408 | 0.573947 | 0.111197 | 0.08-0.142 | 3.00E-12 |
| CigDay | rs56113850 | Theobromine levels | 36635386 | chr19:41353107 | C=0.592, T=0.408 | 0.576193 | 0.105914 | 0.075-0.136 | 1.00E-11 |
| CigDay | rs56113850 | Metabolite levels (hydroxycotinine) | 37253714 | chr19:41353107 | C=0.592, T=0.408 | 0.5489 | 0.2 | 0.14-0.26 | 2.00E-12 |
| CigDay | rs2072659 | Smoking behaviour (cigarettes smoked per day) | 30679032 | chr1:154548521 | C=0.899, G=0.101 | 0.102 | 0.0369 | 0.024-0.05 | 4.00E-08 |
| CigDay | rs2072659 | Cigarettes smoked per day (MTAG) | 30643251 | chr1:154548521 | C=0.899, G=0.101 | 0.0986 | 0.0314007 | 0.024-0.039 | 7.00E-16 |
| CigDay | rs2072659 | Smoking behaviour (cigarettes smoked per day) | 30643251 | chr1:154548521 | C=0.899, G=0.101 | 0.0986 | 0.029984204 | 0.022-0.038 | 3.00E-13 |
| CigDay | rs2072659 | Cigarettes smoked per day | 36477530 | chr1:154548521 | C=0.899, G=0.101 | 0.0984 | 0.0261 | 0.02-0.032 | 6.00E-18 |
| CigDay | rs2072659 | Cigarettes smoked per day | 36477530 | chr1:154548521 | C=0.899, G=0.101 | 0.106 | 0.0231 | 0.018-0.028 | 1.00E-18 |
| CigDay | rs6699355 | Cigarettes smoked per day | 36477530 | chr1:35384605 | C=0.122, T=0.878 | 0.875 | 0.0231 | 0.018-0.028 | 3.00E-17 |
| CigDay | rs6699355 | Cigarettes smoked per day | 36477530 | chr1:35384605 | C=0.122, T=0.878 | 0.787 | 0.019 | 0.015-0.023 | 7.00E-21 |
| CigDay | rs2133203 | Cigarettes smoked per day | 36477530 | chr1:77974530 | C=0.55, T=0.45 | 0.416 | 0.0138 | 0.01-0.018 | 3.00E-13 |
| CigDay | rs2133203 | Cigarettes smoked per day | 36477530 | chr1:77974530 | C=0.55, T=0.45 | 0.409 | 0.01 | 0.0067-0.0133 | 3.00E-12 |
| CigDay | rs1737894 | Cigarettes smoked per day (MTAG) | 30643251 | chr20:31054702 | C=0.622, G=0.378 | 0.408 | 0.0172715 | 0.013-0.022 | 8.00E-15 |
| CigDay | rs1737894 | Smoking behaviour (cigarettes smoked per day) | 30643251 | chr20:31054702 | C=0.622, G=0.378 | 0.408 | 0.016856 | 0.012-0.022 | 1.00E-11 |
| CigDay | rs1737894 | Insomnia | 35835914 | chr20:31054702 | C=0.622, G=0.378 | NR | 0.01 | 0.008-0.012 | 1.00E-15 |
| CigDay | rs2273500 | Parental longevity (father's attained age) | 29227965 | chr20:61986949 | C=0.146, T=0.854 | 0.856 | 0.0176 | 0.012-0.024 | 2.00E-08 |
| CigDay | rs2273500 | Parental longevity (combined parental attained age, Martingale residuals) | 29227965 | chr20:61986949 | C=0.146, T=0.854 | NR | NA | NA | 4.00E-07 |
| CigDay | rs2273500 | Nicotine dependence | 26440539 | chr20:61986949 | C=0.146, T=0.854 | 0.15 | 0.058 | NA | 8.00E-09 |
| CigDay | rs2273500 | Chronic obstructive pulmonary disease | 34594039 | chr20:61986949 | C=0.146, T=0.854 | NR | 0.0947 | 0.064-0.125 | 2.00E-09 |
| CigDay | rs2273500 | Nicotine dependence | 28972577 | chr20:61986949 | C=0.146, T=0.854 | NR | 0.046 | 0.027-0.065 | 2.00E-06 |
| CigDay | rs2273500 | Cigarettes smoked per day (MTAG) | 30643251 | chr20:61986949 | C=0.146, T=0.854 | 0.147 | 0.041068 | 0.035-0.047 | 2.00E-39 |
| CigDay | rs2273500 | Smoking behaviour (cigarettes smoked per day) | 30643251 | chr20:61986949 | C=0.146, T=0.854 | 0.147 | 0.036385704 | 0.03-0.043 | 3.00E-26 |
| CigDay | rs2273500 | Cigarettes smoked per day | 36477530 | chr20:61986949 | C=0.146, T=0.854 | 0.151 | 0.0404 | 0.035-0.045 | 8.00E-58 |
| CigDay | rs2273500 | Problematic tobacco use | 37250466 | chr20:61986949 | C=0.146, T=0.854 | NR | NA | NA | 1.00E-16 |
| CigDay | rs2273500 | Cigarettes smoked per day (MTAG) | 36376304 | chr20:61986949 | C=0.146, T=0.854 | NR | 0.03106 | 0.024-0.038 | 3.00E-20 |
| CigDay | rs2273500 | Cigarettes smoked per day | 36477530 | chr20:61986949 | C=0.146, T=0.854 | 0.149 | 0.0382 | 0.034-0.043 | 3.00E-64 |
| CigDay | rs145104523 | General cognitive ability | 29844566 | chr21:40644110 | C=0.874, T=0.126 | NR | 5.456 | - | 5.00E-08 |
| CigDay | rs145104523 | Intelligence | 29942086 | chr21:40644110 | C=0.874, T=0.126 | NR | 6.165 | - | 7.00E-10 |
| CigDay | rs145104523 | Attention deficit hyperactivity disorder or autism spectrum disorder or intelligence (pleiotropy) | 35764056 | chr21:40644110 | C=0.874, T=0.126 | NR | NA | NA | 6.00E-10 |
| CigDay | rs145104523 | Body mass index | 36581621 | chr21:40644110 | C=0.874, T=0.126 | NR | 0.0146 | 0.0097-0.0195 | 3.00E-09 |
| CigDay | rs11686893 | Neuroticism | 29942085 | chr2:148495050 | C=0.286, T=0.714 | NR | 6.22 | NA | 5.00E-10 |
| CigDay | rs7599488 | Schizophrenia (MTAG) | 32606422 | chr2:60718347 | C=0.583, T=0.417 | NR | NA | NA | 2.00E-09 |
| CigDay | rs7599488 | Mean corpuscular volume | 32888494 | chr2:60718347 | C=0.583, T=0.417 | 0.427125 | 0.0504438 | 0.046-0.055 | 4.00E-44 |
| CigDay | rs7599488 | Mean spheric corpuscular volume | 32888494 | chr2:60718347 | C=0.583, T=0.417 | 0.427254 | 0.03289241 | 0.028-0.038 | 3.00E-39 |
| CigDay | rs7599488 | Mean corpuscular hemoglobin | 32888494 | chr2:60718347 | C=0.583, T=0.417 | 0.42711 | 0.031699445 | 0.027-0.037 | 2.00E-37 |
| CigDay | rs7599488 | General cognitive ability | 29844566 | chr2:60718347 | C=0.583, T=0.417 | NR | 5.897 | - | 4.00E-09 |
| CigDay | rs7599488 | Cognitive ability, years of educational attainment or schizophrenia (pleiotropy) | 31374203 | chr2:60718347 | C=0.583, T=0.417 | NR | 0.016694613 | 0.013-0.021 | 4.00E-16 |
| CigDay | rs7599488 | Schizophrenia | 31740837 | chr2:60718347 | C=0.583, T=0.417 | NR | 1.0519007 | 1.03-1.07 | 3.00E-08 |
| CigDay | rs7599488 | Household income (MTAG) | 31844048 | chr2:60718347 | C=0.583, T=0.417 | 0.4236 | 0.01609844 | 0.012-0.02 | 3.00E-15 |
| CigDay | rs7599488 | Schizophrenia | 26198764 | chr2:60718347 | C=0.583, T=0.417 | NR | 1.0526316 | NA | 6.00E-07 |
| CigDay | rs7599488 | Cigarettes smoked per day (MTAG) | 30643251 | chr2:60718347 | C=0.583, T=0.417 | 0.437 | 0.013978 | 0.0097-0.0183 | 2.00E-10 |
| CigDay | rs7599488 | Smoking behaviour (cigarettes smoked per day) | 30643251 | chr2:60718347 | C=0.583, T=0.417 | 0.437 | 0.014121279 | 0.0093-0.0189 | 9.00E-09 |
| CigDay | rs7599488 | Alzheimer's disease or educational attainment (pleiotropy) | 34743297 | chr2:60718347 | C=0.583, T=0.417 | 0.4254 | 56.879543 | NA | 1.00E-11 |
| CigDay | rs7599488 | Cigarettes smoked per day | 36477530 | chr2:60718347 | C=0.583, T=0.417 | 0.434 | 0.0147 | 0.011-0.018 | 6.00E-16 |
| CigDay | rs7599488 | Schizophrenia | 35396580 | chr2:60718347 | C=0.583, T=0.417 | 0.524 | 1.04404 | 1.03-1.06 | 1.00E-08 |
| CigDay | rs7599488 | Cigarettes smoked per day | 36477530 | chr2:60718347 | C=0.583, T=0.417 | 0.485 | 0.0154 | 0.012-0.019 | 2.00E-21 |
| CigDay | rs7599488 | Short sleep duration (<5 hours) | 37770476 | chr2:60718347 | C=0.583, T=0.417 | NR | 0.036 | 0.023-0.049 | 4.00E-08 |
| CigDay | rs7599488 | Personality traits or cognitive traits (multivariate analysis) | 37365406 | chr2:60718347 | C=0.583, T=0.417 | NR | NA | NA | 2.00E-16 |
| CigDay | rs7599488 | Cigarettes smoked per day (MTAG) | 36376304 | chr2:60718347 | C=0.583, T=0.417 | NR | 0.013872 | 0.009-0.0187 | 2.00E-08 |
| CigDay | rs9881798 | Neuroticism | 29942085 | chr3:16846967 | A=0.608, C=0.392 | NR | 6.123 | NA | 9.00E-10 |
| CigDay | rs9881798 | Schizophrenia | 26198764 | chr3:16846967 | A=0.608, C=0.392 | NR | 1.0526316 | NA | 7.00E-06 |
| CigDay | rs9881798 | Cigarettes smoked per day | 36477530 | chr3:16846967 | A=0.608, C=0.392 | 0.414 | 0.0151 | 0.012-0.019 | 2.00E-16 |
| CigDay | rs9881798 | Cigarettes smoked per day | 36477530 | chr3:16846967 | A=0.608, C=0.392 | 0.397 | 0.0131 | 0.0099-0.0163 | 1.00E-15 |
| CigDay | rs145483821 | Cigarettes smoked per day | 36477530 | chr3:89344540 | G=0.439, T=0.561 | 0.46 | 0.0119 | 0.0081-0.0157 | 9.00E-10 |
| CigDay | rs11725618 | Cognitive ability, years of educational attainment or schizophrenia (pleiotropy) | 31374203 | chr4:67053769 | C=0.313, T=0.687 | NR | 0.012540856 | 0.0082-0.0169 | 1.00E-08 |
| CigDay | rs11725618 | Cigarettes smoked per day | 36477530 | chr4:67053769 | C=0.313, T=0.687 | 0.283 | 0.0196 | 0.016-0.024 | 1.00E-22 |
| CigDay | rs11725618 | Cigarettes smoked per day (MTAG) | 36376304 | chr4:67053769 | C=0.313, T=0.687 | NR | 0.01733 | 0.012-0.023 | 1.00E-10 |
| CigDay | rs11725618 | Cigarettes smoked per day | 36477530 | chr4:67053769 | C=0.313, T=0.687 | 0.269 | 0.0179 | 0.014-0.021 | 8.00E-23 |
| CigDay | rs6831786 | Cigarettes smoked per day | 36477530 | chr4:67875548 | A=0.559, C=0.441 | 0.576 | 0.0129 | 0.0097-0.0161 | 3.00E-16 |
| CigDay | rs215600 | Cognitive ability | 29186694 | chr7:32333642 | A=0.669, G=0.331 | NR | 6.289 | NA | 3.00E-10 |
| CigDay | rs215600 | Cognitive ability (MTAG) | 29186694 | chr7:32333642 | A=0.669, G=0.331 | NR | 6.999 | NA | 3.00E-12 |
| CigDay | rs215600 | Cigarettes smoked per day (MTAG) | 30643251 | chr7:32333642 | A=0.669, G=0.331 | 0.645 | 0.0243837 | 0.02-0.029 | 8.00E-27 |
| CigDay | rs215600 | Smoking behaviour (cigarettes smoked per day) | 30643251 | chr7:32333642 | A=0.669, G=0.331 | 0.645 | 0.024002468 | 0.019-0.029 | 4.00E-21 |
| CigDay | rs215600 | Cigarettes smoked per day | 36477530 | chr7:32333642 | A=0.669, G=0.331 | 0.644 | 0.0214 | 0.018-0.025 | 5.00E-30 |
| CigDay | rs215600 | Problematic tobacco use | 37250466 | chr7:32333642 | A=0.669, G=0.331 | NR | NA | NA | 2.00E-18 |
| CigDay | rs215600 | Cigarettes smoked per day (MTAG) | 36376304 | chr7:32333642 | A=0.669, G=0.331 | NR | 0.02425 | 0.019-0.029 | 2.00E-21 |
| CigDay | rs73229090 | Schizophrenia | 31740837 | chr8:27442127 | A=0.113, C=0.887 | NR | 1.1088196 | 1.07-1.14 | 1.00E-08 |
| CigDay | rs73229090 | Schizophrenia | 25056061 | chr8:27442127 | A=0.113, C=0.887 | 0.884 | 1.1013216 | 1.06-1.14 | 2.00E-08 |
| CigDay | rs73229090 | Schizophrenia | 26198764 | chr8:27442127 | A=0.113, C=0.887 | NR | 1.098901 | NA | 2.00E-08 |
| CigDay | rs73229090 | Smoking behaviour (cigarettes smoked per day) | 30643251 | chr8:27442127 | A=0.113, C=0.887 | 0.112 | 0.026211383 | 0.019-0.034 | 1.00E-11 |
| CigDay | rs73229090 | Smoking initiation | 36477530 | chr8:27442127 | A=0.113, C=0.887 | 0.116 | 0.0315 | 0.029-0.034 | 4.00E-120 |
| CigDay | rs73229090 | Schizophrenia | 35396580 | chr8:27442127 | A=0.113, C=0.887 | 0.902 | 1.10131 | 1.07-1.13 | 2.00E-12 |
| CigDay | rs73229090 | Cigarettes smoked per day (MTAG) | 36376304 | chr8:27442127 | A=0.113, C=0.887 | NR | 0.023214 | 0.016-0.031 | 3.00E-09 |
| CigDay | rs3025383 | Post bronchodilator FEV1 | 26634245 | chr9:136502369 | C=0.181, T=0.819 | 0.718 | 0.051 | NA | 9.00E-07 |
| CigDay | rs3025383 | Post bronchodilator FEV1/FVC ratio | 26634245 | chr9:136502369 | C=0.181, T=0.819 | 0.715 | 0.01 | NA | 4.00E-06 |
| CigDay | rs3025383 | Cigarettes smoked per day (MTAG) | 30643251 | chr9:136502369 | C=0.181, T=0.819 | 0.187 | 0.0314888 | 0.026-0.037 | 5.00E-29 |
| CigDay | rs3025383 | Smoking behaviour (cigarettes smoked per day) | 30643251 | chr9:136502369 | C=0.181, T=0.819 | 0.187 | 0.031379756 | 0.025-0.037 | 1.00E-23 |
| CigDay | rs3025383 | Cigarettes smoked per day (MTAG) | 36376304 | chr9:136502369 | C=0.181, T=0.819 | NR | 0.023232 | 0.017-0.03 | 5.00E-13 |
| CigDay | rs3025383 | Cigarettes smoked per day | 36477530 | chr9:136502369 | C=0.181, T=0.819 | 0.184 | 0.0267 | 0.023-0.031 | 1.00E-38 |
| SmkCes | rs2118362 | Multisite chronic pain | 33830993 | chr11:16373083 | C=0.287, T=0.713 | NR | NA | NA | 1.00E-08 |
| SmkCes | rs2006281 | Multisite chronic pain | 31194737 | chr14:104327732 | C=0.465, T=0.535 | 0.4981 | 0.0135 | 0.0088-0.0182 | 3.00E-08 |
| SmkCes | rs591143 | Smoking cessation (MTAG) | 30643251 | chr15:47647755 | C=0.371, T=0.629 | 0.592 | 0.0136814 | 0.011-0.017 | 3.00E-18 |
| SmkCes | rs591143 | Smoking cessation | 30643251 | chr15:47647755 | C=0.371, T=0.629 | 0.592 | 0.0243296 | 0.017-0.032 | 1.00E-09 |
| SmkCes | rs72740955 | Post bronchodilator FEV1 | 26634245 | chr15:78849779 | C=0.626, T=0.374 | 0.338 | 0.075 | NA | 1.00E-14 |
| SmkCes | rs72740955 | Post bronchodilator FEV1/FVC ratio in COPD | 26634245 | chr15:78849779 | C=0.626, T=0.374 | 0.402 | 0.012 | NA | 7.00E-07 |
| SmkCes | rs72740955 | Smoking cessation | 33082346 | chr15:78849779 | C=0.626, T=0.374 | NR | 0.97 | NA | 9.00E-10 |
| SmkCes | rs72740955 | Smoking behaviour (cigarettes smoked per day) | 32157176 | chr15:78849779 | C=0.626, T=0.374 | 0.349 | 0.142 | 0.1-0.18 | 7.00E-12 |
| SmkCes | rs72740955 | Smoking behaviour (cigarette pack-years) | 32157176 | chr15:78849779 | C=0.626, T=0.374 | 0.35 | 0.106 | 0.073-0.139 | 8.00E-10 |
| SmkCes | rs72740955 | Post bronchodilator FEV1/FVC ratio | 26634245 | chr15:78849779 | C=0.626, T=0.374 | 0.335 | 0.016 | NA | 7.00E-16 |
| SmkCes | rs72740955 | Smoking cessation (MTAG) | 30643251 | chr15:78849779 | C=0.626, T=0.374 | 0.335 | 0.0237025 | 0.021-0.027 | 5.00E-48 |
| SmkCes | rs72740955 | Smoking behaviour (cigarettes smoked per day) | 30643251 | chr15:78849779 | C=0.626, T=0.374 | 0.337 | 0.031752326 | 0.027-0.037 | 2.00E-34 |
| SmkCes | rs56113850 | Smoking behaviour (cigarettes smoked per day) | 30679032 | chr19:41353107 | C=0.592, T=0.408 | 0.575 | 0.0719 | 0.064-0.08 | 3.00E-68 |
| SmkCes | rs56113850 | Smoking behaviour (cigarette pack-years) | 30679032 | chr19:41353107 | C=0.592, T=0.408 | 0.572 | 0.0385 | 0.031-0.046 | 6.00E-25 |
| SmkCes | rs56113850 | Caffeine consumption from coffee | 33287642 | chr19:41353107 | C=0.592, T=0.408 | 0.421821 | 0.02071 | 0.016-0.025 | 5.00E-19 |
| SmkCes | rs56113850 | Caffeine consumption from coffee or tea | 33287642 | chr19:41353107 | C=0.592, T=0.408 | 0.421755 | 0.02649 | 0.022-0.031 | 2.00E-29 |
| SmkCes | rs56113850 | Post bronchodilator FEV1 | 26634245 | chr19:41353107 | C=0.592, T=0.408 | 0.458 | 0.067 | NA | 2.00E-08 |
| SmkCes | rs56113850 | Nicotine metabolite ratio in current smokers | 27488534 | chr19:41353107 | C=0.592, T=0.408 | NR | 0.3644 | 0.32-0.41 | 1.00E-50 |
| SmkCes | rs56113850 | Post bronchodilator FEV1/FVC ratio | 26634245 | chr19:41353107 | C=0.592, T=0.408 | 0.462 | 0.014 | NA | 5.00E-09 |
| SmkCes | rs56113850 | Caffeine metabolism (plasma 1,7-dimethylxanthine (paraxanthine) to 1,3,7-trimethylxanthine (caffeine) ratio) | 27702941 | chr19:41353107 | C=0.592, T=0.408 | 0.43 | 9.59 | - | 9.00E-22 |
| SmkCes | rs56113850 | Caffeine metabolism (plasma 1,7-dimethylxanthine (paraxanthine) level) | 27702941 | chr19:41353107 | C=0.592, T=0.408 | 0.43 | 5.43 | NA | 6.00E-08 |
| SmkCes | rs56113850 | Caffeine metabolism (plasma 3,7-dimethylxanthine (theobromine) level) | 27702941 | chr19:41353107 | C=0.592, T=0.408 | 0.43 | 4.59 | - | 4.00E-06 |
| SmkCes | rs56113850 | Nicotine metabolite ratio in current smokers | 26407342 | chr19:41353107 | C=0.592, T=0.408 | NR | 0.653 | 0.59-0.72 | 6.00E-86 |
| SmkCes | rs56113850 | LDL cholesterol levels | 32203549 | chr19:41353107 | C=0.592, T=0.408 | 0.422594 | 0.013364 | 0.0093-0.0175 | 2.00E-10 |
| SmkCes | rs56113850 | Smoking cessation | 33082346 | chr19:41353107 | C=0.592, T=0.408 | NR | 0.946 | NA | 7.00E-33 |
| SmkCes | rs56113850 | Apolipoprotein B levels | 32203549 | chr19:41353107 | C=0.592, T=0.408 | 0.42254 | 0.0135934 | 0.0095-0.0177 | 6.00E-11 |
| SmkCes | rs56113850 | Local histogram emphysema pattern | 25006744 | chr19:41353107 | C=0.592, T=0.408 | 0.4 | 0.02 | 0.012-0.028 | 1.00E-09 |
| SmkCes | rs56113850 | Local histogram emphysema pattern | 25006744 | chr19:41353107 | C=0.592, T=0.408 | 0.4 | 0.02 | 0.012-0.028 | 1.00E-06 |
| SmkCes | rs56113850 | Lung cancer | 28604730 | chr19:41353107 | C=0.592, T=0.408 | 0.559578448 | 1.1306498 | 1.1-1.16 | 5.00E-19 |
| SmkCes | rs56113850 | Lung adenocarcinoma | 28604730 | chr19:41353107 | C=0.592, T=0.408 | 0.555412482 | 1.121034 | 1.08-1.16 | 9.00E-10 |
| SmkCes | rs56113850 | Squamous cell lung carcinoma | 28604730 | chr19:41353107 | C=0.592, T=0.408 | 0.55529151 | 1.1555213 | 1.11-1.21 | 7.00E-11 |
| SmkCes | rs56113850 | Lung cancer in ever smokers | 28604730 | chr19:41353107 | C=0.592, T=0.408 | 0.564992436 | 1.1372905 | 1.1-1.18 | 4.00E-13 |
| SmkCes | rs56113850 | Lung adenocarcinoma | 32889700 | chr19:41353107 | C=0.592, T=0.408 | NR | 0.93 | 0.89-0.96 | 8.00E-06 |
| SmkCes | rs56113850 | Non-small cell lung cancer | 32889700 | chr19:41353107 | C=0.592, T=0.408 | NR | 0.92 | 0.89-0.94 | 3.00E-09 |
| SmkCes | rs56113850 | Nicotine metabolite ratio in current smokers | 32157176 | chr19:41353107 | C=0.592, T=0.408 | 0.447 | 0.682 | 0.64-0.72 | 6.00E-261 |
| SmkCes | rs56113850 | Smoking intensity (cotinine levels/cigarettes per day) | 32157176 | chr19:41353107 | C=0.592, T=0.408 | 0.447 | 0.155 | 0.11-0.2 | 3.00E-13 |
| SmkCes | rs56113850 | Plasma letrozole concentrations in letrozole treated hormone-receptor-positive breast cancer | 34096894 | chr19:41353107 | C=0.592, T=0.408 | 0.46 | 4693.72 | 3102.87-6284.57 | 2.00E-13 |
| SmkCes | rs56113850 | Nicotine dependence | 33144568 | chr19:41353107 | C=0.592, T=0.408 | NR | 0.031 | 0.023-0.039 | 4.00E-16 |
| SmkCes | rs56113850 | Aerodigestive squamous cell cancer (pleiotropy) | 33667223 | chr19:41353107 | C=0.592, T=0.408 | 0.41 | 0.9 | 0.86-0.94 | 7.00E-11 |
| SmkCes | rs56113850 | Linoleic acid levels | 35213538 | chr19:41353107 | C=0.592, T=0.408 | 0.420788 | 0.0239432 | 0.016-0.032 | 6.00E-09 |
| SmkCes | rs56113850 | Glycine levels | 35213538 | chr19:41353107 | C=0.592, T=0.408 | 0.420828 | 0.0218076 | 0.014-0.03 | 3.00E-08 |
| SmkCes | rs56113850 | Apolipoprotein B levels | 35213538 | chr19:41353107 | C=0.592, T=0.408 | 0.420796 | 0.0239612 | 0.016-0.032 | 9.00E-09 |
| SmkCes | rs56113850 | Cholesterol levels in small VLDL | 35213538 | chr19:41353107 | C=0.592, T=0.408 | 0.420796 | 0.0240384 | 0.016-0.032 | 8.00E-09 |
| SmkCes | rs56113850 | Free cholesterol levels in small VLDL | 35213538 | chr19:41353107 | C=0.592, T=0.408 | 0.420796 | 0.0235212 | 0.015-0.032 | 2.00E-08 |
| SmkCes | rs56113850 | Free cholesterol levels in very small VLDL | 35213538 | chr19:41353107 | C=0.592, T=0.408 | 0.420796 | 0.0231331 | 0.015-0.031 | 2.00E-08 |
| SmkCes | rs56113850 | Phospholipid levels in small LDL | 35213538 | chr19:41353107 | C=0.592, T=0.408 | 0.420796 | 0.0243826 | 0.016-0.033 | 4.00E-09 |
| SmkCes | rs56113850 | Concentration of small LDL particles | 35213538 | chr19:41353107 | C=0.592, T=0.408 | 0.420796 | 0.0241421 | 0.016-0.032 | 8.00E-09 |
| SmkCes | rs56113850 | Concentration of very small VLDL particles | 35213538 | chr19:41353107 | C=0.592, T=0.408 | 0.420796 | 0.0238214 | 0.016-0.032 | 7.00E-09 |
| SmkCes | rs56113850 | Total lipid levels in very small VLDL | 35213538 | chr19:41353107 | C=0.592, T=0.408 | 0.420796 | 0.0224965 | 0.014-0.031 | 4.00E-08 |
| SmkCes | rs56113850 | Phospholipid levels in small VLDL | 35213538 | chr19:41353107 | C=0.592, T=0.408 | 0.420796 | 0.0231974 | 0.015-0.031 | 3.00E-08 |
| SmkCes | rs56113850 | Cholesteryl ester levels in small VLDL | 35213538 | chr19:41353107 | C=0.592, T=0.408 | 0.420796 | 0.0240607 | 0.016-0.032 | 8.00E-09 |
| SmkCes | rs56113850 | Total lipid levels in small LDL | 35213538 | chr19:41353107 | C=0.592, T=0.408 | 0.420796 | 0.0237331 | 0.016-0.032 | 1.00E-08 |
| SmkCes | rs56113850 | Phospholipid levels in very small VLDL | 35213538 | chr19:41353107 | C=0.592, T=0.408 | 0.420796 | 0.0234529 | 0.015-0.031 | 1.00E-08 |
| SmkCes | rs56113850 | Serum alkaline phosphatase levels | 34594039 | chr19:41353107 | C=0.592, T=0.408 | NR | 0.0472 | 0.042-0.052 | 1.00E-89 |
| SmkCes | rs56113850 | Estimated glomerular filtration rate (creatinine) | 35710981 | chr19:41353107 | C=0.592, T=0.408 | 0.5599 | 7.151 | NA | 9.00E-13 |
| SmkCes | rs56113850 | Chronotype | 30696823 | chr19:41353107 | C=0.592, T=0.408 | 0.5769 | 1.0231643 | NA | 2.00E-10 |
| SmkCes | rs56113850 | Bitter non-alcoholic beverage consumption | 31046077 | chr19:41353107 | C=0.592, T=0.408 | 0.5766 | 0.0107 | 0.0076-0.0138 | 7.00E-11 |
| SmkCes | rs56113850 | Coffee consumption | 31046077 | chr19:41353107 | C=0.592, T=0.408 | 0.5776 | 0.0121 | 0.0092-0.015 | 8.00E-16 |
| SmkCes | rs56113850 | Bitter beverage consumption | 31046077 | chr19:41353107 | C=0.592, T=0.408 | 0.5766 | 0.0107 | 0.0076-0.0138 | 8.00E-11 |
| SmkCes | rs56113850 | Cigarettes smoked per day (MTAG) | 30643251 | chr19:41353107 | C=0.592, T=0.408 | 0.555 | 0.0361686 | 0.032-0.041 | 5.00E-58 |
| SmkCes | rs56113850 | Smoking cessation (MTAG) | 30643251 | chr19:41353107 | C=0.592, T=0.408 | 0.567 | 0.00974331 | 0.0067-0.0128 | 6.00E-10 |
| SmkCes | rs56113850 | Smoking behaviour (cigarettes smoked per day) | 30643251 | chr19:41353107 | C=0.592, T=0.408 | 0.555 | 0.0523096 | 0.047-0.057 | 4.00E-99 |
| SmkCes | rs56113850 | Smoking cessation | 30643251 | chr19:41353107 | C=0.592, T=0.408 | 0.567 | 0.05760633 | 0.05-0.065 | 2.00E-48 |
| SmkCes | rs56113850 | Ratio of apolipoprotein B to apolipoprotein A1 levels | 35213538 | chr19:41353107 | C=0.592, T=0.408 | 0.420796 | 0.0227187 | 0.015-0.031 | 3.00E-08 |
| SmkCes | rs56113850 | Concentration of LDL particles | 35213538 | chr19:41353107 | C=0.592, T=0.408 | 0.420796 | 0.0237631 | 0.016-0.032 | 1.00E-08 |
| SmkCes | rs56113850 | Triglyceride levels in large LDL | 35213538 | chr19:41353107 | C=0.592, T=0.408 | 0.420796 | 0.0230106 | 0.015-0.031 | 2.00E-08 |
| SmkCes | rs56113850 | Concentration of large LDL particles | 35213538 | chr19:41353107 | C=0.592, T=0.408 | 0.420796 | 0.0237825 | 0.016-0.032 | 1.00E-08 |
| SmkCes | rs56113850 | Non-small cell lung cancer | 31326317 | chr19:41353107 | C=0.592, T=0.408 | 0.487 | 1.09 | 1.06-1.12 | 3.00E-09 |
| SmkCes | rs56113850 | Lung adenocarcinoma | 35915169 | chr19:41353107 | C=0.592, T=0.408 | NR | 0.07 | 0.031-0.109 | 8.00E-11 |
| SmkCes | rs56113850 | Squamous cell lung carcinoma | 35915169 | chr19:41353107 | C=0.592, T=0.408 | NR | 0.09 | 0.031-0.149 | 3.00E-11 |
| SmkCes | rs56113850 | Lung cancer | 35915169 | chr19:41353107 | C=0.592, T=0.408 | NR | 0.08 | 0.041-0.119 | 8.00E-27 |
| SmkCes | rs56113850 | Urine X-21831 levels in chronic kidney disease | 37277652 | chr19:41353107 | C=0.592, T=0.408 | 0.59 | 0.469 | 0.39-0.55 | 8.00E-31 |
| SmkCes | rs56113850 | Urine X-18935 levels in chronic kidney disease | 37277652 | chr19:41353107 | C=0.592, T=0.408 | 0.58 | 0.294 | 0.24-0.34 | 1.00E-29 |
| SmkCes | rs56113850 | Alanine aminotransferase levels | 33462484 | chr19:41353107 | C=0.592, T=0.408 | NR | 0.0167 | 0.012-0.021 | 3.00E-12 |
| SmkCes | rs56113850 | Nicotine metabolite ratio in current smokers | 34599228 | chr19:41353107 | C=0.592, T=0.408 | NR | 0.671 | 0.54-0.8 | 7.00E-22 |
| SmkCes | rs56113850 | Nicotine metabolite ratio in current smokers | 34599228 | chr19:41353107 | C=0.592, T=0.408 | NR | 0.748 | 0.64-0.85 | 1.00E-37 |
| SmkCes | rs56113850 | Liver enzyme levels (alanine transaminase) | 33972514 | chr19:41353107 | C=0.592, T=0.408 | 0.422385 | 0.00312488 | 0.0024-0.0039 | 1.00E-15 |
| SmkCes | rs56113850 | Urine X-12126 levels in chronic kidney disease | 37277652 | chr19:41353107 | C=0.592, T=0.408 | 0.58 | 0.189 | 0.14-0.24 | 3.00E-14 |
| SmkCes | rs56113850 | Plasma X-12216 levels in chronic kidney disease | 37277652 | chr19:41353107 | C=0.592, T=0.408 | 0.59 | 0.173 | 0.12-0.22 | 8.00E-12 |
| SmkCes | rs56113850 | F-chocolate/coffee liking (derived food-liking factor) | 35585065 | chr19:41353107 | C=0.592, T=0.408 | 0.5774 | 0.090892 | 0.062-0.12 | 1.00E-09 |
| SmkCes | rs56113850 | Low density lipoprotein cholesterol levels | 34887591 | chr19:41353107 | C=0.592, T=0.408 | NR | NA | NA | 3.00E-17 |
| SmkCes | rs56113850 | Low density lipoprotein cholesterol levels | 34887591 | chr19:41353107 | C=0.592, T=0.408 | 0.567435 | 0.0142152 | 0.011-0.017 | 2.00E-16 |
| SmkCes | rs56113850 | Coffee consumption (cups per day) | 32193382 | chr19:41353107 | C=0.592, T=0.408 | 0.422202 | 0.0186901 | 0.015-0.023 | 8.00E-19 |
| SmkCes | rs56113850 | X-12216 levels | 35347128 | chr19:41353107 | C=0.592, T=0.408 | NR | 0.15 | NA | 3.00E-15 |
| SmkCes | rs56113850 | Theobromine levels | 35347128 | chr19:41353107 | C=0.592, T=0.408 | NR | 0.13 | NA | 3.00E-12 |
| SmkCes | rs56113850 | Paraxanthine levels | 35347128 | chr19:41353107 | C=0.592, T=0.408 | NR | 0.17 | NA | 2.00E-19 |
| SmkCes | rs56113850 | 3-methylxanthine levels | 35347128 | chr19:41353107 | C=0.592, T=0.408 | NR | 0.16 | NA | 9.00E-18 |
| SmkCes | rs56113850 | 6-hydroxyindole sulfate levels | 35347128 | chr19:41353107 | C=0.592, T=0.408 | NR | 0.12 | NA | 2.00E-11 |
| SmkCes | rs56113850 | Indolin-2-one levels | 35347128 | chr19:41353107 | C=0.592, T=0.408 | NR | 0.17 | NA | 7.00E-15 |
| SmkCes | rs56113850 | 3-methylxanthine levels | 35050183 | chr19:41353107 | C=0.592, T=0.408 | NR | 0.155 | 0.13-0.18 | 2.00E-24 |
| SmkCes | rs56113850 | X-21286 levels | 35050183 | chr19:41353107 | C=0.592, T=0.408 | NR | 0.104 | 0.075-0.133 | 8.00E-12 |
| SmkCes | rs56113850 | X-12216 levels | 35050183 | chr19:41353107 | C=0.592, T=0.408 | NR | 0.139 | 0.11-0.17 | 4.00E-20 |
| SmkCes | rs56113850 | 6-hydroxyindole sulfate levels | 36357675 | chr19:41353107 | C=0.592, T=0.408 | 0.4287 | 0.09685272 | 0.076-0.117 | 2.00E-20 |
| SmkCes | rs56113850 | X-21286 levels | 36357675 | chr19:41353107 | C=0.592, T=0.408 | 0.4289 | 0.13240077 | 0.11-0.15 | 7.00E-38 |
| SmkCes | rs56113850 | Theobromine levels | 36357675 | chr19:41353107 | C=0.592, T=0.408 | 0.4286 | 0.09372097 | 0.074-0.114 | 8.00E-20 |
| SmkCes | rs56113850 | X-12216 levels | 36357675 | chr19:41353107 | C=0.592, T=0.408 | 0.4278 | 0.1274714 | 0.11-0.15 | 7.00E-35 |
| SmkCes | rs56113850 | X-12126 levels | 36357675 | chr19:41353107 | C=0.592, T=0.408 | 0.4165 | 0.1277143 | 0.1-0.15 | 6.00E-26 |
| SmkCes | rs56113850 | Indolin-2-one levels | 36357675 | chr19:41353107 | C=0.592, T=0.408 | 0.4373 | 0.1445022 | 0.12-0.17 | 1.00E-30 |
| SmkCes | rs56113850 | 3-methylxanthine levels | 36357675 | chr19:41353107 | C=0.592, T=0.408 | 0.4288 | 0.14389715 | 0.12-0.16 | 3.00E-44 |
| SmkCes | rs56113850 | 3-hydroxycotinine glucuronide levels | 36357675 | chr19:41353107 | C=0.592, T=0.408 | 0.4184 | 0.26954755 | 0.2-0.34 | 3.00E-15 |
| SmkCes | rs56113850 | Paraxanthine levels | 36357675 | chr19:41353107 | C=0.592, T=0.408 | 0.4292 | 0.11057526 | 0.09-0.131 | 1.00E-26 |
| SmkCes | rs56113850 | 1,7-dimethylurate levels | 36357675 | chr19:41353107 | C=0.592, T=0.408 | 0.4287 | 0.10510567 | 0.085-0.125 | 3.00E-24 |
| SmkCes | rs56113850 | Smoking cessation | 36477530 | chr19:41353107 | C=0.592, T=0.408 | 0.572 | 0.0303 | 0.028-0.033 | 5.00E-114 |
| SmkCes | rs56113850 | Non-HDL cholesterol levels | 34887591 | chr19:41353107 | C=0.592, T=0.408 | 0.562613 | 0.014895 | 0.012-0.018 | 2.00E-13 |
| SmkCes | rs56113850 | Cigarettes smoked per day | 36477530 | chr19:41353107 | C=0.592, T=0.408 | 0.565 | 0.0557 | 0.052-0.059 | 6.00E-207 |
| SmkCes | rs56113850 | C-reactive protein levels (MTAG) | 36376304 | chr19:41353107 | C=0.592, T=0.408 | NR | 0.01645 | 0.012-0.02 | 8.00E-16 |
| SmkCes | rs56113850 | Non-HDL cholesterol levels | 34887591 | chr19:41353107 | C=0.592, T=0.408 | NR | NA | NA | 2.00E-14 |
| SmkCes | rs56113850 | Smoking cessation | 36477530 | chr19:41353107 | C=0.592, T=0.408 | 0.563 | 0.0303 | 0.028-0.033 | 1.00E-120 |
| SmkCes | rs56113850 | Indolin-2-one levels | 36635386 | chr19:41353107 | C=0.592, T=0.408 | 0.570252 | 0.167321 | 0.14-0.2 | 2.00E-24 |
| SmkCes | rs56113850 | X-18935 levels | 36635386 | chr19:41353107 | C=0.592, T=0.408 | 0.606272 | 0.131701 | 0.092-0.171 | 8.00E-11 |
| SmkCes | rs56113850 | X-21286 levels | 36635386 | chr19:41353107 | C=0.592, T=0.408 | 0.576068 | 0.136846 | 0.11-0.17 | 2.00E-19 |
| SmkCes | rs56113850 | X-12216 levels | 36635386 | chr19:41353107 | C=0.592, T=0.408 | 0.577382 | 0.129952 | 0.1-0.16 | 7.00E-18 |
| SmkCes | rs56113850 | Alanine aminotransferase levels | 38632349 | chr19:41353107 | C=0.592, T=0.408 | NR | 0.0034 | 0.0026-0.0042 | 6.00E-18 |
| SmkCes | rs56113850 | Cigarettes smoked per day (MTAG) | 36376304 | chr19:41353107 | C=0.592, T=0.408 | NR | 0.04714 | 0.042-0.052 | 2.00E-78 |
| SmkCes | rs56113850 | Glycine levels (UKB data field 23462) | 36764567 | chr19:41353107 | C=0.592, T=0.408 | 0.420048 | 0.0279433 | 0.019-0.037 | 7.00E-10 |
| SmkCes | rs56113850 | Cigarettes smoked per day | 36477530 | chr19:41353107 | C=0.592, T=0.408 | 0.55 | 0.0534 | 0.05-0.057 | 3.00E-214 |
| SmkCes | rs56113850 | 6-hydroxyindole sulfate levels | 36635386 | chr19:41353107 | C=0.592, T=0.408 | 0.57613 | 0.109573 | 0.08-0.139 | 6.00E-13 |
| SmkCes | rs56113850 | Paraxanthine levels | 36635386 | chr19:41353107 | C=0.592, T=0.408 | 0.573947 | 0.111197 | 0.08-0.142 | 3.00E-12 |
| SmkCes | rs56113850 | Theobromine levels | 36635386 | chr19:41353107 | C=0.592, T=0.408 | 0.576193 | 0.105914 | 0.075-0.136 | 1.00E-11 |
| SmkCes | rs56113850 | Metabolite levels (hydroxycotinine) | 37253714 | chr19:41353107 | C=0.592, T=0.408 | 0.5489 | 0.2 | 0.14-0.26 | 2.00E-12 |
| SmkCes | rs10402271 | Total cholesterol levels | 29507422 | chr19:45329214 | G=0.345, T=0.655 | 0.674 | 0.062 | NA | 3.00E-36 |
| SmkCes | rs10402271 | Total cholesterol levels | 29507422 | chr19:45329214 | G=0.345, T=0.655 | NR | 0.057 | NA | 2.00E-36 |
| SmkCes | rs10402271 | High density lipoprotein cholesterol levels | 29507422 | chr19:45329214 | G=0.345, T=0.655 | 0.674 | 0.021 | NA | 2.00E-07 |
| SmkCes | rs10402271 | High density lipoprotein cholesterol levels | 29507422 | chr19:45329214 | G=0.345, T=0.655 | NR | 0.019 | NA | 2.00E-07 |
| SmkCes | rs10402271 | Low density lipoprotein cholesterol levels | 29507422 | chr19:45329214 | G=0.345, T=0.655 | 0.674 | 0.081 | NA | 2.00E-58 |
| SmkCes | rs10402271 | Low density lipoprotein cholesterol levels | 29507422 | chr19:45329214 | G=0.345, T=0.655 | NR | 0.073 | NA | 1.00E-56 |
| SmkCes | rs10402271 | Alzheimer's disease | 30636644 | chr19:45329214 | G=0.345, T=0.655 | NR | 1.3904976 | NA | 7.00E-14 |
| SmkCes | rs10402271 | Alzheimer's disease | 30636644 | chr19:45329214 | G=0.345, T=0.655 | NR | 1.4007349 | NA | 2.00E-09 |
| SmkCes | rs11697662 | Cigarettes smoked per day (current) | 33082346 | chr20:61992005 | C=0.196, T=0.804 | NR | 0.016 | NA | 7.00E-09 |
| SmkCes | rs11697662 | Smoking status (heavy vs never) | 26423011 | chr20:61992005 | C=0.196, T=0.804 | 0.195 | 1.0952903 | 1.06-1.13 | 1.00E-07 |
| SmkCes | rs11697662 | Aerodigestive squamous cell cancer (pleiotropy) | 33667223 | chr20:61992005 | C=0.196, T=0.804 | 0.34 | 1.1 | 1.06-1.14 | 2.00E-06 |
| SmkCes | rs11697662 | Cognitive function (delayed memory) (longitudinal) | 35386118 | chr20:61992005 | C=0.196, T=0.804 | 0.206578 | 0.251271 | 0.14-0.36 | 9.00E-06 |
| SmkCes | rs11697662 | Cigarettes smoked per day | 36477530 | chr20:61992005 | C=0.196, T=0.804 | 0.801 | 0.0347 | 0.03-0.039 | 6.00E-53 |
| SmkCes | rs9607805 | Neurociticism | 29500382 | chr22:41854446 | C=0.296, T=0.704 | 0.287107 | 6.41 | NA | 1.00E-10 |
| SmkCes | rs9607805 | PR interval | 32439900 | chr22:41854446 | C=0.296, T=0.704 | 0.7306 | 0.4352 | 0.29-0.58 | 1.00E-08 |
| SmkCes | rs9607805 | PR interval | 32439900 | chr22:41854446 | C=0.296, T=0.704 | 0.7 | 0.42 | 0.28-0.56 | 6.00E-09 |
| SmkCes | rs9607805 | Highest math class taken (MTAG) | 30038396 | chr22:41854446 | C=0.296, T=0.704 | 0.7217 | 0.0169 | 0.013-0.02 | 5.00E-20 |
| SmkCes | rs9607805 | Smoking cessation | 30643251 | chr22:41854446 | C=0.296, T=0.704 | 0.725 | 0.029540751 | 0.021-0.038 | 1.00E-11 |
| SmkCes | rs9607805 | Neuroticism | 29255261 | chr22:41854446 | C=0.296, T=0.704 | 0.71289 | 7.175 | NA | 7.00E-13 |
| SmkCes | rs9607805 | Insomnia | 35835914 | chr22:41854446 | C=0.296, T=0.704 | NR | 0.009 | 0.007-0.011 | 2.00E-12 |
| SmkCes | rs9607805 | Drinks per week | 36477530 | chr22:41854446 | C=0.296, T=0.704 | 0.696 | 0.0061 | 0.0043-0.0079 | 7.00E-11 |
| SmkCes | rs72781639 | Smoking cessation | 36477530 | chr2:24204148 | C=0.84, G=0.16 | 0.148 | 0.0151 | 0.011-0.019 | 8.00E-16 |
| SmkCes | rs56049603 | Glycated hemoglobin levels | 33462484 | chr3:49585243 | C=0.797, G=0.203 | NR | 0.029 | 0.023-0.035 | 2.00E-22 |
| SmkCes | rs4705014 | Smoking cessation (MTAG) | 30643251 | chr5:155852315 | A=0.663, G=0.337 | 0.64 | 0.0095009 | 0.0064-0.0126 | 3.00E-09 |
| SmkCes | rs4705014 | Smoking cessation | 36477530 | chr5:155852315 | A=0.663, G=0.337 | 0.645 | 0.00863 | 0.0059-0.0113 | 4.00E-10 |
| SmkCes | rs1009181 | Childhood ear infection | 28928442 | chr6:26158993 | C=0.335, T=0.665 | NR | 0.0423 | 0.025-0.06 | 3.00E-06 |
| SmkCes | rs1009181 | Smoking initiation (ever regular vs never regular) (MTAG) | 30643251 | chr6:26158993 | C=0.335, T=0.665 | 0.369 | 0.00983185 | 0.0076-0.012 | 1.00E-18 |
| SmkCes | rs1009181 | Monocyte count | 34469753 | chr6:26158993 | C=0.335, T=0.665 | 0.621 | NA | NA | 2.00E-20 |
| SmkCes | rs1009181 | Neutrophil count | 34469753 | chr6:26158993 | C=0.335, T=0.665 | 0.621 | NA | NA | 9.00E-12 |
| SmkCes | rs7807019 | Lifetime smoking index | 31689377 | chr7:117543063 | A=0.529, G=0.471 | 0.54 | 0.015 | 0.011-0.019 | 7.00E-14 |
| SmkCes | rs7807019 | Insomnia | 35835914 | chr7:117543063 | A=0.529, G=0.471 | NR | 0.005 | 0.003-0.007 | 7.00E-09 |
| SmkCes | rs215600 | Cognitive ability | 29186694 | chr7:32333642 | A=0.669, G=0.331 | NR | 6.289 | NA | 3.00E-10 |
| SmkCes | rs215600 | Cognitive ability (MTAG) | 29186694 | chr7:32333642 | A=0.669, G=0.331 | NR | 6.999 | NA | 3.00E-12 |
| SmkCes | rs215600 | Cigarettes smoked per day (MTAG) | 30643251 | chr7:32333642 | A=0.669, G=0.331 | 0.645 | 0.0243837 | 0.02-0.029 | 8.00E-27 |
| SmkCes | rs215600 | Smoking behaviour (cigarettes smoked per day) | 30643251 | chr7:32333642 | A=0.669, G=0.331 | 0.645 | 0.024002468 | 0.019-0.029 | 4.00E-21 |
| SmkCes | rs215600 | Cigarettes smoked per day | 36477530 | chr7:32333642 | A=0.669, G=0.331 | 0.644 | 0.0214 | 0.018-0.025 | 5.00E-30 |
| SmkCes | rs215600 | Problematic tobacco use | 37250466 | chr7:32333642 | A=0.669, G=0.331 | NR | NA | NA | 2.00E-18 |
| SmkCes | rs215600 | Cigarettes smoked per day (MTAG) | 36376304 | chr7:32333642 | A=0.669, G=0.331 | NR | 0.02425 | 0.019-0.029 | 2.00E-21 |
| SmkCes | rs60749569 | Smoking cessation (MTAG) | 30643251 | chr8:42602668 | A=0.918, T=0.082 | 0.0796 | 0.0208199 | 0.015-0.027 | 8.00E-13 |
| SmkCes | rs60749569 | Smoking cessation | 30643251 | chr8:42602668 | A=0.918, T=0.082 | 0.0796 | 0.04007128 | 0.026-0.054 | 3.00E-08 |
| SmkCes | rs11991338 | Smoking cessation | 36477530 | chr8:9288464 | A=0.171, G=0.829 | 0.171 | 0.0129 | 0.0094-0.0164 | 6.00E-13 |
| SmkCes | rs113382419 | Smoking cessation | 33082346 | chr9:136463019 | A=0.09, C=0.91 | NR | 0.922 | NA | 1.00E-27 |
| SmkCes | rs113382419 | Lifetime smoking index | 31689377 | chr9:136463019 | A=0.09, C=0.91 | 0.889 | 0.041 | 0.035-0.047 | 3.00E-37 |
| DrnkWk | rs13024996 | Problematic alcohol use (MTAG) | 32451486 | chr2:144225215 | A=0.359, C=0.641 | 0.3676 | 0.015542448 | 0.011-0.02 | 3.00E-13 |
| DrnkWk | rs13024996 | Alcohol consumption (drinks per week) (MTAG) | 32451486 | chr2:144225215 | A=0.359, C=0.641 | 0.352 | 0.013351299 | 0.0098-0.0169 | 1.00E-13 |
| DrnkWk | rs13024996 | Alcohol consumption | 31358974 | chr2:144225215 | A=0.359, C=0.641 | 0.37 | 0.008 | 0.006-0.01 | 4.00E-13 |
| DrnkWk | rs13024996 | Alcohol consumption (drinks per week) | 30643251 | chr2:144225215 | A=0.359, C=0.641 | 0.364 | 0.010912645 | 0.0079-0.0139 | 6.00E-13 |
| DrnkWk | rs13024996 | Alcohol consumption (drinks per week) (MTAG) | 30643251 | chr2:144225215 | A=0.359, C=0.641 | 0.364 | 0.010881 | 0.0082-0.0136 | 2.00E-15 |
| DrnkWk | rs13024996 | Maximum habitual alcohol consumption (MTAG) | 36301540 | chr2:144225215 | A=0.359, C=0.641 | NR | 0.014123027 | 0.0093-0.0189 | 9.00E-09 |
| DrnkWk | rs13024996 | Problematic alcohol use (MTAG) | 36301540 | chr2:144225215 | A=0.359, C=0.641 | NR | 0.0138 | 0.0093-0.0183 | 1.00E-09 |
| DrnkWk | rs13024996 | Drinks per week | 36477530 | chr2:144225215 | A=0.359, C=0.641 | 0.362 | 0.0105 | 0.0086-0.0124 | 9.00E-29 |
| DrnkWk | rs13024996 | Drinks per week | 36477530 | chr2:144225215 | A=0.359, C=0.641 | 0.347 | 0.00951 | 0.0078-0.0112 | 2.00E-27 |
| DrnkWk | rs13024996 | Alcohol use disorder (MTAG) | 37156939 | chr2:144225215 | A=0.359, C=0.641 | NR | 0.017337 | 0.012-0.023 | 3.00E-09 |
| DrnkWk | rs1260326 | Serum albumin levels | 29403010 | chr2:27730940 | C=0.589, T=0.411 | NR | 0.05949 | 0.051-0.068 | 1.00E-40 |
| DrnkWk | rs1260326 | Serum total protein levels | 29403010 | chr2:27730940 | C=0.589, T=0.411 | NR | 0.03993 | 0.032-0.048 | 4.00E-21 |
| DrnkWk | rs1260326 | Blood sugar levels | 29403010 | chr2:27730940 | C=0.589, T=0.411 | NR | 0.03854 | 0.029-0.048 | 2.00E-16 |
| DrnkWk | rs1260326 | Gamma glutamyl transferase levels | 29403010 | chr2:27730940 | C=0.589, T=0.411 | NR | 0.06015 | 0.052-0.068 | 1.00E-48 |
| DrnkWk | rs1260326 | Platelet count | 29403010 | chr2:27730940 | C=0.589, T=0.411 | NR | 0.04103 | 0.033-0.049 | 6.00E-22 |
| DrnkWk | rs1260326 | Triglyceride levels | 29403010 | chr2:27730940 | C=0.589, T=0.411 | NR | 0.08881 | 0.08-0.097 | 2.00E-94 |
| DrnkWk | rs1260326 | Total cholesterol levels | 29403010 | chr2:27730940 | C=0.589, T=0.411 | NR | 0.03525 | 0.028-0.043 | 3.00E-19 |
| DrnkWk | rs1260326 | White blood cell count | 29403010 | chr2:27730940 | C=0.589, T=0.411 | NR | 0.0256 | 0.017-0.034 | 2.00E-09 |
| DrnkWk | rs1260326 | Serum uric acid levels | 29403010 | chr2:27730940 | C=0.589, T=0.411 | NR | 0.03472 | 0.026-0.043 | 3.00E-16 |
| DrnkWk | rs1260326 | Glomerular filtration rate | 29403010 | chr2:27730940 | C=0.589, T=0.411 | NR | 0.03167 | 0.024-0.039 | 4.00E-17 |
| DrnkWk | rs1260326 | Estimated glomerular filtration rate | 30604766 | chr2:27730940 | C=0.589, T=0.411 | 0.534 | 0.675 | 0.49-0.86//1.732 | 2.00E-35 |
| DrnkWk | rs1260326 | Alcohol consumption (drinks per week) | 30679032 | chr2:27730940 | C=0.589, T=0.411 | 0.608 | 0.0321 | 0.027-0.037 | 5.00E-40 |
| DrnkWk | rs1260326 | Estimated glomerular filtration rate in non-diabetics | 31451708 | chr2:27730940 | C=0.589, T=0.411 | 0.391 | 0.449 | 0.35-0.55 | 4.00E-17 |
| DrnkWk | rs1260326 | Estimated glomerular filtration rate | 31451708 | chr2:27730940 | C=0.589, T=0.411 | 0.3906 | 0.0046 | 0.0038-0.0054 | 3.00E-36 |
| DrnkWk | rs1260326 | Estimated glomerular filtration rate | 31451708 | chr2:27730940 | C=0.589, T=0.411 | 0.422 | 0.0045 | 0.0039-0.0051 | 1.00E-34 |
| DrnkWk | rs1260326 | Metabolic traits | 19060910 | chr2:27730940 | C=0.589, T=0.411 | 0.35 | 0.09 | 0.06-0.12/ | 4.00E-10 |
| DrnkWk | rs1260326 | Lipoprotein-associated phospholipase A2 activity and mass | 23118302 | chr2:27730940 | C=0.589, T=0.411 | 0.44 | 0.0053 | -0.00215-0.01275/ | 5.00E-06 |
| DrnkWk | rs1260326 | Waist circumference and related phenotypes | 18454146 | chr2:27730940 | C=0.589, T=0.411 | NR | NA | NA | 4.00E-08 |
| DrnkWk | rs1260326 | Two-hour glucose challenge | 20081857 | chr2:27730940 | C=0.589, T=0.411 | NR | 0.07 | 0.05-0.09/ | 3.00E-10 |
| DrnkWk | rs1260326 | Metabolite levels | 22286219 | chr2:27730940 | C=0.589, T=0.411 | NR | 0.15 | 0.11-0.19 | 3.00E-18 |
| DrnkWk | rs1260326 | Cardiovascular disease risk factors | 21943158 | chr2:27730940 | C=0.589, T=0.411 | 0.4 | 0.082 | 0.053-0.111/ | 2.00E-08 |
| DrnkWk | rs1260326 | Triglycerides | 20139978 | chr2:27730940 | C=0.589, T=0.411 | 0.45 | 0.101 | 0.072-0.13 | 1.00E-11 |
| DrnkWk | rs1260326 | Hematological and biochemical traits | 20139978 | chr2:27730940 | C=0.589, T=0.411 | 0.44 | 0.085 | 0.056-0.114 | 4.00E-09 |
| DrnkWk | rs1260326 | Non-albumin protein levels | 22558069 | chr2:27730940 | C=0.589, T=0.411 | 0.445 | 0.08 | 0.053-0.107 | 3.00E-09 |
| DrnkWk | rs1260326 | Liver enzyme levels (gamma-glutamyl transferase) | 22001757 | chr2:27730940 | C=0.589, T=0.411 | 0.38 | 3.2 | 2.40-4.0% | 4.00E-13 |
| DrnkWk | rs1260326 | Urate levels | 23263486 | chr2:27730940 | C=0.589, T=0.411 | 0.41 | 0.074 | 0.063-0.084/ | 1.00E-44 |
| DrnkWk | rs1260326 | Cholesterol, total | 20686565 | chr2:27730940 | C=0.589, T=0.411 | 0.41 | 1.91 | 1.54-2.28/ | 7.00E-27 |
| DrnkWk | rs1260326 | Triglycerides | 20686565 | chr2:27730940 | C=0.589, T=0.411 | 0.41 | 8.76 | 7.98-9.54/ | 6.00E-133 |
| DrnkWk | rs1260326 | Triglycerides | 19060906 | chr2:27730940 | C=0.589, T=0.411 | 0.45 | 0.12 | 0.08-0.16.. | 2.00E-31 |
| DrnkWk | rs1260326 | Chronic kidney disease | 20383146 | chr2:27730940 | C=0.589, T=0.411 | 0.41 | 0.01 | 0.007-0.011 | 3.00E-14 |
| DrnkWk | rs1260326 | C-reactive protein levels | 21300955 | chr2:27730940 | C=0.589, T=0.411 | NR | 0.072 | 0.06-0.08 | 5.00E-40 |
| DrnkWk | rs1260326 | Hypertriglyceridemia | 23505323 | chr2:27730940 | C=0.589, T=0.411 | 0.26 | 1.41 | 1.31-1.51 | 2.00E-13 |
| DrnkWk | rs1260326 | Platelet count | 22139419 | chr2:27730940 | C=0.589, T=0.411 | NR | 2.334 | 1.59-3.0810^9/ | 9.00E-10 |
| DrnkWk | rs1260326 | High light scatter reticulocyte count | 32888494 | chr2:27730940 | C=0.589, T=0.411 | 0.605432 | 0.034849964 | 0.03-0.039 | 6.00E-53 |
| DrnkWk | rs1260326 | High light scatter reticulocyte percentage of red cells | 32888494 | chr2:27730940 | C=0.589, T=0.411 | 0.60543 | 0.037775226 | 0.033-0.042 | 5.00E-62 |
| DrnkWk | rs1260326 | Total triglycerides levels | 26690388 | chr2:27730940 | C=0.589, T=0.411 | 0.491 | 0.127 | 0.1-0.15 | 7.00E-24 |
| DrnkWk | rs1260326 | Type 2 diabetes | 30595370 | chr2:27730940 | C=0.589, T=0.411 | NR | NA | NA | 1.00E-15 |
| DrnkWk | rs1260326 | Neutrophil count | 32888493 | chr2:27730940 | C=0.589, T=0.411 | 0.588603 | NA | NA | 2.00E-62 |
| DrnkWk | rs1260326 | Problematic alcohol use (MTAG) | 32451486 | chr2:27730940 | C=0.589, T=0.411 | 0.5967 | 0.028574824 | 0.024-0.033 | 2.00E-42 |
| DrnkWk | rs1260326 | Alcohol consumption (drinks per week) (MTAG) | 32451486 | chr2:27730940 | C=0.589, T=0.411 | 0.595 | 0.024939064 | 0.021-0.028 | 1.00E-45 |
| DrnkWk | rs1260326 | Problematic alcohol use | 32451486 | chr2:27730940 | C=0.589, T=0.411 | 0.4033 | 9.296 | NA | 1.00E-20 |
| DrnkWk | rs1260326 | Mean platelet volume | 32888494 | chr2:27730940 | C=0.589, T=0.411 | 0.605205 | 0.016506068 | 0.012-0.021 | 5.00E-13 |
| DrnkWk | rs1260326 | Platelet count | 32888493 | chr2:27730940 | C=0.589, T=0.411 | 0.556325 | 0.036668 | 0.029-0.044 | 1.00E-22 |
| DrnkWk | rs1260326 | Platelet count | 32888493 | chr2:27730940 | C=0.589, T=0.411 | 0.575648 | NA | NA | 3.00E-118 |
| DrnkWk | rs1260326 | Caffeine consumption from coffee or tea | 33287642 | chr2:27730940 | C=0.589, T=0.411 | 0.391852 | 0.02297 | 0.018-0.028 | 4.00E-22 |
| DrnkWk | rs1260326 | Platelet count | 32888493 | chr2:27730940 | C=0.589, T=0.411 | 0.600973 | 0.039291 | 0.036-0.043 | 1.00E-98 |
| DrnkWk | rs1260326 | Liver fat content (MRI proton density fat fraction measure) | 32247823 | chr2:27730940 | C=0.589, T=0.411 | 0.39117 | 0.046 | 0.028-0.064 | 4.00E-08 |
| DrnkWk | rs1260326 | Monocyte percentage of white cells | 32888494 | chr2:27730940 | C=0.589, T=0.411 | 0.60558 | 0.04200302 | 0.038-0.046 | 3.00E-77 |
| DrnkWk | rs1260326 | Platelet count | 27863252 | chr2:27730940 | C=0.589, T=0.411 | 0.605 | 0.03860428 | 0.031-0.046 | 2.00E-25 |
| DrnkWk | rs1260326 | Hematocrit | 27863252 | chr2:27730940 | C=0.589, T=0.411 | 0.6053 | 0.02209369 | 0.015-0.029 | 8.00E-10 |
| DrnkWk | rs1260326 | Plasma lactate levels | 26433129 | chr2:27730940 | C=0.589, T=0.411 | 0.41 | 0.08 | 0.068-0.092 | 2.00E-47 |
| DrnkWk | rs1260326 | Plasma lactate levels | 26433129 | chr2:27730940 | C=0.589, T=0.411 | 0.41 | 0.08 | NA | 4.00E-52 |
| DrnkWk | rs1260326 | Inflammatory bowel disease | 26192919 | chr2:27730940 | C=0.589, T=0.411 | 0.4059 | 1.080162 | 1.06-1.1 | 1.00E-14 |
| DrnkWk | rs1260326 | Glomerular filtration rate (creatinine) | 26831199 | chr2:27730940 | C=0.589, T=0.411 | 0.42 | 0.0068 | 0.005-0.0086 | 3.00E-14 |
| DrnkWk | rs1260326 | Glomerular filtration rate in non diabetics (creatinine) | 26831199 | chr2:27730940 | C=0.589, T=0.411 | NR | 0.0065 | 0.0047-0.0083 | 2.00E-12 |
| DrnkWk | rs1260326 | Blood metabolite levels | 25898920 | chr2:27730940 | C=0.589, T=0.411 | NR | NA | NA | 6.00E-56 |
| DrnkWk | rs1260326 | Gout | 27899376 | chr2:27730940 | C=0.589, T=0.411 | 0.535 | 1.31 | 1.21-1.42 | 7.00E-11 |
| DrnkWk | rs1260326 | Renal overload gout | 27899376 | chr2:27730940 | C=0.589, T=0.411 | 0.535 | 1.35 | 1.22-1.49 | 5.00E-09 |
| DrnkWk | rs1260326 | Plateletcrit | 27863252 | chr2:27730940 | C=0.589, T=0.411 | 0.6049 | 0.03560478 | 0.028-0.043 | 1.00E-21 |
| DrnkWk | rs1260326 | C-reactive protein levels or triglyceride levels (pleiotropy) | 27286809 | chr2:27730940 | C=0.589, T=0.411 | NR | NA | NA | 4.00E-151 |
| DrnkWk | rs1260326 | C-reactive protein levels or total cholesterol levels (pleiotropy) | 27286809 | chr2:27730940 | C=0.589, T=0.411 | NR | NA | NA | 3.00E-63 |
| DrnkWk | rs1260326 | Caffeine metabolism (plasma 1,7-dimethylxanthine (paraxanthine) to 1,3,7-trimethylxanthine (caffeine) ratio) | 27702941 | chr2:27730940 | C=0.589, T=0.411 | 0.38 | 4.69 | - | 3.00E-06 |
| DrnkWk | rs1260326 | Chronic inflammatory diseases (ankylosing spondylitis, Crohn's disease, psoriasis, primary sclerosing cholangitis, ulcerative colitis) (pleiotropy) | 26974007 | chr2:27730940 | C=0.589, T=0.411 | NR | NA | NA | 2.00E-23 |
| DrnkWk | rs1260326 | White blood cell count | 27863252 | chr2:27730940 | C=0.589, T=0.411 | 0.6053 | 0.03410984 | 0.027-0.041 | 9.00E-21 |
| DrnkWk | rs1260326 | Type 2 diabetes | 32541925 | chr2:27730940 | C=0.589, T=0.411 | 0.5805 | 0.0625 | 0.055-0.07 | 2.00E-57 |
| DrnkWk | rs1260326 | Neutrophil percentage of white cells | 32888494 | chr2:27730940 | C=0.589, T=0.411 | 0.605525 | 0.019570084 | 0.015-0.024 | 4.00E-18 |
| DrnkWk | rs1260326 | Mean spheric corpuscular volume | 32888494 | chr2:27730940 | C=0.589, T=0.411 | 0.605401 | 0.028584257 | 0.024-0.033 | 3.00E-36 |
| DrnkWk | rs1260326 | Gestational insulin sensitivity | 32556615 | chr2:27730940 | C=0.589, T=0.411 | NR | 0.20038068 | 0.14-0.26 | 5.00E-12 |
| DrnkWk | rs1260326 | Neutrophil count | 32888494 | chr2:27730940 | C=0.589, T=0.411 | 0.605551 | 0.0363056 | 0.032-0.041 | 1.00E-57 |
| DrnkWk | rs1260326 | Type 2 diabetes | 32514122 | chr2:27730940 | C=0.589, T=0.411 | 0.437337429 | 1.0786191 | 1.061192016-1.096332338 | 9.00E-20 |
| DrnkWk | rs1260326 | White blood cell count | 32888493 | chr2:27730940 | C=0.589, T=0.411 | 0.575362 | NA | NA | 4.00E-78 |
| DrnkWk | rs1260326 | Serum metabolite levels | 33031748 | chr2:27730940 | C=0.589, T=0.411 | 0.339531 | 0.194 | 0.15-0.24 | 5.00E-16 |
| DrnkWk | rs1260326 | Serum metabolite levels | 33031748 | chr2:27730940 | C=0.589, T=0.411 | 0.339531 | 0.174 | 0.13-0.22 | 8.00E-13 |
| DrnkWk | rs1260326 | Serum metabolite levels | 33031748 | chr2:27730940 | C=0.589, T=0.411 | 0.339531 | 0.159 | 0.11-0.21 | 5.00E-11 |
| DrnkWk | rs1260326 | Serum metabolite levels | 33031748 | chr2:27730940 | C=0.589, T=0.411 | 0.339531 | 0.484 | 0.44-0.53 | 4.00E-89 |
| DrnkWk | rs1260326 | Plateletcrit | 32888494 | chr2:27730940 | C=0.589, T=0.411 | 0.60514 | 0.03774126 | 0.033-0.042 | 2.00E-60 |
| DrnkWk | rs1260326 | Type 2 diabetes | 32541925 | chr2:27730940 | C=0.589, T=0.411 | 0.5946 | 0.0638 | 0.055-0.073 | 3.00E-42 |
| DrnkWk | rs1260326 | Iron status biomarkers (ferritin levels) | 33536631 | chr2:27730940 | C=0.589, T=0.411 | 0.368 | 0.025 | 0.018-0.032 | 1.00E-12 |
| DrnkWk | rs1260326 | Lymphocyte count | 32888494 | chr2:27730940 | C=0.589, T=0.411 | 0.605461 | 0.022390792 | 0.018-0.027 | 3.00E-23 |
| DrnkWk | rs1260326 | Mean platelet volume | 32888493 | chr2:27730940 | C=0.589, T=0.411 | 0.395676 | 0.015312 | 0.011-0.019 | 5.00E-15 |
| DrnkWk | rs1260326 | Hematocrit | 32888494 | chr2:27730940 | C=0.589, T=0.411 | 0.60548 | 0.016021622 | 0.011-0.021 | 1.00E-11 |
| DrnkWk | rs1260326 | Neutrophil count | 32888493 | chr2:27730940 | C=0.589, T=0.411 | 0.602032 | 0.032512 | 0.029-0.036 | 7.00E-62 |
| DrnkWk | rs1260326 | Type 2 diabetes | 32499647 | chr2:27730940 | C=0.589, T=0.411 | 0.456 | 1.07 | 1.05-1.08 | 1.00E-21 |
| DrnkWk | rs1260326 | Lymphocyte count | 32888493 | chr2:27730940 | C=0.589, T=0.411 | 0.602112 | 0.02397 | 0.02-0.028 | 1.00E-35 |
| DrnkWk | rs1260326 | Lymphocyte count | 32888493 | chr2:27730940 | C=0.589, T=0.411 | 0.586366 | NA | NA | 3.00E-41 |
| DrnkWk | rs1260326 | circulating leptin levels | 32917775 | chr2:27730940 | C=0.589, T=0.411 | 0.606 | 0.04 | 0.022-0.058 | 6.00E-06 |
| DrnkWk | rs1260326 | circulating leptin levels | 32917775 | chr2:27730940 | C=0.589, T=0.411 | 0.624 | 0.043 | 0.027-0.059 | 2.00E-07 |
| DrnkWk | rs1260326 | circulating leptin levels adjusted for BMI | 32917775 | chr2:27730940 | C=0.589, T=0.411 | 0.624 | 0.043 | 0.023-0.063 | 8.00E-06 |
| DrnkWk | rs1260326 | circulating leptin levels adjusted for BMI | 32917775 | chr2:27730940 | C=0.589, T=0.411 | 0.624 | 0.059 | 0.043-0.075 | 6.00E-13 |
| DrnkWk | rs1260326 | circulating leptin levels adjusted for BMI | 32917775 | chr2:27730940 | C=0.589, T=0.411 | 0.607 | 0.048 | 0.034-0.062 | 4.00E-13 |
| DrnkWk | rs1260326 | circulating leptin levels adjusted for BMI | 32917775 | chr2:27730940 | C=0.589, T=0.411 | 0.606 | 0.057 | 0.039-0.075 | 9.00E-11 |
| DrnkWk | rs1260326 | circulating leptin levels adjusted for BMI | 32917775 | chr2:27730940 | C=0.589, T=0.411 | 0.624 | 0.057 | 0.039-0.075 | 2.00E-10 |
| DrnkWk | rs1260326 | circulating leptin levels adjusted for BMI | 32917775 | chr2:27730940 | C=0.589, T=0.411 | 0.624 | 0.068 | 0.044-0.092 | 4.00E-09 |
| DrnkWk | rs1260326 | circulating leptin levels adjusted for BMI | 32917775 | chr2:27730940 | C=0.589, T=0.411 | 0.607 | 0.053 | 0.035-0.071 | 2.00E-08 |
| DrnkWk | rs1260326 | circulating leptin levels adjusted for BMI | 32917775 | chr2:27730940 | C=0.589, T=0.411 | 0.606 | 0.062 | 0.037-0.087 | 7.00E-07 |
| DrnkWk | rs1260326 | White blood cell count | 32888493 | chr2:27730940 | C=0.589, T=0.411 | 0.601256 | 0.032863 | 0.029-0.037 | 1.00E-68 |
| DrnkWk | rs1260326 | Serum metabolite levels | 33031748 | chr2:27730940 | C=0.589, T=0.411 | 0.339531 | 0.174 | 0.13-0.22 | 8.00E-13 |
| DrnkWk | rs1260326 | Serum metabolite levels | 33031748 | chr2:27730940 | C=0.589, T=0.411 | 0.339531 | 0.194 | 0.15-0.24 | 5.00E-16 |
| DrnkWk | rs1260326 | Red cell distribution width | 32888493 | chr2:27730940 | C=0.589, T=0.411 | 0.391091 | NA | NA | 2.00E-34 |
| DrnkWk | rs1260326 | Mean reticulocyte volume | 32888494 | chr2:27730940 | C=0.589, T=0.411 | 0.605381 | 0.02733463 | 0.023-0.032 | 7.00E-33 |
| DrnkWk | rs1260326 | circulating leptin levels | 32917775 | chr2:27730940 | C=0.589, T=0.411 | 0.624 | 0.035 | 0.023-0.047 | 5.00E-08 |
| DrnkWk | rs1260326 | circulating leptin levels | 32917775 | chr2:27730940 | C=0.589, T=0.411 | 0.624 | 0.046 | 0.028-0.064 | 2.00E-07 |
| DrnkWk | rs1260326 | circulating leptin levels | 32917775 | chr2:27730940 | C=0.589, T=0.411 | 0.624 | 0.059 | 0.035-0.083 | 4.00E-07 |
| DrnkWk | rs1260326 | circulating leptin levels | 32917775 | chr2:27730940 | C=0.589, T=0.411 | 0.607 | 0.032 | 0.018-0.046 | 2.00E-06 |
| DrnkWk | rs1260326 | circulating leptin levels adjusted for BMI | 32917775 | chr2:27730940 | C=0.589, T=0.411 | 0.624 | 0.05 | 0.038-0.062 | 3.00E-15 |
| DrnkWk | rs1260326 | Nonalcoholic steatohepatitis | 32298765 | chr2:27730940 | C=0.589, T=0.411 | NR | 1.302 | 1.176-1.442 | 4.00E-07 |
| DrnkWk | rs1260326 | Platelet count | 32888494 | chr2:27730940 | C=0.589, T=0.411 | 0.605168 | 0.041072395 | 0.037-0.046 | 2.00E-71 |
| DrnkWk | rs1260326 | Serum alkaline phosphatase levels | 33547301 | chr2:27730940 | C=0.589, T=0.411 | 0.39 | 18.9 | - | 1.00E-79 |
| DrnkWk | rs1260326 | Factor VII activity or levels | 30642921 | chr2:27730940 | C=0.589, T=0.411 | 0.42 | 0.024 | NA | 8.00E-34 |
| DrnkWk | rs1260326 | Factor VII activity | 30642921 | chr2:27730940 | C=0.589, T=0.411 | 0.39 | 0.024 | 0.02-0.028 | 2.00E-30 |
| DrnkWk | rs1260326 | Factor VII activity | 30642921 | chr2:27730940 | C=0.589, T=0.411 | 0.41 | 0.025 | NA | 2.00E-28 |
| DrnkWk | rs1260326 | Alcohol consumption | 31358974 | chr2:27730940 | C=0.589, T=0.411 | 0.3939 | 0.0173 | 0.015-0.019 | 2.00E-60 |
| DrnkWk | rs1260326 | Red cell distribution width | 32888494 | chr2:27730940 | C=0.589, T=0.411 | 0.605449 | 0.026309544 | 0.022-0.031 | 3.00E-31 |
| DrnkWk | rs1260326 | Apolipoprotein A1 levels | 32203549 | chr2:27730940 | C=0.589, T=0.411 | 0.395328 | 0.025843 | 0.022-0.03 | 4.00E-38 |
| DrnkWk | rs1260326 | Type 2 diabetes | 30297969 | chr2:27730940 | C=0.589, T=0.411 | 0.6069 | 1.07 | 1.06-1.08 | 7.00E-25 |
| DrnkWk | rs1260326 | Apolipoprotein B levels | 32203549 | chr2:27730940 | C=0.589, T=0.411 | 0.395476 | 0.0495629 | 0.045-0.054 | 3.00E-121 |
| DrnkWk | rs1260326 | Non-oily fish consumption | 32066663 | chr2:27730940 | C=0.589, T=0.411 | NR | 0.0143394 | 0.0095-0.0192 | 9.00E-09 |
| DrnkWk | rs1260326 | Triglycerides x physical activity interaction (2df test) | 30670697 | chr2:27730940 | C=0.589, T=0.411 | 0.3642 | NA | NA | 3.00E-259 |
| DrnkWk | rs1260326 | Blood protein levels in cardiovascular risk | 28369058 | chr2:27730940 | C=0.589, T=0.411 | 0.44 | 0.14 | NA | 2.00E-18 |
| DrnkWk | rs1260326 | Appendicular lean mass | 31761296 | chr2:27730940 | C=0.589, T=0.411 | 0.605242 | 0.0720571 | 0.053-0.092 | 2.00E-13 |
| DrnkWk | rs1260326 | Serum metabolite levels (CMS) | 31636271 | chr2:27730940 | C=0.589, T=0.411 | NR | 0.105504915 | NA | 8.00E-20 |
| DrnkWk | rs1260326 | Serum metabolite levels (CMS) | 31636271 | chr2:27730940 | C=0.589, T=0.411 | NR | 0.08611423 | NA | 5.00E-14 |
| DrnkWk | rs1260326 | Serum metabolite levels (CMS) | 31636271 | chr2:27730940 | C=0.589, T=0.411 | NR | 0.08934505 | NA | 3.00E-15 |
| DrnkWk | rs1260326 | Serum metabolite levels (CMS) | 31636271 | chr2:27730940 | C=0.589, T=0.411 | NR | 0.091140695 | NA | 3.00E-14 |
| DrnkWk | rs1260326 | Serum metabolite levels (CMS) | 31636271 | chr2:27730940 | C=0.589, T=0.411 | NR | 0.08879713 | NA | 5.00E-13 |
| DrnkWk | rs1260326 | Appendicular lean mass | 31761296 | chr2:27730940 | C=0.589, T=0.411 | 0.605637 | 0.0615595 | 0.048-0.075 | 1.00E-18 |
| DrnkWk | rs1260326 | Serum metabolite levels (CMS) | 31636271 | chr2:27730940 | C=0.589, T=0.411 | NR | 0.079594925 | NA | 8.00E-11 |
| DrnkWk | rs1260326 | Serum metabolite levels (CMS) | 31636271 | chr2:27730940 | C=0.589, T=0.411 | NR | 0.078505106 | NA | 2.00E-13 |
| DrnkWk | rs1260326 | Serum metabolite levels (CMS) | 31636271 | chr2:27730940 | C=0.589, T=0.411 | NR | 0.077943616 | NA | 1.00E-12 |
| DrnkWk | rs1260326 | Serum metabolite levels (CMS) | 31636271 | chr2:27730940 | C=0.589, T=0.411 | NR | 0.07405403 | NA | 2.00E-12 |
| DrnkWk | rs1260326 | Serum metabolite levels (CMS) | 31636271 | chr2:27730940 | C=0.589, T=0.411 | NR | 0.07906318 | NA | 1.00E-11 |
| DrnkWk | rs1260326 | Serum metabolite levels (CMS) | 31636271 | chr2:27730940 | C=0.589, T=0.411 | NR | 0.07566091 | NA | 1.00E-11 |
| DrnkWk | rs1260326 | Serum metabolite levels (CMS) | 31636271 | chr2:27730940 | C=0.589, T=0.411 | NR | 0.0800872 | NA | 5.00E-13 |
| DrnkWk | rs1260326 | Serum metabolite levels (CMS) | 31636271 | chr2:27730940 | C=0.589, T=0.411 | NR | 0.068720095 | NA | 5.00E-12 |
| DrnkWk | rs1260326 | Serum metabolite levels (CMS) | 31636271 | chr2:27730940 | C=0.589, T=0.411 | NR | 0.071902275 | NA | 1.00E-10 |
| DrnkWk | rs1260326 | Serum metabolite levels (CMS) | 31636271 | chr2:27730940 | C=0.589, T=0.411 | NR | 0.078515455 | NA | 5.00E-14 |
| DrnkWk | rs1260326 | Serum metabolite levels | 31636271 | chr2:27730940 | C=0.589, T=0.411 | NR | 0.09025589 | NA | 5.00E-13 |
| DrnkWk | rs1260326 | Serum metabolite levels | 31636271 | chr2:27730940 | C=0.589, T=0.411 | NR | 0.09012033 | NA | 6.00E-13 |
| DrnkWk | rs1260326 | Serum metabolite levels | 31636271 | chr2:27730940 | C=0.589, T=0.411 | NR | 0.089100525 | NA | 9.00E-13 |
| DrnkWk | rs1260326 | Serum metabolite levels | 31636271 | chr2:27730940 | C=0.589, T=0.411 | NR | 0.10920316 | NA | 7.00E-19 |
| DrnkWk | rs1260326 | Serum metabolite levels | 31636271 | chr2:27730940 | C=0.589, T=0.411 | NR | 0.08317139 | NA | 2.00E-11 |
| DrnkWk | rs1260326 | Serum metabolite levels | 31636271 | chr2:27730940 | C=0.589, T=0.411 | NR | 0.086691655 | NA | 4.00E-12 |
| DrnkWk | rs1260326 | Serum metabolite levels | 31636271 | chr2:27730940 | C=0.589, T=0.411 | NR | 0.08626581 | NA | 5.00E-12 |
| DrnkWk | rs1260326 | Serum metabolite levels | 31636271 | chr2:27730940 | C=0.589, T=0.411 | NR | 0.08549069 | NA | 7.00E-12 |
| DrnkWk | rs1260326 | Serum metabolite levels | 31636271 | chr2:27730940 | C=0.589, T=0.411 | NR | 0.08515495 | NA | 9.00E-12 |
| DrnkWk | rs1260326 | Serum metabolite levels | 31636271 | chr2:27730940 | C=0.589, T=0.411 | NR | 0.08497287 | NA | 1.00E-11 |
| DrnkWk | rs1260326 | Serum metabolite levels | 31636271 | chr2:27730940 | C=0.589, T=0.411 | NR | 0.071229525 | NA | 1.00E-08 |
| DrnkWk | rs1260326 | Serum metabolite levels | 31636271 | chr2:27730940 | C=0.589, T=0.411 | NR | 0.0786342 | NA | 3.00E-10 |
| DrnkWk | rs1260326 | Serum metabolite levels | 31636271 | chr2:27730940 | C=0.589, T=0.411 | NR | 0.0759521 | NA | 2.00E-09 |
| DrnkWk | rs1260326 | Serum metabolite levels | 31636271 | chr2:27730940 | C=0.589, T=0.411 | NR | 0.06593476 | NA | 1.00E-07 |
| DrnkWk | rs1260326 | Serum metabolite levels | 31636271 | chr2:27730940 | C=0.589, T=0.411 | NR | 0.07296435 | NA | 7.00E-07 |
| DrnkWk | rs1260326 | Triglyceride levels x short total sleep time interaction (2df test) | 31719535 | chr2:27730940 | C=0.589, T=0.411 | NR | 0.04459 | 0.04-0.049 | 3.00E-100 |
| DrnkWk | rs1260326 | C-reactive protein levels | 31900758 | chr2:27730940 | C=0.589, T=0.411 | 0.392838 | 0.0745916 | 0.071-0.079 | 2.00E-283 |
| DrnkWk | rs1260326 | Urate levels | 31985003 | chr2:27730940 | C=0.589, T=0.411 | NR | 0.073 | 0.063-0.083/ | 1.00E-46 |
| DrnkWk | rs1260326 | Alcohol consumption (heavy vs. light/non-drinkers) | 31998841 | chr2:27730940 | C=0.589, T=0.411 | 0.612 | 7.391 | NA | 1.00E-13 |
| DrnkWk | rs1260326 | Coffee consumption | 31959922 | chr2:27730940 | C=0.589, T=0.411 | NR | 0.096 | 0.074-0.118 | 1.00E-16 |
| DrnkWk | rs1260326 | Alcohol consumption (drinkers vs non-drinkers) | 31959922 | chr2:27730940 | C=0.589, T=0.411 | NR | 0.011 | 0.0081-0.0139 | 2.00E-13 |
| DrnkWk | rs1260326 | Metabolite levels | 23823483 | chr2:27730940 | C=0.589, T=0.411 | NR | 0.15429989 | 0.088-0.221 | 5.00E-06 |
| DrnkWk | rs1260326 | Metabolite levels | 23823483 | chr2:27730940 | C=0.589, T=0.411 | NR | 0.15190773 | 0.086-0.218 | 6.00E-06 |
| DrnkWk | rs1260326 | Metabolite levels | 23823483 | chr2:27730940 | C=0.589, T=0.411 | NR | 0.18466824 | 0.12-0.25 | 3.00E-08 |
| DrnkWk | rs1260326 | Metabolite levels | 23823483 | chr2:27730940 | C=0.589, T=0.411 | NR | 0.180729 | 0.12-0.25 | 7.00E-08 |
| DrnkWk | rs1260326 | Metabolite levels | 23823483 | chr2:27730940 | C=0.589, T=0.411 | NR | 0.16496815 | 0.099-0.231 | 9.00E-07 |
| DrnkWk | rs1260326 | Metabolite levels | 23823483 | chr2:27730940 | C=0.589, T=0.411 | NR | 0.1850016 | 0.12-0.25 | 4.00E-08 |
| DrnkWk | rs1260326 | Metabolite levels | 23823483 | chr2:27730940 | C=0.589, T=0.411 | NR | 0.15362816 | 0.087-0.22 | 6.00E-06 |
| DrnkWk | rs1260326 | Metabolite levels | 23823483 | chr2:27730940 | C=0.589, T=0.411 | NR | 0.15675637 | 0.091-0.222 | 3.00E-06 |
| DrnkWk | rs1260326 | Metabolite levels | 23823483 | chr2:27730940 | C=0.589, T=0.411 | NR | 0.17565545 | 0.11-0.24 | 2.00E-07 |
| DrnkWk | rs1260326 | Metabolite levels | 23823483 | chr2:27730940 | C=0.589, T=0.411 | NR | 0.1853844 | 0.12-0.25 | 3.00E-08 |
| DrnkWk | rs1260326 | Metabolite levels | 23823483 | chr2:27730940 | C=0.589, T=0.411 | NR | 0.19759764 | 0.13-0.26 | 3.00E-09 |
| DrnkWk | rs1260326 | Metabolite levels | 23823483 | chr2:27730940 | C=0.589, T=0.411 | NR | 0.15770823 | 0.092-0.224 | 3.00E-06 |
| DrnkWk | rs1260326 | Metabolite levels | 23823483 | chr2:27730940 | C=0.589, T=0.411 | NR | 0.24086063 | 0.17-0.31 | 3.00E-11 |
| DrnkWk | rs1260326 | Metabolite levels | 23823483 | chr2:27730940 | C=0.589, T=0.411 | NR | 0.16124451 | 0.095-0.227 | 2.00E-06 |
| DrnkWk | rs1260326 | Metabolite levels | 23823483 | chr2:27730940 | C=0.589, T=0.411 | NR | 0.15930784 | 0.094-0.225 | 2.00E-06 |
| DrnkWk | rs1260326 | Hypertriglyceridemia | 20657596 | chr2:27730940 | C=0.589, T=0.411 | 0.41 | 1.75 | 1.45-2.12 | 7.00E-09 |
| DrnkWk | rs1260326 | Metabolite levels | 23823483 | chr2:27730940 | C=0.589, T=0.411 | NR | 0.16869353 | 0.1-0.23 | 4.00E-07 |
| DrnkWk | rs1260326 | Metabolite levels | 23823483 | chr2:27730940 | C=0.589, T=0.411 | NR | 0.16491295 | 0.099-0.231 | 9.00E-07 |
| DrnkWk | rs1260326 | Metabolite levels | 23823483 | chr2:27730940 | C=0.589, T=0.411 | NR | 0.17947723 | 0.11-0.24 | 7.00E-08 |
| DrnkWk | rs1260326 | Metabolite levels | 23823483 | chr2:27730940 | C=0.589, T=0.411 | NR | 0.18267855 | 0.12-0.25 | 5.00E-08 |
| DrnkWk | rs1260326 | Metabolite levels | 23823483 | chr2:27730940 | C=0.589, T=0.411 | NR | 0.16283728 | 0.097-0.229 | 1.00E-06 |
| DrnkWk | rs1260326 | Metabolite levels | 23823483 | chr2:27730940 | C=0.589, T=0.411 | NR | 0.1859652 | 0.12-0.25 | 2.00E-08 |
| DrnkWk | rs1260326 | Metabolite levels | 23823483 | chr2:27730940 | C=0.589, T=0.411 | NR | 0.17034519 | 0.11-0.24 | 2.00E-07 |
| DrnkWk | rs1260326 | Metabolite levels | 23823483 | chr2:27730940 | C=0.589, T=0.411 | NR | 0.17764857 | 0.11-0.24 | 1.00E-07 |
| DrnkWk | rs1260326 | Metabolite levels | 23823483 | chr2:27730940 | C=0.589, T=0.411 | NR | 0.21938264 | 0.15-0.29 | 1.00E-09 |
| DrnkWk | rs1260326 | Metabolite levels | 23823483 | chr2:27730940 | C=0.589, T=0.411 | NR | 0.23077767 | 0.16-0.3 | 8.00E-12 |
| DrnkWk | rs1260326 | Metabolite levels | 23823483 | chr2:27730940 | C=0.589, T=0.411 | NR | 0.17035955 | 0.11-0.24 | 3.00E-07 |
| DrnkWk | rs1260326 | Metabolite levels | 23823483 | chr2:27730940 | C=0.589, T=0.411 | NR | 0.15454282 | 0.089-0.22 | 3.00E-06 |
| DrnkWk | rs1260326 | Cholesterol, total | 24097068 | chr2:27730940 | C=0.589, T=0.411 | 0.39 | 0.051 | NA | 3.00E-42 |
| DrnkWk | rs1260326 | Triglycerides | 24097068 | chr2:27730940 | C=0.589, T=0.411 | 0.39 | 0.115 | / | 2.00E-239 |
| DrnkWk | rs1260326 | Lipid traits | 24386095 | chr2:27730940 | C=0.589, T=0.411 | 0.44 | 0.07 | / | 3.00E-07 |
| DrnkWk | rs1260326 | Glycemic traits (pregnancy) | 23903356 | chr2:27730940 | C=0.589, T=0.411 | 0.409 | 0.0044 | NA | 6.00E-13 |
| DrnkWk | rs1260326 | Glycemic traits (pregnancy) | 23903356 | chr2:27730940 | C=0.589, T=0.411 | 0.409 | 0.0116 | NA | 6.00E-11 |
| DrnkWk | rs1260326 | Glomerular filtration rate | 27588450 | chr2:27730940 | C=0.589, T=0.411 | NR | 0.872 | 0.65-1.1 | 6.00E-14 |
| DrnkWk | rs1260326 | Glomerular filtration rate | 27588450 | chr2:27730940 | C=0.589, T=0.411 | 0.6 | 0.83 | 0.52-1.14 | 2.00E-07 |
| DrnkWk | rs1260326 | Total cholesterol levels | 29507422 | chr2:27730940 | C=0.589, T=0.411 | 0.422 | 0.073 | NA | 2.00E-56 |
| DrnkWk | rs1260326 | Total cholesterol levels | 29507422 | chr2:27730940 | C=0.589, T=0.411 | 0.368 | 0.096 | NA | 5.00E-10 |
| DrnkWk | rs1260326 | Total cholesterol levels | 29507422 | chr2:27730940 | C=0.589, T=0.411 | 0.478 | 0.084 | NA | 1.00E-07 |
| DrnkWk | rs1260326 | Total cholesterol levels | 29507422 | chr2:27730940 | C=0.589, T=0.411 | NR | 0.075 | NA | 3.00E-70 |
| DrnkWk | rs1260326 | Red cell distribution width | 30595370 | chr2:27730940 | C=0.589, T=0.411 | NR | NA | NA | 1.00E-25 |
| DrnkWk | rs1260326 | Low density lipoprotein cholesterol levels | 32154731 | chr2:27730940 | C=0.589, T=0.411 | NR | 0.0548 | 0.05-0.059 | 4.00E-99 |
| DrnkWk | rs1260326 | Triglyceride levels | 32154731 | chr2:27730940 | C=0.589, T=0.411 | NR | 0.1035 | 0.099-0.108 | 0 |
| DrnkWk | rs1260326 | Refractive error | 32231278 | chr2:27730940 | C=0.589, T=0.411 | NR | NA | NA | 5.00E-10 |
| DrnkWk | rs1260326 | Triglyceride levels | 31647587 | chr2:27730940 | C=0.589, T=0.411 | NR | NA | NA | 3.00E-07 |
| DrnkWk | rs1260326 | Triglyceride levels x long total sleep time interaction (2df test) | 31719535 | chr2:27730940 | C=0.589, T=0.411 | NR | 0.04586 | 0.042-0.05 | 5.00E-116 |
| DrnkWk | rs1260326 | Gout (combined type) | 32238385 | chr2:27730940 | C=0.589, T=0.411 | 0.545 | 1.37 | 1.23-1.53 | 1.00E-08 |
| DrnkWk | rs1260326 | Gout | 32238385 | chr2:27730940 | C=0.589, T=0.411 | 0.545 | 1.3 | 1.21-1.39 | 2.00E-13 |
| DrnkWk | rs1260326 | Serum metabolite levels (CMS) | 31636271 | chr2:27730940 | C=0.589, T=0.411 | NR | 0.05097968 | NA | 1.00E-09 |
| DrnkWk | rs1260326 | Serum metabolite levels (CMS) | 31636271 | chr2:27730940 | C=0.589, T=0.411 | NR | 0.049552824 | NA | 2.00E-10 |
| DrnkWk | rs1260326 | Serum metabolite levels (CMS) | 31636271 | chr2:27730940 | C=0.589, T=0.411 | NR | 0.04480671 | NA | 6.00E-10 |
| DrnkWk | rs1260326 | Serum metabolite levels (CMS) | 31636271 | chr2:27730940 | C=0.589, T=0.411 | NR | 0.07117757 | NA | 8.00E-11 |
| DrnkWk | rs1260326 | Serum metabolite levels | 31636271 | chr2:27730940 | C=0.589, T=0.411 | NR | 0.05836895 | NA | 3.00E-06 |
| DrnkWk | rs1260326 | Serum metabolite levels | 31636271 | chr2:27730940 | C=0.589, T=0.411 | NR | 0.091697775 | NA | 2.00E-13 |
| DrnkWk | rs1260326 | Nonalcoholic fatty liver disease | 34841290 | chr2:27730940 | C=0.589, T=0.411 | NR | 0.0755 | 0.043-0.108 | 6.00E-06 |
| DrnkWk | rs1260326 | White blood cell count | 30595370 | chr2:27730940 | C=0.589, T=0.411 | NR | NA | NA | 4.00E-59 |
| DrnkWk | rs1260326 | Serum alkaline phosphatase levels | 31666285 | chr2:27730940 | C=0.589, T=0.411 | 0.341 | 0.03 | 0.02-0.04 | 5.00E-11 |
| DrnkWk | rs1260326 | Gout | 25646370 | chr2:27730940 | C=0.589, T=0.411 | 0.55 | 1.36 | 1.25-1.48 | 2.00E-12 |
| DrnkWk | rs1260326 | Triglycerides | 25961943 | chr2:27730940 | C=0.589, T=0.411 | 0.36 | 0.123 | 0.11-0.13.. | 5.00E-88 |
| DrnkWk | rs1260326 | Cholesterol, total | 25961943 | chr2:27730940 | C=0.589, T=0.411 | 0.36 | 0.045 | 0.033-0.057.. | 3.00E-13 |
| DrnkWk | rs1260326 | Serum alpha1-antitrypsin levels | 26174136 | chr2:27730940 | C=0.589, T=0.411 | 0.432 | 2.05 | 1.56-2.54-1 | 3.00E-16 |
| DrnkWk | rs1260326 | Crohn's disease | 28067908 | chr2:27730940 | C=0.589, T=0.411 | NR | NA | NA | 6.00E-11 |
| DrnkWk | rs1260326 | Gallstone disease | 27094239 | chr2:27730940 | C=0.589, T=0.411 | 0.59 | 1.12 | 1.09–1.15 | 8.00E-08 |
| DrnkWk | rs1260326 | Inflammatory bowel disease | 28067908 | chr2:27730940 | C=0.589, T=0.411 | NR | NA | NA | 1.00E-07 |
| DrnkWk | rs1260326 | Triglyceride levels | 28334899 | chr2:27730940 | C=0.589, T=0.411 | 0.413 | 0.115 | 0.11-0.12() | 4.00E-253 |
| DrnkWk | rs1260326 | Protein C levels | 28082259 | chr2:27730940 | C=0.589, T=0.411 | 0.41 | 0.07 | μ/ | 1.00E-16 |
| DrnkWk | rs1260326 | Lymphocyte count | 27863252 | chr2:27730940 | C=0.589, T=0.411 | 0.6052 | 0.02574395 | 0.019-0.033 | 2.00E-12 |
| DrnkWk | rs1260326 | Serum total protein levels | 23022100 | chr2:27730940 | C=0.589, T=0.411 | 0.44 | 0.0179 | 0.011-0.024 | 6.00E-08 |
| DrnkWk | rs1260326 | Serum total protein levels | 23022100 | chr2:27730940 | C=0.589, T=0.411 | 0.56 | 0.031 | 0.017-0.045 | 4.00E-06 |
| DrnkWk | rs1260326 | Serum albumin levels | 23022100 | chr2:27730940 | C=0.589, T=0.411 | 0.41 | 0.0138 | 0.011-0.017 | 4.00E-19 |
| DrnkWk | rs1260326 | Serum albumin levels | 23022100 | chr2:27730940 | C=0.589, T=0.411 | 0.41 | 0.0124 | 0.0093-0.0155 | 3.00E-14 |
| DrnkWk | rs1260326 | Serum albumin levels | 23022100 | chr2:27730940 | C=0.589, T=0.411 | 0.56 | 0.027 | 0.017-0.037 | 2.00E-08 |
| DrnkWk | rs1260326 | High light scatter reticulocyte percentage of red cells | 27863252 | chr2:27730940 | C=0.589, T=0.411 | 0.6052 | 0.03532376 | 0.028-0.042() | 4.00E-22 |
| DrnkWk | rs1260326 | Granulocyte count | 27863252 | chr2:27730940 | C=0.589, T=0.411 | 0.6052 | 0.03150785 | 0.024-0.039 | 7.00E-18 |
| DrnkWk | rs1260326 | Sum neutrophil eosinophil counts | 27863252 | chr2:27730940 | C=0.589, T=0.411 | 0.6053 | 0.03202804 | 0.025-0.039 | 2.00E-18 |
| DrnkWk | rs1260326 | Sum basophil neutrophil counts | 27863252 | chr2:27730940 | C=0.589, T=0.411 | 0.6053 | 0.03251663 | 0.025-0.04 | 5.00E-19 |
| DrnkWk | rs1260326 | Red cell distribution width | 27863252 | chr2:27730940 | C=0.589, T=0.411 | 0.6052 | 0.02456709 | 0.018-0.032 | 9.00E-12 |
| DrnkWk | rs1260326 | Myeloid white cell count | 27863252 | chr2:27730940 | C=0.589, T=0.411 | 0.6053 | 0.0286908 | 0.022-0.036 | 5.00E-15 |
| DrnkWk | rs1260326 | Low density lipoprotein cholesterol levels | 29507422 | chr2:27730940 | C=0.589, T=0.411 | NR | 0.027 | NA | 7.00E-10 |
| DrnkWk | rs1260326 | Urate levels in overweight individuals | 25811787 | chr2:27730940 | C=0.589, T=0.411 | 0.58 | 0.055 | 0.033-0.077/2 | 8.00E-07 |
| DrnkWk | rs1260326 | Urate levels in overweight individuals | 25811787 | chr2:27730940 | C=0.589, T=0.411 | 0.58 | 0.065 | 0.038-0.092/2 | 6.00E-06 |
| DrnkWk | rs1260326 | Lipid metabolism phenotypes | 19936222 | chr2:27730940 | C=0.589, T=0.411 | NR | 0.075 | NA | 4.00E-32 |
| DrnkWk | rs1260326 | Lipid metabolism phenotypes | 19936222 | chr2:27730940 | C=0.589, T=0.411 | NR | 0.07 | NA | 1.00E-37 |
| DrnkWk | rs1260326 | Lipid metabolism phenotypes | 19936222 | chr2:27730940 | C=0.589, T=0.411 | NR | 0.052 | NA | 3.00E-35 |
| DrnkWk | rs1260326 | Lipid metabolism phenotypes | 19936222 | chr2:27730940 | C=0.589, T=0.411 | NR | 0.056 | NA | 3.00E-29 |
| DrnkWk | rs1260326 | Lipid metabolism phenotypes | 19936222 | chr2:27730940 | C=0.589, T=0.411 | NR | 0.362 | NA | 4.00E-24 |
| DrnkWk | rs1260326 | Lipid metabolism phenotypes | 19936222 | chr2:27730940 | C=0.589, T=0.411 | NR | 0.342 | NA | 3.00E-28 |
| DrnkWk | rs1260326 | Metabolite levels | 22916037 | chr2:27730940 | C=0.589, T=0.411 | 0.36 | NA | NA | 1.00E-12 |
| DrnkWk | rs1260326 | Coffee consumption (cups per day) | 25288136 | chr2:27730940 | C=0.589, T=0.411 | 0.41 | 0.04 | 0.020-0.060 | 7.00E-08 |
| DrnkWk | rs1260326 | Resting heart rate | 27798624 | chr2:27730940 | C=0.589, T=0.411 | 0.39 | 0.2746 | 0.21-0.34 | 4.00E-16 |
| DrnkWk | rs1260326 | Neutrophil count | 27863252 | chr2:27730940 | C=0.589, T=0.411 | 0.6053 | 0.03308063 | 0.026-0.04 | 1.00E-19 |
| DrnkWk | rs1260326 | Low density lipoprotein cholesterol levels | 29507422 | chr2:27730940 | C=0.589, T=0.411 | 0.422 | 0.026 | NA | 3.00E-08 |
| DrnkWk | rs1260326 | Triglycerides | 29507422 | chr2:27730940 | C=0.589, T=0.411 | 0.422 | 0.147 | NA | 2.00E-272 |
| DrnkWk | rs1260326 | Triglycerides | 29507422 | chr2:27730940 | C=0.589, T=0.411 | 0.368 | 0.15 | NA | 1.00E-26 |
| DrnkWk | rs1260326 | Triglycerides | 29507422 | chr2:27730940 | C=0.589, T=0.411 | 0.478 | 0.145 | NA | 1.00E-23 |
| DrnkWk | rs1260326 | Triglycerides | 29507422 | chr2:27730940 | C=0.589, T=0.411 | NR | 0.147 | NA | 1.00E-300 |
| DrnkWk | rs1260326 | Alcohol consumption | 28937693 | chr2:27730940 | C=0.589, T=0.411 | 0.39 | 0.028 | 0.022-0.034 | 1.00E-21 |
| DrnkWk | rs1260326 | Alcohol consumption in current drinkers | 28937693 | chr2:27730940 | C=0.589, T=0.411 | 0.38 | 0.03 | 0.024-0.036 | 7.00E-24 |
| DrnkWk | rs1260326 | Nonalcoholic fatty liver disease | 29385134 | chr2:27730940 | C=0.589, T=0.411 | 0.57 | 1.38 | 1.25-1.53 | 1.00E-09 |
| DrnkWk | rs1260326 | Macular telangiectasia type 2 | 33654266 | chr2:27730940 | C=0.589, T=0.411 | 0.589 | 0.742 | 0.66-0.83 | 1.00E-07 |
| DrnkWk | rs1260326 | Macular telangiectasia type 2 | 33654266 | chr2:27730940 | C=0.589, T=0.411 | 0.589 | NA | NA | 6.00E-07 |
| DrnkWk | rs1260326 | Follistatin levels | 33067605 | chr2:27730940 | C=0.589, T=0.411 | 0.4 | 0.1401 | NA | 3.00E-55 |
| DrnkWk | rs1260326 | Serum 25-Hydroxyvitamin D levels | 32242144 | chr2:27730940 | C=0.589, T=0.411 | 0.393435 | 0.022 | 0.018-0.026 | 4.00E-28 |
| DrnkWk | rs1260326 | Serum 25-Hydroxyvitamin D levels | 32242144 | chr2:27730940 | C=0.589, T=0.411 | NR | 0.0197194 | 0.016-0.024 | 2.00E-21 |
| DrnkWk | rs1260326 | Estimated glomerular filtration rate | 33418499 | chr2:27730940 | C=0.589, T=0.411 | NR | 0.4913817 | 0.32-0.66//1.732 | 3.00E-08 |
| DrnkWk | rs1260326 | Body mass index and LDL-C (pairwise) | 33619380 | chr2:27730940 | C=0.589, T=0.411 | 0.5895 | NA | NA | 5.00E-09 |
| DrnkWk | rs1260326 | Body mass index and triglycerides (pairwise) | 33619380 | chr2:27730940 | C=0.589, T=0.411 | 0.5895 | NA | NA | 2.00E-262 |
| DrnkWk | rs1260326 | Cathepsin D levels | 33067605 | chr2:27730940 | C=0.589, T=0.411 | 0.39 | 0.0598 | NA | 2.00E-13 |
| DrnkWk | rs1260326 | Chitinase-3-like protein 1 levels | 33067605 | chr2:27730940 | C=0.589, T=0.411 | 0.39 | 0.0673 | NA | 6.00E-17 |
| DrnkWk | rs1260326 | Gallstone disease | 30504769 | chr2:27730940 | C=0.589, T=0.411 | NR | 1.0752687 | 1.06-1.1 | 2.00E-16 |
| DrnkWk | rs1260326 | TGFBI/VASN protein level ratio | 38412862 | chr2:27730940 | C=0.589, T=0.411 | NR | 0.0960666 | 0.082-0.11 | 3.00E-42 |
| DrnkWk | rs1260326 | CLEC1A/GPR37 protein level ratio | 38412862 | chr2:27730940 | C=0.589, T=0.411 | NR | 0.103167 | 0.089-0.117 | 5.00E-49 |
| DrnkWk | rs1260326 | LAMP3/PRSS8 protein level ratio | 38412862 | chr2:27730940 | C=0.589, T=0.411 | NR | 0.0915576 | 0.078-0.105 | 2.00E-39 |
| DrnkWk | rs1260326 | LEPR/NRCAM protein level ratio | 38412862 | chr2:27730940 | C=0.589, T=0.411 | NR | 0.186847 | 0.17-0.2 | 1.00E-160 |
| DrnkWk | rs1260326 | LEPR/NTRK2 protein level ratio | 38412862 | chr2:27730940 | C=0.589, T=0.411 | NR | 0.192735 | 0.18-0.21 | 9.00E-168 |
| DrnkWk | rs1260326 | CTSD/PRCP protein level ratio | 38412862 | chr2:27730940 | C=0.589, T=0.411 | NR | 0.0934224 | 0.079-0.108 | 1.00E-37 |
| DrnkWk | rs1260326 | CST3/RARRES2 protein level ratio | 38412862 | chr2:27730940 | C=0.589, T=0.411 | NR | 0.0711327 | 0.058-0.085 | 2.00E-25 |
| DrnkWk | rs1260326 | CTSF/SMPD1 protein level ratio | 38412862 | chr2:27730940 | C=0.589, T=0.411 | NR | 0.136445 | 0.12-0.15 | 3.00E-91 |
| DrnkWk | rs1260326 | CANT1/ROR1 protein level ratio | 38412862 | chr2:27730940 | C=0.589, T=0.411 | NR | 0.0929605 | 0.079-0.106 | 2.00E-41 |
| DrnkWk | rs1260326 | FGF21/LEP protein level ratio | 38412862 | chr2:27730940 | C=0.589, T=0.411 | NR | 0.136132 | 0.12-0.15 | 1.00E-107 |
| DrnkWk | rs1260326 | FGFR2/TGFBR2 protein level ratio | 38412862 | chr2:27730940 | C=0.589, T=0.411 | NR | 0.0932761 | 0.08-0.107 | 1.00E-42 |
| DrnkWk | rs1260326 | Gout | 33832965 | chr2:27730940 | C=0.589, T=0.411 | 0.39 | 1.26 | 1.21-1.31 | 3.00E-31 |
| DrnkWk | rs1260326 | Gout vs asymptomatic hyperuricemia | 33832965 | chr2:27730940 | C=0.589, T=0.411 | 0.41 | 1.14 | 1.10-1.18 | 5.00E-12 |
| DrnkWk | rs1260326 | IGFBP1/IGFBP2 protein level ratio | 38412862 | chr2:27730940 | C=0.589, T=0.411 | NR | 0.132558 | 0.12-0.15 | 1.00E-83 |
| DrnkWk | rs1260326 | IL6ST/LEPR protein level ratio | 38412862 | chr2:27730940 | C=0.589, T=0.411 | NR | 0.182146 | 0.17-0.2 | 2.00E-154 |
| DrnkWk | rs1260326 | Appendicular lean mass | 33097823 | chr2:27730940 | C=0.589, T=0.411 | 0.396 | 0.03 | 0.024-0.036 | 4.00E-37 |
| DrnkWk | rs1260326 | Appendicular lean mass | 33097823 | chr2:27730940 | C=0.589, T=0.411 | 0.396 | 0.03 | 0.024-0.036 | 5.00E-29 |
| DrnkWk | rs1260326 | Total cholesterol levels | 33339817 | chr2:27730940 | C=0.589, T=0.411 | NR | 0.0366 | 0.03-0.043 | 2.00E-25 |
| DrnkWk | rs1260326 | Total testosterone levels | 32042192 | chr2:27730940 | C=0.589, T=0.411 | 0.604817 | 0.06159 | 0.056-0.068 | 3.00E-91 |
| DrnkWk | rs1260326 | Coffee consumption (cups per day) | 31837886 | chr2:27730940 | C=0.589, T=0.411 | 0.61 | 0.033 | NA | 2.00E-08 |
| DrnkWk | rs1260326 | C-reactive protein levels | 31217584 | chr2:27730940 | C=0.589, T=0.411 | NR | 0.09749534 | 0.08-0.115 | 3.00E-27 |
| DrnkWk | rs1260326 | Gamma glutamyl transferase levels | 33339817 | chr2:27730940 | C=0.589, T=0.411 | NR | 0.0561 | 0.048-0.064 | 2.00E-44 |
| DrnkWk | rs1260326 | C-reactive protein levels | 33339817 | chr2:27730940 | C=0.589, T=0.411 | NR | 0.0413 | 0.033-0.05 | 2.00E-20 |
| DrnkWk | rs1260326 | Alcohol use disorder (total score) | 30336701 | chr2:27730940 | C=0.589, T=0.411 | NR | 7.15 | NA | 9.00E-13 |
| DrnkWk | rs1260326 | Alcohol use disorder (consumption score) | 30336701 | chr2:27730940 | C=0.589, T=0.411 | NR | 0.007 | 0.005-0.009 | 6.00E-09 |
| DrnkWk | rs1260326 | Total testosterone levels | 32042192 | chr2:27730940 | C=0.589, T=0.411 | 0.606511 | 0.036635 | 0.031-0.042 | 5.00E-39 |
| DrnkWk | rs1260326 | Total testosterone levels | 32042192 | chr2:27730940 | C=0.589, T=0.411 | 0.605726 | 0.0281178 | 0.026-0.031 | 4.00E-103 |
| DrnkWk | rs1260326 | CD99/CD99L2 protein level ratio | 38412862 | chr2:27730940 | C=0.589, T=0.411 | NR | 0.0928923 | 0.081-0.104 | 8.00E-56 |
| DrnkWk | rs1260326 | COMP/TGFBI protein level ratio | 38412862 | chr2:27730940 | C=0.589, T=0.411 | NR | 0.0744571 | 0.061-0.088 | 2.00E-27 |
| DrnkWk | rs1260326 | EFNA1/PIK3IP1 protein level ratio | 38412862 | chr2:27730940 | C=0.589, T=0.411 | NR | 0.0681955 | 0.055-0.082 | 1.00E-23 |
| DrnkWk | rs1260326 | EFNA1/TNFRSF1A protein level ratio | 38412862 | chr2:27730940 | C=0.589, T=0.411 | NR | 0.0833004 | 0.07-0.097 | 8.00E-35 |
| DrnkWk | rs1260326 | ERBB2/FURIN protein level ratio | 38412862 | chr2:27730940 | C=0.589, T=0.411 | NR | 0.116433 | 0.1-0.13 | 2.00E-65 |
| DrnkWk | rs1260326 | FLT4/ICAM2 protein level ratio | 38412862 | chr2:27730940 | C=0.589, T=0.411 | NR | 0.0622585 | 0.048-0.076 | 1.00E-18 |
| DrnkWk | rs1260326 | Calcium levels | 33887147 | chr2:27730940 | C=0.589, T=0.411 | 0.6051 | 0.004453 | 0.004-0.0049/ | 4.00E-87 |
| DrnkWk | rs1260326 | CNTN1/EGFR protein level ratio | 38412862 | chr2:27730940 | C=0.589, T=0.411 | NR | 0.0752852 | 0.063-0.088 | 2.00E-31 |
| DrnkWk | rs1260326 | PLAUR/PRSS8 protein level ratio | 38412862 | chr2:27730940 | C=0.589, T=0.411 | NR | 0.0861227 | 0.073-0.099 | 3.00E-40 |
| DrnkWk | rs1260326 | CD58/ERBB3 protein level ratio | 38412862 | chr2:27730940 | C=0.589, T=0.411 | NR | 0.152808 | 0.14-0.17 | 3.00E-114 |
| DrnkWk | rs1260326 | FST/FSTL3 protein level ratio | 38412862 | chr2:27730940 | C=0.589, T=0.411 | NR | 0.137481 | 0.12-0.15 | 5.00E-86 |
| DrnkWk | rs1260326 | FURIN/IGSF3 protein level ratio | 38412862 | chr2:27730940 | C=0.589, T=0.411 | NR | 0.136483 | 0.12-0.15 | 7.00E-83 |
| DrnkWk | rs1260326 | FURIN/PLA2G15 protein level ratio | 38412862 | chr2:27730940 | C=0.589, T=0.411 | NR | 0.137226 | 0.12-0.15 | 1.00E-86 |
| DrnkWk | rs1260326 | CREG1/PLA2G15 protein level ratio | 38412862 | chr2:27730940 | C=0.589, T=0.411 | NR | 0.0656785 | 0.052-0.079 | 1.00E-20 |
| DrnkWk | rs1260326 | Alcohol use disorder (consumption score) | 33861876 | chr2:27730940 | C=0.589, T=0.411 | NR | 0.073 | 0.051-0.095 | 1.00E-11 |
| DrnkWk | rs1260326 | ITIH3/VCAM1 protein level ratio | 38412862 | chr2:27730940 | C=0.589, T=0.411 | NR | 0.070002 | 0.057-0.083 | 4.00E-26 |
| DrnkWk | rs1260326 | APOM/MCAM protein level ratio | 38412862 | chr2:27730940 | C=0.589, T=0.411 | NR | 0.132795 | 0.12-0.15 | 2.00E-87 |
| DrnkWk | rs1260326 | SCARA5/TGFBI protein level ratio | 38412862 | chr2:27730940 | C=0.589, T=0.411 | NR | 0.075174 | 0.062-0.088 | 2.00E-28 |
| DrnkWk | rs1260326 | FABP4/FGF21 protein level ratio | 38412862 | chr2:27730940 | C=0.589, T=0.411 | NR | 0.137476 | 0.12-0.15 | 2.00E-92 |
| DrnkWk | rs1260326 | F7/VASN protein level ratio | 38412862 | chr2:27730940 | C=0.589, T=0.411 | NR | 0.152172 | 0.14-0.17 | 9.00E-106 |
| DrnkWk | rs1260326 | GGH/INHBC protein level ratio | 38412862 | chr2:27730940 | C=0.589, T=0.411 | NR | 0.0919974 | 0.078-0.106 | 2.00E-38 |
| DrnkWk | rs1260326 | Appendicular lean mass | 33097823 | chr2:27730940 | C=0.589, T=0.411 | 0.3955 | 0.0323 | 0.029-0.036 | 6.00E-64 |
| DrnkWk | rs1260326 | Daily alcohol intake in rs671 GG genotype | 38277453 | chr2:27730940 | C=0.589, T=0.411 | NR | 0.071 | 0.053-0.089 | 6.00E-16 |
| DrnkWk | rs1260326 | Alcohol drinking status in rs671 GG genotype | 38277453 | chr2:27730940 | C=0.589, T=0.411 | NR | 1.08 | 1.06-1.11 | 2.00E-12 |
| DrnkWk | rs1260326 | Alcohol drinking status (ever drinker vs never drinker) | 38277453 | chr2:27730940 | C=0.589, T=0.411 | NR | 1.07 | 1.05-1.08 | 5.00E-17 |
| DrnkWk | rs1260326 | Daily alcohol intake in rs671 GA genotype | 38277453 | chr2:27730940 | C=0.589, T=0.411 | NR | 0.063 | 0.043-0.083 | 6.00E-11 |
| DrnkWk | rs1260326 | Alcohol drinking status in rs671 GA genotype | 38277453 | chr2:27730940 | C=0.589, T=0.411 | NR | 1.07 | 1.04-1.1 | 2.00E-06 |
| DrnkWk | rs1260326 | Daily alcohol intake x rs671 interaction (2df) | 38277453 | chr2:27730940 | C=0.589, T=0.411 | NR | NA | NA | 3.00E-20 |
| DrnkWk | rs1260326 | Alcohol drinking status x rs671 interaction (2df) | 38277453 | chr2:27730940 | C=0.589, T=0.411 | NR | NA | NA | 7.00E-15 |
| DrnkWk | rs1260326 | Daily alcohol intake | 38277453 | chr2:27730940 | C=0.589, T=0.411 | NR | 0.073 | 0.059-0.087 | 5.00E-25 |
| DrnkWk | rs1260326 | Urate levels | 33356394 | chr2:27730940 | C=0.589, T=0.411 | 0.39 | 0.0513 | 0.048-0.055 | 1.00E-167 |
| DrnkWk | rs1260326 | Phosphatidylcholine-2O_32:0_[M+H]1+ levels | 34503513 | chr2:27730940 | C=0.589, T=0.411 | NR | 0.0947563 | 0.071-0.119 | 8.00E-15 |
| DrnkWk | rs1260326 | Phosphatidylcholine-O_34:1_[M+H]1+/Phosphatidylcholine-P_34:0_[M+H]1+/Phosphatidylethanolamine-O_37:1_[M+H]1+ levels | 34503513 | chr2:27730940 | C=0.589, T=0.411 | NR | 0.113226 | 0.09-0.136 | 3.00E-22 |
| DrnkWk | rs1260326 | Phosphatidylcholine-O_34:3_[M+H]1+/Phosphatidylcholine-P_34:2_[M+H]1+/Phosphatidylethanolamine-P_37:2_[M+H]1+ levels | 34503513 | chr2:27730940 | C=0.589, T=0.411 | NR | 0.0916487 | 0.068-0.116 | 9.00E-14 |
| DrnkWk | rs1260326 | Phosphatidylcholine-O_30:1_[M+H]1+/Phosphatidylcholine-P_30:0_[M+H]1+/Phosphatidylethanolamine-O_33:1_[M+H]1+ levels | 34503513 | chr2:27730940 | C=0.589, T=0.411 | NR | 0.0856885 | 0.064-0.108 | 3.00E-14 |
| DrnkWk | rs1260326 | Phosphatidylcholine-O_31:0_[M+H]1+/Phosphatidylethanolamine-O_34:0_[M+H]1+ levels | 34503513 | chr2:27730940 | C=0.589, T=0.411 | NR | 0.0987751 | 0.076-0.122 | 7.00E-17 |
| DrnkWk | rs1260326 | Phosphatidylcholine-O_34:2_[M+H]1+/Phosphatidylcholine-P_34:1_[M+H]1+/Phosphatidylethanolamine-O_37:2_[M+H]1+ levels | 34503513 | chr2:27730940 | C=0.589, T=0.411 | NR | 0.0896551 | 0.065-0.114 | 5.00E-13 |
| DrnkWk | rs1260326 | Phosphatidylcholine-O_32:1_[M+H]1+/Phosphatidylcholine-P_32:0_[M+H]1+/Phosphatidylethanolamine-O_35:1_[M+H]1+ levels | 34503513 | chr2:27730940 | C=0.589, T=0.411 | NR | 0.102962 | 0.079-0.127 | 2.00E-17 |
| DrnkWk | rs1260326 | Phosphatidylcholine-O_36:2_[M+H]1+/Phosphatidylcholine-P_36:1_[M+H]1+/Phosphatidylethanolamine-P_39:1_[M+H]1+ levels | 34503513 | chr2:27730940 | C=0.589, T=0.411 | NR | 0.0941884 | 0.07-0.118 | 8.00E-15 |
| DrnkWk | rs1260326 | Phosphatidylcholine-O_36:3_[M+H]1+/Phosphatidylcholine-P_36:2_[M+H]1+ levels | 34503513 | chr2:27730940 | C=0.589, T=0.411 | NR | 0.0907773 | 0.067-0.114 | 3.00E-14 |
| DrnkWk | rs1260326 | Sphingomyelin(33:1)_[M-CH3]1- levels | 34503513 | chr2:27730940 | C=0.589, T=0.411 | NR | 0.0725 | 0.059-0.086 | 1.00E-26 |
| DrnkWk | rs1260326 | Diacylglycerol_44:7_[M+H-H2O]1+ levels | 34503513 | chr2:27730940 | C=0.589, T=0.411 | NR | 0.102061 | 0.079-0.125 | 6.00E-18 |
| DrnkWk | rs1260326 | Sphingomyelin(37:1)_[M+H]1+ levels | 34503513 | chr2:27730940 | C=0.589, T=0.411 | NR | 0.0666 | 0.054-0.079 | 1.00E-26 |
| DrnkWk | rs1260326 | Triacylglycerol_52:6_[M+NH4]1+ levels | 34503513 | chr2:27730940 | C=0.589, T=0.411 | NR | 0.0944626 | 0.065-0.124 | 2.00E-10 |
| DrnkWk | rs1260326 | Triacylglycerol_50:2_[M+NH4]1+ levels | 34503513 | chr2:27730940 | C=0.589, T=0.411 | NR | 0.0969913 | 0.067-0.127 | 2.00E-10 |
| DrnkWk | rs1260326 | Triacylglycerol_50:3_[M+NH4]1+ levels | 34503513 | chr2:27730940 | C=0.589, T=0.411 | NR | 0.106515 | 0.077-0.136 | 2.00E-12 |
| DrnkWk | rs1260326 | Triacylglycerol_50:4_[M+NH4]1+ levels | 34503513 | chr2:27730940 | C=0.589, T=0.411 | NR | 0.101962 | 0.073-0.131 | 1.00E-11 |
| DrnkWk | rs1260326 | Triacylglycerol_50:5_[M+NH4]1+ levels | 34503513 | chr2:27730940 | C=0.589, T=0.411 | NR | 0.0944012 | 0.065-0.124 | 3.00E-10 |
| DrnkWk | rs1260326 | Phosphatidylethanolamine_34:1_[M-H]1- levels | 34503513 | chr2:27730940 | C=0.589, T=0.411 | NR | 0.0710223 | 0.05-0.092 | 1.00E-11 |
| DrnkWk | rs1260326 | Diacylglycerol_32:2_[M+H-H2O]1+ levels | 34503513 | chr2:27730940 | C=0.589, T=0.411 | NR | 0.0853598 | 0.063-0.108 | 2.00E-13 |
| DrnkWk | rs1260326 | Diacylglycerol_34:3_[M+H-H2O]1+ levels | 34503513 | chr2:27730940 | C=0.589, T=0.411 | NR | 0.0795827 | 0.058-0.101 | 3.00E-13 |
| DrnkWk | rs1260326 | Diacylglycerol_34:1_[M+H-H2O]1+ levels | 34503513 | chr2:27730940 | C=0.589, T=0.411 | NR | 0.0690224 | 0.048-0.09 | 2.00E-10 |
| DrnkWk | rs1260326 | Diacylglycerol_34:2_[M+H-H2O]1+ levels | 34503513 | chr2:27730940 | C=0.589, T=0.411 | NR | 0.0687245 | 0.048-0.09 | 1.00E-10 |
| DrnkWk | rs1260326 | Lysophosphatidylethanolamine_16:0_[M-H]1- levels | 34503513 | chr2:27730940 | C=0.589, T=0.411 | NR | 0.0814369 | 0.06-0.103 | 1.00E-13 |
| DrnkWk | rs1260326 | Sphingomyelin_34:2_[M+H]1+ levels | 34503513 | chr2:27730940 | C=0.589, T=0.411 | NR | 0.0706021 | 0.049-0.093 | 4.00E-10 |
| DrnkWk | rs1260326 | Sphingomyelin_35:1_[M+H]1+ levels | 34503513 | chr2:27730940 | C=0.589, T=0.411 | NR | 0.0770633 | 0.053-0.101 | 3.00E-10 |
| DrnkWk | rs1260326 | Sphingomyelin_34:1_[M+H]1+ levels | 34503513 | chr2:27730940 | C=0.589, T=0.411 | NR | 0.105691 | 0.082-0.13 | 6.00E-18 |
| DrnkWk | rs1260326 | Sphingomyelin_33:1_[M+H]1+ levels | 34503513 | chr2:27730940 | C=0.589, T=0.411 | NR | 0.091337 | 0.068-0.115 | 2.00E-14 |
| DrnkWk | rs1260326 | Sphingomyelin_34:0_[M+H]1+ levels | 34503513 | chr2:27730940 | C=0.589, T=0.411 | NR | 0.102523 | 0.079-0.126 | 6.00E-18 |
| DrnkWk | rs1260326 | C-reactive protein levels | 29878111 | chr2:27730940 | C=0.589, T=0.411 | 0.2964 | 0.0865 | 0.065-0.108 | 9.00E-15 |
| DrnkWk | rs1260326 | Nonalcoholic fatty liver disease | 34535985 | chr2:27730940 | C=0.589, T=0.411 | 0.392 | 0.87 | 0.84-0.91 | 3.00E-15 |
| DrnkWk | rs1260326 | Triglyceride levels in very small VLDL | 35213538 | chr2:27730940 | C=0.589, T=0.411 | 0.396018 | 0.100959 | 0.093-0.109 | 9.00E-134 |
| DrnkWk | rs1260326 | Triglycerides to total lipids ratio in very small VLDL | 35213538 | chr2:27730940 | C=0.589, T=0.411 | 0.396018 | 0.0926233 | 0.085-0.1 | 2.00E-119 |
| DrnkWk | rs1260326 | Concentration of chylomicrons and extremely large VLDL particles | 35213538 | chr2:27730940 | C=0.589, T=0.411 | 0.396018 | 0.0860614 | 0.078-0.094 | 3.00E-100 |
| DrnkWk | rs1260326 | Neutrophil count | 34594039 | chr2:27730940 | C=0.589, T=0.411 | 0.439605 | 0.0262021 | 0.017-0.035 | 2.00E-08 |
| DrnkWk | rs1260326 | Albumin levels | 35213538 | chr2:27730940 | C=0.589, T=0.411 | 0.396027 | 0.0442801 | 0.036-0.053 | 5.00E-26 |
| DrnkWk | rs1260326 | Docosahexaenoic acid levels | 35213538 | chr2:27730940 | C=0.589, T=0.411 | 0.395992 | 0.0478469 | 0.04-0.056 | 7.00E-33 |
| DrnkWk | rs1260326 | Cholesterol levels in medium HDL | 35213538 | chr2:27730940 | C=0.589, T=0.411 | 0.396018 | 0.0255238 | 0.018-0.033 | 5.00E-11 |
| DrnkWk | rs1260326 | Triglyceride levels in chylomicrons and extremely large VLDL | 35213538 | chr2:27730940 | C=0.589, T=0.411 | 0.396018 | 0.0769642 | 0.069-0.085 | 8.00E-80 |
| DrnkWk | rs1260326 | Platelet count | 34594039 | chr2:27730940 | C=0.589, T=0.411 | 0.442485 | 0.0350207 | 0.028-0.042 | 8.00E-26 |
| DrnkWk | rs1260326 | LDL cholesterol levels | 35213538 | chr2:27730940 | C=0.589, T=0.411 | 0.396018 | 0.0405143 | 0.032-0.049 | 5.00E-22 |
| DrnkWk | rs1260326 | Leucine levels | 35213538 | chr2:27730940 | C=0.589, T=0.411 | 0.396023 | 0.0475855 | 0.04-0.056 | 7.00E-32 |
| DrnkWk | rs1260326 | Lactate levels | 35213538 | chr2:27730940 | C=0.589, T=0.411 | 0.396016 | 0.0294942 | 0.021-0.038 | 3.00E-12 |
| DrnkWk | rs1260326 | Total cholesterol levels | 35213538 | chr2:27730940 | C=0.589, T=0.411 | 0.396018 | 0.0355706 | 0.028-0.044 | 3.00E-18 |
| DrnkWk | rs1260326 | Cholesteryl ester levels in chylomicrons and extremely large VLDL | 35213538 | chr2:27730940 | C=0.589, T=0.411 | 0.396018 | 0.0890692 | 0.081-0.097 | 3.00E-106 |
| DrnkWk | rs1260326 | Phospholipid levels in chylomicrons and extremely large VLDL | 35213538 | chr2:27730940 | C=0.589, T=0.411 | 0.396018 | 0.0871684 | 0.079-0.095 | 3.00E-103 |
| DrnkWk | rs1260326 | Glycoprotein acetyls levels | 35213538 | chr2:27730940 | C=0.589, T=0.411 | 0.396018 | 0.101134 | 0.093-0.109 | 8.00E-130 |
| DrnkWk | rs1260326 | Medication use (HMG CoA reductase inhibitors) | 34594039 | chr2:27730940 | C=0.589, T=0.411 | 0.441796019 | 0.075442664 | 0.057-0.094 | 6.00E-16 |
| DrnkWk | rs1260326 | Triglyceride levels | 33763119 | chr2:27730940 | C=0.589, T=0.411 | NR | 9.347 | / | 3.00E-23 |
| DrnkWk | rs1260326 | Free cholesterol to total lipids ratio in chylomicrons and extremely large VLDL | 35213538 | chr2:27730940 | C=0.589, T=0.411 | 0.396639 | 0.0305836 | 0.022-0.039 | 4.00E-13 |
| DrnkWk | rs1260326 | Total lipid levels in chylomicrons and extremely large VLDL | 35213538 | chr2:27730940 | C=0.589, T=0.411 | 0.396018 | 0.0830804 | 0.075-0.091 | 4.00E-93 |
| DrnkWk | rs1260326 | Acetate levels | 35213538 | chr2:27730940 | C=0.589, T=0.411 | 0.396028 | 0.0305693 | 0.022-0.039 | 6.00E-13 |
| DrnkWk | rs1260326 | Alanine levels | 35213538 | chr2:27730940 | C=0.589, T=0.411 | 0.396018 | 0.0559618 | 0.048-0.064 | 7.00E-41 |
| DrnkWk | rs1260326 | Serum uric acid levels | 34594039 | chr2:27730940 | C=0.589, T=0.411 | 0.441833 | 0.0314887 | 0.025-0.038 | 4.00E-19 |
| DrnkWk | rs1260326 | Serum total protein levels | 34594039 | chr2:27730940 | C=0.589, T=0.411 | 0.44179 | 0.0393828 | 0.032-0.047 | 2.00E-25 |
| DrnkWk | rs1260326 | Triglycerides | 34594039 | chr2:27730940 | C=0.589, T=0.411 | 0.442588 | 0.0863985 | 0.078-0.094 | 7.00E-102 |
| DrnkWk | rs1260326 | Isoleucine levels | 35213538 | chr2:27730940 | C=0.589, T=0.411 | 0.396019 | 0.0467023 | 0.039-0.055 | 2.00E-29 |
| DrnkWk | rs1260326 | Pyruvate levels | 35213538 | chr2:27730940 | C=0.589, T=0.411 | 0.395951 | 0.0376021 | 0.029-0.046 | 6.00E-19 |
| DrnkWk | rs1260326 | Total free cholesterol levels | 35213538 | chr2:27730940 | C=0.589, T=0.411 | 0.396018 | 0.040846 | 0.033-0.049 | 4.00E-23 |
| DrnkWk | rs1260326 | Cholesterol levels in chylomicrons and extremely large VLDL | 35213538 | chr2:27730940 | C=0.589, T=0.411 | 0.396018 | 0.0893474 | 0.081-0.097 | 2.00E-107 |
| DrnkWk | rs1260326 | Free cholesterol levels in chylomicrons and extremely large VLDL | 35213538 | chr2:27730940 | C=0.589, T=0.411 | 0.396018 | 0.0879509 | 0.08-0.096 | 3.00E-104 |
| DrnkWk | rs1260326 | Phospholipids to total lipids ratio in chylomicrons and extremely large VLDL | 35213538 | chr2:27730940 | C=0.589, T=0.411 | 0.396639 | 0.0344321 | 0.026-0.043 | 4.00E-16 |
| DrnkWk | rs1260326 | Glutamine levels | 35213538 | chr2:27730940 | C=0.589, T=0.411 | 0.395949 | 0.0789422 | 0.071-0.087 | 4.00E-78 |
| DrnkWk | rs1260326 | Medication use (drugs used in diabetes) | 34594039 | chr2:27730940 | C=0.589, T=0.411 | 0.441796019 | 0.059331838 | 0.04-0.079 | 2.00E-09 |
| DrnkWk | rs1260326 | Linoleic acid levels | 35213538 | chr2:27730940 | C=0.589, T=0.411 | 0.395992 | 0.0532357 | 0.045-0.061 | 8.00E-38 |
| DrnkWk | rs1260326 | Cholecystitis | 34594039 | chr2:27730940 | C=0.589, T=0.411 | NR | 0.0772 | 0.05-0.104 | 2.00E-08 |
| DrnkWk | rs1260326 | Cholelithiasis | 34594039 | chr2:27730940 | C=0.589, T=0.411 | NR | 0.0819 | 0.065-0.098 | 1.00E-22 |
| DrnkWk | rs1260326 | Polyunsaturated fatty acid levels | 35213538 | chr2:27730940 | C=0.589, T=0.411 | 0.395992 | 0.0769268 | 0.069-0.085 | 1.00E-78 |
| DrnkWk | rs1260326 | Coronary artery disease or factor VII levels (pleiotropy) | 35285134 | chr2:27730940 | C=0.589, T=0.411 | NR | NA | NA | 4.00E-30 |
| DrnkWk | rs1260326 | Total esterified cholesterol levels | 35213538 | chr2:27730940 | C=0.589, T=0.411 | 0.396018 | 0.0331689 | 0.025-0.041 | 4.00E-16 |
| DrnkWk | rs1260326 | Omega-3 fatty acid levels | 35213538 | chr2:27730940 | C=0.589, T=0.411 | 0.395992 | 0.0830756 | 0.075-0.091 | 8.00E-94 |
| DrnkWk | rs1260326 | Total cholesterol minus HDL-C levels | 35213538 | chr2:27730940 | C=0.589, T=0.411 | 0.396018 | 0.0398589 | 0.032-0.048 | 2.00E-21 |
| DrnkWk | rs1260326 | Phospholipid levels in small HDL | 35213538 | chr2:27730940 | C=0.589, T=0.411 | 0.396018 | 0.103413 | 0.095-0.112 | 3.00E-135 |
| DrnkWk | rs1260326 | Triglyceride levels in small HDL | 35213538 | chr2:27730940 | C=0.589, T=0.411 | 0.396018 | 0.106758 | 0.099-0.115 | 1.00E-151 |
| DrnkWk | rs1260326 | Cholesterol to total lipids ratio in small VLDL | 35213538 | chr2:27730940 | C=0.589, T=0.411 | 0.396018 | 0.0680451 | 0.06-0.076 | 5.00E-62 |
| DrnkWk | rs1260326 | Triglycerides to total lipids ratio in small LDL | 35213538 | chr2:27730940 | C=0.589, T=0.411 | 0.396018 | 0.0849433 | 0.077-0.093 | 3.00E-97 |
| DrnkWk | rs1260326 | Cholesterol levels in small VLDL | 35213538 | chr2:27730940 | C=0.589, T=0.411 | 0.396018 | 0.0579202 | 0.05-0.066 | 5.00E-43 |
| DrnkWk | rs1260326 | Phospholipid levels in small VLDL | 35213538 | chr2:27730940 | C=0.589, T=0.411 | 0.396018 | 0.0693681 | 0.061-0.078 | 3.00E-61 |
| DrnkWk | rs1260326 | Total lipid levels in VLDL | 35213538 | chr2:27730940 | C=0.589, T=0.411 | 0.396018 | 0.0885351 | 0.08-0.097 | 4.00E-102 |
| DrnkWk | rs1260326 | Concentration of VLDL particles | 35213538 | chr2:27730940 | C=0.589, T=0.411 | 0.396018 | 0.0760654 | 0.068-0.084 | 6.00E-74 |
| DrnkWk | rs1260326 | Cholesteryl ester levels in very large VLDL | 35213538 | chr2:27730940 | C=0.589, T=0.411 | 0.396018 | 0.0758474 | 0.068-0.084 | 1.00E-74 |
| DrnkWk | rs1260326 | Cholesteryl esters to total lipids ratio in very small VLDL | 35213538 | chr2:27730940 | C=0.589, T=0.411 | 0.396018 | 0.0865383 | 0.079-0.094 | 3.00E-106 |
| DrnkWk | rs1260326 | Ratio of omega-3 fatty acids to total fatty acids | 35213538 | chr2:27730940 | C=0.589, T=0.411 | 0.395992 | 0.03861 | 0.031-0.047 | 2.00E-21 |
| DrnkWk | rs1260326 | Omega-6 fatty acid levels | 35213538 | chr2:27730940 | C=0.589, T=0.411 | 0.395992 | 0.064724 | 0.057-0.073 | 1.00E-55 |
| DrnkWk | rs1260326 | Ratio of omega-6 fatty acids to omega-3 fatty acids | 35213538 | chr2:27730940 | C=0.589, T=0.411 | 0.395992 | 0.066446 | 0.058-0.074 | 3.00E-60 |
| DrnkWk | rs1260326 | Cholesterol levels in small LDL | 35213538 | chr2:27730940 | C=0.589, T=0.411 | 0.396018 | 0.0464557 | 0.038-0.055 | 4.00E-28 |
| DrnkWk | rs1260326 | Free cholesterol to total lipids ratio in small VLDL | 35213538 | chr2:27730940 | C=0.589, T=0.411 | 0.396018 | 0.0719562 | 0.064-0.08 | 4.00E-72 |
| DrnkWk | rs1260326 | Free cholesterol levels in small VLDL | 35213538 | chr2:27730940 | C=0.589, T=0.411 | 0.396018 | 0.050513 | 0.042-0.059 | 4.00E-33 |
| DrnkWk | rs1260326 | Total concentration of branched-chain amino acids (leucine + isoleucine + valine) | 35213538 | chr2:27730940 | C=0.589, T=0.411 | 0.396024 | 0.0514666 | 0.043-0.059 | 2.00E-36 |
| DrnkWk | rs1260326 | Total lipid levels in lipoprotein particles | 35213538 | chr2:27730940 | C=0.589, T=0.411 | 0.396018 | 0.0744082 | 0.066-0.083 | 5.00E-72 |
| DrnkWk | rs1260326 | Total fatty acid levels | 35213538 | chr2:27730940 | C=0.589, T=0.411 | 0.395992 | 0.105845 | 0.098-0.114 | 1.00E-142 |
| DrnkWk | rs1260326 | Triglyceride levels in very large HDL | 35213538 | chr2:27730940 | C=0.589, T=0.411 | 0.396018 | 0.0753301 | 0.067-0.083 | 5.00E-75 |
| DrnkWk | rs1260326 | Phospholipids to total lipids ratio in very large HDL | 35213538 | chr2:27730940 | C=0.589, T=0.411 | 0.396025 | 0.056206 | 0.049-0.064 | 1.00E-47 |
| DrnkWk | rs1260326 | Serum albumin levels | 34594039 | chr2:27730940 | C=0.589, T=0.411 | 0.442009 | 0.0556682 | 0.048-0.063 | 8.00E-49 |
| DrnkWk | rs1260326 | Total lipid levels in very large VLDL | 35213538 | chr2:27730940 | C=0.589, T=0.411 | 0.396018 | 0.0908106 | 0.083-0.099 | 3.00E-111 |
| DrnkWk | rs1260326 | Free cholesterol to total lipids ratio in very small VLDL | 35213538 | chr2:27730940 | C=0.589, T=0.411 | 0.396018 | 0.0619232 | 0.054-0.07 | 2.00E-49 |
| DrnkWk | rs1260326 | Total lipid levels in very small VLDL | 35213538 | chr2:27730940 | C=0.589, T=0.411 | 0.396018 | 0.0457394 | 0.038-0.054 | 2.00E-28 |
| DrnkWk | rs1260326 | Triglycerides to total lipids ratio in medium VLDL | 35213538 | chr2:27730940 | C=0.589, T=0.411 | 0.396018 | 0.0728815 | 0.065-0.081 | 5.00E-75 |
| DrnkWk | rs1260326 | Triglyceride levels in medium VLDL | 35213538 | chr2:27730940 | C=0.589, T=0.411 | 0.396018 | 0.0957627 | 0.088-0.104 | 2.00E-121 |
| DrnkWk | rs1260326 | Cholesteryl esters to total lipids ratio in small HDL | 35213538 | chr2:27730940 | C=0.589, T=0.411 | 0.396018 | 0.0504016 | 0.042-0.058 | 2.00E-34 |
| DrnkWk | rs1260326 | Free cholesterol levels in small HDL | 35213538 | chr2:27730940 | C=0.589, T=0.411 | 0.396018 | 0.0976834 | 0.09-0.106 | 2.00E-123 |
| DrnkWk | rs1260326 | Concentration of medium HDL particles | 35213538 | chr2:27730940 | C=0.589, T=0.411 | 0.396018 | 0.0390425 | 0.031-0.047 | 9.00E-24 |
| DrnkWk | rs1260326 | Valine levels | 35213538 | chr2:27730940 | C=0.589, T=0.411 | 0.396021 | 0.051522 | 0.043-0.06 | 4.00E-36 |
| DrnkWk | rs1260326 | Degree of unsaturation | 35213538 | chr2:27730940 | C=0.589, T=0.411 | 0.395992 | 0.0395183 | 0.032-0.047 | 5.00E-23 |
| DrnkWk | rs1260326 | Cholesteryl ester levels in very large HDL | 35213538 | chr2:27730940 | C=0.589, T=0.411 | 0.396018 | 0.0546903 | 0.047-0.062 | 2.00E-47 |
| DrnkWk | rs1260326 | Triglycerides to total lipids ratio in very large HDL | 35213538 | chr2:27730940 | C=0.589, T=0.411 | 0.396025 | 0.0979442 | 0.09-0.106 | 4.00E-135 |
| DrnkWk | rs1260326 | Phospholipid levels in very large VLDL | 35213538 | chr2:27730940 | C=0.589, T=0.411 | 0.396018 | 0.0864923 | 0.079-0.094 | 2.00E-100 |
| DrnkWk | rs1260326 | Concentration of very large VLDL particles | 35213538 | chr2:27730940 | C=0.589, T=0.411 | 0.396018 | 0.0917567 | 0.084-0.1 | 7.00E-114 |
| DrnkWk | rs1260326 | Phospholipid levels in very small VLDL | 35213538 | chr2:27730940 | C=0.589, T=0.411 | 0.396018 | 0.04814 | 0.04-0.056 | 3.00E-31 |
| DrnkWk | rs1260326 | Plasma ANGPTL8 levels | 33619548 | chr2:27730940 | C=0.589, T=0.411 | NR | 0.18 | NA | 4.00E-07 |
| DrnkWk | rs1260326 | Concentration of very small VLDL particles | 35213538 | chr2:27730940 | C=0.589, T=0.411 | 0.396018 | 0.0429408 | 0.035-0.051 | 4.00E-25 |
| DrnkWk | rs1260326 | Phosphatidylcholine levels | 35213538 | chr2:27730940 | C=0.589, T=0.411 | 0.395992 | 0.0652765 | 0.058-0.073 | 2.00E-61 |
| DrnkWk | rs1260326 | Ratio of omega-6 fatty acids to total fatty acids | 35213538 | chr2:27730940 | C=0.589, T=0.411 | 0.395992 | 0.109743 | 0.1-0.12 | 4.00E-162 |
| DrnkWk | rs1260326 | Triglycerides to total lipids ratio in small HDL | 35213538 | chr2:27730940 | C=0.589, T=0.411 | 0.396018 | 0.0730859 | 0.065-0.081 | 1.00E-74 |
| DrnkWk | rs1260326 | Triglyceride levels in small LDL | 35213538 | chr2:27730940 | C=0.589, T=0.411 | 0.396018 | 0.104733 | 0.097-0.113 | 8.00E-145 |
| DrnkWk | rs1260326 | Phospholipids to total lipids ratio in small LDL | 35213538 | chr2:27730940 | C=0.589, T=0.411 | 0.396018 | 0.0835214 | 0.075-0.092 | 2.00E-87 |
| DrnkWk | rs1260326 | Phospholipids to total lipids ratio in small VLDL | 35213538 | chr2:27730940 | C=0.589, T=0.411 | 0.396018 | 0.0709788 | 0.063-0.079 | 6.00E-70 |
| DrnkWk | rs1260326 | Triglyceride levels in small VLDL | 35213538 | chr2:27730940 | C=0.589, T=0.411 | 0.396018 | 0.105493 | 0.098-0.113 | 1.00E-148 |
| DrnkWk | rs1260326 | Triglyceride levels in VLDL | 35213538 | chr2:27730940 | C=0.589, T=0.411 | 0.396018 | 0.0958623 | 0.088-0.104 | 6.00E-124 |
| DrnkWk | rs1260326 | Phospholipid levels in VLDL | 35213538 | chr2:27730940 | C=0.589, T=0.411 | 0.396018 | 0.0822049 | 0.074-0.09 | 5.00E-87 |
| DrnkWk | rs1260326 | Average diameter for VLDL particles | 35213538 | chr2:27730940 | C=0.589, T=0.411 | 0.396018 | 0.083605 | 0.076-0.091 | 3.00E-100 |
| DrnkWk | rs1260326 | Concentration of very large HDL particles | 35213538 | chr2:27730940 | C=0.589, T=0.411 | 0.396018 | 0.0378791 | 0.03-0.045 | 2.00E-23 |
| DrnkWk | rs1260326 | Cholesteryl esters to total lipids ratio in very large VLDL | 35213538 | chr2:27730940 | C=0.589, T=0.411 | 0.39619 | 0.0747131 | 0.067-0.083 | 1.00E-76 |
| DrnkWk | rs1260326 | Cholesterol to total lipids ratio in very small VLDL | 35213538 | chr2:27730940 | C=0.589, T=0.411 | 0.396018 | 0.086527 | 0.079-0.094 | 8.00E-105 |
| DrnkWk | rs1260326 | Phospholipid levels in medium VLDL | 35213538 | chr2:27730940 | C=0.589, T=0.411 | 0.396018 | 0.0619596 | 0.054-0.07 | 5.00E-49 |
| DrnkWk | rs1260326 | Cholesteryl esters to total lipids ratio in medium VLDL | 35213538 | chr2:27730940 | C=0.589, T=0.411 | 0.396018 | 0.0800727 | 0.072-0.088 | 2.00E-91 |
| DrnkWk | rs1260326 | Free cholesterol to total lipids ratio in medium VLDL | 35213538 | chr2:27730940 | C=0.589, T=0.411 | 0.396018 | 0.0650849 | 0.057-0.073 | 4.00E-59 |
| DrnkWk | rs1260326 | Phospholipids to total lipids ratio in medium VLDL | 35213538 | chr2:27730940 | C=0.589, T=0.411 | 0.396018 | 0.0528421 | 0.045-0.061 | 6.00E-39 |
| DrnkWk | rs1260326 | Cholesterol levels in small HDL | 35213538 | chr2:27730940 | C=0.589, T=0.411 | 0.396018 | 0.0847072 | 0.076-0.093 | 1.00E-89 |
| DrnkWk | rs1260326 | Cholesterol to total lipids ratio in small HDL | 35213538 | chr2:27730940 | C=0.589, T=0.411 | 0.396018 | 0.0535061 | 0.045-0.062 | 4.00E-38 |
| DrnkWk | rs1260326 | Cholesteryl ester levels in small HDL | 35213538 | chr2:27730940 | C=0.589, T=0.411 | 0.396018 | 0.0757393 | 0.067-0.084 | 1.00E-71 |
| DrnkWk | rs1260326 | Free cholesterol to total lipids ratio in small LDL | 35213538 | chr2:27730940 | C=0.589, T=0.411 | 0.396018 | 0.0837481 | 0.076-0.092 | 2.00E-94 |
| DrnkWk | rs1260326 | Total lipid levels in small VLDL | 35213538 | chr2:27730940 | C=0.589, T=0.411 | 0.396018 | 0.0878763 | 0.08-0.096 | 4.00E-99 |
| DrnkWk | rs1260326 | Concentration of small VLDL particles | 35213538 | chr2:27730940 | C=0.589, T=0.411 | 0.396018 | 0.0879906 | 0.08-0.096 | 5.00E-100 |
| DrnkWk | rs1260326 | Ratio of saturated fatty acids to total fatty acids | 35213538 | chr2:27730940 | C=0.589, T=0.411 | 0.395992 | 0.0553075 | 0.047-0.063 | 3.00E-40 |
| DrnkWk | rs1260326 | Ratio of triglycerides to phosphoglycerides | 35213538 | chr2:27730940 | C=0.589, T=0.411 | 0.395992 | 0.0763245 | 0.069-0.084 | 7.00E-85 |
| DrnkWk | rs1260326 | Sphingomyelin levels | 35213538 | chr2:27730940 | C=0.589, T=0.411 | 0.395992 | 0.031068 | 0.023-0.039 | 3.00E-15 |
| DrnkWk | rs1260326 | Free cholesterol levels in very large HDL | 35213538 | chr2:27730940 | C=0.589, T=0.411 | 0.396018 | 0.0725692 | 0.065-0.08 | 5.00E-76 |
| DrnkWk | rs1260326 | Cholesterol to total lipids ratio in very large VLDL | 35213538 | chr2:27730940 | C=0.589, T=0.411 | 0.39619 | 0.0773756 | 0.069-0.085 | 4.00E-82 |
| DrnkWk | rs1260326 | Gamma glutamyl transpeptidase | 34594039 | chr2:27730940 | C=0.589, T=0.411 | 0.443484 | 0.0556103 | 0.049-0.062 | 4.00E-57 |
| DrnkWk | rs1260326 | Glucose levels | 34594039 | chr2:27730940 | C=0.589, T=0.411 | 0.441604 | 0.031105 | 0.024-0.038 | 2.00E-19 |
| DrnkWk | rs1260326 | Triglycerides to total lipids ratio in very large VLDL | 35213538 | chr2:27730940 | C=0.589, T=0.411 | 0.39619 | 0.0722331 | 0.064-0.08 | 5.00E-70 |
| DrnkWk | rs1260326 | Free cholesterol levels in medium VLDL | 35213538 | chr2:27730940 | C=0.589, T=0.411 | 0.396018 | 0.0505425 | 0.042-0.059 | 4.00E-33 |
| DrnkWk | rs1260326 | Total lipid levels in medium VLDL | 35213538 | chr2:27730940 | C=0.589, T=0.411 | 0.396018 | 0.0764126 | 0.068-0.085 | 2.00E-74 |
| DrnkWk | rs1260326 | Concentration of medium VLDL particles | 35213538 | chr2:27730940 | C=0.589, T=0.411 | 0.396018 | 0.0651475 | 0.057-0.073 | 4.00E-54 |
| DrnkWk | rs1260326 | Phosphoglycerides levels | 35213538 | chr2:27730940 | C=0.589, T=0.411 | 0.395992 | 0.0744097 | 0.067-0.082 | 7.00E-78 |
| DrnkWk | rs1260326 | Ratio of polyunsaturated fatty acids to monounsaturated fatty acids | 35213538 | chr2:27730940 | C=0.589, T=0.411 | 0.395992 | 0.0928958 | 0.085-0.101 | 4.00E-117 |
| DrnkWk | rs1260326 | Ratio of polyunsaturated fatty acids to total fatty acids | 35213538 | chr2:27730940 | C=0.589, T=0.411 | 0.395992 | 0.0904386 | 0.082-0.098 | 2.00E-110 |
| DrnkWk | rs1260326 | Cholesteryl ester levels in small LDL | 35213538 | chr2:27730940 | C=0.589, T=0.411 | 0.396018 | 0.0589652 | 0.051-0.067 | 2.00E-44 |
| DrnkWk | rs1260326 | Cholesteryl esters to total lipids ratio in small LDL | 35213538 | chr2:27730940 | C=0.589, T=0.411 | 0.396018 | 0.0770359 | 0.069-0.085 | 1.00E-74 |
| DrnkWk | rs1260326 | Cholesteryl esters to total lipids ratio in small VLDL | 35213538 | chr2:27730940 | C=0.589, T=0.411 | 0.396018 | 0.0577308 | 0.05-0.066 | 8.00E-44 |
| DrnkWk | rs1260326 | Cholesteryl ester levels in small VLDL | 35213538 | chr2:27730940 | C=0.589, T=0.411 | 0.396018 | 0.0613229 | 0.053-0.07 | 3.00E-48 |
| DrnkWk | rs1260326 | Triglycerides to total lipids ratio in small VLDL | 35213538 | chr2:27730940 | C=0.589, T=0.411 | 0.396018 | 0.0700247 | 0.062-0.078 | 2.00E-66 |
| DrnkWk | rs1260326 | Saturated fatty acid levels | 35213538 | chr2:27730940 | C=0.589, T=0.411 | 0.395992 | 0.106394 | 0.098-0.115 | 2.00E-144 |
| DrnkWk | rs1260326 | Free cholesterol levels in VLDL | 35213538 | chr2:27730940 | C=0.589, T=0.411 | 0.396018 | 0.0758241 | 0.068-0.084 | 1.00E-73 |
| DrnkWk | rs1260326 | Cholesteryl ester levels in VLDL | 35213538 | chr2:27730940 | C=0.589, T=0.411 | 0.396018 | 0.0501775 | 0.042-0.058 | 1.00E-32 |
| DrnkWk | rs1260326 | VLDL cholesterol levels | 35213538 | chr2:27730940 | C=0.589, T=0.411 | 0.396018 | 0.0622716 | 0.054-0.071 | 1.00E-49 |
| DrnkWk | rs1260326 | Total lipid levels in very large HDL | 35213538 | chr2:27730940 | C=0.589, T=0.411 | 0.396018 | 0.0531173 | 0.046-0.061 | 1.00E-44 |
| DrnkWk | rs1260326 | Cholesterol levels in very large VLDL | 35213538 | chr2:27730940 | C=0.589, T=0.411 | 0.396018 | 0.081656 | 0.074-0.09 | 1.00E-87 |
| DrnkWk | rs1260326 | Urolithiasis | 34594039 | chr2:27730940 | C=0.589, T=0.411 | 0.443371184 | 0.10490457 | 0.077-0.133 | 4.00E-13 |
| DrnkWk | rs1260326 | Free cholesterol to total lipids ratio in very large VLDL | 35213538 | chr2:27730940 | C=0.589, T=0.411 | 0.39619 | 0.0778523 | 0.07-0.086 | 6.00E-81 |
| DrnkWk | rs1260326 | Free cholesterol levels in very large VLDL | 35213538 | chr2:27730940 | C=0.589, T=0.411 | 0.396018 | 0.0848325 | 0.077-0.093 | 4.00E-96 |
| DrnkWk | rs1260326 | Calcium levels | 34594039 | chr2:27730940 | C=0.589, T=0.411 | 0.441092 | 0.0374873 | 0.028-0.047 | 3.00E-15 |
| DrnkWk | rs1260326 | Triglyceride levels in very large VLDL | 35213538 | chr2:27730940 | C=0.589, T=0.411 | 0.396018 | 0.0937141 | 0.086-0.102 | 8.00E-120 |
| DrnkWk | rs1260326 | Ratio of monounsaturated fatty acids to total fatty acids | 35213538 | chr2:27730940 | C=0.589, T=0.411 | 0.395992 | 0.0880817 | 0.08-0.096 | 2.00E-105 |
| DrnkWk | rs1260326 | Monounsaturated fatty acid levels | 35213538 | chr2:27730940 | C=0.589, T=0.411 | 0.395992 | 0.111017 | 0.1-0.12 | 3.00E-157 |
| DrnkWk | rs1260326 | Total lipid levels in small HDL | 35213538 | chr2:27730940 | C=0.589, T=0.411 | 0.396018 | 0.106016 | 0.098-0.114 | 2.00E-140 |
| DrnkWk | rs1260326 | Concentration of small HDL particles | 35213538 | chr2:27730940 | C=0.589, T=0.411 | 0.396018 | 0.0924126 | 0.084-0.101 | 4.00E-106 |
| DrnkWk | rs1260326 | Total lipid levels in small LDL | 35213538 | chr2:27730940 | C=0.589, T=0.411 | 0.396018 | 0.0500381 | 0.042-0.058 | 2.00E-32 |
| DrnkWk | rs1260326 | Concentration of small LDL particles | 35213538 | chr2:27730940 | C=0.589, T=0.411 | 0.396018 | 0.0483744 | 0.04-0.057 | 2.00E-30 |
| DrnkWk | rs1260326 | Total phospholipid levels in lipoprotein particles | 35213538 | chr2:27730940 | C=0.589, T=0.411 | 0.396018 | 0.0649234 | 0.057-0.073 | 6.00E-59 |
| DrnkWk | rs1260326 | Total triglycerides levels | 35213538 | chr2:27730940 | C=0.589, T=0.411 | 0.396018 | 0.100041 | 0.092-0.108 | 9.00E-134 |
| DrnkWk | rs1260326 | Total concentration of lipoprotein particles | 35213538 | chr2:27730940 | C=0.589, T=0.411 | 0.396018 | 0.0573987 | 0.05-0.065 | 2.00E-47 |
| DrnkWk | rs1260326 | Cholesterol levels in very large HDL | 35213538 | chr2:27730940 | C=0.589, T=0.411 | 0.396018 | 0.059043 | 0.052-0.067 | 3.00E-54 |
| DrnkWk | rs1260326 | Phospholipid levels in very large HDL | 35213538 | chr2:27730940 | C=0.589, T=0.411 | 0.396018 | 0.055096 | 0.048-0.063 | 7.00E-48 |
| DrnkWk | rs1260326 | Height | 34594039 | chr2:27730940 | C=0.589, T=0.411 | 0.441455 | 0.0155541 | 0.012-0.019 | 9.00E-15 |
| DrnkWk | rs1260326 | Phospholipids to total lipids ratio in very small VLDL | 35213538 | chr2:27730940 | C=0.589, T=0.411 | 0.396018 | 0.0250599 | 0.017-0.033 | 1.00E-09 |
| DrnkWk | rs1260326 | Fibrinogen levels or factor VII levels or factor XI levels or tissue plasminogen activator levels (pleiotropy) | 35285134 | chr2:27730940 | C=0.589, T=0.411 | NR | NA | NA | 8.00E-37 |
| DrnkWk | rs1260326 | Venous thromboembolism or factor VII levels (pleiotropy) | 35285134 | chr2:27730940 | C=0.589, T=0.411 | NR | NA | NA | 6.00E-30 |
| DrnkWk | rs1260326 | Serum alkaline phosphatase levels | 34594039 | chr2:27730940 | C=0.589, T=0.411 | NR | 0.043 | 0.039-0.047 | 2.00E-99 |
| DrnkWk | rs1260326 | Type 2 diabetes | 34594039 | chr2:27730940 | C=0.589, T=0.411 | NR | 0.071 | 0.058-0.084 | 6.00E-29 |
| DrnkWk | rs1260326 | Fibrinogen levels or factor VII levels (pleiotropy) | 35285134 | chr2:27730940 | C=0.589, T=0.411 | NR | NA | NA | 1.00E-32 |
| DrnkWk | rs1260326 | Ischemic stroke or factor VII levels (pleiotropy) | 35285134 | chr2:27730940 | C=0.589, T=0.411 | NR | NA | NA | 1.00E-30 |
| DrnkWk | rs1260326 | Serum albumin levels | 34594039 | chr2:27730940 | C=0.589, T=0.411 | NR | 0.058 | 0.054-0.062 | 2.00E-168 |
| DrnkWk | rs1260326 | Urolithiasis | 34594039 | chr2:27730940 | C=0.589, T=0.411 | NR | 0.0965 | 0.074-0.119 | 6.00E-17 |
| DrnkWk | rs1260326 | Fasting insulin adjusted for BMI | 34951656 | chr2:27730940 | C=0.589, T=0.411 | 0.35 | 0.0782 | 0.064-0.092 | 3.00E-28 |
| DrnkWk | rs1260326 | Fasting glucose adjusted for BMI | 34951656 | chr2:27730940 | C=0.589, T=0.411 | 0.3479 | 0.076 | 0.062-0.09 | 8.00E-28 |
| DrnkWk | rs1260326 | Fasting glucose adjusted for BMI | 34951656 | chr2:27730940 | C=0.589, T=0.411 | 0.3419 | 0.0843 | 0.063-0.106 | 3.00E-14 |
| DrnkWk | rs1260326 | Fasting glucose adjusted for BMI | 34951656 | chr2:27730940 | C=0.589, T=0.411 | 0.4103 | 0.085 | 0.065-0.105 | 2.00E-16 |
| DrnkWk | rs1260326 | Blood urea nitrogen levels | 34594039 | chr2:27730940 | C=0.589, T=0.411 | NR | 0.0204 | 0.017-0.024 | 4.00E-26 |
| DrnkWk | rs1260326 | Calcium levels | 34594039 | chr2:27730940 | C=0.589, T=0.411 | NR | 0.0464 | 0.042-0.051 | 1.00E-95 |
| DrnkWk | rs1260326 | Glycated haemoglobin HbA1c levels adjusted for BMI | 34951656 | chr2:27730940 | C=0.589, T=0.411 | 0.4074 | 0.0981 | 0.078-0.118 | 4.00E-21 |
| DrnkWk | rs1260326 | Glycated haemoglobin HbA1c levels adjusted for BMI | 34951656 | chr2:27730940 | C=0.589, T=0.411 | 0.3448 | 0.0592 | 0.035-0.084 | 2.00E-06 |
| DrnkWk | rs1260326 | Triacylglycerol (48:2) [NL-16:0] levels | 35668104 | chr2:27730940 | C=0.589, T=0.411 | NR | NA | NA | 4.00E-15 |
| DrnkWk | rs1260326 | Creatinine levels | 33414548 | chr2:27730940 | C=0.589, T=0.411 | 0.6123 | 8.309 | () | 1.00E-16 |
| DrnkWk | rs1260326 | Isoleucine levels | 33414548 | chr2:27730940 | C=0.589, T=0.411 | 0.3881 | 9.619 | () | 7.00E-22 |
| DrnkWk | rs1260326 | Leucine levels | 33414548 | chr2:27730940 | C=0.589, T=0.411 | 0.388 | 9.375 | () | 7.00E-21 |
| DrnkWk | rs1260326 | lysoPhosphatidylcholine acyl C14:0 levels | 33414548 | chr2:27730940 | C=0.589, T=0.411 | 0.4053 | 8.419 | () | 4.00E-17 |
| DrnkWk | rs1260326 | lysoPhosphatidylcholine acyl C16:0 levels | 33414548 | chr2:27730940 | C=0.589, T=0.411 | 0.3976 | 8.046 | () | 9.00E-16 |
| DrnkWk | rs1260326 | Phosphatidylcholine diacyl C32:1 levels | 33414548 | chr2:27730940 | C=0.589, T=0.411 | 0.3969 | 8.828 | () | 1.00E-18 |
| DrnkWk | rs1260326 | Phosphatidylcholine diacyl C32:2 levels | 33414548 | chr2:27730940 | C=0.589, T=0.411 | 0.395 | 10.859 | () | 2.00E-27 |
| DrnkWk | rs1260326 | Phosphatidylcholine diacyl C34:1 levels | 33414548 | chr2:27730940 | C=0.589, T=0.411 | 0.3984 | 7.694 | () | 1.00E-14 |
| DrnkWk | rs1260326 | lysoPhosphatidylcholine acyl C16:1 levels | 33414548 | chr2:27730940 | C=0.589, T=0.411 | 0.3969 | 9.082 | () | 1.00E-19 |
| DrnkWk | rs1260326 | lysoPhosphatidylcholine acyl C18:0 levels | 33414548 | chr2:27730940 | C=0.589, T=0.411 | 0.397 | 6.808 | () | 1.00E-11 |
| DrnkWk | rs1260326 | lysoPhosphatidylcholine acyl C20:3 levels | 33414548 | chr2:27730940 | C=0.589, T=0.411 | 0.3987 | 7.378 | () | 2.00E-13 |
| DrnkWk | rs1260326 | Glutamine levels | 33414548 | chr2:27730940 | C=0.589, T=0.411 | 0.6119 | 13.557 | () | 7.00E-42 |
| DrnkWk | rs1260326 | Weight | 34594039 | chr2:27730940 | C=0.589, T=0.411 | NR | 0.0161 | 0.013-0.019 | 4.00E-22 |
| DrnkWk | rs1260326 | Asparagine levels | 33414548 | chr2:27730940 | C=0.589, T=0.411 | 0.5983 | 7.781 | () | 7.00E-15 |
| DrnkWk | rs1260326 | Alanine levels | 33414548 | chr2:27730940 | C=0.589, T=0.411 | 0.3881 | 14.574 | () | 4.00E-48 |
| DrnkWk | rs1260326 | Threonine levels | 33414548 | chr2:27730940 | C=0.589, T=0.411 | 0.598 | 13.859 | () | 1.00E-43 |
| DrnkWk | rs1260326 | Estimated glomerular filtration rate (creatinine) | 35710981 | chr2:27730940 | C=0.589, T=0.411 | 0.5988 | 22.363 | NA | 9.00E-111 |
| DrnkWk | rs1260326 | Phosphatidylcholine diacyl C40:5 levels | 33414548 | chr2:27730940 | C=0.589, T=0.411 | 0.3978 | 11.009 | () | 3.00E-28 |
| DrnkWk | rs1260326 | Phosphatidylcholine diacyl C40:6 levels | 33414548 | chr2:27730940 | C=0.589, T=0.411 | 0.3981 | 7.683 | () | 2.00E-14 |
| DrnkWk | rs1260326 | Phosphatidylcholine diacyl C34:3 levels | 33414548 | chr2:27730940 | C=0.589, T=0.411 | 0.3966 | 9.116 | () | 8.00E-20 |
| DrnkWk | rs1260326 | Phosphatidylcholine diacyl C34:4 levels | 33414548 | chr2:27730940 | C=0.589, T=0.411 | 0.397 | 9.146 | () | 6.00E-20 |
| DrnkWk | rs1260326 | Serine levels | 33414548 | chr2:27730940 | C=0.589, T=0.411 | 0.5982 | 10.18 | () | 2.00E-24 |
| DrnkWk | rs1260326 | Phosphatidylcholine diacyl C36:6 levels | 33414548 | chr2:27730940 | C=0.589, T=0.411 | 0.3962 | 7.733 | () | 1.00E-14 |
| DrnkWk | rs1260326 | Phosphatidylcholine diacyl C38:5 levels | 33414548 | chr2:27730940 | C=0.589, T=0.411 | 0.3984 | 7.089 | () | 1.00E-12 |
| DrnkWk | rs1260326 | Gamma glutamyl transpeptidase | 34594039 | chr2:27730940 | C=0.589, T=0.411 | NR | 0.0561 | 0.052-0.06 | 2.00E-191 |
| DrnkWk | rs1260326 | Glucose levels | 34594039 | chr2:27730940 | C=0.589, T=0.411 | NR | 0.0362 | 0.032-0.04 | 8.00E-70 |
| DrnkWk | rs1260326 | Triglyceride levels | 31217584 | chr2:27730940 | C=0.589, T=0.411 | NR | 0.07522435 | 0.066-0.084 | 6.00E-62 |
| DrnkWk | rs1260326 | Sex hormone-binding globulin levels | 32042192 | chr2:27730940 | C=0.589, T=0.411 | 0.607415 | 0.0343345 | 0.032-0.037 | 3.00E-134 |
| DrnkWk | rs1260326 | Low density lipoprotein cholesterol levels | 31217584 | chr2:27730940 | C=0.589, T=0.411 | NR | 1.847823 | 1.17-2.52 | 8.00E-08 |
| DrnkWk | rs1260326 | Total cholesterol levels | 31217584 | chr2:27730940 | C=0.589, T=0.411 | NR | 3.845262 | 3.09-4.6 | 1.00E-23 |
| DrnkWk | rs1260326 | White blood cell count | 31217584 | chr2:27730940 | C=0.589, T=0.411 | NR | 0.01382172 | 0.0085-0.0192 | 4.00E-07 |
| DrnkWk | rs1260326 | Triglyceride levels | 33339817 | chr2:27730940 | C=0.589, T=0.411 | NR | 0.0917 | 0.084-0.099 | 2.00E-133 |
| DrnkWk | rs1260326 | Sex hormone-binding globulin levels | 32042192 | chr2:27730940 | C=0.589, T=0.411 | 0.605695 | 0.0380831 | 0.036-0.041 | 3.00E-219 |
| DrnkWk | rs1260326 | Type 2 diabetes | 29632382 | chr2:27730940 | C=0.589, T=0.411 | 0.63 | 1.06 | 1.05-1.08 | 5.00E-25 |
| DrnkWk | rs1260326 | Type 2 diabetes | 29632382 | chr2:27730940 | C=0.589, T=0.411 | 0.617 | 1.06 | 1.04-1.07 | 1.00E-15 |
| DrnkWk | rs1260326 | Type 2 diabetes (adjusted for BMI) | 29632382 | chr2:27730940 | C=0.589, T=0.411 | 0.63 | 1.06 | 1.04-1.07 | 3.00E-18 |
| DrnkWk | rs1260326 | Type 2 diabetes (adjusted for BMI) | 29632382 | chr2:27730940 | C=0.589, T=0.411 | 0.617 | 1.05 | 1.04-1.07 | 1.00E-14 |
| DrnkWk | rs1260326 | Estradiol levels | 32042192 | chr2:27730940 | C=0.589, T=0.411 | 0.605566 | 0.00556675 | 0.0039-0.0073 | 1.00E-10 |
| DrnkWk | rs1260326 | Sex hormone-binding globulin levels adjusted for BMI | 32042192 | chr2:27730940 | C=0.589, T=0.411 | 0.607415 | 0.0351659 | 0.033-0.038 | 2.00E-188 |
| DrnkWk | rs1260326 | Sex hormone-binding globulin levels adjusted for BMI | 32042192 | chr2:27730940 | C=0.589, T=0.411 | 0.605695 | 0.0411304 | 0.039-0.043 | 1.00E-298 |
| DrnkWk | rs1260326 | Alcohol use disorder | 30940813 | chr2:27730940 | C=0.589, T=0.411 | 0.591 | 1.0749 | 1.06-1.09 | 1.00E-16 |
| DrnkWk | rs1260326 | Alcohol use disorder (consumption score) | 30940813 | chr2:27730940 | C=0.589, T=0.411 | 0.652 | 8.22 | NA | 2.00E-16 |
| DrnkWk | rs1260326 | Alcohol use disorder (consumption score) | 30940813 | chr2:27730940 | C=0.589, T=0.411 | 0.591 | 0.04477 | 0.034-0.055 | 2.00E-16 |
| DrnkWk | rs1260326 | Alcohol use disorder | 30940813 | chr2:27730940 | C=0.589, T=0.411 | 0.651 | 7.33 | NA | 2.00E-13 |
| DrnkWk | rs1260326 | Urolithiasis | 30975718 | chr2:27730940 | C=0.589, T=0.411 | 0.556 | 1.1 | NA | 5.00E-11 |
| DrnkWk | rs1260326 | Triglycerides | 30275531 | chr2:27730940 | C=0.589, T=0.411 | 0.379 | 0.1118 | 0.11-0.12-1 | 1.00E-300 |
| DrnkWk | rs1260326 | Body mass index | 30108127 | chr2:27730940 | C=0.589, T=0.411 | NR | 0.012 | NA | 5.00E-06 |
| DrnkWk | rs1260326 | Total cholesterol levels | 30275531 | chr2:27730940 | C=0.589, T=0.411 | 0.3798 | 0.0748 | 0.069-0.08-1 | 3.00E-160 |
| DrnkWk | rs1260326 | Reticulocyte count | 32888494 | chr2:27730940 | C=0.589, T=0.411 | 0.605475 | 0.033250246 | 0.029-0.038 | 7.00E-48 |
| DrnkWk | rs1260326 | Bitter alcoholic beverage consumption | 31046077 | chr2:27730940 | C=0.589, T=0.411 | 0.6034 | 0.0128 | 0.011-0.015 | 2.00E-35 |
| DrnkWk | rs1260326 | Reticulocyte fraction of red cells | 32888494 | chr2:27730940 | C=0.589, T=0.411 | 0.605469 | 0.03778806 | 0.033-0.042 | 2.00E-61 |
| DrnkWk | rs1260326 | Coffee consumption | 31046077 | chr2:27730940 | C=0.589, T=0.411 | 0.6047 | 0.0136 | 0.011-0.017 | 1.00E-19 |
| DrnkWk | rs1260326 | Bitter beverage consumption | 31046077 | chr2:27730940 | C=0.589, T=0.411 | 0.6058 | 0.0111 | 0.008-0.0142 | 1.00E-11 |
| DrnkWk | rs1260326 | Alcohol consumption (drinks per week) | 30643251 | chr2:27730940 | C=0.589, T=0.411 | 0.601 | 0.020889835 | 0.018-0.024 | 8.00E-45 |
| DrnkWk | rs1260326 | White blood cell count | 32888494 | chr2:27730940 | C=0.589, T=0.411 | 0.605529 | 0.034130413 | 0.03-0.039 | 6.00E-52 |
| DrnkWk | rs1260326 | Fasting blood glucose | 31217584 | chr2:27730940 | C=0.589, T=0.411 | NR | 0.07841215 | 0.059-0.098 | 1.00E-15 |
| DrnkWk | rs1260326 | Alcohol consumption (drinks per week) (MTAG) | 30643251 | chr2:27730940 | C=0.589, T=0.411 | 0.601 | 0.0187927 | 0.016-0.021 | 3.00E-44 |
| DrnkWk | rs1260326 | Triglyceride levels | 30911093 | chr2:27730940 | C=0.589, T=0.411 | NR | NA | NA | 1.00E-06 |
| DrnkWk | rs1260326 | Aspartate aminotransferase levels | 35810165 | chr2:27730940 | C=0.589, T=0.411 | NR | NA | NA | 5.00E-21 |
| DrnkWk | rs1260326 | Familial combined hyperlipidemia defined by Consensus criteria | 34906840 | chr2:27730940 | C=0.589, T=0.411 | 0.393515 | 0.155251 | 0.14-0.17 | 2.00E-88 |
| DrnkWk | rs1260326 | Familial combined hyperlipidemia defined by Mexico criteria | 34906840 | chr2:27730940 | C=0.589, T=0.411 | 0.393515 | 0.277526 | 0.24-0.32 | 1.00E-43 |
| DrnkWk | rs1260326 | Skeletal muscle mass | 35121771 | chr2:27730940 | C=0.589, T=0.411 | 0.44636 | 0.229999 | NA | 3.00E-07 |
| DrnkWk | rs1260326 | Familial combined hyperlipidemia defined by Dutch criteria | 34906840 | chr2:27730940 | C=0.589, T=0.411 | 0.393515 | 0.228516 | 0.21-0.25 | 4.00E-92 |
| DrnkWk | rs1260326 | Familial combined hyperlipidemia defined by Brunzell criteria | 34906840 | chr2:27730940 | C=0.589, T=0.411 | 0.393515 | 0.306791 | 0.26-0.35 | 2.00E-40 |
| DrnkWk | rs1260326 | Familial combined hyperlipidemia defined by Goldstein criteria | 34906840 | chr2:27730940 | C=0.589, T=0.411 | 0.393515 | 0.297168 | 0.23-0.36 | 9.00E-18 |
| DrnkWk | rs1260326 | Dyslipidemia | 33893285 | chr2:27730940 | C=0.589, T=0.411 | 0.424 | 0.92 | NA | 2.00E-11 |
| DrnkWk | rs1260326 | C-reactive protein levels | 35459240 | chr2:27730940 | C=0.589, T=0.411 | NR | 0.076 | 0.072-0.08 | 3.00E-303 |
| DrnkWk | rs1260326 | Apolipoprotein A1 levels | 35213538 | chr2:27730940 | C=0.589, T=0.411 | 0.396018 | 0.0394151 | 0.032-0.047 | 2.00E-24 |
| DrnkWk | rs1260326 | Total cholines levels | 35213538 | chr2:27730940 | C=0.589, T=0.411 | 0.395992 | 0.0645994 | 0.057-0.072 | 9.00E-60 |
| DrnkWk | rs1260326 | Neutrophil count | 34469753 | chr2:27730940 | C=0.589, T=0.411 | 0.395 | NA | NA | 2.00E-28 |
| DrnkWk | rs1260326 | Fasting insulin | 34059833 | chr2:27730940 | C=0.589, T=0.411 | 0.3402 | 0.0297 | 0.017-0.043 | 9.00E-06 |
| DrnkWk | rs1260326 | Fasting glucose | 34059833 | chr2:27730940 | C=0.589, T=0.411 | 0.2392 | 0.0847 | 0.058-0.111 | 3.00E-10 |
| DrnkWk | rs1260326 | Fasting insulin | 34059833 | chr2:27730940 | C=0.589, T=0.411 | 0.3928 | 0.0231 | 0.019-0.027 | 8.00E-38 |
| DrnkWk | rs1260326 | Lymphocyte count | 34469753 | chr2:27730940 | C=0.589, T=0.411 | 0.395 | NA | NA | 2.00E-17 |
| DrnkWk | rs1260326 | Phospholipids to total lipids ratio in large VLDL | 35213538 | chr2:27730940 | C=0.589, T=0.411 | 0.396021 | 0.0618646 | 0.054-0.07 | 1.00E-51 |
| DrnkWk | rs1260326 | Testosterone levels | 35192695 | chr2:27730940 | C=0.589, T=0.411 | 0.387982 | 0.0293367 | 0.023-0.036 | 5.00E-18 |
| DrnkWk | rs1260326 | Triglyceride levels in large VLDL | 35213538 | chr2:27730940 | C=0.589, T=0.411 | 0.396018 | 0.0900044 | 0.082-0.098 | 3.00E-109 |
| DrnkWk | rs1260326 | Ratio of linoleic acid to total fatty acids | 35213538 | chr2:27730940 | C=0.589, T=0.411 | 0.395992 | 0.0815217 | 0.074-0.089 | 2.00E-89 |
| DrnkWk | rs1260326 | Cholesteryl ester levels in LDL | 35213538 | chr2:27730940 | C=0.589, T=0.411 | 0.396018 | 0.049884 | 0.042-0.058 | 2.00E-32 |
| DrnkWk | rs1260326 | Total lipid levels in LDL | 35213538 | chr2:27730940 | C=0.589, T=0.411 | 0.396018 | 0.0458029 | 0.038-0.054 | 1.00E-27 |
| DrnkWk | rs1260326 | Sex hormone-binding globulin levels | 35192695 | chr2:27730940 | C=0.589, T=0.411 | 0.387982 | 0.0743767 | 0.068-0.081 | 2.00E-115 |
| DrnkWk | rs1260326 | Phospholipid levels in LDL | 35213538 | chr2:27730940 | C=0.589, T=0.411 | 0.396018 | 0.0385077 | 0.03-0.047 | 6.00E-20 |
| DrnkWk | rs1260326 | Triglyceride levels in LDL | 35213538 | chr2:27730940 | C=0.589, T=0.411 | 0.396018 | 0.104037 | 0.096-0.112 | 2.00E-140 |
| DrnkWk | rs1260326 | Average diameter for LDL particles | 35213538 | chr2:27730940 | C=0.589, T=0.411 | 0.396018 | 0.0387976 | 0.031-0.047 | 4.00E-21 |
| DrnkWk | rs1260326 | Cholesterol to total lipids ratio in medium HDL | 35213538 | chr2:27730940 | C=0.589, T=0.411 | 0.396018 | 0.0562519 | 0.048-0.064 | 1.00E-44 |
| DrnkWk | rs1260326 | Cholesteryl ester levels in medium HDL | 35213538 | chr2:27730940 | C=0.589, T=0.411 | 0.396018 | 0.0234449 | 0.016-0.031 | 2.00E-09 |
| DrnkWk | rs1260326 | Cholesteryl esters to total lipids ratio in medium HDL | 35213538 | chr2:27730940 | C=0.589, T=0.411 | 0.396018 | 0.062678 | 0.055-0.071 | 2.00E-53 |
| DrnkWk | rs1260326 | Testosterone levels in postmenopausal women | 35192695 | chr2:27730940 | C=0.589, T=0.411 | 0.387982 | 0.0253646 | 0.016-0.035 | 4.00E-07 |
| DrnkWk | rs1260326 | Sex hormone-binding globulin levels in postmenopausal women | 35192695 | chr2:27730940 | C=0.589, T=0.411 | 0.387982 | 0.0788036 | 0.069-0.088 | 1.00E-61 |
| DrnkWk | rs1260326 | Triglyceride to HDL cholesterol ratio | 38200128 | chr2:27730940 | C=0.589, T=0.411 | 0.395 | 0.1076 | 0.1-0.11 | 1.00E-246 |
| DrnkWk | rs1260326 | Free cholesterol levels in medium HDL | 35213538 | chr2:27730940 | C=0.589, T=0.411 | 0.396018 | 0.0328273 | 0.025-0.04 | 1.00E-17 |
| DrnkWk | rs1260326 | Triglyceride to HDL cholesterol ratio | 38200128 | chr2:27730940 | C=0.589, T=0.411 | 0.3958 | 0.0938 | 0.087-0.101 | 7.00E-130 |
| DrnkWk | rs1260326 | Sex hormone-binding globulin levels in premenopausal women | 35192695 | chr2:27730940 | C=0.589, T=0.411 | 0.387982 | 0.0787931 | 0.065-0.092 | 4.00E-30 |
| DrnkWk | rs1260326 | Triglyceride levels in HDL | 35213538 | chr2:27730940 | C=0.589, T=0.411 | 0.396018 | 0.0975113 | 0.089-0.106 | 1.00E-123 |
| DrnkWk | rs1260326 | Free cholesterol to total lipids ratio in IDL | 35213538 | chr2:27730940 | C=0.589, T=0.411 | 0.396018 | 0.054334 | 0.046-0.063 | 2.00E-38 |
| DrnkWk | rs1260326 | Cholesteryl esters to total lipids ratio in IDL | 35213538 | chr2:27730940 | C=0.589, T=0.411 | 0.396018 | 0.0508185 | 0.043-0.059 | 4.00E-35 |
| DrnkWk | rs1260326 | Triglycerides to total lipids ratio in IDL | 35213538 | chr2:27730940 | C=0.589, T=0.411 | 0.396018 | 0.0777125 | 0.07-0.086 | 3.00E-82 |
| DrnkWk | rs1260326 | Cholesteryl esters to total lipids ratio in large HDL | 35213538 | chr2:27730940 | C=0.589, T=0.411 | 0.396018 | 0.0729622 | 0.065-0.081 | 7.00E-74 |
| DrnkWk | rs1260326 | Triglyceride levels in large HDL | 35213538 | chr2:27730940 | C=0.589, T=0.411 | 0.396018 | 0.0585941 | 0.051-0.066 | 2.00E-48 |
| DrnkWk | rs1260326 | Phospholipids to total lipids ratio in large HDL | 35213538 | chr2:27730940 | C=0.589, T=0.411 | 0.396018 | 0.0626845 | 0.055-0.071 | 2.00E-53 |
| DrnkWk | rs1260326 | Cholesteryl ester levels in large LDL | 35213538 | chr2:27730940 | C=0.589, T=0.411 | 0.396018 | 0.0399934 | 0.032-0.048 | 1.00E-21 |
| DrnkWk | rs1260326 | Cholesterol to total lipids ratio in large LDL | 35213538 | chr2:27730940 | C=0.589, T=0.411 | 0.396018 | 0.0389029 | 0.031-0.047 | 2.00E-21 |
| DrnkWk | rs1260326 | Triglyceride levels in large LDL | 35213538 | chr2:27730940 | C=0.589, T=0.411 | 0.396018 | 0.0995963 | 0.092-0.108 | 1.00E-128 |
| DrnkWk | rs1260326 | Triglycerides to total lipids ratio in large LDL | 35213538 | chr2:27730940 | C=0.589, T=0.411 | 0.396018 | 0.065774 | 0.058-0.074 | 5.00E-58 |
| DrnkWk | rs1260326 | Phospholipids to total lipids ratio in large LDL | 35213538 | chr2:27730940 | C=0.589, T=0.411 | 0.396018 | 0.0603469 | 0.052-0.069 | 1.00E-46 |
| DrnkWk | rs1260326 | Total lipid levels in large VLDL | 35213538 | chr2:27730940 | C=0.589, T=0.411 | 0.396018 | 0.0891439 | 0.081-0.097 | 2.00E-106 |
| DrnkWk | rs1260326 | Total lipid levels in medium HDL | 35213538 | chr2:27730940 | C=0.589, T=0.411 | 0.396018 | 0.0461811 | 0.039-0.054 | 4.00E-32 |
| DrnkWk | rs1260326 | Total lipid levels in HDL | 35213538 | chr2:27730940 | C=0.589, T=0.411 | 0.396018 | 0.024187 | 0.017-0.032 | 2.00E-10 |
| DrnkWk | rs1260326 | Concentration of HDL particles | 35213538 | chr2:27730940 | C=0.589, T=0.411 | 0.396018 | 0.0535299 | 0.046-0.061 | 1.00E-41 |
| DrnkWk | rs1260326 | Average diameter for HDL particles | 35213538 | chr2:27730940 | C=0.589, T=0.411 | 0.396018 | 0.0378301 | 0.031-0.045 | 4.00E-24 |
| DrnkWk | rs1260326 | Cholesterol to total lipids ratio in IDL | 35213538 | chr2:27730940 | C=0.589, T=0.411 | 0.396018 | 0.0614012 | 0.053-0.069 | 9.00E-51 |
| DrnkWk | rs1260326 | Cholesterol levels in large HDL | 35213538 | chr2:27730940 | C=0.589, T=0.411 | 0.396018 | 0.0385761 | 0.031-0.046 | 3.00E-25 |
| DrnkWk | rs1260326 | Cholesterol to total lipids ratio in large HDL | 35213538 | chr2:27730940 | C=0.589, T=0.411 | 0.396018 | 0.0757245 | 0.068-0.083 | 2.00E-81 |
| DrnkWk | rs1260326 | Free cholesterol to total lipids ratio in large HDL | 35213538 | chr2:27730940 | C=0.589, T=0.411 | 0.396018 | 0.0535059 | 0.046-0.061 | 2.00E-42 |
| DrnkWk | rs1260326 | Total lipid levels in large HDL | 35213538 | chr2:27730940 | C=0.589, T=0.411 | 0.396018 | 0.0256137 | 0.018-0.033 | 4.00E-12 |
| DrnkWk | rs1260326 | Free cholesterol levels in large HDL | 35213538 | chr2:27730940 | C=0.589, T=0.411 | 0.396018 | 0.0320993 | 0.025-0.039 | 4.00E-18 |
| DrnkWk | rs1260326 | Triglycerides to total lipids ratio in large HDL | 35213538 | chr2:27730940 | C=0.589, T=0.411 | 0.396018 | 0.0714624 | 0.064-0.079 | 2.00E-72 |
| DrnkWk | rs1260326 | Cholesteryl esters to total lipids ratio in large LDL | 35213538 | chr2:27730940 | C=0.589, T=0.411 | 0.396018 | 0.0528011 | 0.045-0.061 | 9.00E-36 |
| DrnkWk | rs1260326 | Free cholesterol to total lipids ratio in large LDL | 35213538 | chr2:27730940 | C=0.589, T=0.411 | 0.396018 | 0.0939994 | 0.086-0.102 | 4.00E-122 |
| DrnkWk | rs1260326 | Cholesterol levels in large VLDL | 35213538 | chr2:27730940 | C=0.589, T=0.411 | 0.396018 | 0.0820347 | 0.074-0.09 | 3.00E-88 |
| DrnkWk | rs1260326 | Cholesteryl ester levels in large VLDL | 35213538 | chr2:27730940 | C=0.589, T=0.411 | 0.396018 | 0.0725852 | 0.064-0.081 | 4.00E-68 |
| DrnkWk | rs1260326 | Cholesterol to total lipids ratio in large VLDL | 35213538 | chr2:27730940 | C=0.589, T=0.411 | 0.396021 | 0.0522503 | 0.044-0.06 | 8.00E-37 |
| DrnkWk | rs1260326 | Concentration of large VLDL particles | 35213538 | chr2:27730940 | C=0.589, T=0.411 | 0.396018 | 0.0910186 | 0.083-0.099 | 4.00E-111 |
| DrnkWk | rs1260326 | Phospholipid levels in HDL | 35213538 | chr2:27730940 | C=0.589, T=0.411 | 0.396018 | 0.030729 | 0.023-0.038 | 7.00E-16 |
| DrnkWk | rs1260326 | Triglyceride levels in IDL | 35213538 | chr2:27730940 | C=0.589, T=0.411 | 0.396018 | 0.0939772 | 0.086-0.102 | 2.00E-115 |
| DrnkWk | rs1260326 | Cholesteryl ester levels in large HDL | 35213538 | chr2:27730940 | C=0.589, T=0.411 | 0.396018 | 0.0405179 | 0.033-0.048 | 2.00E-27 |
| DrnkWk | rs1260326 | Concentration of large HDL particles | 35213538 | chr2:27730940 | C=0.589, T=0.411 | 0.396018 | 0.0256281 | 0.018-0.033 | 4.00E-12 |
| DrnkWk | rs1260326 | Cholesterol levels in large LDL | 35213538 | chr2:27730940 | C=0.589, T=0.411 | 0.396018 | 0.031895 | 0.024-0.04 | 2.00E-14 |
| DrnkWk | rs1260326 | Total lipid levels in large LDL | 35213538 | chr2:27730940 | C=0.589, T=0.411 | 0.396018 | 0.0372393 | 0.029-0.045 | 5.00E-19 |
| DrnkWk | rs1260326 | Cholesteryl esters to total lipids ratio in large VLDL | 35213538 | chr2:27730940 | C=0.589, T=0.411 | 0.396021 | 0.0671407 | 0.059-0.075 | 4.00E-62 |
| DrnkWk | rs1260326 | Free cholesterol levels in large VLDL | 35213538 | chr2:27730940 | C=0.589, T=0.411 | 0.396018 | 0.0886548 | 0.081-0.097 | 4.00E-105 |
| DrnkWk | rs1260326 | Phospholipid levels in large VLDL | 35213538 | chr2:27730940 | C=0.589, T=0.411 | 0.396018 | 0.0895839 | 0.082-0.098 | 9.00E-108 |
| DrnkWk | rs1260326 | Phospholipids to total lipids ratio in medium HDL | 35213538 | chr2:27730940 | C=0.589, T=0.411 | 0.396018 | 0.0351748 | 0.027-0.043 | 2.00E-18 |
| DrnkWk | rs1260326 | Phospholipid levels in medium HDL | 35213538 | chr2:27730940 | C=0.589, T=0.411 | 0.396018 | 0.0535293 | 0.046-0.061 | 5.00E-42 |
| DrnkWk | rs1260326 | Testosterone levels | 35192695 | chr2:27730940 | C=0.589, T=0.411 | NR | 8.763 | NA | 2.00E-18 |
| DrnkWk | rs1260326 | Triglyceride levels in medium HDL | 35213538 | chr2:27730940 | C=0.589, T=0.411 | 0.396018 | 0.096479 | 0.088-0.105 | 3.00E-120 |
| DrnkWk | rs1260326 | Triglycerides to total lipids ratio in medium HDL | 35213538 | chr2:27730940 | C=0.589, T=0.411 | 0.396018 | 0.0670472 | 0.059-0.075 | 4.00E-62 |
| DrnkWk | rs1260326 | Triglyceride to HDL cholesterol ratio | 38200128 | chr2:27730940 | C=0.589, T=0.411 | 0.3943 | 0.0947 | 0.088-0.101 | 1.00E-143 |
| DrnkWk | rs1260326 | Sex hormone-binding globulin levels | 35192695 | chr2:27730940 | C=0.589, T=0.411 | NR | 23.157 | NA | 1.00E-118 |
| DrnkWk | rs1260326 | Cholesterol levels in medium LDL | 35213538 | chr2:27730940 | C=0.589, T=0.411 | 0.396018 | 0.0565467 | 0.048-0.065 | 8.00E-41 |
| DrnkWk | rs1260326 | Cholesteryl ester levels in medium LDL | 35213538 | chr2:27730940 | C=0.589, T=0.411 | 0.396018 | 0.0659301 | 0.058-0.074 | 6.00E-55 |
| DrnkWk | rs1260326 | Cholesterol to total lipids ratio in medium LDL | 35213538 | chr2:27730940 | C=0.589, T=0.411 | 0.396018 | 0.0230705 | 0.015-0.031 | 5.00E-08 |
| DrnkWk | rs1260326 | Cholesteryl esters to total lipids ratio in medium LDL | 35213538 | chr2:27730940 | C=0.589, T=0.411 | 0.396018 | 0.0694059 | 0.061-0.078 | 2.00E-61 |
| DrnkWk | rs1260326 | Free cholesterol to total lipids ratio in medium LDL | 35213538 | chr2:27730940 | C=0.589, T=0.411 | 0.396018 | 0.0913448 | 0.083-0.099 | 2.00E-114 |
| DrnkWk | rs1260326 | Total lipid levels in medium LDL | 35213538 | chr2:27730940 | C=0.589, T=0.411 | 0.396018 | 0.0610287 | 0.053-0.069 | 3.00E-47 |
| DrnkWk | rs1260326 | Concentration of medium LDL particles | 35213538 | chr2:27730940 | C=0.589, T=0.411 | 0.396018 | 0.0514519 | 0.043-0.06 | 5.00E-34 |
| DrnkWk | rs1260326 | Phospholipids to total lipids ratio in medium LDL | 35213538 | chr2:27730940 | C=0.589, T=0.411 | 0.396018 | 0.060574 | 0.052-0.069 | 3.00E-47 |
| DrnkWk | rs1260326 | Phospholipid levels in medium LDL | 35213538 | chr2:27730940 | C=0.589, T=0.411 | 0.396018 | 0.0553573 | 0.047-0.064 | 3.00E-39 |
| DrnkWk | rs1260326 | Triglyceride levels in medium LDL | 35213538 | chr2:27730940 | C=0.589, T=0.411 | 0.396018 | 0.108602 | 0.1-0.12 | 1.00E-153 |
| DrnkWk | rs1260326 | Triglycerides to total lipids ratio in medium LDL | 35213538 | chr2:27730940 | C=0.589, T=0.411 | 0.396018 | 0.0639998 | 0.056-0.072 | 4.00E-54 |
| DrnkWk | rs1260326 | Triglyceride to HDL cholesterol ratio | 38200128 | chr2:27730940 | C=0.589, T=0.411 | 0.265 | 0.116 | 0.083-0.149 | 8.00E-11 |
| DrnkWk | rs1260326 | Cholesterol to total lipids ratio in medium VLDL | 35213538 | chr2:27730940 | C=0.589, T=0.411 | 0.396018 | 0.0769926 | 0.069-0.085 | 4.00E-84 |
| DrnkWk | rs1260326 | Resting heart rate | 29769521 | chr2:27730940 | C=0.589, T=0.411 | 0.397939 | 0.422407 | 0.29-0.55 | 1.00E-10 |
| DrnkWk | rs1260326 | Branched-chain amino acid levels (Isoleucine) | 27898682 | chr2:27730940 | C=0.589, T=0.411 | 0.41 | 0.06 | 0.036-0.084 | 1.00E-09 |
| DrnkWk | rs1260326 | Branched-chain amino acid levels (Leucine) | 27898682 | chr2:27730940 | C=0.589, T=0.411 | NR | 0.049 | 0.023-0.075 | 2.00E-07 |
| DrnkWk | rs1260326 | Branched-chain amino acid levels (Valine) | 27898682 | chr2:27730940 | C=0.589, T=0.411 | NR | 0.05 | 0.024-0.076 | 9.00E-07 |
| DrnkWk | rs1260326 | Fat-free mass | 30593698 | chr2:27730940 | C=0.589, T=0.411 | NR | 0.1816 | 0.13-0.23 | 2.00E-13 |
| DrnkWk | rs1260326 | Fat-free mass | 30593698 | chr2:27730940 | C=0.589, T=0.411 | NR | 0.25305 | 0.18-0.32 | 1.00E-12 |
| DrnkWk | rs1260326 | Fat-free mass | 30593698 | chr2:27730940 | C=0.589, T=0.411 | NR | 0.21751 | 0.17-0.26 | 4.00E-22 |
| DrnkWk | rs1260326 | Alcohol consumption (drinks per week) | 30643258 | chr2:27730940 | C=0.589, T=0.411 | 0.3929 | 0.034923363 | 0.03-0.039 | 1.00E-52 |
| DrnkWk | rs1260326 | Urinary sodium excretion | 31409800 | chr2:27730940 | C=0.589, T=0.411 | 0.395664 | 0.0117743 | 0.0093-0.0142 | 1.00E-20 |
| DrnkWk | rs1260326 | Height | 30595370 | chr2:27730940 | C=0.589, T=0.411 | NR | NA | NA | 1.00E-36 |
| DrnkWk | rs1260326 | Metabolic syndrome | 31589552 | chr2:27730940 | C=0.589, T=0.411 | 0.392938 | 0.0536971 | 0.041-0.067 | 7.00E-16 |
| DrnkWk | rs1260326 | Height | 31562340 | chr2:27730940 | C=0.589, T=0.411 | NR | 0.02332736 | 0.018-0.029 | 2.00E-16 |
| DrnkWk | rs1260326 | LDL cholesterol levels | 30926973 | chr2:27730940 | C=0.589, T=0.411 | 0.4069 | 1.045 | 0.68-1.41 | 2.00E-08 |
| DrnkWk | rs1260326 | Triglyceride levels | 30926973 | chr2:27730940 | C=0.589, T=0.411 | 0.3666 | 0.0482 | 0.043-0.053 | 1.00E-84 |
| DrnkWk | rs1260326 | White blood cell count | 24777453 | chr2:27730940 | C=0.589, T=0.411 | 0.43 | 0.005 | 0.003-0.007 | 9.00E-08 |
| DrnkWk | rs1260326 | LDL cholesterol levels | 32203549 | chr2:27730940 | C=0.589, T=0.411 | 0.395558 | 0.0347351 | 0.031-0.039 | 6.00E-60 |
| DrnkWk | rs1260326 | Type 2 diabetes | 35551307 | chr2:27730940 | C=0.589, T=0.411 | 0.465 | 0.061 | 0.045-0.077 | 2.00E-13 |
| DrnkWk | rs1260326 | Type 2 diabetes | 35551307 | chr2:27730940 | C=0.589, T=0.411 | 0.607 | 0.064 | 0.05-0.078 | 2.00E-22 |
| DrnkWk | rs1260326 | Neutrophil count | 34594039 | chr2:27730940 | C=0.589, T=0.411 | NR | 0.032 | 0.028-0.036 | 6.00E-50 |
| DrnkWk | rs1260326 | Triacylglyceride levels | 35945198 | chr2:27730940 | C=0.589, T=0.411 | 0.74 | 0.15 | 0.13-0.17 | 3.00E-27 |
| DrnkWk | rs1260326 | Total cholesterol levels | 35945198 | chr2:27730940 | C=0.589, T=0.411 | 0.74 | 0.06 | 0.04-0.08 | 8.00E-07 |
| DrnkWk | rs1260326 | Blood urea nitrogen levels | 34272381 | chr2:27730940 | C=0.589, T=0.411 | 0.4185 | 0.0053 | 0.0043-0.0063 | 1.00E-26 |
| DrnkWk | rs1260326 | Platelet count | 34594039 | chr2:27730940 | C=0.589, T=0.411 | NR | 0.04 | 0.036-0.044 | 1.00E-95 |
| DrnkWk | rs1260326 | Estimated glomerular filtration rate (creatinine) | 34272381 | chr2:27730940 | C=0.589, T=0.411 | 0.607243484 | 0.00502647 | 0.0045-0.0055 | 5.00E-85 |
| DrnkWk | rs1260326 | Plasma mannonate* levels in chronic kidney disease | 37277652 | chr2:27730940 | C=0.589, T=0.411 | 0.58 | 0.081 | 0.059-0.103 | 8.00E-14 |
| DrnkWk | rs1260326 | Plasma mannose levels in chronic kidney disease | 37277652 | chr2:27730940 | C=0.589, T=0.411 | 0.58 | 0.104 | 0.08-0.128 | 6.00E-17 |
| DrnkWk | rs1260326 | Lymphocyte count | 34594039 | chr2:27730940 | C=0.589, T=0.411 | NR | 0.0216 | 0.017-0.026 | 4.00E-24 |
| DrnkWk | rs1260326 | Total cholesterol levels | 34594039 | chr2:27730940 | C=0.589, T=0.411 | NR | 0.0417 | 0.038-0.046 | 1.00E-98 |
| DrnkWk | rs1260326 | Triglycerides | 34594039 | chr2:27730940 | C=0.589, T=0.411 | NR | 0.0992 | 0.095-0.103 | 0 |
| DrnkWk | rs1260326 | Serum total protein levels | 34594039 | chr2:27730940 | C=0.589, T=0.411 | NR | 0.0414 | 0.037-0.046 | 9.00E-85 |
| DrnkWk | rs1260326 | Serum uric acid levels | 34594039 | chr2:27730940 | C=0.589, T=0.411 | NR | 0.0445 | 0.041-0.048 | 2.00E-136 |
| DrnkWk | rs1260326 | Mannose levels | 27073872 | chr2:27730940 | C=0.589, T=0.411 | NR | 0.3110107 | 0.23-0.4 | 5.00E-13 |
| DrnkWk | rs1260326 | White blood cell count | 34594039 | chr2:27730940 | C=0.589, T=0.411 | NR | 0.0289 | 0.025-0.033 | 4.00E-48 |
| DrnkWk | rs1260326 | Height | 34594039 | chr2:27730940 | C=0.589, T=0.411 | NR | 0.0157 | 0.013-0.018 | 7.00E-34 |
| DrnkWk | rs1260326 | lysoPhosphatidylcholine acyl C16:0 levels | 35888728 | chr2:27730940 | C=0.589, T=0.411 | NR | 0.1341 | NA | 4.00E-11 |
| DrnkWk | rs1260326 | lysoPhosphatidylcholine acyl C16:1 levels | 35888728 | chr2:27730940 | C=0.589, T=0.411 | NR | 0.1482 | NA | 2.00E-13 |
| DrnkWk | rs1260326 | lysoPhosphatidylcholine acyl C20:3 levels | 35888728 | chr2:27730940 | C=0.589, T=0.411 | NR | 0.1186 | NA | 4.00E-09 |
| DrnkWk | rs1260326 | Phosphatidylcholine diacyl C34:4 levels | 35888728 | chr2:27730940 | C=0.589, T=0.411 | NR | 0.1209 | NA | 3.00E-09 |
| DrnkWk | rs1260326 | Sterol ester (27:1/18:3) levels | 37907536 | chr2:27730940 | C=0.589, T=0.411 | 0.349122 | 0.105379 | 0.071-0.14 | 2.00E-09 |
| DrnkWk | rs1260326 | Low testosterone levels | 34337532 | chr2:27730940 | C=0.589, T=0.411 | 0.39 | 1.13 | NA | 1.00E-25 |
| DrnkWk | rs1260326 | Liver volume | 34128465 | chr2:27730940 | C=0.589, T=0.411 | NR | 0.0636 | NA | 5.00E-19 |
| DrnkWk | rs1260326 | Platelet count | 34469753 | chr2:27730940 | C=0.589, T=0.411 | 0.395 | NA | NA | 4.00E-43 |
| DrnkWk | rs1260326 | Two-hour glucose | 34059833 | chr2:27730940 | C=0.589, T=0.411 | 0.3957 | 0.0486 | 0.033-0.064 | 6.00E-12 |
| DrnkWk | rs1260326 | Fasting glucose | 34059833 | chr2:27730940 | C=0.589, T=0.411 | 0.3872 | 0.0282 | 0.025-0.032 | 4.00E-65 |
| DrnkWk | rs1260326 | Fasting glucose | 34059833 | chr2:27730940 | C=0.589, T=0.411 | 0.3371 | 0.0403 | 0.03-0.051 | 1.00E-11 |
| DrnkWk | rs1260326 | Triglyceride levels | 34074324 | chr2:27730940 | C=0.589, T=0.411 | 0.4 | 0.25 | 0.15-0.35 | 1.00E-07 |
| DrnkWk | rs1260326 | Liver enzyme levels (alkaline phosphatase) | 33972514 | chr2:27730940 | C=0.589, T=0.411 | 0.395387 | 0.00614907 | 0.0057-0.0066 | 1.00E-155 |
| DrnkWk | rs1260326 | Liver enzyme levels (gamma-glutamyl transferase) | 33972514 | chr2:27730940 | C=0.589, T=0.411 | 0.395393 | 0.014005 | 0.013-0.015 | 2.00E-162 |
| DrnkWk | rs1260326 | Estimated glomerular filtration rate (creatinine) | 34272381 | chr2:27730940 | C=0.589, T=0.411 | 0.6071 | 0.00390673 | 0.0033-0.0045 | 9.00E-39 |
| DrnkWk | rs1260326 | Estimated glomerular filtration rate (cystatin c) | 34272381 | chr2:27730940 | C=0.589, T=0.411 | 0.3949 | 0.004 | 0.003-0.005 | 3.00E-17 |
| DrnkWk | rs1260326 | Nonalcoholic fatty liver disease | 35124268 | chr2:27730940 | C=0.589, T=0.411 | NR | 0.19 | 0.13-0.25 | 7.00E-14 |
| DrnkWk | rs1260326 | Biological age (PhenoAge) | 34427645 | chr2:27730940 | C=0.589, T=0.411 | 0.494 | 0.174 | 0.13-0.22 | 3.00E-16 |
| DrnkWk | rs1260326 | Type 2 diabetes | 35551307 | chr2:27730940 | C=0.589, T=0.411 | NR | 1.06 | 1.05-1.07 | 2.00E-35 |
| DrnkWk | rs1260326 | Serum creatinine levels | 34594039 | chr2:27730940 | C=0.589, T=0.411 | NR | 0.0342 | 0.031-0.037 | 1.00E-97 |
| DrnkWk | rs1260326 | Medication use (drugs used in diabetes) | 34594039 | chr2:27730940 | C=0.589, T=0.411 | NR | 0.0673 | 0.052-0.082 | 8.00E-19 |
| DrnkWk | rs1260326 | Medication use (HMG CoA reductase inhibitors) | 34594039 | chr2:27730940 | C=0.589, T=0.411 | NR | 0.0642 | 0.055-0.074 | 1.00E-39 |
| DrnkWk | rs1260326 | Glycemic traits (pleiotropy) | 31021400 | chr2:27730940 | C=0.589, T=0.411 | NR | NA | NA | 6.00E-13 |
| DrnkWk | rs1260326 | Percent glycated albumin | 29844224 | chr2:27730940 | C=0.589, T=0.411 | 0.41 | 0.01 | 0.0061-0.0139 | 2.00E-08 |
| DrnkWk | rs1260326 | Urate levels | 31578528 | chr2:27730940 | C=0.589, T=0.411 | 0.5555 | 0.0496 | 0.041-0.059/ | 4.00E-27 |
| DrnkWk | rs1260326 | Gout | 31578528 | chr2:27730940 | C=0.589, T=0.411 | NR | NA | NA | 4.00E-41 |
| DrnkWk | rs1260326 | Urinary sodium to creatinine ratio | 30910378 | chr2:27730940 | C=0.589, T=0.411 | 0.6082 | 0.029 | 0.023-0.035 | 2.00E-21 |
| DrnkWk | rs1260326 | Serum uric acid levels | 30993211 | chr2:27730940 | C=0.589, T=0.411 | 0.5589 | 0.0357 | 0.028-0.044 | 8.00E-19 |
| DrnkWk | rs1260326 | Medication use (HMG CoA reductase inhibitors) | 31015401 | chr2:27730940 | C=0.589, T=0.411 | 0.395882 | 0.059920058 | 0.049-0.071 | 1.00E-25 |
| DrnkWk | rs1260326 | Urate levels | 31578528 | chr2:27730940 | C=0.589, T=0.411 | 0.3983 | 0.069602 | 0.062-0.077/ | 5.00E-69 |
| DrnkWk | rs1260326 | Urate levels | 31578528 | chr2:27730940 | C=0.589, T=0.411 | 0.5435 | 0.0765 | 0.067-0.086/ | 1.00E-51 |
| DrnkWk | rs1260326 | Fasting blood insulin adjusted for BMI | 25625282 | chr2:27730940 | C=0.589, T=0.411 | NR | 0.023 | 0.015-0.031 | 8.00E-11 |
| DrnkWk | rs1260326 | Fasting blood glucose adjusted for BMI | 25625282 | chr2:27730940 | C=0.589, T=0.411 | NR | 0.031 | 0.023-0.039 | 5.00E-18 |
| DrnkWk | rs1260326 | Serum uric acid levels | 30993211 | chr2:27730940 | C=0.589, T=0.411 | 0.4 | 0.055 | 0.047-0.063 | 3.00E-45 |
| DrnkWk | rs1260326 | Serum uric acid levels | 30993211 | chr2:27730940 | C=0.589, T=0.411 | 0.559 | 0.036 | 0.028-0.044 | 8.00E-19 |
| DrnkWk | rs1260326 | C-reactive protein levels | 30388399 | chr2:27730940 | C=0.589, T=0.411 | 0.39 | 0.073 | 0.065-0.081 | 3.00E-92 |
| DrnkWk | rs1260326 | C-reactive protein levels | 30388399 | chr2:27730940 | C=0.589, T=0.411 | NR | 0.09 | NA | 2.00E-78 |
| DrnkWk | rs1260326 | C-reactive protein levels | 30388399 | chr2:27730940 | C=0.589, T=0.411 | NR | 0.052 | NA | 8.00E-23 |
| DrnkWk | rs1260326 | C-reactive protein levels | 30388399 | chr2:27730940 | C=0.589, T=0.411 | 0.4 | 0.077 | 0.069-0.085 | 9.00E-86 |
| DrnkWk | rs1260326 | Urate levels | 31578528 | chr2:27730940 | C=0.589, T=0.411 | 0.5568 | 0.0526 | 0.041-0.064/ | 1.00E-19 |
| DrnkWk | rs1260326 | Urate levels | 31578528 | chr2:27730940 | C=0.589, T=0.411 | 0.4359 | 0.066 | 0.06-0.072/ | 3.00E-95 |
| DrnkWk | rs1260326 | Urate levels | 31578528 | chr2:27730940 | C=0.589, T=0.411 | 0.1435 | 0.089 | 0.054-0.124/ | 9.00E-07 |
| DrnkWk | rs1260326 | Triglyceride levels | 34887591 | chr2:27730940 | C=0.589, T=0.411 | 0.661261 | 0.118817 | 0.11-0.13 | 1.00E-64 |
| DrnkWk | rs1260326 | Low density lipoprotein cholesterol levels | 34887591 | chr2:27730940 | C=0.589, T=0.411 | NR | NA | NA | 5.00E-114 |
| DrnkWk | rs1260326 | Coagulation factor IX levels | 36168886 | chr2:27730940 | C=0.589, T=0.411 | 0.419591 | 0.197977 | 0.14-0.25 | 7.00E-13 |
| DrnkWk | rs1260326 | Fibroblast growth factor 21 levels | 36349687 | chr2:27730940 | C=0.589, T=0.411 | NR | 0.1952 | 0.14-0.25 | 8.00E-13 |
| DrnkWk | rs1260326 | Diacylglycerol (16:1_18:1) levels | 37907536 | chr2:27730940 | C=0.589, T=0.411 | 0.349122 | 0.11192 | 0.075-0.149 | 2.00E-09 |
| DrnkWk | rs1260326 | Diacylglycerol (18:1_18:1) levels | 37907536 | chr2:27730940 | C=0.589, T=0.411 | 0.349122 | 0.122667 | 0.088-0.158 | 6.00E-12 |
| DrnkWk | rs1260326 | Diacylglycerol (18:1_18:2) levels | 37907536 | chr2:27730940 | C=0.589, T=0.411 | 0.349122 | 0.128985 | 0.094-0.164 | 1.00E-12 |
| DrnkWk | rs1260326 | Phosphatidylcholine (16:0_0:0) levels | 37907536 | chr2:27730940 | C=0.589, T=0.411 | 0.349122 | 0.0990041 | 0.065-0.133 | 2.00E-08 |
| DrnkWk | rs1260326 | Phosphatidylcholine (16:0_16:1) levels | 37907536 | chr2:27730940 | C=0.589, T=0.411 | 0.349122 | 0.108205 | 0.074-0.142 | 6.00E-10 |
| DrnkWk | rs1260326 | Phosphatidylcholine (16:0_18:1) levels | 37907536 | chr2:27730940 | C=0.589, T=0.411 | 0.349122 | 0.101263 | 0.067-0.135 | 7.00E-09 |
| DrnkWk | rs1260326 | Phosphatidylcholine (16:0_18:3) levels | 37907536 | chr2:27730940 | C=0.589, T=0.411 | 0.349122 | 0.115375 | 0.081-0.15 | 4.00E-11 |
| DrnkWk | rs1260326 | Phosphatidylcholine (16:0_20:2) levels | 37907536 | chr2:27730940 | C=0.589, T=0.411 | 0.349122 | 0.0971522 | 0.063-0.131 | 3.00E-08 |
| DrnkWk | rs1260326 | Phosphatidylcholine (16:1_18:0) levels | 37907536 | chr2:27730940 | C=0.589, T=0.411 | 0.349122 | 0.107928 | 0.072-0.144 | 4.00E-09 |
| DrnkWk | rs1260326 | Phosphatidylcholine (16:1_18:1) levels | 37907536 | chr2:27730940 | C=0.589, T=0.411 | 0.349122 | 0.110177 | 0.076-0.144 | 3.00E-10 |
| DrnkWk | rs1260326 | Phosphatidylcholine (16:1_18:2) levels | 37907536 | chr2:27730940 | C=0.589, T=0.411 | 0.349122 | 0.0984695 | 0.064-0.133 | 2.00E-08 |
| DrnkWk | rs1260326 | Phosphatidylethanolamine (18:2_0:0) levels | 37907536 | chr2:27730940 | C=0.589, T=0.411 | 0.349122 | 0.114725 | 0.08-0.15 | 1.00E-10 |
| DrnkWk | rs1260326 | Phosphatidylcholine (14:0_18:1) levels | 37907536 | chr2:27730940 | C=0.589, T=0.411 | 0.349122 | 0.105277 | 0.071-0.14 | 2.00E-09 |
| DrnkWk | rs1260326 | Ceramide (d40:1) levels | 37907536 | chr2:27730940 | C=0.589, T=0.411 | 0.349122 | 0.0994691 | 0.065-0.134 | 1.00E-08 |
| DrnkWk | rs1260326 | Phosphatidylcholine (18:0_22:5) levels | 37907536 | chr2:27730940 | C=0.589, T=0.411 | 0.349122 | 0.101199 | 0.066-0.136 | 2.00E-08 |
| DrnkWk | rs1260326 | Phosphatidylinositol (16:0_20:4) levels | 37907536 | chr2:27730940 | C=0.589, T=0.411 | 0.349122 | 0.109504 | 0.07-0.149 | 4.00E-08 |
| DrnkWk | rs1260326 | Phosphatidylethanolamine (16:0_18:2) levels | 37907536 | chr2:27730940 | C=0.589, T=0.411 | 0.349122 | 0.116253 | 0.077-0.155 | 6.00E-09 |
| DrnkWk | rs1260326 | Phosphatidylethanolamine (18:0_18:2) levels | 37907536 | chr2:27730940 | C=0.589, T=0.411 | 0.349122 | 0.121988 | 0.088-0.156 | 3.00E-12 |
| DrnkWk | rs1260326 | Phosphatidylinositol (18:0_20:4) levels | 37907536 | chr2:27730940 | C=0.589, T=0.411 | 0.349122 | 0.096388 | 0.062-0.13 | 3.00E-08 |
| DrnkWk | rs1260326 | Triacylglycerol (46:2) levels | 37907536 | chr2:27730940 | C=0.589, T=0.411 | 0.349122 | 0.113769 | 0.075-0.153 | 1.00E-08 |
| DrnkWk | rs1260326 | Triacylglycerol (48:1) levels | 37907536 | chr2:27730940 | C=0.589, T=0.411 | 0.349122 | 0.118511 | 0.084-0.153 | 2.00E-11 |
| DrnkWk | rs1260326 | Triacylglycerol (48:2) levels | 37907536 | chr2:27730940 | C=0.589, T=0.411 | 0.349122 | 0.137885 | 0.1-0.17 | 4.00E-15 |
| DrnkWk | rs1260326 | Triacylglycerol (48:3) levels | 37907536 | chr2:27730940 | C=0.589, T=0.411 | 0.349122 | 0.14156 | 0.11-0.18 | 2.00E-15 |
| DrnkWk | rs1260326 | Triacylglycerol (49:2) levels | 37907536 | chr2:27730940 | C=0.589, T=0.411 | 0.349122 | 0.120386 | 0.083-0.158 | 2.00E-10 |
| DrnkWk | rs1260326 | Triacylglycerol (50:1) levels | 37907536 | chr2:27730940 | C=0.589, T=0.411 | 0.349122 | 0.109326 | 0.075-0.144 | 4.00E-10 |
| DrnkWk | rs1260326 | Triacylglycerol (50:2) levels | 37907536 | chr2:27730940 | C=0.589, T=0.411 | 0.349122 | 0.139121 | 0.11-0.17 | 2.00E-15 |
| DrnkWk | rs1260326 | Triacylglycerol (50:3) levels | 37907536 | chr2:27730940 | C=0.589, T=0.411 | 0.349122 | 0.159151 | 0.13-0.19 | 7.00E-20 |
| DrnkWk | rs1260326 | Triacylglycerol (50:4) levels | 37907536 | chr2:27730940 | C=0.589, T=0.411 | 0.349122 | 0.168895 | 0.13-0.2 | 4.00E-22 |
| DrnkWk | rs1260326 | Triacylglycerol (50:5) levels | 37907536 | chr2:27730940 | C=0.589, T=0.411 | 0.349122 | 0.156758 | 0.12-0.2 | 3.00E-15 |
| DrnkWk | rs1260326 | Triacylglycerol (52:5) levels | 37907536 | chr2:27730940 | C=0.589, T=0.411 | 0.349122 | 0.153998 | 0.12-0.19 | 1.00E-18 |
| DrnkWk | rs1260326 | Triacylglycerol (52:6) levels | 37907536 | chr2:27730940 | C=0.589, T=0.411 | 0.349122 | 0.132332 | 0.097-0.168 | 4.00E-13 |
| DrnkWk | rs1260326 | Triacylglycerol (53:2) levels | 37907536 | chr2:27730940 | C=0.589, T=0.411 | 0.349122 | 0.118757 | 0.084-0.153 | 2.00E-11 |
| DrnkWk | rs1260326 | Triacylglycerol (53:3) levels | 37907536 | chr2:27730940 | C=0.589, T=0.411 | 0.349122 | 0.137329 | 0.1-0.17 | 4.00E-15 |
| DrnkWk | rs1260326 | Triacylglycerol (53:4) levels | 37907536 | chr2:27730940 | C=0.589, T=0.411 | 0.349122 | 0.135867 | 0.1-0.17 | 1.00E-13 |
| DrnkWk | rs1260326 | Triacylglycerol (51:2) levels | 37907536 | chr2:27730940 | C=0.589, T=0.411 | 0.349122 | 0.127915 | 0.094-0.162 | 2.00E-13 |
| DrnkWk | rs1260326 | Triacylglycerol (51:3) levels | 37907536 | chr2:27730940 | C=0.589, T=0.411 | 0.349122 | 0.13791 | 0.1-0.17 | 3.00E-15 |
| DrnkWk | rs1260326 | Triacylglycerol (51:4) levels | 37907536 | chr2:27730940 | C=0.589, T=0.411 | 0.349122 | 0.134885 | 0.095-0.175 | 3.00E-11 |
| DrnkWk | rs1260326 | Triacylglycerol (52:2) levels | 37907536 | chr2:27730940 | C=0.589, T=0.411 | 0.349122 | 0.121972 | 0.088-0.156 | 3.00E-12 |
| DrnkWk | rs1260326 | Triacylglycerol (52:3) levels | 37907536 | chr2:27730940 | C=0.589, T=0.411 | 0.349122 | 0.1368 | 0.1-0.17 | 4.00E-15 |
| DrnkWk | rs1260326 | Triacylglycerol (52:4) levels | 37907536 | chr2:27730940 | C=0.589, T=0.411 | 0.349122 | 0.138643 | 0.1-0.17 | 2.00E-15 |
| DrnkWk | rs1260326 | Triacylglycerol (54:4) levels | 37907536 | chr2:27730940 | C=0.589, T=0.411 | 0.349122 | 0.104794 | 0.071-0.139 | 2.00E-09 |
| DrnkWk | rs1260326 | Triacylglycerol (54:5) levels | 37907536 | chr2:27730940 | C=0.589, T=0.411 | 0.349122 | 0.121773 | 0.088-0.156 | 3.00E-12 |
| DrnkWk | rs1260326 | Triacylglycerol (54:6) levels | 37907536 | chr2:27730940 | C=0.589, T=0.411 | 0.349122 | 0.112094 | 0.078-0.147 | 2.00E-10 |
| DrnkWk | rs1260326 | Triacylglycerol (54:7) levels | 37907536 | chr2:27730940 | C=0.589, T=0.411 | 0.349122 | 0.102768 | 0.068-0.138 | 9.00E-09 |
| DrnkWk | rs1260326 | Triacylglycerol (56:6) levels | 37907536 | chr2:27730940 | C=0.589, T=0.411 | 0.349122 | 0.117623 | 0.083-0.152 | 2.00E-11 |
| DrnkWk | rs1260326 | Triacylglycerol (56:7) levels | 37907536 | chr2:27730940 | C=0.589, T=0.411 | 0.349122 | 0.0986077 | 0.064-0.133 | 2.00E-08 |
| DrnkWk | rs1260326 | Triacylglycerol (58:7) levels | 37907536 | chr2:27730940 | C=0.589, T=0.411 | 0.349122 | 0.119307 | 0.08-0.159 | 3.00E-09 |
| DrnkWk | rs1260326 | Low density lipoprotein cholesterol levels | 34887591 | chr2:27730940 | C=0.589, T=0.411 | 0.609624 | 0.0306622 | 0.028-0.033 | 9.00E-78 |
| DrnkWk | rs1260326 | Triglyceride levels | 34887591 | chr2:27730940 | C=0.589, T=0.411 | 0.752767 | 0.123606 | 0.11-0.14 | 4.00E-48 |
| DrnkWk | rs1260326 | Glucokinase regulatory protein levels | 36168886 | chr2:27730940 | C=0.589, T=0.411 | 0.419591 | 0.223161 | 0.17-0.28 | 2.00E-16 |
| DrnkWk | rs1260326 | Coagulation factor IXab levels | 36168886 | chr2:27730940 | C=0.589, T=0.411 | 0.419591 | 0.184923 | 0.13-0.24 | 2.00E-11 |
| DrnkWk | rs1260326 | Gout | 36281732 | chr2:27730940 | C=0.589, T=0.411 | 0.388 | 1.24 | 1.2-1.28 | 3.00E-36 |
| DrnkWk | rs1260326 | Albumin levels | 34321204 | chr2:27730940 | C=0.589, T=0.411 | 0.61 | 0.15 | 0.12-0.18/ | 2.00E-24 |
| DrnkWk | rs1260326 | Albumin levels | 34321204 | chr2:27730940 | C=0.589, T=0.411 | 0.61 | 0.14 | 0.12-0.16/ | 5.00E-28 |
| DrnkWk | rs1260326 | Sex hormone-binding globulin levels | 34321204 | chr2:27730940 | C=0.589, T=0.411 | 0.61 | 1.76 | 1.44-2.08/ | 2.00E-26 |
| DrnkWk | rs1260326 | Sex hormone-binding globulin levels | 34321204 | chr2:27730940 | C=0.589, T=0.411 | 0.61 | 1.82 | 1.54-2.1/ | 5.00E-38 |
| DrnkWk | rs1260326 | Total testosterone levels | 34321204 | chr2:27730940 | C=0.589, T=0.411 | 0.61 | 0.02 | 0.014-0.026/ | 4.00E-10 |
| DrnkWk | rs1260326 | Alcohol consumption (drinks per month) (UKB data field 1578, 4424) | 32193382 | chr2:27730940 | C=0.589, T=0.411 | 0.39494 | 0.0361951 | 0.032-0.04 | 3.00E-64 |
| DrnkWk | rs1260326 | Lactate levels | 35347128 | chr2:27730940 | C=0.589, T=0.411 | NR | 0.14 | NA | 4.00E-13 |
| DrnkWk | rs1260326 | Mannose levels | 35347128 | chr2:27730940 | C=0.589, T=0.411 | NR | 0.49 | NA | 6.00E-149 |
| DrnkWk | rs1260326 | Pyruvate levels | 35347128 | chr2:27730940 | C=0.589, T=0.411 | NR | 0.14 | NA | 9.00E-14 |
[truncated: 329,170 more chars]
