# Supplementary material for: Causal associations between lifestyle factors and hemorrhoidal disease: Insights from Mendelian randomization analysis
Source: Medicine (Baltimore). 2026 May 22;105(21):e48945. doi: 10.1097/MD.0000000000048945 (PMC13200937; doi:10.1097/MD.0000000000048945)
Supplement: Supplementary file 2 [file medi-105-e48945-s002.docx]

| Supplementary Table 4.IVs for initial analysis | | | | | | | | | | | | |  |  |  |  |  |  |
| --- | --- | --- | --- | --- | --- | --- | --- | --- | --- | --- | --- | --- | --- | --- | --- | --- | --- | --- |
| **Expose** | **SNP** | **Chromosome** | **Position** | **Exposure** | | | | | | | **Outcome** | | | | | | **R2** | **F** |
|  |  |  |  | **Effect allele** | **Other allele** | **EAF** | **β** | **SE** | **P-Value** | **Samplesize** | **Effect allele** | **Other allele** | **EAF** | **β** | **SE** | **P-Value** |  |  |
| LST | rs10041724 | 5 | 124273520 | T | C | 0.8071 | 0.0249 | 0.0044 | 1.51E-08 | 525491 | T | C | 0.802 | -0.0119 | 0.0049 | 0.0157699 | 6.09399E-05 | 32.02518803 |
| LST | rs10059100 | 5 | 120100784 | A | G | **0.6074** | -0.0215 | 0.0039 | 4.68E-08 | 468924 | A | G | 0.6172 | 0.0026 | 0.0041 | 0.5254 | 6.48063E-05 | 30.39106039 |
| LST | rs1017550 | 10 | 63587683 | A | G | 0.5864 | 0.0196 | 0.0035 | 2.85E-08 | 525491 | A | G | 0.5898 | 0.0042 | 0.004 | 0.2956 | 5.9674E-05 | 31.35988064 |
| LST | rs10189857 | 2 | 60713235 | A | G | 0.5653 | -0.0274 | 0.0035 | 7.80E-15 | 525491 | A | G | 0.573 | 0.0131 | 0.004 | 0.000992499 | 0.000116614 | 61.28629736 |
| LST | rs10222987 | 4 | 185948359 | A | G | 0.6355 | -0.0209 | 0.0036 | 9.02E-09 | 523875 | A | G | 0.6132 | -0.0015 | 0.0041 | 0.704399 | 6.43327E-05 | 33.70434663 |
| LST | rs10253861 | 7 | 8110475 | A | G | 0.5514 | -0.0198 | 0.0035 | 1.48E-08 | 525489 | A | G | 0.5554 | -0.0044 | 0.004 | 0.2666 | 6.08982E-05 | 32.0031435 |
| LST | rs10400776 | 14 | 97326366 | A | C | 0.2593 | -0.0259 | 0.0044 | 3.45E-09 | 468924 | A | C | 0.2527 | -0.0062 | 0.0045 | 0.1701 | 7.38856E-05 | 34.64912908 |
| LST | rs10765775 | 11 | 95656362 | A | G | 0.3915 | -0.0223 | 0.0039 | 9.80E-09 | 480972 | A | G | 0.4003 | 9.00E-04 | 0.004 | 0.8141 | 6.79722E-05 | 32.69480159 |
| LST | rs10772643 | 12 | 13415288 | T | C | 0.8923 | -0.0385 | 0.0062 | 5.88E-10 | 468924 | T | C | 0.8913 | -0.0039 | 0.0063 | 0.536801 | 8.22242E-05 | 38.55992919 |
| LST | rs10792966 | 11 | 71544105 | C | G | 0.6876 | 0.0237 | 0.0041 | 1.06E-08 | 468924 | C | G | 0.6834 | -0.0158 | 0.0082 | 0.0535599 | 7.12518E-05 | 33.41389675 |
| LST | rs10889193 | 1 | 61112137 | A | C | 0.5547 | 0.0243 | 0.0039 | 4.94E-10 | 469809 | A | C | 0.5444 | 6.00E-04 | 0.0039 | 0.8821 | 8.26278E-05 | 38.82231994 |
| LST | rs11074658 | 16 | 10308335 | T | C | 0.5913 | -0.0239 | 0.0039 | 9.21E-10 | 468924 | T | C | 0.5929 | -0.0029 | 0.004 | 0.465799 | 8.0081E-05 | 37.55473792 |
| LST | rs113838095 | 5 | 138435303 | T | C | 0.6684 | -0.0321 | 0.0043 | 6.88E-14 | 442658 | T | C | 0.6577 | -1.00E-04 | 0.0042 | 0.9839 | 0.000125878 | 55.72770927 |
| LST | rs114590429 | 2 | 166176789 | A | C | 0.1157 | 0.0376 | 0.006 | 3.03E-10 | 468924 | A | C | 0.1103 | -0.0081 | 0.0063 | 0.1991 | 8.37403E-05 | 39.27094362 |
| LST | rs11587591 | 1 | 209762875 | A | G | 0.7344 | 0.0219 | 0.0039 | 2.23E-08 | 526365 | A | G | 0.727 | -0.0048 | 0.0044 | 0.2764 | 5.99026E-05 | 31.53242457 |
| LST | rs1188887 | 6 | 139257866 | T | C | 0.6673 | 0.0258 | 0.0041 | 2.47E-10 | 468924 | T | C | 0.6568 | -0.0028 | 0.0041 | 0.495 | 8.4437E-05 | 39.59768953 |
| LST | rs11972285 | 7 | 99102777 | A | G | 0.8456 | 0.0384 | 0.0056 | 5.13E-12 | 442658 | A | G | 0.8488 | 0.0063 | 0.0055 | 0.2499 | 0.000106212 | 47.02019572 |
| LST | rs12062845 | 1 | 98342685 | A | C | 0.2158 | 0.0281 | 0.0042 | 2.41E-11 | 526370 | A | C | 0.2144 | 0.0015 | 0.0048 | 0.760001 | 8.50327E-05 | 44.76230158 |
| LST | rs12206846 | 6 | 108238917 | A | G | 0.4054 | 0.0199 | 0.0036 | 2.10E-08 | 525490 | A | G | 0.4087 | 0.0035 | 0.004 | 0.3775 | 5.81449E-05 | 30.55621086 |
| LST | rs12214364 | 6 | 67556372 | T | G | 0.5869 | -0.0197 | 0.0036 | 4.87E-08 | 525479 | T | G | 0.5945 | -5.00E-04 | 0.0042 | 0.9055 | 5.69833E-05 | 29.94510208 |
| LST | rs12324720 | 15 | 64092140 | A | G | 0.1756 | -0.0271 | 0.0046 | 3.53E-09 | 525490 | A | G | 0.1875 | -0.0035 | 0.0051 | 0.4925 | 6.60435E-05 | 34.70733482 |
| LST | rs12425850 | 12 | 123501972 | T | C | 0.286 | -0.0236 | 0.0039 | 1.37E-09 | 523034 | T | C | 0.297 | 2.00E-04 | 0.0043 | 0.9648 | 7.00059E-05 | 36.61787444 |
| LST | rs12463321 | 19 | 37651855 | A | G | 0.1478 | -0.0317 | 0.0055 | 6.99E-09 | 468924 | A | G | 0.1546 | -0.0015 | 0.0055 | 0.780899 | 7.0837E-05 | 33.21936245 |
| LST | rs12617870 | 2 | 193746283 | T | G | 0.5377 | 0.0263 | 0.0035 | 6.62E-14 | 525491 | T | G | 0.5255 | -0.0069 | 0.0041 | 0.0921404 | 0.000107439 | 56.46427489 |
| LST | rs12678836 | 8 | 92690148 | A | C | 0.424 | 0.0232 | 0.0035 | 5.37E-11 | 524165 | A | C | 0.4313 | 0.0035 | 0.004 | 0.3751 | 8.38176E-05 | 43.93779153 |
| LST | rs12962050 | 18 | 35179808 | A | G | 0.647 | -0.0234 | 0.0036 | 1.18E-10 | 525490 | A | G | 0.6543 | -0.0148 | 0.0041 | 0.000369496 | 8.03947E-05 | 42.2498392 |
| LST | rs12981974 | 19 | 19419810 | C | G | 0.1361 | 0.0337 | 0.0058 | 8.57E-09 | 468924 | C | G | 0.1724 | -0.003 | 0.0052 | 0.5662 | 7.19897E-05 | 33.75996303 |
| LST | rs13017586 | 2 | 147847198 | A | G | 0.8493 | -0.0401 | 0.0054 | 8.25E-14 | 468924 | A | G | 0.8516 | -0.0017 | 0.0055 | 0.7554 | 0.000117584 | 55.14414066 |
| LST | rs13089152 | 3 | 84765574 | T | C | 0.6691 | -0.0234 | 0.0041 | 1.53E-08 | 468924 | T | C | 0.678 | 8.00E-04 | 0.0043 | 0.8474 | 6.94595E-05 | 32.57332925 |
| LST | rs13188731 | 5 | 7387097 | A | T | 0.3671 | 0.0218 | 0.004 | 4.54E-08 | 468924 | A | T | 0.3725 | 0.0092 | 0.0041 | 0.02377 | 6.33378E-05 | 29.70237332 |
| LST | rs13235840 | 7 | 133505091 | A | T | 0.8186 | -0.0313 | 0.005 | 3.48E-10 | 468924 | A | T | 0.8161 | -0.0121 | 0.0051 | 0.01721 | 8.35622E-05 | 39.18743286 |
| LST | rs13301354 | 9 | 139924637 | T | C | 0.3769 | -0.0211 | 0.0037 | 1.22E-08 | 519906 | T | C | 0.3695 | 0.0102 | 0.0042 | 0.0154301 | 6.25474E-05 | 32.52069301 |
| LST | rs1391954 | 11 | 88575965 | T | G | 0.4439 | 0.0245 | 0.004 | 1.51E-09 | 442658 | T | G | 0.4506 | -0.0025 | 0.008 | 0.7535 | 8.47436E-05 | 37.5154555 |
| LST | rs1445979 | 5 | 60744339 | A | T | 0.3862 | 0.0196 | 0.0036 | 3.74E-08 | 525491 | A | T | 0.3783 | -0.0088 | 0.0041 | 0.0304797 | 5.6405E-05 | 29.64186249 |
| LST | rs16896229 | 4 | 18002583 | T | C | 0.1357 | 0.03 | 0.0051 | 3.80E-09 | 523040 | T | C | 0.1419 | 0.0115 | 0.0056 | 0.0415002 | 6.61513E-05 | 34.60194381 |
| LST | rs17621391 | 7 | 140176596 | T | C | 0.7351 | 0.0243 | 0.0041 | 2.11E-09 | 525485 | T | C | 0.728 | 0.0087 | 0.0044 | 0.0509695 | 6.68429E-05 | 35.12717148 |
| LST | rs17801257 | 20 | 58892520 | A | G | 0.1247 | -0.0292 | 0.0053 | 2.92E-08 | 525487 | A | G | 0.1277 | 0.0022 | 0.0059 | 0.7154 | 5.776E-05 | 30.35374706 |
| LST | rs1802669 | 10 | 21827796 | A | G | 0.3409 | 0.0384 | 0.0041 | 2.39E-21 | 468924 | A | G | 0.349 | 0.0122 | 0.0042 | 0.00350002 | 0.00018703 | 87.71884062 |
| LST | rs1860337 | 17 | 60851559 | T | C | 0.5953 | -0.0253 | 0.0039 | 9.08E-11 | 468924 | T | C | 0.5999 | -0.006 | 0.004 | 0.1356 | 8.97368E-05 | 42.08331821 |
| LST | rs1947066 | 5 | 161101615 | A | G | 0.8028 | 0.0298 | 0.0044 | 8.54E-12 | 525491 | A | G | 0.7996 | -0.007 | 0.0049 | 0.1564 | 8.72819E-05 | 45.86966013 |
| LST | rs197439 | 1 | 112280990 | A | G | 0.6007 | -0.0259 | 0.0039 | 3.29E-11 | 469809 | A | G | 0.5948 | -0.0028 | 0.004 | 0.4828 | 9.3866E-05 | 44.10303382 |
| LST | rs1999065 | 9 | 120514574 | T | C | 0.3377 | 0.0249 | 0.0037 | 1.24E-11 | 526725 | T | C | 0.3363 | 0.0106 | 0.0042 | 0.0111099 | 8.59754E-05 | 45.28909027 |
| LST | rs2473977 | 6 | 113454213 | A | T | 0.3862 | 0.0195 | 0.0036 | 4.60E-08 | 525491 | A | T | 0.3892 | -0.0014 | 0.004 | 0.724599 | 5.58309E-05 | 29.34016611 |
| LST | rs249960 | 5 | 96164771 | A | G | 0.8178 | 0.0298 | 0.005 | 2.43E-09 | 468924 | A | G | 0.8167 | -0.0045 | 0.0052 | 0.3915 | 7.57456E-05 | 35.5214485 |
| LST | rs2529484 | 7 | 111180444 | C | G | 0.3515 | 0.0221 | 0.0036 | 1.39E-09 | 525488 | C | G | 0.3561 | 0.002 | 0.0041 | 0.6303 | 7.1711E-05 | 37.68581336 |
| LST | rs262890 | 5 | 62930015 | A | G | 0.7003 | -0.0344 | 0.0042 | 2.06E-16 | 468924 | A | G | 0.7079 | 4.00E-04 | 0.0043 | 0.9336 | 0.000143039 | 67.08361411 |
| LST | rs2738284 | 2 | 217311609 | A | G | 0.3291 | -0.0215 | 0.0037 | 7.05E-09 | 523619 | A | G | 0.3272 | 0.0038 | 0.0042 | 0.36 | 6.44807E-05 | 33.76539331 |
| LST | rs2783992 | 9 | 1722044 | T | C | 0.5385 | -0.0242 | 0.0038 | 3.36E-10 | 468924 | T | C | 0.5555 | -0.0018 | 0.0039 | 0.6401 | 8.64816E-05 | 40.55661373 |
| LST | rs28458909 | 9 | 140257189 | T | C | 0.1229 | 0.0364 | 0.0059 | 5.48E-10 | 468924 | T | C | 0.1224 | 0.0035 | 0.0067 | 0.6031 | 8.11635E-05 | 38.06246334 |
| LST | rs2964252 | 5 | 152067929 | A | G | 0.3158 | -0.0235 | 0.0037 | 3.16E-10 | 525491 | A | G | 0.3118 | 0.0011 | 0.0042 | 0.7866 | 7.67598E-05 | 40.33951046 |
| LST | rs34864022 | 9 | 22609110 | A | G | 0.9341 | -0.0483 | 0.0078 | 4.71E-10 | 468924 | A | G | 0.9397 | -0.0115 | 0.0082 | 0.161 | 8.17649E-05 | 38.34451101 |
| LST | rs36079846 | 2 | 215367159 | T | C | 0.5205 | -0.0239 | 0.0038 | 4.52E-10 | 468924 | T | C | 0.5209 | 0.0029 | 0.0039 | 0.4667 | 8.43509E-05 | 39.55731051 |
| LST | rs364789 | 5 | 77387439 | A | G | 0.7568 | 0.0275 | 0.0044 | 2.79E-10 | 482490 | A | G | 0.7621 | 0.0043 | 0.0046 | 0.3542 | 8.09537E-05 | 39.06233808 |
| LST | rs3759344 | 12 | 6862646 | A | G | 0.1063 | 0.0452 | 0.0062 | 4.26E-13 | 468924 | A | G | 0.0987 | 0.0155 | 0.0066 | 0.0194998 | 0.000113329 | 53.14857665 |
| LST | rs3781412 | 10 | 126715154 | A | G | 0.6005 | -0.0208 | 0.0036 | 8.38E-09 | 519371 | A | G | 0.6055 | -0.0118 | 0.004 | 0.00318302 | 6.42711E-05 | 33.3825875 |
| LST | rs3791033 | 1 | 44134077 | T | C | 0.669 | 0.0331 | 0.0041 | 3.66E-16 | 469809 | T | C | 0.6696 | -0.0088 | 0.0042 | 0.0354201 | 0.00013871 | 65.17580821 |
| LST | rs396321 | 5 | 112113735 | T | C | 0.5128 | -0.0213 | 0.0035 | 1.29E-09 | 521893 | T | C | 0.4948 | -0.0072 | 0.004 | 0.0699101 | 7.09595E-05 | 37.03577644 |
| LST | rs421151 | 8 | 73462574 | A | G | 0.9161 | -0.036 | 0.0063 | 1.09E-08 | 524166 | A | G | 0.9152 | 0.0048 | 0.0071 | 0.5007 | 6.22914E-05 | 32.65293663 |
| LST | rs4311996 | 10 | 103735978 | A | T | 0.3873 | -0.0217 | 0.0039 | 3.56E-08 | 468924 | A | T | 0.3783 | -0.0055 | 0.0041 | 0.1792 | 6.60175E-05 | 30.9591053 |
| LST | rs4416502 | 4 | 77030872 | A | G | 0.1996 | 0.0291 | 0.0048 | 1.38E-09 | 468924 | A | G | 0.2159 | -0.0052 | 0.0048 | 0.2754 | 7.83731E-05 | 36.75374949 |
| LST | rs4460001 | 4 | 130275243 | A | C | 0.4324 | -0.0193 | 0.0035 | 4.45E-08 | 525491 | A | C | 0.4183 | -0.0027 | 0.0041 | 0.5013 | 5.78613E-05 | 30.40723121 |
| LST | rs4483592 | 11 | 65990439 | T | C | 0.1633 | 0.036 | 0.0052 | 3.97E-12 | 468924 | T | C | 0.1722 | -0.0089 | 0.0052 | 0.0867301 | 0.0001022 | 47.92878966 |
| LST | rs469565 | 22 | 29952437 | T | C | 0.7735 | -0.0259 | 0.0046 | 1.51E-08 | 468924 | T | C | 0.7737 | -0.0142 | 0.0047 | 0.00245098 | 6.76008E-05 | 31.70166063 |
| LST | rs4889530 | 16 | 31065918 | A | T | 0.3812 | -0.0253 | 0.0039 | 1.32E-10 | 468924 | A | T | 0.3923 | 0.0151 | 0.004 | 0.000176202 | 8.97368E-05 | 42.08331821 |
| LST | rs558134 | 6 | 12693454 | T | C | 0.3835 | -0.0225 | 0.0036 | 5.05E-10 | 525479 | T | C | 0.3833 | -0.0061 | 0.0041 | 0.1364 | 7.43314E-05 | 39.06235133 |
| LST | rs56151256 | 15 | 78024806 | A | C | 0.7498 | 0.0285 | 0.0044 | 1.17E-10 | 468924 | A | C | 0.7548 | -2.00E-04 | 0.0047 | 0.966 | 8.94629E-05 | 41.95488304 |
| LST | rs57092155 | 7 | 53856368 | T | C | 0.7826 | -0.0268 | 0.0047 | 8.80E-09 | 468924 | T | C | 0.7701 | -0.0026 | 0.0047 | 0.5756 | 6.93332E-05 | 32.51412117 |
| LST | rs58087899 | 1 | 1867426 | A | G | 0.4556 | -0.0222 | 0.004 | 4.35E-08 | 442658 | A | G | 0.4965 | 0.0016 | 0.0041 | 0.6854 | 6.95805E-05 | 30.80236083 |
| LST | rs58541850 | 6 | 166165563 | A | G | 0.0585 | 0.0522 | 0.0082 | 1.72E-10 | 468924 | A | G | 0.056 | 0.0116 | 0.0087 | 0.1824 | 8.64119E-05 | 40.52391996 |
| LST | rs6010651 | 20 | 62418243 | A | C | 0.6201 | 0.0235 | 0.004 | 3.34E-09 | 468924 | A | C | 0.6233 | 0.0025 | 0.0042 | 0.5457 | 7.36006E-05 | 34.51547779 |
| LST | rs6073637 | 20 | 43714051 | A | C | 0.5235 | -0.0225 | 0.0038 | 4.59E-09 | 468924 | A | C | 0.5126 | -0.0018 | 0.0039 | 0.6468 | 7.47589E-05 | 35.05871474 |
| LST | rs6102913 | 20 | 41202958 | T | C | 0.4736 | -0.0193 | 0.0035 | 2.97E-08 | 525489 | T | C | 0.4914 | -0.0049 | 0.0039 | 0.2133 | 5.78615E-05 | 30.40723121 |
| LST | rs61166637 | 4 | 140771814 | C | G | 0.6711 | 0.0242 | 0.0043 | 1.48E-08 | 442658 | C | G | 0.6729 | 0.0068 | 0.0085 | 0.4223 | 7.15475E-05 | 31.67319383 |
| LST | rs62068672 | 16 | 89647705 | T | C | 0.798 | 0.0263 | 0.0048 | 4.09E-08 | 468924 | T | C | 0.8108 | 0.015 | 0.0052 | 0.00408498 | 6.40175E-05 | 30.02113932 |
| LST | rs62134209 | 2 | 45093457 | A | G | 0.953 | 0.0538 | 0.0091 | 3.28E-09 | 468924 | A | G | 0.9565 | -0.0163 | 0.0097 | 0.0929501 | 7.45327E-05 | 34.9526344 |
| LST | rs62151809 | 2 | 104433256 | T | C | 0.4526 | 0.0228 | 0.0039 | 3.90E-09 | 468924 | T | C | 0.4505 | -0.0017 | 0.004 | 0.662799 | 7.28797E-05 | 34.17736902 |
| LST | rs62244886 | 3 | 71587392 | C | G | 0.6038 | 0.0239 | 0.0039 | 1.24E-09 | 468924 | C | G | 0.5992 | -0.0111 | 0.0041 | 0.00709398 | 8.0081E-05 | 37.55473792 |
| LST | rs6457816 | 6 | 35362848 | T | C | 0.9343 | -0.0414 | 0.007 | 3.97E-09 | 525488 | T | C | 0.9219 | -0.0036 | 0.0075 | 0.6282 | 6.65599E-05 | 34.97864238 |
| LST | rs6556840 | 5 | 93463902 | A | G | 0.3366 | 0.0204 | 0.0037 | 4.32E-08 | 525472 | A | G | 0.3292 | 0.0012 | 0.0042 | 0.7798 | 5.78472E-05 | 30.39871556 |
| LST | rs657412 | 13 | 99047250 | T | G | 0.1047 | -0.033 | 0.0058 | 1.40E-08 | 525489 | T | G | 0.1012 | -0.0028 | 0.0066 | 0.6703 | 6.16001E-05 | 32.37205277 |
| LST | rs6674314 | 1 | 243920895 | A | G | 0.8134 | 0.0274 | 0.0049 | 2.30E-08 | 469809 | A | G | 0.8199 | -0.0094 | 0.0051 | 0.0661104 | 6.65516E-05 | 31.26850496 |
| LST | rs6685030 | 1 | 171805284 | A | G | 0.48 | -0.0217 | 0.0035 | 5.27E-10 | 526371 | A | G | 0.4864 | -0.0057 | 0.004 | 0.1533 | 7.3023E-05 | 38.43985394 |
| LST | rs6727997 | 2 | 146346285 | A | G | 0.3445 | -0.0208 | 0.0037 | 2.04E-08 | 525489 | A | G | 0.3511 | 0.0047 | 0.0042 | 0.2606 | 6.01359E-05 | 31.60250938 |
| LST | rs68049022 | 10 | 66407019 | T | C | 0.7981 | 0.0314 | 0.0048 | 6.18E-11 | 468924 | T | C | 0.8014 | 0.0056 | 0.0049 | 0.2545 | 9.12504E-05 | 42.79322026 |
| LST | rs72671494 | 8 | 93195457 | T | C | 0.8595 | -0.0349 | 0.0056 | 5.72E-10 | 468924 | T | C | 0.8613 | -0.0077 | 0.006 | 0.1987 | 8.28202E-05 | 38.83943894 |
| LST | rs73405293 | 12 | 117522917 | A | G | 0.147 | -0.0308 | 0.0054 | 1.35E-08 | 468924 | A | G | 0.151 | 0.0021 | 0.0055 | 0.7001 | 6.93715E-05 | 32.53209719 |
| LST | rs73420302 | 17 | 77768068 | C | G | 0.1785 | -0.03 | 0.0051 | 3.04E-09 | 468924 | C | G | 0.1706 | -0.0049 | 0.0056 | 0.3817 | 7.37849E-05 | 34.60192854 |
| LST | rs7430216 | 3 | 75201030 | T | C | 0.2227 | 0.025 | 0.0042 | 2.50E-09 | 523040 | T | C | 0.2173 | 0.0121 | 0.0048 | 0.0110701 | 6.77356E-05 | 35.43070352 |
| LST | rs743699 | 4 | 3305116 | A | G | 0.7433 | -0.0267 | 0.0044 | 1.18E-09 | 468924 | A | G | 0.7363 | -0.0038 | 0.0045 | 0.3958 | 7.85201E-05 | 36.82267353 |
| LST | rs74996610 | 12 | 24075007 | C | G | 0.9545 | -0.0513 | 0.0093 | 3.12E-08 | 468924 | C | G | 0.9577 | -0.0045 | 0.0098 | 0.6446 | 6.48841E-05 | 30.42754972 |
| LST | rs7615206 | 3 | 49937505 | T | C | 0.5703 | -0.0345 | 0.0035 | 1.50E-22 | 521894 | T | C | 0.5802 | 0.0048 | 0.004 | 0.2249 | 0.00018614 | 97.16289296 |
| LST | rs7616518 | 3 | 83530809 | A | G | 0.4499 | 0.0196 | 0.0035 | 2.60E-08 | 525487 | A | G | 0.4519 | 0.0071 | 0.004 | 0.0778001 | 5.96744E-05 | 31.35988064 |
| LST | rs76267866 | 3 | 70540347 | A | T | 0.7923 | -0.0297 | 0.0047 | 3.53E-10 | 468924 | A | T | 0.7948 | 0.0051 | 0.0049 | 0.2948 | 8.51486E-05 | 39.93147297 |
| LST | rs7627290 | 3 | 165719378 | A | G | 0.5413 | -0.0231 | 0.0039 | 2.36E-09 | 468924 | A | G | 0.6028 | -0.0065 | 0.004 | 0.1055 | 7.481E-05 | 35.08269061 |
| LST | rs76602404 | 7 | 50737835 | T | C | 0.2194 | -0.0266 | 0.0049 | 4.38E-08 | 442658 | T | C | 0.2339 | -0.003 | 0.0094 | 0.753199 | 6.65693E-05 | 29.46925461 |
| LST | rs7821826 | 8 | 10769439 | T | C | 0.4925 | 0.0207 | 0.0035 | 3.25E-09 | 524166 | T | C | 0.4878 | 0.0073 | 0.0039 | 0.0631306 | 6.67278E-05 | 34.97864205 |
| LST | rs78394231 | 6 | 107649123 | T | C | 0.9015 | -0.0381 | 0.0065 | 3.53E-09 | 468924 | T | C | 0.9042 | 0.0017 | 0.0067 | 0.8008 | 7.32637E-05 | 34.3574866 |
| LST | rs78451709 | 18 | 41515058 | T | C | 0.7954 | -0.0277 | 0.0048 | 9.66E-09 | 468924 | T | C | 0.7957 | 0.0078 | 0.0049 | 0.1095 | 7.1014E-05 | 33.30237532 |
| LST | rs7875078 | 9 | 14494845 | A | C | 0.4517 | 0.0204 | 0.0035 | 7.41E-09 | 526722 | A | C | 0.4605 | 0.0032 | 0.004 | 0.4153 | 6.44933E-05 | 33.9721159 |
| LST | rs7969719 | 12 | 109883577 | T | C | 0.3131 | 0.0271 | 0.0037 | 4.46E-13 | 525490 | T | C | 0.3212 | 0.0069 | 0.0042 | 0.1004 | 0.000102077 | 53.64552263 |
| LST | rs7991062 | 13 | 100739135 | C | G | 0.6577 | -0.0335 | 0.0041 | 1.32E-16 | 468924 | C | G | 0.6573 | -0.0112 | 0.0041 | 0.00656705 | 0.00014235 | 66.76057189 |
| LST | rs841020 | 10 | 125409953 | T | C | 0.1923 | 0.0269 | 0.0044 | 1.15E-09 | 525491 | T | C | 0.1933 | -0.0115 | 0.005 | 0.0223599 | 7.11218E-05 | 37.37640733 |
| LST | rs892087 | 19 | 10794793 | T | C | 0.6335 | -0.0284 | 0.004 | 9.98E-13 | 468924 | T | C | 0.6407 | 0.0052 | 0.0041 | 0.2078 | 0.00010749 | 50.409785 |
| LST | rs9278004 | 6 | 33319815 | A | G | 0.1487 | 0.0394 | 0.0057 | 3.23E-12 | 442658 | A | G | 0.1575 | 0.0051 | 0.0054 | 0.3468 | 0.000107926 | 47.77940862 |
| LST | rs9713906 | 3 | 93543006 | A | T | 0.7409 | -0.0235 | 0.004 | 3.86E-09 | 525491 | A | T | 0.7602 | 0.0045 | 0.0047 | 0.3291 | 6.56783E-05 | 34.51549363 |
| LST | rs9867121 | 3 | 114631548 | A | C | 0.1829 | -0.0317 | 0.005 | 2.02E-10 | 468924 | A | C | 0.192 | -0.022 | 0.0051 | 1.51E-05 | 8.57114E-05 | 40.19542856 |
| MVPA | rs1160545 | 2 | 100832269 | T | C | 0.4025 | 0.0249 | 0.0041 | 1.73E-09 | 483768 | T | C | 0.4025 | -0.0048 | 0.004 | 0.2326 | 7.62361E-05 | 36.88325025 |
| MVPA | rs13201721 | 6 | 141799534 | T | C | 0.7364 | 0.0255 | 0.004 | 1.83E-10 | 522597 | T | C | 0.7364 | -0.0055 | 0.0045 | 0.2179 | 7.77606E-05 | 40.64046947 |
| MVPA | rs1625595 | 11 | 66078129 | T | C | 0.4748 | -0.0213 | 0.0032 | 1.90E-11 | 592552 | T | C | 0.4748 | 8.00E-04 | 0.0041 | 0.8362 | 7.47653E-05 | 44.30551452 |
| MVPA | rs1691471 | 3 | 85011013 | T | C | 0.3759 | 0.0379 | 0.0042 | 1.73E-19 | 483768 | T | C | 0.3759 | -0.0068 | 0.0041 | 0.0961103 | 0.000168294 | 81.42880168 |
| MVPA | rs2668196 | 3 | 165502709 | A | T | 0.1908 | -0.0227 | 0.004 | 2.09E-08 | 601806 | A | T | 0.1908 | 0.0031 | 0.005 | 0.5315 | 5.35121E-05 | 32.20551797 |
| MVPA | rs336620 | 3 | 18628793 | C | G | 0.3016 | 0.0243 | 0.0044 | 4.05E-08 | 483768 | C | G | 0.3016 | -0.0027 | 0.0043 | 0.5318 | 6.30438E-05 | 30.50039043 |
| MVPA | rs370935521 | 6 | 26802963 | T | C | 0.0926 | 0.0434 | 0.0075 | 8.25E-09 | 448902 | T | C | 0.0926 | 0.0174 | 0.007 | 0.01238 | 7.45887E-05 | 33.48536192 |
| MVPA | rs385301 | 17 | 19806828 | T | C | 0.2637 | -0.0284 | 0.0047 | 1.60E-09 | 461744 | T | C | 0.2637 | 0.006 | 0.0045 | 0.1837 | 7.90688E-05 | 36.51229092 |
| MVPA | rs4352559 | 5 | 60586625 | T | C | 0.4964 | 0.018 | 0.0032 | 1.65E-08 | 599961 | T | C | 0.4964 | 0.0068 | 0.004 | 0.0862601 | 5.2735E-05 | 31.64051952 |
| MVPA | rs4865512 | 5 | 50661601 | A | G | 0.6124 | 0.024 | 0.0042 | 7.68E-09 | 483768 | A | G | 0.6124 | 0.0185 | 0.004 | 4.48E-06 | 6.74928E-05 | 32.65292623 |
| MVPA | rs568546 | 11 | 107321156 | T | C | 0.5206 | 0.0237 | 0.0041 | 5.89E-09 | 483768 | T | C | 0.5206 | 0.0019 | 0.0039 | 0.6281 | 6.90656E-05 | 33.41390112 |
| MVPA | rs6427178 | 1 | 169095082 | A | G | 0.5318 | 0.0229 | 0.0041 | 1.71E-08 | 484633 | A | G | 0.5318 | -0.0037 | 0.0039 | 0.3403 | 6.43669E-05 | 31.19618298 |
| MVPA | rs7613360 | 3 | 49916710 | T | C | 0.3963 | -0.0247 | 0.0042 | 2.77E-09 | 483768 | T | C | 0.3963 | -0.0053 | 0.0041 | 0.1887 | 7.1487E-05 | 34.58545792 |
| MVPA | rs9903845 | 17 | 50291181 | A | C | 0.3104 | -0.02 | 0.0034 | 6.05E-09 | 604232 | A | C | 0.3104 | 0.0089 | 0.0042 | 0.0345303 | 5.72629E-05 | 34.60196159 |
| SDC | rs112560164 | 14 | 93112924 | A | G | 0.1893 | -0.0479 | 0.0102 | 2.80E-06 | 124924 | A | G | 0.1893 | 0.0163 | 0.1893 | 0.00126299 | 0.000176501 | 22.05279957 |
| SDC | rs117992146 | 14 | 24795687 | T | C | 0.0585 | -0.0799 | 0.0171 | 3.17E-06 | 124924 | T | C | 0.0581 | -0.0229 | 0.0581 | 0.1647 | 0.000174735 | 21.83204334 |
| SDC | rs13425445 | 2 | 1873425 | C | G | 0.9173 | 0.0639 | 0.0135 | 2.35E-06 | 157045 | C | G | 0.9166 | -0.0038 | 0.9166 | 0.6028 | 0.000142642 | 22.40415912 |
| SDC | rs1423299 | 5 | 33833140 | A | T | 0.0427 | -0.0918 | 0.0187 | 9.42E-07 | 157045 | A | T | 0.0389 | 0.0018 | 0.0389 | 0.8591 | 0.00015343 | 24.09886665 |
| SDC | rs144224971 | 4 | 25575549 | T | C | 0.9852 | -0.1735 | 0.0375 | 3.67E-06 | 123626 | T | C | 0.969 | 0.0518 | 0.969 | 0.3818 | 0.000173122 | 21.40569814 |
| SDC | rs149246922 | 12 | 128921283 | T | C | 0.0267 | 0.1346 | 0.0266 | 4.08E-07 | 124924 | T | C | 0.0257 | 0.0432 | 0.0257 | 0.1287 | 0.000204924 | 25.6047119 |
| SDC | rs162064 | 3 | 107824061 | T | G | 0.356 | -0.0353 | 0.0077 | 4.03E-06 | 157045 | T | G | 0.3519 | 0.0058 | 0.3519 | 0.1602 | 0.000133809 | 21.0165986 |
| SDC | rs16908418 | 8 | 139185487 | T | C | 0.0182 | 0.1262 | 0.0274 | 4.14E-06 | 156383 | T | C | 0.0199 | -0.0083 | 0.0199 | 0.573 | 0.000135634 | 21.21348542 |
| SDC | rs2414010 | 15 | 49983627 | A | G | 0.9109 | -0.0598 | 0.0129 | 3.56E-06 | 157044 | A | G | 0.9116 | 0.0074 | 0.9116 | 0.295 | 0.000136818 | 21.4890599 |
| SDC | rs308676 | 13 | 62685049 | T | C | 0.8626 | -0.0577 | 0.0108 | 7.92E-08 | 157046 | T | C | 0.8745 | 0.0042 | 0.8745 | 0.4952 | 0.000181718 | 28.54293211 |
| SDC | rs35888745 | 10 | 49389869 | T | C | 0.6007 | 0.0392 | 0.0082 | 1.68E-06 | 124924 | T | C | 0.6088 | 0.0023 | 0.6088 | 0.5914 | 0.000182902 | 22.85269778 |
| SDC | rs62280512 | 3 | 166551816 | A | G | 0.9548 | 0.0902 | 0.0196 | 4.08E-06 | 124924 | A | G | 0.9519 | -0.0221 | 0.9519 | 0.0244501 | 0.000169505 | 21.17844061 |
| SDC | rs73046678 | 7 | 3371449 | A | C | 0.1624 | -0.0516 | 0.0109 | 2.11E-06 | 124924 | A | C | 0.1647 | -0.0086 | 0.1647 | 0.1042 | 0.000179359 | 22.40987605 |
| SDC | rs76374890 | 10 | 129676918 | T | C | 0.0579 | -0.0876 | 0.0172 | 3.31E-07 | 124924 | T | C | 0.0618 | 0.007 | 0.0618 | 0.401 | 0.000207594 | 25.93847061 |
| SDW | rs13316695 | 3 | 49684917 | C | G | 0.7075 | -0.0354 | 0.0061 | 6.31E-09 | 284476 | C | G | 0.6987 | -0.0125 | 0.0043 | 0.00352403 | 0.000118372 | 33.67780676 |
| SDW | rs1446951 | 13 | 58114422 | A | C | 0.566 | -0.0243 | 0.0042 | 1.04E-08 | 370955 | A | C | 0.5494 | 0.0023 | 0.004 | 0.569399 | 9.02305E-05 | 33.47430932 |
| SDW | rs2265951 | 13 | 21305164 | A | G | 0.312 | 0.0317 | 0.0058 | 4.60E-08 | 309813 | A | G | 0.3109 | 0.0052 | 0.0043 | 0.2246 | 9.64098E-05 | 29.87168588 |
| SDW | rs4518438 | 5 | 88157552 | T | C | 0.468 | 0.026 | 0.0042 | 4.41E-10 | 370957 | T | C | 0.4868 | -0.002 | 0.0039 | 0.6083 | 0.000103295 | 38.32178885 |
| SDW | rs4596363 | 5 | 92596445 | A | G | 0.3116 | -0.0327 | 0.0058 | 1.56E-08 | 317478 | A | G | 0.2971 | -0.0061 | 0.0043 | 0.1595 | 0.000100111 | 31.78606611 |
| SDW | rs79248502 | 5 | 111012600 | C | G | 0.9407 | -0.0655 | 0.0118 | 3.03E-08 | 284476 | C | G | 0.9437 | -0.0134 | 0.0085 | 0.1132 | 0.000108299 | 30.81169088 |
| SDW | rs8450 | 1 | 153920286 | A | G | 0.3048 | -0.0315 | 0.005 | 2.47E-10 | 297564 | A | G | 0.3094 | -0.0061 | 0.0042 | 0.1534 | 0.000133365 | 39.68973323 |
| SDW | rs9482120 | 6 | 98392667 | A | C | 0.6029 | -0.0536 | 0.0057 | 3.44E-21 | 284476 | A | C | 0.6035 | 0.01 | 0.004 | 0.01273 | 0.000310742 | 88.42535555 |
| SDW | rs995638 | 4 | 130364131 | A | G | 0.3939 | -0.0237 | 0.0043 | 2.53E-08 | 370868 | A | G | 0.3939 | 0.0044 | 0.004 | 0.2785 | 8.19039E-05 | 30.37787836 |
| SmkInit | rs10001365 | 4 | 147797214 | A | G | 0.378728 | -0.0152 | 0.002 | 4.32E-21 | 805431 | A | G | 0.3982 | -0.0097 | 0.004 | 0.0158001 | 7.1708E-05 | 57.75985657 |
| SmkInit | rs1004787 | 2 | 45159091 | A | G | 0.535785 | 0.0161 | 0.002 | 3.91E-24 | 805431 | A | G | 0.55 | -0.0035 | 0.004 | 0.374 | 8.04505E-05 | 64.80233909 |
| SmkInit | rs10062607 | 5 | 79290634 | A | C | 0.635189 | 0.0103 | 0.002 | 2.28E-10 | 805431 | A | C | 0.6173 | 0.0078 | 0.004 | 0.0528202 | 3.29285E-05 | 26.52243414 |
| SmkInit | rs10119117 | 9 | 29740028 | T | C | 0.497018 | 0.00901 | 0.002 | 1.80E-08 | 781945 | T | C | 0.5193 | -0.0015 | 0.0039 | 0.708101 | 2.59539E-05 | 20.29497309 |
| SmkInit | rs10233018 | 7 | 117523709 | G | A | 0.512922 | 0.0139 | 0.002 | 1.49E-18 | 805431 | G | A | 0.5178 | -0.0058 | 0.0039 | 0.1395 | 5.99674E-05 | 48.30238006 |
| SmkInit | rs1025910 | 18 | 49871340 | C | G | 0.294235 | -0.0123 | 0.002 | 4.83E-13 | 805431 | C | G | 0.3058 | 2.00E-04 | 0.0043 | 0.9552 | 4.69571E-05 | 37.82240608 |
| SmkInit | rs10279261 | 7 | 133589846 | A | G | 0.629225 | -0.0122 | 0.002 | 6.87E-14 | 805431 | A | G | 0.614 | 0.0061 | 0.0041 | 0.1307 | 4.61967E-05 | 37.2099076 |
| SmkInit | rs10444314 | 11 | 7951242 | G | T | 0.428429 | -0.00922 | 0.002 | 1.77E-08 | 780334 | G | T | 0.4068 | -1.00E-04 | 0.004 | 0.9763 | 2.72339E-05 | 21.25204553 |
| SmkInit | rs10458563 | 1 | 50612250 | G | A | 0.190855 | 0.0171 | 0.002 | 2.17E-18 | 805431 | G | A | 0.196 | -0.0032 | 0.005 | 0.523801 | 9.07537E-05 | 73.10231848 |
| SmkInit | rs1050847 | 16 | 87443734 | T | C | 0.550696 | -0.0105 | 0.002 | 3.71E-11 | 805431 | T | C | 0.5529 | 0.0036 | 0.004 | 0.3716 | 3.42196E-05 | 27.56243156 |
| SmkInit | rs10698713 | 6 | 158882320 | A | G | 0.0526839 | -0.0201 | 0.004 | 1.84E-08 | 805431 | A | G | 0.0542 | 0.0033 | 0.0089 | 0.7124 | 3.13495E-05 | 25.2505623 |
| SmkInit | rs10745324 | 1 | 112708722 | G | A | 0.672962 | -0.00962 | 0.002 | 1.97E-08 | 748334 | G | A | 0.6506 | -0.0086 | 0.0041 | 0.0365502 | 3.09159E-05 | 23.13603817 |
| SmkInit | rs10753630 | 1 | 163790451 | T | C | 0.348907 | 0.0109 | 0.002 | 9.96E-11 | 780334 | T | C | 0.3372 | 0.0032 | 0.0042 | 0.4404 | 3.80624E-05 | 29.70242387 |
| SmkInit | rs10786721 | 10 | 104654383 | A | C | 0.416501 | 0.0179 | 0.002 | 7.91E-27 | 748334 | A | C | 0.4114 | 0.0075 | 0.004 | 0.0591698 | 0.00010703 | 80.10228592 |
| SmkInit | rs10927039 | 1 | 243721726 | C | T | 0.196819 | -0.0127 | 0.002 | 2.39E-10 | 805431 | C | T | 0.1798 | 0.0115 | 0.0051 | 0.0240099 | 5.00608E-05 | 40.32239987 |
| SmkInit | rs11012726 | 10 | 21797272 | C | T | 0.33002 | 0.0143 | 0.002 | 4.25E-17 | 805431 | C | T | 0.3204 | 0.0144 | 0.0043 | 0.000750499 | 6.34682E-05 | 51.12237306 |
| SmkInit | rs11078713 | 17 | 7795972 | G | A | 0.408549 | -0.0126 | 0.002 | 2.77E-15 | 805431 | G | A | 0.4209 | -0.0029 | 0.004 | 0.467 | 4.92755E-05 | 39.68990144 |
| SmkInit | rs11103667 | 9 | 137978360 | T | C | 0.178926 | 0.0129 | 0.002 | 1.47E-10 | 805431 | T | C | 0.1914 | 0.0148 | 0.0052 | 0.00471205 | 5.16498E-05 | 41.6023967 |
| SmkInit | rs1111578 | 12 | 117913256 | T | G | 0.15507 | -0.0125 | 0.002 | 8.22E-09 | 805431 | T | G | 0.1522 | 0.0012 | 0.0054 | 0.8284 | 4.84965E-05 | 39.062403 |
| SmkInit | rs11130381 | 3 | 53850005 | T | C | 0.525845 | -0.00862 | 0.002 | 4.83E-08 | 805431 | T | C | 0.5407 | -3.00E-04 | 0.0041 | 0.9457 | 2.3063E-05 | 18.57605387 |
| SmkInit | rs11162019 | 1 | 87913176 | T | C | 0.379722 | -0.00971 | 0.002 | 3.40E-09 | 805431 | T | C | 0.3636 | 0.0085 | 0.0041 | 0.0376799 | 2.92643E-05 | 23.57096647 |
| SmkInit | rs111861749 | 6 | 50889019 | A | G | 0.124254 | -0.0179 | 0.003 | 3.16E-12 | 805431 | A | G | 0.1046 | -0.0029 | 0.0064 | 0.6526 | 4.41994E-05 | 35.60102271 |
| SmkInit | rs11192160 | 10 | 106539062 | C | G | 0.185885 | 0.0139 | 0.002 | 8.66E-10 | 630626 | C | G | 0.1738 | 0.0124 | 0.0052 | 0.01632 | 7.65887E-05 | 48.30234681 |
| SmkInit | rs11258417 | 10 | 13533053 | T | C | 0.335984 | -0.0101 | 0.002 | 3.83E-10 | 805431 | T | C | 0.3826 | 0.0029 | 0.004 | 0.4679 | 3.16622E-05 | 25.50243667 |
| SmkInit | rs1126757 | 19 | 55879872 | T | C | 0.472167 | 0.0104 | 0.002 | 4.15E-11 | 805431 | T | C | 0.4704 | -5.00E-04 | 0.004 | 0.8958 | 3.3571E-05 | 27.03993286 |
| SmkInit | rs114900182 | 16 | 72629056 | G | C | 0.0586481 | -0.0209 | 0.003 | 1.45E-12 | 805431 | G | C | 0.0667 | -0.0079 | 0.0079 | 0.3208 | 6.02553E-05 | 48.53432393 |
| SmkInit | rs1155641 | 15 | 97502995 | A | G | 0.333996 | 0.0102 | 0.002 | 7.72E-10 | 805430 | A | G | 0.3449 | -0.0126 | 0.0041 | 0.00227101 | 3.22923E-05 | 26.00993541 |
| SmkInit | rs11626595 | 14 | 104593623 | T | C | 0.156064 | -0.0151 | 0.002 | 2.73E-12 | 805431 | T | C | 0.1596 | -0.0028 | 0.0056 | 0.617 | 7.07677E-05 | 57.00235845 |
| SmkInit | rs11632439 | 15 | 80987012 | G | A | 0.44831 | 0.00898 | 0.002 | 1.31E-08 | 805430 | G | A | 0.4667 | 0.0032 | 0.0039 | 0.4233 | 2.50296E-05 | 20.16004994 |
| SmkInit | rs11656151 | 17 | 44068492 | G | A | 0.200795 | -0.0128 | 0.002 | 5.61E-12 | 805431 | G | A | 0.2404 | 0.0096 | 0.0046 | 0.0373499 | 5.08522E-05 | 40.95989829 |
| SmkInit | rs11673452 | 19 | 33928001 | C | T | 0.11332 | 0.015 | 0.003 | 3.40E-09 | 805431 | C | T | 0.109 | -0.0062 | 0.0063 | 0.3244 | 3.10383E-05 | 24.99993792 |
| SmkInit | rs11695197 | 2 | 97711421 | A | G | 0.115308 | 0.0138 | 0.002 | 6.28E-09 | 799280 | A | G | 0.1294 | -0.0132 | 0.0133 | 0.3212 | 5.95626E-05 | 47.60988087 |
| SmkInit | rs11716705 | 3 | 34728753 | G | A | 0.267396 | 0.0117 | 0.002 | 1.40E-10 | 805431 | G | A | 0.2582 | -4.00E-04 | 0.0047 | 0.9252 | 4.24879E-05 | 34.22241502 |
| SmkInit | rs11742625 | 5 | 91396113 | C | T | 0.115308 | 0.0157 | 0.002 | 2.19E-10 | 805431 | C | T | 0.1206 | -0.0053 | 0.0061 | 0.3783 | 7.65029E-05 | 61.62234698 |
| SmkInit | rs11756490 | 6 | 100341774 | A | T | 0.129225 | -0.0137 | 0.002 | 6.20E-09 | 805431 | A | T | 0.1219 | 5.00E-04 | 0.0061 | 0.94 | 5.82542E-05 | 46.92238348 |
| SmkInit | rs11865123 | 16 | 5841406 | A | T | 0.27336 | 0.0106 | 0.002 | 4.10E-08 | 697342 | A | T | 0.2551 | 9.00E-04 | 0.0045 | 0.8412 | 4.02799E-05 | 28.08991944 |
| SmkInit | rs11873164 | 18 | 42659922 | T | C | 0.131213 | -0.0155 | 0.002 | 1.63E-12 | 805431 | T | C | 0.1578 | -0.0041 | 0.0054 | 0.4437 | 7.45663E-05 | 60.06235086 |
| SmkInit | rs11926232 | 3 | 55936417 | G | A | 0.0467197 | 0.0206 | 0.004 | 1.16E-08 | 805431 | G | A | 0.0462 | 7.00E-04 | 0.0095 | 0.9394 | 3.29285E-05 | 26.52243414 |
| SmkInit | rs12027999 | 1 | 154206358 | C | T | 0.151093 | -0.0184 | 0.002 | 1.54E-13 | 732296 | C | T | 0.1148 | -0.0217 | 0.0063 | 0.000556096 | 0.000115568 | 84.63976884 |
| SmkInit | rs12036050 | 1 | 96917491 | C | T | 0.284294 | 0.0115 | 0.002 | 7.18E-11 | 805431 | C | T | 0.2892 | -0.0023 | 0.0043 | 0.5966 | 4.10478E-05 | 33.0624179 |
| SmkInit | rs12079063 | 1 | 174079585 | G | A | 0.447316 | -0.00872 | 0.002 | 3.88E-08 | 805431 | G | A | 0.4449 | 0.0061 | 0.004 | 0.1282 | 2.36012E-05 | 19.0095528 |
| SmkInit | rs12112638 | 7 | 69735251 | G | A | 0.234592 | -0.0122 | 0.002 | 8.74E-12 | 805431 | G | A | 0.2688 | -0.0013 | 0.0045 | 0.7693 | 4.61967E-05 | 37.2099076 |
| SmkInit | rs12129573 | 1 | 73768366 | A | C | 0.39662 | 0.0142 | 0.002 | 6.44E-18 | 805431 | A | C | 0.3716 | -0.0059 | 0.0041 | 0.151 | 6.25837E-05 | 50.40987482 |
| SmkInit | rs12133063 | 1 | 91214714 | A | C | 0.371769 | 0.0116 | 0.002 | 1.15E-12 | 805431 | A | C | 0.3568 | 0.0015 | 0.0041 | 0.7163 | 4.17647E-05 | 33.63991647 |
| SmkInit | rs12441907 | 15 | 83922387 | A | C | 0.166998 | -0.015 | 0.002 | 6.09E-14 | 805430 | A | C | 0.1912 | -1.00E-04 | 0.005 | 0.9918 | 6.98336E-05 | 56.24986032 |
| SmkInit | rs1246265 | 9 | 86761745 | C | T | 0.701789 | 0.0116 | 0.002 | 1.27E-11 | 805431 | C | T | 0.6928 | -0.0073 | 0.0043 | 0.0850394 | 4.17647E-05 | 33.63991647 |
| SmkInit | rs12485391 | 3 | 181205593 | A | C | 0.12326 | 0.0138 | 0.002 | 1.89E-08 | 805431 | A | C | 0.1207 | 0.0115 | 0.0061 | 0.0598894 | 5.91077E-05 | 47.60988178 |
| SmkInit | rs12600466 | 17 | 50306926 | T | A | 0.301193 | 0.0115 | 0.002 | 1.30E-11 | 805431 | T | A | 0.3167 | 0.01 | 0.0042 | 0.0176401 | 4.10478E-05 | 33.0624179 |
| SmkInit | rs12632110 | 3 | 50224225 | G | A | 0.663022 | -0.0138 | 0.002 | 1.18E-16 | 805431 | G | A | 0.66 | 0.0067 | 0.0043 | 0.1191 | 5.91077E-05 | 47.60988178 |
| SmkInit | rs12642744 | 4 | 28027176 | T | G | 0.757455 | -0.0124 | 0.002 | 5.98E-11 | 748334 | T | G | 0.7446 | -0.0036 | 0.0045 | 0.4237 | 5.13648E-05 | 38.43989727 |
| SmkInit | rs12685816 | 9 | 8290929 | A | G | 0.150099 | 0.0145 | 0.002 | 1.66E-10 | 781945 | A | G | 0.1426 | 0.0134 | 0.0056 | 0.0172199 | 6.72157E-05 | 52.56236556 |
| SmkInit | rs12714017 | 2 | 80999398 | C | T | 0.532803 | 0.0103 | 0.002 | 6.57E-10 | 723237 | C | T | 0.5098 | -0.0094 | 0.0039 | 0.0176999 | 3.66706E-05 | 26.52242666 |
| SmkInit | rs12742446 | 1 | 33882668 | T | G | 0.630219 | 0.0119 | 0.002 | 2.74E-10 | 630626 | T | G | 0.6677 | -0.0055 | 0.0042 | 0.1853 | 5.61355E-05 | 35.40238772 |
| SmkInit | rs12760908 | 1 | 190889685 | T | C | 0.297217 | 0.0109 | 0.002 | 3.90E-08 | 624207 | T | C | 0.2891 | 0.0051 | 0.0043 | 0.236 | 4.75821E-05 | 29.70240483 |
| SmkInit | rs1291865 | 10 | 11082192 | T | G | 0.531809 | 0.0116 | 0.002 | 2.12E-13 | 805431 | T | G | 0.509 | -0.0014 | 0.004 | 0.723099 | 4.17647E-05 | 33.63991647 |
| SmkInit | rs12923427 | 16 | 17575065 | T | C | 0.219682 | -0.0136 | 0.002 | 4.96E-12 | 805431 | T | C | 0.2122 | 0.0052 | 0.0048 | 0.2851 | 5.7407E-05 | 46.23988518 |
| SmkInit | rs12945403 | 17 | 80004094 | C | T | 0.616302 | -0.00941 | 0.002 | 1.79E-08 | 804239 | C | T | 0.6537 | 0.0141 | 0.0044 | 0.00131501 | 2.75247E-05 | 22.13696995 |
| SmkInit | rs13009008 | 2 | 174043233 | G | A | 0.651093 | -0.0097 | 0.002 | 6.92E-09 | 805431 | G | A | 0.6609 | -0.0175 | 0.0042 | 2.50E-05 | 2.9204E-05 | 23.52244159 |
| SmkInit | rs13030994 | 2 | 146143090 | A | G | 0.508946 | 0.0207 | 0.002 | 2.42E-39 | 805431 | A | G | 0.4794 | 0.0028 | 0.004 | 0.4767 | 0.000132983 | 107.122234 |
| SmkInit | rs13145728 | 4 | 140927812 | C | G | 0.360835 | -0.0122 | 0.002 | 6.87E-14 | 805431 | C | G | 0.3711 | -0.0106 | 0.0041 | 0.00921298 | 4.61967E-05 | 37.2099076 |
| SmkInit | rs13162305 | 5 | 12122698 | T | A | 0.33499 | 0.00975 | 0.002 | 1.15E-08 | 780334 | T | A | 0.3287 | 0.0056 | 0.0042 | 0.1827 | 3.04548E-05 | 23.76556409 |
| SmkInit | rs13255625 | 8 | 144269567 | G | A | 0.695825 | 0.0116 | 0.002 | 4.03E-12 | 805431 | G | A | 0.6703 | -0.0052 | 0.0044 | 0.2426 | 4.17647E-05 | 33.63991647 |
| SmkInit | rs13261725 | 8 | 91866297 | C | G | 0.261431 | 0.0131 | 0.002 | 3.43E-13 | 805431 | C | G | 0.257 | 0.0036 | 0.0045 | 0.4257 | 5.32637E-05 | 42.90239347 |
| SmkInit | rs13301073 | 9 | 128284378 | A | G | 0.348907 | 0.0109 | 0.002 | 3.13E-11 | 805431 | A | G | 0.3669 | -0.0077 | 0.0041 | 0.0591793 | 3.68764E-05 | 29.70242624 |
| SmkInit | rs1334557 | 10 | 8790819 | T | C | 0.245527 | 0.0132 | 0.002 | 1.51E-12 | 805431 | T | C | 0.2447 | 0.0072 | 0.0046 | 0.1184 | 5.40799E-05 | 43.55989183 |
| SmkInit | rs13398418 | 2 | 203717108 | A | T | 0.259443 | 0.0119 | 0.002 | 2.09E-11 | 805431 | A | T | 0.2456 | -0.0094 | 0.0046 | 0.0410403 | 4.39528E-05 | 35.40241209 |
| SmkInit | rs134529 | 22 | 28781758 | C | T | 0.379722 | -0.0116 | 0.002 | 8.86E-13 | 805431 | C | T | 0.3605 | 0.0041 | 0.0041 | 0.3096 | 4.17647E-05 | 33.63991647 |
| SmkInit | rs1381287 | 14 | 98597552 | T | C | 0.50497 | 0.0123 | 0.002 | 7.51E-15 | 805431 | T | C | 0.4738 | 0.0019 | 0.004 | 0.6353 | 4.69571E-05 | 37.82240608 |
| SmkInit | rs1392446 | 5 | 30831387 | T | C | 0.532803 | 0.00981 | 0.002 | 5.60E-10 | 805431 | T | C | 0.5447 | -0.0023 | 0.004 | 0.560101 | 2.98701E-05 | 24.05896526 |
| SmkInit | rs1435679 | 15 | 36399245 | A | G | 0.581511 | 0.01 | 0.002 | 2.86E-10 | 805430 | A | G | 0.5595 | -0.0091 | 0.004 | 0.0212202 | 3.10384E-05 | 24.99993792 |
| SmkInit | rs143909875 | 2 | 59294197 | C | T | 0.107356 | 0.0176 | 0.003 | 3.74E-12 | 805431 | C | T | 0.102 | -0.0075 | 0.0066 | 0.2551 | 4.27303E-05 | 34.41769231 |
| SmkInit | rs146628116 | 6 | 67554511 | A | T | 0.379722 | 0.0144 | 0.002 | 1.43E-14 | 603064 | A | T | 0.3978 | 5.00E-04 | 0.0041 | 0.9104 | 8.59536E-05 | 51.83982808 |
| SmkInit | rs147052174 | 1 | 179783167 | T | G | 0.0149105 | 0.0344 | 0.006 | 1.31E-08 | 804537 | T | G | 0.0173 | 0.015 | 0.015 | 0.3171 | 4.08555E-05 | 32.8710294 |
| SmkInit | rs1485272 | 3 | 3727589 | C | T | 0.37674 | -0.00907 | 0.002 | 4.07E-08 | 805431 | C | T | 0.3475 | -0.0057 | 0.0041 | 0.1681 | 2.55338E-05 | 20.56617393 |
| SmkInit | rs1499982 | 3 | 117820386 | T | C | 0.835984 | 0.0216 | 0.002 | 3.01E-23 | 805431 | T | C | 0.8577 | 0.003 | 0.0057 | 0.596901 | 0.000144796 | 116.6397104 |
| SmkInit | rs1503211 | 4 | 94079508 | A | G | 0.454274 | 0.00999 | 0.002 | 3.43E-10 | 805431 | A | G | 0.4617 | -0.0056 | 0.004 | 0.1584 | 3.09763E-05 | 24.94996305 |
| SmkInit | rs1565735 | 8 | 27426077 | A | T | 0.199801 | -0.0232 | 0.002 | 7.49E-32 | 805431 | A | T | 0.2084 | 0.0065 | 0.0049 | 0.1869 | 0.000167038 | 134.5596659 |
| SmkInit | rs160631 | 6 | 52895230 | G | T | 0.728628 | -0.0105 | 0.002 | 3.56E-09 | 805431 | G | T | 0.7301 | 0.0128 | 0.0044 | 0.00386696 | 3.42196E-05 | 27.56243156 |
| SmkInit | rs16896199 | 6 | 65787895 | T | A | 0.166998 | 0.0118 | 0.002 | 5.61E-09 | 805431 | T | A | 0.1811 | -0.0055 | 0.0052 | 0.2926 | 4.32172E-05 | 34.80991356 |
| SmkInit | rs16975171 | 18 | 39269650 | A | C | 0.10835 | -0.0172 | 0.003 | 5.29E-09 | 805431 | A | C | 0.0861 | 6.00E-04 | 0.0071 | 0.9281 | 4.08102E-05 | 32.87102949 |
| SmkInit | rs172032 | 19 | 18633755 | C | T | 0.425447 | 0.00901 | 0.002 | 1.56E-08 | 805431 | C | T | 0.4232 | -0.0112 | 0.0041 | 0.00560402 | 2.51971E-05 | 20.2949746 |
| SmkInit | rs1733756 | 10 | 56700221 | G | A | 0.49503 | 0.0101 | 0.002 | 1.23E-10 | 805431 | G | A | 0.5144 | 0.0068 | 0.0039 | 0.0828591 | 3.16622E-05 | 25.50243667 |
| SmkInit | rs17432775 | 2 | 63466463 | T | C | 0.224652 | -0.0105 | 0.002 | 2.55E-08 | 805431 | T | C | 0.223 | -0.0162 | 0.0047 | 0.0006188 | 3.42196E-05 | 27.56243156 |
| SmkInit | rs17594561 | 14 | 79618750 | G | A | 0.416501 | 0.00966 | 0.002 | 1.27E-09 | 805431 | G | A | 0.4362 | -0.0033 | 0.0041 | 0.4165 | 2.89637E-05 | 23.32884207 |
| SmkInit | rs1776631 | 10 | 31422577 | C | T | 0.774354 | -0.0106 | 0.002 | 3.92E-08 | 805431 | C | T | 0.7834 | -0.0141 | 0.0048 | 0.00311803 | 3.48745E-05 | 28.08993025 |
| SmkInit | rs192477591 | 20 | 19661225 | T | C | 0.500994 | 0.0116 | 0.002 | 1.73E-08 | 472832 | T | C | 0.5375 | 0.0109 | 0.0039 | 0.00564001 | 7.11407E-05 | 33.63985771 |
| SmkInit | rs1945737 | 18 | 53744545 | C | T | 0.514911 | -0.00968 | 0.002 | 8.19E-10 | 805431 | C | T | 0.5061 | 0.003 | 0.0039 | 0.4506 | 2.90837E-05 | 23.42554183 |
| SmkInit | rs1971318 | 12 | 121389500 | T | C | 0.129225 | 0.0156 | 0.002 | 7.80E-13 | 805431 | T | C | 0.1571 | 0.0046 | 0.0054 | 0.3977 | 7.55315E-05 | 60.83984893 |
| SmkInit | rs1994247 | 2 | 156023165 | T | G | 0.514911 | 0.0122 | 0.002 | 1.25E-14 | 805431 | T | G | 0.5312 | 0.0069 | 0.0039 | 0.0785001 | 4.61967E-05 | 37.2099076 |
| SmkInit | rs2022815 | 14 | 28356733 | A | G | 0.335984 | 0.0104 | 0.002 | 1.12E-09 | 805431 | A | G | 0.3281 | -0.0131 | 0.0042 | 0.00169801 | 3.3571E-05 | 27.03993286 |
| SmkInit | rs2072155 | 7 | 77762457 | C | T | 0.699801 | 0.0128 | 0.002 | 1.16E-13 | 805431 | C | T | 0.6953 | 0.009 | 0.0043 | 0.0358303 | 5.08522E-05 | 40.95989829 |
| SmkInit | rs2135160 | 2 | 213214467 | C | T | 0.0914513 | 0.0157 | 0.003 | 8.23E-09 | 748334 | C | T | 0.102 | -0.0187 | 0.0065 | 0.00413504 | 3.6597E-05 | 27.38770458 |
| SmkInit | rs2163413 | 2 | 226349200 | G | A | 0.175944 | -0.0138 | 0.002 | 3.06E-11 | 796935 | G | A | 0.1817 | -0.0137 | 0.0052 | 0.00781808 | 5.97378E-05 | 47.60988052 |
| SmkInit | rs2173019 | 5 | 167614971 | A | T | 0.170974 | 0.0131 | 0.002 | 3.47E-10 | 805431 | A | T | 0.1779 | -0.0046 | 0.0051 | 0.373 | 5.32637E-05 | 42.90239347 |
| SmkInit | rs2237303 | 7 | 21483605 | A | G | 0.656064 | -0.0102 | 0.002 | 6.37E-10 | 805431 | A | G | 0.6514 | -0.0041 | 0.0041 | 0.3169 | 3.22922E-05 | 26.00993541 |
| SmkInit | rs2289791 | 15 | 67476952 | T | G | 0.240557 | -0.0128 | 0.002 | 2.87E-12 | 805430 | T | G | 0.2491 | -0.0192 | 0.0046 | 3.38E-05 | 5.08522E-05 | 40.95989829 |
| SmkInit | rs2292239 | 12 | 56482180 | G | T | 0.666998 | 0.0108 | 0.002 | 1.03E-10 | 805431 | G | T | 0.6607 | 0.0018 | 0.0042 | 0.6664 | 3.62029E-05 | 29.15992759 |
| SmkInit | rs2313500 | 5 | 154808532 | T | C | 0.244533 | 0.0128 | 0.002 | 1.99E-12 | 805431 | T | C | 0.2452 | 0.0073 | 0.0046 | 0.1108 | 5.08522E-05 | 40.95989829 |
| SmkInit | rs2402821 | 7 | 126297947 | A | G | 0.62326 | 0.0103 | 0.002 | 3.51E-09 | 697342 | A | G | 0.6162 | 0.0062 | 0.004 | 0.1238 | 3.80323E-05 | 26.52242393 |
| SmkInit | rs2678903 | 2 | 58137930 | G | A | 0.591451 | 0.0104 | 0.002 | 1.25E-10 | 805431 | G | A | 0.6001 | 0.0073 | 0.004 | 0.06827 | 3.3571E-05 | 27.03993286 |
| SmkInit | rs2708630 | 1 | 8447404 | T | C | 0.691849 | -0.0102 | 0.002 | 9.99E-10 | 805431 | T | C | 0.6785 | -0.0036 | 0.0042 | 0.3978 | 3.22922E-05 | 26.00993541 |
| SmkInit | rs2711607 | 15 | 54117442 | T | G | 0.157058 | 0.0114 | 0.002 | 4.09E-08 | 805430 | T | G | 0.1627 | -0.0013 | 0.0053 | 0.8117 | 4.03371E-05 | 32.48991932 |
| SmkInit | rs2783130 | 13 | 80170160 | G | A | 0.502982 | -0.00877 | 0.002 | 2.71E-08 | 805431 | G | A | 0.4836 | 4.00E-04 | 0.0039 | 0.9118 | 2.38726E-05 | 19.22817725 |
| SmkInit | rs2876586 | 6 | 144862998 | A | G | 0.407555 | 0.00905 | 0.002 | 1.88E-08 | 805431 | A | G | 0.4075 | -0.0011 | 0.004 | 0.7837 | 2.54213E-05 | 20.47557416 |
| SmkInit | rs288181 | 5 | 107349285 | T | C | 0.317097 | -0.00923 | 0.002 | 4.11E-08 | 805431 | T | C | 0.3166 | -0.0077 | 0.0042 | 0.0685993 | 2.64426E-05 | 21.29817211 |
| SmkInit | rs2939756 | 11 | 41436297 | A | G | 0.489066 | -0.0103 | 0.002 | 6.87E-11 | 805431 | A | G | 0.4813 | 0.0042 | 0.0039 | 0.2834 | 3.29285E-05 | 26.52243414 |
| SmkInit | rs3110590 | 18 | 27808203 | A | C | 0.248509 | 0.0111 | 0.002 | 1.22E-09 | 805431 | A | C | 0.2493 | 0.0013 | 0.0046 | 0.7706 | 3.8242E-05 | 30.80242351 |
| SmkInit | rs3213876 | 18 | 73183978 | C | T | 0.315109 | 0.0115 | 0.002 | 5.08E-12 | 805431 | C | T | 0.3323 | -8.00E-04 | 0.0042 | 0.8496 | 4.10478E-05 | 33.0624179 |
| SmkInit | rs326341 | 3 | 107811142 | A | G | 0.462227 | -0.0101 | 0.002 | 6.16E-10 | 748334 | A | G | 0.4646 | 0.0061 | 0.004 | 0.1239 | 3.40779E-05 | 25.50243184 |
| SmkInit | rs332827 | 1 | 61743160 | A | G | 0.452286 | -0.00893 | 0.002 | 2.81E-08 | 780334 | A | G | 0.4563 | 0.0026 | 0.004 | 0.5183 | 2.55477E-05 | 19.9361739 |
| SmkInit | rs34367058 | 2 | 137513264 | T | C | 0.170974 | 0.0138 | 0.002 | 1.36E-13 | 805431 | T | C | 0.207 | 0.0046 | 0.0049 | 0.3483 | 5.91077E-05 | 47.60988178 |
| SmkInit | rs34488670 | 15 | 47684936 | C | T | 0.206759 | 0.0176 | 0.002 | 8.73E-20 | 805430 | C | T | 0.21 | -0.0025 | 0.0048 | 0.596901 | 9.61382E-05 | 77.43980771 |
| SmkInit | rs34638471 | 3 | 146367278 | A | G | 0.360835 | -0.00987 | 0.002 | 2.49E-08 | 697342 | A | G | 0.4106 | -0.0054 | 0.004 | 0.1763 | 3.49231E-05 | 24.35415515 |
| SmkInit | rs35891966 | 11 | 20129311 | A | G | 0.0715706 | -0.019 | 0.003 | 6.52E-10 | 805431 | A | G | 0.0788 | -0.0129 | 0.015 | 0.3901 | 4.97983E-05 | 40.11101151 |
| SmkInit | rs36116523 | 1 | 77088984 | A | G | 0.140159 | -0.0129 | 0.002 | 7.87E-09 | 805431 | A | G | 0.1424 | 0.0039 | 0.0056 | 0.4824 | 5.16498E-05 | 41.6023967 |
| SmkInit | rs3781295 | 10 | 104140602 | A | G | 0.387674 | -0.0116 | 0.002 | 1.13E-12 | 805431 | A | G | 0.3749 | -0.0085 | 0.0041 | 0.0363103 | 4.17647E-05 | 33.63991647 |
| SmkInit | rs3801289 | 7 | 96638267 | C | A | 0.363817 | -0.0115 | 0.002 | 3.64E-12 | 805431 | C | A | 0.3504 | -0.0096 | 0.0042 | 0.0218298 | 4.10478E-05 | 33.0624179 |
| SmkInit | rs3814994 | 7 | 88427385 | T | G | 0.373757 | 0.00962 | 0.002 | 7.44E-09 | 805431 | T | G | 0.3465 | 0.0048 | 0.0041 | 0.246 | 2.87243E-05 | 23.13604255 |
| SmkInit | rs3905125 | 1 | 236864459 | T | C | 0.542744 | 0.0104 | 0.002 | 4.28E-11 | 805431 | T | C | 0.5442 | 0.0046 | 0.004 | 0.2488 | 3.3571E-05 | 27.03993286 |
| SmkInit | rs3934797 | 4 | 112467612 | A | G | 0.171968 | -0.0151 | 0.002 | 1.90E-13 | 805431 | A | G | 0.1829 | -0.0096 | 0.0051 | 0.0617504 | 7.07677E-05 | 57.00235845 |
| SmkInit | rs39784 | 5 | 170559066 | A | C | 0.722664 | 0.0115 | 0.002 | 6.41E-11 | 805431 | A | C | 0.7238 | -0.0031 | 0.0044 | 0.4837 | 4.10478E-05 | 33.0624179 |
| SmkInit | rs404263 | 12 | 125805494 | T | C | 0.65507 | -0.0112 | 0.002 | 1.55E-10 | 697342 | T | C | 0.6295 | 0.0028 | 0.0041 | 0.4946 | 4.49687E-05 | 31.35991006 |
| SmkInit | rs4044321 | 5 | 166989513 | G | A | 0.668986 | -0.0164 | 0.002 | 3.04E-23 | 805431 | G | A | 0.6461 | 0.0028 | 0.0041 | 0.502599 | 8.34763E-05 | 67.23983303 |
| SmkInit | rs4374330 | 2 | 182058432 | T | C | 0.764414 | 0.0127 | 0.002 | 3.73E-12 | 805431 | T | C | 0.7564 | -0.0051 | 0.0046 | 0.2639 | 5.00608E-05 | 40.32239987 |
| SmkInit | rs4479577 | 3 | 5723818 | T | C | 0.498012 | 0.00898 | 0.002 | 2.11E-08 | 780334 | T | C | 0.4765 | 0.0036 | 0.004 | 0.3576 | 2.58346E-05 | 20.16004833 |
| SmkInit | rs4543592 | 9 | 3014254 | C | T | 0.49006 | 0.012 | 0.002 | 2.51E-14 | 805431 | C | T | 0.475 | 0.0037 | 0.004 | 0.3449 | 4.46946E-05 | 35.99991061 |
| SmkInit | rs4571506 | 5 | 87756918 | T | C | 0.481113 | -0.0151 | 0.002 | 1.64E-21 | 805431 | T | C | 0.4741 | -2.00E-04 | 0.0039 | 0.9537 | 7.07677E-05 | 57.00235845 |
| SmkInit | rs4579569 | 8 | 64913762 | A | G | 0.499006 | 0.0122 | 0.002 | 1.23E-14 | 805431 | A | G | 0.4774 | -0.0023 | 0.0039 | 0.5516 | 4.61967E-05 | 37.2099076 |
| SmkInit | rs465646 | 6 | 111620758 | A | G | 0.849901 | -0.0236 | 0.002 | 1.70E-28 | 805431 | A | G | 0.8407 | -0.0069 | 0.0054 | 0.1954 | 0.000172847 | 139.2396542 |
| SmkInit | rs4659805 | 1 | 237859755 | T | G | 0.605368 | 0.00936 | 0.002 | 8.36E-09 | 805431 | T | G | 0.6229 | 0.0063 | 0.0041 | 0.1195 | 2.71927E-05 | 21.90234561 |
| SmkInit | rs4751614 | 10 | 118696266 | T | A | 0.23161 | 0.0122 | 0.002 | 6.35E-11 | 805431 | T | A | 0.2345 | -0.0029 | 0.0046 | 0.5319 | 4.61967E-05 | 37.2099076 |
| SmkInit | rs4785187 | 16 | 49766772 | A | G | 0.244533 | 0.0119 | 0.002 | 2.03E-10 | 805431 | A | G | 0.2245 | -0.0119 | 0.0047 | 0.0120801 | 4.39528E-05 | 35.40241209 |
| SmkInit | rs4819027 | 21 | 46495224 | G | C | 0.718688 | -0.00989 | 0.002 | 1.34E-08 | 805431 | G | C | 0.7135 | -0.0084 | 0.0044 | 0.0558496 | 3.03593E-05 | 24.45296428 |
| SmkInit | rs4837631 | 9 | 122061948 | T | C | 0.457256 | -0.00949 | 0.002 | 3.54E-09 | 781945 | T | C | 0.4417 | 3.00E-04 | 0.004 | 0.9468 | 2.87928E-05 | 22.51496741 |
| SmkInit | rs4857114 | 3 | 94213083 | T | C | 0.406561 | 0.00881 | 0.002 | 3.03E-08 | 805431 | T | C | 0.4262 | -0.0108 | 0.004 | 0.00757495 | 2.40909E-05 | 19.40397682 |
| SmkInit | rs4888444 | 16 | 75690279 | G | A | 0.0397614 | -0.0245 | 0.004 | 6.84E-10 | 805431 | G | A | 0.0456 | -0.031 | 0.0095 | 0.00105 | 4.65762E-05 | 37.51553184 |
| SmkInit | rs4944844 | 11 | 73311705 | C | T | 0.84493 | -0.0133 | 0.002 | 2.33E-10 | 805431 | C | T | 0.8332 | 0.0105 | 0.0054 | 0.0513003 | 5.49024E-05 | 44.22239019 |
| SmkInit | rs4993466 | 3 | 114197912 | C | T | 0.723658 | 0.00983 | 0.002 | 3.89E-08 | 777869 | C | T | 0.7236 | -0.0092 | 0.009 | 0.3041 | 3.10547E-05 | 24.15716289 |
| SmkInit | rs540356 | 11 | 132203816 | A | C | 0.370775 | 0.0116 | 0.002 | 4.07E-13 | 805431 | A | C | 0.4011 | -0.0031 | 0.004 | 0.4368 | 4.17647E-05 | 33.63991647 |
| SmkInit | rs540860 | 11 | 121530888 | G | A | 0.562624 | 0.0117 | 0.002 | 3.18E-13 | 780334 | G | A | 0.5397 | 4.00E-04 | 0.0039 | 0.9151 | 4.38543E-05 | 34.22241229 |
| SmkInit | rs551739 | 12 | 40414075 | T | A | 0.694831 | 0.0102 | 0.002 | 4.42E-09 | 805431 | T | A | 0.7017 | 0.0059 | 0.0043 | 0.1733 | 3.22922E-05 | 26.00993541 |
| SmkInit | rs557544 | 6 | 109020332 | C | T | 0.356859 | -0.00945 | 0.002 | 2.82E-08 | 805431 | C | T | 0.3129 | -0.0123 | 0.0043 | 0.00388902 | 2.77181E-05 | 22.32556956 |
| SmkInit | rs55921136 | 1 | 210359333 | C | T | 0.240557 | -0.0136 | 0.002 | 1.90E-12 | 805431 | C | T | 0.2057 | 0.0134 | 0.0049 | 0.00595004 | 5.7407E-05 | 46.23988518 |
| SmkInit | rs55942317 | 16 | 49626772 | A | G | 0.0795229 | 0.0173 | 0.003 | 4.08E-11 | 805431 | A | G | 0.095 | 0.0094 | 0.0068 | 0.1674 | 4.12861E-05 | 33.25436187 |
| SmkInit | rs56169608 | 1 | 18439991 | A | G | 0.441352 | 0.00906 | 0.002 | 1.00E-08 | 805431 | A | G | 0.4637 | 3.00E-04 | 0.004 | 0.9379 | 2.54775E-05 | 20.52084904 |
| SmkInit | rs56225373 | 16 | 13761304 | G | T | 0.158052 | 0.016 | 0.003 | 3.13E-08 | 500394 | G | T | 0.1519 | 0.0025 | 0.0055 | 0.6482 | 5.68409E-05 | 28.44433076 |
| SmkInit | rs56348592 | 9 | 16749265 | G | A | 0.142147 | 0.0126 | 0.002 | 1.81E-08 | 799280 | G | A | 0.1561 | -0.0037 | 0.0055 | 0.498901 | 4.96547E-05 | 39.68990069 |
| SmkInit | rs56820925 | 20 | 54387374 | T | C | 0.354871 | -0.0108 | 0.002 | 2.25E-10 | 744041 | T | C | 0.3603 | -0.0054 | 0.0041 | 0.1872 | 3.91899E-05 | 29.15992162 |
| SmkInit | rs574835 | 11 | 64110668 | A | G | 0.365805 | -0.0103 | 0.002 | 2.33E-10 | 805431 | A | G | 0.3693 | -0.0105 | 0.0042 | 0.0126599 | 3.29285E-05 | 26.52243414 |
| SmkInit | rs58400863 | 4 | 31184484 | A | G | 0.385686 | -0.0114 | 0.002 | 5.03E-12 | 805431 | A | G | 0.3424 | -0.0044 | 0.0042 | 0.3018 | 4.0337E-05 | 32.48991932 |
| SmkInit | rs58547189 | 19 | 18462708 | A | G | 0.245527 | -0.0106 | 0.002 | 1.73E-08 | 732296 | A | G | 0.2672 | -0.0106 | 0.0049 | 0.02988 | 3.83573E-05 | 28.08992328 |
| SmkInit | rs60453921 | 10 | 87362579 | A | T | 0.150099 | 0.0127 | 0.002 | 1.06E-09 | 805431 | A | T | 0.1643 | 0.0063 | 0.0053 | 0.2368 | 5.00608E-05 | 40.32239987 |
| SmkInit | rs6088618 | 20 | 33409350 | A | G | 0.4334 | -0.0108 | 0.002 | 5.89E-11 | 744041 | A | G | 0.4543 | 0.0028 | 0.004 | 0.479001 | 3.91899E-05 | 29.15992162 |
| SmkInit | rs6141314 | 20 | 31093514 | A | G | 0.215706 | 0.0131 | 0.002 | 3.59E-13 | 801138 | A | G | 0.2531 | -0.0051 | 0.0046 | 0.2705 | 5.35491E-05 | 42.9023929 |
| SmkInit | rs61533748 | 2 | 22582968 | C | T | 0.377734 | 0.0104 | 0.002 | 1.50E-10 | 805431 | C | T | 0.3823 | 0.001 | 0.0041 | 0.8092 | 3.3571E-05 | 27.03993286 |
| SmkInit | rs61959481 | 13 | 55834929 | A | G | 0.203777 | -0.0123 | 0.002 | 2.06E-10 | 805431 | A | G | 0.2064 | -0.0018 | 0.0049 | 0.7061 | 4.69571E-05 | 37.82240608 |
| SmkInit | rs62107261 | 2 | 422144 | C | T | 0.0447316 | -0.0259 | 0.004 | 2.13E-11 | 805431 | C | T | 0.0464 | 0.0493 | 0.0182 | 0.00668606 | 5.20509E-05 | 41.92552089 |
| SmkInit | rs62135525 | 2 | 44299879 | T | C | 0.0387674 | -0.0221 | 0.004 | 4.61E-09 | 805431 | T | C | 0.0476 | -0.0049 | 0.0099 | 0.619901 | 3.78983E-05 | 30.5255492 |
| SmkInit | rs62181771 | 2 | 201042954 | A | G | 0.559642 | 0.00989 | 0.002 | 4.47E-10 | 805431 | A | G | 0.5632 | 0.0102 | 0.004 | 0.01073 | 3.03593E-05 | 24.45296428 |
| SmkInit | rs62250713 | 3 | 85513793 | G | A | 0.633201 | -0.0189 | 0.002 | 1.63E-30 | 805431 | G | A | 0.6293 | 0.0091 | 0.0041 | 0.0258399 | 0.000110863 | 89.30227825 |
| SmkInit | rs62254171 | 3 | 84624766 | A | G | 0.0149105 | 0.031 | 0.005 | 3.73E-09 | 800208 | A | G | 0.0245 | -0.006 | 0.0135 | 0.6556 | 4.80352E-05 | 38.43990392 |
| SmkInit | rs62258903 | 3 | 75102433 | G | C | 0.127237 | -0.0158 | 0.002 | 7.20E-12 | 805431 | G | C | 0.1359 | -0.0096 | 0.0058 | 0.1004 | 7.74805E-05 | 62.40984503 |
| SmkInit | rs62419578 | 6 | 84356959 | A | T | 0.122266 | 0.0131 | 0.002 | 1.08E-08 | 805431 | A | T | 0.1313 | -0.0045 | 0.0063 | 0.4758 | 5.32637E-05 | 42.90239347 |
| SmkInit | rs6464024 | 7 | 1688369 | T | C | 0.437376 | -0.0121 | 0.002 | 3.38E-14 | 805431 | T | C | 0.4144 | -0.0038 | 0.0041 | 0.3435 | 4.54425E-05 | 36.60240911 |
| SmkInit | rs6472232 | 8 | 66792632 | G | T | 0.375746 | -0.00942 | 0.002 | 1.43E-08 | 805431 | G | T | 0.3549 | 0.0025 | 0.0042 | 0.542199 | 2.75424E-05 | 22.18404491 |
| SmkInit | rs6497840 | 16 | 25351633 | A | G | 0.713718 | 0.0128 | 0.002 | 1.13E-12 | 748334 | A | G | 0.672 | 0.009 | 0.0044 | 0.0409902 | 5.47319E-05 | 40.95989053 |
| SmkInit | rs6598539 | 15 | 99204483 | C | T | 0.512922 | 0.011 | 0.002 | 5.76E-12 | 780333 | C | T | 0.5085 | 0.0076 | 0.0039 | 0.0530799 | 3.8764E-05 | 30.24992247 |
| SmkInit | rs66680800 | 3 | 85985324 | T | G | 0.430418 | -0.0125 | 0.002 | 1.13E-14 | 805431 | T | G | 0.3996 | 0.003 | 0.004 | 0.4584 | 4.84965E-05 | 39.062403 |
| SmkInit | rs6688826 | 1 | 80812329 | C | T | 0.302187 | 0.0109 | 0.002 | 3.93E-10 | 805431 | C | T | 0.2806 | -0.0051 | 0.0044 | 0.2395 | 3.68764E-05 | 29.70242624 |
| SmkInit | rs6705147 | 2 | 133196926 | T | C | 0.322068 | 0.00986 | 0.002 | 5.24E-09 | 805431 | T | C | 0.3028 | -0.0033 | 0.0045 | 0.472 | 3.01754E-05 | 24.30483965 |
| SmkInit | rs6728726 | 2 | 623976 | C | T | 0.83002 | 0.0187 | 0.002 | 4.27E-19 | 805431 | C | T | 0.822 | -0.0074 | 0.0051 | 0.148 | 0.000108529 | 87.42228292 |
| SmkInit | rs6861333 | 5 | 50703933 | G | C | 0.357853 | -0.0106 | 0.002 | 3.12E-10 | 748334 | G | C | 0.3812 | -0.0189 | 0.004 | 2.89E-06 | 3.75353E-05 | 28.08992493 |
| SmkInit | rs6868892 | 5 | 22193967 | T | C | 0.50497 | 0.0101 | 0.002 | 1.92E-08 | 624207 | T | C | 0.4966 | 0.0014 | 0.0039 | 0.7172 | 4.08542E-05 | 25.50241829 |
| SmkInit | rs6959670 | 7 | 110909169 | T | C | 0.355865 | 0.00987 | 0.002 | 7.29E-09 | 805431 | T | C | 0.3233 | 0.0018 | 0.0042 | 0.673501 | 3.02366E-05 | 24.35416452 |
| SmkInit | rs7092291 | 10 | 10040004 | T | C | 0.412525 | -0.00986 | 0.002 | 7.74E-10 | 805431 | T | C | 0.4103 | -0.0013 | 0.004 | 0.753199 | 3.01754E-05 | 24.30483965 |
| SmkInit | rs71367545 | 18 | 77576337 | A | G | 0.161034 | 0.0124 | 0.002 | 1.70E-10 | 805431 | A | G | 0.1985 | 0.0074 | 0.0049 | 0.1311 | 4.77237E-05 | 38.43990455 |
| SmkInit | rs71491832 | 11 | 124611997 | G | C | 0.0745527 | -0.0183 | 0.003 | 3.72E-10 | 805431 | G | C | 0.0714 | -0.0065 | 0.0077 | 0.3988 | 4.61967E-05 | 37.2099076 |
| SmkInit | rs71627577 | 5 | 43125795 | G | A | 0.119284 | -0.017 | 0.002 | 5.15E-12 | 805431 | G | A | 0.1182 | -0.0013 | 0.0115 | 0.9069 | 8.96955E-05 | 72.24982059 |
| SmkInit | rs7195043 | 16 | 90020861 | T | C | 0.444334 | -0.00883 | 0.002 | 2.13E-08 | 805431 | T | C | 0.459 | -0.0068 | 0.0041 | 0.0959489 | 2.42004E-05 | 19.4921766 |
| SmkInit | rs7205551 | 16 | 69605968 | A | G | 0.434394 | 0.0102 | 0.002 | 1.15E-09 | 732296 | A | G | 0.4372 | 0.0025 | 0.004 | 0.536701 | 3.55172E-05 | 26.00992896 |
| SmkInit | rs7224742 | 17 | 30657058 | T | C | 0.633201 | -0.011 | 0.002 | 1.02E-11 | 805431 | T | C | 0.6027 | -0.0067 | 0.004 | 0.0967498 | 3.75561E-05 | 30.24992488 |
| SmkInit | rs72664906 | 1 | 32170141 | C | T | 0.119284 | 0.0143 | 0.002 | 1.85E-09 | 748334 | C | T | 0.1354 | 0.0055 | 0.0059 | 0.3537 | 6.83104E-05 | 51.12236337 |
| SmkInit | rs72733235 | 9 | 38275772 | C | T | 0.177932 | 0.0127 | 0.002 | 1.86E-09 | 805431 | C | T | 0.1588 | 4.00E-04 | 0.0054 | 0.9353 | 5.00608E-05 | 40.32239987 |
| SmkInit | rs72789627 | 5 | 106826477 | T | C | 0.134195 | -0.0171 | 0.002 | 1.54E-13 | 805431 | T | C | 0.1381 | -0.0031 | 0.0057 | 0.5914 | 9.07537E-05 | 73.10231848 |
| SmkInit | rs72904370 | 11 | 46040015 | C | G | 0.217694 | -0.0108 | 0.002 | 1.59E-08 | 805431 | C | G | 0.2047 | 0.0142 | 0.0049 | 0.00361801 | 3.62029E-05 | 29.15992759 |
| SmkInit | rs7333559 | 13 | 100546450 | A | G | 0.791252 | -0.014 | 0.002 | 3.24E-13 | 805431 | A | G | 0.7826 | -0.0045 | 0.0049 | 0.3524 | 6.08333E-05 | 48.99987833 |
| SmkInit | rs745570 | 17 | 77781725 | G | A | 0.49503 | -0.0104 | 0.002 | 8.70E-11 | 780334 | G | A | 0.506 | 0.0023 | 0.0041 | 0.57 | 3.46506E-05 | 27.0399307 |
| SmkInit | rs76132272 | 1 | 227456065 | T | C | 0.055666 | 0.018 | 0.003 | 9.43E-09 | 748334 | T | C | 0.0702 | -0.0032 | 0.0077 | 0.6805 | 4.81045E-05 | 35.99990379 |
| SmkInit | rs7629352 | 3 | 16848835 | G | A | 0.296223 | 0.00986 | 0.002 | 7.82E-09 | 805431 | G | A | 0.3072 | 0.0064 | 0.0043 | 0.1347 | 3.01754E-05 | 24.30483965 |
| SmkInit | rs763053 | 16 | 735921 | C | T | 0.267396 | -0.0149 | 0.002 | 6.71E-16 | 799280 | C | T | 0.2321 | -0.0052 | 0.0049 | 0.2812 | 6.94358E-05 | 55.50236112 |
| SmkInit | rs76608582 | 19 | 4474725 | A | C | 0.0457256 | -0.0267 | 0.004 | 2.76E-13 | 799016 | A | C | 0.0496 | -0.0229 | 0.0107 | 0.03252 | 5.576E-05 | 44.55551347 |
| SmkInit | rs7666804 | 4 | 57758974 | C | T | 0.393638 | -0.0109 | 0.002 | 1.18E-08 | 573529 | C | T | 0.3941 | 0.0019 | 0.004 | 0.634 | 5.17863E-05 | 29.70239642 |
| SmkInit | rs77307359 | 4 | 34839767 | C | T | 0.21173 | -0.0112 | 0.002 | 2.07E-08 | 805431 | C | T | 0.1984 | -0.001 | 0.0049 | 0.8443 | 3.89342E-05 | 31.35992213 |
| SmkInit | rs7788527 | 7 | 70579487 | C | T | 0.686879 | 0.00952 | 0.002 | 1.58E-08 | 805431 | C | T | 0.6743 | 0.0016 | 0.0042 | 0.6968 | 2.81302E-05 | 22.65754374 |
| SmkInit | rs78175438 | 21 | 40663255 | C | T | 0.11829 | 0.0162 | 0.002 | 2.02E-11 | 805431 | C | T | 0.1253 | -0.0188 | 0.006 | 0.001893 | 8.14529E-05 | 65.60983708 |
| SmkInit | rs7829715 | 8 | 59803836 | C | T | 0.560636 | -0.0117 | 0.002 | 1.30E-13 | 805431 | C | T | 0.5226 | -0.0026 | 0.0039 | 0.5154 | 4.24879E-05 | 34.22241502 |
| SmkInit | rs7830359 | 8 | 10838461 | T | C | 0.350895 | -0.0103 | 0.002 | 3.88E-09 | 697342 | T | C | 0.3628 | 0.0049 | 0.0042 | 0.2415 | 3.80323E-05 | 26.52242393 |
| SmkInit | rs79222572 | 6 | 165108555 | G | T | 0.255467 | 0.0103 | 0.002 | 1.53E-08 | 805431 | G | T | 0.244 | 0.0095 | 0.0046 | 0.03724 | 3.29285E-05 | 26.52243414 |
| SmkInit | rs7929518 | 11 | 85980958 | G | A | 0.772366 | 0.0116 | 0.002 | 9.06E-10 | 805431 | G | A | 0.7692 | 0.0053 | 0.0047 | 0.2604 | 4.17647E-05 | 33.63991647 |
| SmkInit | rs7947391 | 11 | 66186882 | G | A | 0.577535 | 0.00929 | 0.002 | 7.55E-09 | 805431 | G | A | 0.5896 | -0.0037 | 0.004 | 0.358 | 2.67875E-05 | 21.57597142 |
| SmkInit | rs7969559 | 12 | 69655167 | G | A | 0.681909 | -0.011 | 0.002 | 3.28E-10 | 805431 | G | A | 0.7098 | -0.0172 | 0.0043 | 7.31E-05 | 3.75561E-05 | 30.24992488 |
| SmkInit | rs7984311 | 13 | 100741752 | A | G | 0.387674 | 0.00921 | 0.002 | 1.77E-08 | 805431 | A | G | 0.3657 | 0.0091 | 0.0041 | 0.0261102 | 2.63281E-05 | 21.20597234 |
| SmkInit | rs7986094 | 13 | 31029931 | C | A | 0.694831 | 0.00988 | 0.002 | 5.99E-09 | 805431 | C | A | 0.6989 | -0.0151 | 0.0043 | 0.0005188 | 3.02979E-05 | 24.4035394 |
| SmkInit | rs8001839 | 13 | 97110073 | G | A | 0.323062 | 0.0094 | 0.002 | 3.74E-08 | 780334 | G | A | 0.3342 | -0.0058 | 0.0042 | 0.1637 | 2.83076E-05 | 22.08994338 |
| SmkInit | rs8067305 | 17 | 1975657 | A | G | 0.574553 | 0.011 | 0.002 | 4.95E-12 | 805431 | A | G | 0.5791 | 0.0102 | 0.004 | 0.01027 | 3.75561E-05 | 30.24992488 |
| SmkInit | rs8069451 | 17 | 37504933 | C | T | 0.246521 | 0.0112 | 0.002 | 1.02E-09 | 805431 | C | T | 0.2558 | -0.0099 | 0.0045 | 0.0285299 | 3.89342E-05 | 31.35992213 |
| SmkInit | rs846781 | 6 | 101280434 | C | T | 0.737575 | -0.0115 | 0.002 | 4.80E-11 | 805431 | C | T | 0.7161 | -0.0033 | 0.0044 | 0.4523 | 4.10478E-05 | 33.0624179 |
| SmkInit | rs853684 | 6 | 28294550 | C | T | 0.380716 | -0.0116 | 0.002 | 8.55E-13 | 805431 | C | T | 0.3758 | -0.0121 | 0.0041 | 0.00298903 | 4.17647E-05 | 33.63991647 |
| SmkInit | rs888292 | 18 | 25210972 | T | A | 0.295229 | -0.0107 | 0.002 | 2.03E-09 | 748334 | T | A | 0.2952 | -6.00E-04 | 0.0044 | 0.8827 | 3.82468E-05 | 28.6224235 |
| SmkInit | rs911781 | 10 | 123966989 | G | A | 0.568588 | -0.00878 | 0.002 | 4.99E-08 | 780334 | G | A | 0.5537 | 0.002 | 0.004 | 0.614899 | 2.46966E-05 | 19.27205061 |
| SmkInit | rs9323328 | 14 | 58653514 | G | A | 0.526839 | -0.0108 | 0.002 | 7.25E-12 | 805431 | G | A | 0.5365 | -0.0175 | 0.004 | 1.22E-05 | 3.62029E-05 | 29.15992759 |
| SmkInit | rs9375371 | 6 | 98751680 | A | G | 0.293241 | 0.013 | 0.002 | 1.41E-13 | 805431 | A | G | 0.2766 | -0.0072 | 0.0044 | 0.1049 | 5.24536E-05 | 42.24989509 |
| SmkInit | rs9402093 | 6 | 129353671 | T | G | 0.691849 | 0.0101 | 0.002 | 3.38E-09 | 805431 | T | G | 0.6934 | -0.0033 | 0.0043 | 0.445 | 3.16622E-05 | 25.50243667 |
| SmkInit | rs9423279 | 10 | 125680419 | G | C | 0.638171 | -0.0108 | 0.002 | 9.64E-11 | 775111 | G | C | 0.6487 | -0.0125 | 0.0081 | 0.1256 | 3.7619E-05 | 29.15992476 |
| SmkInit | rs951740 | 1 | 44011737 | A | G | 0.636183 | 0.0172 | 0.002 | 3.78E-26 | 805431 | A | G | 0.6232 | -0.0071 | 0.0041 | 0.0813299 | 9.18182E-05 | 73.95981635 |
| SmkInit | rs9538536 | 13 | 60536321 | G | T | 0.668986 | -0.00936 | 0.002 | 4.28E-08 | 805431 | G | T | 0.6998 | 0.0023 | 0.0043 | 0.597601 | 2.71927E-05 | 21.90234561 |
| SmkInit | rs9541499 | 13 | 69261072 | G | T | 0.082505 | -0.0165 | 0.003 | 2.16E-08 | 752772 | G | T | 0.0771 | 0.0049 | 0.0073 | 0.499501 | 4.01832E-05 | 30.24991963 |
| SmkInit | rs9613472 | 22 | 27972479 | G | A | 0.541749 | 0.00867 | 0.002 | 4.02E-08 | 805431 | G | A | 0.5331 | 0.0023 | 0.0041 | 0.569 | 2.33313E-05 | 18.79217834 |
| SmkInit | rs9627272 | 22 | 46442288 | C | G | 0.389662 | -0.00991 | 0.002 | 1.28E-09 | 780334 | C | G | 0.4131 | -0.0077 | 0.0043 | 0.0740099 | 3.14625E-05 | 24.55196207 |
| SmkInit | rs963354 | 3 | 157393770 | A | C | 0.704771 | 0.0107 | 0.002 | 2.35E-10 | 805431 | A | C | 0.6933 | -0.0036 | 0.0043 | 0.399 | 3.55356E-05 | 28.62242893 |
| SmkInit | rs9679319 | 2 | 104136581 | G | T | 0.56163 | 0.0175 | 0.002 | 4.52E-23 | 640245 | G | T | 0.4625 | 5.00E-04 | 0.0039 | 0.8972 | 0.000119569 | 76.56226083 |
| SmkInit | rs9763225 | 5 | 60306287 | G | A | 0.26839 | 0.0139 | 0.002 | 5.38E-12 | 624207 | G | A | 0.2654 | -0.0043 | 0.0044 | 0.3373 | 7.73762E-05 | 48.30234524 |
| SmkInit | rs993700 | 4 | 67825894 | C | T | 0.767396 | -0.0141 | 0.002 | 1.00E-13 | 805431 | C | T | 0.7732 | -0.0021 | 0.0047 | 0.652299 | 6.17054E-05 | 49.70237658 |
| SmkInit | rs9987376 | 8 | 93190014 | G | T | 0.569583 | -0.0137 | 0.002 | 1.02E-17 | 805431 | G | T | 0.5711 | 0.0069 | 0.004 | 0.0814404 | 5.82542E-05 | 46.92238348 |
| AgeSmk | rs11080208 | 17 | 31554533 | C | T | 0.752485 | -0.0176 | 0.003 | 8.63E-10 | 323386 | C | T | 0.7328 | -0.001 | 0.0044 | 0.8145 | 0.000106418 | 34.41756492 |
| AgeSmk | rs11780471 | 8 | 27344719 | A | G | 0.0755467 | 0.0369 | 0.005 | 1.59E-12 | 323386 | A | G | 0.0646 | 0.0016 | 0.008 | 0.843 | 0.000168391 | 54.46406316 |
| AgeSmk | rs12575642 | 11 | 63979643 | T | G | 0.183897 | -0.0183 | 0.003 | 1.69E-08 | 323386 | T | G | 0.1895 | -0.0065 | 0.005 | 0.1966 | 0.00011505 | 37.20976987 |
| AgeSmk | rs241979 | 12 | 3834474 | A | G | 0.246521 | -0.0159 | 0.003 | 3.87E-08 | 323386 | A | G | 0.2534 | 0.0062 | 0.0045 | 0.1699 | 8.68546E-05 | 28.08982628 |
| AgeSmk | rs2491383 | 10 | 106616640 | G | T | 0.61829 | 0.0152 | 0.003 | 1.30E-08 | 290963 | G | T | 0.6022 | 3.00E-04 | 0.0041 | 0.947 | 8.82203E-05 | 25.67093465 |
| AgeSmk | rs4692334 | 4 | 28617013 | C | G | 0.803181 | 0.018 | 0.003 | 4.05E-08 | 323386 | C | G | 0.8202 | -0.0018 | 0.0051 | 0.7177 | 0.00011131 | 35.99977736 |
| AgeSmk | rs62180314 | 2 | 63373133 | C | G | 0.207753 | 0.0236 | 0.003 | 1.39E-14 | 323386 | C | G | 0.208 | -0.0182 | 0.0049 | 0.0001704 | 0.000191327 | 61.88406172 |
| AgeSmk | rs624833 | 4 | 2881256 | G | T | 0.328032 | 0.0158 | 0.003 | 4.98E-09 | 323386 | G | T | 0.3096 | -0.0012 | 0.0043 | 0.773599 | 8.57656E-05 | 27.73760623 |
| CigDay | rs11076320 | 16 | 52097552 | A | C | 0.632207 | -0.0164 | 0.003 | 1.06E-10 | 324557 | A | C | 0.6097 | 0.0053 | 0.004 | 0.1851 | 9.20692E-05 | 29.88426029 |
| CigDay | rs112178027 | 17 | 27564013 | T | C | 0.181909 | 0.0205 | 0.003 | 8.02E-10 | 326497 | T | C | 0.1745 | 0.0032 | 0.0053 | 0.5373 | 0.000142996 | 46.69415841 |
| CigDay | rs11663346 | 18 | 62152458 | T | A | 0.592445 | -0.0141 | 0.003 | 1.75E-08 | 326497 | T | A | 0.5828 | -0.0031 | 0.004 | 0.4435 | 6.7653E-05 | 22.08986468 |
| CigDay | rs11686893 | 2 | 148495050 | C | T | 0.286282 | 0.0165 | 0.003 | 3.28E-10 | 326497 | C | T | 0.3201 | 0.0074 | 0.0043 | 0.0830902 | 9.26416E-05 | 30.2498147 |
| CigDay | rs11725618 | 4 | 67053769 | C | T | 0.313121 | 0.0181 | 0.003 | 5.71E-11 | 326497 | C | T | 0.2887 | -0.0016 | 0.0044 | 0.719201 | 0.000111477 | 36.40088813 |
| CigDay | rs11928552 | 3 | 48739883 | C | T | 0.651093 | -0.0166 | 0.003 | 2.71E-09 | 280613 | C | T | 0.6334 | -0.0152 | 0.0042 | 0.000335197 | 0.000109098 | 30.61755956 |
| CigDay | rs11940430 | 4 | 99509453 | A | T | 0.654076 | -0.0146 | 0.003 | 2.94E-08 | 326497 | A | T | 0.6594 | 0.0024 | 0.0042 | 0.5659 | 7.25358E-05 | 23.68429936 |
| CigDay | rs12660603 | 6 | 97970749 | C | T | 0.0437376 | -0.0303 | 0.006 | 4.24E-08 | 300003 | C | T | 0.0516 | -0.0068 | 0.0092 | 0.4573 | 8.50003E-05 | 25.50232999 |
| CigDay | rs13254578 | 8 | 42545846 | C | G | 0.777336 | 0.0353 | 0.003 | 4.81E-33 | 326497 | C | G | 0.7715 | -0.0042 | 0.0047 | 0.3722 | 0.000423881 | 138.4535963 |
| CigDay | rs138759397 | 15 | 79011488 | C | G | 0.0258449 | 0.0628 | 0.009 | 5.71E-13 | 325689 | C | G | 0.0226 | 0.02 | 0.0292 | 0.4931 | 0.000149474 | 48.68908372 |
| CigDay | rs141147481 | 15 | 78835239 | G | C | 0.0228628 | -0.0588 | 0.009 | 3.61E-10 | 325625 | G | C | 0.0201 | -0.0373 | 0.0279 | 0.1816 | 0.000131067 | 42.68418228 |
| CigDay | rs1444026 | 8 | 137545826 | G | T | 0.446322 | 0.014 | 0.002 | 2.15E-08 | 326497 | G | T | 0.4344 | 0.0024 | 0.004 | 0.552901 | 0.000150055 | 48.99969984 |
| CigDay | rs145483821 | 3 | 89347020 | G | T | 0.439364 | -0.0165 | 0.003 | 1.41E-08 | 238603 | G | T | 0.4575 | -0.002 | 0.004 | 0.619599 | 0.000126764 | 30.24974644 |
| CigDay | rs1657936 | 15 | 57113013 | T | C | 0.781312 | -0.0189 | 0.003 | 6.52E-10 | 326497 | T | C | 0.7918 | 0.0071 | 0.0049 | 0.1438 | 0.000121548 | 39.68975687 |
| CigDay | rs17197116 | 11 | 46520302 | C | T | 0.084493 | 0.0298 | 0.005 | 4.52E-11 | 326497 | C | T | 0.0803 | 0.0087 | 0.0072 | 0.2285 | 0.000108784 | 35.52138241 |
| CigDay | rs1737894 | 20 | 31054702 | G | C | 0.377734 | 0.0192 | 0.003 | 1.69E-14 | 326497 | G | C | 0.3947 | -0.0023 | 0.0041 | 0.569101 | 0.000125437 | 40.95974909 |
| CigDay | rs185771419 | 15 | 79120489 | T | G | 0.0238569 | -0.054 | 0.007 | 1.20E-13 | 325812 | T | G | 0.0314 | -0.0276 | 0.0258 | 0.2851 | 0.000182619 | 59.50983878 |
| CigDay | rs2060220 | 5 | 166574542 | T | A | 0.133201 | 0.0201 | 0.004 | 2.16E-08 | 326497 | T | A | 0.134 | -2.00E-04 | 0.0058 | 0.9748 | 7.7332E-05 | 25.25047032 |
| CigDay | rs2072659 | 1 | 154548521 | G | C | 0.101392 | -0.0301 | 0.004 | 6.44E-13 | 324557 | G | C | 0.1035 | -0.0121 | 0.0071 | 0.0885605 | 0.00017444 | 56.62527606 |
| CigDay | rs2133203 | 1 | 77981099 | T | C | 0.450298 | -0.0162 | 0.003 | 2.42E-09 | 280416 | T | C | 0.4271 | 0.002 | 0.004 | 0.611299 | 0.000103978 | 29.15979202 |
| CigDay | rs215600 | 7 | 32333642 | A | G | 0.667992 | -0.0225 | 0.003 | 2.81E-18 | 326497 | A | G | 0.6469 | -2.00E-04 | 0.0041 | 0.9519 | 0.000172254 | 56.24965543 |
| CigDay | rs2273500 | 20 | 61986949 | C | T | 0.146123 | 0.035 | 0.003 | 7.09E-24 | 320237 | C | T | 0.1466 | -0.0026 | 0.0058 | 0.6549 | 0.000424852 | 136.110261 |
| CigDay | rs2655008 | 3 | 136123775 | T | A | 0.730616 | 0.0161 | 0.003 | 1.78E-08 | 326497 | T | A | 0.7424 | -0.0054 | 0.0045 | 0.2314 | 8.82047E-05 | 28.80093469 |
| CigDay | rs3025383 | 9 | 136502369 | C | T | 0.180915 | -0.0307 | 0.003 | 6.01E-22 | 326497 | C | T | 0.185 | 0.0027 | 0.0052 | 0.6042 | 0.000320639 | 104.7204696 |
| CigDay | rs34370696 | 1 | 166860052 | T | C | 0.175944 | -0.0192 | 0.003 | 2.36E-08 | 324557 | T | C | 0.163 | -0.0087 | 0.0054 | 0.1066 | 0.000126187 | 40.95974759 |
| CigDay | rs34406232 | 19 | 41305530 | A | C | 0.0258449 | -0.0796 | 0.007 | 1.21E-26 | 325689 | A | C | 0.0256 | 0.0225 | 0.0125 | 0.0728098 | 0.000396876 | 129.3085937 |
| CigDay | rs3796462 | 4 | 96108829 | T | C | 0.701789 | -0.0147 | 0.003 | 3.11E-08 | 326497 | T | C | 0.687 | -0.0105 | 0.0042 | 0.0127201 | 7.35328E-05 | 24.00985292 |
| CigDay | rs56113850 | 19 | 41353107 | C | T | 0.592445 | 0.0622 | 0.002 | 1.36E-137 | 326497 | C | T | 0.5759 | 0.005 | 0.004 | 0.209 | 0.002953636 | 967.2040752 |
| CigDay | rs6078372 | 20 | 11860291 | A | G | 0.420477 | 0.0153 | 0.003 | 1.22E-09 | 326497 | A | G | 0.4157 | -0.0064 | 0.004 | 0.106 | 7.96575E-05 | 26.00984067 |
| CigDay | rs669696 | 16 | 69657996 | A | C | 0.423459 | -0.0194 | 0.003 | 1.69E-11 | 246711 | A | C | 0.4221 | -0.001 | 0.004 | 0.8077 | 0.000169472 | 41.81743878 |
| CigDay | rs6699355 | 1 | 35384605 | T | C | 0.877734 | -0.0207 | 0.004 | 3.05E-08 | 322348 | T | C | 0.8794 | -0.0054 | 0.0063 | 0.3939 | 8.3073E-05 | 26.78045884 |
| CigDay | rs6831786 | 4 | 67875548 | A | C | 0.558648 | -0.0161 | 0.003 | 6.51E-10 | 300003 | A | C | 0.5671 | 0.0057 | 0.0041 | 0.1633 | 9.59935E-05 | 28.80091911 |
| CigDay | rs72738704 | 15 | 78719832 | C | G | 0.370775 | 0.0863 | 0.003 | 1.00E-200 | 326497 | C | G | 0.3328 | -0.0099 | 0.0042 | 0.0189898 | 0.002528137 | 827.516042 |
| CigDay | rs72976960 | 19 | 4029784 | G | A | 0.184891 | -0.0207 | 0.003 | 1.46E-09 | 319050 | G | A | 0.1654 | 4.00E-04 | 0.0055 | 0.9433 | 0.000149202 | 47.60970155 |
| CigDay | rs73229090 | 8 | 27442127 | A | C | 0.11332 | 0.0304 | 0.004 | 1.65E-14 | 326497 | A | C | 0.1249 | -0.0013 | 0.0061 | 0.8309 | 0.000176877 | 57.75964618 |
| CigDay | rs7599488 | 2 | 60718347 | T | C | 0.417495 | 0.0144 | 0.002 | 8.42E-09 | 326497 | T | C | 0.4242 | -0.0137 | 0.004 | 0.000606904 | 0.000158751 | 51.83968245 |
| CigDay | rs7678019 | 4 | 2997194 | A | G | 0.378728 | -0.018 | 0.003 | 9.60E-11 | 281992 | A | G | 0.3565 | -0.0028 | 0.0041 | 0.4912 | 0.000127647 | 35.99974467 |
| CigDay | rs7802341 | 7 | 74358919 | A | T | 0.746521 | -0.0223 | 0.004 | 1.82E-08 | 167512 | A | T | 0.738 | 0.0162 | 0.0093 | 0.0815699 | 0.000185508 | 31.08025391 |
| CigDay | rs7874404 | 9 | 136439803 | T | C | 0.213718 | -0.02 | 0.003 | 6.64E-10 | 300003 | T | C | 0.1912 | -0.017 | 0.013 | 0.1925 | 0.000148125 | 44.44414815 |
| CigDay | rs79250609 | 12 | 109988858 | T | C | 0.176938 | -0.0189 | 0.003 | 7.93E-09 | 326497 | T | C | 0.1631 | -0.007 | 0.0058 | 0.2218 | 0.000121548 | 39.68975687 |
| CigDay | rs7928017 | 11 | 113448762 | A | C | 0.407555 | -0.0176 | 0.002 | 2.22E-12 | 326497 | A | C | 0.433 | 0.007 | 0.004 | 0.0776909 | 0.000237128 | 77.43952563 |
| CigDay | rs7933830 | 11 | 16377119 | T | C | 0.319085 | 0.0194 | 0.003 | 5.23E-13 | 326497 | T | C | 0.3169 | -8.00E-04 | 0.0042 | 0.8507 | 0.000128064 | 41.81752162 |
| CigDay | rs7944241 | 11 | 43695374 | G | A | 0.409543 | -0.0156 | 0.003 | 4.59E-10 | 326497 | G | A | 0.4193 | 0.0121 | 0.004 | 0.00254501 | 8.28117E-05 | 27.03983436 |
| CigDay | rs8021229 | 14 | 104157578 | T | C | 0.296223 | 0.0176 | 0.003 | 2.14E-11 | 326497 | T | C | 0.3273 | -0.0058 | 0.0042 | 0.1646 | 0.000105404 | 34.41756695 |
| CigDay | rs80292109 | 6 | 26237068 | G | A | 0.295229 | -0.0194 | 0.003 | 1.24E-11 | 300003 | G | A | 0.2998 | 0.0133 | 0.0044 | 0.00255 | 0.000139372 | 41.817499 |
| CigDay | rs806255 | 8 | 64619016 | A | G | 0.732604 | -0.0197 | 0.003 | 5.53E-12 | 326497 | A | G | 0.7449 | 0.0017 | 0.0045 | 0.701799 | 0.000132055 | 43.12084697 |
| CigDay | rs9881798 | 3 | 16846967 | C | A | 0.39165 | 0.0147 | 0.003 | 4.47E-09 | 326497 | C | A | 0.4105 | 0.004 | 0.004 | 0.3154 | 7.35328E-05 | 24.00985292 |
| SmkCes | rs1009181 | 6 | 26158993 | C | T | 0.33499 | -0.013 | 0.002 | 3.71E-08 | 388313 | C | T | 0.3711 | 0.0115 | 0.0041 | 0.00476705 | 0.000108792 | 42.24978239 |
| SmkCes | rs10182249 | 2 | 22964436 | A | G | 0.646123 | -0.0136 | 0.002 | 1.61E-08 | 388313 | A | G | 0.6677 | 0.0012 | 0.0042 | 0.772899 | 0.000119065 | 46.23976184 |
| SmkCes | rs10402271 | 19 | 45329214 | G | T | 0.34493 | -0.0132 | 0.002 | 4.30E-08 | 388313 | G | T | 0.3286 | -0.0019 | 0.0042 | 0.6494 | 0.000112165 | 43.55977564 |
| SmkCes | rs10821537 | 9 | 136486824 | G | A | 0.652087 | -0.0153 | 0.002 | 1.58E-10 | 375952 | G | A | 0.6355 | -0.0125 | 0.0095 | 0.1872 | 0.000155641 | 58.52218867 |
| SmkCes | rs113382419 | 9 | 136463019 | A | C | 0.0904573 | 0.0501 | 0.004 | 4.96E-41 | 388313 | A | C | 0.1039 | 0.004 | 0.0064 | 0.5319 | 0.00040383 | 156.874817 |
| SmkCes | rs11697662 | 20 | 61992005 | T | C | 0.804175 | -0.0301 | 0.003 | 2.26E-25 | 378958 | T | C | 0.8069 | 0.0043 | 0.0053 | 0.41 | 0.000265573 | 100.6672465 |
| SmkCes | rs11991338 | 8 | 9303761 | A | G | 0.170974 | 0.0182 | 0.003 | 2.31E-08 | 333791 | A | G | 0.1534 | -0.0108 | 0.0055 | 0.0489903 | 0.00011025 | 36.80422392 |
| SmkCes | rs2006281 | 14 | 104327732 | T | C | 0.534791 | -0.0139 | 0.002 | 8.11E-10 | 388313 | T | C | 0.5109 | 0.008 | 0.004 | 0.0458902 | 0.000124375 | 48.30225122 |
| SmkCes | rs2118362 | 11 | 16373083 | C | T | 0.286282 | 0.0153 | 0.003 | 2.19E-09 | 388313 | C | T | 0.2851 | 0.0016 | 0.0044 | 0.7191 | 6.69776E-05 | 26.00986604 |
| SmkCes | rs215600 | 7 | 32333642 | A | G | 0.667992 | -0.0144 | 0.002 | 1.50E-09 | 388313 | A | G | 0.6469 | -2.00E-04 | 0.0041 | 0.9519 | 0.000133483 | 51.839733 |
| SmkCes | rs35033100 | 20 | 61726373 | C | T | 0.0417495 | -0.0307 | 0.005 | 2.31E-08 | 378958 | C | T | 0.0406 | -4.00E-04 | 0.0109 | 0.9683 | 9.94724E-05 | 37.69940104 |
| SmkCes | rs4705014 | 5 | 155852315 | A | G | 0.661034 | -0.0132 | 0.002 | 1.98E-08 | 388313 | A | G | 0.6446 | 0.0034 | 0.0041 | 0.4055 | 0.000112165 | 43.55977564 |
| SmkCes | rs56049603 | 3 | 49585243 | G | C | 0.202783 | 0.0159 | 0.003 | 8.42E-09 | 388313 | G | C | 0.2199 | 0.0063 | 0.0048 | 0.1833 | 7.23333E-05 | 28.08985532 |
| SmkCes | rs56113850 | 19 | 41353107 | C | T | 0.592445 | -0.033 | 0.002 | 3.85E-47 | 388313 | C | T | 0.5759 | 0.005 | 0.004 | 0.209 | 0.000700618 | 272.2485978 |
| SmkCes | rs591143 | 15 | 47647755 | T | C | 0.629225 | -0.0144 | 0.002 | 5.00E-10 | 388312 | T | C | 0.5973 | 0.0027 | 0.004 | 0.4987 | 0.000133483 | 51.839733 |
| SmkCes | rs60749569 | 8 | 42602668 | T | A | 0.0805169 | -0.0266 | 0.004 | 5.38E-10 | 355742 | T | A | 0.0799 | 0.0081 | 0.0077 | 0.2915 | 0.000124295 | 44.22225138 |
| SmkCes | rs6816088 | 4 | 35563786 | C | T | 0.153082 | 0.0217 | 0.003 | 3.24E-10 | 288485 | C | T | 0.1704 | -0.0031 | 0.0053 | 0.5573 | 0.000181332 | 52.32074838 |
| SmkCes | rs71563618 | 7 | 114837804 | G | A | 0.084493 | 0.0207 | 0.004 | 6.91E-09 | 388313 | G | A | 0.1038 | -0.0198 | 0.0065 | 0.002227 | 6.89618E-05 | 26.78048707 |
| SmkCes | rs72740955 | 15 | 78849779 | T | C | 0.373757 | 0.0252 | 0.002 | 1.30E-25 | 388312 | T | C | 0.342 | -0.0093 | 0.0041 | 0.0249402 | 0.000408679 | 158.7591823 |
| SmkCes | rs72781639 | 2 | 24204148 | G | C | 0.16004 | 0.0203 | 0.003 | 1.12E-09 | 367191 | G | C | 0.1482 | 0.006 | 0.0055 | 0.2751 | 0.000124682 | 45.78752838 |
| SmkCes | rs7807019 | 7 | 117543063 | G | A | 0.470179 | 0.0147 | 0.002 | 1.00E-10 | 388313 | G | A | 0.4734 | -0.0051 | 0.004 | 0.1954 | 0.000139102 | 54.02222176 |
| SmkCes | rs9607805 | 22 | 41854446 | T | C | 0.702783 | 0.0189 | 0.003 | 8.88E-14 | 388313 | T | C | 0.7225 | 5.00E-04 | 0.0044 | 0.9109 | 0.000102201 | 39.68979558 |
| DrnkWk | rs1004787 | 2 | 45159091 | A | G | 0.535785 | 0.0171 | 0.002 | 1.13E-22 | 666978 | A | G | 0.55 | -0.0035 | 0.004 | 0.374 | 0.000109591 | 73.10228079 |
| DrnkWk | rs1011392 | 18 | 53041903 | G | A | 0.331014 | -0.0104 | 0.002 | 1.78E-08 | 666978 | G | A | 0.3263 | 0.0055 | 0.0042 | 0.1866 | 4.05394E-05 | 27.03991892 |
| DrnkWk | rs10236149 | 7 | 98977515 | G | A | 0.137177 | -0.0163 | 0.003 | 4.40E-10 | 666978 | G | A | 0.1237 | -0.0086 | 0.006 | 0.1526 | 4.4259E-05 | 29.52102259 |
| DrnkWk | rs10276148 | 7 | 103733264 | A | G | 0.530815 | 0.0117 | 0.002 | 1.19E-11 | 666978 | A | G | 0.5062 | -0.0022 | 0.004 | 0.5861 | 5.13072E-05 | 34.22239738 |
| DrnkWk | rs10743083 | 11 | 8637926 | G | A | 0.815109 | -0.015 | 0.002 | 1.64E-10 | 611077 | G | A | 0.1784 | -0.001 | 0.0052 | 0.8487 | 9.20421E-05 | 56.2498159 |
| DrnkWk | rs10753661 | 1 | 165119792 | A | G | 0.738569 | -0.0113 | 0.002 | 1.25E-09 | 665054 | A | G | 0.6869 | 0.0017 | 0.0042 | 0.6836 | 4.79976E-05 | 31.922404 |
| DrnkWk | rs10956823 | 8 | 93140909 | T | G | 0.776342 | 0.012 | 0.002 | 8.38E-09 | 666978 | T | G | 0.7778 | 0.0136 | 0.0048 | 0.00463703 | 5.39719E-05 | 35.99989205 |
| DrnkWk | rs11039216 | 11 | 47406592 | T | C | 0.519881 | 0.0155 | 0.002 | 3.76E-19 | 666978 | T | C | 0.5286 | -0.0117 | 0.004 | 0.00306803 | 9.00436E-05 | 60.0623199 |
| DrnkWk | rs11075711 | 16 | 69266456 | T | C | 0.16501 | -0.0134 | 0.002 | 1.31E-08 | 609153 | T | C | 0.1796 | -0.0035 | 0.0052 | 0.4996 | 7.36871E-05 | 44.88985262 |
| DrnkWk | rs111203819 | 4 | 18371143 | G | T | 0.485089 | 0.0101 | 0.002 | 2.41E-08 | 611077 | G | T | 0.4922 | -0.0044 | 0.004 | 0.2788 | 4.1732E-05 | 25.50241653 |
| DrnkWk | rs11607622 | 11 | 113526665 | T | C | 0.100398 | 0.0148 | 0.003 | 2.22E-08 | 666978 | T | C | 0.1143 | 2.00E-04 | 0.0062 | 0.9733 | 3.64883E-05 | 24.3377048 |
| DrnkWk | rs11860773 | 16 | 73912503 | C | T | 0.184891 | -0.0157 | 0.002 | 1.04E-12 | 665054 | C | T | 0.1862 | 0.012 | 0.0052 | 0.0206201 | 9.26493E-05 | 61.62231468 |
| DrnkWk | rs11940694 | 4 | 39414993 | G | A | 0.611332 | 0.0279 | 0.002 | 9.51E-56 | 666978 | G | A | 0.5875 | -0.0103 | 0.0041 | 0.0114699 | 0.000291682 | 194.6019165 |
| DrnkWk | rs11943397 | 4 | 143617304 | C | T | 0.62326 | 0.0122 | 0.002 | 1.23E-11 | 666978 | C | T | 0.6238 | -2.00E-04 | 0.0041 | 0.9532 | 5.57858E-05 | 37.20988842 |
| DrnkWk | rs12121630 | 1 | 50977279 | A | G | 0.149105 | -0.0136 | 0.002 | 1.59E-08 | 665054 | A | G | 0.1582 | 0.007 | 0.0054 | 0.198 | 6.95234E-05 | 46.23986094 |
| DrnkWk | rs12646808 | 4 | 3249828 | C | T | 0.348907 | -0.011 | 0.002 | 2.47E-09 | 666978 | C | T | 0.3499 | 2.00E-04 | 0.0043 | 0.9584 | 4.53518E-05 | 30.24990929 |
| DrnkWk | rs13024996 | 2 | 144225215 | A | C | 0.359841 | -0.0136 | 0.002 | 5.18E-14 | 666978 | A | C | 0.3633 | 0.0071 | 0.0041 | 0.08342 | 6.93228E-05 | 46.23986134 |
| DrnkWk | rs13107325 | 4 | 103188709 | T | C | 0.0795229 | -0.0393 | 0.004 | 2.86E-28 | 666978 | T | C | 0.0795 | 0.01 | 0.0073 | 0.1741 | 0.000144707 | 96.53033554 |
| DrnkWk | rs13236841 | 7 | 69389859 | G | A | 0.235586 | -0.014 | 0.002 | 1.92E-12 | 666978 | G | A | 0.2536 | -0.0042 | 0.0045 | 0.3541 | 7.34603E-05 | 48.99985307 |
| DrnkWk | rs13288470 | 9 | 129688559 | T | A | 0.11332 | -0.016 | 0.003 | 4.05E-09 | 666978 | T | A | 0.1191 | -0.0032 | 0.0061 | 0.6074 | 4.26449E-05 | 28.44435915 |
| DrnkWk | rs13332432 | 16 | 85721809 | G | C | 0.293241 | 0.0129 | 0.002 | 1.64E-11 | 665054 | G | C | 0.2937 | 3.00E-04 | 0.0044 | 0.9451 | 6.25512E-05 | 41.60237489 |
| DrnkWk | rs147431626 | 17 | 44357351 | A | G | 0.249503 | -0.0294 | 0.002 | 7.52E-32 | 463556 | A | G | 0.2123 | 0.0276 | 0.009 | 0.00214398 | 0.00046594 | 216.0890677 |
| DrnkWk | rs147711594 | 3 | 100893355 | T | G | 0.0248509 | -0.0298 | 0.005 | 2.91E-08 | 660241 | T | G | 0.0275 | 0.0185 | 0.0126 | 0.1423 | 5.37981E-05 | 35.5214924 |
| DrnkWk | rs148390057 | 18 | 37719792 | T | C | 0.439364 | -0.0111 | 0.002 | 2.91E-08 | 510881 | T | C | 0.4463 | -0.0092 | 0.0041 | 0.0240398 | 6.02893E-05 | 30.80237941 |
| DrnkWk | rs153106 | 16 | 28526897 | C | T | 0.354871 | -0.0155 | 0.002 | 9.38E-19 | 665054 | C | T | 0.4105 | -0.002 | 0.004 | 0.6148 | 9.03041E-05 | 60.06231938 |
| DrnkWk | rs16854020 | 4 | 42117559 | A | G | 0.11829 | 0.0189 | 0.003 | 6.28E-13 | 666978 | A | G | 0.1236 | -0.0085 | 0.006 | 0.1527 | 5.95037E-05 | 39.68988099 |
| DrnkWk | rs17884691 | 22 | 46481623 | A | G | 0.232604 | -0.0114 | 0.002 | 2.91E-08 | 645837 | A | G | 0.2354 | -0.0054 | 0.005 | 0.2804 | 5.03043E-05 | 32.48989939 |
| DrnkWk | rs1838420 | 8 | 64960855 | T | G | 0.503976 | 0.00981 | 0.002 | 1.53E-08 | 666978 | T | G | 0.4801 | -7.00E-04 | 0.0039 | 0.8634 | 3.60704E-05 | 24.05895286 |
| DrnkWk | rs1906252 | 6 | 98550289 | A | C | 0.50497 | 0.00972 | 0.002 | 2.05E-08 | 666978 | A | C | 0.4873 | -0.0043 | 0.0039 | 0.2697 | 3.54116E-05 | 23.61952917 |
| DrnkWk | rs1942964 | 18 | 55027212 | G | T | 0.49503 | -0.0107 | 0.002 | 3.89E-09 | 611077 | G | T | 0.4935 | 0.0031 | 0.0041 | 0.4478 | 4.68372E-05 | 28.62240632 |
| DrnkWk | rs1971157 | 17 | 27925343 | C | G | 0.394632 | 0.00987 | 0.002 | 2.74E-08 | 666978 | C | G | 0.3835 | -0.0029 | 0.0043 | 0.5002 | 3.6513E-05 | 24.35415197 |
| DrnkWk | rs2087975 | 12 | 92074004 | G | A | 0.616302 | -0.0107 | 0.002 | 2.02E-09 | 666978 | G | A | 0.6086 | 9.00E-04 | 0.0041 | 0.8192 | 4.29119E-05 | 28.62241417 |
| DrnkWk | rs2093186 | 1 | 16000106 | T | C | 0.244533 | -0.0122 | 0.002 | 1.01E-09 | 665054 | T | C | 0.2586 | 2.00E-04 | 0.0045 | 0.9675 | 5.59472E-05 | 37.2098881 |
| DrnkWk | rs2310752 | 1 | 66392405 | A | G | 0.428429 | -0.00977 | 0.002 | 2.57E-08 | 665054 | A | G | 0.4309 | 0.0021 | 0.004 | 0.605399 | 3.58803E-05 | 23.86315324 |
| DrnkWk | rs2424645 | 20 | 24720026 | G | A | 0.348907 | -0.0117 | 0.002 | 1.15E-10 | 666978 | G | A | 0.3527 | -0.0023 | 0.0042 | 0.5781 | 5.13072E-05 | 34.22239738 |
| DrnkWk | rs2533126 | 7 | 153473086 | A | G | 0.471173 | 0.0133 | 0.002 | 1.98E-14 | 666978 | A | G | 0.4858 | 0.0082 | 0.004 | 0.0398703 | 6.62984E-05 | 44.22236739 |
| DrnkWk | rs28616142 | 15 | 86867867 | T | C | 0.418489 | 0.0112 | 0.002 | 2.99E-10 | 666977 | T | C | 0.4056 | -0.0028 | 0.004 | 0.4865 | 4.70159E-05 | 31.35990596 |
| DrnkWk | rs28680958 | 1 | 173848808 | A | G | 0.22167 | -0.0137 | 0.002 | 1.08E-10 | 665054 | A | G | 0.2227 | -7.00E-04 | 0.0047 | 0.8771 | 7.05494E-05 | 46.92235889 |
| DrnkWk | rs28694391 | 4 | 174154311 | C | T | 0.813121 | -0.0132 | 0.002 | 3.81E-09 | 666978 | C | T | 0.8155 | -0.0098 | 0.0051 | 0.0524204 | 6.53052E-05 | 43.55986938 |
| DrnkWk | rs28732378 | 3 | 85403892 | G | A | 0.718688 | -0.0197 | 0.002 | 1.55E-23 | 666978 | G | A | 0.7325 | 0.0082 | 0.0045 | 0.0662995 | 0.000145445 | 97.02220907 |
| DrnkWk | rs28929474 | 14 | 94844947 | T | C | 0.0168986 | -0.0489 | 0.006 | 3.28E-14 | 666978 | T | C | 0.0183 | -3.00E-04 | 0.0146 | 0.9848 | 9.95773E-05 | 66.42230083 |
| DrnkWk | rs322773 | 7 | 127762119 | G | A | 0.552684 | 0.00986 | 0.002 | 1.48E-08 | 666978 | G | A | 0.5553 | 0.0134 | 0.004 | 0.000786303 | 3.6439E-05 | 24.30482712 |
| DrnkWk | rs34121753 | 17 | 7733833 | G | A | 0.591451 | 0.0106 | 0.002 | 1.53E-09 | 666978 | G | A | 0.5666 | 0.003 | 0.004 | 0.4506 | 4.21136E-05 | 28.08991577 |
| DrnkWk | rs34484751 | 12 | 123652527 | C | A | 0.0228628 | 0.0312 | 0.005 | 7.86E-10 | 666978 | C | A | 0.0276 | -0.0195 | 0.0128 | 0.1267 | 5.83757E-05 | 38.93748324 |
| DrnkWk | rs34704785 | 13 | 68117681 | T | C | 0.520875 | -0.0102 | 0.002 | 4.52E-09 | 666978 | T | C | 0.5213 | 0.0081 | 0.0044 | 0.0660906 | 3.89953E-05 | 26.00992201 |
| DrnkWk | rs35011311 | 12 | 38616581 | T | G | 0.266402 | -0.0114 | 0.002 | 8.95E-09 | 648491 | T | G | 0.2694 | -0.0051 | 0.0047 | 0.2717 | 5.00984E-05 | 32.4898998 |
| DrnkWk | rs35807116 | 15 | 74667953 | T | C | 0.567594 | 0.0105 | 0.002 | 2.65E-09 | 666977 | T | C | 0.5919 | 0.0072 | 0.004 | 0.0726892 | 4.13228E-05 | 27.56241735 |
| DrnkWk | rs36123652 | 9 | 109363570 | G | A | 0.195825 | -0.0168 | 0.003 | 5.31E-10 | 463556 | G | A | 0.1755 | 0.0021 | 0.0052 | 0.679599 | 6.76464E-05 | 31.3598647 |
| DrnkWk | rs3768650 | 2 | 152990932 | G | A | 0.316103 | -0.0106 | 0.002 | 1.67E-08 | 666978 | G | A | 0.3049 | 0.0012 | 0.0043 | 0.7751 | 4.21136E-05 | 28.08991577 |
| DrnkWk | rs3809162 | 12 | 54674235 | G | A | 0.384692 | 0.0102 | 0.002 | 6.84E-09 | 666978 | G | A | 0.3942 | -0.0076 | 0.004 | 0.0591494 | 3.89953E-05 | 26.00992201 |
| DrnkWk | rs4337071 | 11 | 113334100 | T | C | 0.362823 | -0.0151 | 0.002 | 5.59E-17 | 666978 | T | C | 0.3755 | -0.0016 | 0.0041 | 0.6924 | 8.54565E-05 | 57.00232907 |
| DrnkWk | rs4481304 | 5 | 50759415 | A | G | 0.422465 | -0.0106 | 0.002 | 1.25E-09 | 666978 | A | G | 0.4373 | -0.0131 | 0.004 | 0.001034 | 4.21136E-05 | 28.08991577 |
| DrnkWk | rs4761961 | 12 | 51799968 | C | A | 0.558648 | -0.0108 | 0.002 | 5.19E-10 | 666978 | C | A | 0.5498 | -1.00E-04 | 0.004 | 0.9872 | 4.37177E-05 | 29.15991256 |
| DrnkWk | rs4890444 | 18 | 40750455 | G | C | 0.374751 | 0.0112 | 0.002 | 1.89E-09 | 611077 | G | C | 0.3762 | 4.00E-04 | 0.0041 | 0.9253 | 5.13166E-05 | 31.35989736 |
| DrnkWk | rs4916723 | 5 | 87854395 | C | A | 0.413519 | -0.0111 | 0.002 | 2.74E-10 | 666978 | C | A | 0.416 | -0.001 | 0.004 | 0.8126 | 4.61801E-05 | 30.80240764 |
| DrnkWk | rs530916 | 11 | 121549091 | G | A | 0.56163 | 0.011 | 0.002 | 4.22E-10 | 650123 | G | A | 0.5525 | 0.0011 | 0.004 | 0.787601 | 4.65275E-05 | 30.24990694 |
| DrnkWk | rs55872084 | 5 | 155902003 | T | G | 0.22664 | 0.012 | 0.002 | 4.68E-09 | 666978 | T | G | 0.2306 | -0.0068 | 0.0047 | 0.1448 | 5.39719E-05 | 35.99989205 |
| DrnkWk | rs55932213 | 9 | 108755622 | G | A | 0.701789 | 0.0127 | 0.002 | 1.61E-10 | 666978 | G | A | 0.7295 | -0.0032 | 0.0046 | 0.4848 | 6.04519E-05 | 40.32237909 |
| DrnkWk | rs55987845 | 20 | 18656930 | T | C | 0.414513 | -0.00979 | 0.002 | 3.41E-08 | 666978 | T | C | 0.3982 | 0.0118 | 0.004 | 0.00335598 | 3.59235E-05 | 23.96095315 |
| DrnkWk | rs56115085 | 12 | 6853615 | T | C | 0.145129 | -0.0132 | 0.002 | 4.14E-08 | 666978 | T | C | 0.1472 | -0.0021 | 0.0057 | 0.715501 | 6.53052E-05 | 43.55986938 |
| DrnkWk | rs56353702 | 5 | 132248575 | G | A | 0.145129 | -0.0141 | 0.003 | 2.16E-08 | 574259 | G | A | 0.16 | -0.0196 | 0.0055 | 0.000327899 | 3.84655E-05 | 22.08992307 |
| DrnkWk | rs60026303 | 3 | 81285635 | G | A | 0.178926 | 0.0121 | 0.002 | 3.58E-08 | 666978 | G | A | 0.1934 | -0.0043 | 0.005 | 0.3885 | 5.48751E-05 | 36.60239024 |
| DrnkWk | rs61934664 | 12 | 81608058 | A | G | 0.463221 | -0.0118 | 0.002 | 8.96E-12 | 666978 | A | G | 0.492 | -3.00E-04 | 0.0039 | 0.9348 | 5.21879E-05 | 34.80989562 |
| DrnkWk | rs62305763 | 4 | 100289405 | T | C | 0.0924453 | 0.0303 | 0.003 | 1.15E-28 | 666978 | T | C | 0.1033 | 0.0072 | 0.0066 | 0.2762 | 0.00015292 | 102.0096941 |
| DrnkWk | rs6531148 | 2 | 16650233 | C | T | 0.757455 | -0.0117 | 0.002 | 7.58E-09 | 666978 | C | T | 0.763 | 0.002 | 0.0046 | 0.6607 | 5.13072E-05 | 34.22239738 |
| DrnkWk | rs6584893 | 10 | 110504315 | C | A | 0.22664 | -0.011 | 0.002 | 3.17E-08 | 666978 | C | A | 0.2531 | -0.0012 | 0.0045 | 0.793999 | 4.53518E-05 | 30.24990929 |
| DrnkWk | rs6698883 | 1 | 34368735 | T | C | 0.899602 | -0.0161 | 0.003 | 1.14E-08 | 665054 | T | C | 0.905 | 0.0027 | 0.007 | 0.7007 | 4.33045E-05 | 28.8010245 |
| DrnkWk | rs6739804 | 2 | 63269604 | C | T | 0.642147 | -0.0136 | 0.002 | 2.85E-13 | 666978 | C | T | 0.6615 | -0.0214 | 0.0042 | 3.44E-07 | 6.93228E-05 | 46.23986134 |
| DrnkWk | rs6787172 | 3 | 158187811 | G | T | 0.537773 | -0.0107 | 0.002 | 7.49E-10 | 666978 | G | T | 0.5517 | -0.0113 | 0.004 | 0.00426305 | 4.29119E-05 | 28.62241417 |
| DrnkWk | rs68084872 | 2 | 58082205 | A | G | 0.266402 | -0.0113 | 0.002 | 6.40E-09 | 666978 | A | G | 0.2783 | -0.0023 | 0.0044 | 0.5947 | 4.78591E-05 | 31.92240428 |
| DrnkWk | rs6887908 | 5 | 144383685 | C | A | 0.563618 | -0.00951 | 0.002 | 4.47E-08 | 666978 | C | A | 0.5365 | -0.0063 | 0.0039 | 0.1093 | 3.38981E-05 | 22.6099572 |
| DrnkWk | rs6899302 | 5 | 166846529 | C | T | 0.514911 | -0.0104 | 0.002 | 1.70E-09 | 666978 | C | T | 0.4985 | -0.002 | 0.0041 | 0.6194 | 4.05394E-05 | 27.03991892 |
| DrnkWk | rs6962879 | 7 | 14295168 | G | C | 0.632207 | 0.00993 | 0.002 | 1.89E-08 | 666978 | G | C | 0.5991 | 0.0047 | 0.0042 | 0.2644 | 3.69582E-05 | 24.65115108 |
| DrnkWk | rs7162115 | 15 | 52291273 | T | G | 0.429423 | 0.00973 | 0.002 | 2.65E-08 | 666977 | T | G | 0.432 | 0.001 | 0.0041 | 0.7982 | 3.54846E-05 | 23.66815403 |
| DrnkWk | rs72770409 | 16 | 24802331 | T | C | 0.054672 | -0.0235 | 0.004 | 3.16E-09 | 665054 | T | C | 0.0554 | -4.00E-04 | 0.0088 | 0.9643 | 5.18963E-05 | 34.5155212 |
| DrnkWk | rs7284839 | 22 | 42015765 | T | C | 0.798211 | 0.0155 | 0.003 | 3.66E-08 | 421810 | T | C | 0.7994 | -3.00E-04 | 0.0049 | 0.9529 | 6.32815E-05 | 26.69431787 |
| DrnkWk | rs75199129 | 2 | 44298775 | T | A | 0.0387674 | -0.0283 | 0.004 | 8.23E-12 | 666978 | T | A | 0.0476 | -0.0049 | 0.0099 | 0.6175 | 7.50428E-05 | 50.0554749 |
| DrnkWk | rs7588444 | 2 | 178185364 | C | T | 0.82008 | 0.013 | 0.002 | 1.76E-09 | 666978 | C | T | 0.8006 | -0.0013 | 0.0049 | 0.784401 | 6.33414E-05 | 42.24987331 |
| DrnkWk | rs79616692 | 16 | 72338507 | C | G | 0.105368 | 0.0196 | 0.003 | 3.54E-12 | 665054 | C | G | 0.1094 | -0.0119 | 0.0064 | 0.0640796 | 6.41778E-05 | 42.68431608 |
| DrnkWk | rs800578 | 8 | 116869477 | C | T | 0.800199 | 0.0114 | 0.002 | 4.51E-08 | 666978 | C | T | 0.781 | -0.0116 | 0.0048 | 0.0155901 | 4.87099E-05 | 32.48990258 |
| DrnkWk | rs8020892 | 14 | 58724089 | A | G | 0.737575 | -0.0134 | 0.002 | 2.06E-12 | 666978 | A | G | 0.7233 | -0.0117 | 0.0044 | 0.00825106 | 6.7299E-05 | 44.88986539 |
| DrnkWk | rs823099 | 1 | 205669322 | A | C | 0.414513 | 0.0105 | 0.002 | 2.31E-09 | 665054 | A | C | 0.4247 | 0.0033 | 0.004 | 0.401 | 4.14423E-05 | 27.56241711 |
| DrnkWk | rs828867 | 2 | 74334462 | A | G | 0.522863 | 0.0103 | 0.002 | 3.44E-09 | 666978 | A | G | 0.5438 | 0.0069 | 0.004 | 0.0829908 | 3.97636E-05 | 26.52242047 |
| DrnkWk | rs838145 | 19 | 49248730 | A | G | 0.581511 | -0.0164 | 0.002 | 5.62E-21 | 665346 | A | G | 0.563 | -0.0097 | 0.004 | 0.0168799 | 0.00010105 | 67.23979788 |
| DrnkWk | rs9745238 | 15 | 49562930 | G | C | 0.667992 | -0.0132 | 0.002 | 1.38E-09 | 471489 | G | C | 0.6586 | 0.0089 | 0.0041 | 0.03111 | 9.23796E-05 | 43.55981522 |
| DrnkWk | rs9875033 | 3 | 184028428 | C | T | 0.766402 | 0.0122 | 0.002 | 7.90E-10 | 666978 | C | T | 0.7559 | 0.0128 | 0.0046 | 0.00569102 | 5.57858E-05 | 37.20988842 |
| LST:Leisure screen time;SmkInit:Smoking initiation;MVPA:Moderate-to-vigorous intensity physical activity during leisure time;SmkCes:Smoking cessation;SDW:Sedentary behaviour at work;SDC:Sedentary commuting behaviour;AgeSmk:Age of initiation;CigDay:Cigarettes per day;DrnkWk:Drinks per week. | | | | | | | | | | | | | | | | | | |
