# Supplementary material for: Causal associations between lifestyle factors and hemorrhoidal disease: Insights from Mendelian randomization analysis
Source: Medicine (Baltimore). 2026 May 22;105(21):e48945. doi: 10.1097/MD.0000000000048945 (PMC13200937; doi:10.1097/MD.0000000000048945)
Supplement: Supplementary file 3 [file medi-105-e48945-s003.docx]

| Supplementary Table 1.Main results of MR analyses | |  |  |  |  |  |  |  |  |  |
| --- | --- | --- | --- | --- | --- | --- | --- | --- | --- | --- |
| Expose | Method | | b | se | pval | lo_ci | up_ci | or | or_lci95 | or_uci95 |
| LST | IVW (RE) |  | 0.051 | 0.026 | 0.047 | 0.001 | 0.101 | 1.052 | 1.001 | 1.106 |
| LST | MR Egger |  | 0.035 | 0.116 | 0.763 | -0.193 | 0.263 | 1.036 | 0.825 | 1.301 |
| LST | WME |  | 0.074 | 0.028 | 0.008 | 0.019 | 0.128 | 1.077 | 1.020 | 1.137 |
| LST | MR PRESSO | Raw | 0.051 | 0.026 | 0.050 | 0.001 | 0.101 | 1.052 | 1.001 | 1.106 |
| LST |  | Outlier-corrected | 0.051 | 0.024 | 0.038 | 0.003 | 0.099 | 1.052 | 1.003 | 1.104 |
| LST | Radial IVW | Effect (1st) | 0.051 | 0.026 | 0.047 | 0.001 | 0.101 | 1.052 | 1.001 | 1.106 |
| LST |  | Iterative | 0.051 | 0.026 | 0.047 | 0.001 | 0.101 | 1.052 | 1.001 | 1.106 |
| LST |  | Exact (FE) | 0.054 | 0.017 | 0.001 | 0.021 | 0.087 | 1.055 | 1.021 | 1.091 |
| LST |  | Exact (RE) | 0.052 | 0.027 | 0.054 | 0.000 | 0.104 | 1.053 | 1.000 | 1.110 |
| LST | MR- lap | observed_effect | 0.031 | 0.017 | 0.074 | -0.003 | 0.064 | 1.031 | 0.997 | 1.066 |
| LST |  | corrected_effect | 0.037 | 0.022 | 0.091 | -0.006 | 0.081 | 1.038 | 0.994 | 1.084 |
| LST | IVW(RE)* |  | 0.053 | 0.019 | 0.006 | 0.015 | 0.091 | 1.055 | 1.015 | 1.096 |
| LST | MR Egger* |  | -0.041 | 0.088 | 0.641 | -0.215 | 0.132 | 0.959 | 0.807 | 1.141 |
| LST | WME* |  | 0.073 | 0.028 | 0.009 | 0.018 | 0.128 | 1.076 | 1.018 | 1.137 |
| LST | MR PRESSO* |  | 0.053 | 0.018 | 0.004 | 0.018 | 0.089 | 1.055 | 1.018 | 1.093 |
| LST | Radial IVW* | Effect (1st) | 0.053 | 0.018 | 0.003 | 0.018 | 0.089 | 1.055 | 1.018 | 1.093 |
| LST |  | Iterative | 0.053 | 0.018 | 0.003 | 0.018 | 0.089 | 1.055 | 1.018 | 1.093 |
| LST |  | Exact (FE) | 0.054 | 0.019 | 0.005 | 0.016 | 0.093 | 1.056 | 1.016 | 1.097 |
| LST |  | Exact (RE) | 0.054 | 0.019 | 0.005 | 0.018 | 0.091 | 1.056 | 1.018 | 1.095 |
| MVPA | IVW (RE) |  | 0.001 | 0.084 | 0.993 | -0.164 | 0.166 | 1.001 | 0.849 | 1.180 |
| MVPA | MR Egger |  | -0.025 | 0.405 | 0.952 | -0.819 | 0.769 | 0.975 | 0.441 | 2.157 |
| MVPA | WME |  | -0.155 | 0.068 | 0.022 | -0.288 | -0.023 | 0.856 | 0.750 | 0.978 |
| MVPA | MR PRESSO | Raw | 0.001 | 0.084 | 0.993 | -0.164 | 0.166 | 1.001 | 0.849 | 1.180 |
| MVPA |  | Outlier-corrected | -0.059 | 0.064 | 0.378 | -0.185 | 0.067 | 0.943 | 0.831 | 1.069 |
| MVPA | Radial IVW | Effect (1st) | 0.001 | 0.084 | 0.993 | -0.164 | 0.166 | 1.001 | 0.849 | 1.180 |
| MVPA |  | Iterative | 0.001 | 0.084 | 0.993 | -0.164 | 0.166 | 1.001 | 0.849 | 1.180 |
| MVPA |  | Exact (FE) | 0.001 | 0.045 | 0.985 | -0.087 | 0.088 | 1.001 | 0.917 | 1.092 |
| MVPA |  | Exact (RE) | 0.001 | 0.085 | 0.993 | -0.166 | 0.168 | 1.001 | 0.847 | 1.183 |
| MVPA | MR- lap | observed_effect | -0.004 | 0.058 | 0.948 | -0.118 | 0.110 | 0.996 | 0.889 | 1.117 |
| MVPA |  | corrected_effect | -0.003 | 0.085 | 0.970 | -0.170 | 0.163 | 0.997 | 0.844 | 1.178 |
| MVPA | IVW(RE)* |  | -0.105 | 0.051 | 0.039 | -0.205 | -0.005 | 0.900 | 0.814 | 0.995 |
| MVPA | MR Egger* |  | -0.395 | 0.292 | 0.214 | -0.967 | 0.178 | 0.674 | 0.380 | 1.195 |
| MVPA | WME* |  | -0.168 | 0.069 | 0.015 | -0.302 | -0.033 | 0.846 | 0.739 | 0.967 |
| MVPA | MR PRESSO* |  | -0.105 | 0.045 | 0.042 | -0.193 | -0.018 | 0.900 | 0.825 | 0.982 |
| MVPA | Radial IVW* | Effect (1st) | -0.105 | 0.045 | 0.018 | -0.193 | -0.018 | 0.900 | 0.825 | 0.982 |
| MVPA |  | Iterative | -0.105 | 0.045 | 0.018 | -0.192 | -0.018 | 0.900 | 0.825 | 0.982 |
| MVPA |  | Exact (FE) | -0.107 | 0.051 | 0.037 | -0.208 | -0.007 | 0.898 | 0.812 | 0.993 |
| MVPA |  | Exact (RE) | -0.107 | 0.044 | 0.037 | -0.193 | -0.021 | 0.898 | 0.824 | 0.979 |
| SDC | IVW (RE) |  | -0.067 | 0.043 | 0.118 | -0.152 | 0.017 | 0.935 | 0.859 | 1.017 |
| SDC | MR Egger |  | -0.041 | 0.128 | 0.757 | -0.293 | 0.211 | 0.960 | 0.746 | 1.235 |
| SDC | WME |  | -0.070 | 0.048 | 0.141 | -0.163 | 0.023 | 0.932 | 0.849 | 1.023 |
| SDC | MR PRESSO | Raw | -0.067 | 0.043 | 0.142 | -0.152 | 0.017 | 0.935 | 0.859 | 1.017 |
| SDC |  | Outlier-corrected | -0.029 | 0.041 | 0.494 | -0.110 | 0.051 | 0.971 | 0.896 | 1.053 |
| SDC | Radial IVW | Effect (1st) | -0.067 | 0.043 | 0.118 | -0.152 | 0.017 | 0.935 | 0.859 | 1.017 |
| SDC |  | Iterative | -0.067 | 0.043 | 0.120 | -0.152 | 0.017 | 0.935 | 0.859 | 1.018 |
| SDC |  | Exact (FE) | -0.072 | 0.032 | 0.025 | -0.136 | -0.009 | 0.930 | 0.873 | 0.991 |
| SDC |  | Exact (RE) | -0.070 | 0.044 | 0.137 | -0.157 | 0.017 | 0.932 | 0.855 | 1.017 |
| SDC | MR- lap | observed_effect | -0.012 | 0.026 | 0.631 | -0.063 | 0.038 | 0.988 | 0.939 | 1.039 |
| SDC |  | corrected_effect | -0.026 | 0.059 | 0.664 | -0.141 | 0.090 | 0.975 | 0.869 | 1.094 |
| SDC | IVW(RE)* |  | -0.065 | 0.037 | 0.078 | -0.137 | 0.007 | 0.937 | 0.872 | 1.007 |
| SDC | MR Egger* |  | -0.058 | 0.101 | 0.579 | -0.255 | 0.140 | 0.944 | 0.775 | 1.150 |
| SDC | WME* |  | -0.071 | 0.048 | 0.137 | -0.165 | 0.023 | 0.931 | 0.848 | 1.023 |
| SDC | MR PRESSO* |  | -0.065 | 0.037 | 0.106 | -0.137 | 0.007 | 0.937 | 0.872 | 1.007 |
| SDC | Radial IVW* | Effect (1st) | -0.065 | 0.037 | 0.078 | -0.137 | 0.007 | 0.937 | 0.872 | 1.007 |
| SDC |  | Iterative | -0.065 | 0.037 | 0.080 | -0.137 | 0.008 | 0.937 | 0.872 | 1.008 |
| SDC |  | Exact (FE) | -0.067 | 0.036 | 0.061 | -0.137 | 0.003 | 0.935 | 0.872 | 1.003 |
| SDC |  | Exact (RE) | -0.067 | 0.032 | 0.059 | -0.129 | -0.004 | 0.936 | 0.879 | 0.996 |
| SDW | IVW (RE) |  | 0.033 | 0.071 | 0.639 | -0.106 | 0.173 | 1.034 | 0.899 | 1.189 |
| SDW | MR Egger |  | -0.030 | 0.248 | 0.908 | -0.516 | 0.456 | 0.971 | 0.597 | 1.578 |
| SDW | WME |  | 0.000 | 0.072 | 0.997 | -0.140 | 0.141 | 1.000 | 0.869 | 1.151 |
| SDW | MR PRESSO | Raw | 0.033 | 0.071 | 0.651 | -0.106 | 0.173 | 1.034 | 0.899 | 1.189 |
| SDW |  | Outlier-corrected | 0.085 | 0.060 | 0.204 | -0.032 | 0.202 | 1.089 | 0.969 | 1.224 |
| SDW | Radial IVW | Effect (1st) | 0.033 | 0.071 | 0.639 | -0.106 | 0.173 | 1.034 | 0.899 | 1.189 |
| SDW |  | Iterative | 0.033 | 0.071 | 0.639 | -0.106 | 0.173 | 1.034 | 0.899 | 1.188 |
| SDW |  | Exact (FE) | 0.036 | 0.041 | 0.381 | -0.045 | 0.117 | 1.037 | 0.956 | 1.124 |
| SDW |  | Exact (RE) | 0.034 | 0.085 | 0.699 | -0.133 | 0.202 | 1.035 | 0.875 | 1.223 |
| SDW | MR lap | observed_effect | 0.024 | 0.052 | 0.644 | -0.078 | 0.126 | 1.024 | 0.925 | 1.134 |
| SDW |  | corrected_effect | 0.035 | 0.075 | 0.646 | -0.113 | 0.182 | 1.035 | 0.893 | 1.199 |
| SDW | IVW(RE)* |  | 0.085 | 0.060 | 0.154 | -0.032 | 0.202 | 1.089 | 0.969 | 1.224 |
| SDW | MR Egger* |  | 0.503 | 0.206 | 0.058 | 0.100 | 0.907 | 1.654 | 1.105 | 2.477 |
| SDW | WME* |  | 0.174 | 0.072 | 0.016 | 0.032 | 0.316 | 1.190 | 1.033 | 1.372 |
| SDW | MR PRESSO* |  | 0.085 | 0.060 | 0.204 | -0.032 | 0.202 | 1.089 | 0.969 | 1.224 |
| SDW | Radial IVW* | Effect (1st) | 0.085 | 0.060 | 0.154 | -0.032 | 0.202 | 1.089 | 0.969 | 1.224 |
| SDW |  | Iterative | 0.085 | 0.060 | 0.156 | -0.032 | 0.202 | 1.089 | 0.968 | 1.224 |
| SDW |  | Exact (FE) | 0.087 | 0.054 | 0.107 | -0.019 | 0.194 | 1.091 | 0.981 | 1.214 |
| SDW |  | Exact (RE) | 0.087 | 0.055 | 0.164 | -0.020 | 0.194 | 1.091 | 0.980 | 1.215 |
| SmkInit | IVW (RE) |  | 0.081 | 0.040 | 0.045 | 0.002 | 0.160 | 1.084 | 1.002 | 1.174 |
| SmkInit | MR Egger |  | 0.029 | 0.176 | 0.869 | -0.316 | 0.374 | 1.030 | 0.729 | 1.453 |
| SmkInit | WME |  | 0.035 | 0.043 | 0.420 | -0.049 | 0.118 | 1.035 | 0.952 | 1.126 |
| SmkInit | MR PRESSO | Raw | 0.081 | 0.040 | 0.046 | 0.002 | 0.160 | 1.084 | 1.002 | 1.174 |
| SmkInit |  | Outlier-corrected | 0.057 | 0.037 | 0.126 | -0.016 | 0.130 | 1.059 | 0.984 | 1.139 |
| SmkInit | Radial IVW | Effect (1st) | 0.081 | 0.040 | 0.045 | 0.002 | 0.160 | 1.084 | 1.002 | 1.174 |
| SmkInit |  | Iterative | 0.081 | 0.040 | 0.045 | 0.002 | 0.160 | 1.084 | 1.002 | 1.174 |
| SmkInit |  | Exact (FE) | 0.088 | 0.025 | 0.000 | 0.039 | 0.136 | 1.092 | 1.040 | 1.146 |
| SmkInit |  | Exact (RE) | 0.083 | 0.039 | 0.032 | 0.008 | 0.159 | 1.087 | 1.008 | 1.173 |
| SmkInit | MR lap | observed_effect | 0.047 | 0.015 | 0.002 | 0.017 | 0.077 | 1.048 | 1.017 | 1.080 |
| SmkInit |  | corrected_effect | 0.056 | 0.018 | 0.002 | 0.020 | 0.092 | 1.058 | 1.020 | 1.096 |
| SmkInit | IVW(RE)* |  | 0.064 | 0.029 | 0.029 | 0.007 | 0.121 | 1.066 | 1.007 | 1.129 |
| SmkInit | MR Egger* |  | -0.033 | 0.125 | 0.792 | -0.277 | 0.211 | 0.968 | 0.758 | 1.235 |
| SmkInit | WME* |  | 0.035 | 0.042 | 0.413 | -0.048 | 0.117 | 1.035 | 0.953 | 1.124 |
| SmkInit | MR PRESSO* |  | 0.064 | 0.029 | 0.030 | 0.007 | 0.121 | 1.066 | 1.007 | 1.129 |
| SmkInit | Radial IVW* | Effect (1st) | 0.064 | 0.029 | 0.029 | 0.007 | 0.121 | 1.066 | 1.007 | 1.129 |
| SmkInit |  | Iterative | 0.064 | 0.029 | 0.029 | 0.007 | 0.121 | 1.066 | 1.007 | 1.129 |
| SmkInit |  | Exact (FE) | 0.066 | 0.028 | 0.019 | 0.011 | 0.121 | 1.068 | 1.011 | 1.129 |
| SmkInit |  | Exact (RE) | 0.066 | 0.030 | 0.032 | 0.006 | 0.125 | 1.068 | 1.006 | 1.133 |
| SmkCes | IVW (RE) |  | -0.145 | 0.063 | 0.021 | -0.268 | -0.022 | 0.865 | 0.765 | 0.978 |
| SmkCes | MR Egger |  | -0.005 | 0.165 | 0.978 | -0.327 | 0.318 | 0.995 | 0.721 | 1.374 |
| SmkCes | WME |  | -0.144 | 0.078 | 0.066 | -0.298 | 0.009 | 0.866 | 0.743 | 1.009 |
| SmkCes | MR PRESSO | Raw | -0.145 | 0.063 | 0.031 | -0.268 | -0.022 | 0.865 | 0.765 | 0.978 |
| SmkCes | Radial IVW | Effect (1st) | -0.145 | 0.063 | 0.021 | -0.268 | -0.022 | 0.865 | 0.765 | 0.978 |
| SmkCes |  | Iterative | -0.145 | 0.063 | 0.021 | -0.268 | -0.022 | 0.865 | 0.765 | 0.978 |
| SmkCes |  | Exact (FE) | -0.148 | 0.050 | 0.003 | -0.246 | -0.051 | 0.862 | 0.782 | 0.950 |
| SmkCes |  | Exact (RE) | -0.147 | 0.059 | 0.022 | -0.263 | -0.031 | 0.863 | 0.769 | 0.970 |
| SmkCes | MR lap | observed_effect | -0.039 | 0.019 | 0.035 | -0.075 | -0.003 | 0.962 | 0.927 | 0.997 |
| SmkCes |  | corrected_effect | -0.050 | 0.024 | 0.035 | -0.097 | -0.003 | 0.951 | 0.907 | 0.997 |
| SmkCes | IVW(RE)* |  | -0.104 | 0.053 | 0.050 | -0.208 | 0.000 | 0.901 | 0.812 | 1.000 |
| SmkCes | MR Egger* |  | -0.049 | 0.141 | 0.732 | -0.326 | 0.228 | 0.952 | 0.722 | 1.256 |
| SmkCes | WME* |  | -0.143 | 0.077 | 0.063 | -0.294 | 0.008 | 0.867 | 0.746 | 1.008 |
| SmkCes | MR PRESSO* |  | -0.104 | 0.053 | 0.065 | -0.208 | 0.000 | 0.901 | 0.812 | 1.000 |
| SmkCes | Radial IVW* | Effect (1st) | -0.104 | 0.053 | 0.050 | -0.208 | 0.000 | 0.901 | 0.812 | 1.000 |
| SmkCes |  | Iterative | -0.104 | 0.053 | 0.050 | -0.208 | 0.000 | 0.901 | 0.812 | 1.000 |
| SmkCes |  | Exact (FE) | -0.106 | 0.051 | 0.038 | -0.206 | -0.006 | 0.900 | 0.814 | 0.994 |
| SmkCes |  | Exact (RE) | -0.106 | 0.052 | 0.055 | -0.207 | -0.004 | 0.900 | 0.813 | 0.996 |
| CigDay | IVW (RE) |  | -0.045 | 0.039 | 0.245 | -0.120 | 0.031 | 0.956 | 0.887 | 1.031 |
| CigDay | MR Egger |  | -0.044 | 0.071 | 0.537 | -0.184 | 0.095 | 0.957 | 0.832 | 1.100 |
| CigDay | WME |  | -0.091 | 0.043 | 0.034 | -0.175 | -0.007 | 0.913 | 0.840 | 0.993 |
| CigDay | MR PRESSO | Raw | -0.045 | 0.039 | 0.251 | -0.120 | 0.031 | 0.956 | 0.887 | 1.031 |
| CigDay |  | Outlier-corrected | -0.055 | 0.036 | 0.133 | -0.126 | 0.015 | 0.946 | 0.882 | 1.016 |
| CigDay | Radial IVW | Effect (1st) | -0.045 | 0.039 | 0.245 | -0.120 | 0.031 | 0.956 | 0.887 | 1.031 |
| CigDay |  | Iterative | -0.045 | 0.039 | 0.245 | -0.120 | 0.031 | 0.956 | 0.887 | 1.031 |
| CigDay |  | Exact (FE) | -0.046 | 0.026 | 0.081 | -0.098 | 0.006 | 0.955 | 0.907 | 1.006 |
| CigDay |  | Exact (RE) | -0.045 | 0.041 | 0.276 | -0.126 | 0.035 | 0.956 | 0.882 | 1.036 |
| CigDay | MR lap | observed_effect | -0.007 | 0.011 | 0.517 | -0.028 | 0.014 | 0.993 | 0.972 | 1.014 |
| CigDay |  | corrected_effect | -0.009 | 0.013 | 0.465 | -0.034 | 0.016 | 0.991 | 0.966 | 1.016 |
| CigDay | IVW(RE)* |  | -0.037 | 0.030 | 0.219 | -0.095 | 0.022 | 0.964 | 0.909 | 1.022 |
| CigDay | MR Egger* |  | -0.053 | 0.055 | 0.335 | -0.160 | 0.054 | 0.948 | 0.852 | 1.055 |
| CigDay | WME* |  | -0.090 | 0.041 | 0.028 | -0.170 | -0.010 | 0.914 | 0.844 | 0.990 |
| CigDay | MR PRESSO* |  | -0.037 | 0.030 | 0.226 | -0.095 | 0.022 | 0.964 | 0.909 | 1.022 |
| CigDay | Radial IVW* | Effect (1st) | -0.037 | 0.030 | 0.219 | -0.095 | 0.022 | 0.964 | 0.909 | 1.022 |
| CigDay |  | Iterative | -0.037 | 0.030 | 0.219 | -0.095 | 0.022 | 0.964 | 0.909 | 1.022 |
| CigDay |  | Exact (FE) | -0.037 | 0.027 | 0.170 | -0.090 | 0.016 | 0.964 | 0.914 | 1.016 |
| CigDay |  | Exact (RE) | -0.037 | 0.038 | 0.340 | -0.112 | 0.038 | 0.964 | 0.894 | 1.039 |
| AgeSmk | IVW (RE) |  | -0.144 | 0.131 | 0.270 | -0.400 | 0.112 | 0.866 | 0.670 | 1.118 |
| AgeSmk | MR Egger |  | -0.342 | 0.561 | 0.565 | -1.442 | 0.758 | 0.711 | 0.236 | 2.135 |
| AgeSmk | WME |  | -0.003 | 0.125 | 0.980 | -0.249 | 0.243 | 0.997 | 0.780 | 1.275 |
| AgeSmk | MR PRESSO | Raw | -0.144 | 0.131 | 0.306 | -0.400 | 0.112 | 0.866 | 0.670 | 1.118 |
| AgeSmk |  | Outlier-corrected | -0.003 | 0.079 | 0.974 | -0.158 | 0.153 | 0.997 | 0.854 | 1.165 |
| AgeSmk | Radial IVW | Effect (1st) | -0.144 | 0.131 | 0.270 | -0.400 | 0.112 | 0.866 | 0.670 | 1.118 |
| AgeSmk |  | Iterative | -0.144 | 0.131 | 0.270 | -0.400 | 0.112 | 0.866 | 0.670 | 1.119 |
| AgeSmk |  | Exact (FE) | -0.151 | 0.089 | 0.091 | -0.327 | 0.024 | 0.860 | 0.721 | 1.025 |
| AgeSmk |  | Exact (RE) | -0.147 | 0.137 | 0.318 | -0.416 | 0.121 | 0.863 | 0.660 | 1.129 |
| AgeSmk | MR lap | observed_effect | -0.043 | 0.024 | 0.076 | -0.090 | 0.005 | 0.958 | 0.914 | 1.005 |
| AgeSmk |  | corrected_effect | -0.056 | 0.032 | 0.075 | -0.118 | 0.006 | 0.945 | 0.888 | 1.006 |
| AgeSmk | IVW(RE)* |  | -0.003 | 0.099 | 0.978 | -0.196 | 0.191 | 0.997 | 0.822 | 1.210 |
| AgeSmk | MR Egger* |  | 0.190 | 0.392 | 0.648 | -0.578 | 0.959 | 1.209 | 0.561 | 2.608 |
| AgeSmk | WME* |  | 0.028 | 0.128 | 0.826 | -0.222 | 0.279 | 1.029 | 0.801 | 1.321 |
| AgeSmk | MR PRESSO* |  | -0.003 | 0.079 | 0.974 | -0.158 | 0.153 | 0.997 | 0.854 | 1.165 |
| AgeSmk | Radial IVW* | Effect (1st) | -0.003 | 0.079 | 0.973 | -0.158 | 0.153 | 0.997 | 0.854 | 1.165 |
| AgeSmk |  | Iterative | -0.003 | 0.079 | 0.973 | -0.158 | 0.153 | 0.997 | 0.854 | 1.165 |
| AgeSmk |  | Exact (FE) | -0.003 | 0.099 | 0.978 | -0.196 | 0.190 | 0.997 | 0.822 | 1.210 |
| AgeSmk |  | Exact (RE) | -0.003 | 0.074 | 0.972 | -0.148 | 0.143 | 0.997 | 0.862 | 1.153 |
| DrnkWk | IVW (RE) |  | 0.004 | 0.058 | 0.951 | -0.110 | 0.118 | 1.004 | 0.896 | 1.125 |
| DrnkWk | MR Egger |  | -0.399 | 0.182 | 0.031 | -0.756 | -0.042 | 0.671 | 0.470 | 0.959 |
| DrnkWk | WME |  | -0.017 | 0.062 | 0.778 | -0.138 | 0.104 | 0.983 | 0.871 | 1.109 |
| DrnkWk | MR PRESSO | Raw | 0.004 | 0.058 | 0.951 | -0.110 | 0.118 | 1.004 | 0.896 | 1.125 |
| DrnkWk |  | Outlier-corrected | -0.018 | 0.053 | 0.741 | -0.122 | 0.087 | 0.982 | 0.885 | 1.090 |
| DrnkWk | Radial IVW | Effect (1st) | 0.004 | 0.058 | 0.951 | -0.110 | 0.118 | 1.004 | 0.896 | 1.125 |
| DrnkWk |  | Iterative | 0.004 | 0.058 | 0.951 | -0.110 | 0.118 | 1.004 | 0.896 | 1.125 |
| DrnkWk |  | Exact (FE) | 0.004 | 0.036 | 0.915 | -0.067 | 0.075 | 1.004 | 0.935 | 1.078 |
| DrnkWk |  | Exact (RE) | 0.004 | 0.063 | 0.953 | -0.119 | 0.126 | 1.004 | 0.888 | 1.135 |
| DrnkWk | MR lap | observed_effect | -0.012 | 0.017 | 0.482 | -0.044 | 0.021 | 0.988 | 0.957 | 1.021 |
| DrnkWk |  | corrected_effect | -0.016 | 0.020 | 0.437 | -0.056 | 0.024 | 0.984 | 0.946 | 1.024 |
| DrnkWk | IVW(RE)* |  | -0.059 | 0.043 | 0.168 | -0.142 | 0.025 | 0.943 | 0.868 | 1.025 |
| DrnkWk | MR Egger* |  | -0.215 | 0.138 | 0.123 | -0.485 | 0.055 | 0.806 | 0.616 | 1.056 |
| DrnkWk | WME* |  | -0.016 | 0.062 | 0.791 | -0.138 | 0.105 | 0.984 | 0.871 | 1.111 |
| DrnkWk | MR PRESSO* |  | -0.059 | 0.039 | 0.141 | -0.136 | 0.019 | 0.943 | 0.873 | 1.019 |
| DrnkWk | Radial IVW* | Effect (1st) | -0.059 | 0.039 | 0.137 | -0.136 | 0.019 | 0.943 | 0.873 | 1.019 |
| DrnkWk |  | Iterative | -0.059 | 0.039 | 0.137 | -0.136 | 0.019 | 0.943 | 0.873 | 1.019 |
| DrnkWk |  | Exact (FE) | -0.060 | 0.043 | 0.159 | -0.143 | 0.023 | 0.942 | 0.866 | 1.024 |
| DrnkWk |  | Exact (RE) | -0.060 | 0.042 | 0.159 | -0.143 | 0.023 | 0.942 | 0.867 | 1.023 |
| “*”Analysis after removal of outliers. |  |  |  |  |  |  |  |  |  |  |
| LST:Leisure screen time;SmkInit:Smoking initiation;MVPA:Moderate-to-vigorous intensity physical activity during leisure time;SmkCes:Smoking cessation;SDW:Sedentary behaviour at work;SDC:Sedentary commuting behaviour;AgeSmk:Age of initiation;CigDay:Cigarettes per day;DrnkWk:Drinks per week. | | | | | | | | | | |
