# Supplementary material for: Causal associations between lifestyle factors and hemorrhoidal disease: Insights from Mendelian randomization analysis
Source: Medicine (Baltimore). 2026 May 22;105(21):e48945. doi: 10.1097/MD.0000000000048945 (PMC13200937; doi:10.1097/MD.0000000000048945)
Supplement: Supplementary file 4 [file medi-105-e48945-s004.docx]

| Supplementary Table 2.Results of MRlap | | | | | | | | | | | | | |
| --- | --- | --- | --- | --- | --- | --- | --- | --- | --- | --- | --- | --- | --- |
| Exposure | Set Distance | Sst p | Nsnp | Observed Effect | Observed Effect se | Observed Effect p | Corrected Effect | Corrected Effect se | Corrected Effect p | Test Difference | p_difference | LDSC_rg | LDSC_h2_exp |
| LST | 5000 Kb | 5.00E-08 | 102 | 0.031 | 0.017 | 0.074 | 0.037 | 0.022 | 0.091 | -1.435 | 0.151 | 0.071 | 0.076 |
| MVPA | 5000 Kb | 5.00E-08 | 16 | -0.004 | 0.058 | 0.948 | -0.003 | 0.085 | 0.970 | -0.026 | 0.980 | -0.087 | 0.029 |
| SDW | 5000 Kb | 5.00E-08 | 9 | 0.024 | 0.052 | 0.644 | 0.035 | 0.075 | 0.646 | -0.446 | 0.656 | -0.058 | 0.028 |
| SDC | 5000 Kb | 5.00E-06 | 18 | -0.012 | 0.026 | 0.631 | -0.026 | 0.059 | 0.664 | 0.399 | 0.690 | 0.119 | 0.026 |
| SmkInit | 5000 Kb | 5.00E-08 | 194 | 0.047 | 0.015 | 0.002 | 0.056 | 0.018 | 0.002 | -2.162 | 0.031 | 0.084 | 0.063 |
| SmkCes | 5000 Kb | 5.00E-08 | 59 | -0.039 | 0.019 | 0.035 | -0.050 | 0.024 | 0.035 | 2.123 | 0.034 | -0.038 | 0.069 |
| CigDay | 5000 Kb | 5.00E-08 | 91 | -0.007 | 0.011 | 0.517 | -0.009 | 0.013 | 0.465 | 1.097 | 0.273 | 0.040 | 0.077 |
| AgeSmk | 5000 Kb | 5.00E-08 | 37 | -0.043 | 0.024 | 0.076 | -0.056 | 0.032 | 0.075 | 1.708 | 0.088 | -0.164 | 0.047 |
| DrnkWk | 5000 Kb | 5.00E-08 | 89 | -0.012 | 0.017 | 0.482 | -0.016 | 0.020 | 0.437 | 1.150 | 0.250 | -0.009 | 0.047 |
| Nsnp, number of single nucleotide polymorphisms (SNPs). LDSC_rg,genetic correlation estimate; LDSC_h2_exp,exposure heritability estimate. | | | | | | | | | | | | | |
| LST:Leisure screen time;SmkInit:Smoking initiation;MVPA:Moderate-to-vigorous intensity physical activity during leisure time;SmkCes:Smoking cessation;SDW:Sedentary behaviour at work;SDC:Sedentary commuting behaviour;AgeSmk:Age of initiation;CigDay:Cigarettes per day;DrnkWk:Drinks per week. | | | | | | | | | | | | | |
