# Supplementary material for: Causal associations between lifestyle factors and hemorrhoidal disease: Insights from Mendelian randomization analysis
Source: Medicine (Baltimore). 2026 May 22;105(21):e48945. doi: 10.1097/MD.0000000000048945 (PMC13200937; doi:10.1097/MD.0000000000048945)
Supplement: Supplementary file 5 [file medi-105-e48945-s005.docx]

| Supplementary Table 3 . Sensitivity analyses | | | | | | | | | | | |
| --- | --- | --- | --- | --- | --- | --- | --- | --- | --- | --- | --- |
| Exposure | Nsnp | Cochran Q test MR-Egger | | | Egger-intercept test | | | MR PRESSO Global Test | | Directionality Test | |
|  |  | Q | Q_df | Q_pval | egger_intercept | se | pval | RSSobs | Pvalue | Correct Causal Direction | Seiger Pval |
| LST | 106 | 243.878 | 104 | 2.99E-13 | 4.25E-04 | 3.10E-03 | 0.891 | 248.988 | <0.001 | TRUE | 0 |
| MVPA | 14 | 46.021 | 12 | 6.88E-06 | 6.76E-04 | 1.03E-02 | 0.949 | 53.815 | <0.001 | TRUE | 6.04E-49 |
| SDC | 14 | 23.571 | 12 | 2.32E-02 | -1.63E-03 | 7.38E-03 | 0.829 | 27.496 | 0.047 | TRUE | 9.19E-51 |
| SDW | 9 | 23.674 | 7 | 1.30E-03 | 2.31E-03 | 8.65E-03 | 0.798 | 36.674 | 0.007 | TRUE | 3.40E-45 |
| SmkInit | 224 | 596.799 | 222 | 4.29E-36 | 6.53E-04 | 2.15E-03 | 0.762 | 602.292 | <0.001 | TRUE | 0 |
| AgeSmk | 8 | 14.741 | 6 | 2.24E-02 | 3.87E-03 | 1.06E-02 | 0.729 | 21.313 | 0.038 | TRUE | 1.72E-38 |
| CigDay without UKB | 47 | 98.035 | 45 | 8.27E-06 | -1.46E-05 | 1.90E-03 | 0.994 | 103.749 | <0.001 | TRUE | 0 |
| SmkCes without UKB | 22 | 32.184 | 20 | 4.14E-02 | -3.10E-03 | 3.37E-03 | 0.368 | 36.679 | 0.045 | TRUE | 1.37E-154 |
| DrnkWk without UKB | 86 | 204.384 | 84 | 4.99E-12 | 5.73E-03 | 2.46E-03 | 0.022 | 223.468 | <0.001 | TRUE | 1.18E-147 |
| LST* | 81 | 67.745 | 79 | 0.813 | 2.55E-03 | 2.32E-03 | 0.276 | 70.762 | 0.821 | TRUE | 0 |
| MVPA* | 10 | 5.831 | 8 | 0.666 | 7.65E-03 | 7.61E-03 | 0.344 | 8.553 | 0.673 | TRUE | 3.08E-46 |
| SDC* | 12 | 11.893 | 10 | 0.292 | -4.64E-04 | 6.02E-03 | 0.940 | 13.546 | 0.438 | TRUE | 3.71E-46 |
| SDW* | 7 | 2.950 | 5 | 0.708 | -1.32E-02 | 6.29E-03 | 0.089 | 9.747 | 0.336 | TRUE | 1.76E-31 |
| SmkInit* | 172 | 182.786 | 170 | 0.238 | 1.22E-03 | 1.53E-03 | 0.426 | 185.697 | 0.216 | TRUE | 0 |
| AgeSmk* | 7 | 3.629 | 5 | 0.604 | -3.64E-03 | 7.16E-03 | 0.633 | 5.102 | 0.716 | TRUE | 2.58E-36 |
| CigDay* | 42 | 49.552 | 40 | 0.143 | 5.59E-04 | 1.53E-03 | 0.716 | 55.119 | 0.148 | TRUE | 0 |
| SmkCes* | 20 | 20.521 | 18 | 0.304 | -1.24E-03 | 2.95E-03 | 0.679 | 23.180 | 0.359 | TRUE | 7.07E-155 |
| DrnkWk* | 67 | 55.162 | 65 | 0.803 | 2.18E-03 | 1.82E-03 | 0.236 | 58.405 | 0.805 | TRUE | 9.11E-287 |
| Nsnp, number of single nucleotide polymorphisms (SNPs).“*”Analysis after removal of outliers. | | | | | | | | | | | |
| LST:Leisure screen time;SmkInit:Smoking initiation;MVPA:Moderate-to-vigorous intensity physical activity during leisure time;SmkCes:Smoking cessation;SDW:Sedentary behaviour at work;SDC:Sedentary commuting behaviour;AgeSmk:Age of initiation;CigDay:Cigarettes per day;DrnkWk:Drinks per week. | | | | | | | | | | | |
