# Supplementary figures and images for: Causal associations between lifestyle factors and hemorrhoidal disease: Insights from Mendelian randomization analysis
Source: Medicine (Baltimore). 2026 May 22;105(21):e48945. doi: 10.1097/MD.0000000000048945 (PMC13200937; doi:10.1097/MD.0000000000048945)

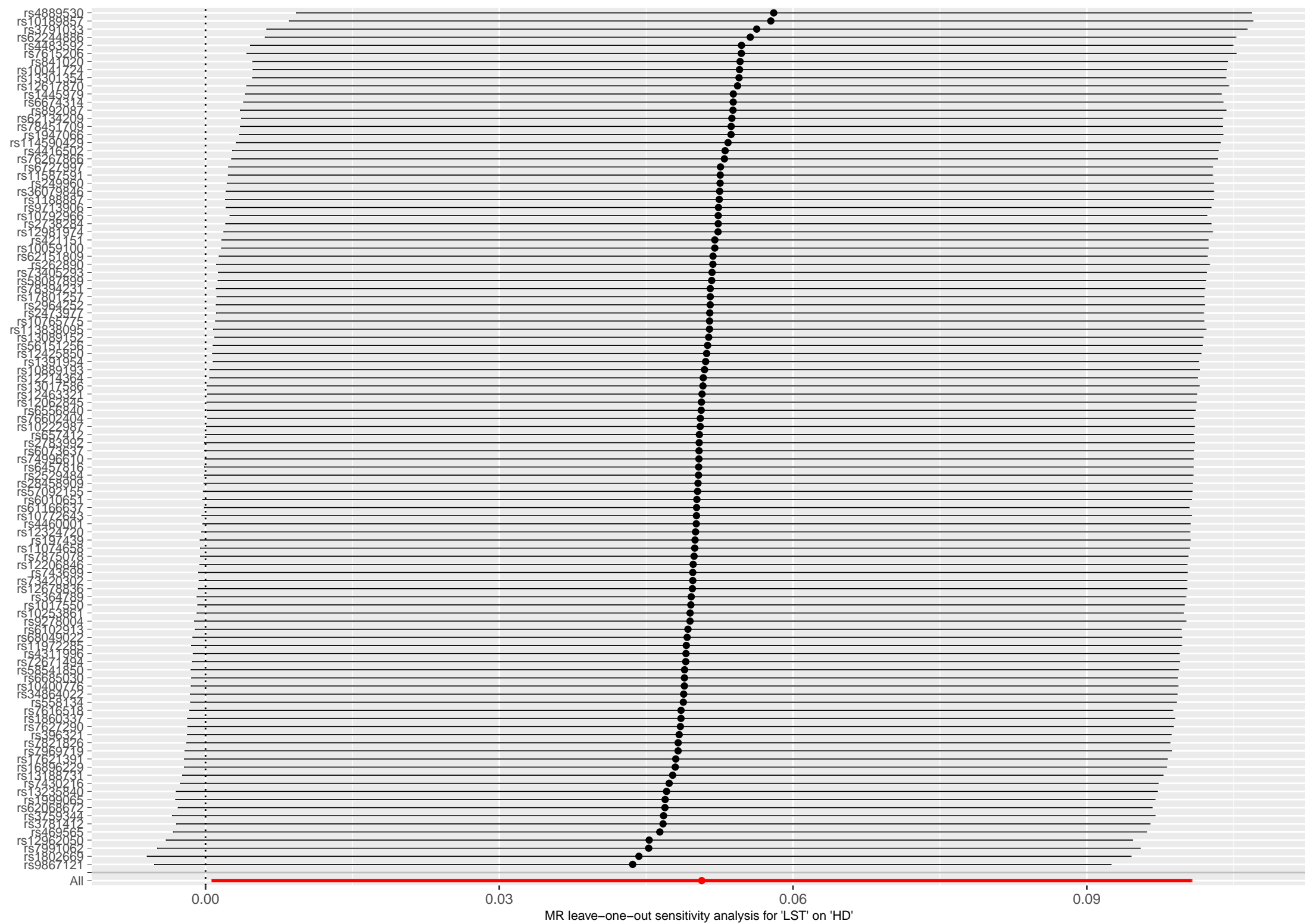

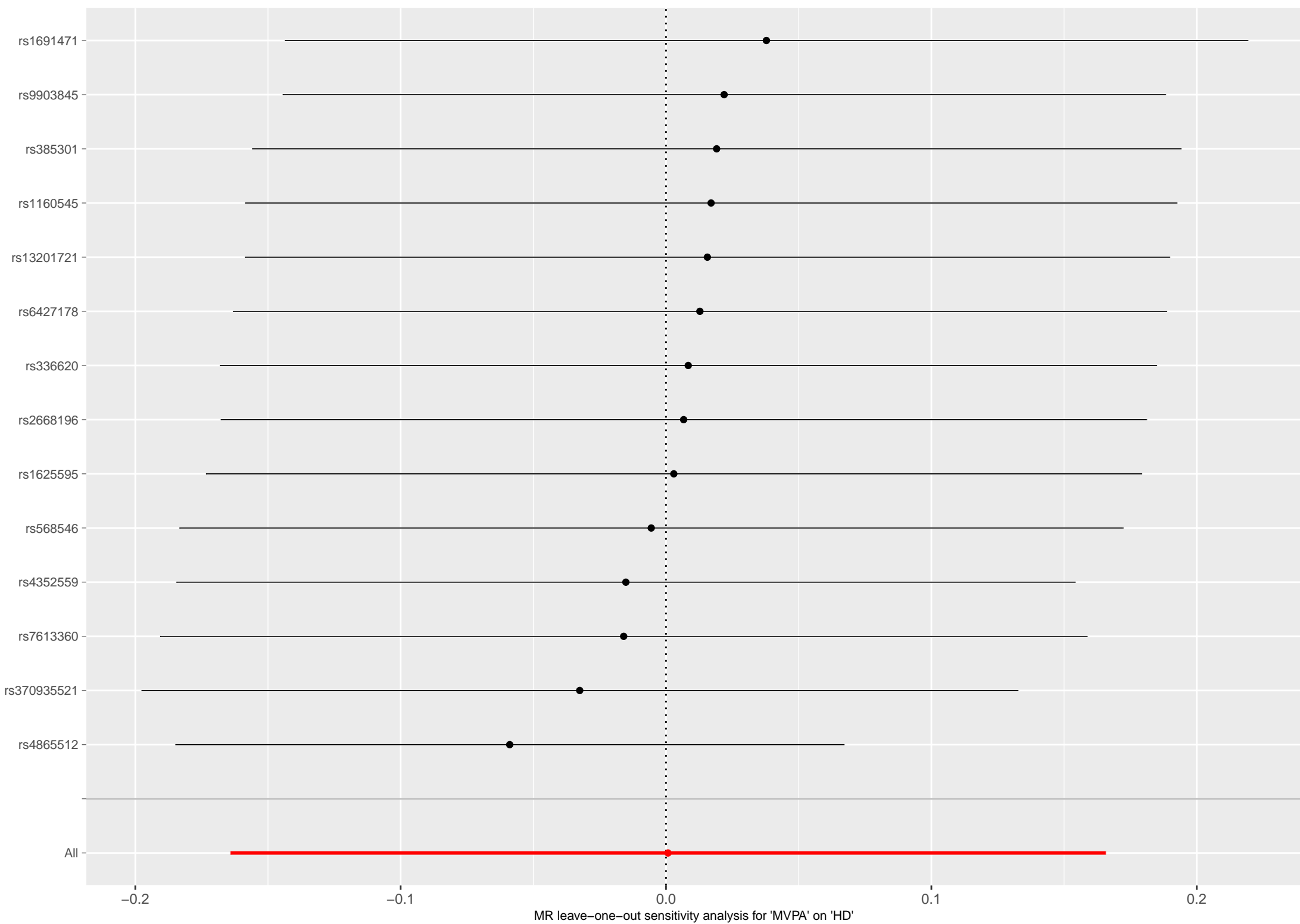

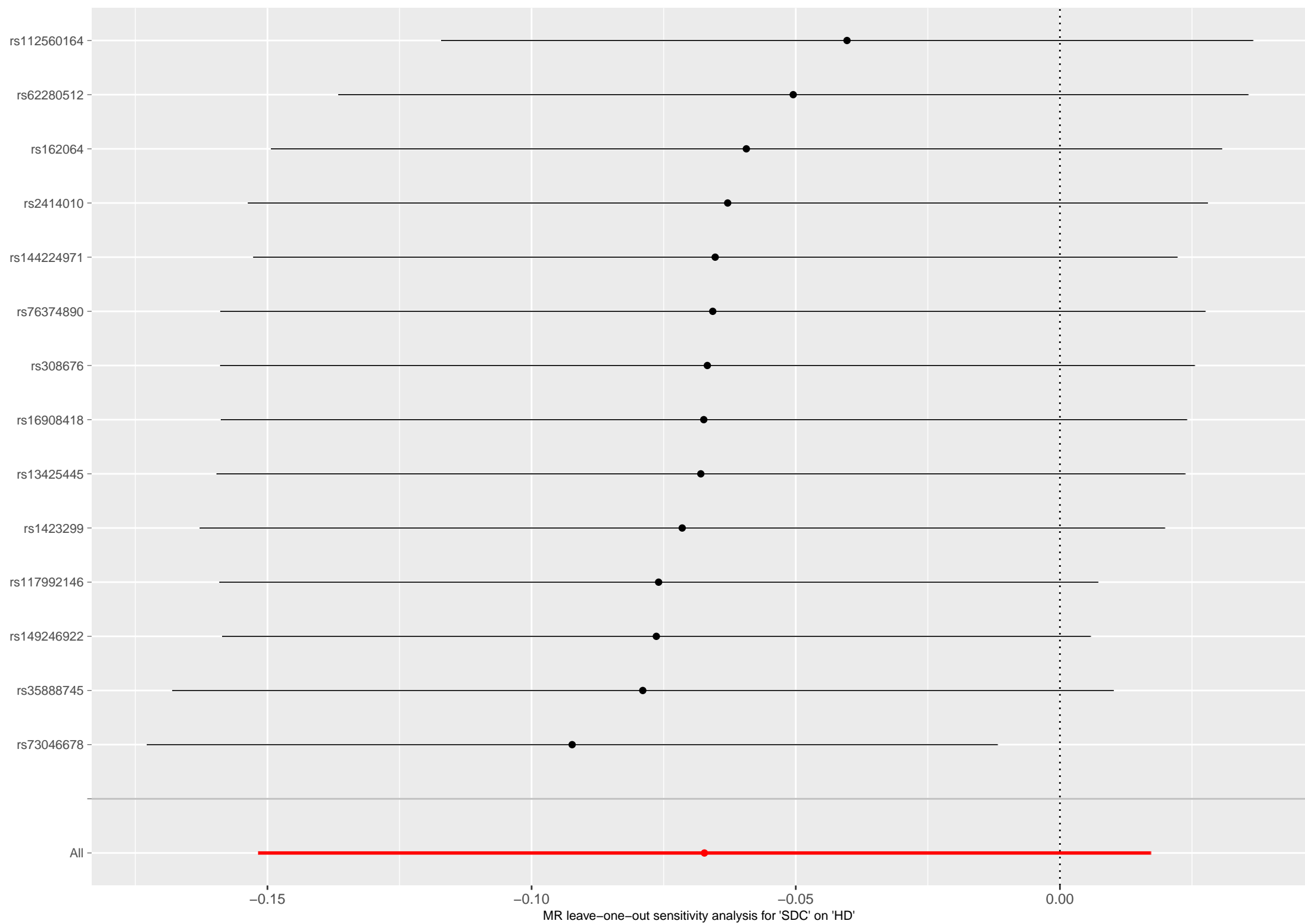

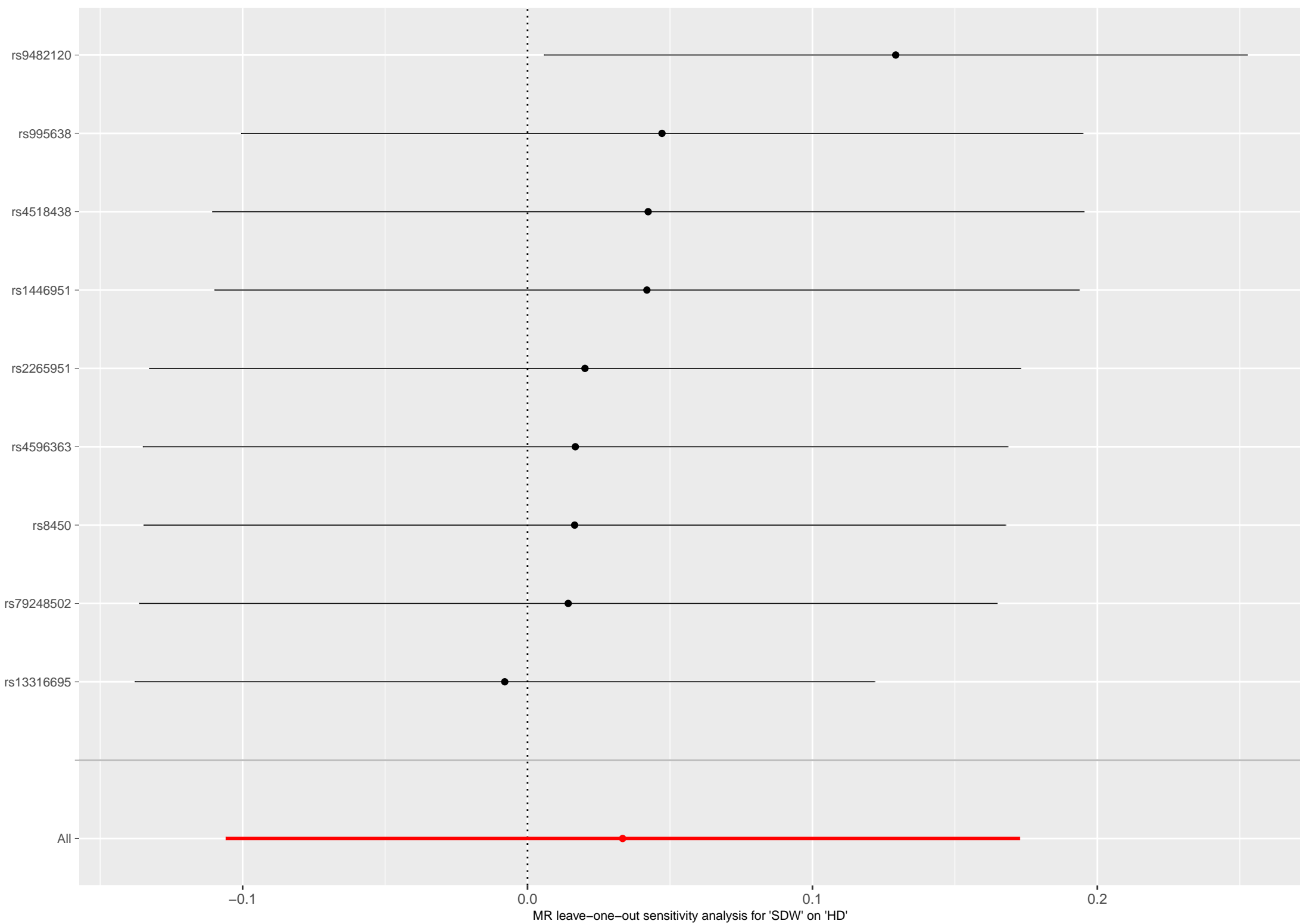

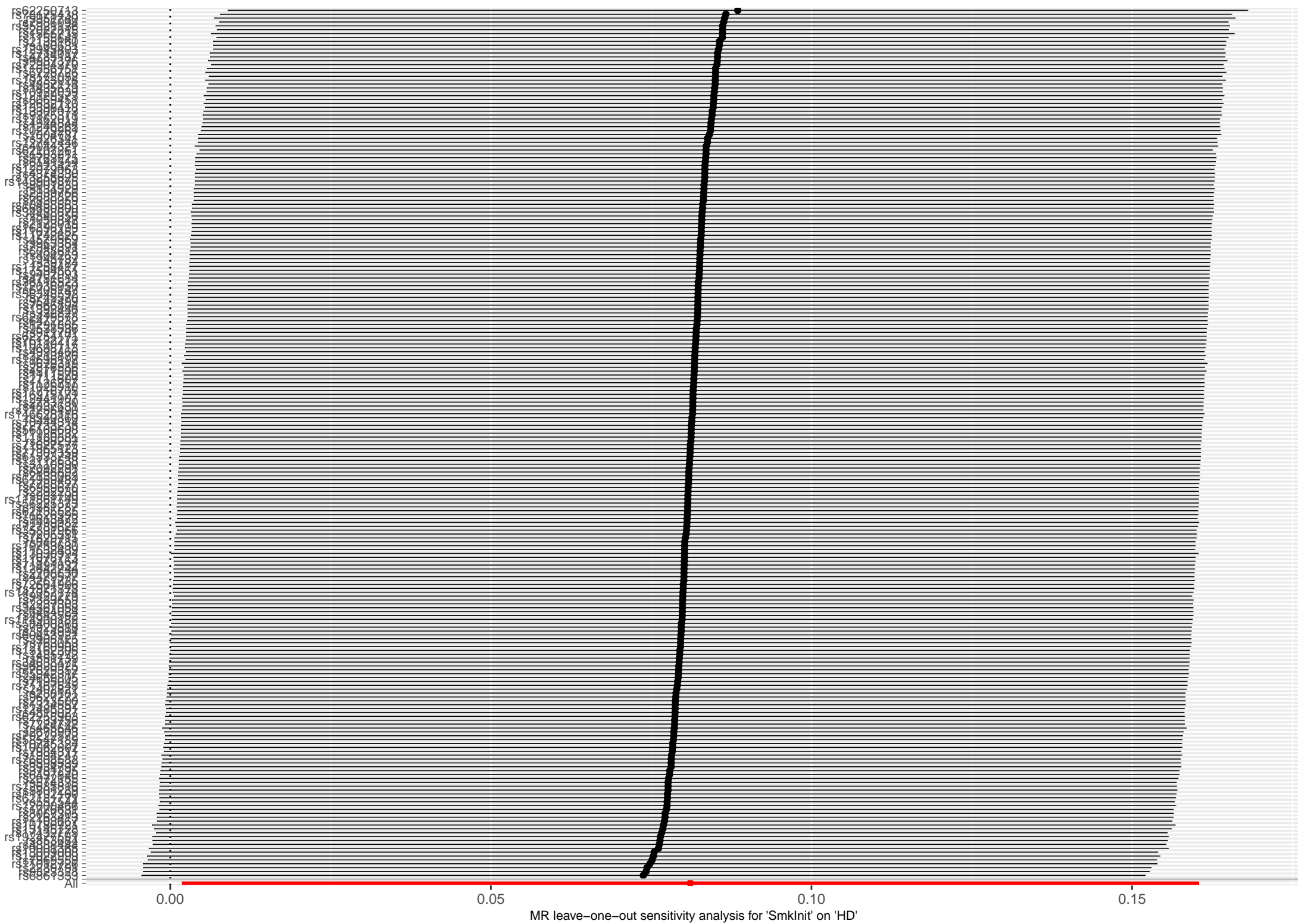

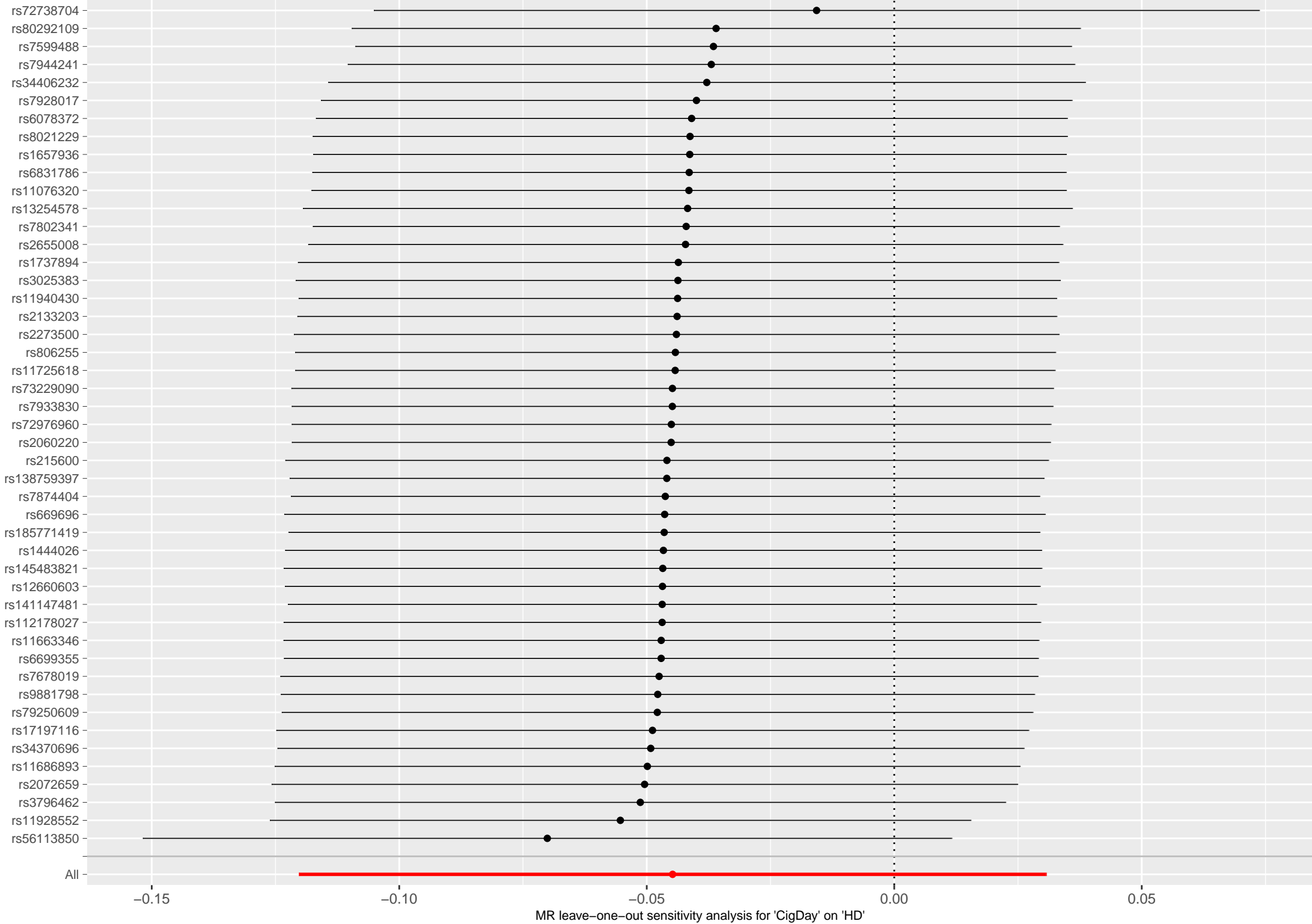

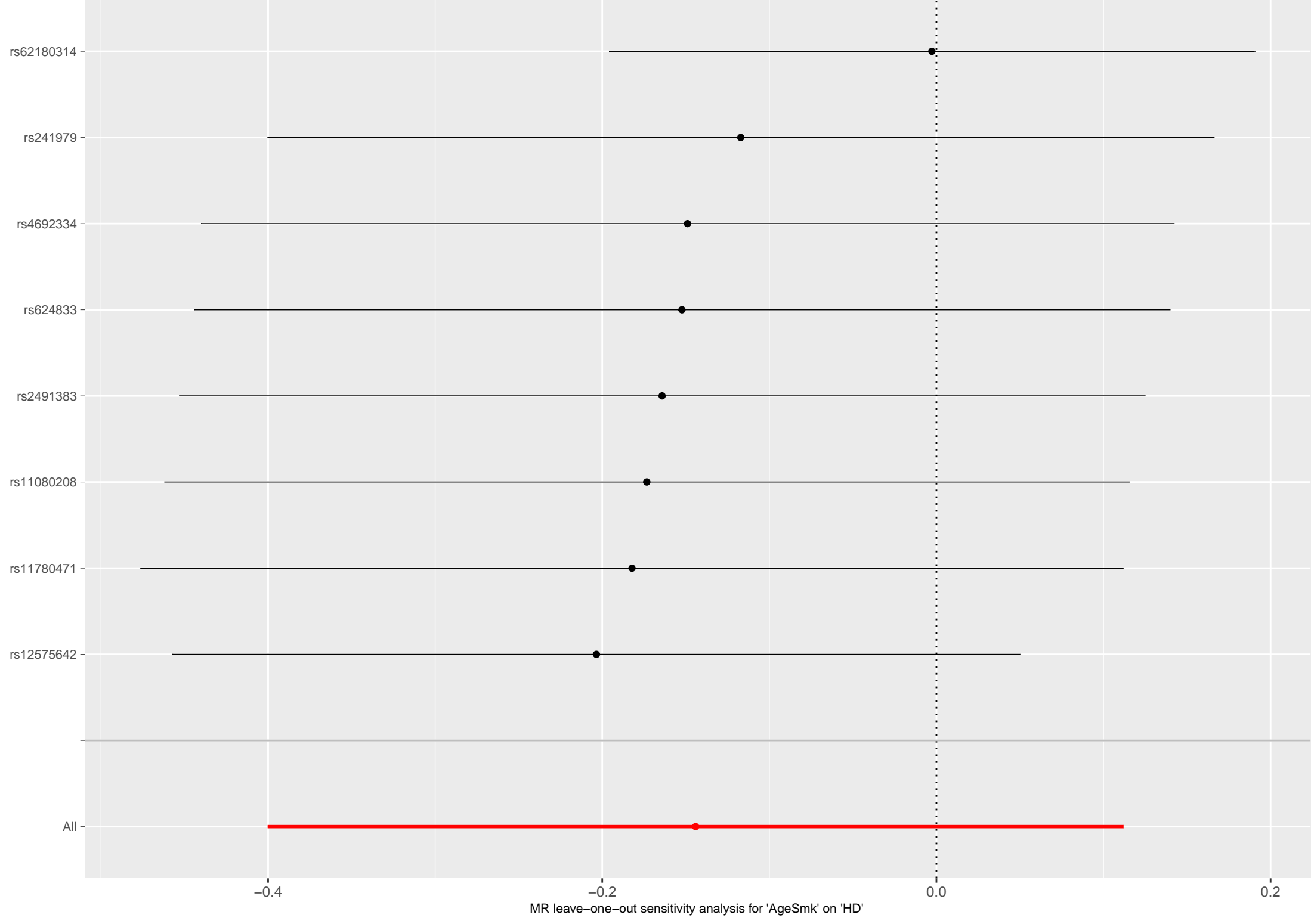

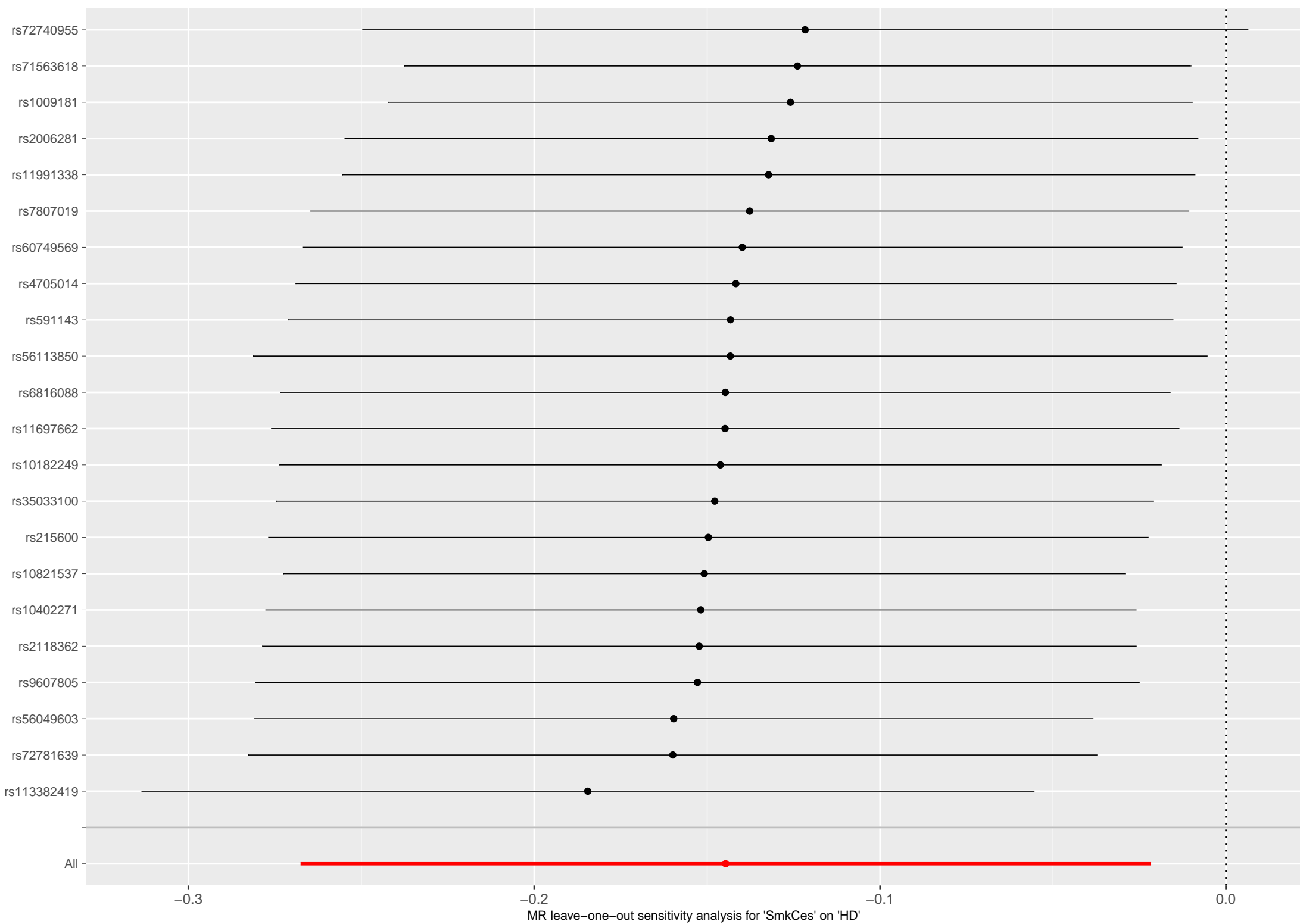

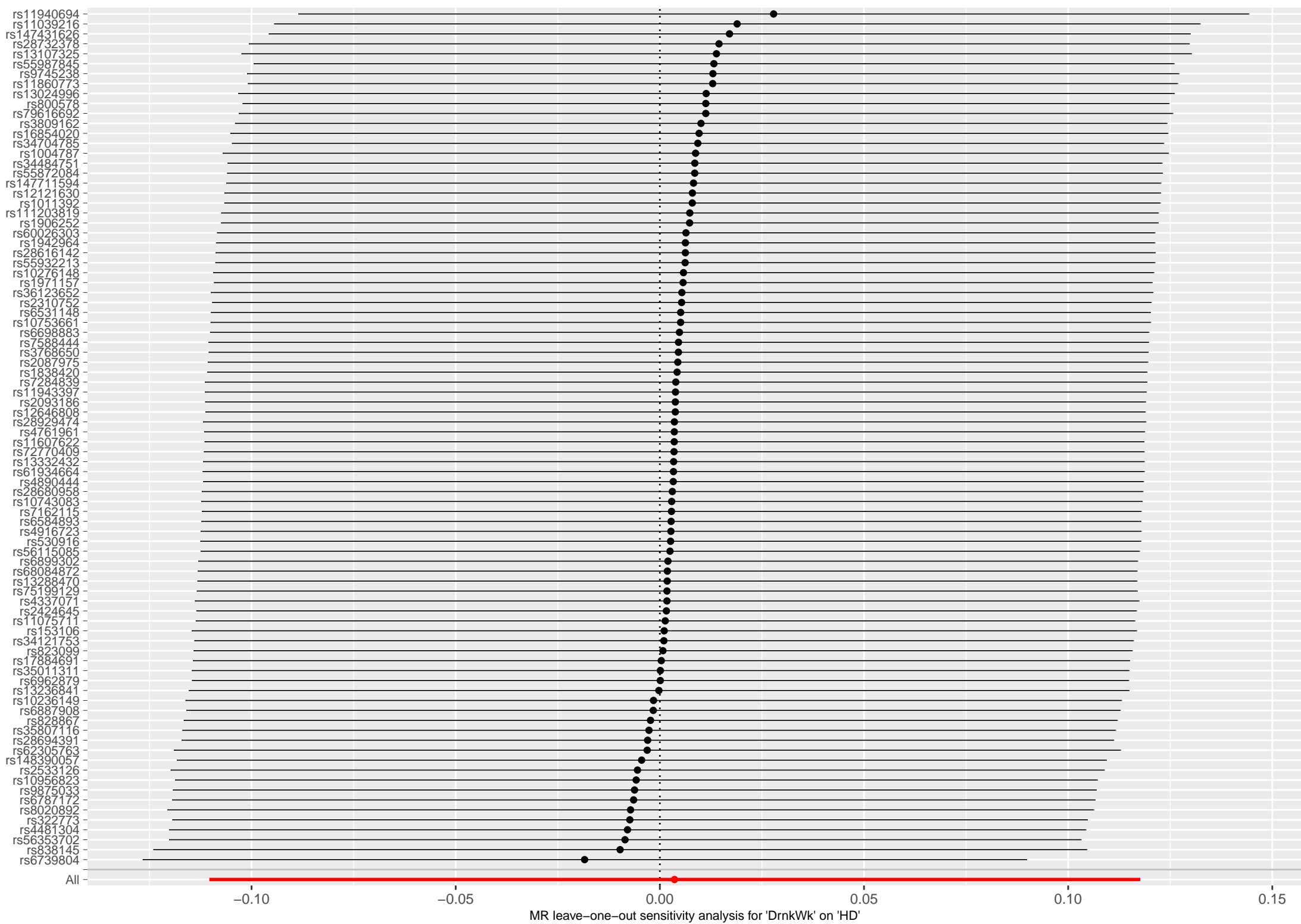

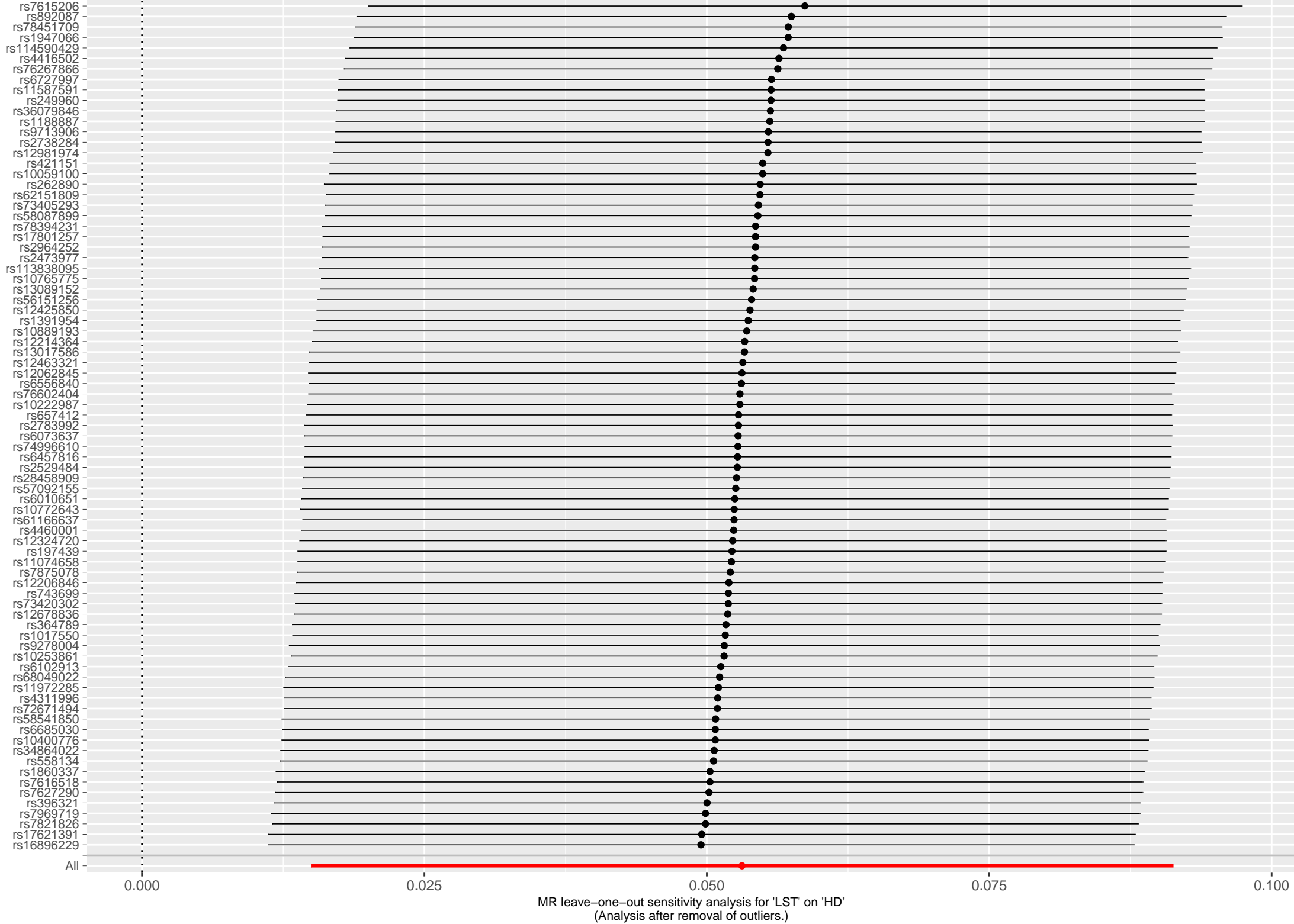

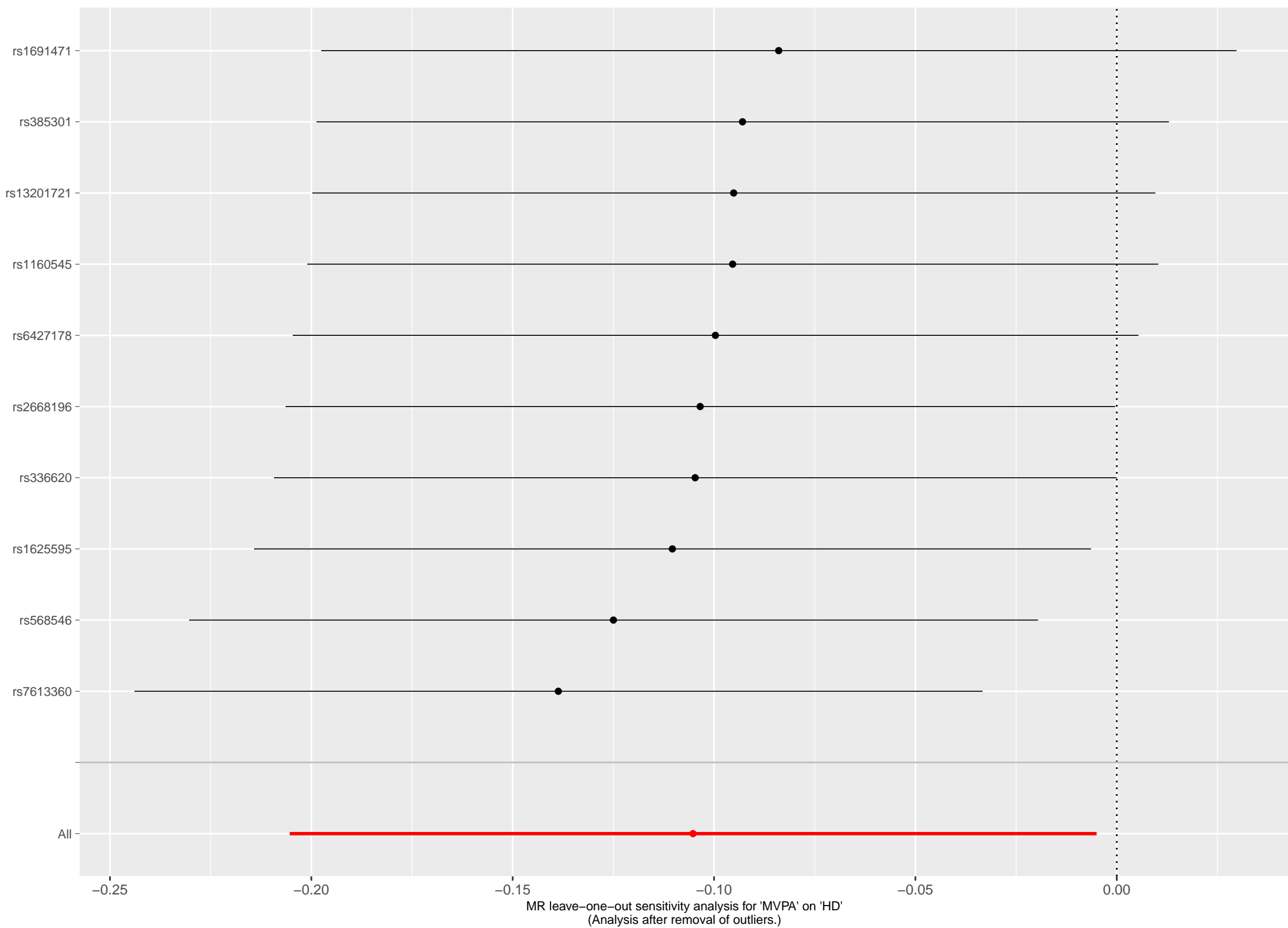

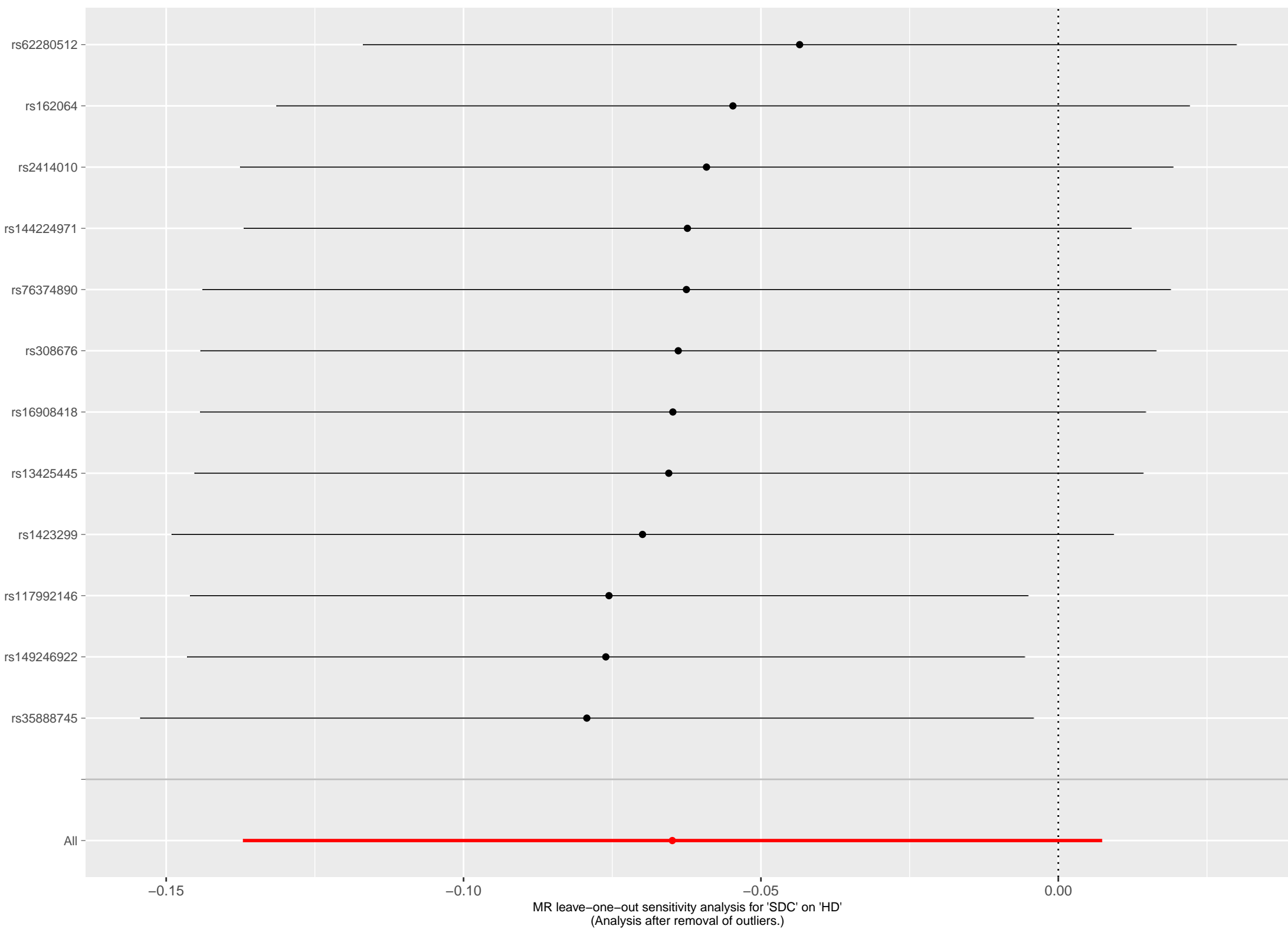

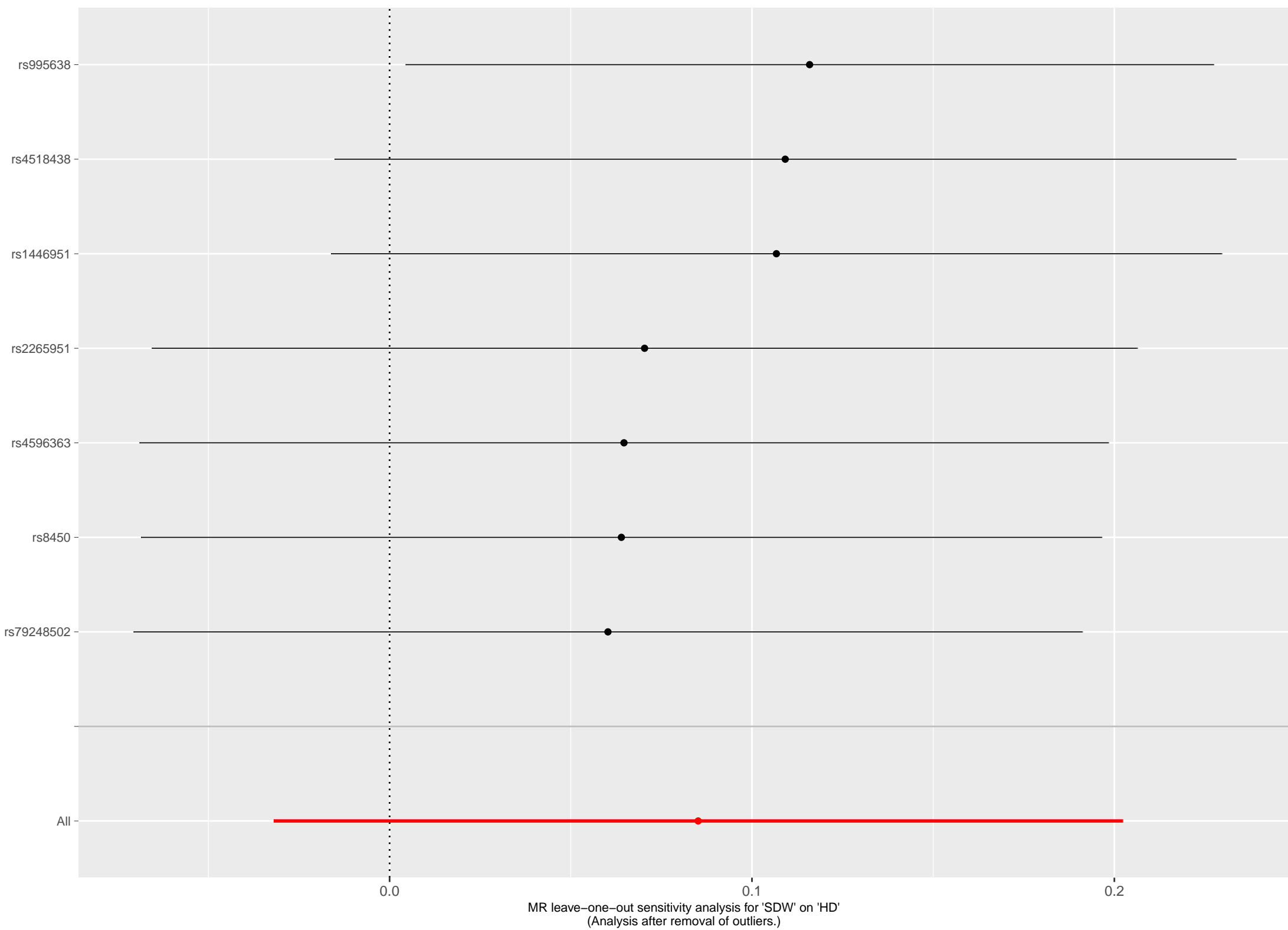

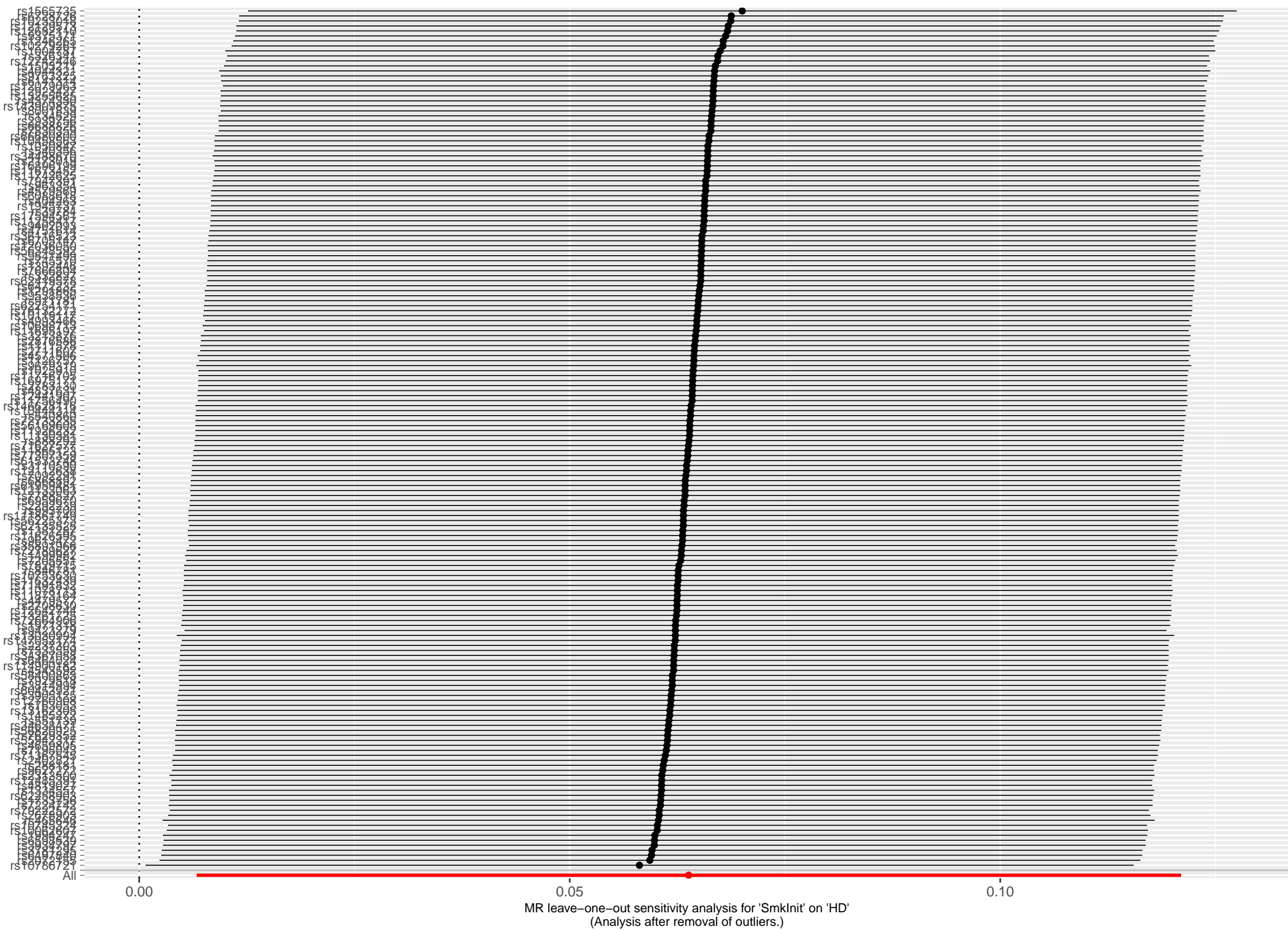

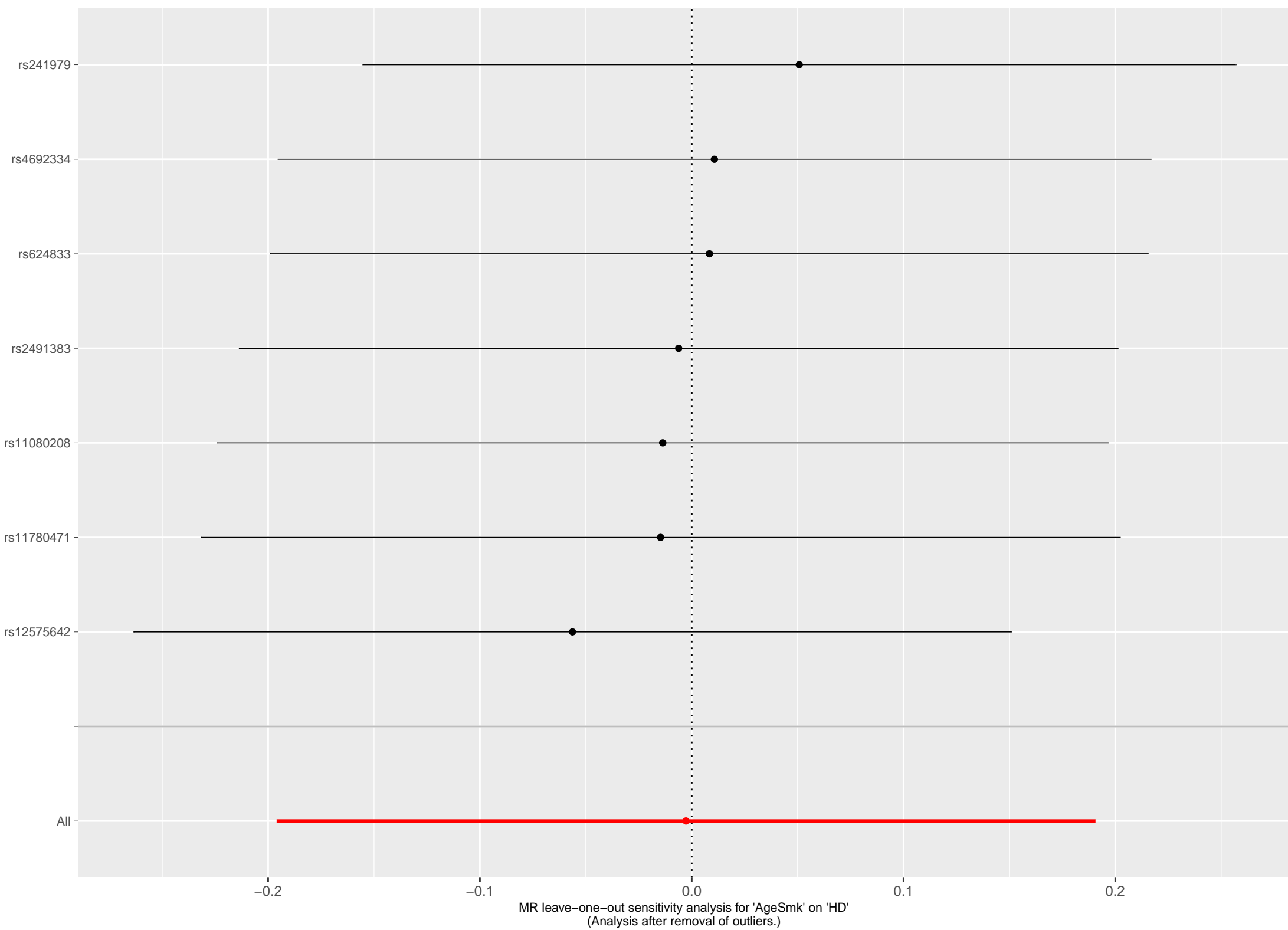

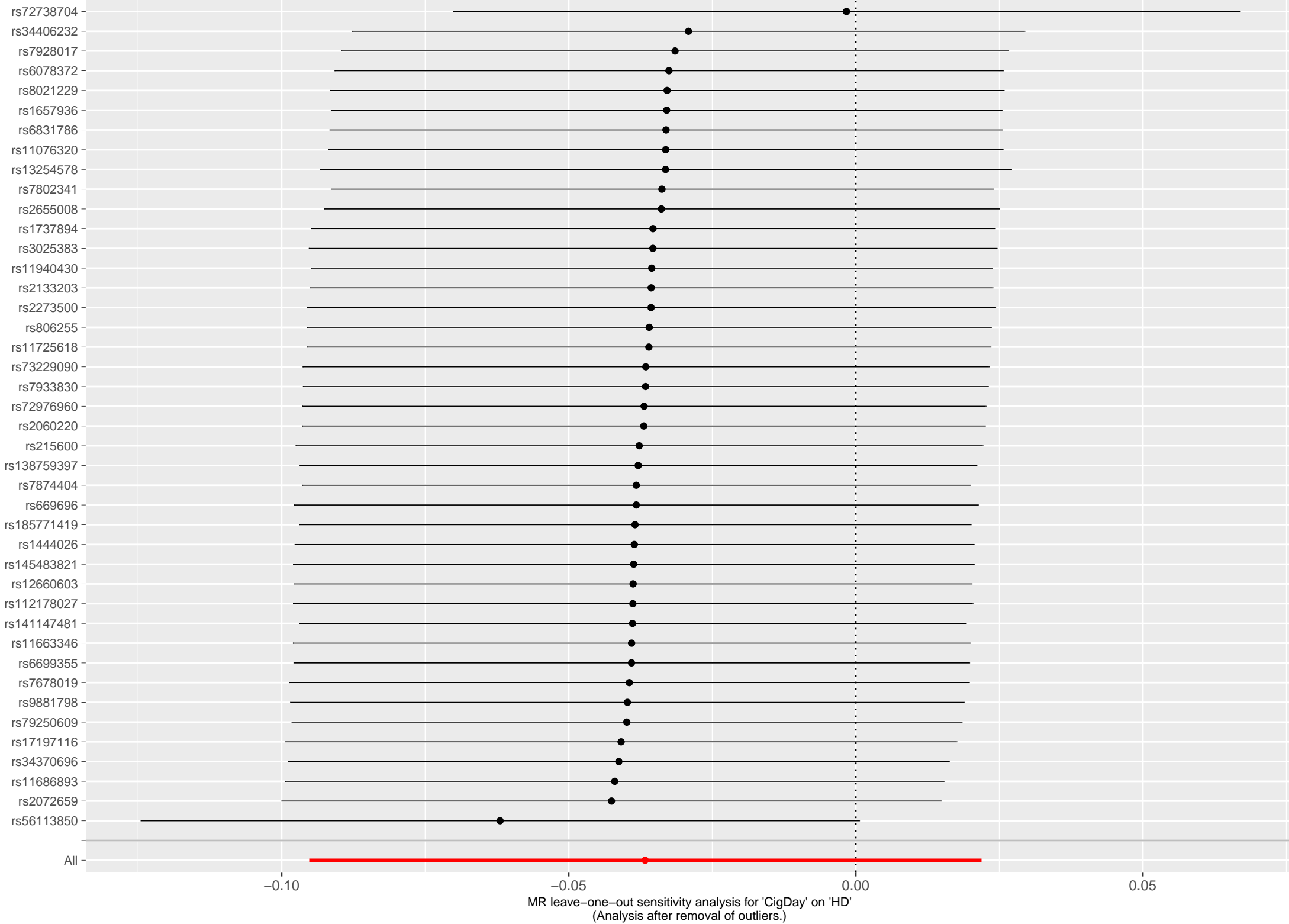

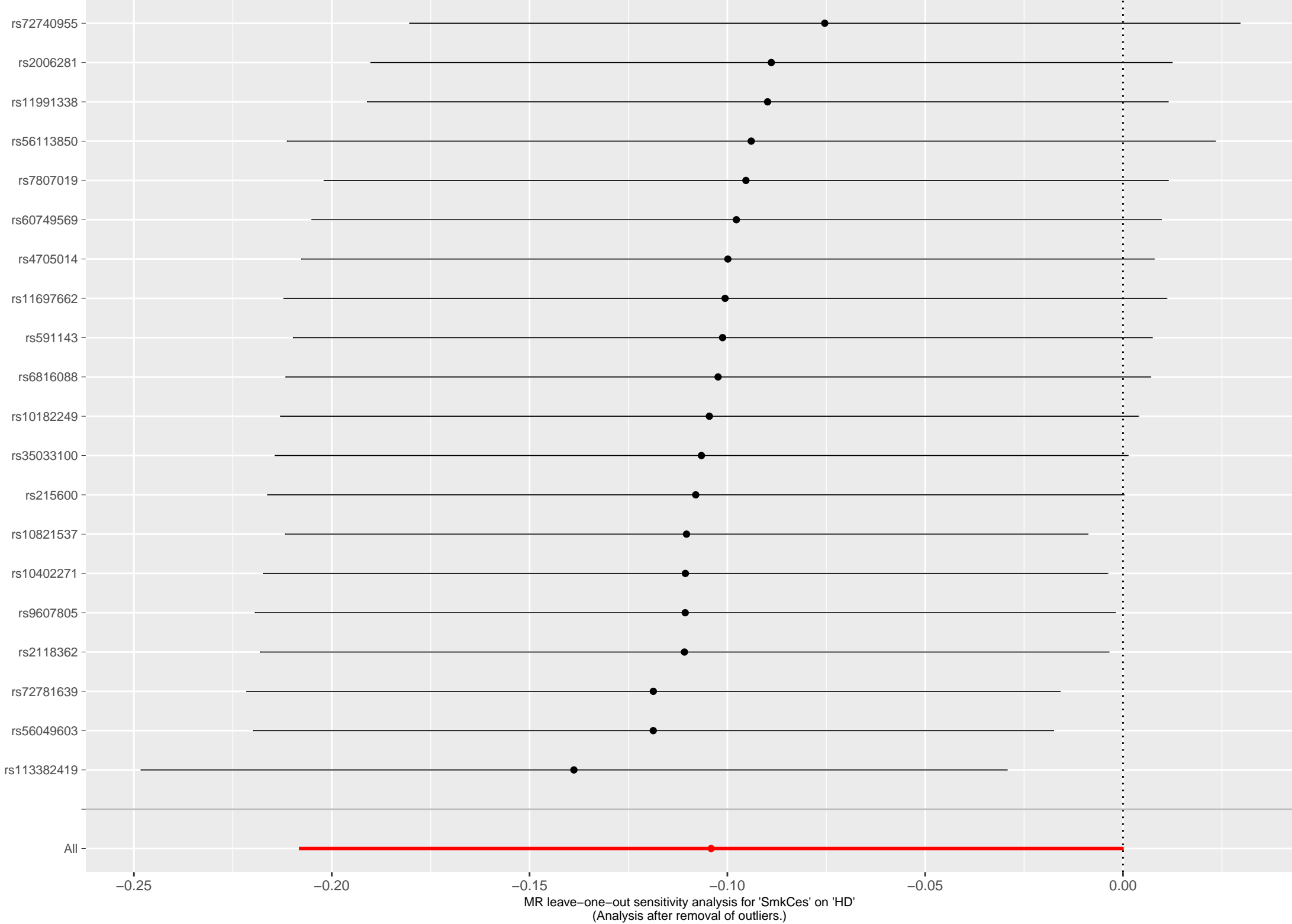

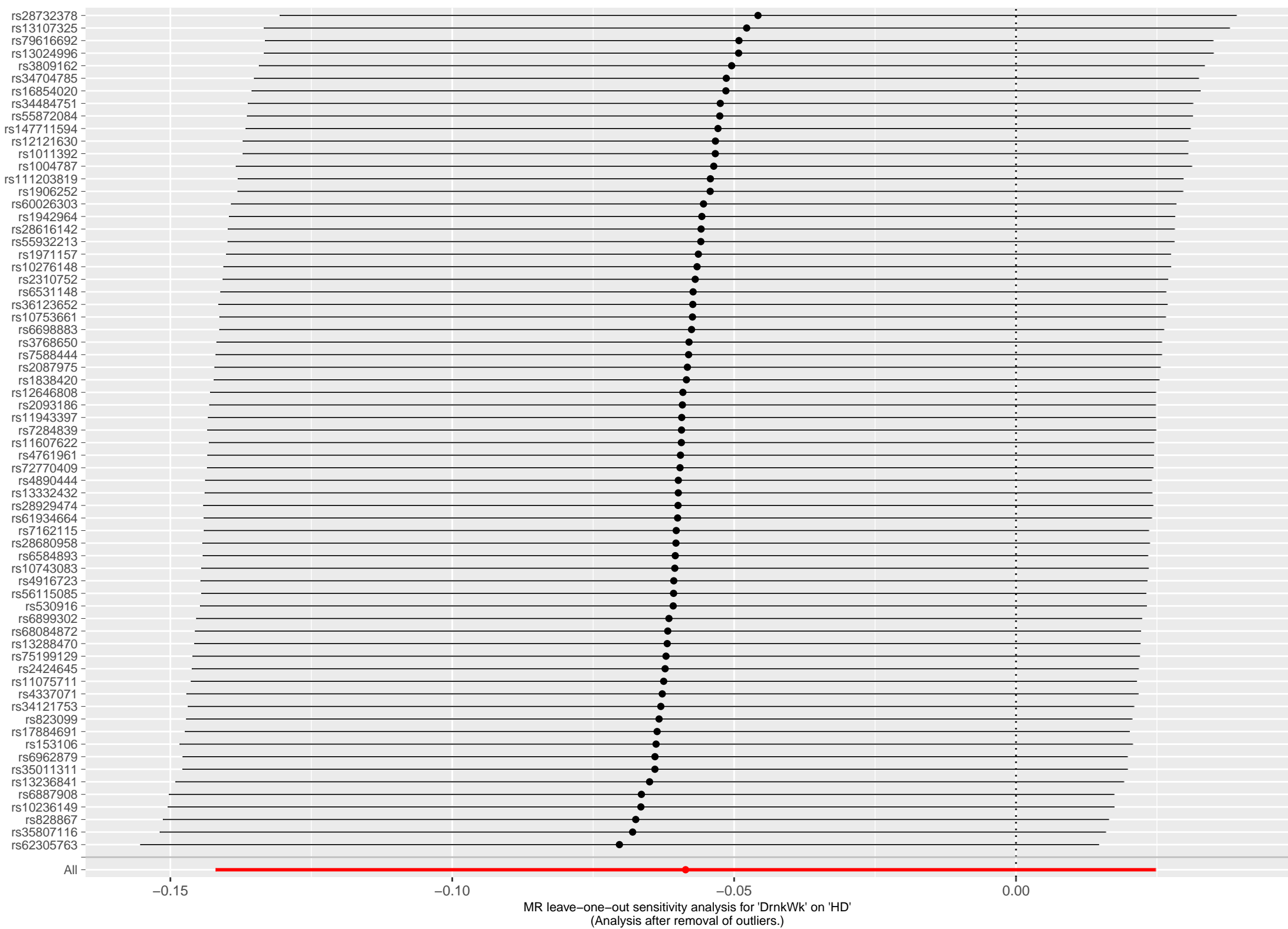

Supplement: Supplementary file 6 [file medi-105-e48945-s006.pdf]
