# Supplementary material for: Causal associations between lifestyle factors and hemorrhoidal disease: Insights from Mendelian randomization analysis
Source: Medicine (Baltimore). 2026 May 22;105(21):e48945. doi: 10.1097/MD.0000000000048945 (PMC13200937; doi:10.1097/MD.0000000000048945)
Supplement: Supplementary file 7 [file medi-105-e48945-s007.pdf]

IVW Radial analysis for 'LST' on 'HD'

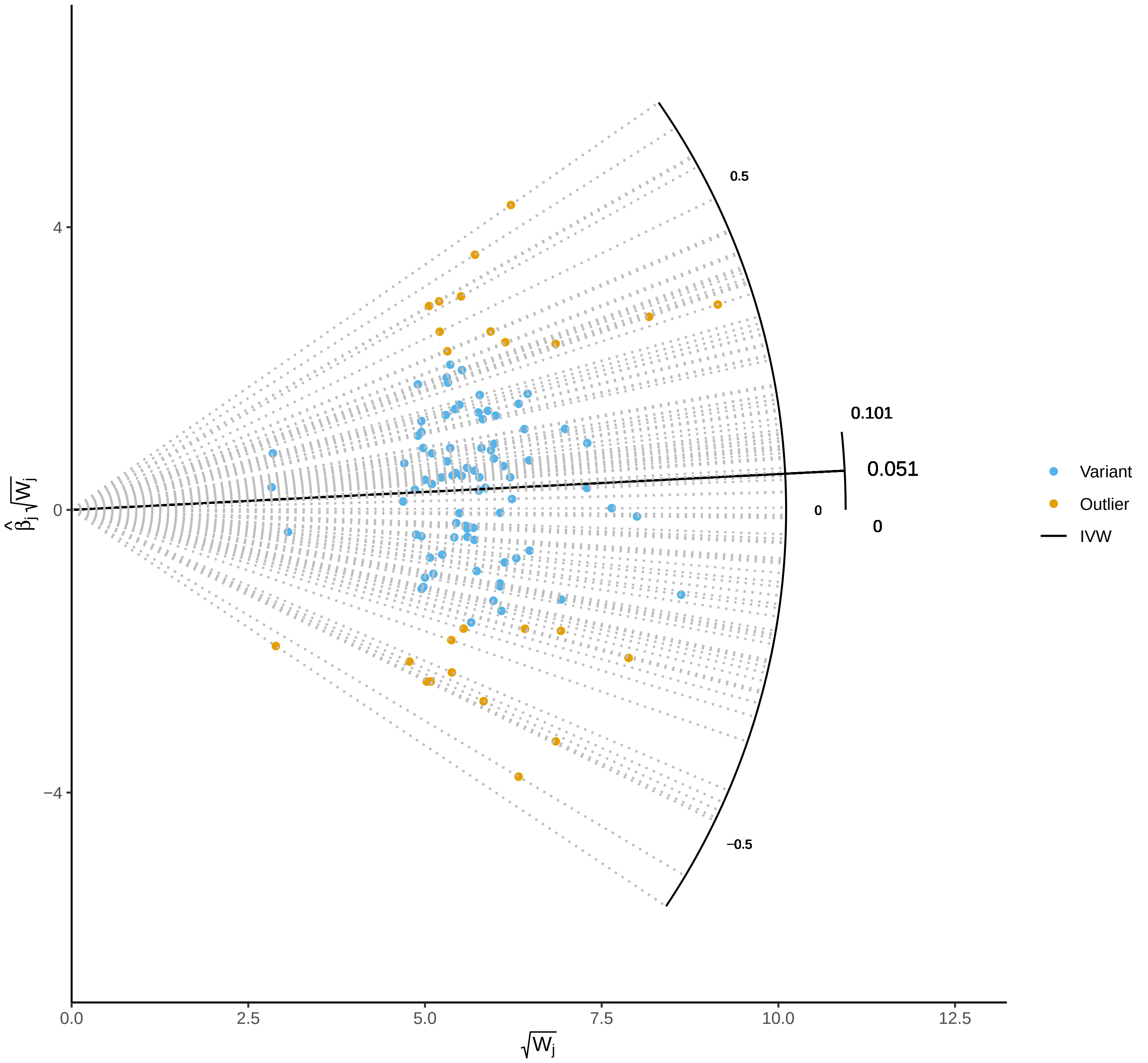

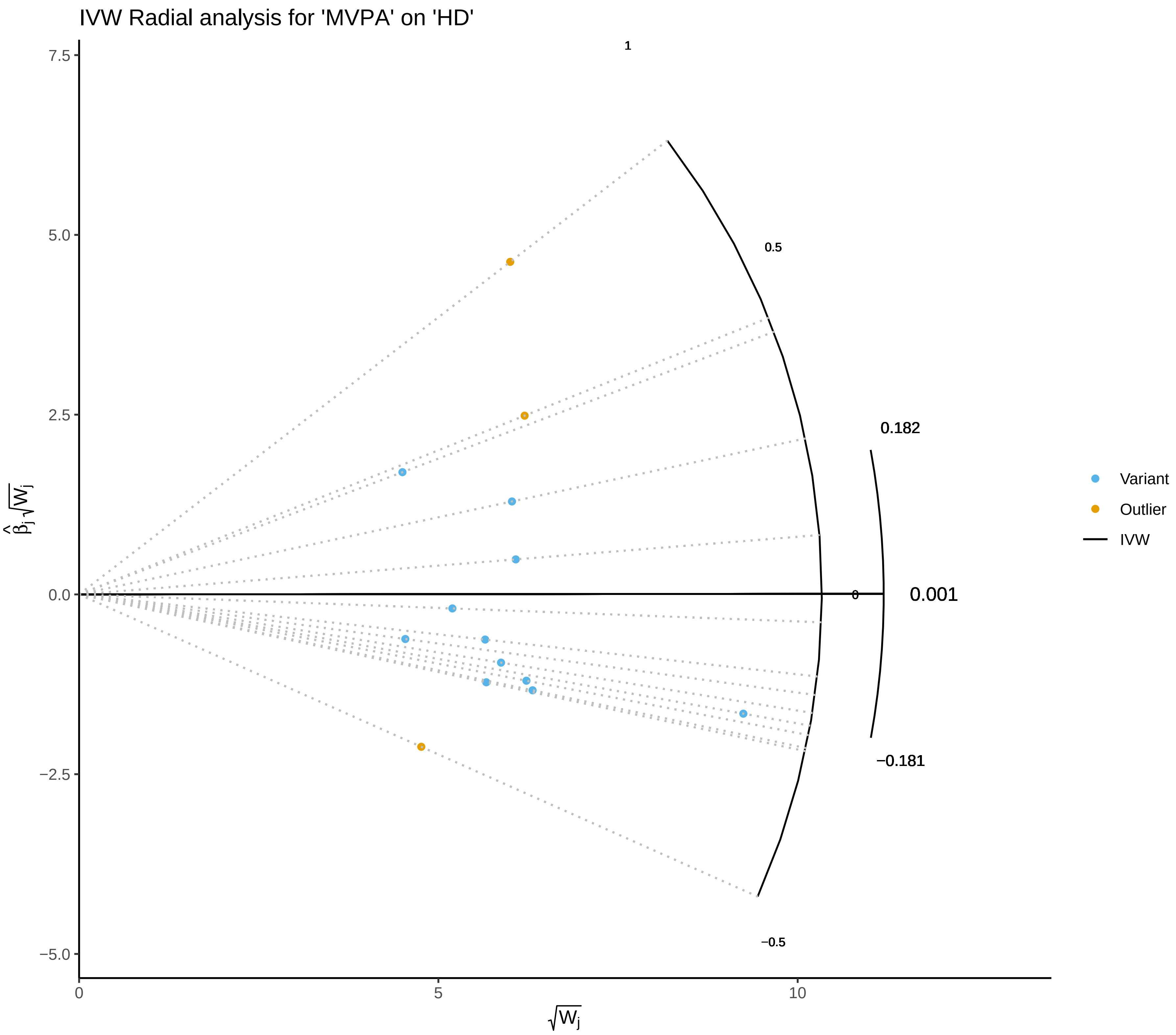

IVW Radial analysis for 'MVPA' on 'HD'
7.5
5.0
2.5
0.0
−2.5
−5.0
0
5
10
1
0.5
0.182
0
0.001
−0.181
−0.5
Variant
Outlier
IVW

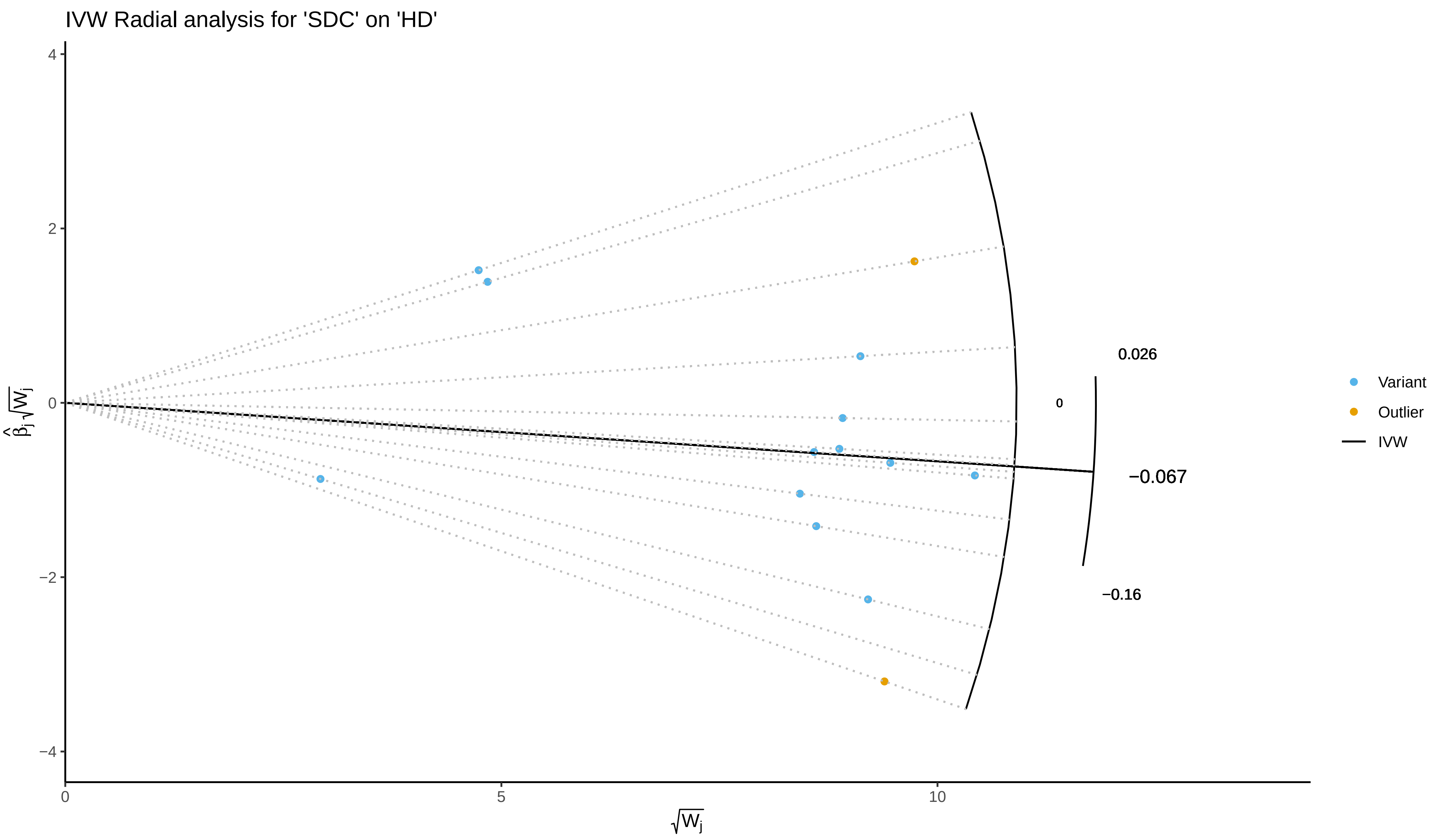
IVW Radial analysis for 'SDC' on 'HD'
4
2
0
−2
−4
$\hat{\beta}_j \sqrt{W_j}$
0
5
10
$\sqrt{W_j}$
0.026
0
−0.067
−0.16
Variant
Outlier
IVW

IVW Radial analysis for 'SDW' on 'HD'

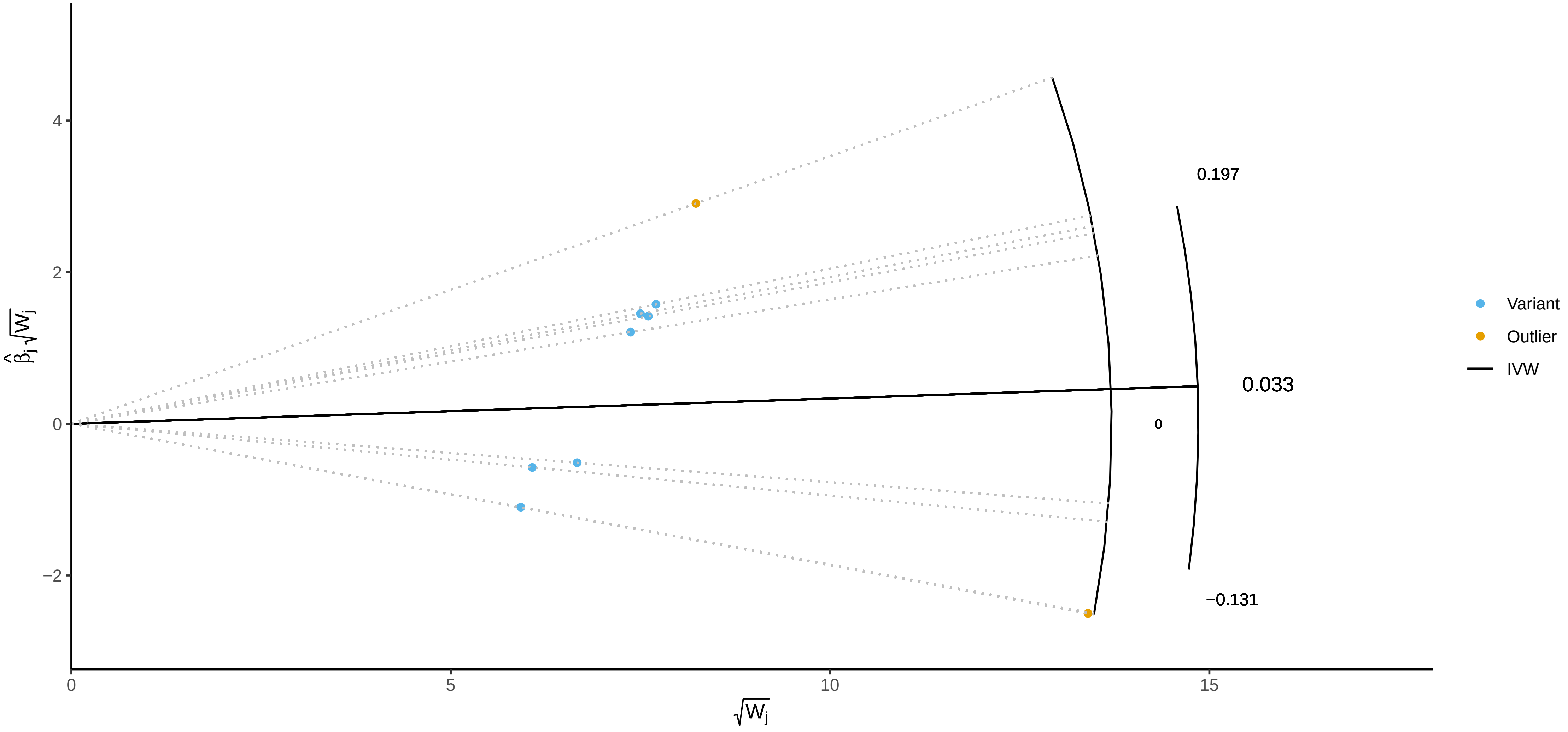

IVW Radial analysis for 'SmkInit' on 'HD'

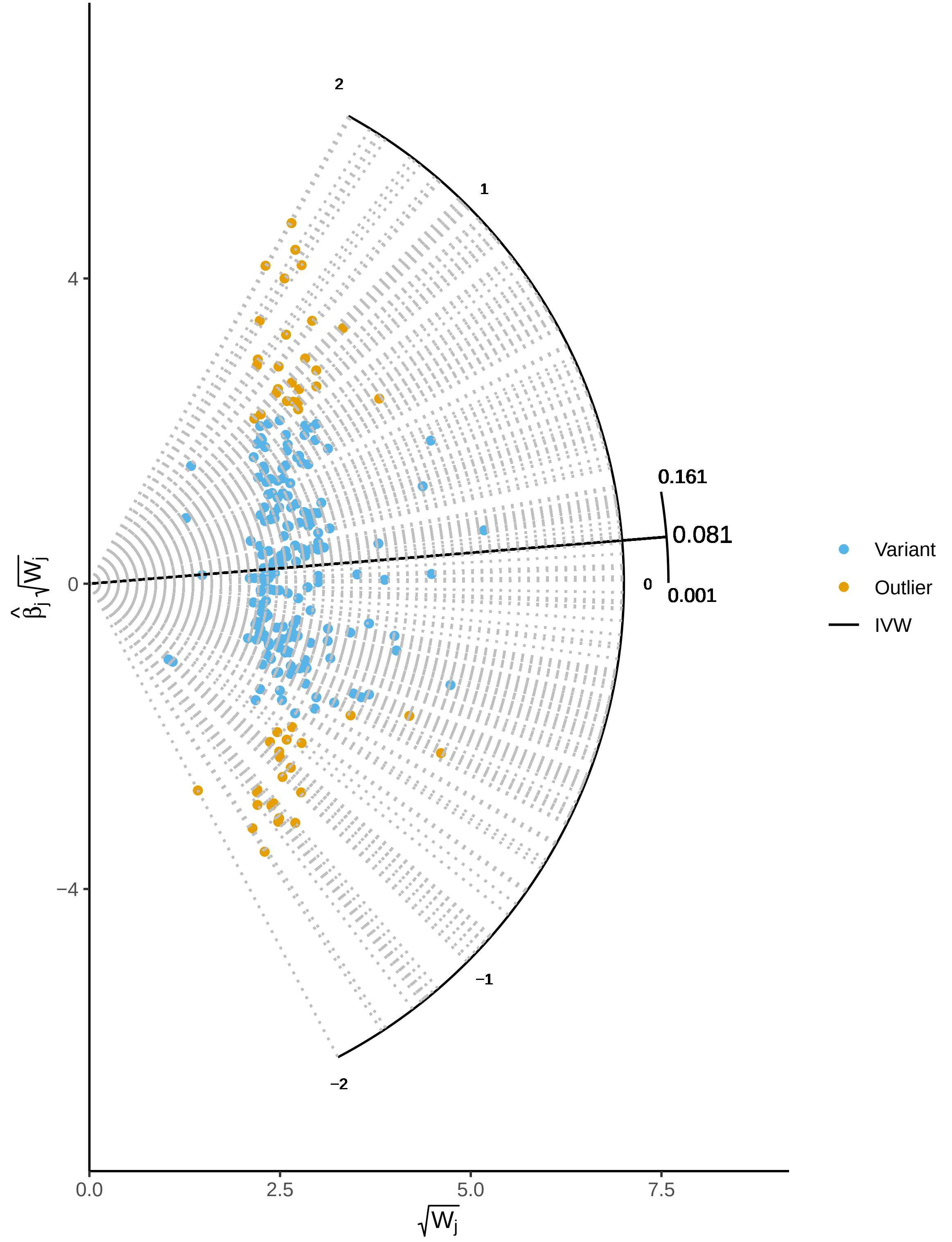

IVW Radial analysis for 'CigDay' on 'HD'

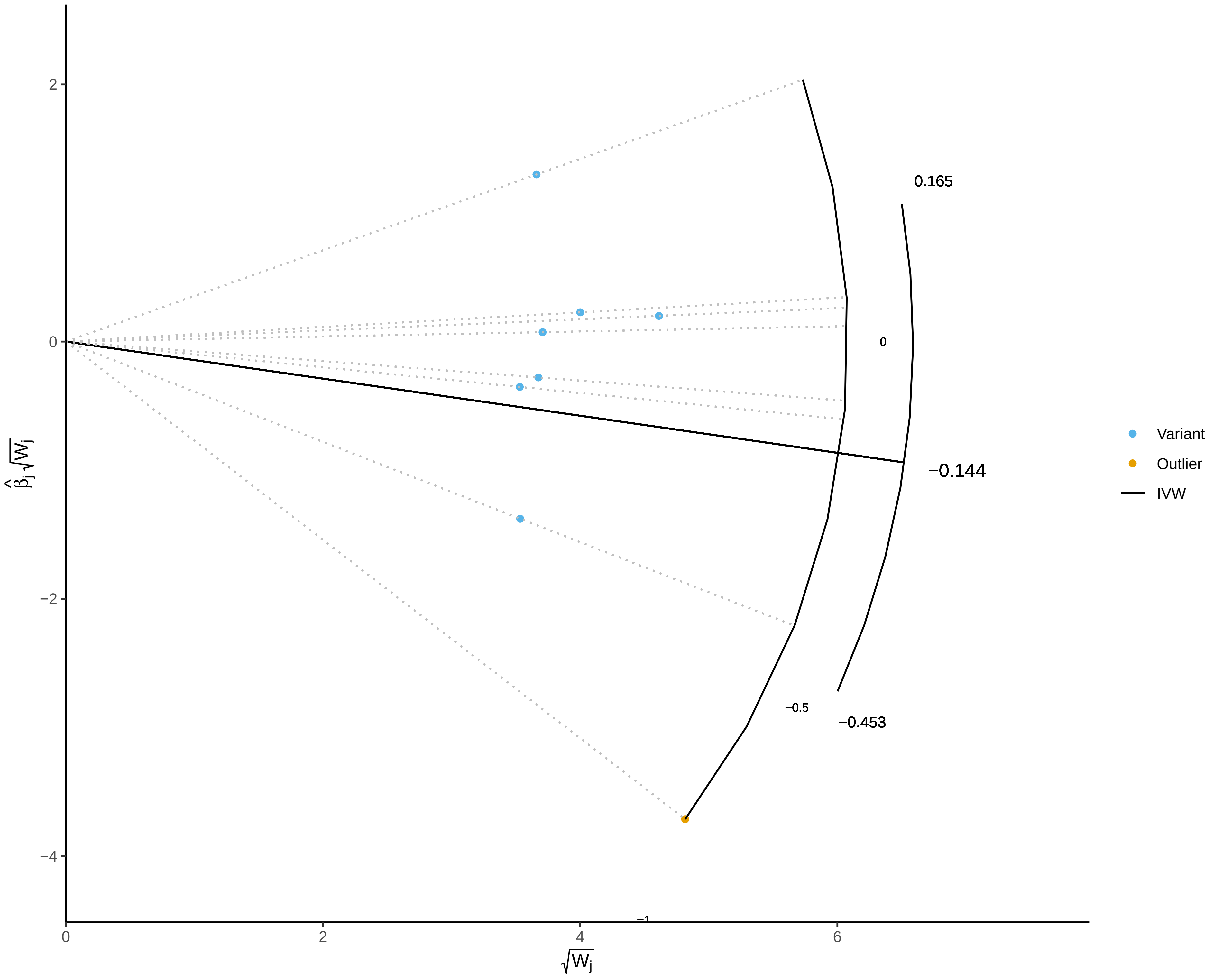

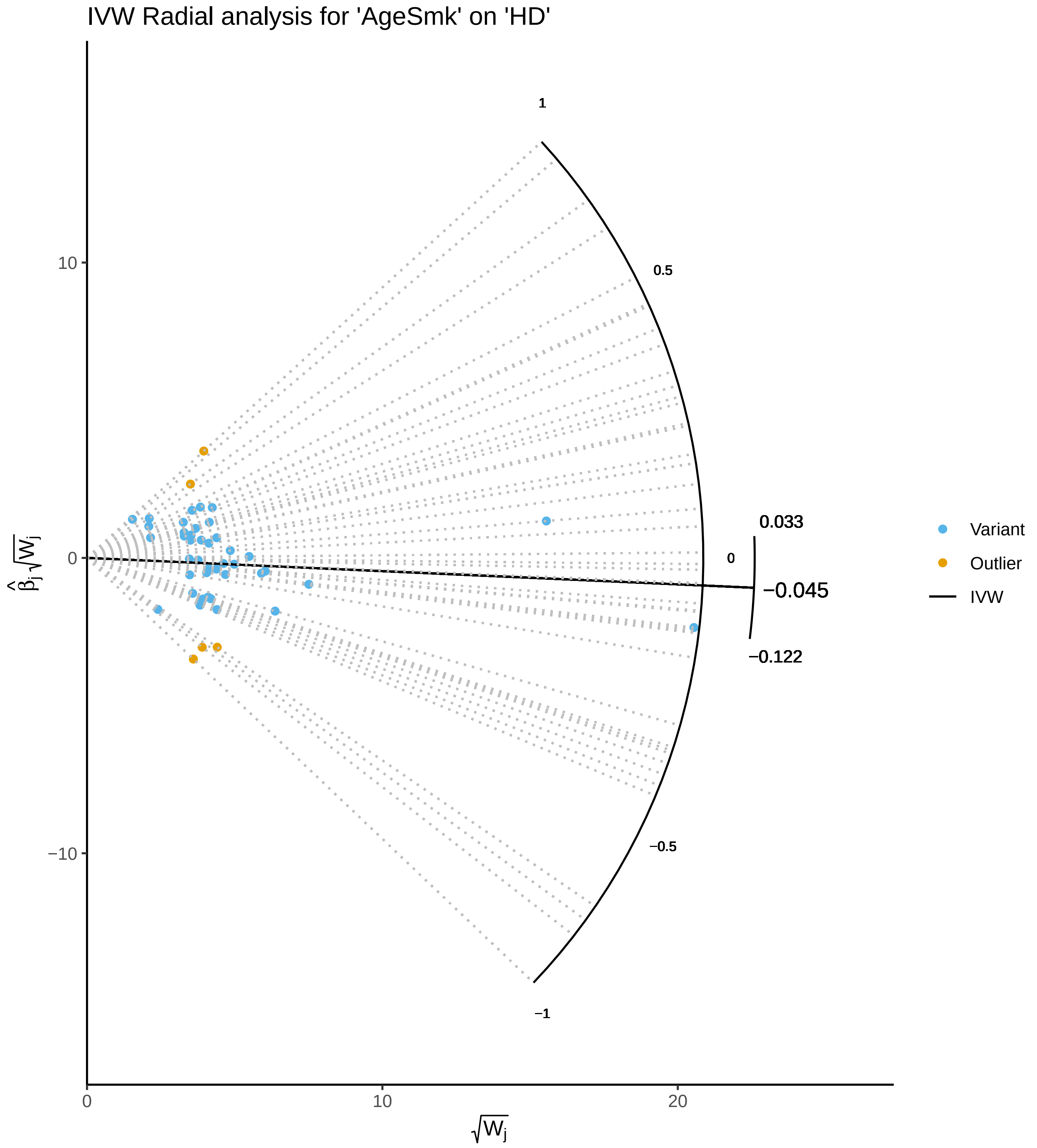

IVW Radial analysis for 'AgeSmk' on 'HD'
10
0
−10
0
10
20
$\hat{\beta}_j \sqrt{W_j}$
$\sqrt{W_j}$
1
0.5
0
−0.5
−1
0.033
−0.045
−0.122
Variant
Outlier
IVW

IVW Radial analysis for 'SmkCes' on 'HD'

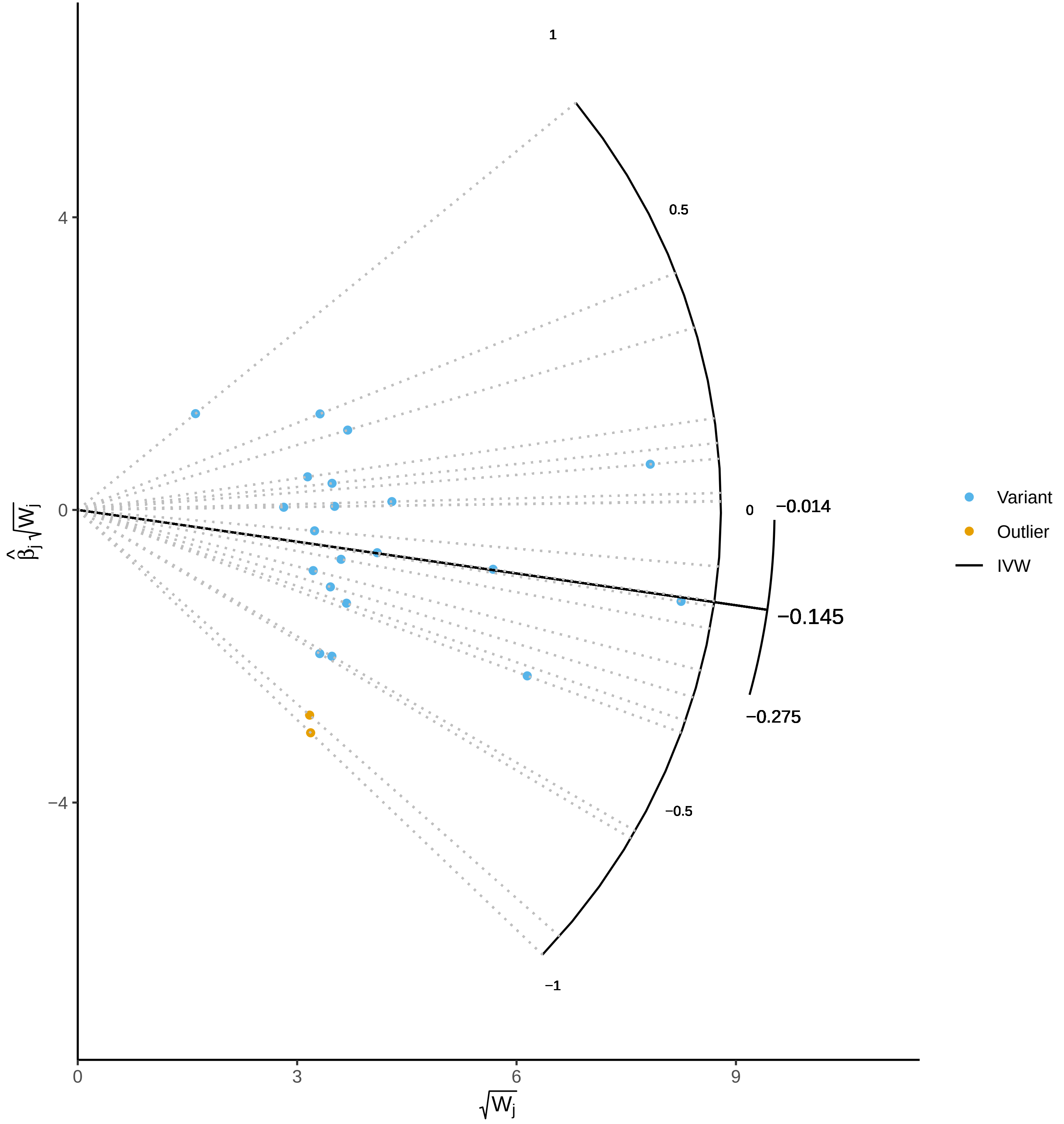

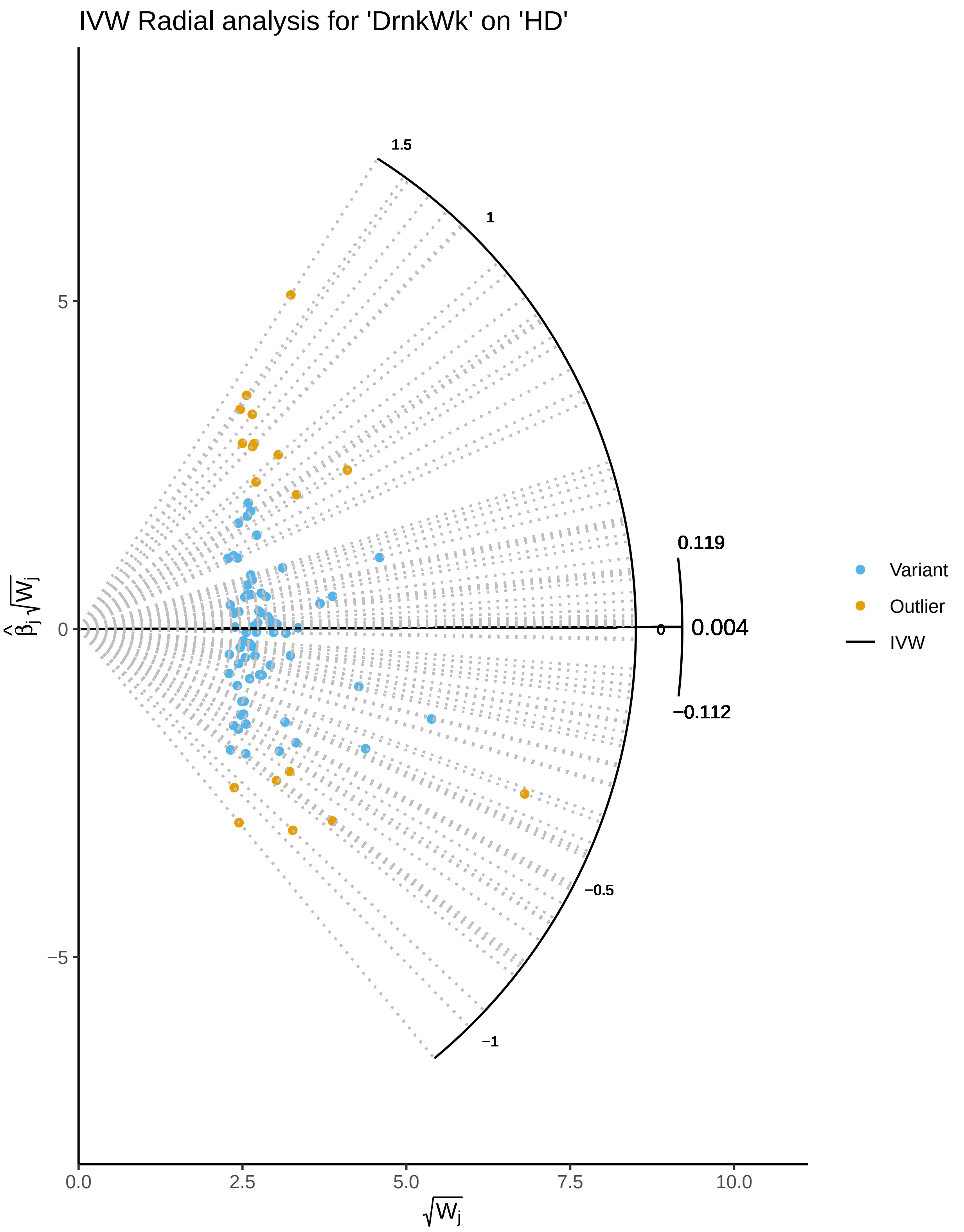
IVW Radial analysis for 'DrnkWk' on 'HD'
$\hat{\beta}_j\sqrt{W_j}$
$\sqrt{W_j}$
5
0
−5
0.0
2.5
5.0
7.5
10.0
1.5
1
0
−0.5
−1
0.119
0.004
−0.112
Variant
Outlier
IVW

IVW Radial analysis for 'LST' on 'HD' (Analysis after removal of outliers.)

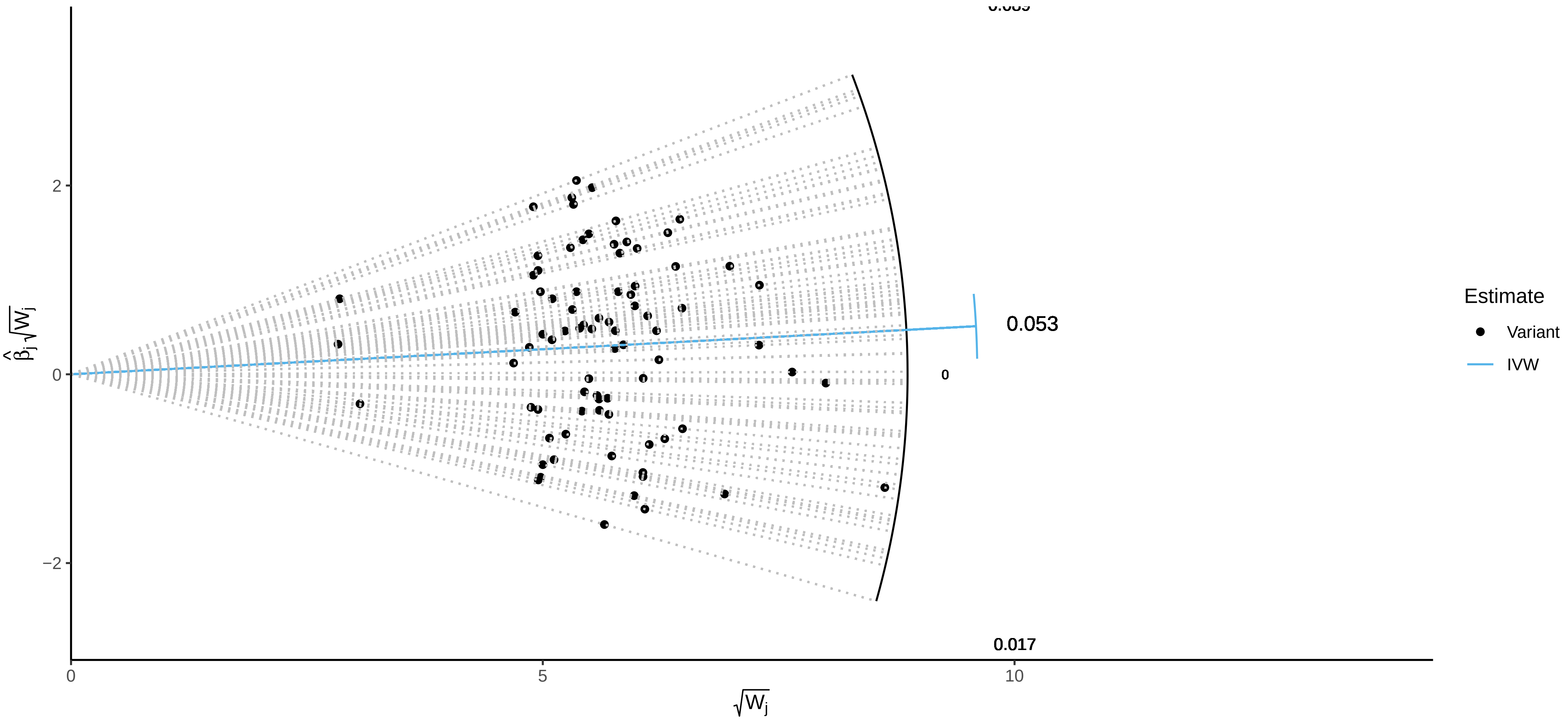

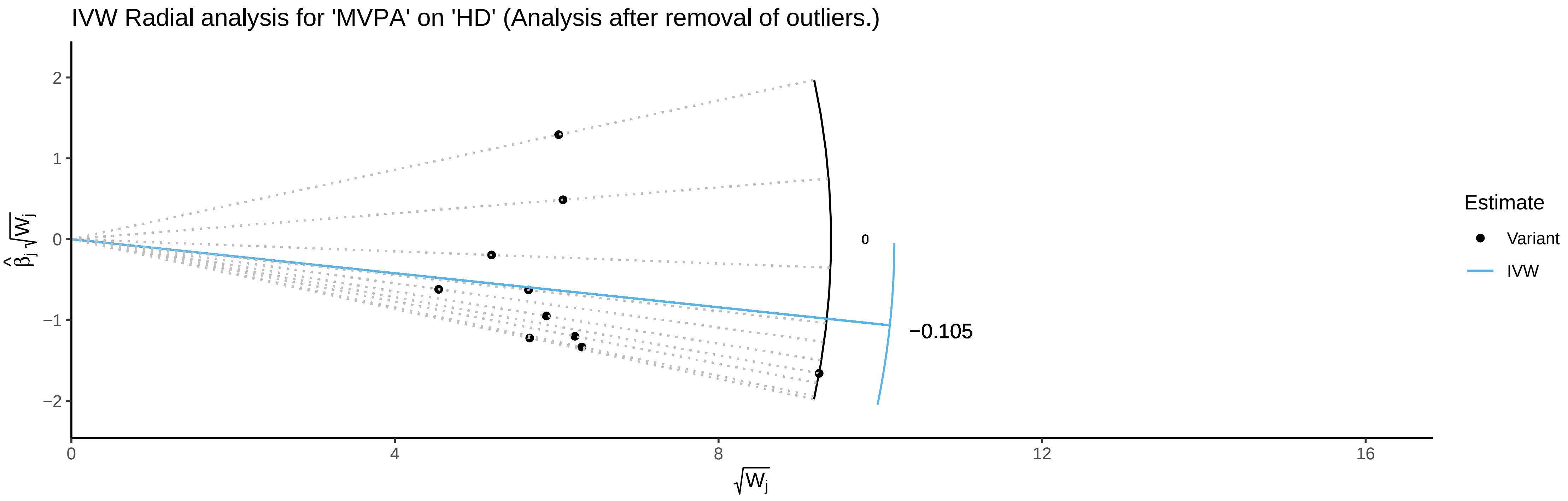
IVW Radial analysis for 'MVPA' on 'HD' (Analysis after removal of outliers.)
2
1
0
−1
−2
$\hat{\beta}_j \sqrt{W_j}$
0
4
8
12
16
$\sqrt{W_j}$
0
−0.105
Estimate
Variant
IVW

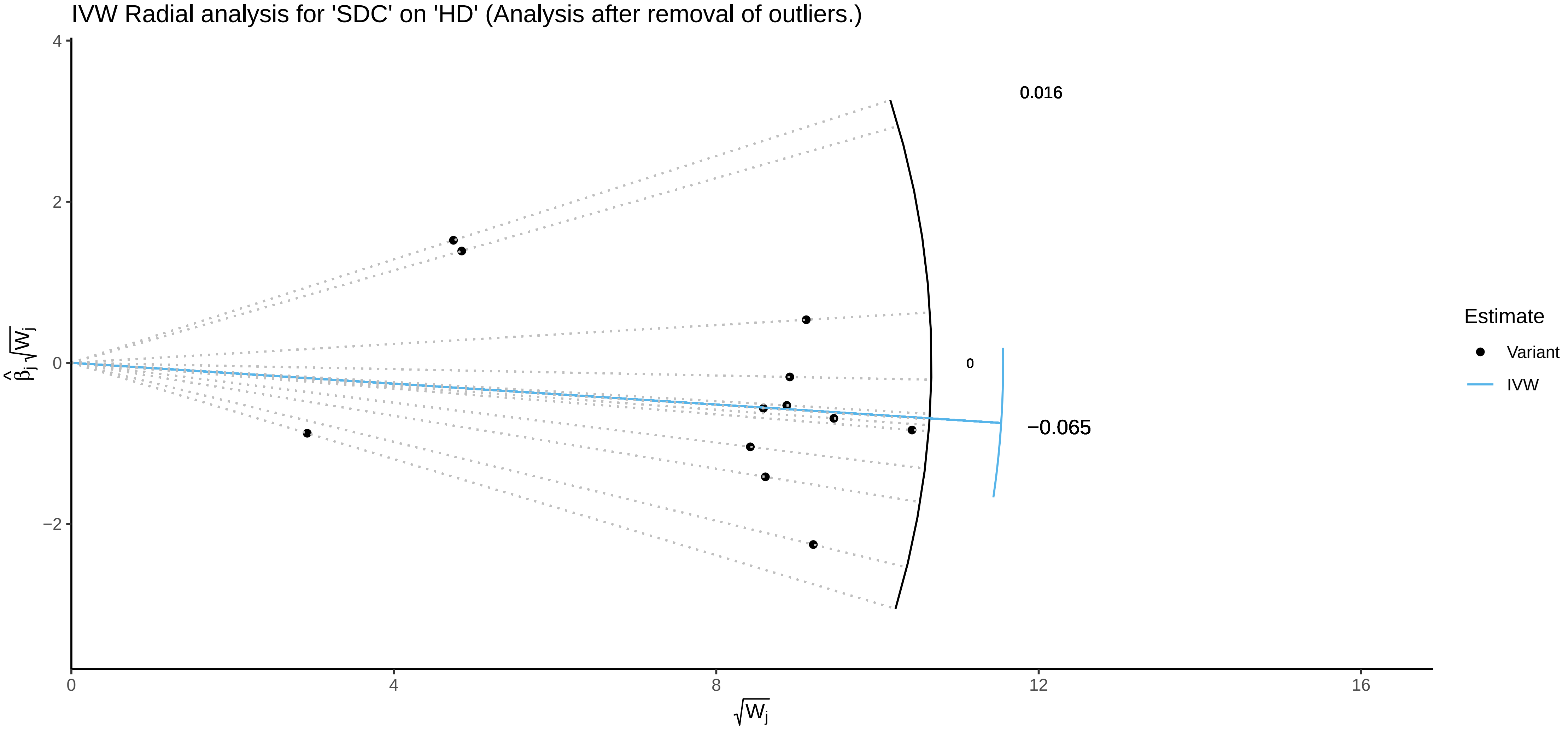

IVW Radial analysis for 'SDC' on 'HD' (Analysis after removal of outliers.)
4
2
0
−2
0.016
0
−0.065
0
4
8
12
16
Estimate
Variant
IVW

IVW Radial analysis for 'SDW' on 'HD' (Analysis after removal of outliers.)

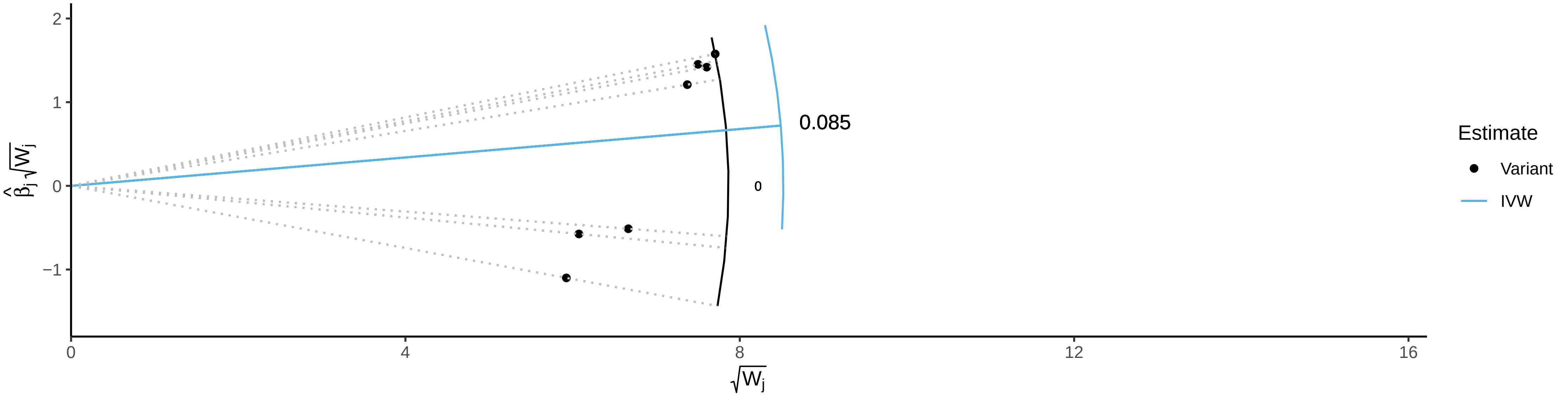

IVW Radial analysis for 'SmkInit' on 'HD' (Analysis after removal of outliers.)

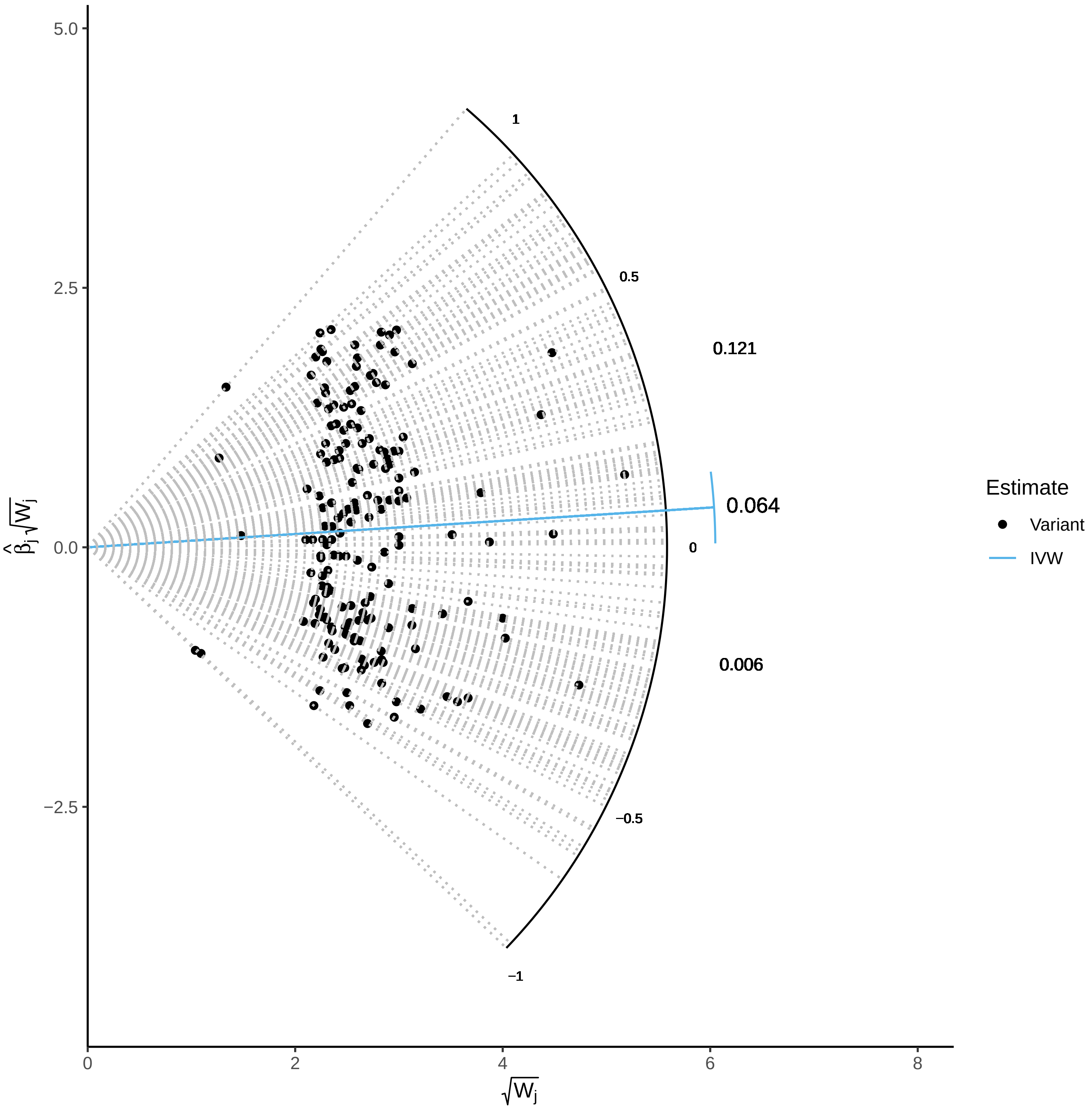

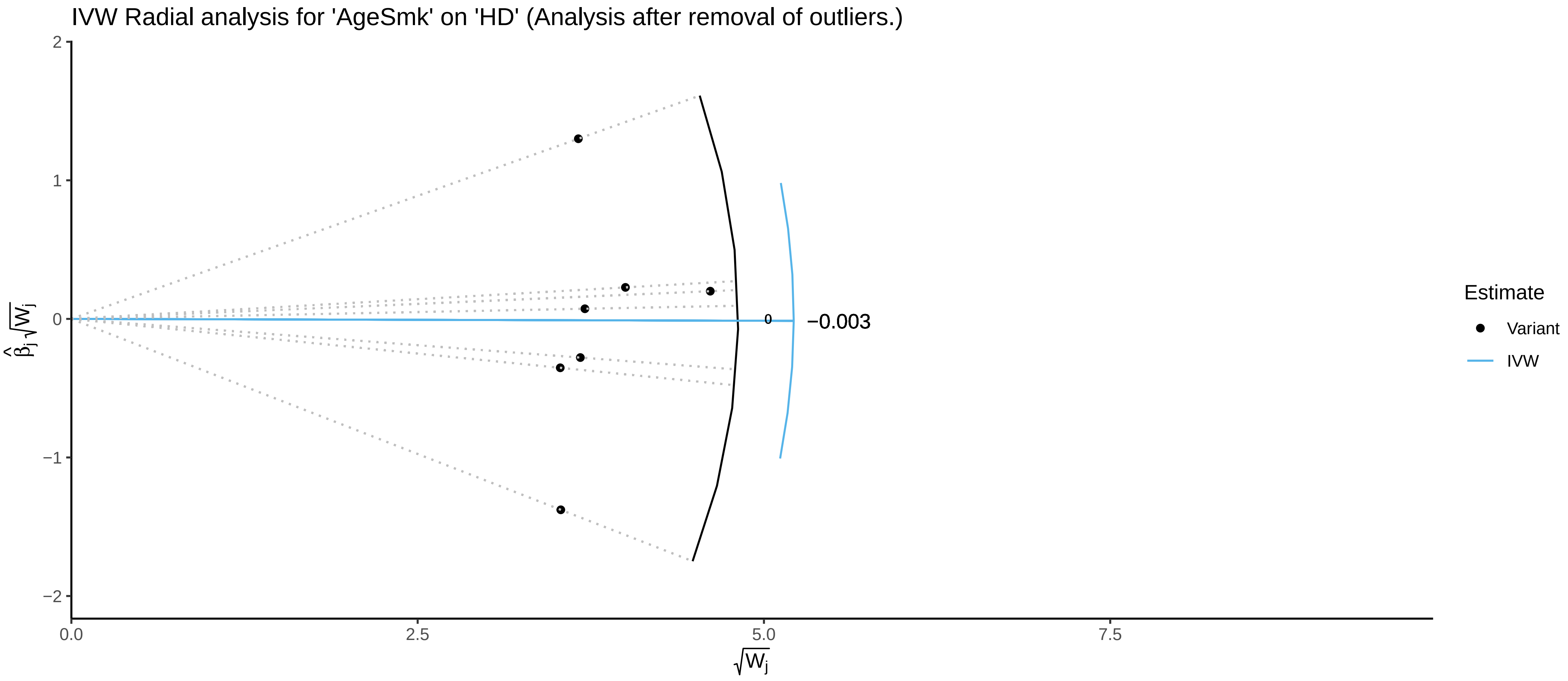

IVW Radial analysis for 'AgeSmk' on 'HD' (Analysis after removal of outliers.)
2
1
0
−1
−2
0.0
2.5
5.0
7.5
0
−0.003
$\hat{\beta}_j \sqrt{W_j}$
$\sqrt{W_j}$
Estimate
Variant
IVW

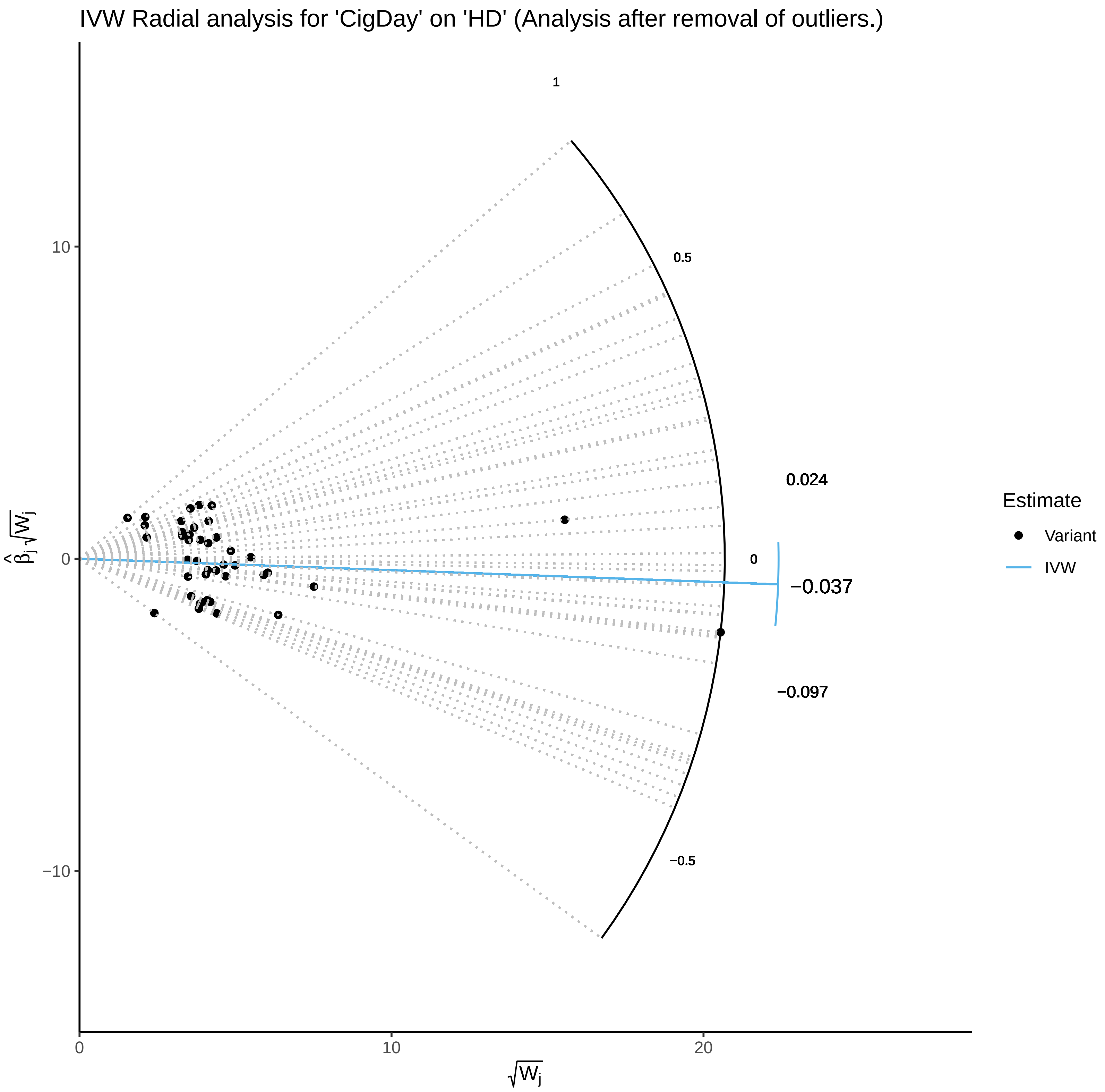

IVW Radial analysis for 'CigDay' on 'HD' (Analysis after removal of outliers.)
1
0.5
0.024
0
−0.037
−0.097
−0.5
10
0
−10
0
10
20
Estimate
Variant
IVW

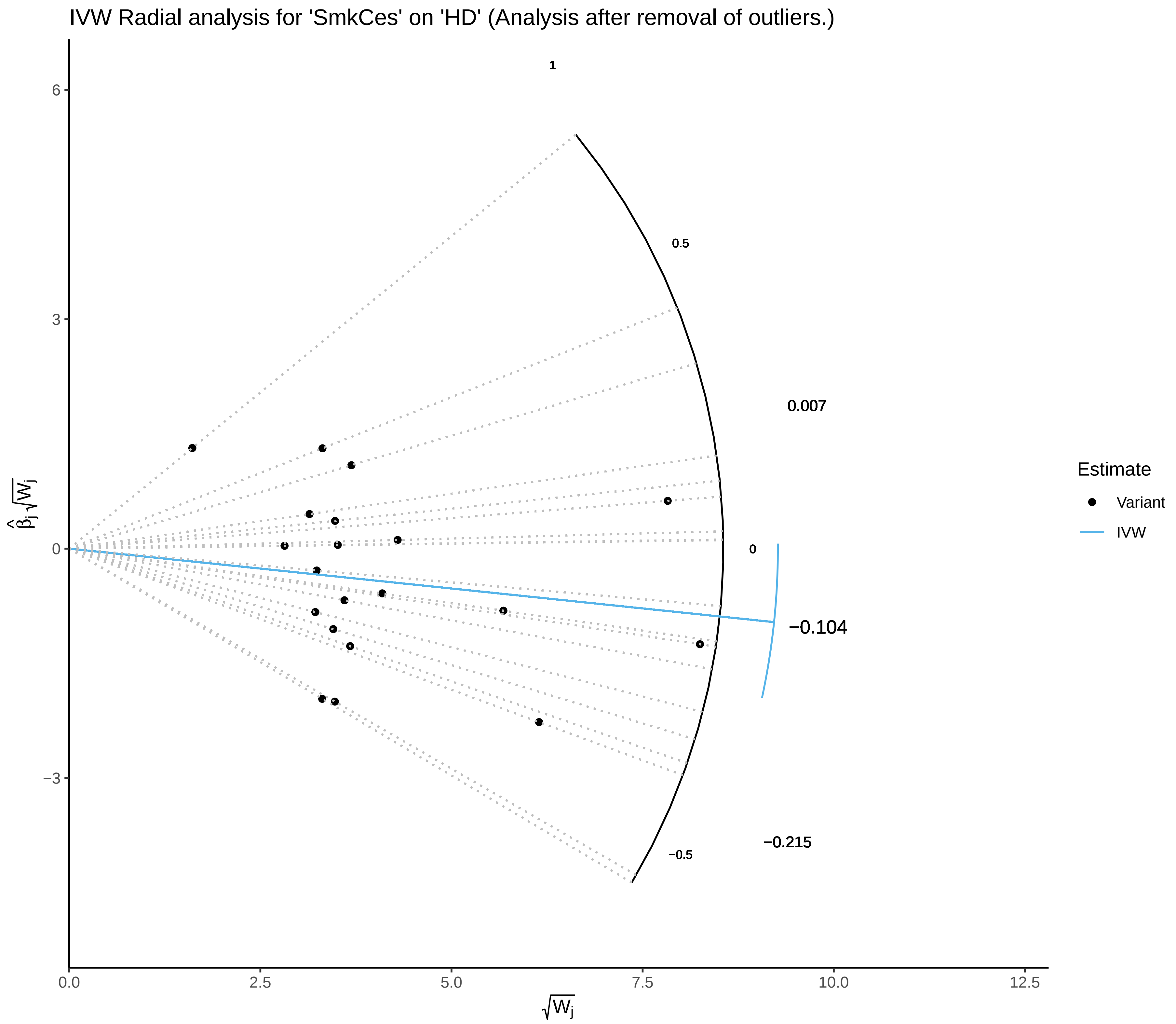

IVW Radial analysis for 'SmkCes' on 'HD' (Analysis after removal of outliers.)
1
0.5
0.007
0
−0.104
−0.215
−0.5
6
3
0
−3
0.0
2.5
5.0
7.5
10.0
12.5
Estimate
Variant
IVW

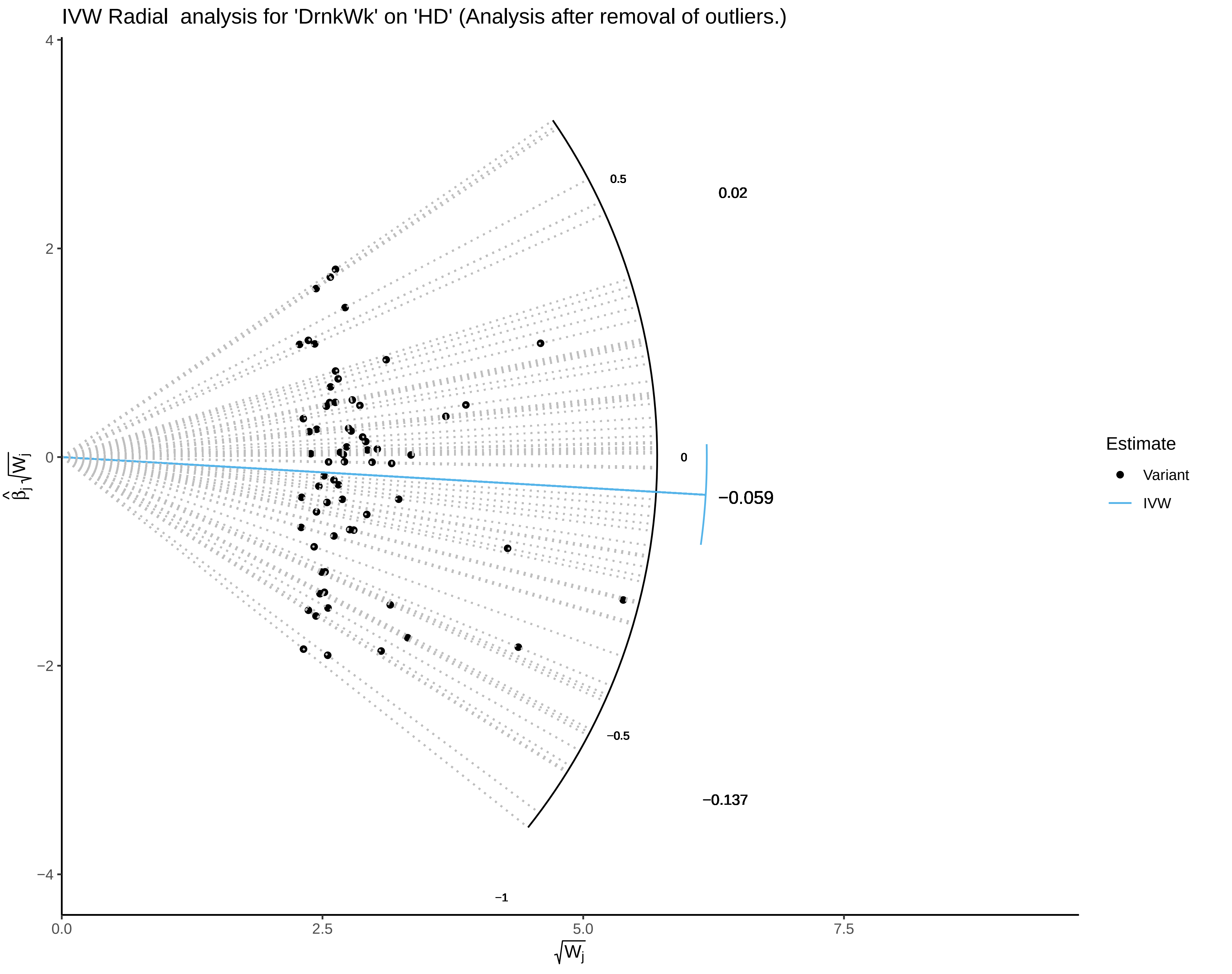
IVW Radial analysis for 'DrnkWk' on 'HD' (Analysis after removal of outliers.)
4
2
0
−2
−4
$\hat{\beta}_j \sqrt{W_j}$
0.0
2.5
5.0
7.5
$\sqrt{W_j}$
0.5
0.02
0
−0.059
−0.5
−0.137
−1
Estimate
Variant
IVW
